# Supplementary material for: The HPAfrica protocol: Assessment of health behaviour and population-based socioeconomic, hygiene behavioural factors - a standardised repeated cross-sectional study in multiple cohorts in sub-Saharan Africa
Source: BMJ Open. 2018 Dec 19;8(12):e021438. doi: 10.1136/bmjopen-2017-021438 (PMC6303690; doi:10.1136/bmjopen-2017-021438)
Supplement: Supplementary file 1 [file bmjopen-2017-021438supp001.pdf]

Appendix 1/Supplementary file 1:
Ethic References
Definitions/Terminology (English)
STROBE Statement—Checklist of items that should be included in reports of *cross-sectional studies*

Ethics References

- IVI Institutional Review Board (IRB), No. 2016-003
- Institute of Tropical Medicine Antwerp Institutional Review Board, Belgium
- Universiteit Antwerpen, Comite voor medische Ethiek, Belgium
- Ministère de la Santé du Burkina Faso – Comité d'Ethique pour la Recherche en Santé, Burkina Faso
- Comité d'Ethique de l'Ecole de Santé Publique de l'Université de Kinshasa, Democratic Republic of Congo
- National Research Ethics Review committee (NRERC) Ministry of Science and Technology, Federal Democratic Republic of Ethiopia
- AHRI-ALERT Ethics Review Committee (AAERC), Ethiopia
- Kwame Nkrumah University of Science and Technology, School of Medical Sciences/Komfo Anokye Teaching Hospital, Committee on Human Research, Publication and Ethics, Ghana
- Ministère de la Santé du Repoblikan'l Madagaskar – Comité d'Ethique
- University of Ibadan/University College Hospital Ethics Committee, Ibadan, Nigeria

Definitions/Terminology (English)

**A**	
Abdominal pain/ Intestinal pain	Pain (acute/short-lived or chronic/long-lived) that occurs anywhere between the chest and the groin (often referred to as the stomach region/belly).
Aches/Pain	A condition of having continuous/prolonged dull pain in a part of the body; the physical feeling, suffering, or discomfort caused by an illness or injury.
**B**	
Bleeding (internal/external)/ Blood loss	The condition of losing blood from the vascular system, either internally into the body (internal bleeding) or externally through a natural orifice or break in the skin (external bleeding); blood escaping from an injured blood vessel.
Blood loss/ Bleeding	The condition of losing blood from the vascular system, either internally into the body (internal bleeding) or externally through a natural orifice or break in the skin (external bleeding); blood escaping from an injured blood vessel.
Blood pressure disorders	A high/low blood pressure or a blood pressure that shows excessive fluctuation.
**C**	
Chills/Shivering	The sensation of coldness that could be accompanied by shivering or shaking of the body and pallor of the skin.
Cholera	An infectious, sometimes fatal disease of the small intestine caused by the bacterium *Vibrio cholerae*; it is spread via the fecal-oral route from the ingestion of contaminated water and food and causes chills, shivering, severe watery diarrhea, vomiting, and dehydration.
Confusion/Dizziness/ Unconsciousness	The state of being bewildered or unclear in mind; an abnormal state of response to stimuli in the environment; faint, wooziness, weakness, unsteadiness or a tendency to fall; the interruption of the awareness of oneself and one's surroundings.
Convulsion	The sudden, violent, irregular movement of a limb/part of the body or of the entire body which is caused by involuntary contraction of muscles.
Cough	The condition of expelling air from the lungs suddenly/rapidly and with a harsh noise; often involuntarily in order to clear the lung airways of fluids/mucus/other material.
**D**	

Dehydration	A condition of excessive loss of water/moisture from the body.
Diarrhea	A condition of passing feces from the body very frequently and in a liquid rather than solid form.
Diphtheria	A highly contagious, sometimes fatal disease caused by the bacterium *Corynebacterium diphtheriae*. It is spread from person-to-person, through the air or contaminated objects and affects the upper respiratory tract. Common symptoms are sore throat, swollen glands/ lymph nodes, malaise, fever/chills, a membranous substance/thick gray material that forms in the throat/tonsils and makes it difficult to breathe/ rapid breathing ("barking cough") and swallow. A form of diphtheria that involves the skin, eyes, or genitals is also reported and is characterized by pain, redness, and swelling of the skin.
Dizziness/Confusion/ Unconsciousness	The state of being bewildered or unclear in mind; an abnormal state of response to stimuli in the environment; faint, wooziness, weakness, unsteadiness or a tendency to fall; the interruption of the awareness of oneself and one's surroundings.
**E**	
Edema/Swelling	The excess collection of a watery fluid/swelling in the cavities or tissues of the body; it's commonly noticed in the hands/arms/feet/legs; volume increase of tissue.
Education - formal	A systematic, organized education model that is structured and administered according to a given set of laws and norms; formal education institutions are administratively, physically and curricularly organized and require a minimum classroom attendance from students; it involves intermediate and final assessments in order to advance to the next learning stage; it confers degrees and diplomas pursuant to a quite strict set of regulations.
**F**	
Fatigue/Malaise/ Weakness	The state of feeling tired, exhausted, or weak; a general feeling of discomfort, uneasiness, unhealthiness, or unhappiness.
Fever, any	An abnormally high body temperature (usually above 38°C).
Fever <3 days	An abnormally high body temperature (usually above 38°C) that appears continuously for less than 3 days.
Fever >3 days	An abnormally high body temperature (usually above 38°C) that lasts longer than 3 days and appears continuously.
**G**	
**H**	
Headache	Any kind of pain in any region of the head.
Healthcare - actual	Healthcare that is related to specific sign/symptom-associated disease onsets or disease onset dates.
Healthcare - general	Healthcare that is unrelated to specific sign/symptom-associated disease onsets or disease onset dates.
Healthcare facility	Places or institutions where healthcare is provided; including hospitals, clinics, healthcare centers, health posts, and specialized care centers.
Haemophilus influenzae type B	A bacterium that causes a range of illnesses and is spread by droplets through coughs and sneezing, and occurs primarily among children <5years of age; common signs are fever, headache, and stiff neck up to sepsis. It can cause ear infections, cellulitis (soft tissue infection), arthritis, upper respiratory infections, pneumonia, meningitis (potential brain damage) and epiglottitis (with airway obstructions).
Heart disorders	Any disorder that affects or is caused by the heart; conditions that involve narrowed/blocked blood vessels that can lead to heart attack, chest pain, or stroke; condition that may affect one's heart muscle, valves, rhythm, or blood pressure.
Hepatitis B	An infectious disease caused by the hepatitis B virus (HBV) that affects the liver (acute/chronic liver infection; may result in liver failure, cancer, or cirrhosis). The virus is spread from person-to-person through blood, semen, or other bodily fluids. Common symptoms are fever, malaise/weakness/fatigue, loss of appetite, nausea/ vomiting, abdominal discomfort/pain, dark-colored urine, joint pain and jaundice.
Hepatitis E	An infectious disease caused by the hepatitis E virus (HEV) that affects the liver (only acute liver infection; may result in acute liver failure). The virus is spread via the fecal-oral route through the ingestion of contaminated food and water, infected

	animals (zoonotically) and blood. Common symptoms are fever, malaise, loss of appetite, diarrhea, nausea, abdominal discomfort, dark-colored urine, joint pain and jaundice.
HIV/AIDS	An infectious disease caused by the human immunodeficiency virus (HIV) that affects/damages the immune system and results in the acquired immunodeficiency syndrome (AIDS), a chronic, potentially life-threatening condition; it is transmitted through infectious body fluids such as blood, semen, and other bodily fluids. Common symptoms of an acute/early infection are fever, fatigue, headache, aches/joint pain, rash, sore throat, swollen glands/lymph nodes, diarrhea, weight loss, oral yeast infection and shingles. The untreated disease progresses to AIDS; symptoms caused by the severely damaged immune system are night sweats, recurring fever, chronic diarrhea, lesions on tongue and in the mouth, fatigue, weight loss, and skin rushes.
Household	A person or a group of related or unrelated persons that live together in the same dwelling unit, that acknowledge one male or female adult as the head of the household, that share the same housekeeping arrangements, that are considered to constitute one unit, and that provide themselves with food or other essentials for living. A household may be located in a single housing unit (single-story building) or in a set of collective living quarters (multi-story building).
**I**	
Income/Wage	Money or another form of payment that someone receives periodically in exchange for providing a good or service. It is usually received from a job/occupation.
Influenza	An infectious, highly contagious disease of the respiratory tract that is caused by flu viruses; it is spread from person-to-person via infected saliva droplets and contaminated objects. Common symptoms are fever, aches in muscles/arms/legs, chills/sweats, headache, fatigue/weakness, runny nose/nasal congestion, sneezing, sore throat and dry persistent cough.
Intestinal pain/ Abdominal pain	Pain (acute/short-lived or chronic/long-lived) that occurs anywhere between the chest and the groin (often referred to as the stomach region/belly).
**J**	
Jaundice	A condition of yellowing of the skin/whites of the eyes.
**K/L**	
**M**	
Malaise/Fatigue/ Weakness	The state of feeling tired, exhausted, or weak; a general feeling of discomfort, uneasiness, unhealthiness, or unhappiness.
Malaria	An infectious, sometimes fatal disease caused by a parasite (*Plasmodium* spp.) that is transmitted primarily by the bite of infected mosquitoes; infection may also spread from mother-to-child prenatally or during delivery, or through contact with contaminated blood. Common symptoms are recurrent attacks of chills, sweating, high fever, headache, vomiting and diarrhea.
Measles	An infectious, sometimes fatal disease of the respiratory tract (nose/throat) that is caused by a virus that affects mainly children <5years of age; it is spread from person-to-person via infected saliva droplets and contaminated objects. Common symptoms are fever, dry cough, runny nose, inflamed eyes, sore throat, large flat spots on the skin, and white spots in the mouth and on the cheek.
Meningitis	A viral/bacterial/fungal, sometimes life-threatening infectious inflammation of the membranes (meninges) surrounding the brain and spinal cord; it is spread from person-to-person via infected saliva droplets and contaminated objects. Common symptoms are high fever, severe headache, nausea/vomiting, confusion, seizures, loss of appetite and thirst, fatigues, sensitivity to light, skin rush and stiff neck.
Mumps	A viral infectious disease caused by the mumps virus that affects mainly the parotid glands that are situated below and in front of the ears; it is spread from person-to-person via infected saliva droplets and contaminated objects. Common symptoms are fever, swelling of one/both parotid glands, headache, muscle aches, weakness/fatigue, loss of appetite and pain while shewing/swallowing; a known complication may be hearing loss.

**N**	
Nausea/Vomiting	A feeling of sickness in the stomach with an inclination to vomit; a condition of ejecting part or all of the contents of the stomach, primarily through the mouth.
Neighbor	A person (related or unrelated) living next door to a household; a neighbor does not live together with members of a household (next door) in the same dwelling unit, does not share the same housekeeping arrangements, food, or other living essentials with members of a household (next door), and does not constitute one unit with members of a household (next door).
**O**	
Occupation	A job, or means of earning a wage/living; often requires the mastery of a complex set of knowledge, tasks, duties and skills acquired through formal education and/or practical experience.
**P**	
Pain/Aches	A condition of having continuous/prolonged dull pain in a part of the body; the physical feeling, suffering, or discomfort caused by an illness or injury.
Pertussis	Pertussis, also known as whooping cough, is a highly contagious bacterial disease. Symptoms similar to a common cold like runny nose, fever and cough are followed by weeks of sever coughing and a high-pitched whooping sound or gasping when a diseased person breathes in; severe coughing my even be followed by vomiting, breaking of ribs, or extreme fatigue. Infected very young and old people may have little or no cough, or they may have periods of interrupted breathing during which they do not breath are all. Pertussis is transmitted by the bacterium *Bordetella pertussis* through coughs and sneezes of an infected person.
Pharmacy	A place where medicines (prescription or nonprescription) are prepared, preserved, compounded and dispensed.
Physician	A person skilled/trained in healing; a person educated, clinically experienced, and licensed to practice medicine.
Pneumococcus	A bacterial infection caused by the bacterium *Streptococcus pneumoniae*; a sometimes fatal inflammation of the lungs that often affects immunocompromised people; it is spread from person-to-person via infected saliva droplets and contaminated objects. Common symptoms are fever, chills, cough (with phlegm/pus), chest pain, difficulty in breathing/shortness of breath, fatigue, nausea/vomiting and diarrhea; rare symptoms are sepsis, pleural effusion, and empyema.
Polio	A viral, highly contagious, sometimes fatal infectious disease caused by the poliovirus; in its most severe form causes paralysis and difficulties in breathing; it is transmitted via the fecal-oral route through ingestion of contaminated water and food, and from person-to-person contact. Common symptoms are fever, sore throat, headache, vomiting, fatigue, back pain/neck pain/stiffness, muscle weakness/aches, meningitis, loss of reflexes, and loose/floppy limbs.
**Q**	
**R**	
Rapid breathing/ Shortness of breath	The condition of abnormally fast breathing; a rate of >20 breaths per minute for adults, >30 breaths per minute for children and >45 breaths per minute for babies.
Respondent	An adult (at the country-specific legal age of majority) household member who is a decision-maker for the entire household within the study site; thus, this adult person serves as a proxy for all household members. This person may be identified by other members of the same household as the person who is primarily involved in the daily healthcare of household members.
Rotavirus	A viral infectious disease caused by rotavirus that results predominantly in diarrhea among children <5years of age; it spreads through the fecal-oral route from person-to-person contact and contaminated objects. Common symptoms are watery diarrhea, fever, vomiting, abdominal pain and dehydration.
Rubella	A contagious, viral infectious disease caused by the rubella virus; known by its distinctive red rash; it is spread from person-to-person via infected saliva droplets), from mother-to-child prenatally via blood, and contaminated objects. Common

	symptoms are mild fever, headache, stuffy/runny nose, inflamed red eyes, enlarged tender lymph nodes at the base of the skull/back of the neck/behind the ears, joint pain and a fine pink rash that moves from the face to the trunk, arms and legs.
Runny nose	The condition when the nose (nasal and adjacent tissues and blood vessels) produces extra mucus/excess drainage.
**S**	
Self-treatment	The medication of oneself or treatment of one's own disease or condition without medical supervision or prescription.
Severe breathing/ Difficulties in breathing	The condition of abnormal breathing that may be accompanied by wheezing, chest pain, chest in-drawing, stridor, swelling in the chest, or cough for instance.
Shivering/Chills	The sensation of coldness that could be accompanied by shivering or shaking of the body and pallor of the skin.
Shortness of breath/ Rapid breathing	The condition of abnormally fast and deep breathing; a rate of >20 breaths per minute for adults, >30 breaths per minute for children and >45 breaths per minute for babies.
Sneezing	The condition of suddenly forcing air out through nose and mouth with a usual loud noise.
Swelling/Edema	A condition of an excess collection of a watery fluid/swelling in the cavities or tissues of the body; it's commonly noticed in the hands/arms/feet/legs; volume increase of tissue.
**T**	
Tetanus	Tetanus is an infection characterized by muscle spasms which commonly begins in the jaw and progresses to the rest of the body. The spasms usually last for a few minutes only and occur frequently during 3-4 weeks of the illness but can be so severe that they may cause bone fractures or death when they affect the respiratory muscles. Tetanus may be characterized by further symptoms like fever, sweating, headache, difficulties in swallowing, high blood pressure, and an increased heart rate. The disease is caused by an infection with the bacterium *Clostridium tetani* that is found in soil, salvia, dust and manure. The bacterium enters through a skin injury (e.g. cut, puncture wound) by a contaminated object. It produces toxins that interfere with muscle contractions that result in the typical spasms.
Traditional healer	A person that aims to maintain health as well as to prevent, diagnose, and improve or treat illnesses by using a sum of knowledge, skills and practices based on theories, beliefs and experiences.
Tuberculosis	A contagious, potentially serious infectious disease caused by the bacteria *Mycobacterium tuberculosis*; primarily affects the lungs, but can also affect the kidneys and spine/brain; it is transmitted from person-to-person via infected saliva droplets. Common symptoms of active tuberculosis are cough (with or without blood), chest pain, weight loss, fatigue, fever, night sweats, chills and loss of appetite.
Typhoid fever	A bacterial, potentially life-threatening infectious disease caused by *Salmonella typhi*; it is transmitted via the fecal-oral route through contaminated food and water, or from person-to-person contact. Common symptoms are high fever, headache, abdominal pain, constipation/diarrhea, and rash. Serious complications (i.e. perforation of the gut) are reported.
**U**	
Unconsciousness/ Dizziness/Confusion	The state of being bewildered or unclear in mind; an abnormal state of response to stimuli in the environment; faint, wooziness, weakness, unsteadiness or a tendency to fall; the interruption of the awareness of oneself and one's surroundings.
**V**	
Varicella	A viral, highly contagious infectious disease, commonly known as chickenpox, that primarily affects children and is caused by the varicella-zoster virus; it is spread from person-to-person by contact with infected blisters or saliva droplets. Common symptoms are mild fever, headache, backache, loss of appetite, and vesicular skin eruptions/rash/red spots on the back and chest accompanied by severe itching.

Visitor	A person (related or unrelated) who comes to a household to spend some time with household members; a visitor does not live together with household members in the same dwelling unit, does not share the same housekeeping arrangements, food, or other living essentials with household members, and does not constitute one unit with household members.
Vomiting/Nausea	A feeling of sickness in the stomach with an inclination to vomit; a condition of ejecting part or all of the contents of the stomach, primarily through the mouth.
**W**	
Weakness/Malaise/ Fatigue	The state of feeling tired, exhausted, or weak; a general feeling of discomfort, uneasiness, unhealthiness, or unhappiness.
Weight loss	The (voluntary or involuntary) decrease in body weight.
Witness	A person who is an adult (at the country-specific legal age of majority), who is independent of the study, who cannot be unfairly influenced by study staff, who attends the informed consent process if the subject or subject's legally acceptable representative is illiterate, and who reads (literate) the informed consent form and any other written information supplied to the subject.
**X/Y/Z**	
Yellow fever	An acute infectious, human pathogenic, viral disease that is marked by the sudden onset of symptoms like fever, chills, loss of appetite, nausea, muscle pain, and headache, that typically resolve within a few days or are followed by more serious symptoms such as jaundice, high fever and hemorrhage. The disease is caused by a virus of the genus Flavivirus and is transmitted by the bite of an infected mosquito.

STROBE Statement—Checklist of items that should be included in reports of ***cross-sectional studies***

	Item No	Recommendation	
**Title and abstract**	1	(*a*) Indicate the study's design with a commonly used term in the title or the abstract	√
		(*b*) Provide in the abstract an informative and balanced summary of what was done and what was found	√
**Introduction**			
Background/rationale	2	Explain the scientific background and rationale for the investigation being reported	√
Objectives	3	State specific objectives, including any prespecified hypotheses	√
**Methods**			
Study design	4	Present key elements of study design early in the paper	√
Setting	5	Describe the setting, locations, and relevant dates, including periods of recruitment, exposure, follow-up, and data collection	√
Participants	6	(*a*) Give the eligibility criteria, and the sources and methods of selection of participants	√
Variables	7	Clearly define all outcomes, exposures, predictors, potential confounders, and effect modifiers. Give diagnostic criteria, if applicable	√
Data sources/ measurement	8*	For each variable of interest, give sources of data and details of methods of assessment (measurement). Describe comparability of assessment methods if there is more than one group	√
Bias	9	Describe any efforts to address potential sources of bias	√
Study size	10	Explain how the study size was arrived at	√
Quantitative variables	11	Explain how quantitative variables were handled in the analyses. If applicable, describe which groupings were chosen and why	√
Statistical methods	12	(*a*) Describe all statistical methods, including those used to control for confounding	√
		(*b*) Describe any methods used to examine subgroups and interactions	√
		(*c*) Explain how missing data were addressed	Not

			applicable
		(*d*) If applicable, describe analytical methods taking account of sampling strategy	Not applicable
		(*e*) Describe any sensitivity analyses	Not applicable
**Results**			
Participants	13*	(a) Report numbers of individuals at each stage of study—eg numbers potentially eligible, examined for eligibility, confirmed eligible, included in the study, completing follow-up, and analysed	Not applicable
		(b) Give reasons for non-participation at each stage	Not applicable
		(c) Consider use of a flow diagram	Not applicable
Descriptive data	14*	(a) Give characteristics of study participants (eg demographic, clinical, social) and information on exposures and potential confounders	Not applicable
		(b) Indicate number of participants with missing data for each variable of interest	Not applicable
Outcome data	15*	Report numbers of outcome events or summary measures	Not applicable
Main results	16	(*a*) Give unadjusted estimates and, if applicable, confounder-adjusted estimates and their precision (eg, 95% confidence interval). Make clear which confounders were adjusted for and why they were included	Not applicable
		(*b*) Report category boundaries when continuous variables were categorized	Not applicable
		(*c*) If relevant, consider translating estimates of relative risk into absolute risk for a meaningful time period	Not applicable
Other analyses	17	Report other analyses done—eg analyses of subgroups and interactions, and sensitivity analyses	Not applicable

**Discussion**			
Key results	18	Summarise key results with reference to study objectives	Not applicable
Limitations	19	Discuss limitations of the study, taking into account sources of potential bias or imprecision. Discuss both direction and magnitude of any potential bias	Not applicable
Interpretation	20	Give a cautious overall interpretation of results considering objectives, limitations, multiplicity of analyses, results from similar studies, and other relevant evidence	Not applicable
Generalisability	21	Discuss the generalisability (external validity) of the study results	Not applicable
**Other information**			
Funding	22	Give the source of funding and the role of the funders for the present study and, if applicable, for the original study on which the present article is based	√
