# Supplementary material for: The HPAfrica protocol: Assessment of health behaviour and population-based socioeconomic, hygiene behavioural factors - a standardised repeated cross-sectional study in multiple cohorts in sub-Saharan Africa
Source: BMJ Open. 2018 Dec 19;8(12):e021438. doi: 10.1136/bmjopen-2017-021438 (PMC6303690; doi:10.1136/bmjopen-2017-021438)
Supplement: Supplementary file 2 [file bmjopen-2017-021438supp002.pdf]

Appendix 2/Supplementary file 2: Study forms (English)

FORM 1 – RECORD OF HOUSEHOLD VISITS

Instructions to the interviewer

- Complete "Form 1 – RECORD OF HOUSEHOLD VISITS" for <u>every household</u> visited.
- Complete "Home/Respondent/Completed" once <u>all survey parts</u> have been completed; if not all data haven been collected at the initial and/or a consecutive visit (max. 3 visits), schedule a revisit.
- <u>Attempt 1:</u> complete the status of the household visit using given options; for "Not Home", "Home/Non-Respondent" and "Home/Respondent/Unavailable" schedule a revisit; for "Home/Respondent/Refusal" state the reason for refusal and visit a replacement household to the nearest on the right or left side of this household; for ~~and~~ "Non-existing/Non-residential" skip this household and visit a replacement household to the nearest on the right or left side of this household instead.
- <u>Attempt 2:</u> complete the status of the household visit using given options; for "Not Home", "Home/Non-Respondent" and "Home/Respondent/Unavailable" schedule a revisit.
- <u>Attempt 3:</u> complete the status of the household visit using given options; for "Not Home", "Home/Non-Respondent", "Home/Respondent/Unavailable" skip this household and visit a replacement household to the nearest on the right or left side of this household instead.
- "Site" and "Subarea" must be entered each as a 3-digit, "House number" as a 4-digit, and "Interviewer ID" as a 2-digit code (use lists individually prepared for each site to obtain codes); enter "Date" in the format DD/MM/YY and "Time" in the format HH:MM and circle AM or PM. "Study label" must be a combination of a 3-digit "Site" code, a 3-digit "Subarea" code and a 4-digit "House number" code; the "Interviewer ID" must be a entered as a 3-digit code.

**Attempt 1**	Date ____/____/____ (DD/MM/YY)	Time _____/_____ AM/PM	Interviewer ID ___ ___
STATUS		Tick one	
1=Not home		○	➡ Schedule revisit (Date/Time)
2=Home/Non-Respondent		○	➡ Schedule revisit (Date/Time)
3=Home/Respondent/Unavailable		○	➡ Schedule revisit (Date/Time)
4=Home/Respondent/Completed		○	
5=Home/Respondent/Refusal		○	Record the reason(s) for refusal; tick all that applies<br>○ 1=No time to answer questions<br>○ 2=Don't want to participate/answer questions<br>○ 3=Other ______________________________<br>○ 98=No response ○ 99=Don't know<br>➡ Visit a replacement household to the nearest right or left side of this household
6=Non-existing/Non-residential		○	➡ Visit a replacement household to the nearest right or left side of this household
**Attempt 2**	Date ____/____/____ (DD/MM/YY)	Time _____/_____ AM/PM	Interviewer ID ___ ___
STATUS		Tick one	
1=Not home		○	➡ Schedule revisit (Date/Time)
2=Home/Non-Respondent		○	➡ Schedule revisit (Date/Time)
3=Home/Respondent/Unavailable		○	➡ Schedule revisit (Date/Time)

4=Home/Respondent/Completed		O	
5=Home/Respondent/Refusal		O	Record the reason(s) for refusal; tick all that applies<br>O 1=No time to answer questions<br>O 2=Don't want to participate/answer questions<br><br>O 3=Other ________________________________<br>O 98=No response O 99=Don't know<br><br>➡ Visit a replacement household to the nearest right or left side of this household
6=Non-existing/Non-residential		O	➡ Visit a replacement household to the nearest right or left side of this household
**Attempt 3**	Date ____/____/____ (DD/MM/YY)	Time _____/_____ AM/PM	Interviewer ID ___ ___
STATUS		Tick one	
1=Not home		O	➡ Visit a replacement household to the nearest right or left side of this household
2=Home/Non-Respondent		O	➡ Visit a replacement household to the nearest right or left side of this household
3=Home/Respondent/Unavailable		O	➡ Visit a replacement household to the nearest right or left side of this household
4=Home/Respondent/Completed		O	
5=Home/Respondent/Refusal		O	Record the reason(s) for refusal; tick all that applies<br>O 1=No time to answer questions<br>O 2=Don't want to participate/answer questions<br><br>O 3=Other ________________________________<br>O 98=No response O 99=Don't know<br><br>➡ Visit a replacement household to the nearest right or left side of this household
6=Non-existing/Non-residential		O	➡ Visit a replacement household to the nearest right or left side of this household

FORM 2 – CONSENT FORM

Healthcare assessment in [add study site and -country]

Instructions to the interviewer

- Read "Form 2 – CONSENT FORM" to the respondent/ask the respondent to carefully read through "Form 2 – CONSENT FORM" if preferred.
- The respondent as defined for this investigation is an adult (at the country-specific legal age of majority) household member and decision-maker with respect to daily healthcare/healthcare utilization for the entire household and its members; it must not be e.g. a neighbour or a visitor.
- Assure that the respondent has fully understood the purpose, procedures, risks and benefits of this study prior to obtaining the respondent's consent to participate.
- The respondent must sign "Form 2 – CONSENT FORM"; if the respondent is unable to sign/illiterate, a thumbprint will serve in place of a signature and a study-independent literate adult witness (e.g. member of same household/neighbour) will sign "Form 2 – CONSENT FORM".
- Collect data about all household members regardless of whether all household members are present at the time of the interview; collecting and recording data only about individuals present at the time of the interview will result in biased data.
- Complete "Form 2 – CONSENT FORM" once for every household consented/enrolled in the study.
- Explain to the respondent that it is anticipated to revisit every household during a 2-year period; the study team will obtain a new consent for participation during each visit.
- "Site" and "Subarea" must be entered each as a 3-digit, "House number" as a 4-digit, "Interviewer ID" as a 2-digit code (use lists individually prepared for each site to obtain codes), and "Date" in the format DD/MM/YYYY. "Study label" must be a combination of a 3-digit "Site" code, a 3-digit "Subarea" code and a 4-digit "House number" code; the "Interviewer ID" must be entered as a 3-digit code.

Study principal- and co-investigators

[add names of study principal- and co-investigators and respective affiliations]

Introduction

The International Vaccine Institute (IVI) and the [add affiliation(s) of local investigators] are working together to look into health problems and healthcare seeking behaviour in your community [add study site and -country], including the socio-economic status, hygiene and sanitation behaviour, and the vaccination status among children. We would like to better understand where you, your family and your neighbours seek healthcare in case of medical conditions associated with fever/hot body and other signs & symptoms. This information will help us to find out how common fever-related and other conditions are, provide a better picture of influencing factors involved in disease transmission and infection, and to find ways to prevent and treat illnesses in your community. You, the primary decision-maker for daily healthcare/healthcare utilization for this household and its members, are invited to participate because you live in the area where we are conducting this study. This consent form will be read by you/to you and you should take your time to make your decision to accept or decline the participation in the study. Please ask members of the study team to explain any word(s) or information that you do not understand. Further information about this study, including its potential risks, benefits and inconveniences will be explained subsequently. After the study has been fully explained to you and if you agree to participate, you will be asked to sign this consent form on behalf of all household members. If you are unable to sign the form, we will ask you to provide your thumbprint, and a study-independent adult person (member of your household/neighbour) will sign the form as a witness. We will give you a signed and dated copy of the consent form.

Purpose

The purpose of this study is to look into health problems and utilization of healthcare in your community [add study site and -country], including the socio-economic status and hygiene and sanitation behaviour. We aim to better understand where you, your family and your neighbours seek healthcare for conditions associated with fever/hot body or other sings & symptoms. This information will help better estimate the burden of illnesses common in your community, and to identify ways to introduce measures to prevent and treat them.

Procedures
We will visit about [add number for study site and -country] households in total in [add study site and -country] and will ask the same questions to each household. Your household was chosen for this study simply by chance. If you agree to participate, we will ask you on behalf of all household members a few questions about your household such as the initials, sex and age of each member. We would like to know some information related to the education and occupation of the household head, and income, possession of household items, the housing itself as well as hygiene and sanitation in the context of personal hygiene, food preparation and handling, water sources and handling, waste disposal, handling of farm and domestic animals as well as farming of this household. We will also ask where members of your household get help if anyone is sick due to fever-related and other conditions and about factors that may influence healthcare seeking behaviour of household members (i.e. travel modalities, availability of health insurance, cultural/ethnographic factors) besides the immunization status of children of this house and the perception of some diseases. Finally, we will measure the geographic location of your house. There are no right or wrong answers. You are free to choose which questions to answer. All in all, it should take about 30-45 minutes to answer all questions. The interview may take a bit longer if there are many members in this household. Also, we will visit your household two times in different seasons (once during the dry season and once towards the end of the rainy season) during the overall study period of about two years to collect data as described in this paragraph of the consent form.

Participation
You, the primary decision-maker for daily healthcare/healthcare utilization for this household and its members, and members of your household are free to choose to accept or decline to be part of the study. If you do not want to participate, that is ok. If you want to stop your participation at any time, that is ok, and there will be no harm to you or members of your household. So, participation in this study is voluntary, and you are free to refuse to participate in the study or you can withdraw your consent at any time without giving reasons and this will not involve any penalty.

Risks & Benefits
There are no direct risks for participating in this study. Asking questions about your household may make you or household members feel uncomfortable. Study teams may help providing information on established community healthcare resources for participants that express distress after completing the questions. You can refuse to answer any question at any time. You may take a break or stop participating in this study at any time. There are no direct benefits for being in this study. During visits of your household, our study teams may identify members of your household that should seek healthcare for fever-associated conditions and others. Hence, they may help providing information on established healthcare resources in your community. Indirect benefits are that all information collected will provide exact information on health problems and healthcare utilization for various conditions, including vaccination for young children, the socio-economic status as well as hygiene and sanitation behaviour in your community. This will help to provide a better picture of influencing factors involved in disease transmission and infection, and to estimate the burden of common diseases as well as to find ways to prevent and treat those in your community.

Costs & Compensation
There are no costs for participants of this study. You and anyone from your household will not be compensated for your participation.

Confidentiality
All study records and all collected information that identify you and members of your household will be kept confidential. All paper-based files will be locked safely and computerized files will be password-protected; all data files (paper-based/computerized) will be stored for a minimum of five years; these files will be accessible to authorized few study staff only. Your name or any identifier will not be used in any publication or reports from this study. Information that we collect about you and members of your household will only be shared with authorized study members such as authorized study staff and officials from ethics committees.

Site ___ ___ ___ Subarea ___ ___ ___ House number ___ ___ ___ ___ Interviewer ID ___ ___

Contact point for questions
If you or members of your household have any questions, please ask them now to the study team or at any time later by contacting [add name of local principal- and/or co-investigator, respective affiliations, and phone numbers]. If you have questions as a study participant about your/your household members' rights, you can contact the [add name and contact details of the local ethics committee].

Statement of consent
This consent form for participating in the assessment of health behaviour and other population-based factors in [add study site and -country] has been read by me/to me carefully. The purpose, procedures, risks & benefits have been explained to me in detail. I have been allowed to ask questions at any time, and my questions have been answered to my satisfaction by the study team. I have been told whom to contact if I have questions, want to discuss problems or concerns. I have been told that I will be given a signed and dated copy of this consent form. I have been reassured that all information obtained as result of this study will be kept confidential and used only for the purpose of this study and only by participating institutions.

I, the respondent, consent hereby voluntarily to participate in this study. I will follow the directions of the study team and give them my full cooperation. I understand that I have the right to withdraw from the study at any time.

RESPONDENT

Name: ____
(given name + middle name (if available) + family name)

Signature: ____
(handwritten depiction of person's name/mark that the person writes on a document as a proof of identity)

Date ______/______/_______________ (DD/MM/YYYY)

Thumbprint

If the respondent is unable to sign/illiterate but agrees to participate, record the respondent's thumb print in the above box, and have an adult study-independent literate witness (e.g. member of the same household or a neighbor) sign below:

WITNESS

Name: ____
(given name + middle name (if available) + family name)

Signature: ____
(handwritten depiction of person's name/mark that the person writes on a document as a proof of identity)

Date ______/______/_______________ (DD/MM/YYYY)

I, the interviewer, have read/explained the study to the above named respondent (witness if respondent is illiterate) in a language that the respondent understands well. I am certain that the respondent has understood the information and he/she agrees to be asked some questions.

INTERVIEWER

Name: ____
(given name + middle name (if available) + family name)

Signature: ____
(handwritten depiction of person's name/mark that the person writes on a document as a proof of identity)

Date ______/______/_______________ (DD/MM/YYYY)

FORM 3: GENERAL HOUSEHOLD INFORMATION

Instructions to the interviewer

- Complete this form only if the respondent consented to participate (see "Form 2 – CONSENT FORM"); the respondent as defined for this investigation is an adult (at the country-specific legal age of majority) household member and decision-maker with respect to daily healthcare/healthcare utilization for the entire household and its members; it must not be e.g. a neighbour or a visitor.
- Collect data about all household members regardless of whether all household members are present at the time of the interview; collecting and recording data only about individuals present at the time of the interview will result in biased data.
- A household is/household members are defined as a person or a group of related or unrelated persons that live together in the same dwelling unit, that acknowledge one male or female adult as the head of the household, that share the same housekeeping arrangements, that are considered to constitute one unit, and that provide themselves with food or other essentials for living.
- "Site" and "Subarea" must be entered each as a 3-digit, "House number" as a 4-digit, and "Interviewer ID" as a 2-digit code (use lists individually prepared for each site to obtain codes). "Study label" must be a combination of a 3-digit "Site" code, a 3-digit "Subarea" code and a 4-digit "House number" code; the "Interviewer ID" must be entered as a 3-digit code.

FORM 3: GENERAL HOUSEHOLD INFORMATION
PART A – DEMOGRAPHIC DATA/GENERAL HOUSEHOLD INFORMATION

HOUSE LOCATION

1 Site, subarea and house number

Instructions: Site (=study site) and subarea (=smallest unit of the study site) must be recorded each as a 3-digit, and house number as a 4-digit code; use individually prepared lists to obtain the respective codes.

Site: ____ ____ ____ Subarea: ____ ____ ____ House number: ____ ____ ____ ____

2 GPS coordinates

Instructions: Take GPS coordinates at the approx. center of the house. Record altitude as a 4-digit number (unit: meter), and latitude/longitude as a 7-digit number (unit: degrees, decimal minutes).

O 1=Original household O 2=Replacement household (right/left side of original household)

Altitude: ___ ___ ___ ___

Latitude: ___ ___°___ ___.___ ___ ___' Longitude: ___ ___°___ ___.___ ___ ___'

RESPONDENT

3 Provide the full name of the respondent.

Instructions: Record given name, middle name (if available) and family name of the respondent.

____

4 Provide the age of the respondent.

Instruction: Recorded as a 3-digit number (unit: year). ____ ____ ____

5 Provide the sex of the respondent.

O 1=Male O 2=Female

6 What is the respondent's relationship to this household?

O 1=Household head
O 2=Son/daughter of household head
O 3=Brother/sister of household head
O 4=Mother/father of household head
O 5=Granddaughter/grandson of household head
O 6=Other, specify _________
O 7=Spouse of household head
O 8=Spouse of son/daughter of household head
O 9=Spouse of brother/sister of household head
O 10=Mother/father in law of household head
O 11=Niece/nephew of household head
O 98=No response

HOUSEHOLD MEMBERS

7 How many household members are living in this house in total (at the day of visit)**?**

Instruction: Recorded as a 2-digit number.

Total number of household members: ____ ____

8 Record age and sex of each household member.

Instructions: Record the respondent's initials, age and sex at the first position/row of the table. Initials, age and sex of all other household members must be recorded subsequently. The initials of a household member consist of the first three letters of the given name and the first three letters of the family name (a potential middle name is not part of the initials). Record the age of each household member as a 3-digit number (unit: year); if a household member's age is ≤12 months, record the age as 1 year. Please complete an additional form if a household has >20 members.

**Household member identifier**	**Initials** [given name/family name]	**Age** [year]	**Sex**
1/Respondent	____ ____ ____/____ ____ ____	____ ____ ____	O1=Male O2=Female
2	____ ____ ____/____ ____ ____	____ ____ ____	O1=Male O2=Female
3	____ ____ ____/____ ____ ____	____ ____ ____	O1=Male O2=Female
4	____ ____ ____/____ ____ ____	____ ____ ____	O1=Male O2=Female
5	____ ____ ____/____ ____ ____	____ ____ ____	O1=Male O2=Female
6	____ ____ ____/____ ____ ____	____ ____ ____	O1=Male O2=Female
7	____ ____ ____/____ ____ ____	____ ____ ____	O1=Male O2=Female
8	____ ____ ____/____ ____ ____	____ ____ ____	O1=Male O2=Female
9	____ ____ ____/____ ____ ____	____ ____ ____	O1=Male O2=Female
10	____ ____ ____/____ ____ ____	____ ____ ____	O1=Male O2=Female
11	____ ____ ____/____ ____ ____	____ ____ ____	O1=Male O2=Female
12	____ ____ ____/____ ____ ____	____ ____ ____	O1=Male O2=Female
13	____ ____ ____/____ ____ ____	____ ____ ____	O1=Male O2=Female
14	____ ____ ____/____ ____ ____	____ ____ ____	O1=Male O2=Female
15	____ ____ ____/____ ____ ____	____ ____ ____	O1=Male O2=Female
16	____ ____ ____/____ ____ ____	____ ____ ____	O1=Male O2=Female
17	____ ____ ____/____ ____ ____	____ ____ ____	O1=Male O2=Female
18	____ ____ ____/____ ____ ____	____ ____ ____	O1=Male O2=Female
19	____ ____ ____/____ ____ ____	____ ____ ____	O1=Male O2=Female
20	____ ____ ____/____ ____ ____	____ ____ ____	O1=Male O2=Female

FORM 3: GENERAL HOUSEHOLD INFORMATION
PART B – SOCIO-ECONOMIC DATA

EDUCATION

1 Can the head of household read in [add the official language of the study site/country]**?**

O 1=Yes O 2=No O 99=Don’t know O 98=No response

2 Can the head of household write in [add the official language of the study site/country]**?**

O 1=Yes O 2=No O 99=Don’t know O 98=No response

3 What is the highest completed level of formal school education of the head of household?

O 1=Primary school O 4=Higher than secondary school O 99=Don’t know
O 2=Middle school O 5=NO EDUCATION O 98=No response
O 3=Secondary school O 6=Other, specify ___________

OCCUPATION/INCOME

4 What is the main occupation of the household head?

Instructions: Record the status of the day of visit only.

O 1=Group 1: Managers
(chief executives, senior officials, legislators; administrative and commercial managers; production and specialized services managers; hospitality, retail and other services managers)

O 2=Group 2: Professional
(science and engineering professionals; health professionals; teaching professionals; business and administration professionals; information and communications technology professionals; legal, social and cultural professionals)

O 3=Group 3: Technicians and Associate Professionals
(science and engineering associate professionals; health associate professionals; business and administration associate professionals; legal, social, cultural and related associate professionals; information and communications technicians)

O 4=Group 4: Clerical Support Workers
(general and keyboard clerks; customer services clerks; numerical and material recording clerks; other clerical support workers)

O 5=Group 5: Services and Sales Workers
(personal services workers; sales workers; personal care workers; protective services workers)

O 6=Group 6: Skilled Agricultural, Forestry, and Fishery Workers
(market-oriented skilled agricultural workers; animal producers; marked-oriented skilled forestry, fishery and hunting workers; subsistence farmers, fishers, hunters and gatherers)

O 7=Group 7: Craft and Related Trades Workers
(building and related trades workers (excluding electricians); metal, machinery and related trades workers; handicraft and printing workers; electrical and electronics trades workers; food processing, woodworking, garment and other craft and related trades workers)

O 8=Group 8: Plant and Machine Operators, and Assemblers
(stationary plant and machine operators; assemblers; drivers and mobile plant operators)

O 9=Group 9: Elementary Occupations
(cleaners and helpers; agricultural, forestry and fishery labourers; labourers in mining, construction, manufacturing and transport; food preparation assistants; street and related sales and services workers; refuse workers and other elementary workers)

O 10=Group 0: Armed Forces Occupations
(commissioned armed forces officers; non-commissioned armed forces officers; armed forces occupations, other ranks)

O 11=Other, specify __________

O 12=NO OCCUPATION O 99=Don’t’ know O 98=No response

5 What is the average monthly income of the household head based on the occupation stated in question 4 and of other household members?

Instruction: Tick one answer per household head/enter one code per household member if applicable/if income available.

Head of household	Member 1	Member 2	Member 3
Code ____________	Code ____________	Code ____________	Code ____________

[1] <100USD [3] ≥150 to <200USD [5] ≥250USD [99] Don't know
[2] ≥100 to <150USD [4] ≥200 to <250USD [6] Head of household NO INCOME (see Q4) [98] No response

6 Which and how much other/additional source(s) of monthly monetary earnings has this household?

Instruction: Tick all that applies; more than one answer is possible.

Other/additional sources	Overall monthly monetary earnings
O 1=Social grants<br>O 2=Insurance (i.e. private, public)<br>O 3=Loan (i.e. from friends, family, bank)<br>O 4=Selling (i.e. livestock, crops, furniture, other goods)<br>O 5=Personal savings<br>O 6=Other, specify __________________	O 1=<100 USD<br>O 2=≥100 to <150 USD<br>O 3=≥150 to <200 USD<br>O 4=≥200 to <250 USD<br>O 5=≥250 USD

O 7=NO OTHER/ADDITIONAL SOURCES
O 99=Don't know
O 98=No response

HOUSING/DWELLING

7 Is the house owned by the members of this household?

O 1=Yes O 3=No, supplied (i.e. government, employer) O 99=Don't know
O 2=No, rented O 4=No, other, specify _________ O 98=No response

8 How many room(s)/individual house structure(s) of this household are used for sleeping, living, cooking and bathing?

Instruction: Enter one code per category only.

Bedroom	Living room	Kitchen	Bathroom
Code ____________	Code ____________	Code ____________	Code ____________

[1] 1-2 [3] >4 [99] Don't know
[2] 3-4 [4] None [98] No response

9 What is the construction material of the floor, the walls and the roof of room(s)/individual house structure(s) of this house?

Instruction: Enter all codes that applies per category; more than one answer per category is possible.

Floor	Walls	Roof
Code ____________	Code ____________	Code ____________

[1] Brick [3] Tiles [5] Wood [7] Metal/Tin [99] Don't know
[2] Cement/Concrete [4] Mud [6] Straw/Leaves [8] Other, specify _________ [98] No response

10 Do members of this household have access to/utilize electricity in this household?

O 1=Yes O 2=No O 99=Don't know O 98=No response

11 Which main source of light have members of this household access to/are utilizing?

O 1=Electric light/Light bulb(s) O 4=Battery-powered lamp(s) O 7=Other, specify _________
O 2=Solar light/Solar lamp(s) O 5=Candle(s) O 99=Don't know
O 3=Paraffin lamp(s)/Oil lamp(s) O 6=NO SOURCE OF LIGHT O 98=No response

HOUSEHOLD ASSETS

12 Which of the following items do members of this household own?
Instructions: Tick all that applies; more than one answer is possible; record the quantity of functional items where applicable.

O 1=Radio	Quantity ____	O None	O 11=Tape/CD player	Quantity ____	O None
O 2=Television	Quantity ____	O None	O 12=Computer/Laptop	Quantity ____	O None
O 3=Internet connection	Quantity ____	O None	O 13=Mobile/Cell phone	Quantity ____	O None
O 4=Car	Quantity ____	O None	O 14=Truck/Bus	Quantity ____	O None
O 5=Motorbike	Quantity ____	O None	O 15=Bicycle	Quantity ____	O None
O 6=Oxcart/Donkey	Quantity ____	O None	O 16=Bed	Quantity ____	O None
O 7=Mattress	Quantity ____	O None	O 17=Bed net	Quantity ____	O None
O 8=Table	Quantity ____	O None	O 18=Chair	Quantity ____	O None
O 9=Refrigerator (4°C)	Quantity ____	O None	O 19=Freezer (-20°C)	Quantity ____	O None
O 10=Fan	Quantity ____	O None	O 20=Sewing machine	Quantity ____	O None

FORM 3: GENERAL HOUSEHOLD INFORMATION
Part C – Hygiene and sanitation

TOILET FACILITY

1 **To what type of toilet facility have members of this household usually access?**
Instruction: Inside means inside a room/individual house structure/building; outside means outside a room/individual house structure/building.

- O 1=Flush toilet/Water closet (inside)
- O 2=Flush toilet/Water closet (outside)
- O 3=Non-flush latrine/pit latrine (inside)
- O 4=Non-flush latrine/pit latrine (outside)
- O 5=Ventilated Improved Pit Latrine (KVIP, inside)
- O 6=Ventilated Improved Pit Latrine (KVIP, outside)
- O 7=Composting toilet (inside)
- O 8=Composting toilet (outside)
- O 9=NO toilet facility, use a bucket
- O 10=NO toilet facility, dig a hole
- O 11=NO toilet facility, free range/open defecation
- O 12=Other, specify _________
- O 99=Don't know
- O 98=No response

2 **Do members of this household usually utilize the toilet facility stated in question 1?**

- O 1=No, members cannot utilize the facility independently (i.e. infants, toddlers)
- O 2=No, members cannot utilize the facility independently (i.e. disabled, long-term/chronically diseased)
- O 3=No, members cannot utilize the facility independently (i.e. seniors)
- O 4=No, limited access (i.e. public, shared with other households/community)
- O 5=No, too far away (i.e. public, shared with other households/community)
- O 6=No, bad condition (i.e. broken, not clean)
- O 7=YES
- O 8=Other, specify _________________
- O 99=Don't know
- O 98=No response

3 **Where is the toilet facility stated in question 1 located? With how many other people is it usually shared?**

- O 1=On the property of this household (private, not shared)
- O 2=On the property of a neighbouring household (private, shared)
  - *Shared* O 1=<10 people O 2=11-24 people O 3=25-49 people O 4=>50 people O 99=Don't know O 98=No response
- O 3=In the community (public, shared)
  - *Shared* O 1=<10 people O 2=11-24 people O 3=25-49 people O 4=>50 people O 99=Don't know O 98=No response
- O 4=Other, specify _________
- O 99=Don't know
- O 98=No response

4 **How do members of this household that have/don't have access to a toilet facility usually clean their hands directly before and after urination/defection?**
Instruction: Enter one code per category only.

Clean hands before urination/defection	Clean hands after urination/defection
Code ____________	Code ____________
[1] Wash hands with soap and water<br>[2] Wash hands with water only<br>[3] Rub hands clean with some leaves/straw/ grass<br>[4] Rub hands clean with some sand<br>[5] Rub hands clean with some cloth/fabric	[6] Don't clean, not needed/not dirty<br>[7] Don't clean, nothing to clean hands nearby/on hands<br>[8] Other, specify _________<br>[99] Don't know<br>[98] No response

KITCHEN/FOOD HANDLING

5 **Do members of this household have a kitchen for cooking/preparing food/meals? Where is it located?**

- O 1=Yes, inside kitchen/in a room/individual house structure; on the property of this household (private, not shared)
- O 2=Yes, outside kitchen/outside a room/individual house structure/ open air; on the property of this

household (private, not shared)
- O 3=Yes, inside kitchen/in a room/individual house structure; on the property of a neighbouring household (private, shared)
- O 4=Yes, outside kitchen/outside a room/individual house structure/ open air; on the property of a neighbouring household (private, shared)
- O 5=NO KITCHEN
- O 6=Other, specify __________ O 99=Don't know O98=No response

6 What energy source do members of this household usually use for cooking/preparing food/meals?

O 1=Electricity O 3=Charcoal O 5=Animal dung O 99=Don't know
O 2=Gas/Kerosene O 4=Wood/straw/leaves O 6=Other, specify _____O 98=No response

7 What cooking spot do members of this household usually use for cooking/preparing food/meals?

O 1=Open fire O 3=Closed stove with chimney O 99=Don't know
O 2=Open stove O 4=Other, specify __________ O 98=No response

8 Where do members of this household usually store fresh/raw food items (e.g. vegetables, lettuce, fruits, dairy products, eggs, meat and fish) and left-over/prepared food/meals?

Instruction: Enter one code per category only. Inside means inside a room/individual house structure/building; outside means outside a room/individual house structure/building.

Fresh/raw food items (i.e. vegetables, lettuce, fruits, dairy products, eggs, meat and fish)	Left-over/prepared food/meals
Code _____________	Code _____________
[1] At room temperature (inside, on the property of this household, private)<br>[2] At room temperature (outside, on the property of this household, private)<br>[3] At room temperature (inside, on the property of a neighboring household, private, shared)<br>[4] At room temperature (outside, on the property of a neighboring household, private, shared)<br>[5] Refrigerator (on the property of this household, private)<br>[6] Refrigerator (on the property of a neighboring household, private, shared)<br>[7] Freezer (on the property of this household, private)<br>[8] Freezer (on the property of a neighboring household, private, shared)<br>[9] Other, specify ______________<br>[10] NO STORAGE [99] Don't know [98] No response	

9 If the answer is "Room temperature" for fresh/raw food items and/or left-over/ prepared food/meals in question 8, how long do members of this household usually store them?

Instruction: Enter one code per category only.

Fresh/raw food items (i.e. vegetables, lettuce, fruits, dairy products, eggs, meat and fish)	Left-over/prepared food/meals
Code _____________	Code _____________
[1] <1 day [3] 3-4 days [5] Not applicable [99] Don't know<br>[2] 1-2 days [4] >4 days [98] No response	

10 Do members of this household usually cover left-over/prepared food/meals? Do they re-heat/re-cook left-over/prepared food/meals prior to consumption?

Instruction: Enter one code per category only.

Cover left-over/prepared food/meals	Re-heat/Re-cook left-over/prepared food/meals
Code _____________	Code _____________
[1] Yes [2] No [99] Don't know [98] No response	

11 Which member(s) of this household usually cook(s)/prepare(s) food/meals for other members of this household?

Instructions: Refer to question 9 of "Form 3 Part A – Demographic data/General household information" and record the initials/identifier(s) of the member.

O Household member, record initials/identifier(s) _________ O 99=Don't know O 98=No response

12 How does/do the household member(s) that usually cook(s)/prepare(s) food/meals for other members of this household as stated in question 11 usually prepare(s) herself/himself/themselves before and after the preparation of meals?

Instruction: Enter one code per category only.

Before the preparation of meals	After the preparation of meals
Code ____________	Code ____________
[1] Wash hands with soap and water<br>[2] Wash hands with water only<br>[3] Rub hands clean with some leaves/straw/ grass<br>[4] Rub hands clean with some sand<br>[5] Rub hands clean with some cloth/fabric	[6] Don't clean, not needed/not dirty<br>[7] Don't clean, nothing to clean hands nearby/on hands<br>[8] Other, specify _________<br>[99] Don't know<br>[98] No response

13 How do members of this household usually prepare themselves before and after eating food/meals, including the household member that usually cooks/prepares food/meals as stated in question 11?

Instruction: Enter one code per category only.

Before the eating of food/meals	After the eating of food/meals
Code ____________	Code ____________
[1] Wash hands with soap and water<br>[2] Wash hands with water only<br>[3] Rub hands clean with some leaves/straw/ grass<br>[4] Rub hands clean with some sand<br>[5] Rub hands clean with some cloth/fabric	[6] Don't clean, not needed/not dirty<br>[7] Don't clean, nothing to clean hands nearby/on hands<br>[8] Other, specify _________<br>[99] Don't know<br>[98] No response

14 How do members of this household usually eat their food/meals, including the household member that usually cooks/prepares food/meals as stated in question 11?

O 1=With cutlery O 3=Other, specify ____________ O 99=Don't know
O 2=With fingers O 98=No response

15 Do members of this household usually eat their food/meals from the same pot, including the household member that usually cooks/prepares food/meals as stated in question 11?

O 1=Yes O 2=No O 99=Don't know O 98=No response

16 Where do members of this household usually get their main fresh/raw food items such as vegetables, fruits and grains/cereals, dairy products, eggs, meat and fish?

Instruction: Enter one code per category only.

Vegetables, fruits, grains/ cereals	Dairy products	Eggs, meat, fish
Code ____________	Code ____________	Code ____________
[1] Self-farming (i.e. back-/courtyard)<br>[2] Supermarket/Grocery store<br>[3] Friend/Family<br>[4] Open market<br>[5] Street vendor	[6] Self-farming (i.e. agricultural land)<br>[7] Butcher<br>[8] Other, specify _____________<br>[99] Don't know<br>[98] No response	

17 Do members of this household usually consume/eat fresh/raw food items (e.g. vegetables, lettuce, fruits, dairy products, ice cream, eggs, meat and fish)? If so, which ones?

Instruction: Tick all that applies; more than one answer is possible.

- O 1=Vegetables/Lettuce (cultivated on bush)
- O 2=Vegetables/Lettuce (wild harvesting from bush)
- O 3=Fruits (cultivated on bush/tree)
- O 4=Fruits (wild harvesting from bush/tree)
- O 5=Dairy products (i.e. milk, cheese, yoghurt from domesticated milk producing animals), specify ____________
- O 6=Eggs (from domesticated poultry), specify _________
- O 7=Animal blood (from domesticated animals), specify _________
- O 8=Beef/Zebu/Buffalo (domesticated), specify _________
- O 9=Pork (domesticated)
- O 10=Sheep (domesticated)
- O 11=Meat from non-domesticated animals (i.e. antelope, boar, poultry, rodents, fish), specify ________
- O 12=NO CONSUMPTION of fresh/raw food items
- O 13=Vegetables/Lettuce (cultivated on ground)
- O 14=Vegetables/Lettuce (wild harvesting from ground)
- O 15=Fruits (cultivated on ground)
- O 16=Fruits (wild harvesting from ground)
- O 17=Dairy products (i.e. milk, cheese, yoghurt from non-domesticated milk producing animals), specify ____________
- O 18=Eggs (from non-domesticated poultry), specify _________
- O 19=Animal blood (from non-domesticated animals), specify _________
- O 20=Poultry (domesticated; i.e. chicken, duck, pigeon, goose, turkey), specify _________
- O 21=Goat (domesticated)
- O 22=Rodents (domesticated), specify _________
- O 23=Fish/Seafood, specify _________
- O 24=Ice cream
- O 25=Other, specify _________
- O 99=Don't know O 98=No response

18 Do members of this household consume meals/food items/beverages usually also outside the house? If so, which member(s) of this household and where?

Instructions: Refer to question 9 of "Form 3 Part A – Demographic data/General household information" and record the initials/identifier of the member(s). Enter all codes that apply for the respective household member.

Member 1<br>Initials/Identifier: _____	Member 2<br>Initials/Identifier: _____	Member 3<br>Initials/Identifier: _____	Member 4<br>Initials/Identifier: _____
Code ____________	Code ____________	Code ____________	Code ____________
[1] Restaurant<br>[2] Fast food chain	[3] Cafeteria<br>[4] Street vendor	[5] Other, specify ____<br>[6] Not applicable	[99] Don't know<br>[98] No response

19 Which meals/food items/beverages do the member(s) of this household listed in question 18 usually consume outside the house?

Instructions: Refer to question 9 of "Form 3 Part A – Demographic data/General household information" and record the initials/identifier of the member(s). List up to 5 meals/food items/beverages per household member. You can refer to food items listed in question 17. Skip this question of no household member consumes meals/food items/beverages outside the house as stated in Q18.

Member 1<br>Initials/Identifier: _____	Member 2<br>Initials/Identifier: _____	Member 3<br>Initials/Identifier: _____	Member 4<br>Initials/Identifier: _____
1: ______________<br>2: ______________<br>3: ______________<br>4: ______________<br>5: ______________	1: ______________<br>2: ______________<br>3: ______________<br>4: ______________<br>5: ______________	1: ______________<br>2: ______________<br>3: ______________<br>4: ______________<br>5: ______________	1: ______________<br>2: ______________<br>3: ______________<br>4: ______________<br>5: ______________

WATER

20 What is the main water supply for members of this household for drinking, cooking/ preparing food/meals, bathing, and cleaning (of house/individual house structure(s), clothing/kitchen tools)?

Instruction: Enter one code per category only.

Drinking	Cooking	Bathing	Cleaning
Code ____________	Code ____________	Code ____________	Code ____________
[1] Tap water (inside the house; private)<br>[2] Tap water (outside the house i.e. back-/courtyard; private)			

[3] Tap water (outside the house; public/shared)
[4] Standpipe/Piped water (inside the house; private)
[5] Standpipe/Piped water (outside the house i.e. back-/ courtyard; private)
[6] Standpipe/Piped water (outside the house; public/shared)
[7] Water truck (public; shared)
[8] Surface water (i.e. canal, irrigation canal)
[9] Surface water (i.e. lake, pond, river, stream)
[10] Spring water
[11] Rain water
[12] Well/Borehole (open/uncovered/unprotected)
[13] Well/Borehole (covered/protected)
[14] Bottled water
[15] Other, specify ___________________ [99] Don't know [98] No response

21 Do members of this household usually treat water for drinking, cooking/ preparing food/meals, bathing, and cleaning (of house/individual house structure(s), clothing/ kitchen tools) prior to consumption? How is water usually treated?

Instruction: Enter all codes that applies per category; more than one answer per category is possible.

Drinking	Cooking	Bathing	Cleaning
Code ____________	Code ____________	Code ____________	Code ____________

[1] No, not at all
[2] No, only for sick members of this household
[3] No, only depending on the season (dry season)
[4] Yes, filtration (i.e. cloth, ceramic, sand)
[5] Yes, sedimentation
[6] Other, specify ___________________
[7] No, only for children of this household
[8] No, only depending on the season (rainy season)
[9] Yes, boiling
[10] Yes, solar disinfection
[11] Yes, chlorination
[99] Don't know [98] No response

22 Which member(s) of this household usually collect(s) the drinking water as stated in question 20 for other members of this household?

Instructions: Refer to question 9 of "Form 3 Part A – Demographic data/General household information" and record the initials/identifier of the member.

O Household member, record initials/identifier(s) _________ O 99=Don't know O 98=No response

23 How do members of this household usually store the drinking water stated in question 20?

O 1=Water tank (uncovered)
O 2=Water tank (covered with i.e. cloth/fabric, lid, leaves)
O 3=Container (uncovered)
O 4=Container (covered with i.e. cloth/fabric, lid, leaves)
O 5=Clay pot (uncovered)
O 6=Clay pot (covered with i.e. cloth/fabric, lid, leaves)
O 7=Bucket (uncovered)
O 8=Bucket (covered with i.e. cloth/fabric, lid, leaves)
O 9=Bowl (uncovered)
O 10=Bowl (covered with i.e. cloth/fabric, lid, leaves)
O 11=Wooden pot (uncovered)
O 12=Wooden pot (covered with i.e. cloth/fabric, lid, leaves)
O 13=Skin vessel (i.e. goat, sheep, cattle; uncovered)
O 14=Skin vessel (i.e. goat, sheep, cattle; covered with i.e. cloth/fabric, lid, leaves)
O 15=NOT STORED
O 16=Other, specify _________
O 99=Don't know
O 98=No response

24 If members of this household use a shared drinking water supply as stated in question 20, with how many other people is this drinking water supply usually shared?

O 1=<10 people
O 2=11-24 people
O 3=25-49 people
O 4=>50 people
O 5=NOT SHARED
O 99=Don't know
O 98=No response

WASTE DISPOSAL

25 Where do members of this household usually dispose waste water from e.g. cooking/ preparation of food/meals, bathing and/or cleaning, dispose domestic waste, human waste/excreta?

Instruction: Enter one code per category only.

Waste water from (e.g. cooking/ preparation of food/ meals, bathing and/or cleaning)	Domestic waste	Human waste/excreta
Code ____________	Code ____________	Code ____________

[1] On the ground/free range near the house (i.e. back-/courtyard, private)
[2] On the ground/free range away from the house in the community (shared, public)
[3] In a hole near the house (i.e. back-/courtyard, private)
[4] In a hole away from the house in the community (shared, public)
[5] Pour in surface water in the community (i.e. lake, pond, river, stream, public, shared)
[6] Pour in surface water in the community (i.e. canal, irrigation canal, public, shared)
[7] Pour in spring water in the community (public, shared)
[8] Pour in a septic tank near the house (i.e. back-/courtyard, near toilet facility if available, private)
[9] Pour in a septic tank in the community (shared, public)
[10] Pour in the sewer near the house (i.e. back-/courtyard, private)
[11] Pour in the sewer in the community (shared, public)
[12] Other, specify ________________________ [99] Don't know [98] No response

26 Do members of this household usually pre-process domestic waste from this house prior to disposing it?

- O 1=Yes, burning garbage
- O 2=Yes, separate/sort out degradable from non-degradable garbage
- O 3=NO
- O 4=Yes, other, specify _________
- O 99=Don't know
- O 98=No response

27 How far from the main drinking water supply of this household as stated in question 20 is the location for disposal of waste water, domestic waste and human waste/excreta of this household as stated in question 25?

Instructions: Enter one code per category only. Record the distance as meter [m].

Waste water from (e.g. cooking/ preparation of food/ meals, bathing and/or cleaning)	Domestic waste	Human waste/excreta
Code ____________	Code ____________	Code ____________

[1] <5 m [3] ≥25 to <50 m [5] ≥100 to <500 m [7] ≥1,000 m
[2] ≥5 to <25 m [4] ≥50 to <100 m [6] ≥500 to <1,000 m [99] Don't know [98] No response

28 How far from the main water supply for cooking/preparing food/meals of this household as stated in question 20 is the location for disposal of waste water, domestic waste and human waste/excreta of this household as stated in question 25?

Instructions: Enter one code per category only. Record the distance as meter [m].

Waste water from (e.g. cooking/preparation of food/ meals, bathing and/or cleaning)	Domestic waste	Human waste/excreta
Code ____________	Code ____________	Code ____________

[1] <5 m [3] ≥25 to <50 m [5] ≥100 to <500 m [7] ≥1,000 m
[2] ≥5 to <25 m [4] ≥50 to <100 m [6] ≥500 to <1,000 m [99] Don't know [98] No response

FARM/DOMESTIC ANIMALS

29 What kind of farm animals/livestock and domestic animals/pets do members of this household have?

Instruction: Tick all that applies per category; more than one answer per category is possible.

Farm animals/livestock		Domestic animals/pets
O 1=Cattle	O 9=Camel	O 1=Dog
O 2=Sheep	O 10= Rodents (i.e. rabbit), specify ____________________	O 2=Rodents (i.e. rabbit), specify ____________________
O 3=Donkey	O 11=Goat	O 3=Cat
O 4=Poultry (i.e. chicken, duck, pigeon, goose, turkey), specify ______________	O 12=Horse	O 4=NO DOMESTIC ANIMALS/ PETS
O 5=Zebu	O 13=NO FARM ANIMALS/ LIVESTOCK	O 5=Other, specify ____________________
O 6=Pig	O 14=Other, specify ____________________	O 99=Don't know
O 7=Fish	O 99=Don't know	O 98=No response
O 8=Buffalo	O 98=No response	

30 Where do members of this household usually keep farm animals/livestock and domestic animals/pets of this household as stated in question 29?
Instruction: Tick one answer per category only.

Farm animals/livestock	Domestic animals/pets
O 1=Stable/Cage/Water basin on the property of the house (i.e. back-/courtyard)	O 1=Cage/Chain near the house (i.e. back-/courtyard)
O 2=Stable/Cage/Water basin outside the house in the community	O 2=Cage/Chain outside the house in the community
O 3=Stable/Cage/Water basin outside the community	O 3=Cage/Chain outside the community (public)
O 4=Free range/Surface water/Spring water on the property of the house (i.e. house, back-/courtyard)	O 4=Free range near the house (i.e. in the house, back-/courtyard)
O 5=Free range/Surface water/Spring water outside the house in the community	O 5=Free range outside the house in the community
O 6=Free range/Surface water/Spring water outside the community	O 6=Free range outside the community
O 7=Not applicable (nowhere/no farm animals/livestock)	O 7=Not applicable (nowhere/no domestic animals/pets)
O 8=Other, specify _________	O 8=Other, specify _________
O 99=Don't know	O 99=Don't know
O 98=No response	O 98=No response

31 What is the extent of the livestock farming that members of this household practice?

O 1=Own demand ("backyard farming")
O 2=Not applicable (no livestock farming)
O 3=Mass/Industrial demand ("mass animal farming")
O 4=Other, specify _________
O 99=Don't know
O 98=No response

32 Which water supply do members of this household usually use to water farm animals/livestock and domestic animals/pets of this household as stated in question 29?
Instruction: Enter one code per category only.

Farm animals/livestock	Domestic animals/pets
Code ____________	Code ____________

[1] Tap water (inside the house; private)
[2] Tap water (outside the house i.e. back-/courtyard; private)
[3] Tap water (outside the house; public/shared)
[4] Standpipe/Piped water (inside the house; private)
[5] Standpipe/Piped water (outside the house i.e. back-/ courtyard; private)
[6] Standpipe/Piped water (outside the house; public/shared)

[7] Water truck (public; shared)
[8] Surface water (i.e. canal, irrigation canal)
[9] Surface water (i.e. lake, pond, river, stream)
[10] Spring water
[11] Rain water
[12] Well/Borehole (open/uncovered/unprotected)
[13] Well/Borehole (covered/protected)
[14] Not applicable (no farm animals/livestock; no domestic animals/pets)
[15] Other, specify ___________________ [99] Don't know [98] No response

33 Do members of this household usually wash their hands after they fed & watered and cleaned the stable/cage/water basin of the farm animals/livestock and domestic animals/pets of this household as stated in question 29?
Instruction: Enter one code per category only.

Farm animals/livestock	Domestic animals/pets
Code _____________	Code _____________
[1] Wash hands with soap and water<br>[2] Wash hands with water only<br>[3] Rub hands clean with some leaves/straw/ grass<br>[4] Rub hands clean with some sand<br>[5] Rub hands clean with some cloth/fabric<br>[6] Don't clean, not needed/not dirty	[7] Don't clean, nothing to clean hands nearby/on hands<br>[8] Other, specify _________<br>[9] Not applicable (no farm animals/livestock; no domestic animals/pets)<br>[99] Don't know<br>[98] No response

CROP FARMING

34 What kind of crops do members of this household grow/cultivate?
Instruction: Tick all that applies; more than one answer is possible.

- O 1=Coffee
- O 2=Rice
- O 3=Fruits (cultivated on bush/tree)
- O 4=Fruits (cultivated on the ground)
- O 5=Vegetables/Lettuce (cultivated on bush/tree)
- O 6=Vegetables/Lettuce (cultivated on the ground)
- O 7=Grains (i.e. wheat, barley, oat, corn)
- O 8=Tea
- O 9=Cashew
- O 10=Tobacco
- O 11=Sugar cane
- O 12=Chad/Chat
- O 13=Bees/Honey
- O 14=Cotton
- O 15=NO CROPS
- O 16=Other, specify __________
- O 99=Don't know
- O 98=No response

35 Where do members of this household have the farmland/agricultural land for crop farming?

- O 1=On the property of the house (i.e. back-/courtyard)
- O 4=Outside the house in the community
- O 2=Outside the community
- O 5=Not applicable (no crop farming)
- O 3=Other, specify __________
- O 99=Don't know
- O 98=No response

36 What is the extent of the crop farming that members of this household practice?

- O 1=Own demand ("backyard farming")
- O 2=Not applicable (no crop farming)
- O 3=Mass/Industrial demand ("mass crop farming")
- O 4=Other, specify __________
- O 99=Don't know
- O 98=No response

37 Do members of this household usually use herbicides, pesticides and fertilizers for the crop farming as stated in question 34?
Instruction: Tick one answer per category only.

Herbicides	Pesticides	Fertilizers
O 1=Yes, specify herbicide ___________________________	O 1=Yes, specify pesticide ___________________________	O 1=Yes, specify fertilizer ___________________________

O 2=Not applicable (no crop farming)<br>O 3=No use of herbicides<br>O 99=Don't know<br>O 98=No response	O 2=Not applicable (no crop farming)<br>O 3=No use of pesticides<br>O 99=Don't know<br>O 98=No response	O 2=Not applicable (no crop farming)<br>O 3=No use of fertilizers<br>O 99=Don't know<br>O 98=No response

38 Do members of this household usually wash their hands after the fieldwork for the crop farming as stated in question 34?

O 1=Yes, wash hands with soap and water
O 2=Yes, rub hands clean with some leaves/straw/grass
O 3=Yes, rub hands clean with some cloth/fabric
O 4=Not applicable (no crop farming)
O 5=Yes, wash hands with water only
O 6=Yes, rub hands clean with some sand
O 7=No, don't clean my hands
O 8=Other, specify __________
O 99=Don't know
O 98=No response

FORM 4: ASSESSMENT OF OVERALL, AGE- AND SEX-STRATIFIED GENERIC HEALTHCARE UTILIZATION

Part A – Assessment of *generic* healthcare utilization

Instructions to the interviewer

- Complete this form only if the respondent consented to participate (see "Form 2 - CONSENT FORM"); the respondent as defined for this investigation is an adult (at the country-specific legal age of majority) household member and decision-maker with respect to daily healthcare/healthcare utilization for the entire household and its members; it must not be e.g. a neighbour or a visitor.
- Refer to "Form 3 – Part A - GENERAL HOUSEHOLD INFORMATION" as a reference to obtain the total number of household members as well as age and sex of each household member.
- Collect data about all household members regardless of whether all household members are present at the time of the interview; collecting and recording data only about individuals present at the time of the interview will result in biased data.
- A household is/household members are defined as a person or a group of related or unrelated persons that live together in the same dwelling unit, that acknowledge one male or female adult as the head of the household, that share the same housekeeping arrangements, that are considered to constitute one unit, and that provide themselves with food or other essentials for living.
- "Site" and "Subarea" must be entered each as a 3-digit, "House number" as a 4-digit, and "Interviewer ID" as a 2-digit code (use lists individually prepared for each site to obtain codes). "Study label" must be a combination of a 3-digit "Site" code, a 3-digit "Subarea" code and a 4-digit "House number" code; the "Interviewer ID" must be entered as a 3-digit code.
- Abbreviation: HCF=private/public primary/secondary/tertiary healthcare facility.

HOUSEHOLD MEMBERS

1 **Record the total number of household members of this house** (at the day of visit)**.**
Instructions: The total number of household members must be recorded as a 2-digit number; see also "Form 3 – Part A - GENERAL HOUSEHOLD INFORMATION".

Total number of household members: ____ ____

2 **Record the number of FEMALE household members by each of the following age groups:**
Instructions: The total number of female household members by age group must be recorded as a 2-digit number; see also "Form 3 – Part A - GENERAL HOUSEHOLD INFORMATION".

<2years: ____ ____ ≥2 to <5years: ____ ____ ≥5 to <15years: ____ ____ ≥15years: ____ ____

3 **Record the number of MALE household members by each of the following age groups:**
Instructions: The total number of male household members by age group must be recorded as a 2-digit number; see also "Form 3 – Part A - GENERAL HOUSEHOLD INFORMATION".

<2years: ____ ____ ≥2 to <5years: ____ ____ ≥5 to <15years: ____ ____ ≥15years: ____ ____

HEALTHCARE SEEKING BEHAVIOR

4 **Record where female/male household members <2years/≥2 to <5years/≥5 to <15years/≥15years of age usually seek healthcare for the following signs/symptoms. Please rate using a range from 1-4.**

Instructions:

- Complete question 4 separately for female and male household members <2years, ≥2 to <5years, ≥5 to <15years and ≥15years of age and circle the sex and age group Q4 was completed for; skip question 4 if there is no female/male household member <2years/ ≥2 to <5years/ ≥5 to <15years/ ≥15years of age.
- Provide an answer to each healthcare option for each sign/symptom; use range 1-4 for rating (1=1st choice; 2=2nd choice; 3=3rd choice; 4=not chosen); enter a code where applicable by using prepared lists (use lists individually prepared for each site to obtain codes); tick "Don't know" if applicable.

Site ___ ___ ___ Subarea ___ ___ ___ House number ___ ___ ___ ___ Interviewer ID ___ ___

Sign/Symptom	Healthcare options									
	Study-HCF		Other HCF		Physician	Pharmacy	Traditional healer	Nowhere Self-treatment	Nowhere Nothing	Don't know
	Rate	Code	Rate	Code	Rate	Rate	Rate	Rate	Rate	Tick
**1** [**A**] Fever, any	○=1<br>○=2<br>○=3<br>○=4		○=1<br>○=2<br>○=3<br>○=4		○=1<br>○=2<br>○=3<br>○=4	○=1<br>○=2<br>○=3<br>○=4	○=1<br>○=2<br>○=3<br>○=4	○=1<br>○=2<br>○=3<br>○=4	○=1<br>○=2<br>○=3<br>○=4	○
**2** Fever <3days (continuous)	○=1<br>○=2<br>○=3<br>○=4		○=1<br>○=2<br>○=3<br>○=4		○=1<br>○=2<br>○=3<br>○=4	○=1<br>○=2<br>○=3<br>○=4	○=1<br>○=2<br>○=3<br>○=4	○=1<br>○=2<br>○=3<br>○=4	○=1<br>○=2<br>○=3<br>○=4	○
**3** Fever ≥3days (continuous)	○=1<br>○=2<br>○=3<br>○=4		○=1<br>○=2<br>○=3<br>○=4		○=1<br>○=2<br>○=3<br>○=4	○=1<br>○=2<br>○=3<br>○=4	○=1<br>○=2<br>○=3<br>○=4	○=1<br>○=2<br>○=3<br>○=4	○=1<br>○=2<br>○=3<br>○=4	○
**4** Chills Shivering	○=1<br>○=2<br>○=3<br>○=4		○=1<br>○=2<br>○=3<br>○=4		○=1<br>○=2<br>○=3<br>○=4	○=1<br>○=2<br>○=3<br>○=4	○=1<br>○=2<br>○=3<br>○=4	○=1<br>○=2<br>○=3<br>○=4	○=1<br>○=2<br>○=3<br>○=4	○
**5** Convulsion	○=1<br>○=2<br>○=3<br>○=4		○=1<br>○=2<br>○=3<br>○=4		○=1<br>○=2<br>○=3<br>○=4	○=1<br>○=2<br>○=3<br>○=4	○=1<br>○=2<br>○=3<br>○=4	○=1<br>○=2<br>○=3<br>○=4	○=1<br>○=2<br>○=3<br>○=4	○
**6** Weight loss	○=1<br>○=2<br>○=3<br>○=4		○=1<br>○=2<br>○=3<br>○=4		○=1<br>○=2<br>○=3<br>○=4	○=1<br>○=2<br>○=3<br>○=4	○=1<br>○=2<br>○=3<br>○=4	○=1<br>○=2<br>○=3<br>○=4	○=1<br>○=2<br>○=3<br>○=4	○
**7** Dehydration	○=1<br>○=2<br>○=3<br>○=4		○=1<br>○=2<br>○=3<br>○=4		○=1<br>○=2<br>○=3<br>○=4	○=1<br>○=2<br>○=3<br>○=4	○=1<br>○=2<br>○=3<br>○=4	○=1<br>○=2<br>○=3<br>○=4	○=1<br>○=2<br>○=3<br>○=4	○
**8** Malaise Weakness/ Fatigue	○=1<br>○=2<br>○=3<br>○=4		○=1<br>○=2<br>○=3<br>○=4		○=1<br>○=2<br>○=3<br>○=4	○=1<br>○=2<br>○=3<br>○=4	○=1<br>○=2<br>○=3<br>○=4	○=1<br>○=2<br>○=3<br>○=4	○=1<br>○=2<br>○=3<br>○=4	○
**9** Aches/ Pain (muscle/joint/bone)	○=1<br>○=2<br>○=3<br>○=4		○=1<br>○=2<br>○=3<br>○=4		○=1<br>○=2<br>○=3<br>○=4	○=1<br>○=2<br>○=3<br>○=4	○=1<br>○=2<br>○=3<br>○=4	○=1<br>○=2<br>○=3<br>○=4	○=1<br>○=2<br>○=3<br>○=4	○
**10** Headache	○=1<br>○=2<br>○=3<br>○=4		○=1<br>○=2<br>○=3<br>○=4		○=1<br>○=2<br>○=3<br>○=4	○=1<br>○=2<br>○=3<br>○=4	○=1<br>○=2<br>○=3<br>○=4	○=1<br>○=2<br>○=3<br>○=4	○=1<br>○=2<br>○=3<br>○=4	○
**11** Dizziness/ Confusion Unconsciousness	○=1<br>○=2<br>○=3<br>○=4		○=1<br>○=2<br>○=3<br>○=4		○=1<br>○=2<br>○=3<br>○=4	○=1<br>○=2<br>○=3<br>○=4	○=1<br>○=2<br>○=3<br>○=4	○=1<br>○=2<br>○=3<br>○=4	○=1<br>○=2<br>○=3<br>○=4	○
**12** Blood pressure disorders	○=1<br>○=2<br>○=3<br>○=4		○=1<br>○=2<br>○=3<br>○=4		○=1<br>○=2<br>○=3<br>○=4	○=1<br>○=2<br>○=3<br>○=4	○=1<br>○=2<br>○=3<br>○=4	○=1<br>○=2<br>○=3<br>○=4	○=1<br>○=2<br>○=3<br>○=4	○

Site ___ ___ ___ Subarea ___ ___ ___ House number ___ ___ ___ ___ Interviewer ID ___ ___

Sign/Symptom	Healthcare options									
	Study-HCF		Other HCF		Physician	Pharmacy	Traditional healer	Nowhere Self-treatment	Nowhere Nothing	Don't know
	Rate	Code	Rate	Code	Rate	Rate	Rate	Rate	Rate	Code
**13** Heart disorders	O=1<br>O=2<br>O=3<br>O=4		O=1<br>O=2<br>O=3<br>O=4		O=1<br>O=2<br>O=3<br>O=4	O=1<br>O=2<br>O=3<br>O=4	O=1<br>O=2<br>O=3<br>O=4	O=1<br>O=2<br>O=3<br>O=4	O=1<br>O=2<br>O=3<br>O=4	O
**14** Nausea Vomitting	O=1<br>O=2<br>O=3<br>O=4		O=1<br>O=2<br>O=3<br>O=4		O=1<br>O=2<br>O=3<br>O=4	O=1<br>O=2<br>O=3<br>O=4	O=1<br>O=2<br>O=3<br>O=4	O=1<br>O=2<br>O=3<br>O=4	O=1<br>O=2<br>O=3<br>O=4	O
**15** Diarrhea (watery/ bloody/ mucopurulent)	O=1<br>O=2<br>O=3<br>O=4		O=1<br>O=2<br>O=3<br>O=4		O=1<br>O=2<br>O=3<br>O=4	O=1<br>O=2<br>O=3<br>O=4	O=1<br>O=2<br>O=3<br>O=4	O=1<br>O=2<br>O=3<br>O=4	O=1<br>O=2<br>O=3<br>O=4	O
**16** Abdominal pain Intestinal pain	O=1<br>O=2<br>O=3<br>O=4		O=1<br>O=2<br>O=3<br>O=4		O=1<br>O=2<br>O=3<br>O=4	O=1<br>O=2<br>O=3<br>O=4	O=1<br>O=2<br>O=3<br>O=4	O=1<br>O=2<br>O=3<br>O=4	O=1<br>O=2<br>O=3<br>O=4	O
**17** Blood loss/ Bleeding (internal)	O=1<br>O=2<br>O=3<br>O=4		O=1<br>O=2<br>O=3<br>O=4		O=1<br>O=2<br>O=3<br>O=4	O=1<br>O=2<br>O=3<br>O=4	O=1<br>O=2<br>O=3<br>O=4	O=1<br>O=2<br>O=3<br>O=4	O=1<br>O=2<br>O=3<br>O=4	O
**18** Blood loss/ Bleeding (external)	O=1<br>O=2<br>O=3<br>O=4		O=1<br>O=2<br>O=3<br>O=4		O=1<br>O=2<br>O=3<br>O=4	O=1<br>O=2<br>O=3<br>O=4	O=1<br>O=2<br>O=3<br>O=4	O=1<br>O=2<br>O=3<br>O=4	O=1<br>O=2<br>O=3<br>O=4	O
**19** Sneezing Runny nose	O=1<br>O=2<br>O=3<br>O=4		O=1<br>O=2<br>O=3<br>O=4		O=1<br>O=2<br>O=3<br>O=4	O=1<br>O=2<br>O=3<br>O=4	O=1<br>O=2<br>O=3<br>O=4	O=1<br>O=2<br>O=3<br>O=4	O=1<br>O=2<br>O=3<br>O=4	O
**20** Cough	O=1<br>O=2<br>O=3<br>O=4		O=1<br>O=2<br>O=3<br>O=4		O=1<br>O=2<br>O=3<br>O=4	O=1<br>O=2<br>O=3<br>O=4	O=1<br>O=2<br>O=3<br>O=4	O=1<br>O=2<br>O=3<br>O=4	O=1<br>O=2<br>O=3<br>O=4	O
**21** Rapid breathing/ shortness of breath	O=1<br>O=2<br>O=3<br>O=4		O=1<br>O=2<br>O=3<br>O=4		O=1<br>O=2<br>O=3<br>O=4	O=1<br>O=2<br>O=3<br>O=4	O=1<br>O=2<br>O=3<br>O=4	O=1<br>O=2<br>O=3<br>O=4	O=1<br>O=2<br>O=3<br>O=4	O
**22** Severe breathing/ Difficulties in breathing	O=1<br>O=2<br>O=3<br>O=4		O=1<br>O=2<br>O=3<br>O=4		O=1<br>O=2<br>O=3<br>O=4	O=1<br>O=2<br>O=3<br>O=4	O=1<br>O=2<br>O=3<br>O=4	O=1<br>O=2<br>O=3<br>O=4	O=1<br>O=2<br>O=3<br>O=4	O
**23** Skin inflammation (rashes/ redness/ pus/ itching)	O=1<br>O=2<br>O=3<br>O=4		O=1<br>O=2<br>O=3<br>O=4		O=1<br>O=2<br>O=3<br>O=4	O=1<br>O=2<br>O=3<br>O=4	O=1<br>O=2<br>O=3<br>O=4	O=1<br>O=2<br>O=3<br>O=4	O=1<br>O=2<br>O=3<br>O=4	O
**24** Swelling/ Edema	O=1<br>O=2<br>O=3<br>O=4		O=1<br>O=2<br>O=3<br>O=4		O=1<br>O=2<br>O=3<br>O=4	O=1<br>O=2<br>O=3<br>O=4	O=1<br>O=2<br>O=3<br>O=4	O=1<br>O=2<br>O=3<br>O=4	O=1<br>O=2<br>O=3<br>O=4	O
**25** Jaundice	O=1<br>O=2<br>O=3<br>O=4		O=1<br>O=2<br>O=3<br>O=4		O=1<br>O=2<br>O=3<br>O=4	O=1<br>O=2<br>O=3<br>O=4	O=1<br>O=2<br>O=3<br>O=4	O=1<br>O=2<br>O=3<br>O=4	O=1<br>O=2<br>O=3<br>O=4	O

FORM 4: ASSESSMENT OF OVERALL, AGE- AND SEX-STRATIFED GENERIC HEALTHCARE UTILIZATION

Part B – Healthcare-associated factors/Vaccination/Disease perception

TRAVEL HEALTHCARE FACILITY

1 In case a healthcare facility (HCF) was rated the 1st choice of care in question 4A (fever), what is the main mode of transportation, travel time (depending on mode of transportation), travel cost (depending on mode of transportation) and travel distance (depending on mode of transportation) that is usually taken to get there?

Instructions: Enter one code per category and sex as well as age group only. Skip this question if there is no female/male member <2years/≥2 to <5years/≥5 to <15years/≥15years of age in this household (see also question 2 and 3 of this form PartA). Skip this question if no HCF was rated the 1st choice of care in question 4A for female/male member <2years/≥2 to <5years/≥5 to <15years/≥15years of age. Record the travel time as minute(s) [min]/ hour(s) [hr], the total costs in USD, including potential gifts, favours and tips, and the total distance as meter(s) [m]/kilometre(s) [km].

Female				Male			
<2 years	≥2 to <5 years	≥5 to <15 years	≥15 years	<2 years	≥2 to <5 years	≥5 to <15 years	≥15 years
**1.1 Transportation mode**							
Code ___	Code ____	Code ____	Code ___	Code ___	Code ____	Code ____	Code ___
[1] Private transportation (i.e. motorbike, car, bus)<br>[2] Public transportation (i.e. motorbike, car, bus)<br>[3] Walking<br>[4] Bicycle<br>[5] Other, specify ________<br>[99] Don't know<br>[98] No response							
**1.2 Travel time**							
Code ___	Code ____	Code ____	Code ___	Code ___	Code ____	Code ____	Code ___
[1] <30 mins<br>[2] ≥30 to <60 mins<br>[3] ≥1.0 to <2.0 hrs<br>[4] ≥2.0 to <3.0 hrs<br>[5] ≥3.0 to <4.0 hrs<br>[6] ≥4.0 hrs<br>[99] Don't know<br>[98] No response							
**1.3 Travel cost**							
Code ___	Code ____	Code ____	Code ___	Code ___	Code ____	Code ____	Code ___
[1] <1.0 USD<br>[2] ≥1.0 to <3.0 USD<br>[3] ≥3.0 to <5.0 USD<br>[4] ≥5.0 to <10.0 USD<br>[5] ≥10.0 USD<br>[99] Don't know<br>[98] No response							
**1.4 Travel distance**							
Code ___	Code ____	Code ____	Code ___	Code ___	Code ____	Code ____	Code ___
[1] <500 m<br>[2] ≥500 m to <1.0 km<br>[3] ≥1.0 to <2.0 km<br>[4] ≥2.0 to <3.0 km<br>[5] ≥3.0 to <4.0 km<br>[6] ≥4.0 km<br>[99] Don't know<br>[98] No response							

INSURANCE

2 For which members of this household does the insurance cover health expenses?

Instructions: Enter one code per category. Skip this question if there is no member <2years/≥2 to <5years/≥5 to <15years/≥15years of age in this household (see question 2 and 3 of this form PartA).

<2 years	≥2 to <5 years	≥5 to <15 years	≥15 years
Code ____________	Code ____________	Code ____________	Code ____________
[1] Yes<br>[2] No<br>[99] Don't know<br>[98] No response			

3 What kind of health expenses are usually covered by the insurance for members of this household?

Instructions: Enter one code by category and age group only. Skip this question if there is no member <2years/≥2 to <5years/≥5 to <15years/≥15years of age in this household (see also question 2 and 3 of this form PartA). Skip this question if there is no insurance that covers health expenses for <2years/≥2 to <5years/≥5 to <15years/≥15years of age.

	<2 years	≥2 to <5years	≥5 to <15years	≥15years
	[1] Yes	[2] No	[99] Don't know	[98] No response
**1** Registration	Code ______	Code _______	Code _______	Code _____
**2** Consultation/ Doctor's visit	Code ______	Code _______	Code _______	Code _____
**3** Physical examination - general	Code ______	Code _______	Code _______	Code _____
**4** Physical examination – specific, specify_______	Code ______	Code _______	Code _______	Code _____
**5** Diagnostics - general laboratory diagnostics	Code ______	Code _______	Code _______	Code _____
**6** Diagnostics - specific laboratory diagnostics, specify__________	Code ______	Code _______	Code _______	Code _____
**7** Diagnostics - general imaging	Code ______	Code _______	Code _______	Code _____
**8** Diagnostics - specific imaging, specify__________	Code ______	Code _______	Code _______	Code _____
**9** Diagnostics - other, specify________	Code ______	Code _______	Code _______	Code _____
**10** Treatment - general medication	Code ______	Code _______	Code _______	Code _____
**11** Treatment - specific medication, specify________	Code ______	Code _______	Code _______	Code _____
**12** Treatment - other medication, specify__________	Code ______	Code _______	Code _______	Code _____
**13** Treatment - interventions (other than surgery), specify_______	Code ______	Code _______	Code _______	Code _____
**14** Treatment Surgery	Code ______	Code _______	Code _______	Code _____
**15** Hospitalization <7days	Code ______	Code _______	Code _______	Code _____
**16** Hospitalization ≥7days	Code ______	Code _______	Code _______	Code _____
**17** Emergency services	Code ______	Code _______	Code _______	Code _____
**18** Antenatal care	Code ______	Code _______	Code _______	Code _____
**19** Postnatal care	Code ______	Code _______	Code _______	Code _____
**20** Preventive medicine	Code ______	Code _______	Code _______	Code _____
**21** Vaccination	Code ______	Code _______	Code _______	Code _____
**22** Home healthcare (i.e. elderly, disabled, chronic/long-term diseased)	Code ______	Code _______	Code _______	Code _____
**23** Supportive therapy (i.e. physiotherapy, ergotherapy, rehabilitation)	Code ______	Code _______	Code _______	Code _____

ETHNOGRAPHY

4 To which religion/ethnic group/tribe belong members of this household?

Instructions: Give one answer by age group only. Skip this question if there is no member >2years/≥2 to <5years/≥5 to <15years/≥15years of age in this household (see also question 2 and 3 of this form PartA).

	<2 years	≥2 to <5years	≥5 to <15years	≥15years
Religion/Ethnic group/Tribe	______________	______________	______________	______________
	O 99=Don't know<br>O 98=No response	O 99=Don't know<br>O 98=No response	O 99=Don't know<br>O 98=No response	O 99=Don't know<br>O 98=No response

5 Does the religion/ethnic group/tribe as stated in question 4 influence the various aspects of utilization of healthcare for members of this household?

Instructions: Enter one code by category and age group only. Skip this question if there is member >2years/≥2 to <5years/≥5 to <15years/≥15years of age in this household (see also question 2 and 3 of this form PartA).

	<2 years	≥2 to <5years	≥5 to <15years	≥15years
	[1] Yes	[2] No	[99] Don't know	[98] No response
**1** Visiting a healthcare facility (i.e. hospital, health care center, health post)	Code _____	Code _______	Code _______	Code _____
**2** Visiting a physician	Code _____	Code _______	Code _______	Code _____
**3** Visiting a pharmacy	Code _____	Code _______	Code _______	Code _____
**4** Visiting a traditional healer	Code _____	Code _______	Code _______	Code _____
**5** Deciding for self-treatment	Code _____	Code _______	Code _______	Code _____
**6** Deciding for non-treatment	Code _____	Code _______	Code _______	Code _____
**7** Physical examination – general	Code _____	Code _______	Code _______	Code _____
**8** Physical examination – specific, specify_______	Code _____	Code _______	Code _______	Code _____
**9** Collection of specimen (i.e. blood, urine, stool, smear, swab, cerebrospinal fluid)	Code _____	Code _______	Code _______	Code _____
**10** Diagnostics – general laboratory diagnostics	Code _____	Code _______	Code _______	Code _____
**11** Diagnostics – specific laboratory diagnostics, specify__________	Code _____	Code _______	Code _______	Code _____
**12** Diagnostics - general imaging	Code _____	Code _______	Code _______	Code _____
**13** Diagnostics - specific imaging, specify________	Code _____	Code _______	Code _______	Code _____
**14** Diagnostics - other, specify________	Code _____	Code _______	Code _______	Code _____
**15** Treatment - general medication	Code _____	Code _______	Code _______	Code _____
**16** Treatment - specific medication, specify_______	Code _____	Code _______	Code _______	Code _____
**17** Treatment - other medication, specify__________	Code _____	Code _______	Code _______	Code _____

**18** Treatment - interventions (other than surgery), specify________________	Code _____	Code _______	Code _______	Code _____
**19** Treatment - Surgery	Code _____	Code _______	Code _______	Code _____
**20** Hospitalization <7days	Code _____	Code _______	Code _______	Code _____
**21** Hospitalization ≥7days	Code _____	Code _______	Code _______	Code _____
**22** Emergency services	Code _____	Code _______	Code _______	Code _____
**23** Antenatal care	Code _____	Code _______	Code _______	Code _____
**24** Postnatal care	Code _____	Code _______	Code _______	Code _____
**25** Preventive medicine	Code _____	Code _______	Code _______	Code _____
**26** Vaccination	Code _____	Code _______	Code _______	Code _____
**27** Home healthcare (i.e. elderly, disabled, chronic/long-term diseased)	Code _____	Code _______	Code _______	Code _____
**28** Supportive therapy (i.e. physiotherapy, ergotherapy, rehabilitation)	Code _____	Code _______	Code _______	Code _____

VACCINATION

6 How were children of this household fed during the first six months of life?

Instructions: Skip this question if there are no male/female children in this household (see also question 2 and 3 of this form PartA).

- O 1=Breastfed exclusively
- O 2=Breastfed and other feeds, specify ______
- O 3=Formula and other feeds, specify ________
- O 4=Breastfed and formula
- O 5=Formula exclusively
- O 6=Other, specify ________________
- O 99=Don't know
- O 98=No response

7 Which water source was usually used to prepare formula or formula and other feeds as stated in question 6 for children of this household?

Instructions: Skip this question if there are no children in this household (see also question 2 and 3 of this form PartA).

- O 1=Tap water (inside the house; private)
- O 2=Tap water (outside the house i.e. back-/courtyard; private)
- O 3=Tap water (outside the house; public, shared)
- O 4=Standpipe/Piped water (inside the house; private)
- O 5=Standpipe/Piped water (outside the house i.e. back-/courtyard; private)
- O 6=Standpipe/Piped water (outside the house; public, shared)
- O 7=Water truck (public; shared)
- O 8=Bottled water
- O 9=Surface water (i.e. lake, pond, river, stream)
- O 10=Surface water (i.e. canal, irrigation canal)
- O 11=Spring water
- O 12=Rain water
- O 13=Well/Borehole (open/uncovered/unprotected)
- O 14=Well/Borehole (covered/protected)
- O 15=NO FORMULA/OTHER FEEDS PREPARED
- O 16=Other, specify _____________
- O 99=Don't know
- O 98=No response

8 Did children of this household receive any vaccines since birth, including the birth vaccines?

Instructions: Enter one code by category and child only. Skip this question if there are no children in this household (see also question 2 and 3 of this form PartA). Refer to question 9 of "Form 3 Part A – Demographic data/General household information" and record the initials/identifier of the member(s).

Child 1<br>Initials/Identifier: _____	Child 2<br>Initials/Identifier: _____	Child 3<br>Initials/Identifier: _____	Child 4<br>Initials/Identifier: _____

[1] YES, vaccinated<br>[2] No, caretaker busy<br>[3] No, caretaker decided no need<br>[4] No, unknown where to go	[5] No, lack of vaccine<br>[6] No, child too old/young<br>[7] No, caretaker refused/too worried<br>[8] No, other, specify ___________	[9] No, HCF busy<br>[10] No, child sick<br>[11] No, no vaccination card<br>[99] Don't know	[98] No response
**8.1 Diphtheria and Tetanus and Pertussis and *Haemophilus influenza* and Hepatitis B vaccine/DTwPHibHepB** (e.g. 6/8, 10/12, 14/16 weeks)			
Code __________	Code __________	Code __________	Code __________
**8.2 Tetanus toxoid (TT)** (e.g. 1st contact pregnancy, 1, 6, 12/24 months)			
Code __________	Code __________	Code __________	Code __________
**8.3 Tetanus and diphtheria toxoid (TD)** (e.g. 1st contact pregnancy, 1, 6, 12 months)			
Code __________	Code __________	Code __________	Code __________
**8.4 Tuberculosis/BCG** (at birth)			
Code __________	Code __________	Code __________	Code __________
**8.5 Rotavirus/RV** (e.g. 6/8, 10/12, 16 weeks)			
Code __________	Code __________	Code __________	Code __________
**8.6 Measles/Measles and Rubella (MR)** (e.g. 9, 15/18months)			
Code __________	Code __________	Code __________	Code __________
**8.7 Polio/OPV** (e.g. birth, 6/8, 10/12, 14/16 weeks)			
Code __________	Code __________	Code __________	Code __________
**8.8 Pneumococcus/PCV** (e.g. 6/8, 10/12, 14/16 weeks)			
Code __________	Code __________	Code __________	Code __________
**8.9 Yellow fever (YF)** (e.g. 9 months)			
Code __________	Code __________	Code __________	Code __________
**8.10 Other Vaccines**			
Specify, __________	Specify, __________	Specify, __________	Specify, __________
Specify, __________	Specify, __________	Specify, __________	Specify, __________
Specify, __________	Specify, __________	Specify, __________	Specify, __________
Specify, __________	Specify, __________	Specify, __________	Specify, __________

Site ___ ___ ___ Subarea ___ ___ ___ House number ___ ___ ___ ___ Interviewer ID ___ ___

DISEASE PERCEPTION

9 Knowledge, perception, causes and prevention of common infectious diseases

**Disease**	**9.1 Disease known by household members?**<br>[1] Yes<br>[2] No<br>[99] Don't know<br>[98] No response	**9.2 Perception of disease by household members?**<br>*Instruction:* Describe briefly if known.	**9.3 Causes for disease known by household members?**<br>*Instructions:* Enter all codes (list A) that apply; more than one answer is possible.	**9.4 Measures to prevent disease known by household members?**<br>*Instructions:* Enter all codes (list B) that apply; more than one answer is possible.	**9.5 Measures to prevent disease utilized by household members?**<br>*Instructions:* Enter all codes (list B) that apply; more than one answer is possible.
**A.** Typhoid fever	Code ___________		Code ___________	Code ___________	Code ___________
**B.** Cholera	Code ___________		Code ___________	Code ___________	Code ___________
**C.** Malaria	Code ___________		Code ___________	Code ___________	Code ___________
**D.** Influenza	Code ___________		Code ___________	Code ___________	Code ___________
**E.** Meningitis	Code ___________		Code ___________	Code ___________	Code ___________
**F.** Hepatitis E	Code ___________		Code ___________	Code ___________	Code ___________
**G.** Tuberculosis	Code ___________		Code ___________	Code ___________	Code ___________
**H.** HIV/AIDS	Code ___________		Code ___________	Code ___________	Code ___________

List A: Possible causes	List B: Possible preventive measures
[1] Alcohol consumption<br>[2] Weather/witchcraft<br>[3] Mosquito/insect bite<br>[4] Dog bite	[1] No alcohol consumption<br>[2] Sleeping under bed net<br>[3] Using mosquito/insect spray<br>[4] Maintain a clean house and immediate surroundings of the house

[5] Drinking untreated surface/spring water (i.e. lake, pond, river, stream, canal/irrigation canal)<br>[6] Close body contact/kissing/hugging<br>[7] Eating raw food items such as vegetables/lettuce, fruits, dairy products, eggs, meat or fish<br>[8] Not cleaning hands properly when preparing food/meals<br>[9] Not cleaning hands properly before/after visiting a toilet<br>[10] Contact to human blood (i.e. injection, transfusion, surgery)<br>[11] Consuming raw animal blood<br>[12] Other, specify _______________<br>[99] Don't know<br>[98] No response	[5] Practice proper disposal of human and domestic waste<br>[6] Avoiding close contact to dogs<br>[7] Treating water (i.e. boiling, filtration, solar disinfection, sedimentation, chlorination)<br>[8] Not cooking/preparing food/meals with untreated water<br>[9] Not washing food items to be consumed raw with untreated water<br>[10] Re-boiling/re-heating left-over/prepared food/meals prior to consumption<br>[11] Not eating raw food items such as vegetables/lettuce, fruits, dairy products, eggs, meat or fish<br>[12] Cover food items/meals<br>[13] Protect food items/meals from flies<br>[14] Avoid food/beverage from street vendors<br>[15] Avoid close contact to sick people<br>[16] No close body contact/kissing/hugging<br>[17] Covering nose/mouth while sneezing/coughing<br>[18] No contact/consumption of human/animal blood<br>[19] Practicing proper hand hygiene when preparing food/meals (i.e. washing hands with soap and water, rub hands clean)<br>[20] Practicing proper hand hygiene before/after visiting a toilet (i.e. washing hands with soap and water, rub hands clean)<br>[21] Other, specify ____________________<br>[99] Don't know<br>[98] No response

10 For which members of this household are the preventive measures as listed in question 9.5 against typhoid fever used?

Instructions: Tick all that applies; more than one answer is possible.

O 1=<2years O 3=≥5 to <15years O 5=NOT USED O 99=Don't know
O 2=≥2 to < 5years O 4=≥15years O 98=No response

11 Name the household members that were diagnosed with typhoid fever during the past 3 months. Which diagnostics were used to confirm typhoid fever?

Instructions: Enter all codes that apply for each household member; more than one answer is possible.

Member 1<br>Initials/Identifier: _____	Member 2<br>Initials/Identifier: _____	Member 3<br>Initials/Identifier: _____	Member 4<br>Initials/Identifier: _____
[1] Blood culture<br>[2] Stool culture	[3] Blood/Widal test<br>[4] Other test, specify_____________	[5] Not applicable (not diagnosed with typhoid fever)<br>[99] Don't know	[98] No response
Code __________	Code __________	Code __________	Code __________

12 (Where were members of this household recorded in question 11 diagnosed for typhoid fever?) Where did members of this household seek care?

Instructions: Enter one code by household member only.

Member 1<br>Initials/Identifier: _____	Member 2<br>Initials/Identifier: _____	Member 3<br>Initials/Identifier: _____	Member 4<br>Initials/Identifier: _____
[1] Healthcare facility, specify ____________<br>[2] Physician<br>[3] Nowhere, self-treatment<br>[4] Traditional healer<br>[5] Pharmacy		[6] Nowhere, did not seek care<br>[7] Other, specify ____________<br>[8] Not applicable<br>[99] Don't know<br>[98] No response	
Code __________	Code __________	Code __________	Code __________

13 (Did members of this household that were diagnosed with typhoid fever as recorded in question 11 get any treatment?) Which treatment did they get?

Instructions: Tick one answer by household member only.

Member 1<br>Initials/Identifier: _____	Member 2<br>Initials/Identifier: _____	Member 3<br>Initials/Identifier: _____	Member 4<br>Initials/Identifier: _____
O 1=Treatment, specify<br>_______________<br>O 2=Not applicable<br>O 99=Don't know<br>O 98=No response	O 1=Treatment, specify<br>_______________<br>O 2=Not applicable<br>O 99=Don't know<br>O 98=No response	O 1=Treatment, specify<br>_______________<br>O 2=Not applicable<br>O 99=Don't know<br>O 98=No response	O 1=Treatment, specify<br>_______________<br>O 2=Not applicable<br>O 99=Don't know<br>O 98=No response

Site ___ ___ ___ Subarea ___ ___ ___ House number ___ ___ ___ ___ Interviewer ID ___ ___

FORM 5: ASSESSMENT OF OVERALL, AGE-AND SEX-STRATIFIED ACTUAL HEALTHCARE UTILIZATION

HEALTHCARE SEEKING BEHAVIOR

1 Record below any occurrence of a sign(s)/ a symptom(s) (see List 1) and specify where (see List 2) and how often a member/members sought healthcare during the past 3 months?

Instructions: See "Form 3 - Part A - GENERAL HOUSEHOLD INFORMATION" to obtain the initials/identifier of each household member. Indicate one sign/symptom per line only. Start a new line if different healthcare options were selected for recurring signs/symptoms. Indicate the number of occurrences for each sign/symptom during the past 3 months. If a different healthcare option was taken for a recurring sign/symptom, indicate this on a new line. Please complete an additional form if >20 occurrences took place during the past 3 months.

**List 1: Sign/Symptom**		**List 2: Healthcare option**
[A] Fever, any<br>[B] Fever <3days (continuous)<br>[C] Fever ≥3days (continuous)<br>[D] Chills/ Shivering<br>[E] Convulsion<br>[F] Weight loss<br>[G] Dehydration<br>[H] Malaise/ Weakness/ Fatigue<br>[I] Aches/Pain (muscle/joint/bone)<br>[J] Headache<br>[K] Dizziness/Confusion/ Unconsciousness<br>[L] Blood pressure disorders<br>[M] Heart disorders	[N] Nausea/ Vomiting<br>[O] Diarrhea (watery/bloody/mucopurulent)<br>[P] Abdominal pain/ Intestinal pain<br>[Q] Blood loss/ Bleeding (internal)<br>[R] Blood loss/ Bleeding (external)<br>[S] Sneezing/ Runny nose<br>[T] Cough<br>[U] Rapid breathing/ Shortness of breath<br>[V] Severe breathing/ Difficulties in breathing<br>[W] Skin inflammation (rashes/redness/ pus/itching)<br>[X] Swelling/ Edema<br>[Y] Jaundice	[1] Study-HCF, Code __________<br>[2] Other HCF, Code __________<br>[3] Physician<br>[4] Pharmacy<br>[5] Traditional healer<br>[6] Nowhere/Self-treatment<br>[7] Nowhere/Nothing

**No.**	**Household member** Record the initials/identifier of a household member; see "Form 3 - Part A - GENERAL HOUSEHOLD INFORMATION"	**Sign/ Symptom** Use List 1 to select a code	**Healthcare option**		**Frequency** Indicate the number of incidences for the sign/symptom listed
			Use List 2 to select a code	HCF code	
1					
2					
3					
4					
5					
6					
7					
8					
9					

10					
11					
12					
13					
14					
15					
16					
17					
18					
19					
20					
