# Supplementary material for: The HPAfrica protocol: Assessment of health behaviour and population-based socioeconomic, hygiene behavioural factors - a standardised repeated cross-sectional study in multiple cohorts in sub-Saharan Africa
Source: BMJ Open. 2018 Dec 19;8(12):e021438. doi: 10.1136/bmjopen-2017-021438 (PMC6303690; doi:10.1136/bmjopen-2017-021438)
Supplement: Supplementary file 3 [file bmjopen-2017-021438supp003.pdf]

**Appendix 3/Supplementary file 3: Protocol, Definitions/Terminology and Study Forms (French)**

# Protocol

# Le protocole d'étude HPAfrica: Évaluation du comportement de santé et des facteurs démographiques socio-économiques, comportement d'hygiène - une étude standardisée répétée transversale dans plusieurs cohortes en Afrique subsaharienne


## Abstrait

**Introduction** L'objectif de l'étude HPAfrica est de déterminer les comportements sanitaires et facteurs démographiques, y compris les facteurs socio-économiques, ethnographiques aussi bien que d'hygiène et d'assainissement, sur les sites d'étude du programme Fièvre Typhoïde Sévère en Afrique (SETA). L'objectif du programme SETA est d'enquêter sur la surveillance de la fièvre au niveau des établissements de soins de santé au Burkina Faso, en République Démocratique du Congo, en Éthiopie, au Ghana, à Madagascar et au Nigéria. Les estimations précises de la charge de morbidité nécessitent un ajustement des styles de comportements de santé, qui sont supposés varier au sein d'une population d'étude.

**Methodes et Analyses** Pour la taille d'échantillon minimale d'interviews de ménages requise, les hypothèses d'une population infinie, d'un effet de design et d'une stratification par âge et par sexe sont considérées. En l'absence d'un cadre d'échantillonnage de la population ou d'une liste de ménages, une approche spatiale sera utilisée pour générer des points aléatoires géographiques avec un outil ArcGIS (Aeronautical Reconnaissance Coverage Geographic Information System). Les impressions de l'imagerie satellite de Google Earth Pro® visualisent ces points. Les données d'intérêt seront évaluées au niveau des ménages en différentes saisons en appliquant un échantillonnage stratifié en fonction de la population. Une application basée sur Android et un service Web sera développé pour la capture de données électroniques et la synchronisation avec le serveur de base de données sur Internet en temps réel. Les poids d'échantillonnage seront calculés pour tenir compte des differences possibles dans les probabilitiés de selection. Des analyses de données descriptives seront effectuées afin d'évaluer l'informations de base de chaque population étudée et le comportement santé stratifié par âge et par sexe. Ceci permettra d'ajuster les estimations de la charge de morbidité. En outre, des analyses multivariées seront appliquées pour examiner les associations entre le comportement santé, les facteurs demographiques et le fardeau de la maladie trouvée dans l'étude SETA.

### Ethique et diffusion

Les approbations éthique pour ce protocole ont été reçues par l'International Vaccine Institute (IVI, n ° 2016-0003) et par toutes les institutions collaboratrices des pays participants. Il est prévu de diffuser les résultats de cette étude par publication dans un journal révise par des pairs.

### Les forces et les limites de cette étude

- La normalisation dans la recherche multi-pays /-site à base communautaire nécessite de la prudence compte tenu des variations de cadre, de langue et de culture.
- Les évaluations de santé publique à grande échelle complèteront les données sur la charge de morbidité en étudiant les sources de transmission et d'infection.
- Les données de population actuelles dans les sites d'étude et leurs sous-zones définies administrativement et les bases de sondage de population sont supposées être limitées.
- La collecte de données électroniques peut entraîner problèmes techniques liés aux dommages, aux pannes fonctionnelles ou à la perte de dispositifs.
- Une connexion Internet sans fil instable peut limiter les équipes de données à mettre à jour l'application basée sur Android, communiquer en temps réel avec les responsables de l'étude et à synchroniser les données collectées avec le serveur.

**Inscription:** non requise

**Mots clés:** comportement sanité/hygiénique, assainissement, socio-économique, cadre d'échantillonnage de la population/l'échantillonnage spatiale, Afrique subsaharienne, HPAfrica étude

**Contexte**
L'évaluation du comportement de santé, y compris l'utilisation des soins de santé, est un déterminant important pour la production d'estimations précises du fardeau de la maladie parmi les populations cibles. Ceci est particulièrement important pour les études qui utilisent un modèle de surveillance observationnelle basé sur les établissements de santé, car une variation substantielle des comportements de santé influence fortement les estimations de la maladie (1). L'ajustement des calculs de la maladie pour des variations du comportement de santé augmentera l'exactitude de l'évaluation des mesures de la fréquence des maladies causées par des pathogènes bactériens, fongiques, viraux ou parasitaires parmi les populations étudiées. De plus, l'influence d'une grande variété de facteurs basés sur la population sur le comportement de santé et le fardeau de la maladie a été reconnue. Cela inclut des facteurs socioéconomiques tels que l'éducation, la profession, le revenu, la disponibilité des biens des ménages (2, 3), les facteurs ethniques / religieux et culturels (4, 5), mais aussi l'hygiène et l'assainissement (6-10), la manipulation des aliments (11-14) et les contacts avec des animaux (9).

L'étude **H**ealth **P**opulation **Africa** (HPAfrica) sera menée au Burkina Faso, en République démocratique du Congo (RDC), en Éthopie, au Ghana, à Madagascar et au Nigéria. Les expériences du programme Typhoid Fever Surveillance in Africa Program (TSAP) seront reportées sur le programme Severe Typhoid in Africa (SETA) (15, 16). Dans ce programme, une surveillance standardisée de la fièvre, basée sur les établissements de santé, est effectuée sur des sites d'étude sélectionnés dans les pays participants sur une période de deux ans (17). SETA vise principalement à collecter systématiquement des informations sur le fardeau des infections graves à *Salmonella*. Les sujets vivant dans la zone the chalandise définie, avec un épisode de fièvre aiguë, des antécédents de fièvre ≥ 3 jours consécutifs, une fièvre typhoïde cliniquement soupçonnée ou des perforations gastro-intestinales cliniquement diagnostiquées dues à la fièvre typhoïde seront éligibles. En outre, la sévérité des maladies provoquées par *Salmonella* et l'histoire naturelle de l'infection à *Salmonella* seront enregistrées avec des analyses supplémentaires sur les réponses immunitaires de l'hôte et le portage chronique.

**Objectifs et Résultats**
L'objectif de l'étude HPAfrica est d'évaluer le comportement de santé générique et réel stratifié par âge et par sexe. Le comportement générique se rapporte au comportement associé à la fièvre et à d'autres signes et symptômes sans l'apparition, alors que le comportement réel est lié à l'apparition (Formulaire 4 Partie A, Formulaire 5). Les données sont utilisées pour ajuster les mesures de l'occurrence de la maladie comme l'incidence d'agents pathogènes infectieux stratifiés selon l'âge et le sexe pour la proportion d'une population étudiée non capturée dans le programme de surveillance SETA. Les données documentaires pour divers signes et symptômes ou une combinaison de ceux-ci sont utilisés pour une classification progressive de l'utilisation des soins de santé en fonction de la gravité de la maladie.

L'étude HPAfrica recueillera des données sur les facteurs associés aux soins de santé, tels que les modalités de voyage, la possession d'une assurance maladie, les facteurs culturels/ethnographiques, l'état vaccinal des enfants (18, 19) et la fréquence et la perception individuelle des maladies (Formulaire 4 Partie B). Les données démographiques (Formulaire 3 Partie A) seront utilisées pour la stratification par âge et par sexe de la population étudiée qui est nécessaire pour calculer les facteurs d'ajustement pour les mesures de l'occurrence de la maladie. Les données socio-économiques (Formulaire 3 Partie B) seront utilisées pour calculer les indices de richesse pour les approches descriptives et analytiques. Les informations sur l'hygiène et l'assainissement (Formulaire 3 Partie C) peuvent permettre une meilleure compréhension de l'occurrence et de la fréquence des agents pathogènes identifiés parmi les populations étudiées.

**Méthodes**
**Sites d'étude**

Les limites de chaque site seront définies en utilisant des indications préexistantes qui avaient été déterminées par les autorités statistiques ou ministères de la santé (Tableau 1), couplées à des données géospatiales à haute résolution de source ouverte. L'examen rétrospectif des registres de des deux dernières années aux établissements de soins de santé de recrutement SETA permettra de redéfinir les limites du site. On s'attend à ce que cette revue révèle les résidences ou au moins les zones résidentielles administratives plus larges telles que des communautés, des districts ou des villages de patients qui ont cherché des soins de santé pour une raison quelconque. Des cartes et des images satellites peuvent être utilisées pour mieux visualiser les résidences des patients et les limites générales, y compris les sous-zones définies géographiquement ou administrativement, les secteurs de recensement ou les strates de chaque site. Là où les limites ne peuvent être clairement définies, des facteurs supplémentaires, tels que la distance à un établissement de soins de santé de recrutement, peuvent être pris en compte.

**Taille d'échantillons**

Toutes les sources disponibles seront utilisées pour recueillir des données sur la population le plus à jour stratifiées selon l'âge et le sexe par site d'étude et par les sous-zones définies administrativement ou géographiquement ou les strates. Les strates seront définies comme la plus petite unité administrative publiée par le recensement d'un pays participant. Les ménages dans les strates devraient être répartis de manière homogène. Les sources de données sur la population pour les dénombrements par strate cCela peut inclure les dernières informations démographiques issues d'un recensement d'un total de la population ou d'un DSS (Demographic Surveillance System) / HDSS (Health and Demographic Surveillance System). Les chiffres sommaires sur la population et les facteurs de croissance de la population, s'ils sont disponibles, peuvent constituer une source de données supplémentaire. Pour les sites avec des chiffres de population obsolètes ou indisponibles, des outils d'échantillonnage en accès libre couplés à des sources de données sur la population, comme les données sur la population quadrillée basées sur la densité de WorldPop peuvent également être utilisés (20-25).

Toutes les strates par site d'étude seront incluses. Un ménage tel que défini pour l'étude HPAfrica constitue l'unité d'échantillonnage primaire (PSU). Le nombre minimum de ménages sélectionnés au hasard sera calculé en utilisant l'équation basée de précision (équation-I) en supposant une population infinie et en considérant un effet de design (DEFF) (26-28), y compris les hypothèses qui l'accompagnent:

**Equation-I: Calcul de la taille d'échantillon basé sur la précision par site d'étude pour une population infinie (26-32)**

$$n_0 = DEFF \cdot \left[ {z^2}_{1-\alpha/2} \cdot \frac{4 \cdot p \cdot (1-p)}{d^2} \right]$$

Hypothèses d'Equation-I

| | |
|---|---|
| $n_0$ | Nombre minimum total de ménages à interroger dans une zone d'étude en supposant une population infinie |
| $DEFF$ | Effect de design; fixé à 1.5<br>≤1.0=negative correlation of the outcome(s) of interest between household members; 1.0=no correlation of the outcome(s) of interest between household members; ≥1.0=positive correlation of the outcome(s) of interest between household members |
| $z_{1-\frac{\alpha}{2}}$ | Déviation Standard correspondant à un intervalle de confiance de 95% (1.96 pour alpha de 0.05) |
| $d$ | Précision (erreur acceptable); estimation de point; fixé à 0.2 |
| $p$ | Proportion de la population de l'étude devant se rendre dans un établissement de soins de santé de recrutement pour des conditions associées à de la fièvre et d'autres signes et symptômes (proportion capturée); fixé à 0.2 |
| $1-p$ | Proportion de la population de l'étude ne devant se rendre dans un établissement de soins de santé de recrutement pour des conditions associées à de la fièvre et d'autres signes et symptômes (proportion non capturée) |

L'effet de design est défini comme un facteur d'ajustement pour le regroupement naturel des comportements de santé comme principal résultat d'intérêt parmi les membres du ménage. Il rend compte d'une plus grande variance statistique et, par conséquent, d'une plus faible précision par rapport à l'échantillonnage aléatoire simple (15, 30, 33, 34). Pour HPAfrica, le DEFF peut être fixé de manière conservatrice à 1,5 sur la base d'une estimation de 1,42 résultant d'une taille moyenne de ménage de sept et d'un c'efficient de corrélation intracluster (ICC) de 0,07 provenant de l'étude TSAP (15, 31, 32, 35). Une proportion p de 0,2 pourra être considérée si aucune autre estimation n'est disponible ou p peut être basé sur des informations plus précises disponibles par site ou sur les expériences au cours de l'étude TSAP (Tableau 2): Madagascar, Isotry : p=0,01, Burkina Faso, Polesgo: p=0,9 (36). Combiner tous les hypothèses dans l'équation-I va avoir pour résultat un nombre global minimum ($n_0$) de 92 ménages à interroger par site.

En outre, $n_0$ pourra être pris en compte pour la répartition par âge et par sexe de chaque population de l'étude représentée par $q$, la proportion minimum supposée de stratification par groupe d'âge (<5 ans, ≥5 à <15 ans, ≥15 ans). Le facteur $q$ pourra être défini de façon arbitraire à 0,2 ou être basé sur les expériences précédentes: Éthopie, Butajira : $q$ =0,09, Burkina Faso, Polesgo : $q$ =0,23 (15). Appliquer $\widehat{q}$ à l'Equation-I pour une distribution binomiale, y compris ses postulats connexes, aura pour résultat le nombre minimum de ménages à interroger ($\overline{n_0}$) de 461 stratifié selon l'âge (Equation-II):

**Equation-II: Calcul de la taille d'échantillon basé sur la précision par site d'étude pour une population infinie avec stratification selon l'âge et population-poids par sous-zone ou strate:**

$$\overline{n_0} = \left( DEFF \cdot \left[ {z^2}_{1-\frac{\alpha}{2}} \cdot \frac{4 \cdot p \cdot (1-p)}{d^2} \right] \right) / q \qquad \overline{n_{0w}} = \overline{n_0} \cdot (n/N)$$

Hypothèses d'Equation-II

| | |
|---|---|
| $\overline{n_0}$ | Nombre total minimum de ménages à interroger dans une zone d'étude en supposant une population infinie et une stratification selon l'âge |
| $\overline{n_{0w}}$ | Minimum number of households to be interviewed by subarea or stratum |
| $n/N$ | Population size of subarea or stratum divided by the population size of the total study area |
| $DEFF$ | Effect de design; fixé à 1.5 |
| $z_{1-\frac{\alpha}{2}}$ | Déviation Standard correspondant à un intervalle de confiance de 95% (1.96 pour alpha de 0.05) |
| $d$ | Précision (erreur acceptable); estimation de point; fixé à 0.2 |
| $p$ | Proportion de la population de l'étude devant se rendre dans un établissement de soins de santé de recrutement pour des conditions associées à de la fièvre et d'autres signes et symptômes (proportion capturée); fixé à 0.2 |
| $1-p$ | Proportion de la population de l'étude ne devant se rendre dans un établissement de soins de santé de recrutement pour des conditions associées à de la fièvre et d'autres signes et symptômes (proportion non capturée) |
| $q$ | Proportion minimum de stratification par groupe d'âge, définie à 0,2 |

Les comportements de santé seront évalués deux fois dans les mêmes ménages si possible ou dans des ménages alternatifs, au cas où la population de l'étude serait instable en raison de la migration et / ou de taux de natalité / mortalité élevés. Des ménages supplémentaires, supposés être de 20% en moyenne (Tableau 2), peuvent être ajoutés pour tenir compte de la possible perte de suivi entre les deux évaluations. Eventuellement, l'échantillonnage stratifié pondéré en fonction de la population selon la proportion de la population des strates $(n/N)$ pour tenir compte de la probabilité inégale de sélection due à la répartition hétérogène des populations entre les strates (poids de la population $(n/N)$ par strat) sera appliqué à $\overline{n_0}$de 553 (37, 38).

**Echantillonnage avec et sans cadre d'échantillonnage de population**

Si une base d'échantillonnage de la population ou une liste de ménages compréhensif et à jour existe par DSS / HDSS ou recensement (39, 40), une sélection aléatoire informatisée des ménages en tant que PSU sera effectuée en utilisant SAS (Système d'analyse statistique, version 9.4, SAS Institute, Cary NC) en appliquant une sélection aléatoire simple en série sans remplacement pondéré selon la proportion de la population des strates ($n/N$). Les ménages sélectionnés avec des identifiants sont visualisés en utilisant des DDS / HDSS ou des outils de recensement. En cas de refus, d'absence d'un répondant ou de localisation d'un ménage abandonné, les enquêteurs sont formés pour visiter un ménage alternatif le plus proche du la côte droit ou gauche d'un ménage d'origine. Cela suit le principe de la proximité la plus proche comme vu dans la recherche précédente (41-44). Pour limiter, en outre, les probabilités de sélection inégale, donc les probabilités de sélection par les enquêteurs, nous présélectionnons un nombre arbitraire de cinq ménages alternatifs d'échantillonnage de population qui doivent être plus proches du ménage d'original plutôt que de demander par exemple une sélection en base de loterie ou une sélection dans le sens des aiguilles d'une montre (37, 41, 44). En outre, la notation de l'un (=le plus proche) à cinq (=le plus éloigné) est appliquée aux ménages alternatifs et les enquêteurs sont invités à suivre strictement la séquence de notation au cours de la procédure de sélection du ménage. La conformité des enquêteurs avec les règles de sélection des ménages originaux et alternatifs sera vérifiée quotidiennement. Les écarts seront étudiés et une formation complémentaire sera fournie aux enquêteurs au besoin.

S'il n'existe pas de cadre d'échantillonnage de la population, nous appliquerons une technique d'échantillonnage spatial stratifié pondérée par la population de proportion ($n/N$) (37). Guidés par des recherches antérieures, des points spatiaux aléatoires sont générés à l'aide de l'outil de génération de points aléatoires ArcGIS (Aeronautical Reconnaissance Coverage Geographic Information System, Redlands, CA, USA, version 10.2) (21, 23, 37, 40, 43, 45-51). L'outil place de la manière aléatoire des nombres spécifiés de points dans les entités d'un polygone correspondant à une sous-zone administrative ou à une strate. Les coordonnées X et Y définissent un point jusqu'à ce que la taille d'échantillon calculée par strate soit atteinte. Les points générés par ArcGIS sont convertis en un fichier image au format KML (Keyhole Markup Language) ou KMZ (Keyhole Markup landguage Zipped) et importés dans Google Earth Pro®. Un identifiant numérique unique et des coordonnées géographiques sont affectés à chaque point. L'annexe 4 illustre notre approche en utilisant des exemples du Ghana et de Madagascar. Les points spatiaux étiquetés avec des identifiants sont représentés sur des impressions de la taille d'une affiche de (60 x 60 à 60 x 90 cm) d'images satellite Google Earth Pro avec une haute résolution ou alternativement en utilisant une application de source ouverte pour la navigation et la cartographie automatiques hors ligne / en ligne pour localiser les points sélectionnés sur le terrain. Des récepteurs GPS (Global Positioning System) portables (Garmin-eTrex, Garmin Ltd., Lenexa, KS, USA) aident à vérifier les emplacements. Les récepteurs seront positionnés près d'un point localisé, dans une position statique et en plein air pour assurer une lecture sans obstacle. Les enquêteurs évaluent les points spatiaux de la présence d'un ménage en tant que PSU (24). En cas de refus, d'absence du répondant ou de localisation d'un point non résidentiel, les enquêteurs sont formés pour visiter un point spatial alternatif présélectionné le plus proche de la droite ou de la gauche d'un point original et l'évaluer pour la présence d'un ménage (41-43, 52). Pour limiter les chances de probabilité de sélection inégale, nous présélectionnons au moins cinq points spatiaux alternatifs à proximité immédiate du point d'origine qui représentent une structure de taille appropriée et de forme rectangulaire ou carrée à partir de l'imagerie satellite Google Earth Pro outil de mesure de distance. Commue pour les sites ayant un cadre d'échantillonnage de la population, la notation d'un à cinq est appliquée aux points spatiaux alternatifs et les enquêteurs doivent suivre la séquence de notation. La conformité des enquêteurs avec les règles de sélection des ménages originaux et alternatifs sera vérifiée quotidiennement. Les écarts seront étudiés et une formation complémentaire sera fournie aux enquêteurs au besoin. Dans le cas où un point spatial est placé dans l'équidistance de deux structures (ou plus), l'intervieweur saute le point spatial original et choisit un point spatial alternatif présélectionné suivant la séquence de notation. Dans le cas où deux points spatiaux originaux (ou plus) sont placés sur la même structure, l'intervieweur évalue la structure de la présence d'un ménage et choisit un second point spatial alternatif (ou plus) présélectionné après la séquence de notation.

Indépendamment de la présence ou de l'absence d'un cadre d'échantillonnage de la population, un ménage unifamilial à un étage sera directement contacté pour participer à l'étude. Cependant, un ménage seulement est inscrit dans une structure multifamiliale à un seul étage selon les procédures suivants : l'enquêteur entre dans la structure et évalue le nombre total de ménage : le premier ménage du côté droit / gauche (selon la construction intérieure) de la structure est approché ; si le premier ménage ne participe pas, le second ménage du côté droit / gauche de la même structure est approché. L'enquêteur continue jusqu'à ce qu'un ménage par structure soit inscrit. De même, un ménage seulement est inscrit dans une structure multifamiliale à plusieurs étages. Après avoir évalué le nombre total de ménage présents, l'enquêteur se rapproche du premier ménage ne participe pas, le deuxième ménage le plus proche de l'entrée sur le même étage / suivant (en fonction de la construction intérieure) est approché ; l'enquêteur continue jusqu'à ce qu'un ménage par structure soit inscrit. L'enquêteur choisit un point spatial alternatif présélectionné après la séquence de notation au cas où aucun ménage ne peut être inscrit.

**Fréquence de la collecte de données**
Les points résidentiels seront visités deux fois pour des entrevues au cours du programme SETA pour prendre en compte un possible impact lié à la saison, sur le comportement sanitaire (53-57). Les enquêtes auront lieu à différentes saisons - une à la fin de la saison sèche ou au moins un mois après son début et une vers la fin de la saison des pluies ou au moins un mois après son début (Tableau 3). Actuellement, l'étude HPAfrica est en cours au Burkina Faso, au Ghana et à Madagascar et devrait se poursuivre dans les autres pays au cours des prochains mois.

**Critères d'inclusion et d'exclusion**
Les membres du ménage de tout âge et des deux sexes qui vivent dans la zone d'étude au jour de l'interview seront éligibles pour l'inclusion. Un ménage sera exclu si le répondant désigne refuse de participer ou n'est pas disponible après trois visites consécutives. Tous les visiteurs et individus dont le lieu de résidence n'est pas connu ou est en-dehors de la zone d'étude seront exclus.

Pour raison de cette enquête, un ménage est défini comme une personne ou un groupe de personnes apparentées ou non apparentées vivant dans la même unité d'habitation, reconnaissant un individu adulte comme chef du ménage, partageant les mêmes installations domestiques, et subvenant indépendamment à leurs besoins en termes de nourriture et des autres éléments essentiels pour vivre (36).

L'interview aura lieu exclusivement avec le répondant, qui est un membre adulte du ménage à l'âge de majorité légale spécifique au pays. Cette personne peut être identifiée comme décideur par les membres du même ménage et sert de substitut pour un ménage entier (15). D'autres définitions pertinentes sont expliquées subséquemment.

**Collecte de données**
Les intervieweurs de site recevront une formation sur la localisation des points géographiques, l'identification des répondants, les procédures de consentement éclairé en mettant l'accent sur la participation volontaire, et sur le déploiement de formulaires d'étude standardisés et pré-testés avant le lancement de HPAfrica (58). Les formulaires de consentement éclairé et d'étude seront traduits dans la langue officielle du pays par deux traducteurs bilingue indépendants parlant couramment l'anglais et les locuteurs natifs de la langue cible qui connaissent le concept et la terminologie des formulaires. Les deux traductions seront comparées pour les divergences avec les traducteurs et un coordinateur, et un consensus sera recherché. La traduction avancée synthétisée sera traduite et traduite de la langue cible vers l'anglais par deux traducteurs indépendants, aveugles aux formes originales. Un consensus sur les écarts de traduction et les formulaires originaux seront recherchés avec les traducteurs et un coordinateur. Les formes traduites feront l'objet d'un test pilote parmi un échantillon de commodité des ménages de la population cible avant leur finalisation pour assurer la compréhensibilité interculturelle et l'équivalence sémantique, idiomatique, expérientielle et conceptuelle. Un rapport de traduction sera préparé (58-60).

**Gestion de données**

La gestion des données dépendra du mode de collecte, qui sera principalement électronique plutôt que sur support papier. Les expériences passées ont montré les inconvénients suivants de la collecte de données sur papier: une forte probabilité d'erreurs lors du remplissage des formulaires d'étude, et le besoin de numérisation des données, qui est une étape supplémentaire, laborieuse, qui consomme du temps et sujette aux erreurs. La capture des données électroniques à l'aide d'une application pour un appareil mobile, comme un smartphone ou une tablette, réduit ces facteurs limitants à la qualité des données.

Il peut y avoir une période pilote au cours de laquelle des données sur support papier et sans papier seront collectées avant la mise en œuvre complète de la collecte de données électroniques. Ce dernier utilise l'application Android « HPA Collect » (Google Android 5.0.1 9API 23, disponible à l'adresse https://play.google.com/store/apps/details?id=anint.ivi.hpa) et la plate-forme « HPA Web » (CSS, JavaScript et JSP, disponible sur http://hpa.ivi.int/), qui sont accompagnés d'un dictionnaire de variables contenant les codes, la signification et les propriétés des variables au moins en anglais et en français. Les deux, « HPA Collect » et « HPA Web », seront conçus spécifiquement pour les besoins de cette étude. L'expertise d'IVI en ingénierie informatique permet la construction fondamentale, la configuration et le développement des composants nécessaires. Différent de nombreux programmes de source ouverte, une fonction d'installation serveur permet le stockage de toutes les données originales collectées sur le serveur institutionnel en plus des fonctionnalités supplémentaires pour la vérification des erreurs profondes, les relations logiques entre variables et formulaires, les fonctions de recherche et l'analyse des données collectées. Une connectivité Internet sans fil stable utilisant des navigateurs par défaut est nécessaire pour assurer une synchronisation simultanée, attribuable, originale et précise des données entre « HPA Collect » et « HPA Web ». L'accès à « HPA Collect » et « HPA Web » est protégé par un mot de passe. L'annexe 5 affiche le diagramme du système, qui a les caractéristiques suivantes:

**Structure ou fonctionnalités de « HPA Collect »**

- **Gestion des utilisateurs:** Une interface utilisateur simple fournit différents privilèges aux utilisateurs pour entrer, modifier (sauf pour les étiquettes d'étude) et exporter ou transférer des données. Il établit une piste d'audit qui enregistre lorsque les utilisateurs entrent des données dans le serveur.
- **Saisie de données:** Les données sont entrées directement dans le smartphone ou la tablette. Les champs de saisie limitent les réponses possibles à des réponses plausibles uniquement en offrant différentes méthodes de saisie:
    - Modifier le texte: texte libre
    - Groupe radio / bouton Multi-clic: choix unique (radio group/Multi click button)
    - Case à cocher: choix multiple
    - Sélecteur de date et d'heure: date et heure d'entrée
    - Spinner: bouton déroulant

  Des fonctionnalités supplémentaires permettent à l'enquêteur de gagner du temps en transférant les données collectées plus tôt aux champs de données requis à un stade ultérieur de l'interview en effectuant des vérifications de vraisemblance pour divers champs de saisie de données et en reconnaissant facilement les modèles de saut. Les vérifications d'erreurs, les notifications de données manquantes, les erreurs de code et les erreurs logiques sont également incluses.
- **Vue des données:** Les données brutes entrées à l'origine peuvent être vues dans le même format lisible par l'homme et la machine, à savoir le format JSON, étant donné qu'elles seront transférées vers un serveur de base de données (Windows, MySQL). Des fonctions supplémentaires telles que «effacer» pour effacer tous les enregistrements, «réinitialiser» pour revenir à la page de vue de données principale, et «rechercher» pour rechercher une étiquette d'étude sont disponibles.
- **Exporter :** Toutes les données entrées seront envoyées au serveur IVI via une connexion Internet sans fil. Chaque enregistrement de formulaire ayant une étiquette d'étude unique du ménage inscrit est transformé en une requête d'insertion SQL et inséré sur le serveur

uniquement s'il n'y a pas d'étiquette d'étude en double trouvée. L'insertion de données sera ignorée dans le cas d'étiquettes d'étude dupliquées.

- **Exporter ers local :** Tous les données entrées seront sauvegardées trois fois dans un dossier de stockage public de l'appareil mobile au format JSON et txt. Les fichiers txt contiennent des données de chaque formulaire individuel. Une version de fichiers JSON contient des données par chaque formulaire individuel et une deuxième version de fichiers JSON contient toutes les données d'un ménage inscrit. Les données au format JSON peuvent être extraites par USB ou connexion Internet sans fil.
- **Rapport :** Cette fonctionnalité permet à un utilisateur de signaler toute requête à l'administrateur principal de « HPA Collect » à IVI par e-mail our par une application tierce.
- **Paramètres:** la possibilité de changer la langue du système entre l'anglais et le français est fournie.
- **À propos de:** La version actuellement installée de «HPA Collect» est affichée.

**Structure ou caractéristiques de « HPA Web »**

- **Accueil:** une introduction à la plateforme est donnée et un tutoriel en français et en anglais sera accessible.
- **Aperçu du Formulaire:** Les formulaires d'étude sont affichés séparément montrant chaque question et les données d'entrée correspondantes en tant que variables définies.
- **Modifier le formulaire:** cette fonction permet la correction des données téléchargées. Il est uniquement accessible aux gestionnaires de données de chaque pays participant et à IVI.
- **Cherche:** Les étiquettes d'étude spécifiques données à chaque formulaire d'étude peuvent être filtrées.
- **Dictionnaire des variables:** Le dictionnaire variable peut être téléchargé.
- **Exporter:** Les données peuvent être converties à partir du serveur dans un fichier Excel transférable.
- **Contactez-nous:** Une correspondance rapide et facile entre les utilisateurs et le gestionnaire de données à IVI est donnée.

Les données seront examinées quotidiennement par le personnel clé de l'étude et vérifiées en termes de consistance et d'exactitude avant l'analyse des données. La qualité des données peut être vérifiée en sélectionnant un sous-ensemble de 5% pour la validation par rapport aux données originales sur support papier, si possible (58). Les noms des membres du ménage ne seront pas liés aux formulaires d'étude de HPAfrica et ne seront pas enregistrés dans la base de données afin d'assurer la confidentialité. L'accès à la base de données sera limité au personnel d'étude autorisé uniquement et les données seront conservées dans un endroit sécurisé et protégé. Des fichiers de sauvegarde provisoires périodiques et au moins trois fichiers de sauvegarde définitifs de la base de données stockés dans un endroit sécurisé et verrouillé éviteront la perte de données et garantiront la sécurité des données. Toutes les données seront conservées pendant au moins cinq ans.

**L'analyse des données**

Les poids d'échantillonnage combinés seront calculés pour tenir compte des différences possibles dans les probabilités de sélection et pour accroître la précision des estimations de l'étude (22, 61). Les poids sont générés dans une approche à plusieurs niveaux pour prendre en compte des probabilités de sélection de par exemple les  ménages, les poids inverses au sein des ménages (c'est-à-dire la structure à plusieurs étages à plusieurs familles, la structure à plusieurs étages multifamiliale) et les poids inverses parmi les membres du ménage participant à l'étude HPAfrica (21, 43). Les outils de source ouverte, les données de recensement et les données générées par HPAfrica telles que la taille de la population, le nombre de ménages ou de structures, le nombre de ménages dans les structures multifamiliales / multi-étages et la taille des ménages par strate seront utilisés pour calculer poids (62, 63).

Des analyses descriptives telles que les fréquences absolues et relatives, les médianes ou les moyennes arithmétiques seront effectuées pour construire une description contemporaine de la population de base.

Les analyses de base comprendront le calcul des proportions stratifiées selon l'âge et le sexe, des comportements sanitaires *génériques* et *réels*, y compris l'utilisation des soins de santé, pour divers signes et symptômes ou combinaisons de signes et de symptômes. Ces analyses permettront une classification graduelle basée sur la sévérité de la maladie, incluant un intervalle de confiance de 95%. Le comportement de santé sera ajusté pour un effet de regroupement naturel présumé parmi les membres du ménage. Des données stratifiées les plus à jour sur la population par site et les données démographiques dérivées de HPAfrica seront utilisées pour générer les proportions pour l'ajustement des calculs d'incidence.

Les informations socio-économiques collectées seront utilisées pour construire des indices de richesse des populations étudiées en appliquant des analyses factorielles basées sur la méthode des composantes principales (38). De plus, des analyses de régression bi et multivariée seront effectuées pour identifier les facteurs de confusion potentiels ou la modification des effets en utilisant les scores factoriels.

Les analyses de régression bi et multivariée seront effectuées en utilisant les scores factoriels pour l'évaluation des variables suivantes:

- Données socioéconomiques telles que l'éducation, le revenu (64), l'assurance maladie, le logement et les biens des ménages
- Hygiène et assainissement tels que l'accès aux toilettes, les sources d'eau, la manipulation des aliments, l'élimination des déchets et les contacts avec les animaux
- Comportement sanitaire: *réel* (stratifié selon l'âge et le sexe, gravité, fréquence), *générique* (stratifié selon l'âge et le sexe, gravité, classement par choix)
- Modalités de déplacement vers les établissements de santé, stratifiées selon l'âge
- Saisonnalité liée à l'hygiène et à l'assainissement, comportement sanitaire, modalités de déplacement
- Facteurs culturels / religieux / ethnographiques, stratifiés selon l'âge
- Statut de vaccination parmi les enfants ≤ 6 ans (18, 19)
- Connaissance et perception des maladies courantes

**Considérations éthiques**

Il n'y a pas d'avantages directs pour les ménages inscrits et leurs membres. Les avantages indirects pour la population générale sur les sites seront les informations mises à jour sur le fardeau des maladies transmissibles, y compris les facteurs influençant la transmission de la maladie et l'infection. L'étude HPAfrica génère des évidences pour aider les décideurs à introduire des mesures appropriées pour la prévention et le contrôle des maladies.

Ce protocole a fait l'objet d'examens par le Comité de Révision Institutionnelle d'IVI et des comités locaux spécifiques aux sites, comme indiqué dans la section «Références éthiques». Pendant et après l'étude HPAfrica, toutes les données des sujets inscrits seront gardés avec une stricte confidentialité et ne seront pas divulguées à une tierce partie par aucun membre de l'équipe de recherche. La protection par mot de passe des appareils et de la base de données est utilisée pour une stricte confidentialité. Toutes les données sur support papier (formulaires de consentement) seront conservées dans un endroit sécurisé et verrouillé. Les informations confidentielles stockées sur des ordinateurs et des sources de données sur support papier seront seulement mises à la disposition des co-chercheurs et du personnel d'IVI directement impliqués dans les activités d'étude de HPAfrica.

**Abbréviations**

JSON: JavaScript Objective Notation: CSS: Cascading Style Sheets: JSP: Java Server Page; SQL: Structured Query Language

## Définitions/Terminologie

| | |
|---|---|
| **A** | |
| Automédication | Fait de se médicamenter soi-même ou de traiter sa propre maladie ou son état sans supervision médicale ou ordonnance. |
| **B** | |
| **C** | |
| Choléra | Maladie infectieuse de l'intestin grêle qui peut être mortelle causée par la bactérie *Vibrio cholerae* ; elle se propage par voie fécale-orale par l'ingestion d'eau et d'aliments contaminés et provoque fièvre, diarrhée aqueuse sévère, vomissements et déshydratation. |
| Confusion/Vertiges/Perte de connaissance | L'état de désorientation ou d'absence de clarté d'esprit ; l'état anormal de réponse aux stimuli dans l'environnement; faible, wooziness, faiblesse, instabilité ou tendance à tomber; l'interruption de la conscience de soi et de son environnement. |
| Convulsion | Mouvement soudain, violent et irrégulier d'un membre/d'une partie du corps ou de tout le corps causé par une contraction involontaire des muscles. |
| Coqueluche (Pertussis) | La coqueluche est une maladie bactérienne très contagieuse. Les symptômes d'un froid commun comme nez qui coule, fièvre et toux sont suivis par des semaines de toux sévère et un son aigu de la coqueluche ou haletant quand une personne malade inspire; toux sévère peut être suivie de vomissements, rupture des côtes ou fatigue extrême. Les très jeunes et les personnes âgées infectées peuvent avoir peu ou pas de toux, ou peuvent avoir des périodes de respiration interrompue pendant lesquelles elles ne respirent pas. La coqueluche est transmise par la bactérie *Bordetella pertussis* par toux et éternuements d'une personne infectée. |
| **D** | |
| Déshydration | Une condition de perte excessive d'eau/d'hydratation par le corps. |
| Diarrhée | Une condition d'évacuer les matières fécales du corps très fréquemment et sous forme plutôt liquide que solide. |
| Difficultés respiratoires/ Respiration sévère | Une condition de la respiration anormale accompagnée d'une respiration sifflante, d'une douleur thoracique, d'un tirage thoracique, d'un stridor, d'un gonflement de la poitrine ou d'une toux par exemple. |
| Diphthérie | Maladie hautement contagieuse, parfois mortelle, causée par la bactérie *Corynebacterium diphtheriae*. Elle se transmet de personne à personne, par l'air ou les objets contaminés et affecte les voies respiratoires supérieures. Les symptômes courants sont maux de gorge, gonflement des glandes/ ganglions lymphatiques, malaise, fièvre/frissons, substance membraneuse/matière grise et épaisse qui se forme dans la gorge/sur les amygdales et gêne/accélère la respiration (toux rauque) et gêne pour avaler. Une forme de diphtérie qui implique la peau, les yeux et les organes génitaux est également signalée et se caractérise par une peau douloureuse, rouge et enflée. |
| Douleur abdominale/ Douleur intestinale | Douleur (aiguë/courte durée ou chronique/longue durée) qui survient entre la poitrine et l'aine (souvent désignée comme la région de l'estomac/du ventre) |
| Douleur intestinale/Douleur abdominale | Douleur (aiguë/courte durée ou chronique/longue durée) qui survient entre la poitrine et l'aine (souvent désignée comme la région de l'estomac/du ventre) |

| | |
|---|---|
| Douleur/Maux | État caractérisé par une douleur sourde continue/prolongée dans une partie du corps ; la sensation/souffrance/inconfort physique causée par une maladie/une blessure/quelque chose qui fait mal au corps. |
| **E** | |
| Éducation formelle | Modèle d'éducation systématique et organisée structuré et administré selon un ensemble de lois et de normes ; les institutions d'éducation formelle sont organisées de façon administrative, physique et pédagogique et exigent des étudiants une assiduité minimum ; il implique des évaluations intermédiaires et finales afin d'accéder au niveau d'apprentissage suivant ; il délivre des certificats et des diplômes conformément à un ensemble de règles strictes. |
| Essoufflement/ Respiration rapide | Fait de respirer anormalement rapidement et profondément ; à une fréquence de >20 respirations par minute pour les adultes, >30 respirations par minute pour les enfants et >45 respirations par minute par les bébés. |
| Établissement de santé | Lieux ou institutions où des soins de santé sont prodigués ; dont les hôpitaux, les cliniques, les centres de santé, les postes de santé et les centres de soins spécialisé. |
| Éternuement | Fait d'expulser soudainement l'air par le nez et la bouche avec un bruit important caractéristique. |
| **F** | |
| Faiblesse/Malaise/Fatigue | État de se sentir fatigué/épuisé/à bout de forces ; sentiment général d'inconfort/malaise/de ne pas être en bonne santé ou heureux. |
| Fatigue/Malaise/ Faiblesse | État de se sentir fatigué/épuisé/à bout de forces ; sentiment général d'inconfort/malaise/de ne pas être en bonne santé ou heureux. |
| Fièvre | Température corporelle anormalement élevée (habituellement au-dessus de 38°C). |
| Fièvre <3 jours | Température corporelle anormalement élevée (habituellement au-dessus de 38°C) qui dure moins de 3 jours et apparaît en continu. |
| Fièvre >3 jours | Température corporelle anormalement élevée (habituellement au-dessus de 38°C) qui dure plus de 3 jours et apparaît en continu. |
| Fièvre jaune | Maladie infectieuse aiguë, pathogène pour l'homme et virale caractérisée par l'apparition soudaine de symptômes comme fièvre, frissons, perte d'appétit, nausée, douleurs musculaires et maux de tête qui disparaissent généralement en quelques jours ou sont suivis de symptômes plus graves comme jaunisse, fièvre forte et hémorragie. La maladie est causée par un virus du genre Flavivirus et est transmise par la piqûre d'un moustique infecté. |
| Fièvre typhoïde | Maladie infectieuse bactérienne potentiellement mortelle causée par *Salmonella typhi* ; elle se transmet par la voie fécale-orale par les aliments et l'eau contaminés ou de personne à personne. Les symptômes courants sont forte fièvre, maux de tête, douleur abdominale, constipation/diarrhée et éruption cutanée. Des complications graves (perforation de l'intestin) sont rapportées. |
| Frissons | Sensation de froid qui peut être accompagnée de frissons/tremblements du corps et de pâleur de la peau |
| **G** | |
| Gonflement/Œdème | État d'accumulation excessive de liquide aqueux/gonflement des cavités ou des tissus du corps ; couramment constaté dans les mains/bras/pieds/jambes ; augmentation du volume de tissu. |
| Grippe | Maladie infectieuse hautement contagieuse des voies respiratoires causée par le virus de la grippe ; elle se propage de personne à personne |

| | |
|---|---|
| | par les particules expulsées par la toux/les éternuements/la parole et les objets contaminés. Les symptômes courants sont fièvre, douleurs dans les muscles/bras/jambes, frissons/sueur, maux de tête, fatigue/faiblesse, nez qui coule/congestion nasale, éternuements, maux de gorge et toux sèche et persistante. |
| Guérisseur traditionnel | Personne qui cherche à maintenir la santé aussi bien qu'à prévenir, diagnostiquer et améliorer ou traiter les maladies en utilisant un ensemble de connaissances, compétences et pratiques basées sur des théories, des croyances et des expériences. |
| **H** | |
| *Haemophilus influenzae* de type B | Bactérie qui provoque une série de maladies et se propage par les particules expulsées par la toux ou les éternuements, et surviennent surtout chez l'enfant de moins de 5 ans ; les symptômes courants sont fièvre, maux de tête et raideur dans le cou jusqu'à l'état septique. Elle peut causer otites, cellulites (infection des tissus mous), arthrite, infections des voies respiratoires supérieures, pneumonies, méningites (avec dommages potentiels au cerveau) et épiglottites (avec obstruction des voies aériennes). |
| Hémorragie (interne/externe)/ Perte de sang | État caractérisé par une perte de sang du système vasculaire, soit de façon interne à l'intérieur du corps (hémorragie interne) soit de façon externe par un orifice naturel ou une lésion de la peau (hémorragie externe) ; sang qui s'échappe d'un vaisseau sanguin endommagé. |
| Hépatite B | Maladie infectieuse causée par le virus de l'hépatite B (VHB) qui affecte le foie (infection hépatique aiguë/chronique ; peut entraîner une insuffisance hépatique/un cancer/une cirrhose). Le virus se transmet de personne à personne par le sang/le sperme/les autres fluides corporels. Les symptômes courants sont fièvre, malaise/faiblesse/fatigue, perte d'appétit, nausée/vomissements, gêne/douleur abdominale, urines foncées, douleurs articulaires et jaunisse. |
| Hépatite E | Maladie infectieuse causée par le virus de l'hépatite E (VHE) qui affecte le foie (infection hépatique aiguë uniquement ; peut entraîner une insuffisance hépatique aiguë). Le virus se propage par les voies fécales-orales par l'ingestion d'eau ou d'aliments contaminés, les animaux contaminés (de façon zoonotique) et par le sang. Les symptômes courants sont fièvre, malaise, perte d'appétit, diarrhée, nausée, gêne abdominale, urines foncées, douleurs articulaires et jaunisse. |
| **I** | |
| **J** | |
| Jaunisse | Maladie qui fait jaunir la peau/le blanc des yeux. |
| **K/L** | |
| **M** | |
| Malaise/Fatigue/ Faiblesse | État de se sentir fatigué/épuisé/à bout de forces ; sentiment général d'inconfort/malaise/de ne pas être en bonne santé ou heureux. |
| Maux de tête | Douleur continue dans une région de la tête. |
| Maux/Douleur | État caractérisé par une douleur sourde continue/prolongée dans une partie du corps ; la sensation/souffrance/inconfort physique causée par une maladie/une blessure/quelque chose qui fait mal au corps. |
| Médecin | Une personne compétente/formée aux soins ; une personne éduquée, cliniquement expérimentée et autorisée à pratiquer la médecine. |
| Ménage | Personne ou groupe de personnes apparentées ou non qui vivent ensemble dans la même unité d'habitation, qui reconnaissent un adulte de sexe masculin ou féminin comme le chef du ménage, qui partagent |

| | |
|---|---|
| | les mêmes installations domestiques, qu'on considère comme formant une unité, et qui subviennent à leurs besoins en termes de nourriture et des autres éléments essentiels pour vivre. Un ménage peut être situé dans une unité d'habitation unique (bâtiment à un seul étage) ou dans un ensemble de logements collectifs (bâtiment à plusieurs étages). |
| Méningite | Inflammation virale/bactérienne/fongique infectieuse parfois mortelle des membranes (méninges) qui entourent le cerveau et la moelle épinière ; elle se propage de personne à personne par les particules expulsées par la toux/les éternuements/la parole et les objets contaminés. Les symptômes courants sont forte fièvre, maux de tête sévères, nausée/vomissements, confusion, convulsions ; perte d'appétit et de la soif, fatigue, sensibilité à la lumière, éruption cutanée et raideur au cou. |
| **N** | |
| Nausée/Vomissements | Sensation de malaise dans l'estomac avec tendance à vomir ; fait d'éjecter une partie/tout le contenu de l'estomac principalement par la bouche. |
| Nez qui coule | État résultant de la production par le nez (tissus nasaux et adjacents et vaisseaux sanguins) de mucus/drainage excessif, qui coule du nez pour retirer quelque chose. |
| **O** | |
| Œdème/Gonflement | État d'accumulation excessive de liquide aqueux/gonflement des cavités ou des tissus du corps ; couramment constaté dans les mains/bras/pieds/jambes ; augmentation du volume de tissu. |
| Oreillons | Maladie infectieuse virale causée par le virus des oreillons qui affecte principalement les glandes parotides qui sont situées sous et devant les oreilles ; elle se propage de personne à personne par la salive infectée/les particules de salive infectées expulsées par la toux/les éternuements/la parole et les objets contaminés. Les symptômes courants sont fièvre, gonflement d'une/des deux glandes parotides, maux de tête ; douleurs musculaires, faiblesse/fatigue, perte d'appétit et douleurs en mâchant/avalant ; une complication connue est une possible perte d'audition. |
| **P** | |
| Paludisme | Maladie infectieuse parfois mortelle causée par un parasite (*Plasmodium* spp.) transmis principalement par la piqûre de moustiques infectés, en dehors de la transmission mère-enfant et par le sang. Les symptômes courants sont attaques récurrentes de frissons, sueurs, forte fièvre, maux de tête, vomissements et diarrhée. |
| Personne interrogée | Membre adulte (à l'âge légal de majorité du pays concerné) du ménage qui est un décideur en ce qui concerne les soins de santé/l'utilisation des soins de santé pour le ménage entier à l'intérieur du site d'étude ; cette personne adulte parle ainsi au nom de tous les membres du ménage. Cette personne peut être identifiée par les autres membres du même ménage comme la personne qui est principalement impliquée dans les soins quotidiens des membres du ménage. |
| Perte de connaissance/ Vertiges/Confusion | L'état de désorientation ou d'absence de clarté d'esprit ; l'état anormal de réponse aux stimuli dans l'environnement; faible, wooziness, faiblesse, instabilité ou tendance à tomber; l'interruption de la conscience de soi et de son environnement. |
| Perte de poids | Baisse (volontaire/involontaire) du poids du corps. |
| Perte de sang/ Hémorragie | État caractérisé par une perte de sang du système vasculaire, soit de façon interne à l'intérieur du corps (hémorrage interne) soit de façon |

| | |
|---|---|
| | externe par un orifice naturel ou une lésion de la peau (hémorragie externe) ; sang qui s'échappe d'un vaisseau sanguin endommagé. |
| Pharmacie | Endroit où seuls des médicaments (ordonnance requise/non requise) sont préparés, conservés, composés et dispensés. |
| Pneumocoque | Une inflammation infectieuse bactérienne des poumons parfois mortelle qui affecte souvent les personnes immunodéprimées et est causée par la bactérie *Streptococcus pneumoniae* ; elle se propage de personne à personne par les particules expulsées par la toux/les éternuements/la parole et les objets contaminés. Les symptômes courants sont fièvre, frissons, toux (avec mucosités/pus), douleurs thoraciques, difficulté à respirer/essoufflement, fatigue, nausée/vomissements et diarrhée ; des symptômes rares sont septicémie, épanchement pleural et empyème. |
| Polio | Maladie virale infectieuse, très contagieuse, parfois mortelle, causée par le poliovirus qui cause dans sa forme la plus sévère paralysie et difficultés à respirer ; il se transmet par la voie fécale-orale par ingestion d'eau et d'aliments contaminés et de personne à personne. Les symptômes courants sont fièvre, maux de gorge, maux de tête, vomissements, fatigue, maux de dos, de cou/raideur au cou, faiblesse des muscles/muscles douloureux, méningite, perte de réflexes, membres lourds/pendants. |
| Occupation | A job, or means of earning a wage/living; often requires the mastery of a complex set of knowledge, tasks, duties and skills acquired through formal education and/or practical experience. |
| **Q** | |
| **R** | |
| Respiration rapide/ Essoufflement | Fait de respirer anormalement rapidement et profondément ; à une fréquence de >20 respirations par minute pour les adultes, >30 respirations par minute pour les enfants et >45 respirations par minute par les bébés. |
| Respiration sévère/ Difficultés respiratoires | Une condition de la respiration anormale accompagnée d'une respiration sifflante, d'une douleur thoracique, d'un tirage thoracique, d'un stridor, d'un gonflement de la poitrine ou d'une toux par exemple. |
| Revenus/Salaire | Argent ou autre forme de paiement que quelqu'un reçoit périodiquement/régulièrement en échange de biens ou de services. Provient généralement d'un emploi/d'une profession. |
| Rotavirus | Infection virale causée par le rotavirus qui cause principalement des diarrhées pour les enfants de moins de 5 ans ; elle se propage par la voie fécale-orale, de personne à personne et par les objets contaminés. Les symptômes courants sont diarrhée aqueuse, fièvre, vomissements, douleurs abdominales et déshydratation. |
| Rougeole | Maladie infectieuse parfois mortelle des voies respiratoire (nez/gorge) causée par un virus qui affecte principalement les enfants de moins de 5 ans ; elle se propage de personne à personne par les particules expulsées par la toux/les éternuements/la parole et les objets contaminés. Les symptômes courants sont fièvre, toux sèche, nez qui coule, inflammation des yeux, maux de gorge, larges plaques sur la peau et points blancs dans la bouche et sur les joues. |
| Rubéole | Maladie infectieuse virale contagieuse connue pour son éruption cutanée rouge distinctive qui est causée par le virus de la rubéole ; elle se transmet de personne à personne (toux/éternuements/parole), de la mère à l'enfant par le sang et les objets contaminés. Les symptômes courants sont fièvre modérée, maux de tête, nez bouché/qui coule, yeux rouges/inflammation des yeux, ganglions lymphatiques gonflés et |

| | |
|---|---|
| | sensibles à la base du crâne/à l'arrière du cou/derrière les oreilles, douleurs articulaires et éruption cutanée rose qui se déplace du visage au torse, aux bras et aux jambes. |
| **S** | |
| Soins de santé - généraux | Soins de santé pour les maladies qui n'ont aucun lien avec l'apparition de maladies associées aux signes et symptômes ou les dates de début de maladies. |
| Soins de santé - réels | Soins de santé liés l'apparition de maladies associées aux signes et symptômes ou les dates de début de maladies. |
| **T** | |
| Témoin | Personne adulte (à l'âge légal de majorité du pays concerné), qui est indépendante de l'étude, qui ne peut pas être indûment influencée par le personnel de l'étude, qui participe au processus de consentement éclairé si le sujet/le représentant légal du sujet est analphabète, et qui lit (lettré) le formulaire de consentement éclairé et toute autre information écrite présentée au sujet. |
| Tétanos | Une maladie infectieuse caractérisée par des spasmes musculaires qui commencent généralement dans la mâchoire et progresse vers le reste du corps. Les spasmes durent généralement quelques minutes et se produisent fréquemment pendant les 3-4 semaines au début de la maladie, ils peuvent être si graves qu'ils peuvent causer des fractures osseuses ou la mort lorsqu'ils affectent les muscles respiratoires. Elle peut être caractérisée par d'autres symptômes comme fièvre, sueurs, maux de tête, difficultés à avaler, pression artérielle élevée et augmentation du rythme cardiaque. La maladie est causée par une infection par la bactérie *Clostridium tetani* qui se trouve dans sol, saliva, poussière et fumier. La bactérie entre par une lésion cutanée (par exemple, coupure, blessure par ponction) par un objet contaminé. Il produit des toxines qui interfèrent avec les contractions musculaires qui résultent des spasmes typiques. |
| Toux | Fait d'expulser de façon soudaine/rapide et bruyamment l'air des poumons ; souvent de façon involontaire afin de dégager les voies respiratoires des poumons de fluides/mucus/autres matières |
| Troubles cardiaques | Tout trouble que affecte le cœur ; maladies qui impliquent des vaisseaux sanguins rétrécis/bloqués qui peuvent entraîner une attaque cardiaque/douleur thoracique/accident vasculaire cérébral ; maladie qui affecte le muscle/les valves/le rythme du cœur. |
| Troubles de la pression artérielle | Pression artérielle élevée/faible ou pression artérielle qui présente des fluctuations excessives. |
| Tuberculose | Maladie infectieuse bactérienne (*Mycobacterium tuberculosum*), contagieuse, potentiellement grave qui affecte principalement les poumons en plus des reins et de la colonne vertébrale/du cerveau ; elle se transmet de personne à personne par les particules expulsées par la toux/les éternuements/la parole. Les symptômes courants d'une tuberculose active sont toux (avec/sans sang), douleur thoracique, perte de poids, fatigue, fièvre, sueurs nocturnes, frissons et perte d'appétit. |
| **U** | |
| **V** | |
| Vertiges/Confusion/Perte de connaissance | L'état de désorientation ou d'absence de clarté d'esprit ; l'état anormal de réponse aux stimuli dans l'environnement; faible, wooziness, faiblesse, instabilité ou tendance à tomber; l'interruption de la conscience de soi et de son environnement. |

| | |
|---|---|
| VIH/SIDA | Maladie infectieuse causée par le virus de l'immunodéficience humaine (VIH) qui affecte/endommage le système immunitaire et entraîne le syndrome immunodéficitaire acquis (SIDA), une maladie chronique et potentiellement mortelle ; il se transmet par les liquides corporels infectieux comme le sang/le sperme/les autres fluides corporels. Les symptômes courants d'une infection aiguë/précoce sont fièvre, fatigue, maux de tête, douleurs articulaires, éruption cutanée, maux de gorge, ganglions lymphatiques enflés, diarrhée, perte de poids, candidose orale et zona. La maladie non traitée évolue en SIDA ; les symptômes causés par un système immunitaire gravement endommagé sont sueurs, fièvre récurrente, diarrhée chronique, lésions sur la langue et dans la bouche, fatigue, perte de poids et éruptions cutanées. |
| Visiteur | Personne (apparentée ou non) qui rend visite à un ménage pour passer du temps avec les membres du ménage ; un visiteur ne vit pas avec les membres du ménage dans la même unité d'habitation, ne partage pas les mêmes installations domestiques, la même nourriture et autres éléments essentiels à la vie que les membres du ménage, et ne constitue pas une unité avec les membres du ménage. |
| Voisin | Personne (apparentée ou non) vivant dans l'habitation à côté de celle d'un ménage cible ; un voisin ne vit pas avec les membres d'un ménage (à côté) dans la même unité d'habitation, ne partage pas les mêmes installations domestiques, aliments ou autres éléments essentiels à la vie que les membres du ménage (à côté), et ne constitue pas une unité avec les membres du ménage (à côté). |
| Vomissements/Nausée | Sensation de malaise dans l'estomac avec tendance à vomir ; fait d'éjecter une partie/tout le contenu de l'estomac principalement par la bouche. |
| **W/X/Y/Z** | |

## Formulaires de l'étude

Site ___ ___ ___ Sous-zone ___ ___ ___ Numéro d'habitation ___ ___ ___ ___ Identifiant de l'enquêteur ___ __

### FORMULAIRE 1 – COMPTE RENDU DES VISITES AUX MÉNAGES

**Instructions pour l'enquêteur**

- Compléter « Formulaire 1 – COMPTE RENDU DES VISITES AUX MÉNAGES » pour chaque ménage visité.
- Compléter « Foyer/Personne interrogée/Complété » une fois que toutes les parties de l'enquête ont été complétées ; si toutes les données n'ont pas été recueillies lors de la visite initiale et/ou d'une visite ultérieure (max. 3 visites), programmer une nouvelle visite.
- <u>Tentative 1:</u> Compléter le statut de la visite au ménage et utilisant les choix proposés ; pour « pas à la maison », « à la maison/ne répond pas » et « à la maison/répond/indisponible », programmer une nouvelle visite ; pour « à la maison/répond/refus » indiquez la raison de refus et visitez un ménage de remplacement au plus proche à droite ou à gauche de ce ménage; pour « inexistant/pas d' habitation », passer ce ménage et visiter un ménage de remplacement au plus proche à droite ou à gauche à la place de ce ménage.
- <u>Tentative 2:</u> Compléter le statut de la visite du ménage et utilisant les choix proposés ; pour « pas à la maison », « à la maison/ne répond pas » et « à la maison/répond/indisponible », programmer une nouvelle visite.
- <u>Tentative 3:</u> Compléter le statut de la visite au ménage et utilisant les choix proposés ; pour « pas à la maison », « à la maison/ne répond pas » et « à la maison/répond/indisponible », passer ce ménage et visiter un ménage de remplacement au plus proche à droite ou à gauche à la place de ce ménage.
- « Site » et « sous-zone » doivent être un numéro à trois chiffres, « numéro d'habitation » un numéro à quatre chiffres, et « identifiant de l'enquêteur » un numéro à deux chiffres (utiliser les listes préparées individuellement pour chaque site pour obtenir les codes) ; saisir « date » au format JJ/MM/AAAA et « heure » au format HH:MM et entourer MATIN ou APRÈS-MIDI. «Étiquette de l'étude» doit être une combinaison d'un code «site» à trois chiffres, d'un code «sous-zone» à trois chiffres et d'un code «numéro d'habitation» à quatre chiffres; le « identifiant de l'enquêteur » doit être un numéro à trois chiffres.

| **Tentative 1** | Date ____/____/____ (JJ/MM/AA) | Heure _____/_____ MATIN/APRÈS-MIDI | Identifiant de l'enquêteur ___ ___ |
|---|---|---|---|
| STATUT | | Cocher un | |
| 1=Pas à la maison | | O | ➡ Planifier nouvelle visite (date/heure) |
| 2=À la maison/ Non-répondant | | O | ➡ Planifier nouvelle visite (date/heure) |
| 3=À la maison/ répondant /Indisponible | | O | ➡ Planifier nouvelle visite (date/heure) |
| 4= À la maison/ Répondant /Complété | | O | |
| 5= À la maison/ répondant /Refus | | O | Notez la/les raison(s) du refus; cochez tout ce que s'applique:<br>O 1=Pas de temps pour répondre aux questions<br>O 2=Ne veulent pas participer/répondre aux questions<br><br>O 3=Autre ____________________________<br>O 99=Pas de réponse O 98=Ne sait pas<br><br>➡ Visitez un ménage de remplacement le plus proche de ce ménage du côté droit ou gauche |

| | | |
|---|---|---|
| 6=Inexistant/Pas une habitation | O | ➡ Visitez un ménage de remplacement le plus proche de ce ménage du côté droit ou gauche |
| **Tentative 2** Date ____/____/____ (JJ/MM/AA) | Heure _____/_____ MATIN/APRÈS-MIDI | Identifiant de l'enquêteur ___ ___ |
| STATUT | Cocher un | |
| 1=Pas à la maison | O | ➡ Planifier nouvelle visite (date/heure) |
| 2=À la maison/Ne répond pas | O | ➡ Planifier nouvelle visite (date/heure) |
| 3=À la maison/Répond/Indisponible | O | ➡ Planifier nouvelle visite (date/heure) |
| 4= À la maison/Répond/Complété | O | |
| 5= À la maison/ répondant /Refus | O | Notez la/les raison(s) du refus; cochez tout ce que s'applique:<br>O 1=Pas de temps pour répondre aux questions<br>O 2=Ne veulent pas participer/répondre aux questions<br><br>O 3=Autre ____________________________<br>O 99=Pas de réponse O 98=Ne sait pas<br><br>➡ Visitez un ménage de remplacement le plus proche de ce ménage du côté droit ou gauche |
| 6=Inexistant/Pas une habitation | O | ➡ Visitez un ménage de remplacement le plus proche de ce ménage du côté droit ou gauche |
| **Tentative 3** Date ____/____/____ (JJ/MM/AA) | Heure _____/_____ MATIN/APRÈS-MIDI | Identifiant de l'enquêteur ___ ___ |
| STATUT | Cocher un | |
| 1=Pas à la maison | O | ➡ Visitez un ménage de remplacement le plus proche de ce ménage du côté droit ou gauche |
| 2=À la maison/Ne répond pas | O | ➡ Visitez un ménage de remplacement le plus proche de ce ménage du côté droit ou gauche |
| 3=À la maison/Répond/Indisponible | O | ➡ Visitez un ménage de remplacement le plus proche de ce ménage du côté droit ou gauche |
| 4= À la maison/Répond/Complété | O | |
| 5= À la maison/ répondant /Refus | O | Notez la/les raison(s) du refus; cochez tout ce que s'applique:<br>O 1=Pas de temps pour répondre aux questions<br>O 2=Ne veulent pas participer/répondre aux questions<br><br>O 3=Autre ____________________________<br>O 99=Pas de réponse O 98=Ne sait pas |

| | | |
|---|---|---|
| | | ➡ Visitez un ménage de remplacement le plus proche de ce ménage du côté droit ou gauche |
| 6=Inexistant/Pas une habitation | ○ | ➡ Visitez un ménage de remplacement le plus proche de ce ménage du côté droit ou gauche |

Site ___ ___ ___ Sous-zone ___ ___ ___ Numéro d'habitation ___ ___ ___ ___ Identifiant de l'enquêteur ___ __

**FORMULAIRE 2 – FORMULAIRE DE CONSENTEMENT**

Evaluation des soins de santé à [site SETA and –pays]

**Instructions à l'enquêteur**

- Lire le "Formulaire 2 – FORMULAIRE DE CONSENTEMENT" pour le répondant/demander au répondant de lire attentivement le "Formulaire 2 – FORMULAIRE DE CONSENTEMENT" s'il préfère.
- Le répondant tel que défini dans cette enquête est un adulte (ayant l'âge de la majorité spécifique au pays) membre du ménage et responsable des décisions journalières concernant la santé des membres du ménage; il ne doit pas par exemple être un voisin ou un visiteur.
- S'assurer que le répondant a pleinement compris l'objectif, les procédures, les risques et les bénéfices de cette étude avant d'obtenir son consentement à participer.
- Le répondant doit signer le "Formulaire 2 – FORMULAIRE DE CONSENTEMENT"; si le répondant est incapable de signer/analphabète, une empreinte digitale servira en lieu et place de la signature, et un adulte lettré indépendant de l'étude qui servira de témoin (un membre du même ménage ou un voisin par exemple) signera le "Formulaire 2 – FORMULAIRE DE CONSENTEMENT".
- Collecter les informations sur tous les membres du ménage indépendamment du fait qu'ils soient présents ou non au moment de l'entretien; Recueillir l'information sur seulement les individus présents au moment de l'entretien résultera en un biais dans les données.
- Remplir le "Formulaire 2 – FORMULAIRE DE CONSENTEMENT" pour chaque ménage consentant enrôlé dans l'étude.
- Expliquer au répondant qu'il est prévu de revisiter chaque ménage durant une période de 2 ans; a chaque visite, l'équipe de recherche obtiendra un nouveau consentement.
- «Site» et «sous-zone» doivent être un numéro à trois chiffres, «numéro d'habitation» un numéro à quatre chiffres, et «identifiant de l'enquêteur» un numéro à deux chiffres (utiliser les listes préparées individuellement pour chaque site pour obtenir les codes); saisir «date» au format JJ/MM/AAAA et «heure» au format HH:MM et entourer MATIN ou APRÈS-MIDI. «Étiquette de l'étude» doit être une combinaison d'un code «site» à trois chiffres, d'un code «sous-zone» à trois chiffres et d'un code «numéro d'habitation» à quatre chiffres; le «identifiant de l'enquêteur» doit être un numéro à trois chiffres.

**Investigateur principal et co-investigateur de l'étude**

[Ajouter les noms de l'investigateur principal et des co-investigateurs de l'étude ainsi que leurs affiliations respectives]

**Introduction**

L'Institut International des Vaccins (IVI) et [ajouter l'institution d'affiliation des investigateurs locaux] travaillent ensemble pour examiner les problèmes de santé et le comportement de santé dans votre communauté [ajouter le site SETA-pays], y compris le statu socio-économique, le comportement d'hygiène et d'assainissement et le statut vaccinal des enfants. Nous aimerions mieux comprendre où vous, votre famille et vos voisins cherchez des soins de santé en cas de maladies causant la fièvre/corps chaud et autres chante & symptômes. Cette information nous aidera à savoir à quelle fréquence vous souffrez de maladies causant de la fièvre et les autres liées à la fièvre, fournir une meilleure image des facteurs influençant la transmission de la maladie et l'infection, et à trouver des moyens pour les prévenir et traiter dans votre communauté. Vous, en tant que responsable principal pour le comportement de santé/l'utilisation des soins de santé quotidienne pour les membres de ce ménage, êtes invités à participer parce que vous vivez dans la localité où nous effectuons cette étude. Ce formulaire de consentement sera lu par vous ou pour vous et vous aurez le temps pour prendre votre décision d'accepter ou de refuser de participer à l'étude. S'il vous plaît, demandez aux membres de l'étude d'expliquer tout mot ou toute information que vous ne comprenez pas. On vous donnera en conséquence plus d'informations sur cette étude, y compris ses risques potentiels, les avantages et les inconvénients. Après vous avoir entièrement expliqué l'étude et si vous acceptez de participer, on vous demandera de signer ce formulaire de consentement au nom de tous les membres du ménage. Si vous ne pouvez pas signer le formulaire, nous vous demandons de fournir votre empreinte digitale, et une personne adulte indépendante de l'étude (membre de votre ménage/voisin) signera le formulaire en tant que témoin. Nous allons vous remettre une copie signée et datée du formulaire de consentement.

**Objectif**

L'objectif de cette étude est d'examiner les problèmes de santé et l'utilisation des soins de santé dans votre communauté [ajouter le site SETA-pays], y compris le statu socio-économique et le comportement d'hygiène et d'assainissement. Notre objectif est de mieux comprendre où vous, votre famille et vos voisins cherchez les soins de santé pour des maladies causant de la fièvre/corps chaud et autres chante & symptômes. Cette information

permettra de mieux estimer le fardeau des maladies communes et d'identifier les moyens pour la mise en place de mesures de prévention et de traitement pour les maladies.

**Procédures**
Nous visiterons environ [ajouter le nombre pour site d'étude et pays] ménages au total à [ajouter le site et pays] et poserons les mêmes questions dans chaque ménage. Votre ménage a été choisi pour cette étude simplement par hasard. Si vous acceptez de participer, nous vous poserons, au nom de tous les membres du ménage, quelques questions au sujet de votre ménage tel que les initiales, le sexe et l'âge de chaque membre. Nous aimerions savoir certaines informations relatives à l'éducation et l'occupation du chef de ménage, au revenu, à la possession de biens, au logement lui-même ainsi qu'à l'hygiène et à l'assainissement dans le contexte de l'hygiène personnelle, de la préparation et de la manipulation des aliments, des sources d'eau et de la manipulation, de l'élimination des déchets, de la manipulation des animaux de ferme et domestiques ainsi que de l'élevage de ce ménage. Nous allons également demander où les membres de votre ménage obtiennent les soins si quelqu'un a une maladie causant de la fièvre ou toute autre maladie et les facteurs qui peuvent influer sur l'utilisation des soins de santé des membres du ménage (c'est-à-dire les modalités de transport, la disponibilité d'assurance maladie, les facteurs culturels) en plus du statut vaccinal et de la perception de certaines maladies. Enfin, nous allons mesurer la position géographique de votre maison. Il n'y a pas de bonnes ou de mauvaises réponses. Vous êtes libre de choisir les questions à répondre. Somme toute, il devrait prendre environ 30-45 minutes pour répondre à toutes les questions. L'entretien peut prendre un peu plus de temps s'il y a beaucoup de membres dans ce ménage. En outre, nous visiterons votre ménage deux fois en différentes saisons (une fois pendant la saison sèche et une fois vers la fin de la saison des pluies) pendant la période totale de l'étude qui est de deux ans environ pour recueillir des données comme décrit dans ce paragraphe du formulaire de consentement.

**Participation**
Vous, le décideur principal pour le comportement de santé quotidienne et l'utilisation des soins de santé dans ce ménage, ainsi que les membres de votre ménage, êtes libres de choisir d'accepter ou de refuser de participer à l'étude. Il n'y a aucun problème si vous ne voulez pas participer. Il n'y a non plus aucun problème si vous voulez arrêter votre participation à tout moment, et cela ne causera aucun tort à vous ou aux membres de votre ménage. Donc, la participation à cette étude est volontaire, et vous êtes libre de refuser de participer à l'étude ou vous pouvez retirer votre consentement à tout moment sans donner de raisons et ceci n'entraînera aucune pénalité.

**Risques et avantages**
La participation à cette étude n'entraine pas de risque direct. Poser des questions au sujet de votre ménage peut mettre mal à l'aise vous ou les membres de votre ménage. Après avoir terminé les questions, l'équipe de recherche pourra fournir des informations sur les soins de santé communautaires pour les participants qui expriment de la détresse. Vous pouvez refuser de répondre à toute question à tout moment. Vous pouvez faire une pause ou arrêter de participer à cette étude à tout moment. Il n'y a pas d'avantages directs à être dans cette étude. Au cours des visites de votre ménage, notre équipe de recherche peut identifier les membres de votre ménage qui devraient chercher des soins de santé pour les maladies causant de la fièvre et autres. Par conséquent, elle peut aider à fournir de l'information sur l'offre de soins établie dans votre communauté. Les avantages indirects sont que toutes les informations collectées contribueront à fournir des données exactes sur les problèmes de santé, y compris le statu socio-économique, le comportement d'hygiène et d'assainissement et le statut vaccinal des enfants dans votre communauté. Cela aidera à donner une meilleure compréhension des facteurs influençant la transmission de la maladie et l'infection, et à estimer le fardeau des maladies courantes ainsi qu'à trouver des moyens de prévenir et de traiter les personnes dans votre communauté.

**Coûts et Compensations**
Il n'a pas de coûts pour les participants de cette étude. Ni vous ni aucune personne de votre ménage ne sera indemnisée d'avoir participé.

**Confidentialité**
Tous les fichiers de l'étude et de toutes les informations qui identifient vous et les membres de votre ménage seront gardées confidentielles. Tous les questionnaires sur papier seront gardés en toute sécurité et les fichiers informatisés seront protégés par un mot de passe; tous les fichiers de données (sur papier/informatisés) seront stockées pour un minimum de cinq ans; ces fichiers seront accessibles seulement à quelques personnes autorisées de l'étude. Ni votre nom ni aucun identifiant ne sera utilisé dans les publications ou les rapports de cette étude. Les informations que nous collectons à votre sujet et au sujet des membres de votre ménage seront

partagées uniquement avec le personnel de l'étude autorisé et des représentants du comité d'éthique.

**Personnes de contact pour des questions**
Si vous ou des membres de votre ménage avez des questions, s'il vous plaît, demandez maintenant à l'équipe de recherche ou à tout moment plus tard en contactant [ajouter le nom de l'investigateur principal et/ou du co-investigateur, leurs affiliations respectives et les numéros de téléphone]. Si vous avez des questions sur vos droits ou ceux des membres du ménage en tant que participant à l'étude, vous pouvez contacter [ajouter le nom et les coordonnées du comité d'éthique local].

**Déclaration de consentement**
Ce formulaire de consentement pour participer à l'évaluation des soins de santé et d'autres facteurs liés à la population à [ajouter le site et pays] a été bien lu par moi/pour moi. L'objectif, les procédures, les risques et les avantages ont m'ont été expliqués en détails. J'ai été autorisé à poser des questions à tout moment, et mes questions ont été répondues à ma satisfaction par l'équipe de recherche. On m'a dit la personne à contacter en cas de questions ou si je veux discuter de problèmes ou préoccupations. On m'a dit que je recevrai une copie signée et datée de ce formulaire de consentement. Je suis rassuré que toutes les informations obtenues par suite de cette étude seront gardées confidentielles et utilisées uniquement aux fins de cette étude, et seulement par les institutions participantes.

Moi, le répondant, consens, par la présente, volontairement à participer à cette étude. Je suivrai les instructions de l'équipe de recherche et donnerai mon entière collaboration. Je comprends que j'ai le droit de me retirer de l'étude à tout moment.

**RÉPONDANT**

Nom : ______________________________
(prénom + deuxième prénom (si disponible) + nom de famille)

Signature : ______________________________
(Représentation manuscrite du nom/ marque que la personne écrit sur un document comme une preuve d'identité)

Date ______/______/_______________ (YY/MM/AAAA)

Empreinte du pouce

Si le répondant ne peut pas signer/est analphabète, mais accepte de participer, enregistre l'empreinte du pouce dans la boîte ci-dessus, et demande à un témoin adulte, alphabétisé et indépendant de l'étude (par exemple membre du même ménage ou un voisin), de signer ci-dessous :

**TÉMOIN**

Nom : ______________________________
(prénom + deuxième prénom (si disponible) + nom de famille)

Signature : ______________________________
(Représentation manuscrite du nom/ marque que la personne écrit sur un document comme une preuve d'identité)

Date ______/______/_______________ (YY/MM/AAAA)

Moi, l'enquêteur, j'ai lu/expliqué l'étude au répondant nommé ci-dessus (témoin si le répondant est analphabète) dans une langue que le répondant comprend bien. Je suis certain que le répondant a compris l'information et accepte qu'on lui pose des questions.

**INTERVIEWER**

Nom : ______________________________
(prénom + deuxième prénom (si disponible) + nom de famille)

Signature : ______________________________
(Représentation manuscrite du nom/ marque que la personne écrit sur un document comme une preuve d'identité)

Date ______/______/_______________ (YY/MM/AAAA)

## FORMULAIRE 3: INFORMATIONS GÉNÉRALES SUR LE MÉNAGE

**Instructions pour l'enquêteur**

- Compléter ce formulaire uniquement si la personne interrogée a consenti à participer (voir « Formulaire 2 — FORMULAIRE DE CONSENTEMENT ») ; la personne interrogée ainsi que définie pour cette enquête est un adulte (selon l'âge légal de majorité du pays), un membre du ménage et un décideur en ce qui concerne les soins de santé quotidiens et l'utilisation de soins de santé pour le ménage entier et ses membre ; il ne doit pas être, par exemple, un voisin ou un visiteur.
- Recueillir les données de tous les membres du ménage que tous les membres du ménage soient présents ou non au moment de l'entretien ; ne recueillir et n'inscrire les données que des personnes présentes au moment de l'entretien fausserait les données.
- Un ménage est défini/les membres d'un ménage sont définis comme une personne ou un groupe de personnes avec ou sans lien de parenté qui vivent ensemble dans la même unité d'habitation, qui reconnaissent un adulte de sexe masculin ou féminin comme chef de ménage, qui partagent les mêmes installations domestiques, qui sont considérés comme constituant une unité, et qui subviennent à leurs besoins en termes de nourriture et des autres éléments essentiels pour vivre.
- « Site » et « sous-zone » doivent être un numéro à trois chiffres, « numéro d'habitation » un numéro à quatre chiffres, et « identifiant de l'enquêteur » un numéro à deux chiffres (utiliser les listes préparées individuellement pour chaque site pour obtenir les codes) ; saisir « date » au format JJ/MM/AAAA et « heure » au format HH:MM et entourer MATIN ou APRÈS-MIDI. «Étiquette de l'étude» doit être une combinaison d'un code «site» à trois chiffres, d'un code «sous-zone» à trois chiffres et d'un code «numéro d'habitation» à quatre chiffres; le « identifiant de l'enquêteur » doit être un numéro à trois chiffres.

## FORMULAIRE 3: INFORMATIONS GÉNÉRALES SUR LE MÉNAGE
## PARTIE A – DONNÉES DÉMOGRAPHIQUES/ INFORMATIONS GÉNÉRALES SUR LE MÉNAGE

### EMPLACEMENT DU DOMICILE

1 **Numéros de site, de sous-zone et d'habitation**

*Instructions :* Site (=site d'étude) et sous-zone (=plus petite unité du site d'étude) doivent être tous les deux des numéros à trois chiffres, et numéro d'habitation doit être un code à quatre chiffres ; utiliser les listes préparées individuellement pour obtenir les codes respectifs.

Site: ____ ____ ____ Sous-zone: ____ ____ ____ Numéro d'habitation: ____ ____ ____ ____

2 **Coordonnées GPS**

*Instructions :* Prendre les coordonnées GPS approximativement au centre de la maison. Inscrire l'altitude par un nombre à quatre chiffres (unité : mètre), et la latitude/longitude par un numéro à huit chiffres (unités : degrés, minutes décimales).

O 1=Ménage d'origine O 2=Ménage de remplacement (côte droit/gauche du ménage d'origine)

Altitude: ___ ___ ___ ___

Latitude: ___ ___°___ ___.___ ___ ___' Longitude: ___ ___°___ ___.___ ___ ___'

### PERSONNE INTERROGÉE

3 **Inscrire le nom complet de la personne interrogée.**

*Instructions :* Inscrire le prénom, le deuxième nom (le cas échéant) et le nom de famille de la personne interrogée.

________________________________________________

4 **Inscrire l'âge de la personne interrogée.**

*Instruction :* Utiliser un numéro à trois chiffres (unité: année). _____ _____ _____

5 **Inscrire le sexe de la personne interrogée.**

O 1=Masculin O 2=Féminin

6 **Quel est le lien de la personne interrogée avec le ménage ?**

O 1=Chef de ménage
O 7=Conjoint du chef de ménage
O 2=Fils/fille du chef de ménage
O 8=Conjoint du fils/fille du chef de ménage

| | |
|---|---|
| O 3=Frère/sœur du chef de ménage | O 9=Conjoint du frère/sœur du chef de ménage |
| O 4=Mère/père du chef de ménage | O 10=Belle-mère/beau-père du chef de ménage |
| O 5=Petite-fille/petit-fils du chef de ménage | O 11=Nièce/neveu du chef de ménage |
| O 6=Autre, préciser _________ | O 98=Pas de réponse |

## MEMBRES DU MÉNAGE

**7 Combien de membres du ménage vivent dans ce domicile au total** (au moment de la visite) **?**

*Instruction :* Utiliser un numéro à deux chiffres.

Nombre total de membres du ménage : ____ ____

**8 Inscrire l'âge et le sexe de chaque membre du ménage.**

*Instructions :* Inscrire les initiales, l'âge et le sexe de la personne interrogée en première position/ligne du tableau. Les initiales, l'âge et le sexe de tous les autres membres du ménage doivent être inscrits ensuite. Les initiales d'un membre du ménage consistent en les trois premières lettres du prénom et les trois premières lettres du nom de famille (un éventuel deuxième nom ne fait pas partie des initiales). Inscrire l'âge de chaque membre du ménage avec un numéro à trois chiffres (unité : années) ; si l'âge d'un des membres du ménage ≤ 12 mois, inscrire 1 an comme âge. Merci de compléter un formulaire supplémentaire si le ménage a plus de vingt membres.

| **Membre du ménage Identifiant** | **Initiales** [prénom/nom de famille] | **Âge** [années] | **Sexe** |
|---|---|---|---|
| 1/Personne interrogée | ____ ____ ____/____ ____ ____ | ____ ____ ____ | O1=Masculin O2=Féminin |
| 2 | ____ ____ ____/____ ____ ____ | ____ ____ ____ | O1=Masculin O2=Féminin |
| 3 | ____ ____ ____/____ ____ ____ | ____ ____ ____ | O1=Masculin O2=Féminin |
| 4 | ____ ____ ____/____ ____ ____ | ____ ____ ____ | O1=Masculin O2=Féminin |
| 5 | ____ ____ ____/____ ____ ____ | ____ ____ ____ | O1=Masculin O2=Féminin |
| 6 | ____ ____ ____/____ ____ ____ | ____ ____ ____ | O1=Masculin O2=Féminin |
| 7 | ____ ____ ____/____ ____ ____ | ____ ____ ____ | O1=Masculin O2=Féminin |
| 8 | ____ ____ ____/____ ____ ____ | ____ ____ ____ | O1=Masculin O2=Féminin |
| 9 | ____ ____ ____/____ ____ ____ | ____ ____ ____ | O1=Masculin O2=Féminin |
| 10 | ____ ____ ____/____ ____ ____ | ____ ____ ____ | O1=Masculin O2=Féminin |
| 11 | ____ ____ ____/____ ____ ____ | ____ ____ ____ | O1=Masculin O2=Féminin |
| 12 | ____ ____ ____/____ ____ ____ | ____ ____ ____ | O1=Masculin O2=Féminin |
| 13 | ____ ____ ____/____ ____ ____ | ____ ____ ____ | O1=Masculin O2=Féminin |
| 14 | ____ ____ ____/____ ____ ____ | ____ ____ ____ | O1=Masculin O2=Féminin |
| 15 | ____ ____ ____/____ ____ ____ | ____ ____ ____ | O1=Masculin O2=Féminin |
| 16 | ____ ____ ____/____ ____ ____ | ____ ____ ____ | O1=Masculin O2=Féminin |
| 17 | ____ ____ ____/____ ____ ____ | ____ ____ ____ | O1=Masculin O2=Féminin |
| 18 | ____ ____ ____/____ ____ ____ | ____ ____ ____ | O1=Masculin O2=Féminin |
| 19 | ____ ____ ____/____ ____ ____ | ____ ____ ____ | O1=Masculin O2=Féminin |
| 20 | ____ ____ ____/____ ____ ____ | ____ ____ ____ | O1=Masculin O2=Féminin |

## FORMULAIRE 3: INFORMATIONS GÉNÉRALES SUR LE MÉNAGE
## PARTIE B – DONNÉÉS SOCIO-ECONOMIQUES

### ÉDUCATION

**1 Le chef de ménage sait-il lire en** [inscrire la langue officielle du site d'étude/pays] **?**

O 1=Oui O 2=Non O 99=Ne sait pas O 98=Pas de réponse

**2 Le chef de ménage sait-il écrire en** [inscrire la langue officielle du site d'étude/pays] **?**

O 1=Oui O 2=Non O 99=Ne sait pas O 98=Pas de réponse

**3 Quel est le niveau d'éducation scolaire le plus élevé atteint par le chef de ménage ?**

O 1=École primaire O 4=Plus élevé que le lycée O 99=Ne sait pas
O 2=Collège O 5= PAS D'ÉDUCATION O 98=Pas de réponse
O 3=Lycée O 6=Autre, préciser ___________

### PROFESSION/RESSOURCES FINANCIÈRES

**4 Quelle est la profession principale du chef de ménage ?**

*Instructions :* Inscrire le statut du jour de visite uniquement.

O 1=Groupe 1 : Cadres
(directeurs généraux, hauts fonctionnaires, législateurs ; cadres administratifs et commerciaux ; responsables de production et de services spécialisés ; responsables dans l'hôtellerie, la vente et d'autres services)

O 2=Groupe 2 : Professionnels
(scientifiques ou ingénieurs ; professionnels de santé ; professionnels de l'enseignement ; professionnels de l'administration et des affaires ; professionnels de l'information et des technologies de communication ; professionnels du droit, du social ou de la culture)

O 3=Groupe 3 : Techniciens et professions intermédiaires
(Professions intermédiaires des sciences et techniques ; professions intermédiaires de la santé ; professions intermédiaires de l'administration et des affaires ; professions intermédiaires du droit, du social, de la culture et assimilés ; techniciens de l'information et des communications)

O 4=Groupe 4 : Employés de bureau
(secrétaires et dactylographes ; employé de service clientèle ; employé de conservation des données et de la documentation ; autres employés de bureau)

O 5=Groupe 5 : Travailleurs des services et de la vente
(travailleurs du service aux personnes ; travailleurs de la vente ; travailleurs des soins aux personnes ; travailleurs des services de protection)

O 6=Groupe 6 : Travailleurs qualifiés de l'agriculture, la sylviculture et la pêche
(travailleurs qualifiés de l'agriculture orientés marché ; éleveurs d'animaux; travailleurs qualifiés de la sylviculture, la pêche et la chasse orientés marché ; agriculteurs, pêcheurs, chasseurs et cueilleurs de subsistance)

O 7=Groupe 7 : Travailleurs manuels et assimilés
(travailleurs de la construction et assimilés (à l'exception des électriciens) ; travailleurs de la métallurgie, sur machines et assimilés ; artisans et travailleurs de l'imprimerie ; travailleurs des métiers de l'électrique et de l'électronique ; travailleurs des métiers de la transformation des aliments, du bois, des vêtements et assimilés)

O 8=Groupe 8 : Opérateurs de machines et d'installations, et monteurs
(Opérateurs de machines et d'installations fixes ; monteurs ; conducteurs opérateurs d'installations mobiles)

O 9=Groupe 9 : Emplois non qualifiés
(agents d'entretien et assistants ; ouvriers de l'agricultures, la sylviculture et la pêche ; travailleurs des mines, de la construction, de l'industrie et des transports ; assistants à la préparation des aliments ; vendeurs et services de rue et assimilés ; travailleurs de la collecte des ordures et autres travailleurs non qualifiés)

O 10=Groupe 0 : Professions des forces armées
(officiers des forces armées ; sous-officiers des forces armées ; professions des forces armées, autres grades)

O 11=Autre, préciser _________
O 12=PAS DE PROFESSION O 99=Ne sait pas O 98=Pas de réponse

**5 Quel est le montant moyen des revenus mensuels du chef de ménage en lien avec à la profession indiquée en question 4 ainsi que celui des autres membres du ménage ?**

*Instruction:* Tick one answer per household head/enter one code per household member if applicable/if income available.

| Chef de ménage | Membre 1 | Membre 2 | Membre 3 |
|---|---|---|---|
| Code ____________ | Code ____________ | Code ____________ | Code ____________ |

[1] <100USD [3] ≥150 à <200USD [5] ≥250USD [99] Ne sait pas
[2] ≥100 à <150USD [4] ≥200 à <250USD [6] PAS DE REVENU (chef de ménage; voir Q4) [98] Pas de réponse

**6 Quels sont les autres sources ou les sources supplémentaires de revenus mensuels du ménage et quel en est le montant ?**

*Instruction :* Cocher plusieurs cases si nécessaire ; il peut y avoir plus d'une réponse.

| Autres sources/Sources supplémentaires | Revenus financiers mensuels globaux |
|---|---|
| O 1=Aides sociales<br>O 2=Assurances (publiques ou privées)<br>O 3=Prêt (d'amis, de membres de la famille, d'une banque)<br>O 4=Vente (bétail, récoltes, meubles, autres biens)<br>O 5=Économies personnelles<br>O 6=Autre, préciser _______________ | O 1=<100 USD<br>O 2=≥100 à <150 USD<br>O 3=≥150 à <200 USD<br>O 4=≥200 à <250 USD<br>O 5=≥250 USD |

O 7=PAS D'AUTRES SOURCES/DE SOURCES SUPPLÉMENTAIRES
O 99=Ne sait pas
O 98=Pas de réponse

## LOGEMENT/HABITATION

**7 Les membres du ménage sont-ils propriétaires de l'habitation ?**

O 1=Oui O 3=Non, logement fourni (par le gouvernement, un employeur) O 99=Ne sait pas
O 2=Non, locataires O 4=Non, autres, préciser _________ O 98=Pas de réponse

**8 Combien de pièces ou structures individuelles de l'habitation sont utilisées pour dormir, vivre, faire la cuisine ou se laver ?**

*Instruction :* N'indiquer qu'un code par catégorie.

| Chambre | Salon | Cuisine | Salle de bain |
|---|---|---|---|
| Code ____________ | Code ____________ | Code ____________ | Code ____________ |

[1] 1-2 [2] >4 [99] Ne sait pas
[2] 3-4 [2] Aucune [98] Pas de réponse

**9 En quel matériau sont construits le sol, les murs et le toit des pièces/des structures individuelles de l'habitation ?**

*Instruction :* Indiquer tous les codes par catégorie ; plus d'une réponse par catégorie est possible.

| Sol | Murs | Toit |
|---|---|---|
| Code ____________ | Code ____________ | Code ____________ |

[1] Brique [3] Tuiles [5] Bois [7] Métal/Étain [99] Ne sait pas
[2] Ciment/Béton [4] Terre [6] Paille/feuilles [8] Autre, préciser_________ [98] Pas de réponse

**10 Les membres de ce ménage ont-ils accès à/utilisent-ils l'électricité dans cette habitation ?**

O 1=Oui O 2=Non O 99=Ne sait pas O 98=Pas de réponse

**11 À quelle source de lumière principale les membres de ce ménage ont-ils accès/Quelle source de lumière principale utilisent-ils ?**

O 1=Lumière/ampoule(s) électrique(s) O 4=Lampe(s) à piles O 7= Autre, préciser_____
O 2=Lumière du soleil/Lampe(s) solaire(s) O 5=Bougie(s) O 99=Ne sait pas
O 3=Lampe(s) à pétrole/à huile O 6=PAS DE SOURCE DE LUMIÈRE O 98=Pas de réponse

## BIEN DU MÉNAGE

**12 Parmi les articles suivants, lesquels les membres du ménage possèdent-ils ?**

*Instructions :* Cocher plusieurs cases si nécessaire ; il peut y avoir plus d'une réponse ; indiquer la quantité d'articles fonctionnels si nécessaire

| | | | | | |
|---|---|---|---|---|---|
| O 1=Radio | Quantité ____ | O Rien | O 11=Lecteur cassettes/CD | Quantité ____ | O Rien |
| O 2=Télévision | Quantité ____ | O Rien | O 12=Ordinateur de bureau/portable/tablette | Quantité ____ | O Rien |
| O 3=Connexion internet | Quantité ____ | O Rien | O 13=Téléphone portable | Quantité ____ | O Rien |
| O 4=Voiture | Quantité ____ | O Rien | O 14=Camion/bus | Quantité ____ | O Rien |
| O 5=Moto | Quantité ____ | O Rien | O 15=Vélo | Quantité ____ | O Rien |
| O 6=Char à bœufs/ânes | Quantité ____ | O Rien | O 16=Lit | Quantité ____ | O Rien |
| O 7=Matelas | Quantité ____ | O Rien | O 17=Moustiquaire | Quantité ____ | O Rien |
| O 8=Table | Quantité ____ | O Rien | O 18=Chaise | Quantité ____ | O Rien |
| O 9=Réfrigérateur (4°C) | Quantité ____ | O Rien | O 19=Congélateur (-20°C) | Quantité ____ | O Rien |
| O 10=Ventilateur | Quantité ____ | O Rien | O 20=Machine à coudre | Quantité ____ | O Rien |

## FORMULAIRE 3: INFORMATIONS GÉNÉRALES SUR LE MÉNAGE
## Partie C – Hygiène et installations sanitaires

### TOILETTES

**1 À quel type de toilettes les membres du ménage ont-ils habituellement accès ?**

*Instruction :* À l'intérieur signifie à l'intérieur d'une pièce/d'une habitation individuelle/d'un bâtiment ; à l'extérieur signifie à l'extérieur d'une pièce/d'une habitation individuelle/d'un bâtiment.

- O 1=Toilettes avec chasse d'eau/Cabinets (à l'intérieur)
- O 2=Toilettes avec chasse d'eau/Cabinets (à l'extérieur)
- O 3=Latrines sans chasse/latrines à fosse (à l'intérieur)
- O 4=Latrines sans chasse/latrines à fosse (à l'extérieur)
- O 5=Latrines à fosse améliorées par une ventilation (KVIP, à l'intérieur)
- O 6=Latrines à fosse améliorées par une ventilation (KVIP, à l'extérieur)
- O 7=Toilettes à compostage (à l'intérieur)
- O 8=Toilettes à compostage (à l'extérieur)
- O 9=PAS de toilettes, utilisent un seau
- O 10=PAS de toilettes, creusent un trou
- O 11=PAS de toilettes, défécation libre/en plein air
- O 12=Autre, préciser _________
- O 99=Ne sait pas
- O 98=Pas de réponse

**2 Les membres du ménage utilisent-ils habituellement les toilettes indiquées à la question 1 ?**

- O 1=Non, les membres ne peuvent utiliser les toilettes de façon autonome (bébés, enfants en bas-âge)
- O 2=Non, les membres ne peuvent utiliser les toilettes de façon autonome (handicapés, malades chroniques/longue durée)
- O 3=Non, les membres ne peuvent utiliser les toilettes de façon autonome (personnes âgées)
- O 4=Non, accès limité (publiques, partagées avec d'autres ménages/communautés)
- O 5=Non, accès limité (publiques, partagées avec d'autres ménages/communautés)
- O 6= Non, mauvais état (cassé, pas propre)
- O 7=OUI
- O 8=Autre, préciser ________________
- O 99=Ne sait pas
- O 98=Pas de réponse

**3 Où sont situées les toilettes indiquées à la question 1 ? Par combien de personnes sont-elles habituellement partagées ?**

- O 1=Sur la propriété du ménage (privées, non partagées)
- O 2=Sur la propriété d'un ménage voisin (privées, partagées)
  - *Partagées par* O 1=<10 personnes O 2=11-24 personnes O 3=25-49 personnes O 4=>50 personnes O 99=Ne sait pas O 98=Pas de réponse
- O 3=Dans la communauté (publiques, partagées)
  - *Partagées par* O 1=<10 personnes O 2=11-24 personnes O 3=25-49 personnes O 4=>50 personnes O 99=Ne sait pas O 98=Pas de réponse
- O 4=Autre, préciser_________
- O 99=Ne sait pas
- O 98=Pas de réponse

**4 Comment les membres du ménage qui ont accès/n'ont pas accès aux toilettes se lavent-ils habituellement les mains tout de suite avant et après avoir uriné/déféqué ?**

*Instruction :* Indiquer un code par catégorie uniquement.

| Lavent les mains avant d'avoir uriné/déféqué | Lavent les mains après avoir uriné/déféqué |
|---|---|
| Code ____________ | Code ____________ |
| [1] Lavent les mains avec du savon et de l'eau<br>[2] Lavent les mains avec de l'eau uniquement<br>[3] Frottent les mains avec des feuilles/de la paille/de l'herbe<br>[4] Frottent les mains avec du sable<br>[5] Frottent les mains avec un chiffon/du tissu | [6] Ne lavent pas, pas besoin/pas sale<br>[7] Ne lavent pas, rien pour laver les mains<br>[8] Autre, préciser _________<br>[99] Ne sait pas<br>[98] Pas de réponse |

### CUISINE/MANIPULATION DES ALIMENTS

**5 Les membres de ce ménage ont-ils une cuisine pour faire cuire/préparer la nourriture/les repas ? Où est-elle située ?**

- O 1=Oui, cuisine intérieure/dans une pièce/structure individuelle d'habitation ; sur la propriété du ménage (privée, non partagée)
- O 2=Oui, cuisine extérieure/hors d'une pièce/structure individuelle d'habitation/ en plein air ; sur la propriété du ménage (privée, non partagée)

○ 3=Oui, cuisine intérieure/dans une pièce/structure individuelle d'habitation ; sur la propriété d'un ménage voisin (privée, partagée)
○ 4=Oui, cuisine extérieure/hors d'une pièce/structure individuelle d'habitation/ en plein air ; sur la propriété d'un ménage voisin (privée, partagée)
○ 5=PAS DE CUISINE
○ 6=Autre, préciser _________ ○ 99=Ne sait pas ○ 98=Pas de réponse

6 **Quelle source d'énergie les membres de ce ménage utilisent-ils habituellement pour cuisiner/préparer la nourriture/les repas ?**

○ 1=Électricité ○ 3=Charbon de bois ○ 5=Excréments d'animaux ○ 99=Ne sait pas
○ 2=Gaz/pétrole ○ 4=Bois/paille/feuilles ○ 6=Autre, préciser _________ ○ 98=Pas de réponse

7 **Quelle moyen de cuisson les membres de ce ménage utilisent-ils habituellement pour cuisiner/préparer la nourriture/les repas ?**

○ 1=Foyer ouvert ○ 3=Fourneau intérieur avec cheminée ○ 99=Ne sait pas
○ 2=Fourneau extérieur ○ 4=Autre, préciser _________ ○ 98=Pas de réponse

8 **Où les membres de ce ménage conservent-ils habituellement les produits alimentaires frais/crus (comme les légumes, la salade, les fruits, les produits laitiers, les œufs, la viande et le poisson) et les restes/les aliments/repas préparés ?**

*Instruction :* Indiquer un code par catégorie uniquement. À l'intérieur signifie à l'intérieur d'une pièce/d'une habitation individuelle/d'un bâtiment ; à l'extérieur signifie à l'extérieur d'une pièce/d'une habitation individuelle/d'un bâtiment.

| Des produits alimentaires frais/crus (légumes, salade, fruits, produits laitiers, œufs, viande et poisson) | Des restes/aliments/repas préparés |
|---|---|
| Code ____________ | Code ____________ |
| [1] À température ambiante (à l'intérieur, sur la propriété du ménage, privé)<br>[2] À température ambiante (à l'extérieur, sur la propriété du ménage, privé)<br>[3] À température ambiante (à l'intérieur, sur la propriété d'un ménage voisin, privé, partagé)<br>[4] À température ambiante (à l'extérieur, sur la propriété d'un ménage voisin, privé, partagé)<br>[5] Réfrigérateur (sur la propriété du ménage, privé)<br>[6] Réfrigérateur (sur la propriété d'un ménage voisin, privé, partagé)<br>[7] Congélateur (sur la propriété du ménage, privé)<br>[8] Congélateur (sur la propriété d'un ménage voisin, privé, partagé)<br>[9] Autre, préciser _____________ [10] PAS DE CONSERVATION [99] Ne sait pas [98] Pas de réponse | |

9 **Si la réponse à la question 8 au sujet des produits alimentaires frais/crus et/ou des restes/aliments/repas préparés est «température ambiante», combien de temps les membres de ce ménage les conservent-ils habituellement?**

*Instruction:* Indiquer un code par catégorie uniquement.

| Des produits alimentaires frais/crus (légumes, salade, fruits, produits laitiers, œufs, viande et poisson) | Des restes/aliments/repas préparés |
|---|---|
| Code ____________ | Code ____________ |
| [1] <1 jour [3] 3-4 jours [5] Pas applicable [99] Ne sait pas<br>[2] 1-2 jours [4] >4 jours [98] Pas de réponse | |

10 **Les membres de ce ménage couvrent-ils habituellement les restes/aliments/repas préparés? Réchauffent-ils/recuisent-ils les restes/aliments/repas préparés avant de les consommer?**

*Instruction :* Indiquer un code par catégorie uniquement.

| Couvrent les restes/les aliments/repas préparés | Réchauffent/Recuisent les restes/les aliments/repas préparés |
|---|---|
| Code ____________ | Code ____________ |
| [1] Oui [2] Non [99] Ne sait pas [98] Pas de réponse | |

**11 Quel membre(s) de ce ménage cuisine(s)/prépare(s) la nourriture/les repas habituellement pour les autres membres de ce ménage ?**

*Instructions :* Se référer à la question 9 du « Formulaire 3 Partie A – Données démographiques/Informations générales sur le ménage » et indiquer les initiales/ Identifiant (s) du membre.

○ Membre du ménage, indiquer les initiales/identificateur(s) _________

○ 99=Ne sait pas ○ 98=Pas de réponse

**12 Comment le(s) membre(s) de la famille qui cuisine(nt)/prépare(nt) la nourriture/les repas habituellement pour les autres membres de ce ménage comme indiqué à la question 11 se prépare(nt)-t-il(s)/elle(s) habituellement avant et après la préparation des repas?**

*Instruction:* Indiquer un code par catégorie uniquement.

| Avant la préparation des repas | Après la préparation des repas |
|---|---|
| Code ____________ | Code ____________ |
| [1] Lavent les mains avec du savon et de l'eau<br>[2] Lavent les mains avec de l'eau uniquement<br>[3] Frottent les mains avec des feuilles/de la paille/de l'herbe<br>[4] Frottent les mains avec du sable<br>[5] Frottent les mains avec un chiffon/du tissu | [6] Ne lavent pas, pas besoin/pas sale<br>[7] Ne lavent pas, rien pour laver les mains<br>[8] Autre, préciser _________<br>[99] Ne sait pas<br>[98] Pas de réponse |

**13 Comment les membres de ce ménage se préparent-ils habituellement avant et après avoir mangé de la nourriture/des repas, y compris le membre du ménage qui cuisine/prépare la nourriture/les repas habituellement comme indiqué à la question 11?**

*Instruction :* Indiquer un code par catégorie uniquement.

| Avant la préparation des repas | Après la préparation des repas |
|---|---|
| Code ____________ | Code ____________ |
| [1] Lavent les mains avec du savon et de l'eau<br>[2] Lavent les mains avec de l'eau uniquement<br>[3] Frottent les mains avec des feuilles/de la paille/de l'herbe<br>[4] Frottent les mains avec du sable<br>[5] Frottent les mains avec un chiffon/du tissu | [6] Ne lavent pas, pas besoin/pas sale<br>[7] Ne lavent pas, rien pour laver les mains<br>[8] Autre, préciser _________<br>[99] Ne sait pas<br>[98] Pas de réponse |

**14 Comment les membres de ce ménage mangent-ils habituellement leur nourriture/leurs repas, y compris le membre du ménage qui cuisine/prépare la nourriture/les repas habituellement comme indiqué à la question 11?**

○ 1=Avec des couverts ○ 3=Autre, préciser _________ ○ 99=Ne sait pas

○ 2=Avec les doints ○ 98=Pas de réponse

**15 Les membres de ce ménage mangent-ils habituellement leur nourriture/leurs repas dans le même récipient, y compris le membre du ménage qui cuisine/prépare la nourriture/les repas habituellement comme indiqué à la question 11?**

○ 1=Oui ○ 2=Non ○ 99=Ne sait pas ○ 98=Pas de réponse

**16 Où les membres de ce ménage obtiennent-ils habituellement leurs produits alimentaires frais/crus principaux comme les légumes, les fruits, les grains/céréales, les produits laitiers, les œufs, la viande et le poisson?**

*Instruction :* Indiquer un code par catégorie uniquement.

| Légumes, fruits, grains/céréales | Produits laitiers | Œufs, viande, poisson |
|---|---|---|
| Code ____________ | Code ____________ | Code ____________ |
| [1] Agriculture vivrière (cour/arrière-cour)<br>[2] Supermarché/Épicerie<br>[3] Amis/famille<br>[4] Marché ouvert<br>[5] Vendeur de rue | | [6] Agriculture vivrière (terre agricole)<br>[7] Boucher<br>[8] Autre, préciser ______<br>[99] Ne sait pas<br>[98] Pas de réponse |

**17 Les membres de ce ménage consomment/mangent-ils des produits alimentaires frais/crus (comme les légumes, la salade, les fruits, les produits laitiers, la glace, les œufs, la viande et le poisson) ? Si oui, lesquels?**

*Instruction :* Cocher plusieurs cases si nécessaire ; plus d'une réponse est possible.

O 1=Légumes/salade (cultivés sur arbuste)
O 2=Légumes/salade (récolte sauvage sur arbuste)
O 3=Fruits (cultivés sur arbuste/arbre)
O 4=Fruits (récolte sauvage sur arbuste/arbre)
O 5=Produits laitiers (lait, fromage, yaourt d'animaux domestiques produisant du lait), préciser ____________
O 6=Œufs (de volailles domestiques), préciser _________
O 7=Sang animal (d'animaux domestiques), domestiques), préciser _________
O 8=Bœuf/Zébu/Buffle (domestiqué), préciser _________
O 9=Porc (domestiqué)
O 10=Mouton (domestiqué)
O 11=Viande d'animaux non-domestiqués (antilope, sanglier, volaille, rongeurs, poisson), préciser ________
O 12=PAS DE CONSOMMATION de produits frais/crus

O 13=Légumes/salade (cultivés au sol)
O 14=Légumes/salade (récolte sauvage sur le sol)
O 15=Fruits (cultivés au sol)
O 16=Fruits (récolte sauvage sur le sol)
O 17=Produits laitiers (lait, fromage, yaourt d'animaux non-domestiques produisant du lait), préciser ____________
O 18= Œufs (de volailles non-domestiques), préciser _________
O 19= Sang animal (d'animaux non- préciser _________
O 20=Volaille (domestiquée; poulet, canard, pigeon, oie, dinde), préciser _________
O 21=Chèvre (domestiquée)
O 22= Rongeurs (domestiqués), préciser ______
O 23=Poisson/fruits de mer, préciser _____
O 24=Glace
O 25=Autre, préciser _________
O 99=Ne sait pas O 98=Pas de réponse

**18 Les membres de ce ménage consomment-ils habituellement des repas/produits alimentaires/boissons également en dehors de l'habitation ? Si oui, quel(s) membre(s) de ce ménage et où ?**

*Instructions :* Se référer à la question 9 du « Formulaire 3 Partie A – Données démographiques/Informations générales sur le ménage » et indiquer les initiales du/des membre(s). Indiquer tous les codes nécessaires pour les membres du ménage respectifs.

| Membre 1<br>Initiales/Identifiant : ___ | Membre 2<br>Initiales/Identifiant : ___ | Membre 3<br>Initiales/Identifiant : ___ | Membre 4<br>Initiales/ Identifiant : ___ |
|---|---|---|---|
| Code ____________ | Code ____________ | Code ____________ | Code ____________ |
| [1] Restaurant<br>[2] Chaîne de restauration rapide | [3] Cafétéria<br>[4] Vendeur de rue | [5] Autre, préciser ____<br>[6] Pas applicable | [99] Ne sait pas<br>[98] Pas de réponse |

**19 Quels/Quelles repas/produits alimentaires/boissons le/les membre(s) de ce ménage listé à la question 18 consomment-ils habituellement en dehors de l'habitation ?**

*Instructions :* Se référer à la question 9 du « Formulaire 3 Partie A – Données démographiques/Informations générales sur le ménage » et indiquer/identifiant les initiales du/des membre(s). Indiquer jusqu'à 5 repas/produits alimentaires/boissons par membre du ménage. Vous pouvez vous référer aux produits alimentaires listés à la question 17. Passer cette question si aucun membre du ménage ne consomme les repas/les produits alimentaires/les boissons en dehord de la maison comme indiqué dans Q18.

| Membre 1<br>Initiales/Identifiant : ____ | Membre 2<br>Initiales/Identifiant : ____ | Membre 3<br>Initiales/Identifiant : ____ | Membre 4<br>Initiales/ Identifiant : ___ |
|---|---|---|---|
| 1: ______________<br>2: ______________<br>3: ______________<br>4: ______________<br>5: ______________ | 1: ______________<br>2: ______________<br>3: ______________<br>4: ______________<br>5: ______________ | 1: ______________<br>2: ______________<br>3: ______________<br>4: ______________<br>5: ______________ | 1: ______________<br>2: ______________<br>3: ______________<br>4: ______________<br>5: ______________ |

## EAU

**20 Quelle est la source d'approvisionnement en eau principale des membres de ce ménage pour la boisson, la cuisine/la préparation des aliments/repas, le bain et le ménage (de l'habitation/de la/les structure(s) individuelle(s) d'habitation(s), les vêtements/les outils de cuisine) ?**

*Instruction :* Indiquer un code par catégorie uniquement.

| Boisson | Cuisine | Bain | Nettoyage |
|---|---|---|---|
| Code ____________ | Code ____________ | Code ____________ | Code ____________ |

[1] Eau du robinet (à l'intérieur de l'habitation ; privée)
[2] Eau du robinet (à l'extérieur de l'habitation : cour/arrière-cour ; privée)
[3] Eau du robinet (à l'extérieur de l'habitation ; publique/partagée)
[4] Borne-fontaine/eau courante (à l'intérieur de l'habitation ; privée)
[5] Borne-fontaine/eau courante (à l'extérieur de l'habitation : cour/arrière-cour ; privée)
[6] Borne-fontaine/eau courante (à l'extérieur de l'habitation ; publique/partagée)
[7] Camion-citerne (public ; partagé)
[8] Eaux de surface (canal, canal d'irrigation)
[9] Eaux de surface (lac, étang, rivière, ruisseau)
[10] Eau de source
[11] Eau de pluie
[12] Puits/Puits de forage (en plein air/non couvert/non protégé)
[13] Puits/Puits de forage (couvert/protégé)
[14] Eau en bouteille
[15] Autre, préciser ___________________ [99] Ne sait pas [98] Pas de réponse

**21 Les membres de ce ménage traitent-ils habituellement l'eau pour la boisson, la cuisine/la préparation des aliments/des repas, le bain, et le nettoyage (de l'habitation/des/de la structure(s) individuelle(s) d'habitation(s), des vêtements/des outils de cuisine) avant la consommation ? Comment l'eau est-elle habituellement traitée ?**

*Instruction:* Indiquer tous les codes nécessaires par catégorie ; plus d'une réponse par catégorie est possible.

| Boisson | Cuisine | Bain | Nettoyage |
|---|---|---|---|
| Code ____________ | Code ____________ | Code ____________ | Code ____________ |

[1] Non, pas du tout
[2] Non, seulement pour les malades du ménage
[3] Non, seulement selon la saison (saison sèche)
[4] Oui, par filtration (tissu, céramique, sable)
[5] Oui, par sédimentation
[6] Autre, préciser ________________
[7] Non, seulement pour les enfants du ménage
[8] Non, seulement selon la saison (saison humide)
[9] Oui, par ébullition
[10] Oui, par désinfection solaire
[11] Oui, par chloration
[99] Ne sait pas [98] Pas de réponse

**22 Quel(s) membre(s) de ce ménage recueille(nt) habituellement l'eau potable comme indiqué à la question 20 pour les autres membres de ce ménage ?**

*Instructions :* Se référer à la question 9 du « Formulaire 3 Partie A – Données démographiques/Informations générales sur le ménage » et indiquer/identifiant les initiales du membre.

○ Membre du ménage, indiquer les initiales/identifiant(s) _________
○ 99=Ne sait pas ○ 98=Pas de réponse

**23 Comment les membres de ce ménage conservent-ils habituellement l'eau potable indiquée à la question 20?**

○ 1=Réservoir d'eau (non couvert)
○ 2=Réservoir d'eau (couvert par chiffon/tissu, couvercle, feuilles)
○ 3=Récipient (non couvert)
○ 4=Récipient (couvert par chiffon/tissu, couvercle, feuilles)
○ 5=Pot en terre (non couvert)
○ 6=Pot en terre (couvert par chiffon/tissu, couvercle, feuilles)
○ 7=Seau (non couvert)
○ 8=Seau (couvert par chiffon/tissu, couvercle, feuilles)
○ 9=Bol (non couvert)
○ 10=Bol (couvert par chiffon/tissu, couvercle, feuilles)
○ 11=Pot en bois (non couvert)
○ 12= Pot en bois (couvert par chiffon/ tissu, couvercle, feuilles)
○ 13= Récipient en peau (chèvre, mouton, bovins, non couvert)
○ 14=Récipient en peau (chèvre, mouton, bovins ; couvert par un chiffon/du tissu, un couvercle, des feuilles
○ 15=NON CONSERVÉE
○ 16=Autre, préciser _________
○ 99=Ne sait pas
○ 98= Pas de réponse

**24 Si les membres de ce ménage utilisent une source d'approvisionnement en eau potable partagée comme indiqué à la question 20, par combien de personnes cette eau potable est-elle partagée ?**

O 1=<10 personnes　O 3=25-49 personnes　O 5=NON PARTAGÉE　O 99=Ne sait pas
O 2=11-24 personnes　O 4=>50 personnes　O 98=Pas de réponse

## TRAITEMENT DES DÉCHETS

**25 Où les membres de ce ménage jettent-ils habituellement les eaux usées de, par exemple, la cuisine/la préparation des aliments/repas, le bain et/ou le ménage, et jettent-ils habituellement les déchets domestiques, les déchets humains/déjections ?**

*Instruction:* Indiquer un code par catégorie uniquement.

| Eaux usées de par exemple cuisine/préparation des aliments/ repas, bain et/ou ménage | Déchets domestiques | Déchets humains/déjections |
|---|---|---|
| Code ____________ | Code ____________ | Code ____________ |

[1] Sur le sol/surface libre proche de l'habitation (cour/arrière-cour, privé)
[2] Sur le sol/surface libre loin de l'habitation dans la communauté (partagé, public)
[3] Dans un trou proche de la maison (cour/arrière-cour, privé)
[4] Dans un trou loin de la maison dans la communauté (partagé, public)
[5] Versement dans des eaux de surface dans la communauté (lac, étang, rivière, ruisseau, public, partagé)
[6] Versement dans des eaux de surface dans la communauté (canal, canal d'irrigation, public, partagé)
[7] Versement dans de l'eau de source dans la communauté (public, partagé)
[8] Versement dans une fosse septique proche de l'habitation (cour/arrière-cour, proche de toilettes si disponibles, privé)
[9] Versement dans une fosse septique dans la communauté (partagé, public)
[10] Versement dans les égouts proches de la maison (cour/arrière-cour, privé)
[11] Versement dans les égouts dans la communauté (partagé, public)
[12] Autre, préciser ___________________________　[99] Ne sait pas　[98] Pas de réponse

**26 Les membres de ce ménage traitent-ils habituellement les déchets domestiques de la maison au préalable avant de les jeter ?**

O 1=Oui, incinération des déchets　O 3=NON
O 2=Oui, séparation/tri des déchet dégradable et non dégradables　O 4=Oui, Autre, préciser _________
O 99=Ne sait pas　O 98=Pas de réponse

**27 À quelle distance de la source d'approvisionnement en eau de ce ménage comme indiqué à la question 20 se trouve l'emplacement de l'élimination des eaux usées, des déchets domestiques et humains/excrétions de ce ménage comme indiqué à la question 25 ?**

*Instructions :* Indiquer un code par catégorie uniquement. Indiquer la distance en mètres [m].

| Eaux usées de, par exemple, cuisine/préparation des aliments/ repas, bain et/ou ménage | Déchets domestiques | Déchets humains/déjections |
|---|---|---|
| Code ____________ | Code ____________ | Code ____________ |

[1] <5 m　[3] ≥25 à <50 m　[5] ≥100 à <500 m　[7] ≥1,000 m
[2] ≥5 à <25 m　[4] ≥50 à <100 m　[6] ≥500 à <1,000 m　[99] Ne sait pas　[98] Pas de réponse

**28 À quelle distance de la source d'approvisionnement en eau principale de ce ménage pour la cuisine/la préparation des aliments/des repas comme indiqué à la question 20 se trouve l'emplacement de l'élimination des eaux usées, des déchets domestiques et humains/excrétions de ce ménage comme indiqué à la question 25 ?**

*Instructions :* Indiquer un code par catégorie uniquement. Indiquer la distance en mètres [m].

| Eaux usées de par exemple cuisine/préparation des aliments/ repas, bain et/ou ménage | Déchets domestiques | Déchets humains/excréments |
|---|---|---|
| Code ____________ | Code ____________ | Code ____________ |

| | | | |
|---|---|---|---|
| [1] <5 m | [3] ≥25 à <50 m | [5] ≥100 à <500 m | [7] ≥1,000 m |
| [2] ≥5 à <25 m | [4] ≥50 à <100 m | [6] ≥500 à <1,000 m | [99] Ne sait pas [98] Pas de réponse |

## ANIMAUX DE FERME/DOMESTIQUES

**29 Quel genre d'animaux/de bétail et d'animaux domestiques/de compagnie les membres de ce ménage possèdent-ils ?**

*Instruction :* Cocher plusieurs cases par catégorie si nécessaire ; il peut y avoir plus d'une réponse par catégorie.

| Animaux de ferme/bétail | | Animaux domestiques/de compagnie |
|---|---|---|
| O 1=Bovin | O9=Chameau | O 1=Chien |
| O 2=Mouton | O 10=Rongeurs (lapin), préciser ______ | O 2=Rongeurs (lapin), préciser ______ |
| O 3=Âne | O 11=Chèvre | O 3=Chat |
| O 4= Volaille (i.e. poulet, canard, pigeon, oie, dinde), préciser ______ | O 12=Cheval | O 4=PAS D'ANIMAUX DOMESTIQUES/DE COMPAGNIE |
| O 5=Zébu | O 13=PAS D'ANIMAUX DE FERME/DE BÉTAIL | O 5=Autre, préciser ______ |
| O 6=Cochon | O 14=Autre, préciser ______ | O 99=Ne sait pas |
| O 7=Poisson | O 99=Ne sait pas | O 98=Pas de réponse |
| O 8=Buffle | O 98=Pas de réponse | |

**30 Où les membres de ce ménage gardent-ils habituellement les animaux de ferme/le bétail et les animaux domestiques/de compagnie de ce ménage comme indiqué à la question 29 ?**

*Instruction :* Indiquer un code par catégorie uniquement.

| Animaux de ferme/bétail | Animaux domestiques/de compagnie |
|---|---|
| O 1=Étable/Cage/Bassin d'eau sur la propriété de l'habitation (arrière-cour/cour) | O 1=Cage/Chaîne proche de l'habitation (arrière-cour/cour) |
| O 2=Étable/Cage/Bassin d'eau à l'extérieur de l'habitation dans la communauté | O 2= Cage/Chaîne à l'extérieur de l'habitation dans la communauté |
| O 3=Étable/Cage/Bassin d'eau à l'extérieur de la communauté | O 3= Cage/Chaîne à l'extérieur de la communauté (public) |
| O 4=Surface libre/Eaux de surface/Eau de source sur la propriété de l'habitation (habitation, arrière-cour/cour) | O 4=Surface libre proche de l'habitation (dans la maison, arrière-cour/cour) |
| O 5=Surface libre/Eaux de surface/Eau de source à l'extérieur de l'habitation dans la communauté | O 5=Surface libre à l'extérieur de l'habitation dans la communauté |
| O 6=Surface libre/Eaux de surface/Eau de source à l'extérieur de la communauté | O 6=Surface libre à l'extérieur de la communauté |
| O 7=Pas applicable (nulle part /pas d'animaux de ferme/bétail) | O 7=Pas applicable (nulle part/pas d'animaux domestiques/de compagnie) |
| O 8=Autre, préciser ______ | O 8=Autre, préciser ______ |
| O 99=Ne sait pas | O 99=Ne sait pas |
| O 98=Pas de réponse | O 98=Pas de réponse |

**31 Quelle est l'importance de l'élevage que les membres de ce ménage pratiquent ?**

O 1=Pour leurs propres besoins («élevage d'arrière-cour»)
O 2=Pas applicable (pas d'élevage)
O 3=3=Demande massive/industrielle («élevage intensif»)
O 4=Autre, préciser ______
O 99=Ne sait pas
O 98=Pas de réponse

**32 Quel approvisionnement en eau les membres de ce ménage utilisent-ils pour fournir de l'eau aux animaux de ferme/au bétail et aux animaux domestiques/de compagnie de ce ménage comme indiqué à la question 29 ?**

*Instruction :* Indiquer un code par catégorie uniquement.

| Animaux de ferme/bétail | Animaux domestiques/de compagnie |
|---|---|
| Code ____________ | Code ____________ |

[1] Eau du robinet (à l'intérieur de l'habitation ; privée)
[2] Eau du robinet (à l'extérieur de l'habitation : cour/arrière-cour ; privée)
[3] Eau du robinet (à l'extérieur de l'habitation ; publique/partagée)
[4] Borne-fontaine/eau courante (à l'intérieur de l'habitation ; privée)
[5] Borne-fontaine/eau courante (à l'extérieur de l'habitation : cour/arrière-cour ; privée)
[6] Borne-fontaine/eau courante (à l'extérieur de l'habitation ; publique/partagée)
[7] Camion-citerne (public ; partagé)
[8] Eaux de surface (canal, canal d'irrigation)
[9] Eaux de surface (lac, étang, rivière, ruisseau)
[10] Eau de source
[11] Eau de pluie
[12] Puits/Puits de forage (en plein air/non couvert/non protégé)
[13] Puits/Puits de forage (couvert/protégé)
[14] Pas applicable (pas d'animaux de ferme/bétail; pas d'animaux domestiques/de compagnie)
[15] Autre, préciser ___________________ [99] Ne sait pas [98] Pas de réponse

**33 Les membres de ce ménage se lavent-ils habituellement les mains après avoir nourri, donné de l'eau et nettoyé l'étable/la cage/le bassin d'eau des animaux de ferme/du bétail de ce ménage comme indiqué à la question 29 ?**

*Instruction :* Indiquer un code par catégorie uniquement.

| Animaux de ferme/bétail | Animaux domestiques/de compagnie |
|---|---|
| Code ____________ | Code ____________ |

[1] Lavent les mains avec du savon et de l'eau
[2] Lavent les mains avec de l'eau uniquement
[3] Frottent les mains avec des feuilles/de la paille/de l'herbe
[4] Frottent les mains avec du sable
[5] Frottent les mains avec un chiffon/du tissu
[6] Ne lavent pas, pas besoin/pas sale
[7] Ne lavent pas, rien pour laver les mains
[8] Autre, préciser _________
[9] Pas applicable (pas d'animaux de ferme/bétail ; pas d'animaux domestiques/de compagnie
[99] Ne sait pas [98] Pas de réponse

## CULTURES AGRICOLES

**34 Quel genre de cultures les membres de ce ménage font-ils pousser/cultivent-ils ?**

*Instruction :* Cocher plusieurs cases si nécessaire ; il peut y avoir plus d'une réponse.

O 1=Café
O 2=Riz
O 3=Fruits (cultivés sur arbustes/arbre)
O 4=Fruits (cultivés au sol)
O 5=Légumes/salade (cultivés sur arbustes/arbre)
O 6=Légumes/salade (cultivés au sol)
O 7=Céréales (blé, orge, avoine, maïs)
O 8=Thé
O 9=Noix de cajou
O 10=Tabac
O 11=Canne à Sucre
O 12=Chad/Chat
O 13=Bees/Honey
O 14=Coton
O 15=PAS DE CULTURES
O 16=Autre, préciser _________
O 99=Ne sait pas
O 98=Pas de réponse

**35 Où les champs/terres agricoles des membres de ce ménage se situent-ils/elles?**

O 1=On the property of the house (i.e. back-/courtyard)
O 4=Outside the house in the community
O 2=Outside the community
O 5=Not applicable (no crop farming)
O 3=Other, specify _________
O 99=Don't know
O 98=No response

**36 Quelle est l'importance des cultures des membres de ce ménage?**

- O 1=Leurs propres besoins (« agriculture d'arrière-cour »)
- O 2=2=Pas applicable (pas de cultures)
- O 3=Demande massive/industrielle (agriculture intensive)
- O 4=Autre, préciser _________
- O 99=Ne sait pas
- O 98= Pas de réponse

**37 Les membres de ce ménage utilisent-ils habituellement des herbicides, pesticides et engrais pour les cultures indiquées à la question 34?**

*Instruction :* Indiquer un code par catégorie uniquement.

| Herbicides | Pesticides | Engrais |
|---|---|---|
| O 1=Oui, préciser l'herbicide ______________________ | O 1=Oui, préciser le pesticide ______________________ | O 1=Oui, préciser l'engrais ______________________ |
| O 2=Pas applicable (pas de cultures) | O 2=Pas applicable (pas de cultures) | O 2=Pas applicable (pas de cultures) |
| O 3=Pas d'utilisation des herbicides | O 3=Pas d'utilisation des pesticides | O 3=Pas d'utilisation des engrais |
| O 99=Ne sait pas | O 99=Ne sait pas | O 99=Ne sait pas |
| O 98=Pas de réponse | O 98=Pas de réponse | O 98=Pas de réponse |

**38 Les membres de ce ménage se lavent-ils les mains après avoir travaillé dans les terres agricoles pour les cultures indiquées à la question 34 ?**

- O 1=Oui, se lavent les mains avec du savon et de l'eau
- O 2=Oui, se frottent les mains avec des feuilles/de la paille/de l'herbe
- O 3=Oui, se frottent les mains avec des chiffons/du tissu
- O 4=Pas applicable (pas de cultures)
- O 5=Oui, se lavent les mains avec de l'eau uniquement
- O 6=Oui, se frottent les mains avec du sable
- O 7=Non, ne se lavent pas les mains
- O 8=Autre, préciser __________
- O 99=Ne sait pas
- O 98= Pas de réponse

Site ___ ___ ___ Sous-zone ___ ___ ___ Numéro d'habitation ___ ___ ___ ___ Identifiant de l'enquêteur ___ __

**FORMULAIRE 4: ÉVALUATION DE L'UTILISATION GÉNÉRIQUE DES SOINS DE SANTÉ GLOBAUX, STRATIFIÉE SELON L'ÂGE ET LE SEXE**
**Partie A –Évaluation de l'utilisation *générique* des soins de santé**

## Instructions pour l'enquêteur

- Compléter ce formulaire uniquement si la personne interrogée a consenti à participer (voir « Formulaire 2 — FORMULAIRE DE CONSENTEMENT ») ; la personne interrogée ainsi que définie pour cette enquête est un adulte (selon l'âge légal de majorité du pays), un membre du ménage et un décideur en ce qui concerne les soins de santé quotidiens et l'utilisation de soins de santé pour le ménage entier et ses membres ; il ne doit pas être, par exemple, un voisin ou un visiteur.
- Se référer à « Formulaire 3 — Partie A — Informations générales sur le ménage » comme référence pour obtenir le nombre total de membres du ménage ainsi que l'âge et le sexe de chaque membre du ménage.
- Recueillir les données de tous les membres du ménage que tous les membres du ménage soient présents ou non au moment de l'entretien ; ne recueillir et n'inscrire les données que des personnes présentes au moment de l'entretien fausserait les données.
- Un ménage est défini/les membres d'un ménage sont définis comme une personne ou un groupe de personnes avec ou sans lien de parenté qui vivent ensemble dans la même unité d'habitation, qui reconnaissent un adulte de sexe masculin ou féminin comme chef de ménage, qui partagent les mêmes installations domestiques, qui sont considérés comme constituant une unité, et qui subviennent à leurs besoins en termes de nourriture et des autres éléments essentiels pour vivre.
- « Site » et « sous-zone » doivent être un numéro à trois chiffres, « numéro d'habitation » un numéro à quatre chiffres, et « identifiant de l'enquêteur » un numéro à deux chiffres (utiliser les listes préparées individuellement pour chaque site pour obtenir les codes) ; saisir « date » au format JJ/MM/AAAA et « heure » au format HH:MM et entourer MATIN ou APRÈS-MIDI. «Étiquette de l'étude» doit être une combinaison d'un code «site» à trois chiffres, d'un code «sous-zone» à trois chiffres et d'un code «numéro d'habitation» à quatre chiffres; le « identifiant de l'enquêteur » doit être un numéro à trois chiffres.
- Abréviation : EDS = Établissement de santé public/privé primaire/secondaire/tertiaire.

## MEMBRES DU MÉNAGE

**1 Indiquer le nombre total de membres du ménage de cette habitation** (au jour de la visite).
*Instructions :* Le nombre total de membres du ménage doit être indiqué à l'aide d'un numéro à 2 chiffres ; voir également « Formulaire 3 — Partie A — INFORMATIONS GÉNÉRALES SUR LE MÉNAGE ».

Nombre total de membres du ménage : ____ ____

**2 Indiquer le nombre de membres FÉMININS du ménage pour chacun des groupes d'âge suivants :**
*Instructions :* Le nombre total de membres féminins du ménage par groupe d'âge doit être indiqué à l'aide d'un numéro à 2 chiffres ; voir également « Formulaire 3 — Partie A — INFORMATIONS GÉNÉRALES SUR LE MÉNAGE ».

<2 ans : ____ ____ ≥2 à <5 ans : ____ ____ ≥5 à <15 ans : ____ ____ ≥15 ans : ____ ____

**3 Indiquer le nombre de membres MASCULINS du ménage pour chacun des groupes d'âge suivants :**
*Instructions :* Le nombre total de membres masculins du ménage par groupe d'âge doit être indiqué à l'aide d'un numéro à 2 chiffres ; voir également « Formulaire 3 — Partie A — INFORMATIONS GÉNÉRALES SUR LE MÉNAGE ».

<2 ans : ____ ____ ≥2 à <5 ans : ____ ____ ≥5 à <15 ans : ____ ____ ≥15 ans : ____ ____

## COMPORTEMENT DE RECHERCHE DE SOINS DE SANTÉ

**4 Indiquer où les membres féminins/masculins du foyer d'âges <2 ans/≥2 à <5 ans/≥5 à <15 ans/≥15 ans recherchent habituellement des soins de santé pour les signes/symptômes suivants. Merci d'attribuer une note entre 1 et 4.**

*Instructions :*
- Compléter la question 4 séparément pour les membres du ménage féminins et masculins d'âge <2 ans, ≥2 à <5 ans, ≥5 à <15 ans et ≥15 ans et entoure le sexe et le groupe d'âge pour lequel Q4 a été completée; passer la question 4 s'il n'y a aucun membre féminin/masculin du ménage d'âge <2 ans/ ≥2 à <5 ans/ ≥5 à <15 ans/ ≥15 ans.
- Indiquer une réponse pour chaque choix de soins de santé pour chaque signe/symptôme; attribuer une note de 1 à 4 (1=1er choix; 2=2ème choix; 3=3ème choix; 4=non choisi); indiquer un code s'il y a lieu en utilisant les listes préparées (utiliser listes préparées individuellement pour chaque site pour obtenir les codes); cocher «Ne sait pas» s'il y a lieu.

| Signe/Symptôme | Choix de soins de santé | | | | | | | | | |
|---|---|---|---|---|---|---|---|---|---|---|
| | EDS SETA | | Autre EDS | | Médecin | Pharmacie | Guérisseur traditionnel | Nulle part Auto-médication | Nulle part Rien | Ne sait pas |
| | Note | Code | Note | Code | Note | Note | Note | Note | Note | Cocher |
| **1** [**A**] Fièvre | ○=1<br>○=2<br>○=3<br>○=4 | | ○=1<br>○=2<br>○=3<br>○=4 | | ○=1<br>○=2<br>○=3<br>○=4 | ○=1<br>○=2<br>○=3<br>○=4 | ○=1<br>○=2<br>○=3<br>○=4 | ○=1<br>○=2<br>○=3<br>○=4 | ○=1<br>○=2<br>○=3<br>○=4 | ○ |
| **2** Fièvre <3 jours (continus) | ○=1<br>○=2<br>○=3<br>○=4 | | ○=1<br>○=2<br>○=3<br>○=4 | | ○=1<br>○=2<br>○=3<br>○=4 | ○=1<br>○=2<br>○=3<br>○=4 | ○=1<br>○=2<br>○=3<br>○=4 | ○=1<br>○=2<br>○=3<br>○=4 | ○=1<br>○=2<br>○=3<br>○=4 | ○ |
| **3** Fièvre ≥3 jours (continus) | ○=1<br>○=2<br>○=3<br>○=4 | | ○=1<br>○=2<br>○=3<br>○=4 | | ○=1<br>○=2<br>○=3<br>○=4 | ○=1<br>○=2<br>○=3<br>○=4 | ○=1<br>○=2<br>○=3<br>○=4 | ○=1<br>○=2<br>○=3<br>○=4 | ○=1<br>○=2<br>○=3<br>○=4 | ○ |
| **4** Frissons Tremblements | ○=1<br>○=2<br>○=3<br>○=4 | | ○=1<br>○=2<br>○=3<br>○=4 | | ○=1<br>○=2<br>○=3<br>○=4 | ○=1<br>○=2<br>○=3<br>○=4 | ○=1<br>○=2<br>○=3<br>○=4 | ○=1<br>○=2<br>○=3<br>○=4 | ○=1<br>○=2<br>○=3<br>○=4 | ○ |
| **5** Convulsion | ○=1<br>○=2<br>○=3<br>○=4 | | ○=1<br>○=2<br>○=3<br>○=4 | | ○=1<br>○=2<br>○=3<br>○=4 | ○=1<br>○=2<br>○=3<br>○=4 | ○=1<br>○=2<br>○=3<br>○=4 | ○=1<br>○=2<br>○=3<br>○=4 | ○=1<br>○=2<br>○=3<br>○=4 | ○ |
| **6** Perte de poids | ○=1<br>○=2<br>○=3<br>○=4 | | ○=1<br>○=2<br>○=3<br>○=4 | | ○=1<br>○=2<br>○=3<br>○=4 | ○=1<br>○=2<br>○=3<br>○=4 | ○=1<br>○=2<br>○=3<br>○=4 | ○=1<br>○=2<br>○=3<br>○=4 | ○=1<br>○=2<br>○=3<br>○=4 | ○ |
| **7** Déshydratation | ○=1<br>○=2<br>○=3<br>○=4 | | ○=1<br>○=2<br>○=3<br>○=4 | | ○=1<br>○=2<br>○=3<br>○=4 | ○=1<br>○=2<br>○=3<br>○=4 | ○=1<br>○=2<br>○=3<br>○=4 | ○=1<br>○=2<br>○=3<br>○=4 | ○=1<br>○=2<br>○=3<br>○=4 | ○ |
| **8** Malaise Faiblesse/ Fatigue | ○=1<br>○=2<br>○=3<br>○=4 | | ○=1<br>○=2<br>○=3<br>○=4 | | ○=1<br>○=2<br>○=3<br>○=4 | ○=1<br>○=2<br>○=3<br>○=4 | ○=1<br>○=2<br>○=3<br>○=4 | ○=1<br>○=2<br>○=3<br>○=4 | ○=1<br>○=2<br>○=3<br>○=4 | ○ |
| **9** Douleurs (muscles/articulations /os) | ○=1<br>○=2<br>○=3<br>○=4 | | ○=1<br>○=2<br>○=3<br>○=4 | | ○=1<br>○=2<br>○=3<br>○=4 | ○=1<br>○=2<br>○=3<br>○=4 | ○=1<br>○=2<br>○=3<br>○=4 | ○=1<br>○=2<br>○=3<br>○=4 | ○=1<br>○=2<br>○=3<br>○=4 | ○ |
| **10** Maux de tête | ○=1<br>○=2<br>○=3<br>○=4 | | ○=1<br>○=2<br>○=3<br>○=4 | | ○=1<br>○=2<br>○=3<br>○=4 | ○=1<br>○=2<br>○=3<br>○=4 | ○=1<br>○=2<br>○=3<br>○=4 | ○=1<br>○=2<br>○=3<br>○=4 | ○=1<br>○=2<br>○=3<br>○=4 | ○ |
| **11** Vertiges/ Confusion Perte de connaissance | ○=1<br>○=2<br>○=3<br>○=4 | | ○=1<br>○=2<br>○=3<br>○=4 | | ○=1<br>○=2<br>○=3<br>○=4 | ○=1<br>○=2<br>○=3<br>○=4 | ○=1<br>○=2<br>○=3<br>○=4 | ○=1<br>○=2<br>○=3<br>○=4 | ○=1<br>○=2<br>○=3<br>○=4 | ○ |
| **12** Troubles de la pression artérielle | ○=1<br>○=2<br>○=3<br>○=4 | | ○=1<br>○=2<br>○=3<br>○=4 | | ○=1<br>○=2<br>○=3<br>○=4 | ○=1<br>○=2<br>○=3<br>○=4 | ○=1<br>○=2<br>○=3<br>○=4 | ○=1<br>○=2<br>○=3<br>○=4 | ○=1<br>○=2<br>○=3<br>○=4 | ○ |
| **13** Troubles cardiaques | ○=1<br>○=2<br>○=3<br>○=4 | | ○=1<br>○=2<br>○=3<br>○=4 | | ○=1<br>○=2<br>○=3<br>○=4 | ○=1<br>○=2<br>○=3<br>○=4 | ○=1<br>○=2<br>○=3<br>○=4 | ○=1<br>○=2<br>○=3<br>○=4 | ○=1<br>○=2<br>○=3<br>○=4 | ○ |

| Signe/Symptôme | Choix de soins de santé | | | | | | | | | |
|---|---|---|---|---|---|---|---|---|---|---|
| | EDS SETA | | Autre EDS | | Médecin | Pharmacie | Guérisseur traditionnel | Nulle part Auto-médication | Nulle part Rien | Ne sait pas |
| | Note | Code | Note | Code | Note | Note | Note | Note | Note | Cocher |
| **14** Nausée Vomissements | O=1<br>O=2<br>O=3<br>O=4 | | O=1<br>O=2<br>O=3<br>O=4 | | O=1<br>O=2<br>O=3<br>O=4 | O=1<br>O=2<br>O=3<br>O=4 | O=1<br>O=2<br>O=3<br>O=4 | O=1<br>O=2<br>O=3<br>O=4 | O=1<br>O=2<br>O=3<br>O=4 | O |
| **15** Diarrhée (liquide/sanglante/ mucopurulente) | O=1<br>O=2<br>O=3<br>O=4 | | O=1<br>O=2<br>O=3<br>O=4 | | O=1<br>O=2<br>O=3<br>O=4 | O=1<br>O=2<br>O=3<br>O=4 | O=1<br>O=2<br>O=3<br>O=4 | O=1<br>O=2<br>O=3<br>O=4 | O=1<br>O=2<br>O=3<br>O=4 | O |
| **16** Douleur abdominale Douleur intestinale | O=1<br>O=2<br>O=3<br>O=4 | | O=1<br>O=2<br>O=3<br>O=4 | | O=1<br>O=2<br>O=3<br>O=4 | O=1<br>O=2<br>O=3<br>O=4 | O=1<br>O=2<br>O=3<br>O=4 | O=1<br>O=2<br>O=3<br>O=4 | O=1<br>O=2<br>O=3<br>O=4 | O |
| **17** Perte de sang/ Hémorragie (interne) | O=1<br>O=2<br>O=3<br>O=4 | | O=1<br>O=2<br>O=3<br>O=4 | | O=1<br>O=2<br>O=3<br>O=4 | O=1<br>O=2<br>O=3<br>O=4 | O=1<br>O=2<br>O=3<br>O=4 | O=1<br>O=2<br>O=3<br>O=4 | O=1<br>O=2<br>O=3<br>O=4 | O |
| **18** Perte de sang/ Hémorragie (externe) | O=1<br>O=2<br>O=3<br>O=4 | | O=1<br>O=2<br>O=3<br>O=4 | | O=1<br>O=2<br>O=3<br>O=4 | O=1<br>O=2<br>O=3<br>O=4 | O=1<br>O=2<br>O=3<br>O=4 | O=1<br>O=2<br>O=3<br>O=4 | O=1<br>O=2<br>O=3<br>O=4 | O |
| **19** Éternuements Nez qui coule | O=1<br>O=2<br>O=3<br>O=4 | | O=1<br>O=2<br>O=3<br>O=4 | | O=1<br>O=2<br>O=3<br>O=4 | O=1<br>O=2<br>O=3<br>O=4 | O=1<br>O=2<br>O=3<br>O=4 | O=1<br>O=2<br>O=3<br>O=4 | O=1<br>O=2<br>O=3<br>O=4 | O |
| **20** Toux | O=1<br>O=2<br>O=3<br>O=4 | | O=1<br>O=2<br>O=3<br>O=4 | | O=1<br>O=2<br>O=3<br>O=4 | O=1<br>O=2<br>O=3<br>O=4 | O=1<br>O=2<br>O=3<br>O=4 | O=1<br>O=2<br>O=3<br>O=4 | O=1<br>O=2<br>O=3<br>O=4 | O |
| **21** Respiration rapide/ essoufflement | O=1<br>O=2<br>O=3<br>O=4 | | O=1<br>O=2<br>O=3<br>O=4 | | O=1<br>O=2<br>O=3<br>O=4 | O=1<br>O=2<br>O=3<br>O=4 | O=1<br>O=2<br>O=3<br>O=4 | O=1<br>O=2<br>O=3<br>O=4 | O=1<br>O=2<br>O=3<br>O=4 | O |
| **22** Difficultés respiratoires graves | O=1<br>O=2<br>O=3<br>O=4 | | O=1<br>O=2<br>O=3<br>O=4 | | O=1<br>O=2<br>O=3<br>O=4 | O=1<br>O=2<br>O=3<br>O=4 | O=1<br>O=2<br>O=3<br>O=4 | O=1<br>O=2<br>O=3<br>O=4 | O=1<br>O=2<br>O=3<br>O=4 | O |
| **23** Inflammation de la peau (éruptions cutanées/ rougeurs/pus/ démangeaisons) | O=1<br>O=2<br>O=3<br>O=4 | | O=1<br>O=2<br>O=3<br>O=4 | | O=1<br>O=2<br>O=3<br>O=4 | O=1<br>O=2<br>O=3<br>O=4 | O=1<br>O=2<br>O=3<br>O=4 | O=1<br>O=2<br>O=3<br>O=4 | O=1<br>O=2<br>O=3<br>O=4 | O |
| **24** Gonflement/ Œdème | O=1<br>O=2<br>O=3<br>O=4 | | O=1<br>O=2<br>O=3<br>O=4 | | O=1<br>O=2<br>O=3<br>O=4 | O=1<br>O=2<br>O=3<br>O=4 | O=1<br>O=2<br>O=3<br>O=4 | O=1<br>O=2<br>O=3<br>O=4 | O=1<br>O=2<br>O=3<br>O=4 | O |
| **25** Jaunisse | O=1<br>O=2<br>O=3<br>O=4 | | O=1<br>O=2<br>O=3<br>O=4 | | O=1<br>O=2<br>O=3<br>O=4 | O=1<br>O=2<br>O=3<br>O=4 | O=1<br>O=2<br>O=3<br>O=4 | O=1<br>O=2<br>O=3<br>O=4 | O=1<br>O=2<br>O=3<br>O=4 | O |

**FORMULAIRE 4: ÉVALUATION DE L'UTILISATION GÉNÉRIQUE DES SOINS DE SANTÉ GLOBAUX, STRATIFIÉE SELON L'ÂGE ET LE SEXE**
**Partie B – Facteurs associés aux soins de santé/Vaccination/Percéption de la maladie**

## DÉPLACEMENT À L'ÉTABLISSEMENT DE SANTÉ

**1 Au cas où un établissement de santé (EDS) a été noté comme 1er choix de soins à la question 4A (fièvre), quel est habituellement le moyen de transport principal, le temps de trajet (selon le moyen de transport), le coût du trajet (selon le moyen de transport) et la distance du trajet (selon le moyen de transport) pour s'y rendre ?**

*Instructions :* Indiquer uniquement un code par catégorie, sexe ainsi que groupe d'âge. Passer cette question s'il n'y a pas de membre féminin/masculin d'âge <2 ans/≥2 à <5 ans/≥5 à <15 ans/≥15 ans dans ce ménage (voir également questions 2 et 3 de ce formulaire Partie A). Passer cette question si aucun EDS n'a été noté comme 1er choix de soins à la question 4A pour les membres masculins/féminins d'âge <2 ans/≥2 à <5 ans/≥5 à <15 ans/≥15 ans. Indiquer le temps de trajet en minute(s) [min]/ heure(s) [h], le coût total en USD, y compris les cadeaux éventuels, les aides financières et les dons, et la distance totale en mètre(s) [m]/kilomètre(s) [km].

| Membre féminin | | | | Membre masculin | | | |
|---|---|---|---|---|---|---|---|
| <2 ans | ≥2 à <5 ans | ≥5 à <15 ans | ≥15 ans | <2 ans | ≥2 à <5 ans | ≥5 à <15 ans | ≥15 ans |
| **1.1 Moyen de transport** | | | | | | | |
| Code ____ | Code ____ | Code ____ | Code ____ | Code ____ | Code ____ | Code ____ | Code ____ |
| [1] Private transportation (i.e. motorbike, car, bus)<br>[2] Public transportation (i.e. motorbike, car, bus) | | | | [3] Walking<br>[4] Bicycle | [5] Other, specify ________<br>[99] Don't know [98] No response | | |
| **1.2 Temps de trajet** | | | | | | | |
| Code ____ | Code ____ | Code ____ | Code ____ | Code ____ | Code ____ | Code ____ | Code ____ |
| [1] <30 min<br>[2] ≥30 à <60 min | | [3] ≥1.0 à <2.0 h<br>[4] ≥2.0 à <3.0 h | | [5] ≥3.0 à <4.0 h<br>[6] ≥4.0h | | [99] Ne sait pas<br>[98] Pas de réponse | |
| **1.3 Coût du transport** | | | | | | | |
| Code ____ | Code ____ | Code ____ | Code ____ | Code ____ | Code ____ | Code ____ | Code ____ |
| [1] <1.0 USD<br>[2] ≥1.0 à <3.0 USD | | [3] ≥3.0 à <5.0 USD<br>[4] ≥5.0 à <10.0 USD | | [5] ≥10.0 USD | | [99] Ne sait pas<br>[98] Pas de réponse | |
| **1.4 Distance du trajet** | | | | | | | |
| Code ____ | Code ____ | Code ____ | Code ____ | Code ____ | Code ____ | Code ____ | Code ____ |
| [1] <500 m<br>[2] ≥500 m à <1.0 km | | [3] ≥1.0 à <2.0 km<br>[4] ≥2.0 à <3.0 km | | [5] ≥3.0 à <4.0 km<br>[6] ≥4.0 km | | [99] Ne sait pas<br>[98] Pas de réponse | |

## ASSURANCE

**2 Pour quels membres de ce ménage l'assurance couvre-t-elle les dépenses de santé ?**

*Instructions :* Indiquer un code par catégorie. Passer cette question s'il n'y a aucun membre d'âge <2 ans/≥2 à <5 ans/≥5 à <15 ans/≥15 ans dans ce ménage (voir questions 2 et 3 de ce formulaire partie A).

| <2 ans | ≥2 à <5 ans | ≥5 à <15 ans | ≥15 ans |
|---|---|---|---|
| Code ____________ | Code ____________ | Code ____________ | Code ____________ |
| [1] Oui | [2] Non [99] Ne sait pas | [98] Pas de réponse | |

**3 Quels types de dépenses de santé sont habituellement couverts par l'assurance pour les membres de ce ménage ?**

*Instructions :* Indiquer uniquement un code par catégorie et par groupe d'âge. Passer cette question s'il n'y a aucun membre d'âge <2 ans/≥2 à <5 ans/≥5 à <15 ans/≥15 ans dans ce ménage (voir également les questions 2 et 3 de ce formulaire partie

A). Passer cette question s'il n'y a aucune assurance qui couvre les dépenses de santé des membres d'âge <2 ans/≥2 à <5 ans/≥5 à <15 ans/≥15 ans.

| | <2 ans | ≥2 à <5 ans | ≥5 à <15 ans | ≥15 ans |
|---|---|---|---|---|
| | [1] Oui réponse | [2] Non | [99] Ne sait pas | [98] Pas de |
| **1** Inscription | Code _______ | Code _______ | Code _______ | Code _______ |
| **2** Consultation/Visite chez le médecin | Code _______ | Code _______ | Code _______ | Code _______ |
| **3** Examen physique - général | Code _______ | Code _______ | Code _______ | Code _______ |
| **4** Examen physique – spécifique, préciser_______ | Code _______ | Code _______ | Code _______ | Code _______ |
| **5** Diagnostics - diagnostics généraux de laboratoire | Code _______ | Code _______ | Code _______ | Code _______ |
| **6** Diagnostics – diagnostics spécifiques de laboratoire, préciser__________ | Code _______ | Code _______ | Code _______ | Code _______ |
| **7** Diagnostics Imagerie générale | Code _______ | Code _______ | Code _______ | Code _______ |
| **8** Diagnostics Imagerie spécifique, préciser__________ | Code _______ | Code _______ | Code _______ | Code _______ |
| **9** Diagnostics Autre, préciser________ | Code _______ | Code _______ | Code _______ | Code _______ |
| **10** Traitement Médication générale | Code _______ | Code _______ | Code _______ | Code _______ |
| **11** Traitement Médication spécifique, préciser________ | Code _______ | Code _______ | Code _______ | Code _______ |
| **12** Traitement Autre médication, préciser__________ | Code _______ | Code _______ | Code _______ | Code _______ |
| **13** Traitement Interventions (autres que chirurgie), préciser_______ | Code _______ | Code _______ | Code _______ | Code _______ |
| **14** Traitement Chirurgie | Code _______ | Code _______ | Code _______ | Code _______ |
| **15** Hospitalisation <7 jours | Code _______ | Code _______ | Code _______ | Code _______ |
| **16** Hospitalisation ≥7 jours | Code _______ | Code _______ | Code _______ | Code _______ |
| **17** Services d'urgence | Code _______ | Code _______ | Code _______ | Code _______ |
| **18** Soins prénatals | Code _______ | Code _______ | Code _______ | Code _______ |
| **19** Soins postnatals | Code _______ | Code _______ | Code _______ | Code _______ |
| **20** Médecine préventive | Code _______ | Code _______ | Code _______ | Code _______ |
| **21** Vaccination | Code _______ | Code _______ | Code _______ | Code _______ |
| **22** Soins à domicile (personnes âgées, handicapés, malades chroniques/à long terme) | Code _______ | Code _______ | Code _______ | Code _______ |

| | | | | |
|---|---|---|---|---|
| **23** Thérapie de soutien (physiothérapie, ergothérapie, réhabilitation) | Code _______ | Code _______ | Code _______ | Code _______ |

## ETHNOGRAPHIE

### 4 À quelle religion/groupe ethnique/tribu appartiennent les membres de ce ménage ?

*Instructions :* Donner une réponse par groupe d'âge uniquement. <u>Passer</u> cette question s'il n'y a <u>aucun membre d'âge>2 ans/≥2 à <5 ans/≥5 à <15 ans/≥</u>15 ans dans ce ménage (voir également questions 2 et 3 de ce formulaire Partie A).

| | <2 ans | ≥2 to <5 ans | ≥5 to <15 ans | ≥15 ans |
|---|---|---|---|---|
| Religion/Groupe ethnique/Tribu | _____________ | _____________ | _____________ | _____________ |
| | O 99=Ne sait pas<br>O 98=Pas de réponse | O 99=Ne sait pas<br>O 98=Pas de réponse | O 99=Ne sait pas<br>O 98=Pas de réponse | O 99=Ne sait pas<br>O 98=Pas de réponse |

### 5 La religion/le groupe ethnique/la tribu comme indiqué à la question 4 influence-t-elle/il les différents aspects de l'utilisation des soins de santé pour les membres de ce ménage ?

*Instructions :* Indiquer un code par catégorie et groupe d'âge uniquement. <u>Passer</u> cette question s'il y a un <u>membre d'âge >2 ans/≥2 à <5 ans/≥5 à <15 ans/≥</u>15 ans dans ce ménage (voir également questions 2 t 3 de ce formulaire Partie A).

| | <2 ans | ≥2 à <5 ans | ≥5 to <15 ans | ≥15 ans |
|---|---|---|---|---|
| | [1] Oui | [2] Non | [99] Ne sait pas | [98] pas réponse |
| **1** Visite dans un établissement de santé (hôpital, centre de soins, poste de santé) | Code _____ | Code _______ | Code _______ | Code _____ |
| **2** Visite chez un médecin | Code _____ | Code _______ | Code _______ | Code _____ |
| **3** Visite dans une pharmacie | Code _____ | Code _______ | Code _______ | Code _____ |
| **4** Visite chez un guérisseur traditionnel | Code _____ | Code _______ | Code _______ | Code _____ |
| **5** Décision d'automédication | Code _____ | Code _______ | Code _______ | Code _____ |
| **6** Décision de non traitement | Code _____ | Code _______ | Code _______ | Code _____ |
| **7** Examen physique - général | Code _____ | Code _______ | Code _______ | Code _____ |
| **8** Examen physique – spécifique, préciser_______ | Code _____ | Code _______ | Code _______ | Code _____ |
| **9** Prélèvements d'échantillons (sang, urine, selles, frottis, écouvillons, liquide cérébro-spinal) | Code _____ | Code _______ | Code _______ | Code _____ |
| **10** Diagnostics - diagnostics généraux de laboratoire | Code _____ | Code _______ | Code _______ | Code _____ |
| **11** Diagnostics – diagnostics spécifiques de laboratoire, préciser__________ | Code _____ | Code _______ | Code _______ | Code _____ |
| **12** Diagnostics Imagerie générale | Code _____ | Code _______ | Code _______ | Code _____ |
| **13** Diagnostics Imagerie spécifique, préciser__________ | Code _____ | Code _______ | Code _______ | Code _____ |
| **14** Diagnostics Autre, préciser________ | Code _____ | Code _______ | Code _______ | Code _____ |
| **15** Traitement Médication générale | Code _____ | Code _______ | Code _______ | Code _____ |
| **16** Traitement Médication spécifique, préciser________ | Code _____ | Code _______ | Code _______ | Code _____ |

| | | | | |
|---|---|---|---|---|
| **17** Traitement<br>Autre médication, préciser___________ | Code _____ | Code _______ | Code _______ | Code _____ |
| **18** Traitement<br>Interventions (autres que chirurgie), préciser________ | Code _____ | Code _______ | Code _______ | Code _____ |
| **19** Traitement<br>Chirurgie | Code _____ | Code _______ | Code _______ | Code _____ |
| **20** Hospitalisation<br><7 jours | Code _____ | Code _______ | Code _______ | Code _____ |
| **21** Hospitalisation<br>≥7 jours | Code _____ | Code _______ | Code _______ | Code _____ |
| **22** Services d'urgence | Code _____ | Code _______ | Code _______ | Code _____ |
| **23** Soins prénatals | Code _____ | Code _______ | Code _______ | Code _____ |
| **24** Soins postnatals | Code _____ | Code _______ | Code _______ | Code _____ |
| **25** Médecine préventive | Code _____ | Code _______ | Code _______ | Code _____ |
| **26** Vaccination | Code _____ | Code _______ | Code _______ | Code _____ |
| **27** Soins à domicile (personnes âgées, handicapés, malades chroniques/à long terme) | Code _____ | Code _______ | Code _______ | Code _____ |
| **28** Thérapie de soutien (physiothérapie, ergothérapie, réhabilitation) | Code _____ | Code _______ | Code _______ | Code _____ |

## VACCINATION

**6 Comment les enfants de ce ménage ont-ils été nourris pendant les 6 premiers mois de leur vie ?**

*Instructions :* Passer cette question s'il n'y a pas d'enfants de sexe masculin ou féminin dans ce ménage (voir également questions 2 et 3 de ce formulaire partie A).

- O 1=Lait maternisé exclusivement
- O 2=Lait maternisé et autres aliments, préciser ______
- O 3=Allaitement et autres aliments, préciser ________
- O 4=Lait maternisé et allaitement
- O 5=Allaitement exclusivement
- O 6=Autre, préciser _____
- O 99=Ne sait pas
- O 98=Pas de réponse

**7 Quelle source d'approvisionnement en eau était-elle habituellement utilisée pour préparer le lait maternisé ou le lait maternisé et d'autres aliments comme indiqué à la question 6 pour les enfants de ce ménage ?**

*Instructions :* Passer cette question s'il n'y a pas d'enfants dans ce ménage (voir également les questions 2 et 3 de ce formulaire Partie A).

- O 1=Eau du robinet (à l'intérieur de l'habitation, privé)
- O 2=Eau du robinet (à l'extérieur de l'habitation, arrière- cour/cour, privé)
- O 3=Eau du robinet (à l'extérieur de l'habitation, public, partagé)
- O 4=Borne-fontaine/Eau courante (à l'intérieur de l'habitation, privé)
- O 5=Borne-fontaine/Eau courante (à l'extérieur de l'habitation, arrière- cour/cour, privé)
- O 6=Borne-fontaine/Eau courante (à l'extérieur de l'habitation, public, partagé)
- O 7=Camion-citerne (public, partagé)
- O 8=Eau en bouteille
- O 9=Eaux de surface (lac, étang, rivière, ruisseau)
- O 10=Eaux de surface (canal, canal d'irrigation)
- O 11=Eau de source
- O 12=Eau de pluie
- O 13=Puits/Puits de forage (ouvert/non couvert/non protégé)
- O 14=Puits/Puits de forage (couvert/protégé)
- O 15=NO FORMULA/OTHER FEEDS PREPARED
- O 16=Autre, préciser _____________
- O 99=Ne sait pas
- O 98=Pas de réponse

**8** **Est-ce que les enfants de ce ménage ont été vaccinés depuis leur naissance, y compris les vaccins de naissance ?**

*Instructions :* Indiquer un code par catégorie et par enfant uniquement. Passer cette question s'il n'y a aucun enfant dans ce ménage (voir également les questions 2 et 3 de ce formulaire PartieA). Se référer à la question 9 « Formulaire 3 Partie A – Données démographiques/Informations générales sur le ménage » et indiquer les initiales/identificateur du/des membre(s).

| Enfant 1<br>Initiales/Identifiant : ____ | Enfant 2<br>Initiales/Identifiant : ____ | Enfant 3<br>Initiales/Identifiant : ____ | Enfant 4<br>Initiales/Identifiant : ____ |
|---|---|---|---|
| [1] OUI, vacciné [5] Non, manque de vaccins [9] Non, EDS débordé<br>[2] Non, parent/tuteur débordé [6] Non, enfant trop jeune/vieux [10] Non, enfant malade<br>[3] Non, parent/tuteur a décidé que pas nécessaire [7] Non, parent/tuteur a refusé/trop inquiet [11] Non, pas de carte de vaccination<br>[4] Non, ne sait pas où aller [8] Non, Autre, préciser ____________ [99] Don't know [98] No response | | | |
| **8.1 Diphtérie et tétanos et coqueluche et *Haemophilus influenza* et vaccin contre l'hépatite B / DTwPHibHepB** (par exemple 6/8, 10/12, 14/16 semaines) | | | |
| Code __________ | Code __________ | Code __________ | Code __________ |
| **8.2 Anatoxine tétanique (TT)** (par exemple, 1er contact, grossesse, 1, 6, 12/24 mois) | | | |
| Code __________ | Code __________ | Code __________ | Code __________ |
| **8.3 Anatoxine tétanique et diphtérique (TD)** (par exemple, grossesse avec un premier contact, 1, 6, 12 mois) | | | |
| Code __________ | Code __________ | Code __________ | Code __________ |
| **8.4 Tuberculose / BCG** (à la naissance) | | | |
| Code __________ | Code __________ | Code __________ | Code __________ |
| **8.5 Rotavirus / RV** (par exemple 6/8, 10/12, 16 semaines) | | | |
| Code __________ | Code __________ | Code __________ | Code __________ |
| **8.6 Rougeole / rougeole et rubéole (MR)** (par exemple 9, 15/18 mois) | | | |
| Code __________ | Code __________ | Code __________ | Code __________ |
| **8.7 Polio / VPO** (par exemple naissance, 6/8, 10/12, 14/16 semaines) | | | |
| Code __________ | Code __________ | Code __________ | Code __________ |
| **8.8 Pneumocoque / PCV** (par exemple 6/8, 10/12, 14/16 semaines) | | | |
| Code __________ | Code __________ | Code __________ | Code __________ |
| **8.9 Fièvre jaune (YF)** (par exemple 9 mois) | | | |
| Code __________ | Code __________ | Code __________ | Code __________ |
| **8.10 Autres vaccins** | | | |
| Préciser, __________ | Préciser, __________ | Préciser, __________ | Préciser, __________ |
| Préciser, __________ | Préciser, __________ | Préciser, __________ | Préciser, __________ |

| | | | |
|---|---|---|---|
| Préciser, ___________ | Préciser, ___________ | Préciser, ___________ | Préciser, ___________ |
| Préciser, ___________ | Préciser, ___________ | Préciser, ___________ | Préciser, ___________ |

## PERCEPTION DE LA MALADIE

**9 Connaissance, perception, causes et prévention des maladies infectieuses communes**

| Maladie | **9.1 Maladie connue des membres du ménage ?**<br>[1] Oui<br>[2] Non<br>[99] Ne sait pas<br>[98] Pas de réponse | **9.2 Perception de la maladie par les membres du ménage**<br>*Instruction :* Décrire brièvement si elle est connue. | **9.3 Causes de la maladie connue des membres du ménage ?**<br>*Instructions :* Indiquer tous les codes (liste A) qui s'appliquent; plus d'une réponse est possible | **9.4 Mesures pour prévenir la maladie connues des membres du ménage ?**<br>*Instructions :* Indiquer tous les codes (liste B) qui s'appliquent ; plus d'une réponse est possible. | **9.5 Mesures pour prévenir la maladie utilisées par les membres du foyer ?**<br>*Instructions :* Indiquer tous les codes (liste B) qui s'appliquent ; il peut y avoir plus d'une réponse. |
|---|---|---|---|---|---|
| **A.** Fièvre typhoïde | Code __________ | | Code __________ | Code __________ | Code __________ |
| **B.** Choléra | Code __________ | | Code __________ | Code __________ | Code __________ |
| **C.** Paludisme | Code __________ | | Code __________ | Code __________ | Code __________ |
| **D.** Grippe | Code __________ | | Code __________ | Code __________ | Code __________ |
| **E.** Méningite | Code __________ | | Code __________ | Code __________ | Code __________ |
| **F.** Hépatite E | Code __________ | | Code __________ | Code __________ | Code __________ |
| **G.** Tuberculose | Code __________ | | Code __________ | Code __________ | Code __________ |
| **H.** VIH/SIDA | Code __________ | | Code __________ | Code __________ | Code __________ |

| Liste A: Causes possibles | Liste B: Mesures préventives possibles |
|---|---|
| [1] Consommation d'alcool | [1] Pas de consommation d'alcool |

| | |
|---|---|
| [2] Temps/sorcellerie<br>[3] Piqûre de moustique/d'insecte<br>[4] Morsure de chien<br>[5] Boire de l'eau non traitée de surface/de source (lac, étang, rivière, ruisseau, canal, canal d'irrigation)<br>[6] Contact physique étroit/baiser/embrassade<br>[7] Manger des produits alimentaires crus comme des légumes/de la salade, des fruits, des produits laitiers, des œufs, de la viande ou du poisson<br>[8] Ne pas se laver les mains correctement en préparant la nourriture/les repas<br>[9] Ne pas se laver les mains correctement avant/après être allé aux toilettes<br>[10] Contact avec du sang humain (injection, transfusion, chirurgie)<br>[11] Consommation de sang animal cru<br>[12] Autre, préciser ____________________<br>[99] Ne sait pas<br>[98] Pas de réponse | [2] Dormir sous une moustiquaire<br>[3] Utiliser des sprays insecticides/anti-moustiques<br>[4] Maintenir la maison et ses environs immédiats propres<br>[5] Avoir une pratique correcte de l'élimination des déchets humains et domestiques<br>[6] Éviter le contact rapproché avec des chiens<br>[7] Traiter l'eau (faire bouillir, filtration, désinfection solaire, sédimentation, chloration)<br>[8] Ne pas cuisiner/préparer la nourriture/les repas avec de l'eau non traitée<br>[9] Ne pas laver les produits alimentaires à consommer crus avec de l'eau non traitée<br>[10] Faire bouillir/chauffer à nouveau les restes/les aliments/repas préparés avant de les consommer<br>[11] Ne pas manger des produits alimentaires crus comme légumes/salade, fruits, produits laitiers, œufs, viande ou poisson<br>[12] Couvrir les produits alimentaires/repas<br>[13] Protéger les produits alimentaires/repas contre les mouches<br>[14] Éviter la nourriture/les boissons des vendeurs de rue<br>[15] Éviter le contact rapproché avec les personnes malades<br>[16] Pas de contact physique étroit/baisers/embrassades<br>[17] Se couvrir le nez/la bouche en éternuant/toussant<br>[18] Pas de contact/consommation de sang humain/animal<br>[19] Avoir une bonne hygiène des mains en préparant la nourriture/les repas (se laver les mains avec du savon et de l'eau, se laver les mains par frottement)<br>[20] Avoir une bonne hygiène des mains avant/après être allé aux toilettes (se laver les mains avec du savon et de l'eau, se laver les mains par frottement)<br>[21] Autre, préciser ____________________<br>[99] Ne sait pas<br>[98] Pas de réponse |

**10 Pour quels membres de ce ménage les mesures préventives listées à la question 9.5 contre la fièvre typhoïde sont-elles utilisées ?**

*Instructions :* Cocher plusieurs cases si nécessaire ; il peut y avoir plus d'une réponse.

O 1=<2 ans
O 2=≥2 à < 5 ans
O 3=≥5 à <15 ans
O 4=≥15 ans
O 5=PAS UTILISÉ
O 99=Ne sait pas
O 98=Pas de réponse

**11 Nommer les membres du ménage pour lesquels une fièvre typhoïde a été diagnostiquée dans les 3 derniers mois. Quels éléments de diagnostic ont été utilisés pour confirmer la fièvre typhoïde?**

*Instructions :* Indiquer plusieurs codes si nécessaire pour chaque membre du ménage ; il peut y avoir plus d'une réponse.

| Membre 1<br>Initiales/Identifiant: __ | Membre 2<br>Initiales/Identifiant: __ | Membre 3<br>Initiales/Identifiant: __ | Membre 4<br>Initiales/Identifiant: __ |
|---|---|---|---|
| [1] Hémoculture [3] Test sanguin/de Widal [5] Pas applicable (pas diagnostiqué avec la fièvre typhoïde)<br>[2] Coproculture [4] Autre test, préciser ______________ [99] Ne sait pas [98] Pas de réponse | | | |
| Code ___________ | Code ___________ | Code ___________ | Code ___________ |

**12 (Où les membres de ce ménage indiqués à la question 11 ont-ils été diagnostiqués pour la fièvre typhoïde?) Où les membres de ce ménage ont-ils recherché des soins de santé?**

*Instructions :* Indiquer un code par membre du ménage uniquement.

| Membre 1<br>Initiales/Identifiant: __ | Membre 2<br>Initiales/Identifiant: __ | Membre 3<br>Initiales/Identifiant: __ | Membre 4<br>Initiales/Identifiant: __ |
|---|---|---|---|
| [1] Établissement de santé, préciser _____________<br>[2] Médecin<br>[3] Nulle part, automédication<br>[4] Guérisseur traditionnel<br>[5] Pharmacien | | [6] Nulle part, pas de recherche de soins<br>[7] Autre, préciser _____________<br>[8] Pas applicable<br>[99] Ne sait pas<br>[98] Pas de réponse | |
| Code ___________ | Code ___________ | Code ___________ | Code ___________ |

**13 (Les membres de ce ménage pour lesquels une fièvre typhoïde a été diagnostiquée comme indiqué à la question 11 ont-ils reçu un traitement?) Quel traitement ont-ils reçu?**

*Instructions :* Cocher une réponse par membre du ménage uniquement.

| Membre 1<br>Initiales/Identifiant: __ | Membre 2<br>Initiales/Identifiant: __ | Membre 3<br>Initiales/Identifiant: __ | Membre 4<br>Initiales/Identifiant: __ |
|---|---|---|---|
| O1=Traitement, préciser ________________<br>O 2=Pas applicable<br>O 99=Ne sait pas<br>O 98=Pas de réponse | O1=Traitement, préciser ________________<br>O 2=Pas applicable<br>O 99=Ne sait pas<br>O 98=Pas de réponse | O1=Traitement, préciser ________________<br>O 2=Pas applicable<br>O 99=Ne sait pas<br>O 98=Pas de réponse | O1=Traitement, préciser ________________<br>O 2=Pas applicable<br>O 99=Ne sait pas<br>O 98=Pas de réponse |

Site ___ ___ ___   Sous-zone ___ ___ ___   Numéro d'habitation ___ ___ ___ ___ Identifiant de l'enquêteur ___ __

**FORMULAIRE 5: ÉVALUATION DE L'UTILISATION RÉELLE DES SOINS DE SANTÉ GLOBAUX, STRATIFIÉE SELON L'ÂGE ET LE SEXE**

**Partie A: Évaluation de l'utilisation *réelle* des soins de santé**

**COMPORTEMENT DE RECHERCHE DE SOINS**

**1 Consigner ci-dessous l'apparition de signe(s) ou symptôme(s) (voir liste 1) et préciser où (voir liste 2) et comment le(s) membre(s) a/ont recherché les soins de santé durant les 3 derniers mois ?**

*Instructions* : voir « formulaire 3- partie A-INFORMATIONS GENERALES SUR LE MENAGE » pour obtenir les initiaux/identifiants de chacun des membres du ménage. Indiquer seulement un signe/symptome par ligne. Commencer avec une nouvelle ligne pour tous nouveaux soins de santé sélectionnée pour chaque signe/symptome recurrent. Indiquer le nombre des apparitions de chaque signe/symptômes durant les 3 derniers mois. Si une différente option de soins de santé a été sélectionnée pour un signe/symptôme recurrent, indiquer cela dans une nouvelle ligne. Completer s'il vous plaît, un formulaire supplémentaire s'il y a eu plus de 20 signes/symptômes apparus durant les 3 derniers mois.

| **Liste 1: Signe/Symptôme** | | **Liste 2: Option de soins de santé** |
|---|---|---|
| [A] Fièvre<br>[B] Fièvre <3 jours (continus)<br>[C] Fièvre ≥3 jours (continus)<br>[D] Frissons/Tremblements<br>[E] Convulsion<br>[F] Perte de poids<br>[G] Déshydratation<br>[H] Malaise/Faiblees/Fatigue<br>[I] Douleurs (muscles/articulations/os)<br>[J] Maux de tête<br>[K] Vertiges/Confusion/ Perte de connaissance<br>[L] Troubles de la pression sanguine<br>[M] Troubles cardiaques | [N] Nausée/Vomissements<br>[O] Diarrhée (liquide/sanglante/mucopurulente)<br>[P] Douleur abdominale/Douleur intestinale<br>[Q] Perte de sang/Hémorragie (interne)<br>[R] Perte de sang/Hémorragie (externe)<br>[S] Éternuements Nez qui coule<br>[T] Toux<br>[U] Respiration rapide/Essoufflement<br>[V] Difficultés respiratoires graves<br>[W] Inflammation de la peau (éruptions cutanées/ rougeurs/pus/démangeaisons)<br>[X] Gonflement/Œdème<br>[Y] Jaunisse | [1] EDS-SETA, Code _______________<br>[2] Autre EDS Code _______________<br>[3] Médecin<br>[4] Pharmacie<br>[5] Guérisseur traditionnel<br>[6] Nulle part Auto-médication<br>[7] Nulle part Rien |

| **No.** | **Membre du ménage** Enregistrer les initiaux/identifiants du membre du ménage ; voir formulaire 3 –partie A- INFORMATIONS GENERALES SUR LE MENAGE » | **Signes/ Symptômes** Utiliser la Liste 1 pour sélectionner le code | **Options de soins de santé** | | **Fréquence** Indiquer le nombre d'incidences pour le signe/symptôme listé |
|---|---|---|---|---|---|
| | | | Utiliser la Liste 2 pour sélectionner le code | EDS Code | |
| 1 | | | | | |
| 2 | | | | | |
| 3 | | | | | |
| 4 | | | | | |
| 5 | | | | | |

| | | | | |
|---|---|---|---|---|
| 6 | | | | |
| 7 | | | | |
| 8 | | | | |
| 9 | | | | |
| 10 | | | | |
| 11 | | | | |
| 12 | | | | |
| 13 | | | | |
| 14 | | | | |
| 15 | | | | |
| 16 | | | | |
| 17 | | | | |
| 18 | | | | |
| 19 | | | | |
| 20 | | | | |
