# Supplementary material for: The HPAfrica protocol: Assessment of health behaviour and population-based socioeconomic, hygiene behavioural factors - a standardised repeated cross-sectional study in multiple cohorts in sub-Saharan Africa
Source: BMJ Open. 2018 Dec 19;8(12):e021438. doi: 10.1136/bmjopen-2017-021438 (PMC6303690; doi:10.1136/bmjopen-2017-021438)
Supplement: Supplementary file 4 [file bmjopen-2017-021438supp004.pdf]

Appendix 4/Supplementary file 4: Geospatial random selection with application of simple, weighted-stratified sampling (examples of Ghana and Madagascar)

Figure 1 Geographic random points generated by ArcGIS for Asante Akim North and Central, Ghana

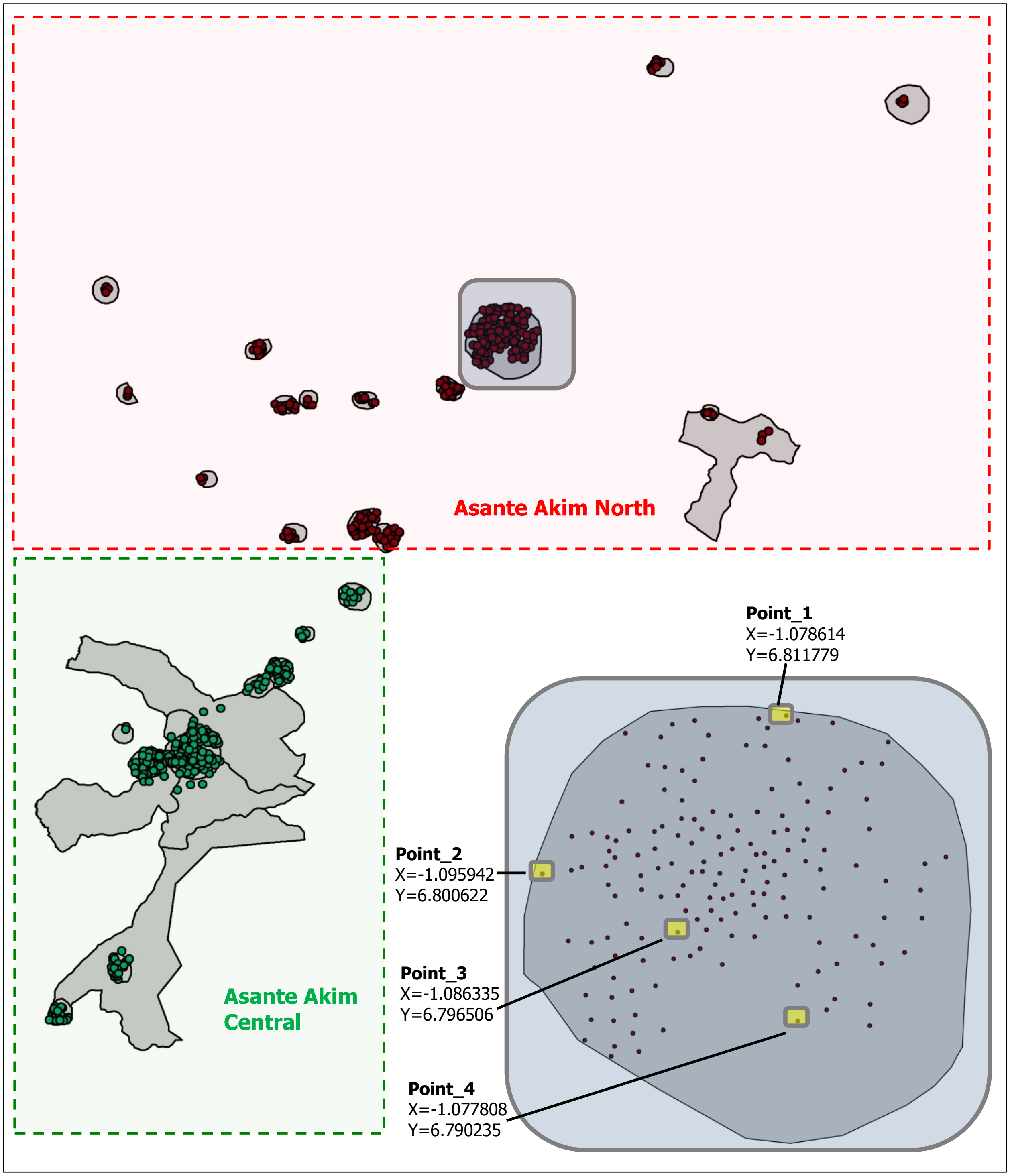

The study area of Asante Akim North (light red rectangle) and Asante Akim Central (light green rectangle) is highlighted in grey. The geographic random points are shown as red and green points for Asante Akim North and Asante Akim Central, respectively. The illustration at the bottom, right highlighted in light blue shows the enlarged area that is indicated as a small central light blue highlighted rectangle. The X- and Y-data of four geographic random points are given as examples; points are indicated as yellow highlighted rectangles. ArcGIS-generated geographic random points converted and visualized in Google Earth Pro® for Asante Akim North and Central, Ghana, can be shared on request.

Figure 2 Geographic random points generated by ArcGIS for Renivohitra/Antananarivo, Madagascar

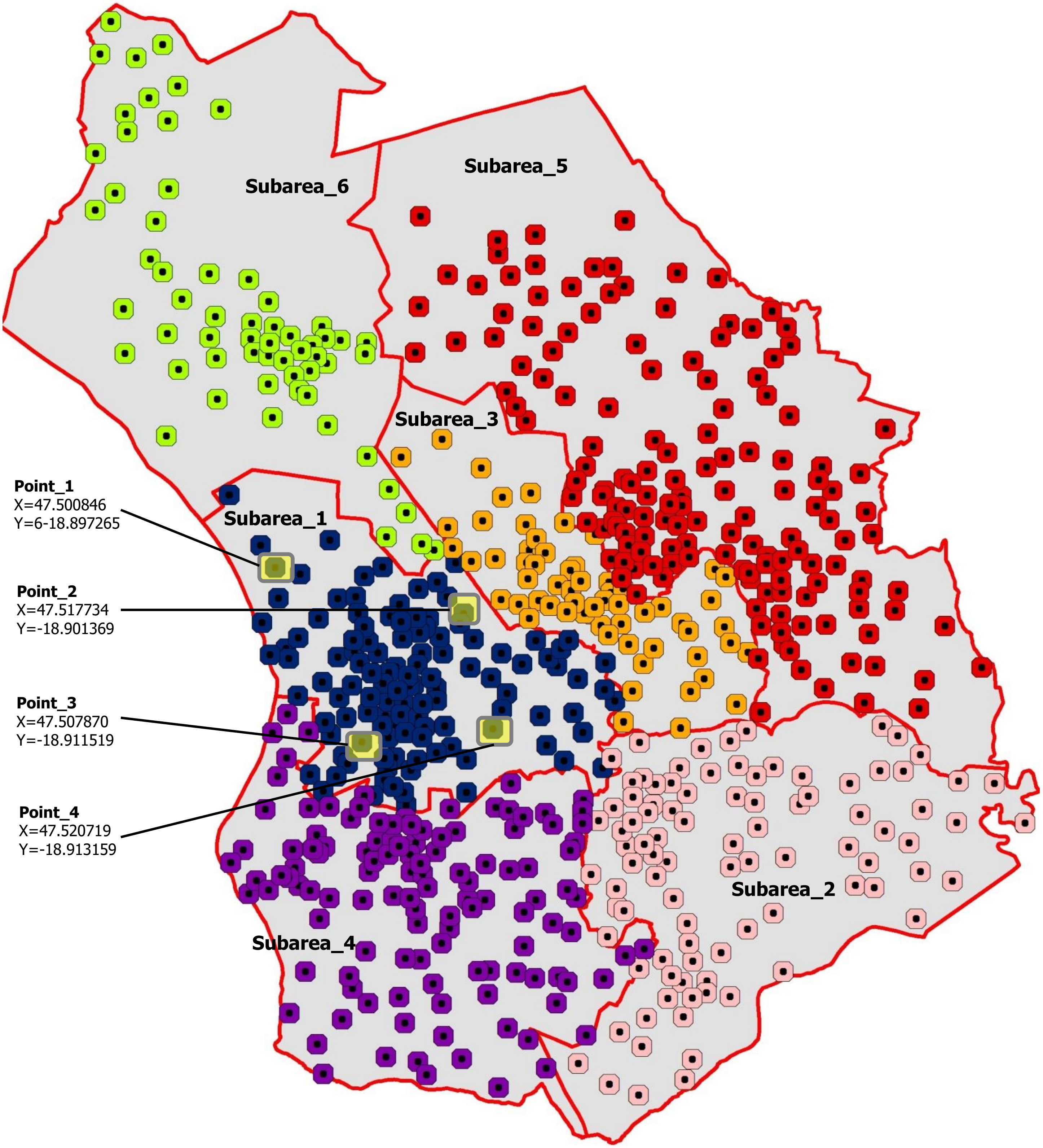

The study area of Renivohitra/Antananarivo, which is subdivided into six administrative subareas, is highlighted in grey. The geographic random points are shown as blue (subarea_1), pink (subarea_2), orange (subarea_3), purple (subarea_4), red (subarea_5) and green (subarea_6) points. The X- and Y-data of four geographic random points are given as examples; points are indicated as yellow highlighted rectangles. ArcGIS-generated geographic random points converted and visualized in Google Earth Pro® for Renivohitra/Antananarivo, Madagascar, can be shared on request.
