# Supplementary material for: The HPAfrica protocol: Assessment of health behaviour and population-based socioeconomic, hygiene behavioural factors - a standardised repeated cross-sectional study in multiple cohorts in sub-Saharan Africa
Source: BMJ Open. 2018 Dec 19;8(12):e021438. doi: 10.1136/bmjopen-2017-021438 (PMC6303690; doi:10.1136/bmjopen-2017-021438)
Supplement: Supplementary file 5 [file bmjopen-2017-021438supp005.pdf]

Appendix 5/Supplementary file 5: System diagram of the data collection and data management platform: the Android application "HPA Collect" and the platform "HPA Web"

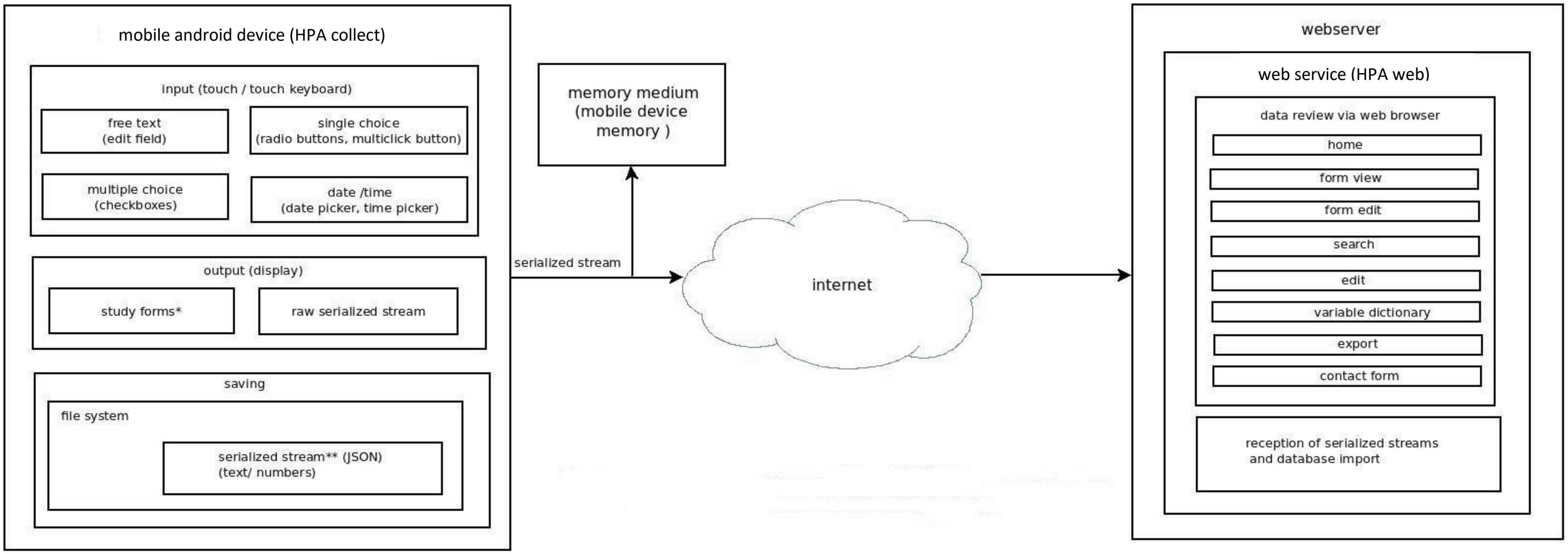

Note. *study forms: Form 1, Form 3 Part A to C, Form 4 Part A to B, Form 5; **serialized stream: two- or more-dimensional data (e.g. a table) is brought to a one-dimensional form; the application uses the JSON (Java Script Object Notation) format.
