# Supplementary material for: The HPAfrica protocol: Assessment of health behaviour and population-based socioeconomic, hygiene behavioural factors - a standardised repeated cross-sectional study in multiple cohorts in sub-Saharan Africa
Source: BMJ Open. 2018 Dec 19;8(12):e021438. doi: 10.1136/bmjopen-2017-021438 (PMC6303690; doi:10.1136/bmjopen-2017-021438)
Supplement: Author's manuscript [file bmjopen-2017-021438.draft_revisions.pdf]

BMJ Open

BMJ Open is committed to open peer review. As part of this commitment we make the peer review history of every article we publish publicly available.

When an article is published we post the peer reviewers' comments and the authors' responses online. We also post the versions of the paper that were used during peer review. These are the versions that the peer review comments apply to.

The versions of the paper that follow are the versions that were submitted during the peer review process. They are not the versions of record or the final published versions. They should not be cited or distributed as the published version of this manuscript.

BMJ Open is an open access journal and the full, final, typeset and author-corrected version of record of the manuscript is available on our site with no access controls, subscription charges or pay-per-view fees (http://bmjopen.bmj.com).

If you have any questions on BMJ Open's open peer review process please email info.bmjopen@bmj.com

BMJ Open

Assessment of health behavior and other population-based factors: a standardized study in multiple cohorts in sub-Saharan Africa

Journal:	*BMJ Open*
Manuscript ID	bmjopen-2017-021438
Article Type:	Protocol
Date Submitted by the Author:	30-Dec-2017

Complete List of Authors:	Pak, GiDeok; International Vaccine Institute, Epidemiology<br>Haselbeck, Andrea; International Vaccine Institute, Epidemiology<br>Seo, Hyeong-Won; International Vaccine Institute<br>Osei, Isaac; Kumasi Centre for Collaborative Research in Tropical Medicine, Kwame Nkrumah University of Science and Technology (KNUST)<br>Amuasi, John; Kumasi Centre for Collaborative Research in Tropical Medicine, Kwame Nkrumah University of Science and Technology (KNUST)<br>Breiman, Robert; Emory University , Global Health Institute<br>Cruz Espinosa, Ligia Maria; International Vaccine Institute, Epidemiology<br>Im, Justin; International Vaccine Institute, Epidemiology<br>Jang, Geun-Hyeog; International Vaccine Institute, Epidemiology<br>Jeon, Hyonjin; International Vaccine Institute, Epidemiology<br>Luby, Stephen; International Centre for Diarrhoeal Disease Research, Centre for Communicable Diseases; Stanford University,<br>Lunguya-Metila, Octavie; Institut National de Recherche Biomedicales; Service de Microbiologie, Cliniques Universitaires de Kinshasa<br>MacWright, William; Global Health Institute, Emory University<br>Mogeni, Ondari; International Vaccine Institute, Epidemiology<br>Okeke, Iruka; University of Ibadan<br>Owusu-Dabo, Ellis; Kumasi Center for Collaborative Research in Tropical Medicine; Kwame Nkrumah University of Science and Technology<br>Park, Jin Kyung; International Vaccine Institute<br>Park, SeEun; International Vaccine Institute, Epidemiology<br>Popoola, Femi; University of Ibadan<br>Seo, Hye-jin; International Vaccine Institute, Epidemiology<br>Soura, Abdramane ; Institut Superieur des Sciences de la Population, University of Ouagadougou<br>Teferi, Mekonnen; Armauer Hansen Research Institute, ALERT Campus<br>Toy, Trevor; International Vaccine Institute, Epidemiology<br>Chon, Yun; International Vaccine Institute<br>Rahaniminina, Lydia; Universite d'Antananarivo<br>Rakotozandrindrainy, Raphael; Universite d'Antananarivo<br>Meyer, Christian; Institute of Tropical Medicine, Eberhard Karls University; Faculty of Medicine, Duy Tan University<br>Marks, Florian ; International Vaccine Institute; The Department of Medicine, The University of Cambridge<br>Panzner, Ursula; International Vaccine Institute, Epidemiology

Keywords:	health/hygiene behavior, sanitation, socio-economic, geospatial random selection, sub-Saharan Africa, HPAfrica study

SCHOLARONE™ Manuscripts

Assessment of health behavior and other population-based factors: a standardized study in multiple cohorts in sub-Saharan Africa

Short Title

The **H**ealth **P**opulation **Africa** (HPAfrica) study

Authors

Gi-Deok Pak[1#], Andrea Haselbeck[1#], Hyeong-Won Seo[1], Isaac Osei[2], John Amuasi[2], Robert Breiman[3], Ligia Maria Cruz Espinoza[1], Justin Im[1], Geun-Hyeog Jang[1], Hyon-Jin Jeon[1], Stephen Luby[4], Octavie Lunguya-Metila[5,6], William MacWright[3], Ondari D. Mogeni[1], Iruka N. Okeke[7], Ellis Owusu-Dabo[1,2], Jin-Kyung Park[1], Se Eun Park[1], Oluwafemi Popoola[7], Hye-Jin Seo[1], Abdramane Bassiahi Soura[8], Mekonnen Teferi[9], Trevor Toy[1], Yun Chon[1], Lydia Beithine Rahaniminina[10], Raphael Rakotozandrindrainy[10], Christian G. Meyer[11,12], Florian Marks[1,13*] and Ursula Panzner[1]

Affiliations

1. International Vaccine Institute, Seoul, Republic of Korea
2. Kumasi Centre for Collaborative Research in Tropical Medicine, Kwame Nkrumah University of Science and Technology (KNUST), Kumasi, Ghana
3. Global Health Institute, Emory University, Atlanta, United States
4. Infectious Diseases and Geographic Medicine, Stanford University, Stanford, United States
5. Institut National de Recherche Biom ́edicales, Kinshasa, Democratic Republic of Congo
6. Service de Microbiologie, Cliniques Universitaires de Kinshasa, Kinshasa, Democratic Republic of the Congo
7. University of Ibadan, Ibadan, Nigeria
8. Institut Supérieur des Sciences de la Population, University of Ouagadougou, Ouagadougou, Burkina Faso
9. Armauer Hansen Research Institute, Jimma Road, ALERT Campus, Addis Ababa, Ethiopia
10. University of Antananarivo, Antananarivo, Madagascar
11. Institute of Tropical Medicine, Eberhard Karls University, Tübingen, Germany
12. Faculty of Medicine, Duy Tan University, Da Nang, Vietnam
13. The Department of Medicine, The University of Cambridge, Cambridge, United Kingdom

# Contributed equally

*Corresponding Author:

Florian Marks, MPH, PhD, International Vaccine Institute, SNU Research Park, 1 Gwanak-ro, Gwanak-gu, Seoul, 08826, Republic of Korea, Phone: +82-2-881-1133, Fax: +82-2-881-1164, E-mail: fmarks@ivi.int

For peer review only

For peer review only - http://bmjopen.bmj.com/site/about/guidelines.xhtml

Abstract

Introduction

The objective of the **H**ealth **P**opulation **Africa** (HPAfrica) study is to determine health behavior and other population-based factors, including socio-economic, ethnographic, hygiene and sanitation factors, at sites of the Severe Typhoid Fever in Africa (SETA) program. SETA aims to investigate healthcare facility-based fever surveillance in Burkina Faso, the Democratic Republic of the Congo (DRC), Ethiopia, Ghana, Madagascar, and Nigeria. Meaningful disease burden estimates require adjustment for health behavior patterns, which are assumed to vary among a study population.

Methods and Analysis

For the minimum sample size required, the assumptions of an infinite population, a design effect, and age- and sex-stratification are considered. In the absence of a sampling frame or household list, a geospatial approach will be used to generate geographic random points with an Aeronautical Reconnaissance Coverage Geographic Information System (ArcGIS) tool. Printouts of Google Earth Pro® satellite imagery visualize these points. Data of interest will be assessed in different seasons by applying simple, weighted-stratified sampling. An Android-based application and a web service have been developed for electronic data capturing and synchronization with the database server in real time. Descriptive data analyses will be performed in order to assess baseline information of each study population and age- and sex-stratified health behavior. This will allow adjusting disease burden estimates. In addition, multivariate analyses will be applied to look into associations between health behavior, population-based factors, and the disease burden as determined in the SETA study.

Ethics and Dissemination

This protocol was first approved by the Institutional Review Board of the International Vaccine Institute in October 2016 and undergoes annual review and renewal also by Ethics Committees of collaborating institutions (section Ethics References). All participants are required to provide written

For peer review only - http://bmjopen.bmj.com/site/about/guidelines.xhtml

informed consent. Findings from the HPAfrica study will be disseminated through publications in peer-reviewed journals and collaborating institutions.

Expected Challenges

- Standardization in community-based multi-country/-site research requires cautiousness considering variations in setting, language and culture.
- Current population data in study sites and their administratively-defined subareas are assumed to be limited.
- Accessibility to households may be complicated by environmental factors.
- Electronic data collection may cause technical problems related to damage, functional failure, or loss of devices.
- Unstable wireless internet connection may limit data teams to update the Android-based application, communicate with study managers in real time, and synchronize collected data with the server.

Registration: Not required

Ethics References

- IVI Institutional Review Board (IRB), No. 2016-003
- Institute of Tropical Medicine Antwerp Institutional Review Board, Belgium
- Universiteit Antwerpen, Comite voor medische Ethiek, Belgium
- Ministère de la Santé du Burkina Faso – Comité d'Ethique pour la Recherche en Santé, Burkina Faso
- Comité d'Ethique de l'Ecole de Santé Publique de l'Université de Kinshasa, Democratic Republic of Congo
- National Research Ethics Review committee (NRERC) Ministry of Science and Technology, Federal Democratic Republic of Ethiopia

For peer review only - http://bmjopen.bmj.com/site/about/guidelines.xhtml

- AHRI-ALERT Ethics Review Committee (AAERC), Ethiopia
- Kwame Nkrumah University of Science and Technology, School of Medical Sciences/Komfo Anokye Teaching Hospital, Committee on Human Research, Publication and Ethics, Ghana
- Ministère de la Santé du Repoblikan'l Madagaskar – Comité d'Ethique
- University of Ibadan/University College Hospital Ethics Committee, Ibadan, Nigeria

Keywords: health/hygiene behavior, sanitation, socio-economic, geospatial random selection, sub-Saharan Africa, HPAfrica study

For peer review only - http://bmjopen.bmj.com/site/about/guidelines.xhtml

Introduction

Assessment of health behavior, including healthcare utilization, is an important determinant for the generation of accurate disease burden estimates among target populations. This is particularly critical for studies which utilize observational, healthcare facility-based surveillance designs as substantial variation in health behavior patterns strongly influences disease burden estimates (1, 2). The adjustment of disease burden calculations for variation in health behavior will increase the accuracy of assessing prevalences of diseases caused by bacterial, fungal, viral, and parasitic pathogens among populations under investigation. Furthermore, the influence of a large variety of population-based factors on both health behavior and disease burden has been recognized. This includes socioeconomic factors such as education, occupation, income, the availability of household assets (3, 4), ethnical/religious and cultural factors (5, 6), but also hygiene and sanitation facilities (7-11), food handling (12-14), and animal contacts (10).

The **H**ealth **P**opulation **Africa** (HPAfrica) study will be conducted in Burkina Faso, Democratic Republic of the Congo (DRC), Ethiopia, Ghana, Madagascar, and Nigeria. Experiences from the Typhoid Fever Surveillance in Africa Program (TSAP) will be transferred to the Severe Typhoid in Africa (SETA) program (15, 16). In this program, standardized, healthcare facility-based fever surveillance is performed at selected study sites in participating countries over a two-years period (17). SETA primarily aims to systematically collect information on the burden of severe *Salmonella* infections. Subjects living in defined catchment areas with either an acute fever episode, a fever history of ≥3 consecutive days, clinically-suspected typhoid fever or clinically-diagnosed gastrointestinal perforations due to typhoid fever will be eligible. In addition, the severity of *Salmonella* caused illnesses and the natural history of *Salmonella* infection will be recorded with further analyses on host immune responses and chronic carriage.

Objectives and Outcomes

The goal of the HPAfrica study is to assess the *generic* and *actual* age- and sex-stratified health

For peer review only - http://bmjopen.bmj.com/site/about/guidelines.xhtml

behavior. *Generic* health behavior pertains to behavior in conditions associated with fever and other signs and symptoms unrelated to onsets, whereas *actual* health behavior is related to onsets (Appendix 1/Supplementary file 1; Appendix 2/Supplementary file 2: Form 4 Part A, Form 5; Appendix 3/Supplementary file 3). Data will be used to adjust age- and sex-stratified incidences of infectious pathogens in the proportion of a study population not captured by activities of HPAfrica. Data documented for various signs and symptoms or a combination of these will be used for a gradual classification of healthcare utilization based on disease severity.

The HPAfrica study will, moreover, collect data on healthcare-associated factors such as travel modalities, possession of a health insurance, cultural/ethnographic factors, immunization status among children (18, 19), and the frequency of occurrence and individual perception of selected diseases (Appendix 2/Supplementary file 2: Form 4 Part B; Appendix 3/Supplementary file 3:Form 4 Part B). Demographic data (Appendix 2/ Supplementary file 2: Form 3 Part A; Appendix 3/Supplementary file 3, Form 3 Part A) will be used for age- and sex-stratification of the population surveyed, which is required to compute adjustment factors for disease incidences. Socio-economic data (Appendix 2/ Supplementary file 2: Form 3 Part B; Appendix 3/ Supplementary file 3: Form 3 Part B) will be utilized to calculate wealth indices for descriptive and analytical approaches of the populations studied. Information on hygiene and sanitation facilities (Appendix 2/ Supplementary file 2: Form 3 Part C; Appendix 3/Supplementary file 3: Form 3 Part C) may provide a better understanding of the occurrence and frequency of pathogens identified in the populations studied.

Methods

Study sites

The boundaries of each site will be defined using pre-existing information set by statistical authorities or ministries of health (Table 1). A retrospective review of records of recruitment healthcare facilities from the past two years will support the re-definement of site limits. It is expected that this review will reveal the residences or at least the broader administrative residential areas such as communities,

For peer review only - http://bmjopen.bmj.com/site/about/guidelines.xhtml

154 districts or villages of patients who sought healthcare for any reason. Maps and satellite imagery may be used to better visualize patients' residences and the overall boundaries, including the geographically or administratively defined subareas, of each site. Where boundaries cannot be clearly defined, additional factors such as the distance to a recruitment healthcare facility may be considered.

For peer review only

For peer review only - http://bmjopen.bmj.com/site/about/guidelines.xhtml

Table 1: Background data of participating study sites of target countries

Country	Study site	Setting*	Approximate total site population (year)	Approximate age-stratified site population [year]				Approximate sex-stratified site population [year]	
				<2	≥2 to <5	≥5 to <15	≥15	Male	Female
Burkina Faso	Nioko II[1]	Urban	19,251 (2017)	2,394	1,977	4,657	10,223	9,321	9,930
	Polesgo[1]	Rural	7,897 (2017)	934	893	1,808	4,262	3,856	4,041
	Ouagadougou[1]	Urban	2,532,311 (2015)	421,429		693,723	1,417,159	1,271,302	1,261,009
DRC	Kisantu[2]	Urban	291,252 (2017)	48,043		243,209		91,598#	99,231#
Ethiopia	Wolayita Zone[3]	Urban/ Rural	1,968,735 (2017)	100,800	206,520	635,015	1,026300	964,577	1,004,058
	Wolayita Sodo[3]	Urban/ Rural	120,288 (2017)	6,161	12,617	38,807	62,703	59,898	60,390
	East Shewa & Arsi Zone[3]	Urban/ Rural	3,249,722	104,408	422,939	1,016,917	1,705,458	1,671,699	1,578,023
	Adama Wenji[3]	Urban/ Rural	53,540 (2017)	3,330	7,204	17,028	25,978	27,199	26,341
Ghana	Asante Akim North & Central[4]	Urban/ Rural	140,694 (2010)	11,606	8,363	35,618	85,107	67,673	73,021
	Kumasi (Metropolis)[4,5]	Urban	1,730,249 (2010)	52,516	178,575	421,834	1,077,324	826,479	903,770
Madagascar	Antananarivo[6] (Renivohitra)	Urban	1,247,025 (2009)	N/A$	N/A$	N/A$	N/A$	N/A$	N/A$
	Imerintsiatosika[7]	Rural	44,669 (2016)	3,582	4,449	7,610	29,028	N/A$	N/A$
Nigeria	Ibadan[8]	Urban	1,343,147 (2006)	176,110		305,656	861,381	661,818	681,329

Note. Table 1 shows population data that were available at the time of the HPAfrica protocol writing; population data and boundaries of geographically and/or administratively defined study sites may be subject to changes during the course of the study. *The classification of sites by country is based on best local knowledge; #The sex-stratification is based on a total population of 190,829; $Population data not available at the time of protocol writing.

Source of population data: **Burkina Faso** ([1]http://www.insd.bf/n/; [2]http://www.indepth-network.org/member-centres/ouagadougou-hdss); **DRC** ([2]Kisantu Central Health Zone Office report, 2016); **Ethiopia** ([3]Health Management Information System of the Ethiopian Ministry of Health (zonal and district health offices); **Ghana** ([4]Ghana Statistical Service, 2010 Population & Housing Census, Asante Akim Central Municipality; [5]Ghana Statistical Service, 2010 Population & Housing Census, Summary report of final results); **Madagascar** ([6]Population par Fokotany selon la declaration des Chefs Fokotany: Source: Donnee de la cartographie censitaire mises-a-jour en juillet 2009 - INSTAT/DDSS; [7]University of Antananarivo); **Nigeria** ([8]Federal Republic of Nigeria 2006 Population and Housing Census (Table DS5), National Population Commission, Abuja, Nigeria

For peer review only

For peer review only - http://bmjopen.bmj.com/site/about/guidelines.xhtml

Sample size

All available sources will be used to gather most up-to-date age- and sex-stratified population data by study site and its administratively or geographically defined subareas. This may include latest demographic information from a population census or a Demographic Surveillance System (DSS)/Health and Demographic Surveillance System (HDSS). Population summary figures and population growth factors, if available, may also be used.

The minimum number of randomly selected households to be interviewed will be calculated using the precision-based equation (Equation-I) (20-23) assuming an infinite population and considering a design effect (DEFF), including its accompanying assumptions:

Equation-I: precision-based sample size calculation for an infinite population (20-23):

$$n_0 = DEFF \cdot \left[z^2{}_{1-\alpha/2} \cdot \frac{4 \cdot p \cdot (1-p)}{d^2} \right]$$

<u>Assumptions Equation-I</u>

n_0	Minimum number of households to be interviewed in a study area assuming an infinite population
DEFF	Design effect; set at 1.5
$z_{1-\alpha/2}$	Normal deviation corresponding to a 95% confidence interval (1.96 for alpha of 0.05)
d	Precision (acceptable error); point estimation; set at 0.2
p	Proportion of the study population expected to visit a recruitment healthcare facility for conditions associated with fever and other signs and symptoms (proportion captured); set at 0.2
1-p	Proportion of the study population expected to not visit a recruitment healthcare facility for conditions associated with fever and other signs and symptoms (proportion not captured)

The design effect is an adjustment factor for natural clustering of health behavior among household members (21). It is arbitrarily set at 1.5, depending on the household size, as no exact estimates are available (24, 25). A proportion p of 0.2 may be assumed if no other estimates are available or p may be based on experiences during the TSAP study: Madagascar, Isotry: p=0.01, Burkina Faso, Polesgo: p=0.9 (26). Combining all assumptions into Equation-I will result in a total minimum number (n_0) of 92 household interviews at each site.

In addition, n_0 will be accounted for the age- and sex-distribution of a study population reflected by $\hat{p}$,

For peer review only - http://bmjopen.bmj.com/site/about/guidelines.xhtml

the assumed minimum proportion of stratification per age group (<5 years, ≥5 to <15 years, ≥15 years). Factor $\hat{p}$ may be arbitrarily set at 0.2 or may be based on previous experiences: Ethiopia, Butajira: $\hat{p}$=0.09, Burkina Faso, Polesgo: $\hat{p}$=0.23 (26). Applying $\hat{p}$ to Equation-I for a binomial distribution, including its accompanying assumptions, will result in the age-stratified minimum number of household interviews ($\overline{n_0}$) (Equation-II):

Equation-II: precision-based sample size calculation for an infinite population considering age-stratification:

$$\overline{n_0} = \left(DEFF \cdot \left[z^2_{1-\frac{\alpha}{2}} \cdot \frac{4 \cdot p \cdot (1-p)}{d^2}\right]\right) / \hat{p}$$

<u>Assumptions Equation-II</u>

$\overline{n_0}$	Minimum number of households to be interviewed in the study area assuming an infinite population and age-stratification
DEFF	Design effect; set at 1.5
$z_{1-\alpha/2}$	Normal deviation corresponding to a 95% confidence interval (1.96 for alpha of 0.05)
D	Precision (acceptable error); point estimation; set at 0.2
P	Proportion of the study population expected to visit a recruitment healthcare facility for conditions associated with fever and other signs and symptoms (proportion captured); set at 0.2
1-p	Proportion of the study population expected to not visit a recruitment healthcare facility for conditions associated with fever and other signs and symptoms (proportion not captured)
$\hat{p}$	The minimum proportion of stratification per age group; set at 0.2

Health behavior will be assessed twice at the same households if possible or at alternative households in case the study population is instable due to migration and/or high birth/death rates. Additional households, assumed to be 20% on average, (Table 2) may be added to account for possible loss to follow-up between the two assessments. Eventually, $\overline{n_0}$ will be divided by the number of subareas and weighted proportionally to the population size by applying weighted-stratified sampling [16].

Table 2: Sample sizes considering infinite population and age-stratification applying Equation-II:

Proportion (p)	Total minimum number of households without DEFF	Total minimum number of households (n_0) with DEFF=1.5	Total minimum number of households ($\overline{n_0}$) with DEFF=1.5 and $\hat{p}$=0.2	$\overline{n_0}$ adjusted for loss to follow-up
0.1	35	52	259	311
0.2	62	92	461	553
0.3	81	121	605	726
0.4	92	138	691	830
0.5	96	144	720	864

For peer review only

For peer review only - http://bmjopen.bmj.com/site/about/guidelines.xhtml

0.6	92	138	691	830
0.7	81	121	605	726
0.8	62	92	461	553
0.9	35	52	259	311

Abbreviations
p=Proportion of the study population expected to visit a recruitment healthcare facility for conditions associated with fever and other signs and symptoms (proportion captured); set at 0.2
DEFF=Design effect; set at 1.5
n_0=Total minimum number of households to be interviewed in a study area assuming an infinite population
$\overline{n}_0$=Total minimum number of households to be interviewed in the study area assuming an infinite population and age-stratification
$\hat{p}$=Minimum proportion of stratification per age group; set at 0.2

Sampling

If comprehensive up-to-date sampling frames or household lists exist (through DSS/HDSS or available census), computerized random selection will be done using SAS (Statistical Analysis System, version 9.4, SAS Institute, Cary, NC) applying serial simple random selection without replacement. If no sampling frame exists, it can be constructed using satellite imagery or aerial maps in order to enumerate all structures (26), rastering (27), or grid cells (28, 29).

We apply a geospatial technique for the creation of points generated by the ArcGIS (Aeronautical Reconnaissance Coverage Geographic Information System; Redlands, CA, USA, version 10.2) random point generation tool with the application of simple, weighted-stratified sampling (30, 31). The tool randomly places specified numbers of points inside the features of a polygon that corresponds to an administrative subarea. X- and Y-coordinates define one point until the calculated sample size is reached. ArcGIS-generated random points are converted into an image file of the Keyhole Markup Language (KML) or Keyhole Markup language Zipped (KMZ) format and imported into Google Earth Pro® (Google Inc., Mountain View, CA, USA, version 6.2.2.6613). A unique numerical identifier and horizontal geographic coordinates are assigned to each point. The location of each point is verified on an up-to-date Google Earth Pro® satellite imagery. Appendix 4/Supplementary file 4 illustrates our approach when utilizing examples from Ghana (Appendix 4/Supplementary file 5) and Madagascar (Appendix 4/Supplementary file 6).

For peer review only

For peer review only - http://bmjopen.bmj.com/site/about/guidelines.xhtml

Geographic points labeled with identifiers are pictured on poster-size (60x60 to 60x90 cm) printouts of Google Earth Pro® satellite imagery with high resolution up to the structure level. The satellite maps help to locate selected points on the ground. Global Positioning System (GPS) receivers (Garmin-eTrex; Garmin Ltd., Lenexa, KS, USA) allow to verify the locations. The receivers will be positioned closely to a located point, in a static position and an open area to assure barrier-free reading. In case of refusal, absence of the respondent or locating of a non-residential point, a residential point closest to the right or left side will be visited instead (32).

Frequency of data collection

Residential points will be visited twice for household interviews during the SETA program to assess seasonal influences on health behavior (33-37). Interviews will take place in differing seasons – one at the end of the dry season or at least one month after its beginning and one towards at end of the wet season or at least one month after its beginning (Table 3). Currently, the HPAfrica study is ongoing in Burkina Faso, Ghana and Madagascar and is anticipated to be continued in the remaining countries during the upcoming months.

Table 3: Seasonality in participating countries

Country	Period of Wet Season	Period of Dry Season
Burkina Faso	May/June - September/October	October/November - April/May
DRC	November - March	April - October
Ethiopia	June - August	October - May
Ghana	April – July, September - November	December - March
Madagascar	November - March/April	April/May - October
Nigeria	May - August	October - April

Note. The seasonality by country is based on best local knowledge.

Inclusion and exclusion criteria

Household members of all ages and both sexes living in a study area at the day of the interview will be eligible for inclusion. A household will be excluded if the designated respondent declines participation or is unavailable after three consecutive visits. All visitors and individuals with unknown

For peer review only - http://bmjopen.bmj.com/site/about/guidelines.xhtml

residence or residence outside the study area will be excluded.

For the purpose of this investigation, a household is defined as a person or a group of related or unrelated persons living in the same dwelling unit, acknowledging one adult individual as the household head, sharing the same housekeeping arrangements, and independently procuring food and other essential for living (26).

The interview will exclusively be held with the respondent, who is an adult household member at the country-specific legal age of majority. This person may be identified as decision-maker by members of the same household and serves as a proxy for an entire household (15). Further relevant definitions are explained in Appendix 1/Supplementary file 1 and Appendix 3/Supplementary file 3.

Data collection

On-site interviewers will be trained on locating the geographic points, identification of respondents, informed consent procedures with emphasis on voluntary participation, and the deployment of standardized, pre-tested study forms (Appendix 2/Supplementary file 2 and Appendix 3/Supplementary file 3) prior to the initiation of HPAfrica. Informed consent and study forms will be translated into the country-specific official language and back-translated into English to account for translation errors and to make corrections, where required.

Data management

Management of data will depend on the mode of collection, which will be primarily electronic rather than paper-based. Past experiences showed following disadvantages of paper-based data collection: a high probability of errors while filling study forms, and the need of subsequent data digitalization, which is an extra, labor- and time-intensive and error-prone step. Electronic data capturing using a smartphone or tablet application minimizes these limiting factors to the data quality.

For peer review only - http://bmjopen.bmj.com/site/about/guidelines.xhtml

There may be a pilot period during which both paper-based and paperless data will be collected prior to full implementation of electronic data collection. The latter utilizes the Android application "HPA Collect" (Google Android 5.0.1 9API 23; available at https://play.google.com/store/apps/details?id=anint.ivi.hpa) and the platform "HPA Web" (CSS, JavaScript and JSP; available at http://hpa.ivi.int/), which are both accompanied by a variable dictionary containing codes, meaning and properties of variables in at least English and French. Stable wireless internet connectivity using default browsers is required to assure a contemporaneous, attributable, original and accurate synchronization of data between "HPA Collect" and "HPA Web". Access to both "HPA Collect" and "HPA Web" is password-protected. Appendix 5/Supplementary file 7 displays the system diagram, which has the following features:

Structure or features of "HPA Collect"

- **User management:** A simple user interface provides different privileges for users to enter, edit (except for study labels), and export or transfer data. It establishes an audit trail that records when users enter data into the server.
- **Data input:** Data are entered directly into the smartphone or tablet device. Entry fields limit possible answers to plausible ones only by offering different input methods:
    - Edit Text: free text
    - Radio group/Multi click button: single choice
    - Check box: multiple choice
    - Date and time picker: entering date and time
    - Spinner: drop-down button

  Additional features allow the surveyor to save time by transferring data collected at an earlier stage to data fields required at a later stage of the interview by performing plausibility checks for diverse data input fields, and by easily recognizing skip patterns. Error checks, missing data notifications, code errors, and logical errors are also included.

For peer review only - http://bmjopen.bmj.com/site/about/guidelines.xhtml

- **Data view:** Raw, originally entered data can be seen in the same, human- and machine-readable format, namely the JSON format, as they will be transferred to a database server (Windows, MySQL). Additional functions like "clear" to clear all records, "reset" to return back to the main data entry page, and "search" to search for a study label are available.
- **Settings:** The ability to switch the system language between English and French is provided.
- **About:** The currently installed version of "HPA Collect" is displayed.

Structure or features of "HPA Web"

- **Home:** An introduction to the platform is given and a tutorial in French and English will be accessible.
- **Form View:** The study forms are displayed separately showing every question and the corresponding input data as defined variables.
- **Form Edit:** This feature allows the correction of the uploaded data. It is only accessible to data managers in each participating country and at IVI.
- **Search:** Specific study labels given to each study form can be filtered.
- **Variable dictionary:** The variable dictionary can be downloaded.
- **Export:** Data can be converted from the server into a transferrable excel file.
- **Contact Us:** Quick and easy correspondence between users and the data manager at IVI is given.

Data will be reviewed by key study personnel on a day-to-day basis and checked for consistency and accuracy prior to data analysis. The quality of data may be checked by selecting a subset of 5% for validation against the original, paper-based data if possible. Names of household members will not be linked to study forms of HPAfrica and will not be recorded in the database in order to ensure confidentiality. Access to the database will be restricted to authorized study personnel only and data

For peer review only - http://bmjopen.bmj.com/site/about/guidelines.xhtml

will be kept in a locked, protected location. Periodic interim backup files and at least three final backup files of the database stored in a secure, locked place will avoid loss of data and ensure data safety. All data will be stored for a minimum of five years.

Data analysis

Descriptive analyses such as absolute and relative frequencies, medians or arithmetic means will be conducted to construct a contemporary baseline population description.

Basic analyses will include the calculation of age- and sex-stratified proportions of *generic* and *actual* health behavior, including healthcare utilization, for various signs and symptoms or combinations of signs and symptoms. These analyses will allow for a gradual classification based on disease severity, including 95% confidence interval. Health behavior will be adjusted for an assumed natural clustering effect among household members. Most up-to-date stratified population data by site and demographic data derived from HPAfrica will be used to generate the proportions for the adjustment of incidence calculations.

Socio-economic information collected will be used to construct wealth indices of studied populations by applying factor analyses based on the principal component method. In addition, bi- and multivariate regression analyses will be performed to identify potential confounding factors or effect modification using factor scores.

Bi- and multivariate regression analyses will be carried out using factor scores for the assessment of the following variables:

- Socioeconomic data such as education, income (38), health insurance, housing, and household assets
- Hygiene and sanitation such as toilet access, water sources, food handling, waste disposal, and animal contacts

For peer review only - http://bmjopen.bmj.com/site/about/guidelines.xhtml

- Health behavior: *actual* (age- and sex-stratified, severity, frequency), *generic* (age- and sex-stratified, severity, rating by choice)
- Travel modalities to health facilities, age-stratified
- Seasonality related to hygiene & sanitation, health behavior, travel modalities
- Cultural/religious/ethnographic factors, age-stratified
- Vaccination status among children (18, 19)
- Knowledge and perception of common diseases

Ethical considerations

There are no direct benefits for enrolled households and their members. Indirect benefits for the general population at sites will be the updated information on the burden of communicable diseases, including factors influencing disease transmission and infection. The HPAfrica study generates evidence to support decision-makers on the introduction of appropriate measures for disease prevention and control.

This protocol underwent reviews by IVI's Institutional Review Board and site-specific local boards as shown in the section "Ethics References". During and after the HPAfrica study, all data of enrolled subjects will be kept in strict confidence and will not be disclosed to a third party by any member of the research team. Password protection of devices and the database is used for strict confidence. All paper-based data (consent forms) will be kept in a secure, locked location. Confidential information stored on computers and paper-based data sources will only be made available to co-investigators and IVI staff directly involved in study activities of HPAfrica.

Dissemination

We plan to disseminate the study findings by reporting descriptive as well as analytic and stratified data through publications in peer-reviewed journals and collaborating institutions.

For peer review only - http://bmjopen.bmj.com/site/about/guidelines.xhtml

Authors' contributions

GDP, JKP and UP conceptualized the initial study concept of the HPAfrica study. HWS and GHJ developed the tools "HPA Collect" and "HPA Web" for electronic data collection with primary support of UP and AH. IO and JA supported the development and finalizing of the data collection tools "HPA Collect" and "HPA Web". LBR and RR supported the development and finalization of the HPAfrica study forms. The appendices were conceptualized by GDP, JKP, YC, JI, TT, AH and UP. IO, JA, RB, LMCE, JI, HJJ, SL, OLM, WMW, ODM, INO, EOD, SP, OP, HJS, ABS, MT, TT, LBR and RR supported the further development of the initial study protocol and appendices. FM, YC and CGM participated in the finalization of the study protocol, including appendices. AH wrote the first draft of the manuscript. GDP, HWS, IO, JA, RB, LMCE, JI, GHJ, HJJ, SL, OLM, WMW, ODM, INO, EOD, JKP, SP, OP, HJS, ABS, MT, TT, YC, LBR, RR, CGM and FM critically reviewed and revised the manuscript draft. GDP, AH, HWS, IO, JA, RB, LMCE, JI, GHJ, HJJ, SL, OLM, WMW, ODM, INO, EOD, JKP, SP, OP, HJS, ABS, MT, TT, YC, LBR, RR, CGM, FM and UP agreed and approved the final manuscript as submitted.

Acknowledgement

We thank David Kauderer for the support and advice in computer engineering, the support in proofreading French study materials from Dr. Paul M. Tshiminyi, and the administrative support from Soo Young Kwon and Ji Hyun Han.

Funding statement

This study was supported by the Bill & Melinda Gates Foundation (OPP1127988). The funders had no role in study design, data collection, data analysis, data interpretation, or writing of the report; the conclusions and findings presented are those of the authors and do not necessarily reflect positions or policies of the Bill & Melinda Gates Foundation or the International Vaccine Institute (IVI). The IVI acknowledges its donors, including the Republic of Korea and the Swedish International Development Cooperation Agency (Sida).

For peer review only - http://bmjopen.bmj.com/site/about/guidelines.xhtml

Competing interests

The authors declare that they have no competing interests.

Abbreviations

JSON: JavaScript Objective Notation: CSS: Cascading Style Sheets: JSP: Java Server Page; SQL: Structured Query Language

Appendices

Appendix1 (Supplementary file 1)

Definitions/Terminology (English)

Appendix 2 (Supplementary file 2)

Study Forms (English)

Form 1: Record of household visits

Form 2: Consent Form

Form 3: General household information

Part A: Demographic data/General household information

Part B: Socio-economic data

Part C: Hygiene and sanitation

Form 4: Assessment of age- and sex-stratified *generic* healthcare utilization

Part A: Assessment of *generic* healthcare utilization

Part B: Healthcare-associated factors/Vaccination/Disease perception

Form 5: Assessment of age- and sex-stratified *actual* healthcare utilization

Appendix 3 (Supplementary file 3)

Protocol, Definitions/Terminology and Study Forms (French)

Appendix 4 (Supplementary file 4)

Geospatial random selection (examples of Ghana (Supplementary file 5) and Madagascar

For peer review only - http://bmjopen.bmj.com/site/about/guidelines.xhtml

(Supplementary file 6))

Appendix 5 (Supplementary file 7)

System diagram of the data collection and data management: the Android application “HPA Collect” and the platform “HPA Web”.

References

1. Bigogo G, Audi A, Aura B, Aol G, Breiman RF, Feikin DR. Health-seeking patterns among participants of population-based morbidity surveillance in rural western Kenya: implications for calculating disease rates. Int J Infect Dis. 2010;14(11):e967-73.
2. Jordan HT, Prapasiri P, Areerat P, Anand S, Clague B, Sutthirattana S, et al. A comparison of population-based pneumonia surveillance and health-seeking behavior in two provinces in rural Thailand. Int J Infect Dis. 2009;13(3):355-61.
3. Saeed BI, Xicang Z, Yawson AE, Nguah SB, Nsowah-Nuamah NN. Impact of socioeconomic status and medical conditions on health and healthcare utilization among aging Ghanaians. BMC public health. 2015;15:276.
4. Thoa NT, Thanh NX, Chuc NT, Lindholm L. The impact of economic growth on health care utilization: a longitudinal study in rural Vietnam. International journal for equity in health. 2013;12:19.
5. Okafor IP, Sekoni AO, Ezeiru SS, Ugboaja JO, Inem V. Orthodox versus unorthodox care: A qualitative study on where rural women seek healthcare during pregnancy and childbirth in Southwest, Nigeria. Malawi medical journal : the journal of Medical Association of Malawi. 2014;26(2):45-9.
6. Farag TH, Kotloff KL, Levine MM, Onwuchekwa U, Van Eijk AM, Doh S, et al. Seeking care for pediatric diarrheal illness from traditional healers in Bamako, Mali. The American journal of tropical medicine and hygiene. 2013;89(1 Suppl):21-8.
7. Akullian A, Ng'eno E, Matheson AI, Cosmas L, Macharia D, Fields B, et al. Environmental Transmission of Typhoid Fever in an Urban Slum. PLoS Negl Trop Dis. 2015;9(12):e0004212.
8. Steele AD, Hay Burgess DC, Diaz Z, Carey ME, Zaidi AK. Challenges and Opportunities for Typhoid Fever Control: A Call for Coordinated Action. Clinical infectious diseases : an official publication of the Infectious Diseases Society of America. 2016;62 Suppl 1:S4-8.
9. Tran HH, Bjune G, Nguyen BM, Rottingen JA, Grais RF, Guerin PJ. Risk factors associated with typhoid fever in Son La province, northern Vietnam. Transactions of the Royal Society of Tropical Medicine and Hygiene. 2005;99(11):819-26.
10. Rweyemamu M, Kambarage D, Karimuribo E, Wambura P, Matee M, Kayembe JM, et al. Development of a One Health National Capacity in Africa : the Southern African Centre for Infectious Disease Surveillance (SACIDS) One Health Virtual Centre Model. Current topics in microbiology and immunology. 2013;366:73-91.
11. Slayton RB, Date KA, Mintz ED. Vaccination for typhoid fever in sub-Saharan Africa. Human vaccines & immunotherapeutics. 2013;9(4):903-6.
12. Sharma PK, Ramakrishnan R, Hutin Y, Manickam P, Gupte MD. Risk factors for typhoid in Darjeeling, West Bengal, India: evidence for practical action. Tropical medicine & international health : TM & IH. 2009;14(6):696-702.
13. Eguale T, Gebreyes WA, Asrat D, Alemayehu H, Gunn JS, Engidawork E. Non-typhoidal Salmonella serotypes, antimicrobial resistance and co-infection with parasites among patients with diarrhea and other gastrointestinal complaints in Addis Ababa, Ethiopia. BMC infectious diseases. 2015;15:497.

For peer review only - http://bmjopen.bmj.com/site/about/guidelines.xhtml

14. Paudyal N, Anihouvi V, Hounhouigan J, Matsheka MI, Sekwati-Monang B, Amoa-Awua W, et al. Prevalence of foodborne pathogens in food from selected African countries - A meta-analysis. International journal of food microbiology. 2017;249:35-43.
15. Panzner U, Pak GD, Aaby P, Adu-Sarkodie Y, Ali M, Aseffa A, et al. Utilization of Healthcare in the Typhoid Fever Surveillance in Africa Program. Clinical infectious diseases : an official publication of the Infectious Diseases Society of America. 2016;62 Suppl 1:S56-68.
16. von Kalckreuth V, Konings F, Aaby P, Adu-Sarkodie Y, Ali M, Aseffa A, et al. The Typhoid Fever Surveillance in Africa Program (TSAP): Clinical, Diagnostic, and Epidemiological Methodologies. Clinical infectious diseases : an official publication of the Infectious Diseases Society of America. 2016;62 Suppl 1:S9-S16.
17. Park SE, Toy T, Adu-Sarkodie Y, Baker S, Breiman RF, Cruz Espinoza LM, et al. The Severe Typhoid in Africa Program: a multicentre population-based surveillance program with a nested case-control and cohort study. under submission.
18. WHO. WHO vaccine-preventable diseases: monitoring system. 2017 global summary 2017 [Available from: http://apps.who.int/immunization_monitoring/globalsummary/schedules/.
19. WHO recommendations for routine immunization - summary tables [Internet]. 2017 [cited 7 December 2017]. Available from: http://www.who.int/immunization/policy/immunization_tables/en/.
20. Fleiss JL, Levin, B., Paik, M.C. Statistical Methods for Rates and Proportions. 3rd ed. New York: John. Wiley & Sons; 2003.
21. Henderson RH, Sundaresan T. Cluster sampling to assess immunization coverage: a review of experience with a simplified sampling method. Bulletin of the World Health Organization. 1982;60(2):253-60.
22. Henderson RH, Davis H, Eddins DL, Foege WH. Assessment of vaccination coverage, vaccination scar rates, and smallpox scarring in five areas of West Africa. Bulletin of the World Health Organization. 1973;48(2):183-94.
23. Newcombe RG. Two-sided confidence intervals for the single proportion: comparison of seven methods. Statistics in medicine. 1998;17(8):857-72.
24. Katz J, Zeger SL. Estimation of design effects in cluster surveys. Ann Epidemiol. 1994;4(4):295-301.
25. Bostoen K, Chalabi Z. Optimization of household survey sampling without sample frames. Int J Epidemiol. 2006;35(3):751-5.
26. Panzner U, Ali M, Meyer CG, Baker S, Deerin JF, Eltayeb MA, et al. Geospatial sampling frames for household-based studies: Lessons learned from a multi-country surveillance network in Senegal, South Africa and Sudan. 2017 (submitted).
27. Lister AJ, Scott CT. Use of space-filling curves to select sample locations in natural resource monitoring studies. Environ Monit Assess. 2009;149(1-4):71-80.
28. Keating J, MacIntyre K, Mbogo C, Githeko A, Regens JL, Swalm C, et al. A geographic sampling strategy for studying relationships between human activity and malaria vectors in urban Africa. Am J Trop Med Hyg. 2003;68(3):357-65.
29. Keating J, Macintyre K, Mbogo CM, Githure JI, Beier JC. Characterization of potential larval habitats for Anopheles mosquitoes in relation to urban land-use in Malindi, Kenya. Int J Health Geogr. 2004;3(1):9.
30. Lohr SL. Sampling: Design and Analysis. 2nd edition ed. Pacific Grove, CA: Duxbury Press; 2010.
31. ArcGIS. [Available from: http://pro.arcgis.com/en/pro-app/tool-reference/data-management/create-random-points.htm.
32. Kondo MC, Bream KD, Barg FK, Branas CC. A random spatial sampling method in a rural developing nation. BMC public health. 2014;14:338.
33. Kasper MR, Blair PJ, Touch S, Sokhal B, Yasuda CY, Williams M, et al. Infectious etiologies of acute febrile illness among patients seeking health care in south-central Cambodia. The American journal of tropical medicine and hygiene. 2012;86(2):246-53.

For peer review only - http://bmjopen.bmj.com/site/about/guidelines.xhtml

553 34. Dewan AM, Corner R, Hashizume M, Ongee ET. Typhoid Fever and its association with environmental factors in the Dhaka Metropolitan Area of Bangladesh: a spatial and time-series approach. PLoS Negl Trop Dis. 2013;7(1):e1998.

556 35. Naeem Khan M, Shafee M, Hussain K, Samad A, Arif Awan M, Manan A, et al. Typhoid fever in paediatric patients in Quetta, Balochistan, Pakistan. Pakistan journal of medical sciences. 2013;29(4):929-32.

559 36. Crump JA, Youssef FG, Luby SP, Wasfy MO, Rangel JM, Taalat M, et al. Estimating the incidence of typhoid fever and other febrile illnesses in developing countries. Emerging infectious diseases. 2003;9(5):539-44.

562 37. Osei-Tutu B, Anto F. Trends of reported foodborne diseases at the Ridge Hospital, Accra, Ghana: a retrospective review of routine data from 2009-2013. BMC infectious diseases. 2016;16:139.

565 38. (ILO) ILO. International Standard Classification of Occupations ISCO-08, 2012 [Available from: http://www.ilo.org/wcmsp5/groups/public/---dgreports/---dcomm/---publ/documents/publication/wcms_172572.pdf.

For peer review only - http://bmjopen.bmj.com/site/about/guidelines.xhtml

Appendix 1/Supplementary file 1: Definitions/Terminology (English)

**A**	
Abdominal pain/ Intestinal pain	Pain (acute/short-lived or chronic/long-lived) that occurs anywhere between the chest and the groin (often referred to as the stomach region/belly).
Aches/Pain	A condition of having continuous/prolonged dull pain in a part of the body; the physical feeling, suffering, or discomfort caused by an illness or injury.
**B**	
Bleeding (internal/external)/ Blood loss	The condition of losing blood from the vascular system, either internally into the body (internal bleeding) or externally through a natural orifice or break in the skin (external bleeding); blood escaping from an injured blood vessel.
Blood loss/ Bleeding	The condition of losing blood from the vascular system, either internally into the body (internal bleeding) or externally through a natural orifice or break in the skin (external bleeding); blood escaping from an injured blood vessel.
Blood pressure disorders	A high/low blood pressure or a blood pressure that shows excessive fluctuation.
**C**	
Chills/Shivering	The sensation of coldness that could be accompanied by shivering or shaking of the body and pallor of the skin.
Cholera	An infectious, sometimes fatal disease of the small intestine caused by the bacterium *Vibrio cholerae*; it is spread via the fecal-oral route from the ingestion of contaminated water and food and causes chills, shivering, severe watery diarrhea, vomiting, and dehydration.
Confusion/Dizziness/ Unconsciousness	The state of being bewildered or unclear in mind; an abnormal state of response to stimuli in the environment; faint, wooziness, weakness, unsteadiness or a tendency to fall; the interruption of the awareness of oneself and one's surroundings.
Convulsion	The sudden, violent, irregular movement of a limb/part of the body or of the entire body which is caused by involuntary contraction of muscles.
Cough	The condition of expelling air from the lungs suddenly/rapidly and with a harsh noise; often involuntarily in order to clear the lung airways of fluids/mucus/other material.
**D**	
Dehydration	A condition of excessive loss of water/moisture from the body.
Diarrhea	A condition of passing feces from the body very frequently and in a liquid rather than solid form.
Diphtheria	A highly contagious, sometimes fatal disease caused by the bacterium *Corynebacterium diphtheriae*. It is spread from person-to-person, through the air or contaminated objects and affects the upper respiratory tract. Common symptoms are sore throat, swollen glands/ lymph nodes, malaise, fever/chills, a membranous substance/thick gray material that forms in the throat/tonsils and makes it difficult to breathe/ rapid breathing ("barking cough") and swallow. A form of diphtheria that involves the skin, eyes, or genitals is also reported and is characterized by pain, redness, and swelling of the skin.
Dizziness/Confusion/ Unconsciousness	The state of being bewildered or unclear in mind; an abnormal state of response to stimuli in the environment; faint, wooziness, weakness, unsteadiness or a tendency to fall; the interruption of the awareness of oneself and one's surroundings.
**E**	
Edema/Swelling	The excess collection of a watery fluid/swelling in the cavities or tissues of the body; it's commonly noticed in the hands/arms/feet/legs; volume increase of tissue.
Education - formal	A systematic, organized education model that is structured and administered according to a given set of laws and norms; formal education institutions are administratively, physically and curricularly organized and require a minimum classroom attendance from students; it involves intermediate and final assessments in order to advance to the next learning stage; it confers degrees and diplomas pursuant to a quite strict set of regulations.
**F**	
Fatigue/Malaise/	The state of feeling tired, exhausted, or weak; a general feeling of discomfort,

For peer review only - http://bmjopen.bmj.com/site/about/guidelines.xhtml

Weakness	uneasiness, unhealthiness, or unhappiness.
Fever, any	An abnormally high body temperature (usually above 38°C).
Fever <3 days	An abnormally high body temperature (usually above 38°C) that appears continuously for less than 3 days.
Fever >3 days	An abnormally high body temperature (usually above 38°C) that lasts longer than 3 days and appears continuously.
**G**	
**H**	
Headache	Any kind of pain in any region of the head.
Healthcare - actual	Healthcare that is related to specific sign/symptom-associated disease onsets or disease onset dates.
Healthcare - general	Healthcare that is unrelated to specific sign/symptom-associated disease onsets or disease onset dates.
Healthcare facility	Places or institutions where healthcare is provided; including hospitals, clinics, healthcare centers, health posts, and specialized care centers.
Haemophilus influenzae type B	A bacterium that causes a range of illnesses and is spread by droplets through coughs and sneezing, and occurs primarily among children <5years of age; common signs are fever, headache, and stiff neck up to sepsis. It can cause ear infections, cellulitis (soft tissue infection), arthritis, upper respiratory infections, pneumonia, meningitis (potential brain damage) and epiglottitis (with airway obstructions).
Heart disorders	Any disorder that affects or is caused by the heart; conditions that involve narrowed/blocked blood vessels that can lead to heart attack, chest pain, or stroke; condition that may affect one's heart muscle, valves, rhythm, or blood pressure.
Hepatitis B	An infectious disease caused by the hepatitis B virus (HBV) that affects the liver (acute/chronic liver infection; may result in liver failure, cancer, or cirrhosis). The virus is spread from person-to-person through blood, semen, or other bodily fluids. Common symptoms are fever, malaise/weakness/fatigue, loss of appetite, nausea/ vomiting, abdominal discomfort/pain, dark-colored urine, joint pain and jaundice.
Hepatitis E	An infectious disease caused by the hepatitis E virus (HEV) that affects the liver (only acute liver infection; may result in acute liver failure). The virus is spread via the fecal-oral route through the ingestion of contaminated food and water, infected animals (zoonotically) and blood. Common symptoms are fever, malaise, loss of appetite, diarrhea, nausea, abdominal discomfort, dark-colored urine, joint pain and jaundice.
HIV/AIDS	An infectious disease caused by the human immunodeficiency virus (HIV) that affects/damages the immune system and results in the acquired immunodeficiency syndrome (AIDS), a chronic, potentially life-threatening condition; it is transmitted through infectious body fluids such as blood, semen, and other bodily fluids. Common symptoms of an acute/early infection are fever, fatigue, headache, aches/joint pain, rash, sore throat, swollen glands/lymph nodes, diarrhea, weight loss, oral yeast infection and shingles. The untreated disease progresses to AIDS; symptoms caused by the severely damaged immune system are night sweats, recurring fever, chronic diarrhea, lesions on tongue and in the mouth, fatigue, weight loss, and skin rushes.
Household	A person or a group of related or unrelated persons that live together in the same dwelling unit, that acknowledge one male or female adult as the head of the household, that share the same housekeeping arrangements, that are considered to constitute one unit, and that provide themselves with food or other essentials for living. A household may be located in a single housing unit (single-story building) or in a set of collective living quarters (multi-story building).
**I**	
Income/Wage	Money or another form of payment that someone receives periodically in exchange for providing a good or service. It is usually received from a job/occupation.
Influenza	An infectious, highly contagious disease of the respiratory tract that is caused by flu viruses; it is spread from person-to-person via infected saliva droplets and contaminated objects. Common symptoms are fever, aches in muscles/arms/legs,

For peer review only

	chills/sweats, headache, fatigue/weakness, runny nose/nasal congestion, sneezing, sore throat and dry persistent cough.
Intestinal pain/ Abdominal pain	Pain (acute/short-lived or chronic/long-lived) that occurs anywhere between the chest and the groin (often referred to as the stomach region/belly).
**J**	
Jaundice	A condition of yellowing of the skin/whites of the eyes.
**K/L**	
**M**	
Malaise/Fatigue/ Weakness	The state of feeling tired, exhausted, or weak; a general feeling of discomfort, uneasiness, unhealthiness, or unhappiness.
Malaria	An infectious, sometimes fatal disease caused by a parasite (*Plasmodium* spp.) that is transmitted primarily by the bite of infected mosquitoes; infection may also spread from mother-to-child prenatally or during delivery, or through contact with contaminated blood. Common symptoms are recurrent attacks of chills, sweating, high fever, headache, vomiting and diarrhea.
Measles	An infectious, sometimes fatal disease of the respiratory tract (nose/throat) that is caused by a virus that affects mainly children <5years of age; it is spread from person-to-person via infected saliva droplets and contaminated objects. Common symptoms are fever, dry cough, runny nose, inflamed eyes, sore throat, large flat spots on the skin, and white spots in the mouth and on the cheek.
Meningitis	A viral/bacterial/fungal, sometimes life-threatening infectious inflammation of the membranes (meninges) surrounding the brain and spinal cord; it is spread from person-to-person via infected saliva droplets and contaminated objects. Common symptoms are high fever, severe headache, nausea/vomiting, confusion, seizures, loss of appetite and thirst, fatigues, sensitivity to light, skin rush and stiff neck.
Mumps	A viral infectious disease caused by the mumps virus that affects mainly the parotid glands that are situated below and in front of the ears; it is spread from person-to-person via infected saliva droplets and contaminated objects. Common symptoms are fever, swelling of one/both parotid glands, headache, muscle aches, weakness/fatigue, loss of appetite and pain while shewing/swallowing; a known complication may be hearing loss.
**N**	
Nausea/Vomiting	A feeling of sickness in the stomach with an inclination to vomit; a condition of ejecting part or all of the contents of the stomach, primarily through the mouth.
Neighbor	A person (related or unrelated) living next door to a household; a neighbor does not live together with members of a household (next door) in the same dwelling unit, does not share the same housekeeping arrangements, food, or other living essentials with members of a household (next door), and does not constitute one unit with members of a household (next door).
**O**	
Occupation	A job, or means of earning a wage/living; often requires the mastery of a complex set of knowledge, tasks, duties and skills acquired through formal education and/or practical experience.
**P**	
Pain/Aches	A condition of having continuous/prolonged dull pain in a part of the body; the physical feeling, suffering, or discomfort caused by an illness or injury.
Pertussis	Pertussis, also known as whooping cough, is a highly contagious bacterial disease. Symptoms similar to a common cold like runny nose, fever and cough are followed by weeks of sever coughing and a high-pitched whooping sound or gasping when a diseased person breathes in; severe coughing my even be followed by vomiting, breaking of ribs, or extreme fatigue. Infected very young and old people may have little or no cough, or they may have periods of interrupted breathing during which they do not breath are all. Pertussis is transmitted by the bacterium *Bordetella pertussis* through coughs and sneezes of an infected person.
Pharmacy	A place where medicines (prescription or nonprescription) are prepared, preserved, compounded and dispensed.

For peer review only - http://bmjopen.bmj.com/site/about/guidelines.xhtml

Physician	A person skilled/trained in healing; a person educated, clinically experienced, and licensed to practice medicine.
Pneumococcus	A bacterial infection caused by the bacterium *Streptococcus pneumoniae*; a sometimes fatal inflammation of the lungs that often affects immunocompromised people; it is spread from person-to-person via infected saliva droplets and contaminated objects. Common symptoms are fever, chills, cough (with phlegm/pus), chest pain, difficulty in breathing/shortness of breath, fatigue, nausea/vomiting and diarrhea; rare symptoms are sepsis, pleural effusion, and empyema.
Polio	A viral, highly contagious, sometimes fatal infectious disease caused by the poliovirus; in its most severe form causes paralysis and difficulties in breathing; it is transmitted via the fecal-oral route through ingestion of contaminated water and food, and from person-to-person contact. Common symptoms are fever, sore throat, headache, vomiting, fatigue, back pain/neck pain/stiffness, muscle weakness/aches, meningitis, loss of reflexes, and loose/floppy limbs.
**Q**	
**R**	
Rapid breathing/ Shortness of breath	The condition of abnormally fast breathing; a rate of >20 breaths per minute for adults, >30 breaths per minute for children and >45 breaths per minute for babies.
Respondent	An adult (at the country-specific legal age of majority) household member who is a decision-maker for the entire household within the study site; thus, this adult person serves as a proxy for all household members. This person may be identified by other members of the same household as the person who is primarily involved in the daily healthcare of household members.
Rotavirus	A viral infectious disease caused by rotavirus that results predominantly in diarrhea among children <5years of age; it spreads through the fecal-oral route from person-to-person contact and contaminated objects. Common symptoms are watery diarrhea, fever, vomiting, abdominal pain and dehydration.
Rubella	A contagious, viral infectious disease caused by the rubella virus; known by its distinctive red rash; it is spread from person-to-person via infected saliva droplets), from mother-to-child prenatally via blood, and contaminated objects. Common symptoms are mild fever, headache, stuffy/runny nose, inflamed red eyes, enlarged tender lymph nodes at the base of the skull/back of the neck/behind the ears, joint pain and a fine pink rash that moves from the face to the trunk, arms and legs.
Runny nose	The condition when the nose (nasal and adjacent tissues and blood vessels) produces extra mucus/excess drainage.
**S**	
Self-treatment	The medication of oneself or treatment of one's own disease or condition without medical supervision or prescription.
Severe breathing/ Difficulties in breathing	The condition of abnormal breathing that may be accompanied by wheezing, chest pain, chest in-drawing, stridor, swelling in the chest, or cough for instance.
Shivering/Chills	The sensation of coldness that could be accompanied by shivering or shaking of the body and pallor of the skin.
Shortness of breath/ Rapid breathing	The condition of abnormally fast and deep breathing; a rate of >20 breaths per minute for adults, >30 breaths per minute for children and >45 breaths per minute for babies.
Sneezing	The condition of suddenly forcing air out through nose and mouth with a usual loud noise.
Swelling/Edema	A condition of an excess collection of a watery fluid/swelling in the cavities or tissues of the body; it's commonly noticed in the hands/arms/feet/legs; volume increase of tissue.
**T**	
Tetanus	Tetanus is an infection characterized by muscle spasms which commonly begins in the jaw and progresses to the rest of the body. The spasms usually last for a few minutes only and occur frequently during 3-4 weeks of the illness but can be so

For peer review only - http://bmjopen.bmj.com/site/about/guidelines.xhtml

	severe that they may cause bone fractures or death when they affect the respiratory muscles. Tetanus may be characterized by further symptoms like fever, sweating, headache, difficulties in swallowing, high blood pressure, and an increased heart rate. The disease is caused by an infection with the bacterium *Clostridium tetani* that is found in soil, salvia, dust and manure. The bacterium enters through a skin injury (e.g. cut, puncture wound) by a contaminated object. It produces toxins that interfere with muscle contractions that result in the typical spasms.
Traditional healer	A person that aims to maintain health as well as to prevent, diagnose, and improve or treat illnesses by using a sum of knowledge, skills and practices based on theories, beliefs and experiences.
Tuberculosis	A contagious, potentially serious infectious disease caused by the bacteria *Mycobacterium tuberculosis*; primarily affects the lungs, but can also affect the kidneys and spine/brain; it is transmitted from person-to-person via infected saliva droplets. Common symptoms of active tuberculosis are cough (with or without blood), chest pain, weight loss, fatigue, fever, night sweats, chills and loss of appetite.
Typhoid fever	A bacterial, potentially life-threatening infectious disease caused by *Salmonella typhi*; it is transmitted via the fecal-oral route through contaminated food and water, or from person-to-person contact. Common symptoms are high fever, headache, abdominal pain, constipation/diarrhea, and rash. Serious complications (i.e. perforation of the gut) are reported.
**U**	
Unconsciousness/ Dizziness/Confusion	The state of being bewildered or unclear in mind; an abnormal state of response to stimuli in the environment; faint, wooziness, weakness, unsteadiness or a tendency to fall; the interruption of the awareness of oneself and one's surroundings.
**V**	
Varicella	A viral, highly contagious infectious disease, commonly known as chickenpox, that primarily affects children and is caused by the varicella-zoster virus; it is spread from person-to-person by contact with infected blisters or saliva droplets. Common symptoms are mild fever, headache, backache, loss of appetite, and vesicular skin eruptions/rash/red spots on the back and chest accompanied by severe itching.
Visitor	A person (related or unrelated) who comes to a household to spend some time with household members; a visitor does not live together with household members in the same dwelling unit, does not share the same housekeeping arrangements, food, or other living essentials with household members, and does not constitute one unit with household members.
Vomiting/Nausea	A feeling of sickness in the stomach with an inclination to vomit; a condition of ejecting part or all of the contents of the stomach, primarily through the mouth.
**W**	
Weakness/Malaise/ Fatigue	The state of feeling tired, exhausted, or weak; a general feeling of discomfort, uneasiness, unhealthiness, or unhappiness.
Weight loss	The (voluntary or involuntary) decrease in body weight.
Witness	A person who is an adult (at the country-specific legal age of majority), who is independent of the study, who cannot be unfairly influenced by study staff, who attends the informed consent process if the subject or subject's legally acceptable representative is illiterate, and who reads (literate) the informed consent form and any other written information supplied to the subject.
**X/Y/Z**	
Yellow fever	An acute infectious, human pathogenic, viral disease that is marked by the sudden onset of symptoms like fever, chills, loss of appetite, nausea, muscle pain, and headache, that typically resolve within a few days or are followed by more serious symptoms such as jaundice, high fever and hemorrhage. The disease is caused by a virus of the genus Flavivirus and is transmitted by the bite of an infected mosquito.

For peer review only

Site ___ ___ ___ Subarea ___ ___ ___ House number ___ ___ ___ ___ Interviewer ID ___ ___

Appendix 2/Supplementary file 2: Study forms (English)

FORM 1 – RECORD OF HOUSEHOLD VISITS

Instructions to the interviewer

- Complete “Form 1 – RECORD OF HOUSEHOLD VISITS” for every household visited.
- Complete “Home/Respondent/Completed” once all survey parts have been completed; if not all data haven been collected at the initial and/or a consecutive visit (max. 3 visits), schedule a revisit.
- Attempt 1: complete the status of the household visit using given options; for “Not Home”, “Home/Non-Respondent” and “Home/Respondent/Unavailable” schedule a revisit; for “Home/Respondent/Refusal” state the reason for refusal and visit a replacement household to the nearest on the right or left side of this household; for ~~and~~ “Non-existing/Non-residential” skip this household and visit a replacement household to the nearest on the right or left side of this household instead.
- Attempt 2: complete the status of the household visit using given options; for “Not Home”, “Home/Non-Respondent” and “Home/Respondent/Unavailable” schedule a revisit.
- Attempt 3: complete the status of the household visit using given options; for “Not Home”, “Home/Non-Respondent”, “Home/Respondent/Unavailable” skip this household and visit a replacement household to the nearest on the right or left side of this household instead.
- “Site” and “Subarea” must be entered each as a 3-digit, “House number” as a 4-digit, and “Interviewer ID” as a 2-digit code (use lists individually prepared for each site to obtain codes); enter “Date” in the format DD/MM/YY and “Time” in the format HH:MM and circle AM or PM. “Study label” must be a combination of a 3-digit “Site” code, a 3-digit “Subarea” code and a 4-digit “House number” code; the “Interviewer ID” must be a entered as a 3-digit code.

**Attempt 1**	Date ____/____/____ (DD/MM/YY)	Time _____/_____ AM/PM	Interviewer ID ___ ___
STATUS		Tick one	
1=Not home		O	➡ Schedule revisit (Date/Time)
2=Home/Non-Respondent		O	➡ Schedule revisit (Date/Time)
3=Home/Respondent/Unavailable		O	➡ Schedule revisit (Date/Time)
4=Home/Respondent/Completed		O	
5=Home/Respondent/Refusal		O	Record the reason(s) for refusal; tick all that applies<br>O 1=No time to answer questions<br>O 2=Don’t want to participate/answer questions<br>O 3=Other ______________________<br>O 98=No response O 99=Don’t know<br>➡ Visit a replacement household to the nearest right or left side of this household
6=Non-existing/Non-residential		O	➡ Visit a replacement household to the nearest right or left side of this household
**Attempt 2**	Date ____/____/____ (DD/MM/YY)	Time _____/_____ AM/PM	Interviewer ID ___ ___
STATUS		Tick one	
1=Not home		O	➡ Schedule revisit (Date/Time)
2=Home/Non-Respondent		O	➡ Schedule revisit (Date/Time)
3=Home/Respondent/Unavailable		O	➡ Schedule revisit (Date/Time)

For peer review only - http://bmjopen.bmj.com/site/about/guidelines.xhtml

Site ___ ___ ___ Subarea ___ ___ ___ House number ___ ___ ___ ___ Interviewer ID ___ ___

4=Home/Respondent/Completed	O	
5=Home/Respondent/Refusal	O	Record the reason(s) for refusal; tick all that applies<br>O 1=No time to answer questions<br>O 2=Don't want to participate/answer questions<br>O 3=Other ________________<br>O 98=No response O 99=Don't know<br>➡ Visit a replacement household to the nearest right or left side of this household
6=Non-existing/Non-residential	O	➡ Visit a replacement household to the nearest right or left side of this household
**Attempt 3** Date ____/____/____ (DD/MM/YY)	Time _____/_____ AM/PM	Interviewer ID ___ ___
STATUS	Tick one	
1=Not home	O	➡ Visit a replacement household to the nearest right or left side of this household
2=Home/Non-Respondent	O	➡ Visit a replacement household to the nearest right or left side of this household
3=Home/Respondent/Unavailable	O	➡ Visit a replacement household to the nearest right or left side of this household
4=Home/Respondent/Completed	O	
5=Home/Respondent/Refusal	O	Record the reason(s) for refusal; tick all that applies<br>O 1=No time to answer questions<br>O 2=Don't want to participate/answer questions<br>O 3=Other ________________<br>O 98=No response O 99=Don't know<br>➡ Visit a replacement household to the nearest right or left side of this household
6=Non-existing/Non-residential	O	➡ Visit a replacement household to the nearest right or left side of this household

For peer review only

Site ___ ___ ___ Subarea ___ ___ ___ House number ___ ___ ___ ___ Interviewer ID ___ ___

FORM 2 – CONSENT FORM

Healthcare assessment in [add study site and -country]

Instructions to the interviewer

– Read "Form 2 – CONSENT FORM" to the respondent/ask the respondent to carefully read through "Form 2 – CONSENT FORM" if preferred.
– The respondent as defined for this investigation is an adult (at the country-specific legal age of majority) household member and decision-maker with respect to daily healthcare/healthcare utilization for the entire household and its members; it must not be e.g. a neighbour or a visitor.
– Assure that the respondent has fully understood the purpose, procedures, risks and benefits of this study prior to obtaining the respondent's consent to participate.
– The respondent must sign "Form 2 – CONSENT FORM"; if the respondent is unable to sign/illiterate, a thumbprint will serve in place of a signature and a study-independent literate adult witness (e.g. member of same household/neighbour) will sign "Form 2 – CONSENT FORM".
– Collect data about all household members regardless of whether all household members are present at the time of the interview; collecting and recording data only about individuals present at the time of the interview will result in biased data.
– Complete "Form 2 – CONSENT FORM" once for every household consented/enrolled in the study.
– Explain to the respondent that it is anticipated to revisit every household during a 2-year period; the study team will obtain a new consent for participation during each visit.
– "Site" and "Subarea" must be entered each as a 3-digit, "House number" as a 4-digit, "Interviewer ID" as a 2-digit code (use lists individually prepared for each site to obtain codes), and "Date" in the format DD/MM/YYYY. "Study label" must be a combination of a 3-digit "Site" code, a 3-digit "Subarea" code and a 4-digit "House number" code; the "Interviewer ID" must be entered as a 3-digit code.

Study principal- and co-investigators

[add names of study principal- and co-investigators and respective affiliations]

Introduction

The International Vaccine Institute (IVI) and the [add affiliation(s) of local investigators] are working together to look into health problems and healthcare seeking behaviour in your community [add study site and -country], including the socio-economic status, hygiene and sanitation behaviour, and the vaccination status among children. We would like to better understand where you, your family and your neighbours seek healthcare in case of medical conditions associated with fever/hot body and other signs & symptoms. This information will help us to find out how common fever-related and other conditions are, provide a better picture of influencing factors involved in disease transmission and infection, and to find ways to prevent and treat illnesses in your community. You, the primary decision-maker for daily healthcare/healthcare utilization for this household and its members, are invited to participate because you live in the area where we are conducting this study. This consent form will be read by you/to you and you should take your time to make your decision to accept or decline the participation in the study. Please ask members of the study team to explain any word(s) or information that you do not understand. Further information about this study, including its potential risks, benefits and inconveniences will be explained subsequently. After the study has been fully explained to you and if you agree to participate, you will be asked to sign this consent form on behalf of all household members. If you are unable to sign the form, we will ask you to provide your thumbprint, and a study-independent adult person (member of your household/neighbour) will sign the form as a witness. We will give you a signed and dated copy of the consent form.

Purpose

The purpose of this study is to look into health problems and utilization of healthcare in your community [add study site and -country], including the socio-economic status and hygiene and sanitation behaviour. We aim to better understand where you, your family and your neighbours seek healthcare for conditions associated with fever/hot body or other sings & symptoms. This information will help better estimate the burden of illnesses common in your community, and to identify ways to introduce measures to prevent and treat them.

For peer review only - http://bmjopen.bmj.com/site/about/guidelines.xhtml

Site ___ ___ ___ Subarea ___ ___ ___ House number ___ ___ ___ ___ Interviewer ID ___ ___

Procedures
We will visit about [add number for study site and -country] households in total in [add study site and -country] and will ask the same questions to each household. Your household was chosen for this study simply by chance. If you agree to participate, we will ask you on behalf of all household members a few questions about your household such as the initials, sex and age of each member. We would like to know some information related to the education and occupation of the household head, and income, possession of household items, the housing itself as well as hygiene and sanitation in the context of personal hygiene, food preparation and handling, water sources and handling, waste disposal, handling of farm and domestic animals as well as farming of this household. We will also ask where members of your household get help if anyone is sick due to fever-related and other conditions and about factors that may influence healthcare seeking behaviour of household members (i.e. travel modalities, availability of health insurance, cultural/ethnographic factors) besides the immunization status of children of this house and the perception of some diseases. Finally, we will measure the geographic location of your house. There are no right or wrong answers. You are free to choose which questions to answer. All in all, it should take about 30-45 minutes to answer all questions. The interview may take a bit longer if there are many members in this household. Also, we will visit your household two times in different seasons (once during the dry season and once towards the end of the rainy season) during the overall study period of about two years to collect data as described in this paragraph of the consent form.

Participation
You, the primary decision-maker for daily healthcare/healthcare utilization for this household and its members, and members of your household are free to choose to accept or decline to be part of the study. If you do not want to participate, that is ok. If you want to stop your participation at any time, that is ok, and there will be no harm to you or members of your household. So, participation in this study is voluntary, and you are free to refuse to participate in the study or you can withdraw your consent at any time without giving reasons and this will not involve any penalty.

Risks & Benefits
There are no direct risks for participating in this study. Asking questions about your household may make you or household members feel uncomfortable. Study teams may help providing information on established community healthcare resources for participants that express distress after completing the questions. You can refuse to answer any question at any time. You may take a break or stop participating in this study at any time. There are no direct benefits for being in this study. During visits of your household, our study teams may identify members of your household that should seek healthcare for fever-associated conditions and others. Hence, they may help providing information on established healthcare resources in your community. Indirect benefits are that all information collected will provide exact information on health problems and healthcare utilization for various conditions, including vaccination for young children, the socio-economic status as well as hygiene and sanitation behaviour in your community. This will help to provide a better picture of influencing factors involved in disease transmission and infection, and to estimate the burden of common diseases as well as to find ways to prevent and treat those in your community.

Costs & Compensation
There are no costs for participants of this study. You and anyone from your household will not be compensated for your participation.

Confidentiality
All study records and all collected information that identify you and members of your household will be kept confidential. All paper-based files will be locked safely and computerized files will be password-protected; all data files (paper-based/computerized) will be stored for a minimum of five years; these files will be accessible to authorized few study staff only. Your name or any identifier will not be used in any publication or reports from this study. Information that we collect about you and members of your household will only be shared with authorized study members such as authorized study staff and officials from ethics committees.

For peer review only - http://bmjopen.bmj.com/site/about/guidelines.xhtml

Site ___ ___ ___ Subarea ___ ___ ___ House number ___ ___ ___ ___ Interviewer ID ___ ___

Contact point for questions

If you or members of your household have any questions, please ask them now to the study team or at any time later by contacting [add name of local principal- and/or co-investigator, respective affiliations, and phone numbers]. If you have questions as a study participant about your/your household members' rights, you can contact the [add name and contact details of the local ethics committee].

Statement of consent

This consent form for participating in the assessment of health behaviour and other population-based factors in [add study site and -country] has been read by me/to me carefully. The purpose, procedures, risks & benefits have been explained to me in detail. I have been allowed to ask questions at any time, and my questions have been answered to my satisfaction by the study team. I have been told whom to contact if I have questions, want to discuss problems or concerns. I have been told that I will be given a signed and dated copy of this consent form. I have been reassured that all information obtained as result of this study will be kept confidential and used only for the purpose of this study and only by participating institutions.

I, the respondent, consent hereby voluntarily to participate in this study. I will follow the directions of the study team and give them my full cooperation. I understand that I have the right to withdraw from the study at any time.

RESPONDENT

Name: ____

(given name + middle name (if available) + family name)

Signature: ____

(handwritten depiction of person's name/mark that the person writes on a document as a proof of identity)

Date ______/______/_______________ (DD/MM/YYYY)

Thumbprint

If the respondent is unable to sign/illiterate but agrees to participate, record the respondent's thumb print in the above box, and have an adult study-independent literate witness (e.g. member of the same household or a neighbor) sign below:

WITNESS

Name: ____

(given name + middle name (if available) + family name)

Signature: ____

(handwritten depiction of person's name/mark that the person writes on a document as a proof of identity)

Date ______/______/_______________ (DD/MM/YYYY)

I, the interviewer, have read/explained the study to the above named respondent (witness if respondent is illiterate) in a language that the respondent understands well. I am certain that the respondent has understood the information and he/she agrees to be asked some questions.

INTERVIEWER

Name: ____

(given name + middle name (if available) + family name)

Signature: ____

(handwritten depiction of person's name/mark that the person writes on a document as a proof of identity)

Date ______/______/_______________ (DD/MM/YYYY)

For peer review only - http://bmjopen.bmj.com/site/about/guidelines.xhtml

Site ___ ___ ___ Subarea ___ ___ ___ House number ___ ___ ___ ___ Interviewer ID ___ ___

FORM 3: GENERAL HOUSEHOLD INFORMATION

Instructions to the interviewer

- Complete this form only if the respondent consented to participate (see "Form 2 – CONSENT FORM"); the respondent as defined for this investigation is an adult (at the country-specific legal age of majority) household member and decision-maker with respect to daily healthcare/healthcare utilization for the entire household and its members; it must not be e.g. a neighbour or a visitor.
- Collect data about all household members regardless of whether all household members are present at the time of the interview; collecting and recording data only about individuals present at the time of the interview will result in biased data.
- A household is/household members are defined as a person or a group of related or unrelated persons that live together in the same dwelling unit, that acknowledge one male or female adult as the head of the household, that share the same housekeeping arrangements, that are considered to constitute one unit, and that provide themselves with food or other essentials for living.
- "Site" and "Subarea" must be entered each as a 3-digit, "House number" as a 4-digit, and "Interviewer ID" as a 2-digit code (use lists individually prepared for each site to obtain codes). "Study label" must be a combination of a 3-digit "Site" code, a 3-digit "Subarea" code and a 4-digit "House number" code; the "Interviewer ID" must be entered as a 3-digit code.

FORM 3: GENERAL HOUSEHOLD INFORMATION
PART A – DEMOGRAPHIC DATA/GENERAL HOUSEHOLD INFORMATION

HOUSE LOCATION

1 Site, subarea and house number

Instructions: Site (=study site) and subarea (=smallest unit of the study site) must be recorded each as a 3-digit, and house number as a 4-digit code; use individually prepared lists to obtain the respective codes.

Site: ____ ____ ____ Subarea: ____ ____ ____ House number: ____ ____ ____ ____

2 GPS coordinates

Instructions: Take GPS coordinates at the approx. center of the house. Record altitude as a 4-digit number (unit: meter), and latitude/longitude as a 7-digit number (unit: degrees, decimal minutes).

O 1=Original household O 2=Replacement household (right/left side of original household)

Altitude: ___ ___ ___ ___

Latitude: ___ ___°___ ___.___ ___ ___' Longitude: ___ ___°___ ___.___ ___ ___'

RESPONDENT

3 Provide the full name of the respondent.

Instructions: Record given name, middle name (if available) and family name of the respondent.

____

4 Provide the age of the respondent.

Instruction: Recorded as a 3-digit number (unit: year). ____ ____ ____

5 Provide the sex of the respondent.

O 1=Male O 2=Female

6 What is the respondent's relationship to this household?

O 1=Household head
O 2=Son/daughter of household head
O 3=Brother/sister of household head
O 4=Mother/father of household head
O 5=Granddaughter/grandson of household head
O 6=Other, specify __________
O 7=Spouse of household head
O 8=Spouse of son/daughter of household head
O 9=Spouse of brother/sister of household head
O 10=Mother/father in law of household head
O 11=Niece/nephew of household head
O 98=No response

For peer review only - http://bmjopen.bmj.com/site/about/guidelines.xhtml

Site ___ ___ ___ Subarea ___ ___ ___ House number ___ ___ ___ ___ Interviewer ID ___ ___

HOUSEHOLD MEMBERS

7 How many household members are living in this house in total (at the day of visit)**?**

Instruction: Recorded as a 2-digit number.

Total number of household members: ____ ____

8 Record age and sex of each household member.

Instructions: Record the respondent's initials, age and sex at the first position/row of the table. Initials, age and sex of all other household members must be recorded subsequently. The initials of a household member consist of the first three letters of the given name and the first three letters of the family name (a potential middle name is not part of the initials). Record the age of each household member as a 3-digit number (unit: year); if a household member's age is ≤12 months, record the age as 1 year. Please complete an additional form if a household has >20 members.

Household member identifier	Initials [given name/family name]	Age [year]	Sex
1/Respondent	____ ____ ____/____ ____ ____	____ ____ ____	O1=Male O2=Female
2	____ ____ ____/____ ____ ____	____ ____ ____	O1=Male O2=Female
3	____ ____ ____/____ ____ ____	____ ____ ____	O1=Male O2=Female
4	____ ____ ____/____ ____ ____	____ ____ ____	O1=Male O2=Female
5	____ ____ ____/____ ____ ____	____ ____ ____	O1=Male O2=Female
6	____ ____ ____/____ ____ ____	____ ____ ____	O1=Male O2=Female
7	____ ____ ____/____ ____ ____	____ ____ ____	O1=Male O2=Female
8	____ ____ ____/____ ____ ____	____ ____ ____	O1=Male O2=Female
9	____ ____ ____/____ ____ ____	____ ____ ____	O1=Male O2=Female
10	____ ____ ____/____ ____ ____	____ ____ ____	O1=Male O2=Female
11	____ ____ ____/____ ____ ____	____ ____ ____	O1=Male O2=Female
12	____ ____ ____/____ ____ ____	____ ____ ____	O1=Male O2=Female
13	____ ____ ____/____ ____ ____	____ ____ ____	O1=Male O2=Female
14	____ ____ ____/____ ____ ____	____ ____ ____	O1=Male O2=Female
15	____ ____ ____/____ ____ ____	____ ____ ____	O1=Male O2=Female
16	____ ____ ____/____ ____ ____	____ ____ ____	O1=Male O2=Female
17	____ ____ ____/____ ____ ____	____ ____ ____	O1=Male O2=Female
18	____ ____ ____/____ ____ ____	____ ____ ____	O1=Male O2=Female
19	____ ____ ____/____ ____ ____	____ ____ ____	O1=Male O2=Female
20	____ ____ ____/____ ____ ____	____ ____ ____	O1=Male O2=Female

For peer review only

For peer review only - http://bmjopen.bmj.com/site/about/guidelines.xhtml

Site ___ ___ ___ Subarea ___ ___ ___ House number ___ ___ ___ ___ Interviewer ID ___ ___

FORM 3: GENERAL HOUSEHOLD INFORMATION
PART B – SOCIO-ECONOMIC DATA

EDUCATION

1 Can the head of household read in [add the official language of the study site/country]**?**

O 1=Yes O 2=No O 99=Don't know O 98=No response

2 Can the head of household write in [add the official language of the study site/country]**?**

O 1=Yes O 2=No O 99=Don't know O 98=No response

3 What is the highest completed level of formal school education of the head of household?

O 1=Primary school O 4=Higher than secondary school O 99=Don't know
O 2=Middle school O 5=NO EDUCATION O 98=No response
O 3=Secondary school O 6=Other, specify ___________

OCCUPATION/INCOME

4 What is the main occupation of the household head?

Instructions: Record the status of the day of visit only.

O 1=Group 1: Managers
(chief executives, senior officials, legislators; administrative and commercial managers; production and specialized services managers; hospitality, retail and other services managers)

O 2=Group 2: Professional
(science and engineering professionals; health professionals; teaching professionals; business and administration professionals; information and communications technology professionals; legal, social and cultural professionals)

O 3=Group 3: Technicians and Associate Professionals
(science and engineering associate professionals; health associate professionals; business and administration associate professionals; legal, social, cultural and related associate professionals; information and communications technicians)

O 4=Group 4: Clerical Support Workers
(general and keyboard clerks; customer services clerks; numerical and material recording clerks; other clerical support workers)

O 5=Group 5: Services and Sales Workers
(personal services workers; sales workers; personal care workers; protective services workers)

O 6=Group 6: Skilled Agricultural, Forestry, and Fishery Workers
(market-oriented skilled agricultural workers; animal producers; marked-oriented skilled forestry, fishery and hunting workers; subsistence farmers, fishers, hunters and gatherers)

O 7=Group 7: Craft and Related Trades Workers
(building and related trades workers (excluding electricians); metal, machinery and related trades workers; handicraft and printing workers; electrical and electronics trades workers; food processing, woodworking, garment and other craft and related trades workers)

O 8=Group 8: Plant and Machine Operators, and Assemblers
(stationary plant and machine operators; assemblers; drivers and mobile plant operators)

O 9=Group 9: Elementary Occupations
(cleaners and helpers; agricultural, forestry and fishery labourers; labourers in mining, construction, manufacturing and transport; food preparation assistants; street and related sales and services workers; refuse workers and other elementary workers)

O 10=Group 0: Armed Forces Occupations
(commissioned armed forces officers; non-commissioned armed forces officers; armed forces occupations, other ranks)

O 11=Other, specify __________

O 12=NO OCCUPATION O 99=Don't' know O 98=No response

For peer review only - http://bmjopen.bmj.com/site/about/guidelines.xhtml

Site ___ ___ ___ Subarea ___ ___ ___ House number ___ ___ ___ ___ Interviewer ID ___ ___

5 What is the average monthly income of the household head based on the occupation stated in question 4 and of other household members?
Instruction: Tick one answer per household head/enter one code per household member if applicable/if income available.

Head of household	Member 1	Member 2	Member 3
Code ____________	Code ____________	Code ____________	Code ____________

[1] <100USD [3] ≥150 to <200USD [5] ≥250USD [99] Don't know
[2] ≥100 to <150USD [4] ≥200 to <250USD [6] Head of household NO INCOME (see Q4) [98] No response

6 Which and how much other/additional source(s) of monthly monetary earnings has this household?
Instruction: Tick all that applies; more than one answer is possible.

Other/additional sources	Overall monthly monetary earnings
O 1=Social grants<br>O 2=Insurance (i.e. private, public)<br>O 3=Loan (i.e. from friends, family, bank)<br>O 4=Selling (i.e. livestock, crops, furniture, other goods)<br>O 5=Personal savings<br>O 6=Other, specify ________________	O 1=<100 USD<br>O 2=≥100 to <150 USD<br>O 3=≥150 to <200 USD<br>O 4=≥200 to <250 USD<br>O 5=≥250 USD
O 7=NO OTHER/ADDITIONAL SOURCES<br>O 99=Don't know<br>O 98=No response	

HOUSING/DWELLING

7 Is the house owned by the members of this household?
O 1=Yes O 3=No, supplied (i.e. government, employer) O 99=Don't know
O 2=No, rented O 4=No, other, specify _________ O 98=No response

8 How many room(s)/individual house structure(s) of this household are used for sleeping, living, cooking and bathing?
Instruction: Enter one code per category only.

Bedroom	Living room	Kitchen	Bathroom
Code ____________	Code ____________	Code ____________	Code ____________

[1] 1-2 [3] >4 [99] Don't know
[2] 3-4 [4] None [98] No response

9 What is the construction material of the floor, the walls and the roof of room(s)/individual house structure(s) of this house?
Instruction: Enter all codes that applies per category; more than one answer per category is possible.

Floor	Walls	Roof
Code ____________	Code ____________	Code ____________

[1] Brick [3] Tiles [5] Wood [7] Metal/Tin [99] Don't know
[2] Cement/Concrete [4] Mud [6] Straw/Leaves [8] Other, specify _________ [98] No response

10 Do members of this household have access to/utilize electricity in this household?
O 1=Yes O 2=No O 99=Don't know O 98=No response

11 Which main source of light have members of this household access to/are utilizing?
O 1=Electric light/Light bulb(s) O 4=Battery-powered lamp(s) O 7=Other, specify _________
O 2=Solar light/Solar lamp(s) O 5=Candle(s) O 99=Don't know
O 3=Paraffin lamp(s)/Oil lamp(s) O 6=NO SOURCE OF LIGHT O 98=No response

For peer review only - http://bmjopen.bmj.com/site/about/guidelines.xhtml

Site ___ ___ ___ Subarea ___ ___ ___ House number ___ ___ ___ ___ Interviewer ID ___ ___

HOUSEHOLD ASSETS

12 Which of the following items do members of this household own?

Instructions: Tick all that applies; more than one answer is possible; record the quantity of functional items where applicable.

O 1=Radio	Quantity ____	O None	O 11=Tape/CD player	Quantity ____	O None
O 2=Television	Quantity ____	O None	O 12=Computer/Laptop	Quantity ____	O None
O 3=Internet connection	Quantity ____	O None	O 13=Mobile/Cell phone	Quantity ____	O None
O 4=Car	Quantity ____	O None	O 14=Truck/Bus	Quantity ____	O None
O 5=Motorbike	Quantity ____	O None	O 15=Bicycle	Quantity ____	O None
O 6=Oxcart/Donkey	Quantity ____	O None	O 16=Bed	Quantity ____	O None
O 7=Mattress	Quantity ____	O None	O 17=Bed net	Quantity ____	O None
O 8=Table	Quantity ____	O None	O 18=Chair	Quantity ____	O None
O 9=Refrigerator (4°C)	Quantity ____	O None	O 19=Freezer (-20°C)	Quantity ____	O None
O 10=Fan	Quantity ____	O None	O 20=Sewing machine	Quantity ____	O None

For peer review only

For peer review only - http://bmjopen.bmj.com/site/about/guidelines.xhtml

Site ___ ___ ___ Subarea ___ ___ ___ House number ___ ___ ___ ___ Interviewer ID ___ ___

FORM 3: GENERAL HOUSEHOLD INFORMATION
Part C – Hygiene and sanitation

TOILET FACILITY

1 To what type of toilet facility have members of this household usually access?

Instruction: Inside means inside a room/individual house structure/building; outside means outside a room/individual house structure/building.

- O 1=Flush toilet/Water closet (inside)
- O 2=Flush toilet/Water closet (outside)
- O 3=Non-flush latrine/pit latrine (inside)
- O 4=Non-flush latrine/pit latrine (outside)
- O 5=Ventilated Improved Pit Latrine (KVIP, inside)
- O 6=Ventilated Improved Pit Latrine (KVIP, outside)
- O 7=Composting toilet (inside)
- O 8=Composting toilet (outside)
- O 9=NO toilet facility, use a bucket
- O 10=NO toilet facility, dig a hole
- O 11=NO toilet facility, free range/open defecation
- O 12=Other, specify __________
- O 99=Don't know
- O 98=No response

2 Do members of this household usually utilize the toilet facility stated in question 1?

- O 1=No, members cannot utilize the facility independently (i.e. infants, toddlers)
- O 2=No, members cannot utilize the facility independently (i.e. disabled, long-term/chronically diseased)
- O 3=No, members cannot utilize the facility independently (i.e. seniors)
- O 4=No, limited access (i.e. public, shared with other households/community)
- O 5=No, too far away (i.e. public, shared with other households/community)
- O 6=No, bad condition (i.e. broken, not clean)
- O 7=YES
- O 8=Other, specify _________________
- O 99=Don't know
- O 98=No response

3 Where is the toilet facility stated in question 1 located? With how many other people is it usually shared?

- O 1=On the property of this household (private, not shared)
- O 2=On the property of a neighbouring household (private, shared)
  - *Shared* O 1=<10 people O 2=11-24 people O 3=25-49 people O 4=>50 people O 99=Don't know O 98=No response
- O 3=In the community (public, shared)
  - *Shared* O 1=<10 people O 2=11-24 people O 3=25-49 people O 4=>50 people O 99=Don't know O 98=No response
- O 4=Other, specify __________
- O 99=Don't know
- O 98=No response

4 How do members of this household that have/don't have access to a toilet facility usually clean their hands directly before and after urination/defection?

Instruction: Enter one code per category only.

Clean hands before urination/defection	Clean hands after urination/defection
Code _____________	Code _____________
[1] Wash hands with soap and water<br>[2] Wash hands with water only<br>[3] Rub hands clean with some leaves/straw/ grass<br>[4] Rub hands clean with some sand<br>[5] Rub hands clean with some cloth/fabric	[6] Don't clean, not needed/not dirty<br>[7] Don't clean, nothing to clean hands nearby/on hands<br>[8] Other, specify __________<br>[99] Don't know<br>[98] No response

KITCHEN/FOOD HANDLING

5 Do members of this household have a kitchen for cooking/preparing food/meals? Where is it located?

- O 1=Yes, inside kitchen/in a room/individual house structure; on the property of this household (private, not shared)
- O 2=Yes, outside kitchen/outside a room/individual house structure/ open air; on the property of this

For peer review only - http://bmjopen.bmj.com/site/about/guidelines.xhtml
For peer review only

Site ___ ___ ___ Subarea ___ ___ ___ House number ___ ___ ___ ___ Interviewer ID ___ ___

household (private, not shared)
O 3=Yes, inside kitchen/in a room/individual house structure; on the property of a neighbouring household (private, shared)
O 4=Yes, outside kitchen/outside a room/individual house structure/ open air; on the property of a neighbouring household (private, shared)
O 5=NO KITCHEN
O 6=Other, specify __________ O 99=Don't know O98=No response

6 What energy source do members of this household usually use for cooking/preparing food/meals?

O 1=Electricity O 3=Charcoal O 5=Animal dung O 99=Don't know
O 2=Gas/Kerosene O 4=Wood/straw/leaves O 6=Other, specify _____ O 98=No response

7 What cooking spot do members of this household usually use for cooking/preparing food/meals?

O 1=Open fire O 3=Closed stove with chimney O 99=Don't know
O 2=Open stove O 4=Other, specify __________ O 98=No response

8 Where do members of this household usually store fresh/raw food items (e.g. vegetables, lettuce, fruits, dairy products, eggs, meat and fish) and left-over/prepared food/meals?

Instruction: Enter one code per category only. Inside means inside a room/individual house structure/building; outside means outside a room/individual house structure/building.

Fresh/raw food items (i.e. vegetables, lettuce, fruits, dairy products, eggs, meat and fish)	Left-over/prepared food/meals
Code _____________	Code _____________

[1] At room temperature (inside, on the property of this household, private)
[2] At room temperature (outside, on the property of this household, private)
[3] At room temperature (inside, on the property of a neighboring household, private, shared)
[4] At room temperature (outside, on the property of a neighboring household, private, shared)
[5] Refrigerator (on the property of this household, private)
[6] Refrigerator (on the property of a neighboring household, private, shared)
[7] Freezer (on the property of this household, private)
[8] Freezer (on the property of a neighboring household, private, shared)
[9] Other, specify ______________
[10] NO STORAGE [99] Don't know [98] No response

9 If the answer is "Room temperature" for fresh/raw food items and/or left-over/ prepared food/meals in question 8, how long do members of this household usually store them?

Instruction: Enter one code per category only.

Fresh/raw food items (i.e. vegetables, lettuce, fruits, dairy products, eggs, meat and fish)	Left-over/prepared food/meals
Code _____________	Code _____________

[1] <1 day [3] 3-4 days [5] Not applicable [99] Don't know
[2] 1-2 days [4] >4 days [98] No response

10 Do members of this household usually cover left-over/prepared food/meals? Do they re-heat/re-cook left-over/prepared food/meals prior to consumption?

Instruction: Enter one code per category only.

Cover left-over/prepared food/meals	Re-heat/Re-cook left-over/prepared food/meals
Code _____________	Code _____________

[1] Yes [2] No [99] Don't know [98] No response

For peer review only - http://bmjopen.bmj.com/site/about/guidelines.xhtml

Site ___ ___ ___ Subarea ___ ___ ___ House number ___ ___ ___ ___ Interviewer ID ___ ___

11 Which member(s) of this household usually cook(s)/prepare(s) food/meals for other members of this household?
Instructions: Refer to question 9 of "Form 3 Part A – Demographic data/General household information" and record the initials/identifier(s) of the member.
O Household member, record initials/identifier(s) __________ O 99=Don't know O 98=No response

12 How does/do the household member(s) that usually cook(s)/prepare(s) food/meals for other members of this household as stated in question 11 usually prepare(s) herself/himself/themselves before and after the preparation of meals?
Instruction: Enter one code per category only.

Before the preparation of meals	After the preparation of meals
Code _____________	Code _____________
[1] Wash hands with soap and water<br>[2] Wash hands with water only<br>[3] Rub hands clean with some leaves/straw/ grass<br>[4] Rub hands clean with some sand<br>[5] Rub hands clean with some cloth/fabric	[6] Don't clean, not needed/not dirty<br>[7] Don't clean, nothing to clean hands nearby/on hands<br>[8] Other, specify __________<br>[99] Don't know<br>[98] No response

13 How do members of this household usually prepare themselves before and after eating food/meals, including the household member that usually cooks/prepares food/meals as stated in question 11?
Instruction: Enter one code per category only.

Before the eating of food/meals	After the eating of food/meals
Code _____________	Code _____________
[1] Wash hands with soap and water<br>[2] Wash hands with water only<br>[3] Rub hands clean with some leaves/straw/ grass<br>[4] Rub hands clean with some sand<br>[5] Rub hands clean with some cloth/fabric	[6] Don't clean, not needed/not dirty<br>[7] Don't clean, nothing to clean hands nearby/on hands<br>[8] Other, specify __________<br>[99] Don't know<br>[98] No response

14 How do members of this household usually eat their food/meals, including the household member that usually cooks/prepares food/meals as stated in question 11?
O 1=With cutlery O 3=Other, specify _____________ O 99=Don't know
O 2=With fingers O 98=No response

15 Do members of this household usually eat their food/meals from the same pot, including the household member that usually cooks/prepares food/meals as stated in question 11?
O 1=Yes O 2=No O 99=Don't know O 98=No response

16 Where do members of this household usually get their main fresh/raw food items such as vegetables, fruits and grains/cereals, dairy products, eggs, meat and fish?
Instruction: Enter one code per category only.

Vegetables, fruits, grains/ cereals	Dairy products	Eggs, meat, fish
Code _____________	Code _____________	Code _____________

[1] Self-farming (i.e. back-/courtyard)
[2] Supermarket/Grocery store
[3] Friend/Family
[4] Open market
[5] Street vendor
[6] Self-farming (i.e. agricultural land)
[7] Butcher
[8] Other, specify _____________
[99] Don't know
[98] No response

17 Do members of this household usually consume/eat fresh/raw food items (e.g. vegetables, lettuce, fruits, dairy products, ice cream, eggs, meat and fish)? If so, which ones?

For peer review only - http://bmjopen.bmj.com/site/about/guidelines.xhtml

Site ___ ___ ___ Subarea ___ ___ ___ House number ___ ___ ___ ___ Interviewer ID ___ ___

Instruction: Tick all that applies; more than one answer is possible.

- O 1=Vegetables/Lettuce (cultivated on bush)
- O 2=Vegetables/Lettuce (wild harvesting from bush)
- O 3=Fruits (cultivated on bush/tree)
- O 4=Fruits (wild harvesting from bush/tree)
- O 5=Dairy products (i.e. milk, cheese, yoghurt from domesticated milk producing animals), specify _____________
- O 6=Eggs (from domesticated poultry), specify __________
- O 7=Animal blood (from domesticated animals), specify __________
- O 8=Beef/Zebu/Buffalo (domesticated), specify __________
- O 9=Pork (domesticated)
- O 10=Sheep (domesticated)
- O 11=Meat from non-domesticated animals (i.e. antelope, boar, poultry, rodents, fish), specify _________
- O 12=NO CONSUMPTION of fresh/raw food items
- O 13=Vegetables/Lettuce (cultivated on ground)
- O 14=Vegetables/Lettuce (wild harvesting from ground)
- O 15=Fruits (cultivated on ground)
- O 16=Fruits (wild harvesting from ground)
- O 17=Dairy products (i.e. milk, cheese, yoghurt from non-domesticated milk producing animals), specify _____________
- O 18=Eggs (from non-domesticated poultry), specify __________
- O 19=Animal blood (from non-domesticated animals), specify __________
- O 20=Poultry (domesticated; i.e. chicken, duck, pigeon, goose, turkey), specify __________
- O 21=Goat (domesticated)
- O 22=Rodents (domesticated), specify __________
- O 23=Fish/Seafood, specify __________
- O 24=Ice cream
- O 25=Other, specify __________
- O 99=Don't know O 98=No response

18 Do members of this household consume meals/food items/beverages usually also outside the house? If so, which member(s) of this household and where?

Instructions: Refer to question 9 of "Form 3 Part A – Demographic data/General household information" and record the initials/identifier of the member(s). Enter all codes that apply for the respective household member.

Member 1<br>Initials/Identifier: _____	Member 2<br>Initials/Identifier: _____	Member 3<br>Initials/Identifier: _____	Member 4<br>Initials/Identifier: _____
Code _____________	Code _____________	Code _____________	Code _____________
[1] Restaurant<br>[2] Fast food chain	[3] Cafeteria<br>[4] Street vendor	[5] Other, specify ____<br>[6] Not applicable	[99] Don't know<br>[98] No response

19 Which meals/food items/beverages do the member(s) of this household listed in question 18 usually consume outside the house?

Instructions: Refer to question 9 of "Form 3 Part A – Demographic data/General household information" and record the initials/identifier of the member(s). List up to 5 meals/food items/beverages per household member. You can refer to food items listed in question 17. Skip this question of no household member consumes meals/food items/beverages outside the house as stated in Q18.

Member 1<br>Initials/Identifier: _____	Member 2<br>Initials/Identifier: _____	Member 3<br>Initials/Identifier: _____	Member 4<br>Initials/Identifier: _____
1: ______________<br>2: ______________<br>3: ______________<br>4: ______________<br>5: ______________	1: ______________<br>2: ______________<br>3: ______________<br>4: ______________<br>5: ______________	1: ______________<br>2: ______________<br>3: ______________<br>4: ______________<br>5: ______________	1: ______________<br>2: ______________<br>3: ______________<br>4: ______________<br>5: ______________

WATER

20 What is the main water supply for members of this household for drinking, cooking/ preparing food/meals, bathing, and cleaning (of house/individual house structure(s), clothing/kitchen tools)?

Instruction: Enter one code per category only.

Drinking	Cooking	Bathing	Cleaning
Code _____________	Code _____________	Code _____________	Code _____________
[1] Tap water (inside the house; private)<br>[2] Tap water (outside the house i.e. back-/courtyard; private)			

For peer review only - http://bmjopen.bmj.com/site/about/guidelines.xhtml

Site ___ ___ ___ Subarea ___ ___ ___ House number ___ ___ ___ ___ Interviewer ID ___ ___

[3] Tap water (outside the house; public/shared)<br>[4] Standpipe/Piped water (inside the house; private)<br>[5] Standpipe/Piped water (outside the house i.e. back-/ courtyard; private)<br>[6] Standpipe/Piped water (outside the house; public/shared)<br>[7] Water truck (public; shared)<br>[8] Surface water (i.e. canal, irrigation canal)<br>[9] Surface water (i.e. lake, pond, river, stream)<br>[10] Spring water<br>[11] Rain water<br>[12] Well/Borehole (open/uncovered/unprotected)<br>[13] Well/Borehole (covered/protected)<br>[14] Bottled water<br>[15] Other, specify ________________ [99] Don't know [98] No response

21 Do members of this household usually treat water for drinking, cooking/ preparing food/meals, bathing, and cleaning (of house/individual house structure(s), clothing/ kitchen tools) prior to consumption? How is water usually treated?

Instruction: Enter all codes that applies per category; more than one answer per category is possible.

Drinking	Cooking	Bathing	Cleaning
Code ____________	Code ____________	Code ____________	Code ____________

[1] No, not at all	[7] No, only for children of this household
[2] No, only for sick members of this household	[8] No, only depending on the season (rainy season)
[3] No, only depending on the season (dry season)	[9] Yes, boiling
[4] Yes, filtration (i.e. cloth, ceramic, sand)	[10] Yes, solar disinfection
[5] Yes, sedimentation	[11] Yes, chlorination
[6] Other, specify ________________	[99] Don't know [98] No response

22 Which member(s) of this household usually collect(s) the drinking water as stated in question 20 for other members of this household?

Instructions: Refer to question 9 of "Form 3 Part A – Demographic data/General household information" and record the initials/identifier of the member.

O Household member, record initials/identifier(s) _________ O 99=Don't know O 98=No response

23 How do members of this household usually store the drinking water stated in question 20?

O 1=Water tank (uncovered)
O 2=Water tank (covered with i.e. cloth/fabric, lid, leaves)
O 3=Container (uncovered)
O 4=Container (covered with i.e. cloth/fabric, lid, leaves)
O 5=Clay pot (uncovered)
O 6=Clay pot (covered with i.e. cloth/fabric, lid, leaves)
O 7=Bucket (uncovered)
O 8=Bucket (covered with i.e. cloth/fabric, lid, leaves)
O 9=Bowl (uncovered)
O 10=Bowl (covered with i.e. cloth/fabric, lid, leaves)
O 11=Wooden pot (uncovered)
O 12=Wooden pot (covered with i.e. cloth/fabric, lid, leaves)
O 13=Skin vessel (i.e. goat, sheep, cattle; uncovered)
O 14=Skin vessel (i.e. goat, sheep, cattle; covered with i.e. cloth/fabric, lid, leaves)
O 15=NOT STORED
O 16=Other, specify _________
O 99=Don't know
O 98=No response

24 If members of this household use a shared drinking water supply as stated in question 20, with how many other people is this drinking water supply usually shared?

O 1=<10 people
O 2=11-24 people
O 3=25-49 people
O 4=>50 people
O 5=NOT SHARED
O 99=Don't know
O 98=No response

For peer review only - http://bmjopen.bmj.com/site/about/guidelines.xhtml

Site ___ ___ ___ Subarea ___ ___ ___ House number ___ ___ ___ ___ Interviewer ID ___ ___

WASTE DISPOSAL

25 Where do members of this household usually dispose waste water from e.g. cooking/ preparation of food/meals, bathing and/or cleaning, dispose domestic waste, human waste/excreta?

Instruction: Enter one code per category only.

Waste water from (e.g. cooking/ preparation of food/ meals, bathing and/or cleaning)	Domestic waste	Human waste/excreta
Code ____________	Code ____________	Code ____________

[1] On the ground/free range near the house (i.e. back-/courtyard, private)
[2] On the ground/free range away from the house in the community (shared, public)
[3] In a hole near the house (i.e. back-/courtyard, private)
[4] In a hole away from the house in the community (shared, public)
[5] Pour in surface water in the community (i.e. lake, pond, river, stream, public, shared)
[6] Pour in surface water in the community (i.e. canal, irrigation canal, public, shared)
[7] Pour in spring water in the community (public, shared)
[8] Pour in a septic tank near the house (i.e. back-/courtyard, near toilet facility if available, private)
[9] Pour in a septic tank in the community (shared, public)
[10] Pour in the sewer near the house (i.e. back-/courtyard, private)
[11] Pour in the sewer in the community (shared, public)
[12] Other, specify ________________________ [99] Don't know [98] No response

26 Do members of this household usually pre-process domestic waste from this house prior to disposing it?

O 1=Yes, burning garbage
O 2=Yes, separate/sort out degradable from non-degradable garbage
O 3=NO
O 4=Yes, other, specify _________
O 99=Don't know
O 98=No response

27 How far from the main drinking water supply of this household as stated in question 20 is the location for disposal of waste water, domestic waste and human waste/excreta of this household as stated in question 25?

Instructions: Enter one code per category only. Record the distance as meter [m].

Waste water from (e.g. cooking/ preparation of food/ meals, bathing and/or cleaning)	Domestic waste	Human waste/excreta
Code ____________	Code ____________	Code ____________

[1] <5 m [3] ≥25 to <50 m [5] ≥100 to <500 m [7] ≥1,000 m
[2] ≥5 to <25 m [4] ≥50 to <100 m [6] ≥500 to <1,000 m [99] Don't know [98] No response

28 How far from the main water supply for cooking/preparing food/meals of this household as stated in question 20 is the location for disposal of waste water, domestic waste and human waste/excreta of this household as stated in question 25?

Instructions: Enter one code per category only. Record the distance as meter [m].

Waste water from (e.g. cooking/preparation of food/ meals, bathing and/or cleaning)	Domestic waste	Human waste/excreta
Code ____________	Code ____________	Code ____________

[1] <5 m [3] ≥25 to <50 m [5] ≥100 to <500 m [7] ≥1,000 m
[2] ≥5 to <25 m [4] ≥50 to <100 m [6] ≥500 to <1,000 m [99] Don't know [98] No response

FARM/DOMESTIC ANIMALS

29 What kind of farm animals/livestock and domestic animals/pets do members of this household have?

Instruction: Tick all that applies per category; more than one answer per category is possible.

For peer review only - http://bmjopen.bmj.com/site/about/guidelines.xhtml

Site ___ ___ ___ Subarea ___ ___ ___ House number ___ ___ ___ ___ Interviewer ID ___ ___

Farm animals/livestock		Domestic animals/pets
O 1=Cattle	O 9=Camel	O 1=Dog
O 2=Sheep	O 10= Rodents (i.e. rabbit), specify ____________________	O 2=Rodents (i.e. rabbit), specify ____________________
O 3=Donkey	O 11=Goat	O 3=Cat
O 4=Poultry (i.e. chicken, duck, pigeon, goose, turkey), specify ______________	O 12=Horse	O 4=NO DOMESTIC ANIMALS/ PETS
O 5=Zebu	O 13=NO FARM ANIMALS/ LIVESTOCK	O 5=Other, specify ____________________
O 6=Pig	O 14=Other, specify ______________	O 99=Don't know
O 7=Fish	O 99=Don't know	O 98=No response
O 8=Buffalo	O 98=No response	

30 Where do members of this household usually keep farm animals/livestock and domestic animals/pets of this household as stated in question 29?

Instruction: Tick one answer per category only.

Farm animals/livestock	Domestic animals/pets
O 1=Stable/Cage/Water basin on the property of the house (i.e. back-/courtyard)<br>O 2=Stable/Cage/Water basin outside the house in the community<br>O 3=Stable/Cage/Water basin outside the community<br>O 4=Free range/Surface water/Spring water on the property of the house (i.e. house, back-/courtyard)<br>O 5=Free range/Surface water/Spring water outside the house in the community<br>O 6=Free range/Surface water/Spring water outside the community<br>O 7=Not applicable (nowhere/no farm animals/livestock)<br>O 8=Other, specify __________<br>O 99=Don't know<br>O 98=No response	O 1=Cage/Chain near the house (i.e. back-/courtyard)<br>O 2=Cage/Chain outside the house in the community<br>O 3=Cage/Chain outside the community (public)<br>O 4=Free range near the house (i.e. in the house, back-/courtyard)<br>O 5=Free range outside the house in the community<br>O 6=Free range outside the community<br>O 7=Not applicable (nowhere/no domestic animals/pets)<br>O 8=Other, specify __________<br>O 99=Don't know<br>O 98=No response

31 What is the extent of the livestock farming that members of this household practice?

O 1=Own demand ("backyard farming")
O 2=Not applicable (no livestock farming)
O 3=Mass/Industrial demand ("mass animal farming")
O 4=Other, specify __________
O 99=Don't know
O 98=No response

32 Which water supply do members of this household usually use to water farm animals/livestock and domestic animals/pets of this household as stated in question 29?

Instruction: Enter one code per category only.

Farm animals/livestock	Domestic animals/pets
Code _____________	Code _____________

[1] Tap water (inside the house; private)
[2] Tap water (outside the house i.e. back-/courtyard; private)
[3] Tap water (outside the house; public/shared)
[4] Standpipe/Piped water (inside the house; private)
[5] Standpipe/Piped water (outside the house i.e. back-/ courtyard; private)
[6] Standpipe/Piped water (outside the house; public/shared)

For peer review only - http://bmjopen.bmj.com/site/about/guidelines.xhtml

Site ___ ___ ___ Subarea ___ ___ ___ House number ___ ___ ___ ___ Interviewer ID ___ ___

[7] Water truck (public; shared)
[8] Surface water (i.e. canal, irrigation canal)
[9] Surface water (i.e. lake, pond, river, stream)
[10] Spring water
[11] Rain water
[12] Well/Borehole (open/uncovered/unprotected)
[13] Well/Borehole (covered/protected)
[14] Not applicable (no farm animals/livestock; no domestic animals/pets)
[15] Other, specify ___________________ [99] Don't know [98] No response

33 Do members of this household usually wash their hands after they fed & watered and cleaned the stable/cage/water basin of the farm animals/livestock and domestic animals/pets of this household as stated in question 29?
Instruction: Enter one code per category only.

Farm animals/livestock	Domestic animals/pets
Code ____________	Code ____________
[1] Wash hands with soap and water<br>[2] Wash hands with water only<br>[3] Rub hands clean with some leaves/straw/ grass<br>[4] Rub hands clean with some sand<br>[5] Rub hands clean with some cloth/fabric<br>[6] Don't clean, not needed/not dirty	[7] Don't clean, nothing to clean hands nearby/on hands<br>[8] Other, specify _________<br>[9] Not applicable (no farm animals/livestock; no domestic animals/pets)<br>[99] Don't know<br>[98] No response

CROP FARMING

34 What kind of crops do members of this household grow/cultivate?
Instruction: Tick all that applies; more than one answer is possible.

- O 1=Coffee
- O 2=Rice
- O 3=Fruits (cultivated on bush/tree)
- O 4=Fruits (cultivated on the ground)
- O 5=Vegetables/Lettuce (cultivated on bush/tree)
- O 6=Vegetables/Lettuce (cultivated on the ground)
- O 7=Grains (i.e. wheat, barley, oat, corn)
- O 8=Tea
- O 9=Cashew
- O 10=Tobacco
- O 11=Sugar cane
- O 12=Chad/Chat
- O 13=Bees/Honey
- O 14=Cotton
- O 15=NO CROPS
- O 16=Other, specify __________
- O 99=Don't know
- O 98=No response

35 Where do members of this household have the farmland/agricultural land for crop farming?

- O 1=On the property of the house (i.e. back-/courtyard)
- O 4=Outside the house in the community
- O 2=Outside the community
- O 5=Not applicable (no crop farming)
- O 3=Other, specify __________
- O 99=Don't know
- O 98=No response

36 What is the extent of the crop farming that members of this household practice?

- O 1=Own demand ("backyard farming")
- O 2=Not applicable (no crop farming)
- O 3=Mass/Industrial demand ("mass crop farming")
- O 4=Other, specify __________
- O 99=Don't know
- O 98=No response

37 Do members of this household usually use herbicides, pesticides and fertilizers for the crop farming as stated in question 34?
Instruction: Tick one answer per category only.

Herbicides	Pesticides	Fertilizers
O 1=Yes, specify herbicide ________________	O 1=Yes, specify pesticide ________________	O 1=Yes, specify fertilizer ________________

For peer review only - http://bmjopen.bmj.com/site/about/guidelines.xhtml

Site ___ ___ ___ Subarea ___ ___ ___ House number ___ ___ ___ ___ Interviewer ID ___ ___

O 2=Not applicable (no crop farming)<br>O 3=No use of herbicides<br>O 99=Don't know<br>O 98=No response	O 2=Not applicable (no crop farming)<br>O 3=No use of pesticides<br>O 99=Don't know<br>O 98=No response	O 2=Not applicable (no crop farming)<br>O 3=No use of fertilizers<br>O 99=Don't know<br>O 98=No response

38 Do members of this household usually wash their hands after the fieldwork for the crop farming as stated in question 34?

O 1=Yes, wash hands with soap and water
O 2=Yes, rub hands clean with some leaves/straw/grass
O 3=Yes, rub hands clean with some cloth/fabric
O 4=Not applicable (no crop farming)
O 5=Yes, wash hands with water only
O 6=Yes, rub hands clean with some sand
O 7=No, don't clean my hands
O 8=Other, specify __________
O 99=Don't know
O 98=No response

For peer review only

For peer review only - http://bmjopen.bmj.com/site/about/guidelines.xhtml

Site ___ ___ ___ Subarea ___ ___ ___ House number ___ ___ ___ ___ Interviewer ID ___ ___

FORM 4: ASSESSMENT OF OVERALL, AGE- AND SEX-STRATIFIED GENERIC HEALTHCARE UTILIZATION

Part A – Assessment of *generic* healthcare utilization

Instructions to the interviewer

– Complete this form only if the respondent consented to participate (see "Form 2 - CONSENT FORM"); the respondent as defined for this investigation is an adult (at the country-specific legal age of majority) household member and decision-maker with respect to daily healthcare/healthcare utilization for the entire household and its members; it must not be e.g. a neighbour or a visitor.
– Refer to "Form 3 – Part A - GENERAL HOUSEHOLD INFORMATION" as a reference to obtain the total number of household members as well as age and sex of each household member.
– Collect data about all household members regardless of whether all household members are present at the time of the interview; collecting and recording data only about individuals present at the time of the interview will result in biased data.
– A household is/household members are defined as a person or a group of related or unrelated persons that live together in the same dwelling unit, that acknowledge one male or female adult as the head of the household, that share the same housekeeping arrangements, that are considered to constitute one unit, and that provide themselves with food or other essentials for living.
– "Site" and "Subarea" must be entered each as a 3-digit, "House number" as a 4-digit, and "Interviewer ID" as a 2-digit code (use lists individually prepared for each site to obtain codes). "Study label" must be a combination of a 3-digit "Site" code, a 3-digit "Subarea" code and a 4-digit "House number" code; the "Interviewer ID" must be entered as a 3-digit code.
– Abbreviation: HCF=private/public primary/secondary/tertiary healthcare facility.

HOUSEHOLD MEMBERS

1 Record the total number of household members of this house (at the day of visit)**.**
Instructions: The total number of household members must be recorded as a 2-digit number; see also "Form 3 – Part A - GENERAL HOUSEHOLD INFORMATION".

Total number of household members: ____ ____

2 Record the number of FEMALE household members by each of the following age groups:
Instructions: The total number of female household members by age group must be recorded as a 2-digit number; see also "Form 3 – Part A - GENERAL HOUSEHOLD INFORMATION".

<2years: ____ ____ ≥2 to <5years: ____ ____ ≥5 to <15years: ____ ____ ≥15years: ____ ____

3 Record the number of MALE household members by each of the following age groups:
Instructions: The total number of male household members by age group must be recorded as a 2-digit number; see also "Form 3 – Part A - GENERAL HOUSEHOLD INFORMATION".

<2years: ____ ____ ≥2 to <5years: ____ ____ ≥5 to <15years: ____ ____ ≥15years: ____ ____

HEALTHCARE SEEKING BEHAVIOR

4 Record where female/male household members <2years/≥2 to <5years/≥5 to <15years/≥15years of age usually seek healthcare for the following signs/symptoms. Please rate using a range from 1-4.

Instructions:
- Complete question 4 separately for female and male household members <2years, ≥2 to <5years, ≥5 to <15years and ≥15years of age and circle the sex and age group Q4 was completed for; skip question 4 if there is no female/male household member <2years/ ≥2 to <5years/ ≥5 to <15years/ ≥15years of age.
- Provide an answer to each healthcare option for each sign/symptom; use range 1-4 for rating (1=1st choice; 2=2nd choice; 3=3rd choice; 4=not chosen); enter a code where applicable by using prepared lists (use lists individually prepared for each site to obtain codes); tick "Don't know" if applicable.

For peer review only

For peer review only - http://bmjopen.bmj.com/site/about/guidelines.xhtml

Site ___ ___ ___ Subarea ___ ___ ___ House number ___ ___ ___ ___ Interviewer ID ___ ___

Sign/Symptom	Healthcare options									
	Study-HCF		Other HCF		Physician	Pharmacy	Traditional healer	Nowhere Self-treatment	Nowhere Nothing	Don't know
	Rate	Code	Rate	Code	Rate	Rate	Rate	Rate	Rate	Tick
**1** [**A**] Fever, any	O=1<br>O=2<br>O=3<br>O=4		O=1<br>O=2<br>O=3<br>O=4		O=1<br>O=2<br>O=3<br>O=4	O=1<br>O=2<br>O=3<br>O=4	O=1<br>O=2<br>O=3<br>O=4	O=1<br>O=2<br>O=3<br>O=4	O=1<br>O=2<br>O=3<br>O=4	O
**2** Fever <3days (continuous)	O=1<br>O=2<br>O=3<br>O=4		O=1<br>O=2<br>O=3<br>O=4		O=1<br>O=2<br>O=3<br>O=4	O=1<br>O=2<br>O=3<br>O=4	O=1<br>O=2<br>O=3<br>O=4	O=1<br>O=2<br>O=3<br>O=4	O=1<br>O=2<br>O=3<br>O=4	O
**3** Fever ≥3days (continuous)	O=1<br>O=2<br>O=3<br>O=4		O=1<br>O=2<br>O=3<br>O=4		O=1<br>O=2<br>O=3<br>O=4	O=1<br>O=2<br>O=3<br>O=4	O=1<br>O=2<br>O=3<br>O=4	O=1<br>O=2<br>O=3<br>O=4	O=1<br>O=2<br>O=3<br>O=4	O
**4** Chills Shivering	O=1<br>O=2<br>O=3<br>O=4		O=1<br>O=2<br>O=3<br>O=4		O=1<br>O=2<br>O=3<br>O=4	O=1<br>O=2<br>O=3<br>O=4	O=1<br>O=2<br>O=3<br>O=4	O=1<br>O=2<br>O=3<br>O=4	O=1<br>O=2<br>O=3<br>O=4	O
**5** Convulsion	O=1<br>O=2<br>O=3<br>O=4		O=1<br>O=2<br>O=3<br>O=4		O=1<br>O=2<br>O=3<br>O=4	O=1<br>O=2<br>O=3<br>O=4	O=1<br>O=2<br>O=3<br>O=4	O=1<br>O=2<br>O=3<br>O=4	O=1<br>O=2<br>O=3<br>O=4	O
**6** Weight loss	O=1<br>O=2<br>O=3<br>O=4		O=1<br>O=2<br>O=3<br>O=4		O=1<br>O=2<br>O=3<br>O=4	O=1<br>O=2<br>O=3<br>O=4	O=1<br>O=2<br>O=3<br>O=4	O=1<br>O=2<br>O=3<br>O=4	O=1<br>O=2<br>O=3<br>O=4	O
**7** Dehydration	O=1<br>O=2<br>O=3<br>O=4		O=1<br>O=2<br>O=3<br>O=4		O=1<br>O=2<br>O=3<br>O=4	O=1<br>O=2<br>O=3<br>O=4	O=1<br>O=2<br>O=3<br>O=4	O=1<br>O=2<br>O=3<br>O=4	O=1<br>O=2<br>O=3<br>O=4	O
**8** Malaise Weakness/ Fatigue	O=1<br>O=2<br>O=3<br>O=4		O=1<br>O=2<br>O=3<br>O=4		O=1<br>O=2<br>O=3<br>O=4	O=1<br>O=2<br>O=3<br>O=4	O=1<br>O=2<br>O=3<br>O=4	O=1<br>O=2<br>O=3<br>O=4	O=1<br>O=2<br>O=3<br>O=4	O
**9** Aches/ Pain (muscle/joint/bone)	O=1<br>O=2<br>O=3<br>O=4		O=1<br>O=2<br>O=3<br>O=4		O=1<br>O=2<br>O=3<br>O=4	O=1<br>O=2<br>O=3<br>O=4	O=1<br>O=2<br>O=3<br>O=4	O=1<br>O=2<br>O=3<br>O=4	O=1<br>O=2<br>O=3<br>O=4	O
**10** Headache	O=1<br>O=2<br>O=3<br>O=4		O=1<br>O=2<br>O=3<br>O=4		O=1<br>O=2<br>O=3<br>O=4	O=1<br>O=2<br>O=3<br>O=4	O=1<br>O=2<br>O=3<br>O=4	O=1<br>O=2<br>O=3<br>O=4	O=1<br>O=2<br>O=3<br>O=4	O
**11** Dizziness/ Confusion Unconsciousness	O=1<br>O=2<br>O=3<br>O=4		O=1<br>O=2<br>O=3<br>O=4		O=1<br>O=2<br>O=3<br>O=4	O=1<br>O=2<br>O=3<br>O=4	O=1<br>O=2<br>O=3<br>O=4	O=1<br>O=2<br>O=3<br>O=4	O=1<br>O=2<br>O=3<br>O=4	O
**12** Blood pressure disorders	O=1<br>O=2<br>O=3<br>O=4		O=1<br>O=2<br>O=3<br>O=4		O=1<br>O=2<br>O=3<br>O=4	O=1<br>O=2<br>O=3<br>O=4	O=1<br>O=2<br>O=3<br>O=4	O=1<br>O=2<br>O=3<br>O=4	O=1<br>O=2<br>O=3<br>O=4	O

For peer review only - http://bmjopen.bmj.com/site/about/guidelines.xhtml

Site ___ ___ ___ Subarea ___ ___ ___ House number ___ ___ ___ ___ Interviewer ID ___ ___

Sign/Symptom	Healthcare options									
	Study-HCF		Other HCF		Physician	Pharmacy	Traditional healer	Nowhere Self-treatment	Nowhere Nothing	Don't know
	Rate	Code	Rate	Code	Rate	Rate	Rate	Rate	Rate	Code
**13** Heart disorders	O=1<br>O=2<br>O=3<br>O=4		O=1<br>O=2<br>O=3<br>O=4		O=1<br>O=2<br>O=3<br>O=4	O=1<br>O=2<br>O=3<br>O=4	O=1<br>O=2<br>O=3<br>O=4	O=1<br>O=2<br>O=3<br>O=4	O=1<br>O=2<br>O=3<br>O=4	O
**14** Nausea Vomitting	O=1<br>O=2<br>O=3<br>O=4		O=1<br>O=2<br>O=3<br>O=4		O=1<br>O=2<br>O=3<br>O=4	O=1<br>O=2<br>O=3<br>O=4	O=1<br>O=2<br>O=3<br>O=4	O=1<br>O=2<br>O=3<br>O=4	O=1<br>O=2<br>O=3<br>O=4	O
**15** Diarrhea (watery/ bloody/ mucopurulent)	O=1<br>O=2<br>O=3<br>O=4		O=1<br>O=2<br>O=3<br>O=4		O=1<br>O=2<br>O=3<br>O=4	O=1<br>O=2<br>O=3<br>O=4	O=1<br>O=2<br>O=3<br>O=4	O=1<br>O=2<br>O=3<br>O=4	O=1<br>O=2<br>O=3<br>O=4	O
**16** Abdominal pain Intestinal pain	O=1<br>O=2<br>O=3<br>O=4		O=1<br>O=2<br>O=3<br>O=4		O=1<br>O=2<br>O=3<br>O=4	O=1<br>O=2<br>O=3<br>O=4	O=1<br>O=2<br>O=3<br>O=4	O=1<br>O=2<br>O=3<br>O=4	O=1<br>O=2<br>O=3<br>O=4	O
**17** Blood loss/ Bleeding (internal)	O=1<br>O=2<br>O=3<br>O=4		O=1<br>O=2<br>O=3<br>O=4		O=1<br>O=2<br>O=3<br>O=4	O=1<br>O=2<br>O=3<br>O=4	O=1<br>O=2<br>O=3<br>O=4	O=1<br>O=2<br>O=3<br>O=4	O=1<br>O=2<br>O=3<br>O=4	O
**18** Blood loss/ Bleeding (external)	O=1<br>O=2<br>O=3<br>O=4		O=1<br>O=2<br>O=3<br>O=4		O=1<br>O=2<br>O=3<br>O=4	O=1<br>O=2<br>O=3<br>O=4	O=1<br>O=2<br>O=3<br>O=4	O=1<br>O=2<br>O=3<br>O=4	O=1<br>O=2<br>O=3<br>O=4	O
**19** Sneezing Runny nose	O=1<br>O=2<br>O=3<br>O=4		O=1<br>O=2<br>O=3<br>O=4		O=1<br>O=2<br>O=3<br>O=4	O=1<br>O=2<br>O=3<br>O=4	O=1<br>O=2<br>O=3<br>O=4	O=1<br>O=2<br>O=3<br>O=4	O=1<br>O=2<br>O=3<br>O=4	O
**20** Cough	O=1<br>O=2<br>O=3<br>O=4		O=1<br>O=2<br>O=3<br>O=4		O=1<br>O=2<br>O=3<br>O=4	O=1<br>O=2<br>O=3<br>O=4	O=1<br>O=2<br>O=3<br>O=4	O=1<br>O=2<br>O=3<br>O=4	O=1<br>O=2<br>O=3<br>O=4	O
**21** Rapid breathing/ shortness of breath	O=1<br>O=2<br>O=3<br>O=4		O=1<br>O=2<br>O=3<br>O=4		O=1<br>O=2<br>O=3<br>O=4	O=1<br>O=2<br>O=3<br>O=4	O=1<br>O=2<br>O=3<br>O=4	O=1<br>O=2<br>O=3<br>O=4	O=1<br>O=2<br>O=3<br>O=4	O
**22** Severe breathing/ Difficulties in breathing	O=1<br>O=2<br>O=3<br>O=4		O=1<br>O=2<br>O=3<br>O=4		O=1<br>O=2<br>O=3<br>O=4	O=1<br>O=2<br>O=3<br>O=4	O=1<br>O=2<br>O=3<br>O=4	O=1<br>O=2<br>O=3<br>O=4	O=1<br>O=2<br>O=3<br>O=4	O
**23** Skin inflammation (rashes/ redness/ pus/ itching)	O=1<br>O=2<br>O=3<br>O=4		O=1<br>O=2<br>O=3<br>O=4		O=1<br>O=2<br>O=3<br>O=4	O=1<br>O=2<br>O=3<br>O=4	O=1<br>O=2<br>O=3<br>O=4	O=1<br>O=2<br>O=3<br>O=4	O=1<br>O=2<br>O=3<br>O=4	O
**24** Swelling/ Edema	O=1<br>O=2<br>O=3<br>O=4		O=1<br>O=2<br>O=3<br>O=4		O=1<br>O=2<br>O=3<br>O=4	O=1<br>O=2<br>O=3<br>O=4	O=1<br>O=2<br>O=3<br>O=4	O=1<br>O=2<br>O=3<br>O=4	O=1<br>O=2<br>O=3<br>O=4	O
**25** Jaundice	O=1<br>O=2<br>O=3<br>O=4		O=1<br>O=2<br>O=3<br>O=4		O=1<br>O=2<br>O=3<br>O=4	O=1<br>O=2<br>O=3<br>O=4	O=1<br>O=2<br>O=3<br>O=4	O=1<br>O=2<br>O=3<br>O=4	O=1<br>O=2<br>O=3<br>O=4	O

For peer review only - http://bmjopen.bmj.com/site/about/guidelines.xhtml

Site ___ ___ ___ Subarea ___ ___ ___ House number ___ ___ ___ ___ Interviewer ID ___ ___

FORM 4: ASSESSMENT OF OVERALL, AGE- AND SEX-STRATIFED GENERIC HEALTHCARE UTILIZATION

Part B – Healthcare-associated factors/Vaccination/Disease perception

TRAVEL HEALTHCARE FACILITY

1 In case a healthcare facility (HCF) was rated the 1st choice of care in question 4A (fever), what is the main mode of transportation, travel time (depending on mode of transportation), travel cost (depending on mode of transportation) and travel distance (depending on mode of transportation) that is usually taken to get there?

Instructions: Enter one code per category and sex as well as age group only. Skip this question if there is no female/male member <2years/≥2 to <5years/≥5 to <15years/≥15years of age in this household (see also question 2 and 3 of this form PartA). Skip this question if no HCF was rated the 1st choice of care in question 4A for female/male member <2years/≥2 to <5years/≥5 to <15years/≥15years of age. Record the travel time as minute(s) [min]/ hour(s) [hr], the total costs in USD, including potential gifts, favours and tips, and the total distance as meter(s) [m]/kilometre(s) [km].

Female				Male			
<2 years	≥2 to <5 years	≥5 to <15 years	≥15 years	<2 years	≥2 to <5 years	≥5 to <15 years	≥15 years
**1.1 Transportation mode**							
Code ___	Code ____	Code ____	Code ___	Code ___	Code ____	Code ____	Code ___
[1] Private transportation (i.e. motorbike, car, bus) [2] Public transportation (i.e. motorbike, car, bus)				[3] Walking [4] Bicycle	[5] Other, specify _________ [99] Don't know		[98] No response
**1.2 Travel time**							
Code ___	Code ____	Code ____	Code ___	Code ___	Code ____	Code ____	Code ___
[1] <30 mins [2] ≥30 to <60 mins		[3] ≥1.0 to <2.0 hrs [4] ≥2.0 to <3.0 hrs		[5] ≥3.0 to <4.0 hrs [6] ≥4.0 hrs		[99] Don't know [98] No response	
**1.3 Travel cost**							
Code ___	Code ____	Code ____	Code ___	Code ___	Code ____	Code ____	Code ___
[1] <1.0 USD [2] ≥1.0 to <3.0 USD		[3] ≥3.0 to <5.0 USD [4] ≥5.0 to <10.0 USD		[5] ≥10.0 USD		[99] Don't know [98] No response	
**1.4 Travel distance**							
Code ___	Code ____	Code ____	Code ___	Code ___	Code ____	Code ____	Code ___
[1] <500 m [2] ≥500 m to <1.0 km		[3] ≥1.0 to <2.0 km [4] ≥2.0 to <3.0 km		[5] ≥3.0 to <4.0 km [6] ≥4.0 km		[99] Don't know [98] No response	

INSURANCE

2 For which members of this household does the insurance cover health expenses?

Instructions: Enter one code per category. Skip this question if there is no member <2years/≥2 to <5years/≥5 to <15years/≥15years of age in this household (see question 2 and 3 of this form PartA).

<2 years	≥2 to <5 years	≥5 to <15 years	≥15 years
Code ____________	Code ____________	Code ____________	Code ____________
[1] Yes	[2] No	[99] Don't know	[98] No response

For peer review only - http://bmjopen.bmj.com/site/about/guidelines.xhtml

Site ___ ___ ___ Subarea ___ ___ ___ House number ___ ___ ___ ___ Interviewer ID ___ ___

3 What kind of health expenses are usually covered by the insurance for members of this household?

Instructions: Enter one code by category and age group only. Skip this question if there is no member <2years/≥2 to <5years/≥5 to <15years/≥15years of age in this household (see also question 2 and 3 of this form PartA). Skip this question if there is no insurance that covers health expenses for <2years/≥2 to <5years/≥5 to <15years/≥15years of age.

	<2 years	≥2 to <5years	≥5 to <15years	≥15years
	[1] Yes	[2] No	[99] Don't know	[98] No response
**1** Registration	Code ______	Code _______	Code _______	Code _____
**2** Consultation/ Doctor's visit	Code ______	Code _______	Code _______	Code _____
**3** Physical examination - general	Code ______	Code _______	Code _______	Code _____
**4** Physical examination – specific, specify_______	Code ______	Code _______	Code _______	Code _____
**5** Diagnostics - general laboratory diagnostics	Code ______	Code _______	Code _______	Code _____
**6** Diagnostics - specific laboratory diagnostics, specify__________	Code ______	Code _______	Code _______	Code _____
**7** Diagnostics - general imaging	Code ______	Code _______	Code _______	Code _____
**8** Diagnostics - specific imaging, specify__________	Code ______	Code _______	Code _______	Code _____
**9** Diagnostics - other, specify________	Code ______	Code _______	Code _______	Code _____
**10** Treatment - general medication	Code ______	Code _______	Code _______	Code _____
**11** Treatment - specific medication, specify________	Code ______	Code _______	Code _______	Code _____
**12** Treatment - other medication, specify__________	Code ______	Code _______	Code _______	Code _____
**13** Treatment - interventions (other than surgery), specify_______	Code ______	Code _______	Code _______	Code _____
**14** Treatment Surgery	Code ______	Code _______	Code _______	Code _____
**15** Hospitalization <7days	Code ______	Code _______	Code _______	Code _____
**16** Hospitalization ≥7days	Code ______	Code _______	Code _______	Code _____
**17** Emergency services	Code ______	Code _______	Code _______	Code _____
**18** Antenatal care	Code ______	Code _______	Code _______	Code _____
**19** Postnatal care	Code ______	Code _______	Code _______	Code _____
**20** Preventive medicine	Code ______	Code _______	Code _______	Code _____
**21** Vaccination	Code ______	Code _______	Code _______	Code _____
**22** Home healthcare (i.e. elderly, disabled, chronic/long-term diseased)	Code ______	Code _______	Code _______	Code _____
**23** Supportive therapy (i.e. physiotherapy, ergotherapy, rehabilitation)	Code ______	Code _______	Code _______	Code _____

For peer review only

Site ___ ___ ___ Subarea ___ ___ ___ House number ___ ___ ___ ___ Interviewer ID ___ ___

ETHNOGRAPHY

4 To which religion/ethnic group/tribe belong members of this household?

Instructions: Give one answer by age group only. Skip this question if there is no member >2years/≥2 to <5years/≥5 to <15years/≥15years of age in this household (see also question 2 and 3 of this form PartA).

	<2 years	≥2 to <5years	≥5 to <15years	≥15years
Religion/Ethnic group/Tribe	______________	______________	______________	______________
	O 99=Don't know<br>O 98=No response	O 99=Don't know<br>O 98=No response	O 99=Don't know<br>O 98=No response	O 99=Don't know<br>O 98=No response

5 Does the religion/ethnic group/tribe as stated in question 4 influence the various aspects of utilization of healthcare for members of this household?

Instructions: Enter one code by category and age group only. Skip this question if there is member >2years/≥2 to <5years/≥5 to <15years/≥15years of age in this household (see also question 2 and 3 of this form PartA).

	<2 years	≥2 to <5years	≥5 to <15years	≥15years
	[1] Yes	[2] No	[99] Don't know	[98] No response
**1** Visiting a healthcare facility (i.e. hospital, health care center, health post)	Code _____	Code _______	Code _______	Code _____
**2** Visiting a physician	Code _____	Code _______	Code _______	Code _____
**3** Visiting a pharmacy	Code _____	Code _______	Code _______	Code _____
**4** Visiting a traditional healer	Code _____	Code _______	Code _______	Code _____
**5** Deciding for self-treatment	Code _____	Code _______	Code _______	Code _____
**6** Deciding for non-treatment	Code _____	Code _______	Code _______	Code _____
**7** Physical examination – general	Code _____	Code _______	Code _______	Code _____
**8** Physical examination – specific, specify________	Code _____	Code _______	Code _______	Code _____
**9** Collection of specimen (i.e. blood, urine, stool, smear, swab, cerebrospinal fluid)	Code _____	Code _______	Code _______	Code _____
**10** Diagnostics – general laboratory diagnostics	Code _____	Code _______	Code _______	Code _____
**11** Diagnostics – specific laboratory diagnostics, specify___________	Code _____	Code _______	Code _______	Code _____
**12** Diagnostics - general imaging	Code _____	Code _______	Code _______	Code _____
**13** Diagnostics - specific imaging, specify_________	Code _____	Code _______	Code _______	Code _____
**14** Diagnostics - other, specify_________	Code _____	Code _______	Code _______	Code _____
**15** Treatment - general medication	Code _____	Code _______	Code _______	Code _____
**16** Treatment - specific medication, specify________	Code _____	Code _______	Code _______	Code _____
**17** Treatment - other medication, specify___________	Code _____	Code _______	Code _______	Code _____

For peer review only - http://bmjopen.bmj.com/site/about/guidelines.xhtml

Site ___ ___ ___ Subarea ___ ___ ___ House number ___ ___ ___ ___ Interviewer ID ___ ___

**18** Treatment - interventions (other than surgery), specify________________	Code _____	Code _______	Code _______	Code _____
**19** Treatment - Surgery	Code _____	Code _______	Code _______	Code _____
**20** Hospitalization <7days	Code _____	Code _______	Code _______	Code _____
**21** Hospitalization ≥7days	Code _____	Code _______	Code _______	Code _____
**22** Emergency services	Code _____	Code _______	Code _______	Code _____
**23** Antenatal care	Code _____	Code _______	Code _______	Code _____
**24** Postnatal care	Code _____	Code _______	Code _______	Code _____
**25** Preventive medicine	Code _____	Code _______	Code _______	Code _____
**26** Vaccination	Code _____	Code _______	Code _______	Code _____
**27** Home healthcare (i.e. elderly, disabled, chronic/long-term diseased)	Code _____	Code _______	Code _______	Code _____
**28** Supportive therapy (i.e. physiotherapy, ergotherapy, rehabilitation)	Code _____	Code _______	Code _______	Code _____

VACCINATION

6 How were children of this household fed during the first six months of life?

Instructions: Skip this question if there are no male/female children in this household (see also question 2 and 3 of this form PartA).

- O 1=Breastfed exclusively
- O 2=Breastfed and other feeds, specify ______
- O 3=Formula and other feeds, specify ________
- O 4=Breastfed and formula
- O 5=Formula exclusively
- O 6=Other, specify ________________
- O 99=Don't know
- O 98=No response

7 Which water source was usually used to prepare formula or formula and other feeds as stated in question 6 for children of this household?

Instructions: Skip this question if there are no children in this household (see also question 2 and 3 of this form PartA).

- O 1=Tap water (inside the house; private)
- O 2=Tap water (outside the house i.e. back-/courtyard; private)
- O 3=Tap water (outside the house; public, shared)
- O 4=Standpipe/Piped water (inside the house; private)
- O 5=Standpipe/Piped water (outside the house i.e. back-/courtyard; private)
- O 6=Standpipe/Piped water (outside the house; public, shared)
- O 7=Water truck (public; shared)
- O 8=Bottled water
- O 9=Surface water (i.e. lake, pond, river, stream)
- O 10=Surface water (i.e. canal, irrigation canal)
- O 11=Spring water
- O 12=Rain water
- O 13=Well/Borehole (open/uncovered/unprotected)
- O 14=Well/Borehole (covered/protected)
- O 15=NO FORMULA/OTHER FEEDS PREPARED
- O 16=Other, specify ____________
- O 99=Don't know
- O 98=No response

8 Did children of this household receive any vaccines since birth, including the birth vaccines?

Instructions: Enter one code by category and child only. Skip this question if there are no children in this household (see also question 2 and 3 of this form PartA). Refer to question 9 of "Form 3 Part A – Demographic data/General household information" and record the initials/identifier of the member(s).

Child 1	Child 2	Child 3	Child 4
Initials/Identifier: _____	Initials/Identifier: _____	Initials/Identifier: _____	Initials/Identifier: _____

For peer review only - http://bmjopen.bmj.com/site/about/guidelines.xhtml

Site ___ ___ ___ Subarea ___ ___ ___ House number ___ ___ ___ ___ Interviewer ID ___ ___

[1] YES, vaccinated<br>[2] No, caretaker busy<br>[3] No, caretaker decided no need<br>[4] No, unknown where to go	[5] No, lack of vaccine<br>[6] No, child too old/young<br>[7] No, caretaker refused/too worried<br>[8] No, other, specify ____________	[9] No, HCF busy<br>[10] No, child sick<br>[11] No, no vaccination card<br>[99] Don't know	[98] No response
**8.1 Diphtheria and Tetanus and Pertussis and *Haemophilus influenza* and Hepatitis B vaccine/DTwPHibHepB** (e.g. 6/8, 10/12, 14/16 weeks)			
Code ___________	Code ___________	Code ___________	Code ___________
**8.2 Tetanus toxoid (TT)** (e.g. 1st contact pregnancy, 1, 6, 12/24 months)			
Code ___________	Code ___________	Code ___________	Code ___________
**8.3 Tetanus and diphtheria toxoid (TD)** (e.g. 1st contact pregnancy, 1, 6, 12 months)			
Code ___________	Code ___________	Code ___________	Code ___________
**8.4 Tuberculosis/BCG** (at birth)			
Code ___________	Code ___________	Code ___________	Code ___________
**8.5 Rotavirus/RV** (e.g. 6/8, 10/12, 16 weeks)			
Code ___________	Code ___________	Code ___________	Code ___________
**8.6 Measles/Measles and Rubella (MR)** (e.g. 9, 15/18months)			
Code ___________	Code ___________	Code ___________	Code ___________
**8.7 Polio/OPV** (e.g. birth, 6/8, 10/12, 14/16 weeks)			
Code ___________	Code ___________	Code ___________	Code ___________
**8.8 Pneumococcus/PCV** (e.g. 6/8, 10/12, 14/16 weeks)			
Code ___________	Code ___________	Code ___________	Code ___________
**8.9 Yellow fever (YF)** (e.g. 9 months)			
Code ___________	Code ___________	Code ___________	Code ___________
**8.10 Other Vaccines**			
Specify, ___________	Specify, ___________	Specify, ___________	Specify, ___________
Specify, ___________	Specify, ___________	Specify, ___________	Specify, ___________
Specify, ___________	Specify, ___________	Specify, ___________	Specify, ___________
Specify, ___________	Specify, ___________	Specify, ___________	Specify, ___________

For peer review only - http://bmjopen.bmj.com/site/about/guidelines.xhtml

Site ___ ___ ___ Subarea ___ ___ ___ House number ___ ___ ___ ___ Interviewer ID ___ ___

DISEASE PERCEPTION

9 Knowledge, perception, causes and prevention of common infectious diseases

**Disease**	**9.1 Disease known by household members?** [1] Yes [2] No [99] Don't know [98] No response	**9.2 Perception of disease by household members?** *Instruction:* Describe briefly if known.	**9.3 Causes for disease known by household members?** *Instructions:* Enter all codes (list A) that apply; more than one answer is possible.	**9.4 Measures to prevent disease known by household members?** *Instructions:* Enter all codes (list B) that apply; more than one answer is possible.	**9.5 Measures to prevent disease utilized by household members?** *Instructions:* Enter all codes (list B) that apply; more than one answer is possible.
**A.** Typhoid fever	Code __________		Code __________	Code __________	Code __________
**B.** Cholera	Code __________		Code __________	Code __________	Code __________
**C.** Malaria	Code __________		Code __________	Code __________	Code __________
**D.** Influenza	Code __________		Code __________	Code __________	Code __________
**E.** Meningitis	Code __________		Code __________	Code __________	Code __________
**F.** Hepatitis E	Code __________		Code __________	Code __________	Code __________
**G.** Tuberculosis	Code __________		Code __________	Code __________	Code __________
**H.** HIV/AIDS	Code __________		Code __________	Code __________	Code __________

List A: Possible causes	List B: Possible preventive measures
[1] Alcohol consumption<br>[2] Weather/witchcraft<br>[3] Mosquito/insect bite<br>[4] Dog bite	[1] No alcohol consumption<br>[2] Sleeping under bed net<br>[3] Using mosquito/insect spray<br>[4] Maintain a clean house and immediate surroundings of the house

For peer review only - http://bmjopen.bmj.com/site/about/guidelines.xhtml

Site ___ ___ ___ Subarea ___ ___ ___ House number ___ ___ ___ ___ Interviewer ID ___ ___

[5] Drinking untreated surface/spring water (i.e. lake, pond, river, stream, canal/irrigation canal)<br>[6] Close body contact/kissing/hugging<br>[7] Eating raw food items such as vegetables/lettuce, fruits, dairy products, eggs, meat or fish<br>[8] Not cleaning hands properly when preparing food/meals<br>[9] Not cleaning hands properly before/after visiting a toilet<br>[10] Contact to human blood (i.e. injection, transfusion, surgery)<br>[11] Consuming raw animal blood<br>[12] Other, specify _______________<br>[99] Don't know<br>[98] No response	[5] Practice proper disposal of human and domestic waste<br>[6] Avoiding close contact to dogs<br>[7] Treating water (i.e. boiling, filtration, solar disinfection, sedimentation, chlorination)<br>[8] Not cooking/preparing food/meals with untreated water<br>[9] Not washing food items to be consumed raw with untreated water<br>[10] Re-boiling/re-heating left-over/prepared food/meals prior to consumption<br>[11] Not eating raw food items such as vegetables/lettuce, fruits, dairy products, eggs, meat or fish<br>[12] Cover food items/meals<br>[13] Protect food items/meals from flies<br>[14] Avoid food/beverage from street vendors<br>[15] Avoid close contact to sick people<br>[16] No close body contact/kissing/hugging<br>[17] Covering nose/mouth while sneezing/coughing<br>[18] No contact/consumption of human/animal blood<br>[19] Practicing proper hand hygiene when preparing food/meals (i.e. washing hands with soap and water, rub hands clean)<br>[20] Practicing proper hand hygiene before/after visiting a toilet (i.e. washing hands with soap and water, rub hands clean)<br>[21] Other, specify ____________________<br>[99] Don't know<br>[98] No response

For peer review only

For peer review only - http://bmjopen.bmj.com/site/about/guidelines.xhtml

Site ___ ___ ___ Subarea ___ ___ ___ House number ___ ___ ___ ___ Interviewer ID ___ ___

10 For which members of this household are the preventive measures as listed in question 9.5 against typhoid fever used?

Instructions: Tick all that applies; more than one answer is possible.

- O 1=<2years
- O 2=≥2 to < 5years
- O 3=≥5 to <15years
- O 4=≥15years
- O 5=NOT USED
- O 99=Don't know
- O 98=No response

11 Name the household members that were diagnosed with typhoid fever during the past 3 months. Which diagnostics were used to confirm typhoid fever?

Instructions: Enter all codes that apply for each household member; more than one answer is possible.

Member 1<br>Initials/Identifier: ______	Member 2<br>Initials/Identifier: ______	Member 3<br>Initials/Identifier: ______	Member 4<br>Initials/Identifier: ______
[1] Blood culture<br>[2] Stool culture	[3] Blood/Widal test<br>[4] Other test, specify______________	[5] Not applicable (not diagnosed with typhoid fever)<br>[99] Don't know	[98] No response
Code ___________	Code ___________	Code ___________	Code ___________

12 (Where were members of this household recorded in question 11 diagnosed for typhoid fever?) Where did members of this household seek care?

Instructions: Enter one code by household member only.

Member 1<br>Initials/Identifier: ______	Member 2<br>Initials/Identifier: ______	Member 3<br>Initials/Identifier: ______	Member 4<br>Initials/Identifier: ______
[1] Healthcare facility, specify ____________<br>[2] Physician<br>[3] Nowhere, self-treatment<br>[4] Traditional healer<br>[5] Pharmacy		[6] Nowhere, did not seek care<br>[7] Other, specify ____________<br>[8] Not applicable<br>[99] Don't know<br>[98] No response	
Code ___________	Code ___________	Code ___________	Code ___________

13 (Did members of this household that were diagnosed with typhoid fever as recorded in question 11 get any treatment?) Which treatment did they get?

Instructions: Tick one answer by household member only.

Member 1<br>Initials/Identifier: ______	Member 2<br>Initials/Identifier: ______	Member 3<br>Initials/Identifier: ______	Member 4<br>Initials/Identifier: ______
O 1=Treatment, specify<br>_______________<br>O 2=Not applicable<br>O 99=Don't know<br>O 98=No response	O 1=Treatment, specify<br>_______________<br>O 2=Not applicable<br>O 99=Don't know<br>O 98=No response	O 1=Treatment, specify<br>_______________<br>O 2=Not applicable<br>O 99=Don't know<br>O 98=No response	O 1=Treatment, specify<br>_______________<br>O 2=Not applicable<br>O 99=Don't know<br>O 98=No response

For peer review only - http://bmjopen.bmj.com/site/about/guidelines.xhtml

Site ___ ___ ___ Subarea ___ ___ ___ House number ___ ___ ___ ___ Interviewer ID ___ ___

FORM 5: ASSESSMENT OF OVERALL, AGE-AND SEX-STRATIFIED ACTUAL HEALTHCARE UTILIZATION

HEALTHCARE SEEKING BEHAVIOR

1 Record below any occurrence of a sign(s)/ a symptom(s) (see List 1) and specify where (see List 2) and how often a member/members sought healthcare during the past 3 months?

Instructions: See "Form 3 - Part A - GENERAL HOUSEHOLD INFORMATION" to obtain the initials/identifier of each household member. Indicate one sign/symptom per line only. Start a new line if different healthcare options were selected for recurring signs/symptoms. Indicate the number of occurrences for each sign/symptom during the past 3 months. If a different healthcare option was taken for a recurring sign/symptom, indicate this on a new line. Please complete an additional form if >20 occurrences took place during the past 3 months.

**List 1: Sign/Symptom**		**List 2: Healthcare option**
[A] Fever, any<br>[B] Fever <3days (continuous)<br>[C] Fever ≥3days (continuous)<br>[D] Chills/ Shivering<br>[E] Convulsion<br>[F] Weight loss<br>[G] Dehydration<br>[H] Malaise/ Weakness/ Fatigue<br>[I] Aches/Pain (muscle/joint/bone)<br>[J] Headache<br>[K] Dizziness/Confusion/ Unconsciousness<br>[L] Blood pressure disorders<br>[M] Heart disorders	[N] Nausea/ Vomiting<br>[O] Diarrhea (watery/bloody/mucopurulent)<br>[P] Abdominal pain/ Intestinal pain<br>[Q] Blood loss/ Bleeding (internal)<br>[R] Blood loss/ Bleeding (external)<br>[S] Sneezing/ Runny nose<br>[T] Cough<br>[U] Rapid breathing/ Shortness of breath<br>[V] Severe breathing/ Difficulties in breathing<br>[W] Skin inflammation (rashes/redness/ pus/itching)<br>[X] Swelling/ Edema<br>[Y] Jaundice	[1] Study-HCF, Code ___________<br>[2] Other HCF, Code ___________<br>[3] Physician<br>[4] Pharmacy<br>[5] Traditional healer<br>[6] Nowhere/Self-treatment<br>[7] Nowhere/Nothing

No.	**Household member** Record the initials/identifier of a household member; see "Form 3 - Part A - GENERAL HOUSEHOLD INFORMATION"	**Sign/ Symptom** Use List 1 to select a code	**Healthcare option**		**Frequency** Indicate the number of incidences for the sign/symptom listed
			Use List 2 to select a code	HCF code	
1					
2					
3					
4					
5					
6					
7					
8					
9					

For peer review only - http://bmjopen.bmj.com/site/about/guidelines.xhtml

Site ___ ___ ___ Subarea ___ ___ ___ House number ___ ___ ___ ___ Interviewer ID ___ ___

10					
11					
12					
13					
14					
15					
16					
17					
18					
19					
20					

For peer review only

For peer review only - http://bmjopen.bmj.com/site/about/guidelines.xhtml

Appendix 3/Supplementary file 3: Protocol, Definitions/Terminology and Study Forms (French)

Protocol
Évaluation du comportement de santé et d'autres facteurs démographiques: une étude standardisée dans plusieurs cohortes en Afrique subsaharienne

Abstrait
Introduction L'objectif de l'étude HPAfrica est de déterminer les comportements sanitaires et autres facteurs démographiques, y compris les facteurs socio-économiques, ethnographiques aussi bien que d'hygiène et d'assainissement sur les sites d'étude du programme Fièvre Typhoïde Sévère en Afrique (SETA). L'objectif du programme SETA est d'enquêter sur la surveillance de la fièvre au niveau des établissements de soins de santé au Burkina Faso, en République Démocratique du Congo (RDC), en Éthiopie, au Ghana, à Madagascar et au Nigéria. Les estimations précises de la charge de morbidité nécessitent un ajustement des styles de comportements sanitaires, qui sont supposés varier au sein d'une population d'étude.

Methodes Pour la taille d'échantillon minimale requise, les hypothèses d'une population infinie, d'un effet de plan et d'une stratification par âge et sexe sont considérées. En l'absence d'un cadre d'échantillonnage ou d'une liste de ménages, une approche géospatiale sera utilisée pour générer des points aléatoires géographiques avec un outil ArcGIS (Aeronautical Reconnaissance Coverage Geographic Information System). Les impressions de l'imagerie satellite Google Earth Pro® visualisent ces points. Les données d'intérêt seront évaluées au niveau des ménages en différentes saisons en appliquant un échantillonnage simple, stratifié en fonction du poids. Une application basée sur Android et un service Web ont été mis au point pour la capture de données électroniques et la synchronisation avec le serveur de base de données sur Internet en temps réel. Cela permet la documentation de données simultanée.

Analyse Des analyses de données descriptives seront effectuées afin d'évaluer les informations de base sur / à propos de chaque population d'étude HPAfrica et le comportement santé stratifié par âge et par sexe. Ceci permettra d'ajuster les estimations de la charge de morbidité. En outre, des analyses multivariées seront appliquées pour examiner les associations entre le comportement santé, les facteurs demographiques et la charge de morbidité (fardeau de la maladie) trouvée dans l'étude SETA.

Défis attendus
- La normalisation dans la recherche multi-pays /-site à base communautaire nécessite de la prudence compte tenu des variations de cadre, de langue et de culture.
- Les données de population actuelles dans les sites d'étude et leurs sous-zones définies administrativement sont supposées être limitées.
- L'accessibilité aux ménages peut être compliqué par des facteurs environnementaux.
- La collecte de données électroniques peut entraîner problèmes techniques liés aux dommages, aux pannes fonctionnelles ou à la perte de dispositifs.
- Une connexion Internet sans fil instable peut limiter les équipes de données à mettre à jour l'application basée sur Android, communiquer en temps réel avec les responsables de l'étude et à synchroniser les données collectées avec le serveur.

Ethics References
- Comité d'Examen Institutionnel d'IVI, No. 2015-006
- Institute de Médecine Tropicale d'Antwerp, Comité d'Examen, Belgique
- Universiteit Antwerpen, Comite voor medische Ethiek, Belgique
- Ministère de la Santé du Burkina Faso – Comité d'Ethique pour la Recherche en Santé, Burkina Faso
- Comité d'Ethique de l'Ecole de Santé Publique de l'Université de Kinshasa, République Démocratique du Congo

For peer review only - http://bmjopen.bmj.com/site/about/guidelines.xhtml

- Comité National d'Examen de l'Éthique de la Recherche, Ministère des Sciences et de la Technologie, République Fédérale Démocratique d'Éthopie
- Comité d'Éthique de l'AHRI-ALERT, République Fédérale Démocratique d'Éthopie
- Kwame Nkrumah Université ded Sciences et de la Technologie, École des Sciences Médicales/Hôpital Universitaire Komfo Anokye, Comité de la Recherche humaines, de la Publication et de l'Éthique, Ghana
- Ministère de la Santé du Repoblikan Madagaskar – Comité d'Ethique
- Comité d'Éthique de l'Université d'Idadan/ Hôpital du Collège Universitaire/Comité de l'Éthique, Ibadan, Nigéria

Mots clés: comportement santé/hygiénique, assainissement, socio-économique, sélection aléatoire geospatiale, Afrique subsaharienne, HPAfrica étude

Contexte
L'évaluation du comportement de santé, y compris l'utilisation des soins de santé, est un déterminant important pour la production d'estimations précises du fardeau de la maladie parmi les populations cibles. Ceci est particulièrement important pour les études qui utilisent un modèle de surveillance observationnelle basé sur les établissements de santé, car une variation substantielle des comportements de santé influence fortement les estimations de la maladie (1, 2). L'ajustement des calculs de la maladie pour des variations du comportement en matière de santé augmentera l'exactitude de la prévalence des maladies causée par des pathogènes bactériens, fongiques, viraux ou parasitaires parmi la population étudiée. De plus, l'influence d'une grande variété de facteurs basés sur la population sur le comportement en matière de santé et le fardeau de la maladie a été reconnue. Cela inclut des facteurs socioéconomiques tels que l'éducation, la profession, le revenu, la disponibilité des biens des ménages (3, 4), les facteurs ethniques / religieux et culturels (5, 6), mais aussi l'hygiène et l'assainissement (7-11) - 14), et les contacts avec des animaux (10).

L'étude **H**ealth **P**opulation **Africa** (HPAfrica) sera menée au Burkina Faso, en République démocratique du Congo (RDC), en Éthopie, au Ghana, à Madagascar et au Nigéria (Tableau 1). Les expériences du programme Typhoid Fever Surveillance in Africa Program (TSAP) seront reportées sur le programme Severe Typhoid in Africa (SETA) (15, 16). Dans ce programme, une surveillance standardisée de la fièvre, basée sur les établissements de santé, est effectuée sur des sites d'étude sélectionnés dans les pays participants sur une période de deux ans (17). SETA vise principalement à collecter systématiquement des informations sur le fardeau des infections graves à *Salmonella*. Les sujets vivant dans la zone the chalandise définie, avec un épisode de fièvre aiguë, des antécédents de fièvre ≥ 3 jours consécutifs, une fièvre typhoïde cliniquement soupçonnée ou des perforations gastro-intestinales cliniquement diagnostiquées dues à la fièvre typhoïde seront éligibles. En outre, la sévérité des maladies provoquées par *Salmonella* et l'histoire naturelle de l'infection à *Salmonella* seront enregistrées avec des analyses supplémentaires sur les réponses immunitaires de l'hôte et le portage chronique.

Objectifs et Résultats
L'objectif de l'étude HPAfrica est d'évaluer le comportement de santé générique et réel stratifié par âge et par sexe. Le comportement générique se rapporte au comportement associé à la fièvre et à d'autres signes et symptômes sans l'apparition, alors que le comportement réel est lié à l'apparition (Formulaire 4 Partie A, Formulaire 5). Les données sont utilisées pour ajuster les incidences d'agents pathogènes infectieux stratifiés selon l'âge et le sexe pour la proportion d'une population étudiée non capturée par les activités de HPAfrica. Les données documentaires pour divers signes et symptômes ou une combinaison de ceux-ci sont utilisés pour une classification progressive de l'utilisation des soins de santé en fonction de la gravité de la maladie.

L'étude HPAfrica recueillera des données sur les facteurs associés aux soins de santé, tels que les modalités de voyage, la possession d'une assurance maladie, les facteurs culturels/ethnographiques, l'état vaccinal des enfants (18, 19) et la fréquence et la perception individuelle des maladies (Formulaire

For peer review only - http://bmjopen.bmj.com/site/about/guidelines.xhtml

4 Partie B). Les données démographiques (Formulaire 3 Partie A) seront utilisés pour la stratification par âge et sexe de la population étudiée qui est nécessaire pour calculer les facteurs d'ajustement pour les incidences de la maladie. Les données socio-économiques (Formulaire 3 Partie B) seront utilisées pour calculer les indices de richesse pour les approches descriptives et analytiques des populations étudiées. Les informations sur l'hygiène et l'assainissement (Formulaire 3 Partie C) peuvent permettre une meilleure compréhension de l'occurrence et de la fréquence des agents pathogènes identifiés parmi les populations étudiées.

Méthodes

Sites d'étude

Les limites de chaque site seront définies en utilisant des indications préexistantes qui avaient été déterminées par les autorités statistiques ou ministères de la santé (Tableau 1). L'examen rétrospectif des dossiers (registres/archives) de des deux dernières années aux établissements de soins de santé de recrutement permettra de redéfinir les limites du site. On s'attend à ce que cette revue révèle les résidences ou au moins les zones résidentielles administratives plus larges telles que des communautés, des districts ou des villages de patients qui ont cherché des soins de santé pour une raison quelconque. Des cartes et des images satellites peuvent être utilisées pour mieux visualiser les résidences des patients et les limites générales, y compris les sous-zones définies géographiquement ou administrativement de chaque site. Là où les limites ne peuvent être clairement définies, des facteurs supplémentaires, tels que la distance à un établissement de soins de santé de recrutement, peuvent être pris en compte.

Taille d'échantillons

Toutes les sources disponibles seront utilisées pour recueillir des données sur la population le plus à jour stratifiées selon l'âge et le sexe par site d'étude et par les sous-zones définies administrativement ou géographiquement. Cela peut inclure les dernières informations démographiques issues d'un recensement de la population ou d'un DSS (Demographic Surveillance System) / HDSS (Health and Demographic Surveillance System). Les chiffres de population et les facteurs de croissance de la population, s'ils sont disponibles, peuvent également être utilisés.

Le nombre minimum de ménages sélectionnés au hasard sera calculé en utilisant l'équation basée de précision (équation-I) (20-23) en supposant une population infinie et en considérant un effet de design (design effect, DEFF), y compris les hypothèses qui l'accompagnent:

Equation-I: Calcul de la taille d'échantillon basé sur la précision pour une population infinie (20-23):

$$n_0 = DEFF \cdot \left[z^2{}_{1-\alpha/2} \cdot \frac{4 \cdot p \cdot (1-p)}{d^2} \right]$$

Hypothèses d'Equation-I

n_0	Nombre minimum de ménages à interroger dans une zone d'étude en supposant une population infinie
DEFF	Effect de design; fixé à 1.5
$z_{1-\alpha/2}$	Déviation Standard correspondant à un intervalle de confiance de 95% (1.96 pour alpha de 0.05)
d	Précision (erreur acceptable); estimation de point; fixé à 0.2
p	Proportion de la population de l'étude devant se rendre dans un établissement de soins de santé de recrutement pour des conditions associées à de la fièvre et d'autres signes et symptômes (proportion capturée); fixé à 0.2
1-p	Proportion de la population de l'étude ne devant se rendre dans un établissement de soins de santé de recrutement pour des conditions associées à de la fièvre et d'autres signes et symptômes (proportion non capturée)

For peer review only - http://bmjopen.bmj.com/site/about/guidelines.xhtml

L'effet de design est un facteur d'ajustement pour le regroupement naturel des comportements de santé au sein des membres d'un ménage (21). Il est défini de façon arbitraire à 1,5 selon la taille du ménage puisqu'aucune estimation exacte n'est disponible (24, 25). Une proportion p de 0,2 pourra être considérée si aucune autre estimation n'est disponible ou p pourra être basé sur les expériences au cours de l'étude TSAP: Madagascar, Isotry : p=0,01, Burkina Faso, Polesgo: p=0,9 (26). Combiner tous les hypothèses dans l'équation-I va avoir pour résultat un nombre global minimum (n_0) de 92 ménages à interroger à chaque site.

En outre, n_0 pourra être pris en compte pour la répartition par âge et sexe de chaque population de l'étude représentée par $\hat{p}$, la proportion minimum supposée de stratification par groupe d'âge (<5 ans, ≥5 à <15 ans, ≥15 ans). Le facteur $\hat{p}$ pourra être défini de façon arbitraire à 0,2 ou être basé sur les expériences précédentes: Éthopie, Butajira : $\hat{p}$ =0,09, Burkina Faso, Polesgo : $\hat{p}$ =0,23 (26). Appliquer $\hat{p}$ à l'Equation-I pour une distribution binomiale, y compris ses postulats connexes, aura pour résultat le nombre minimum de ménages à interroger stratifié selon l'âge (Equation-II):

Equation-II: Calcul de la taille d'échantillon basé sur la précision pour une population infinie avec stratification selon l'âge:

$$\overline{n_0} = \left(DEFF \cdot \left[z^2{}_{1-\frac{\alpha}{2}} \cdot \frac{4 \cdot p \cdot (1-p)}{d^2} \right] \right) / \hat{p}$$

Hypothèses d'Equation-II

$\overline{n_0}$	Nombre minimum de ménages à interroger dans une zone d'étude en supposant une population infinie et une stratification selon l'âge
DEFF	Effect de design; fixé à 1.5
$z_{1-\alpha/2}$	Déviation Standard correspondant à un intervalle de confiance de 95% (1.96 pour alpha de 0.05)
d	Précision (erreur acceptable); estimation de point; fixé à 0.2
p	Proportion de la population de l'étude devant se rendre dans un établissement de soins de santé de recrutement pour des conditions associées à de la fièvre et d'autres signes et symptômes (proportion capturée); fixé à 0.2
1-p	Proportion de la population de l'étude ne devant se rendre dans un établissement de soins de santé de recrutement pour des conditions associées à de la fièvre et d'autres signes et symptômes (proportion non capturée)
$\hat{p}$	Proportion minimum de stratification par groupe d'âge, définie à 0,2

Les comportements de santé seront évalués deux fois dans les mêmes ménages si possible ou dans des ménages alternatifs, au cas où la population de l'étude serait instable en raison de la migration et / ou de taux de natalité / mortalité élevés. Des ménages supplémentaires, supposés être de 20% en moyenne (Tableau 2), peuvent être ajoutés pour tenir compte de la possible perte de suivi entre les deux évaluations. Eventuellement, $\overline{n_0}$ sera divisé par le nombre de sous-zones et pondéré proportionnellement à la taille de la population en appliquant un échantillonnage stratifié selon le poids (16).

Echantillonnage

Si une base d'échantillonnage ou une liste de ménages compréhensif et à jour existe (par DSS / HDSS ou recensement disponible), une sélection aléatoire informatisée sera effectuée en utilisant SAS (Système d'analyse statistique, version 9.4, SAS Institute, Cary NC) en appliquant une sélection aléatoire simple en série sans remplacement. S'il n'existe aucune base de sondage, celle-ci peut être construite en utilisant l'imagerie satellitaire ou les cartes aériennes pour dénombrer toutes les structures (26), raster (27) ou cellules de grille (28, 29).

Cependant, nous utilisons une technique géospatiale pour la création de points générés par l'outil de génération de points ArcGIS (Aeronautical Reconnaissance Coverage Geographic Information System, Redlands, CA, USA, version 10.2) avec l'application d'un échantillonnage simple stratifié selon le poids

For peer review only - http://bmjopen.bmj.com/site/about/guidelines.xhtml

(30, 31). L'outil place de façon aléatoire des nombres de points spécifiés dans les entités d'un polygone correspondant à une sous-zone administrative. Les coordonnées X et Y définissent un point jusqu'à ce que la taille d'échantillon soit atteinte. Les points aléatoires géographiques générés par ArcGIS sont convertis en un fichier image au format KML (Keyhole Markup Language) ou KMZ (Keyhole Markup landguage Zipped) et importés dans Google Earth Pro® (Google Inc., Mountain View, CA, États-Unis, version 6.2.2.6613). Un identifiant numérique unique et des coordonnées géographiques horizontales sont affectés à chaque point. L'emplacement de chaque point est vérifié sur une imagerie satellite Google Earth Pro® mise à jour. L'annexe 4 illustre notre approche en utilisant des exemples du Ghana et de Madagascar.

Les points géographiques étiquetés avec des identifiants sont représentés sur des impressions de taille d'affiche (60 x 60 à 60 x 90 cm) d'images satellite Google Earth Pro avec une haute résolution jusqu'au niveau de la structure. Ces cartes satellites aideront à localiser précisément les points sélectionnés sur le terrain. Des récepteurs GPS (Global Positioning System) portables (Garmin-eTrex, Garmin Ltd., Lenexa, KS, USA) aide à vérifier les emplacements. Les récepteurs seront positionnés près d'un point localisé, dans une position statique et en plein air pour assurer une lecture sans obstacle. En cas de refus, d'absence du répondant ou de localisation d'un point non résidentiel, un point résidentiel le plus proche de la droite ou de la gauche sera visité (32).

Fréquence de la collecte de données
Les points résidentiels seront visités deux fois pour des entrevues au cours du programme SETA pour prendre en compte un possible impact lié à la saison, sur le comportement sanitaire (33-37). Les enquêtes auront lieu à différentes saisons - une à la fin de la saison sèche ou au moins un mois après son début et une vers la fin de la saison des pluies ou au moins un mois après son début (Tableau 3). Actuellement, l'étude HPAfrica est en cours au Burkina Faso, au Ghana et à Madagascar et devrait se poursuivre dans les autres pays au cours des prochains mois.

Critères d'inclusion et d'exclusion
Les membres du ménage de tout âge et des deux sexes qui vivent dans la zone d'étude au jour de l'interview seront éligibles pour l'inclusion. Un ménage sera exclu si le répondant désigne refuse de participer ou n'est pas disponible après trois visites consécutives. Tous les visiteurs et individus dont le lieu de résidence n'est pas connu ou est en-dehors de la zone d'étude seront exclus.

Pour raison de cette enquête, un ménage est défini comme une personne ou un groupe de personnes apparentées ou non apparentées vivant dans la même unité d'habitation, reconnaissant un individu adulte comme chef du ménage, partageant les mêmes installations domestiques, et subvenant indépendamment à leurs besoins en termes de nourriture et des autres éléments essentiels pour vivre (26).

L'interview aura lieu exclusivement avec le répondant, qui est un membre adulte du ménage à l'âge de majorité légale spécifique au pays. Cette personne peut être identifiée comme décideur par les membres du même ménage et sert de substitut pour un ménage entier (15). D'autres définitions pertinentes sont expliquées subséquemment.

Collecte de données
Les intervieweurs de site recevront une formation sur la localisation des points géographiques, l'identification des répondants, les procédures de consentement éclairé en mettant l'accent sur la participation volontaire, et sur le déploiement de formulaires d'étude standardisés et pré-testés avant le lancement de HPAfrica. Les formulaires de consentement éclairé et d'étude seront traduits dans la langue officielle du pays et retraduits en anglais pour prendre en compte des erreurs de traduction et apporter des corrections, le cas échéant.

Gestion de données

For peer review only - http://bmjopen.bmj.com/site/about/guidelines.xhtml

La gestion des données dépendra du mode de collecte, qui sera principalement électronique plutôt que sur support papier. Les expériences passées ont montré les inconvénients suivants de la collecte de données sur papier: une forte probabilité d'erreurs lors du remplissage des formulaires d'étude, et le besoin de numérisation des données, qui est une étape supplémentaire, laborieuse, qui consomme du temps et sujette aux erreurs. La capture électronique de données à l'aide d'une application pour smartphone ou tablette réduit ces facteurs limitants à la qualité des données.

Il peut y avoir une période pilote au cours de laquelle des données sur support papier et sans papier seront collectées avant la mise en œuvre complète de la collecte de données électroniques. Ce dernier utilise l'application Android "HPA Collect" (Google Android 5.0.1 9API 23, disponible à l'adresse https://play.google.com/store/apps/details?id=anint.ivi.hpa) et la plate-forme "HPA Web" (CSS, JavaScript et JSP, disponible sur http://hpa.ivi.int/), qui sont tous deux accompagnés d'un dictionnaire de variables contenant les codes, la signification et les propriétés des variables au moins en anglais et en français. Une connectivité internet sans fil stable utilisant des navigateurs par défaut est nécessaire pour assurer une synchronisation simultanée, attribuable, originale et précise des données entre "HPA Collect" et "HPA Web". L'accès à "HPA Collect" et "HPA Web" est protégé par un mot de passe. L'annexe 5 affiche le diagramme du système, qui a les caractéristiques suivantes:

Structure ou fonctionnalités de "HPA Collect"

- **Gestion des utilisateurs:** Une interface utilisateur simple fournit différents privilèges aux utilisateurs pour entrer, modifier (sauf pour les étiquettes d'étude) et exporter ou transférer des données. Il établit une piste d'audit qui enregistre lorsque les utilisateurs entrent des données dans le serveur.
- **Saisie de données:** Les données sont entrées directement dans le smartphone ou la tablette. Les champs de saisie limitent les réponses possibles à des réponses plausibles uniquement en offrant différentes méthodes de saisie:
    - Modifier le texte: texte libre
    - Groupe radio / bouton Multi-clic: choix unique (radio group/Multi click button)
    - Case à cocher: choix multiple
    - Sélecteur de date et d'heure: date et heure d'entrée
    - Spinner: bouton déroulant

  Des fonctionnalités supplémentaires permettent à l'enquêteur de gagner du temps en transférant les données collectées plus tôt aux champs de données requis à un stade ultérieur de l'interview en effectuant des vérifications de vraisemblance pour divers champs de saisie de données et en reconnaissant facilement les modèles de saut. Les vérifications d'erreurs, les notifications de données manquantes, les erreurs de code et les erreurs logiques sont également incluses.
- **Vue des données:** Les données brutes entrées à l'origine peuvent être vues dans le même format lisible par l'homme et la machine, à savoir le format JSON, étant donné qu'elles seront transférées vers un serveur de base de données (Windows, MySQL). Des fonctions supplémentaires telles que «effacer» pour effacer tous les enregistrements, «réinitialiser» pour revenir à la page d'entrée de données principale, et «rechercher» pour rechercher une étiquette d'étude sont disponibles.
- **Paramètres:** la possibilité de changer la langue du système entre l'anglais et le français est fournie.
- **À propos de:** La version actuellement installée de «HPA Collect» est affichée.

Structure ou caractéristiques de "HPA Web"

- **Accueil:** une introduction à la plateforme est donnée et un tutoriel en français et en anglais sera accessible.
- **Aperçu du Formulaire:** Les formulaires d'étude sont affichés séparément montrant chaque question et les données d'entrée correspondantes en tant que variables définies.

For peer review only - http://bmjopen.bmj.com/site/about/guidelines.xhtml

- **Modifier le formulaire:** cette fonction permet la correction des données téléchargées. Il est uniquement accessible aux gestionnaires de données de chaque pays participant et à IVI.
- **Cherche:** Les étiquettes d'étude spécifiques données à chaque formulaire d'étude peuvent être filtrées.
- **Dictionnaire des variables:** Le dictionnaire variable peut être téléchargé.
- **Exporter:** Les données peuvent être converties à partir du serveur dans un fichier Excel transférable.
- **Contactez-nous:** Une correspondance rapide et facile entre les utilisateurs et le gestionnaire de données à IVI est donnée.

Les données seront examinées quotidiennement par le personnel clé de l'étude et vérifiées en termes de consistance et d'exactitude avant l'analyse des données. La qualité des données peut être vérifiée en sélectionnant un sous-ensemble de 5% pour la validation par rapport aux données originales sur support papier, si possible. Les noms des membres du ménage ne seront pas liés aux formulaires d'étude de HPAfrica et ne seront pas enregistrés dans la base de données afin d'assurer la confidentialité. L'accès à la base de données sera limité au personnel d'étude autorisé uniquement et les données seront conservées dans un endroit sécurisé et protégé. Des fichiers de sauvegarde provisoires périodiques et au moins trois fichiers de sauvegarde définitifs de la base de données stockés dans un endroit sécurisé et verrouillé éviteront la perte de données et garantiront la sécurité des données. Toutes les données seront conservées pendant au moins cinq ans.

L'analyse des données

Des analyses descriptives telles que les fréquences absolues et relatives, les médianes ou les moyennes arithmétiques seront effectuées pour construire une description contemporaine de la population de base.

Les analyses de base comprendront le calcul des proportions stratifiées selon l'âge et le sexe, des comportements sanitaires *génériques* et *réels*, y compris l'utilisation des soins de santé, pour divers signes et symptômes ou combinaisons de signes et de symptômes. Ces analyses permettront une classification graduelle basée sur la sévérité de la maladie, incluant un intervalle de confiance de 95%. Le comportement de santé sera ajusté pour un effet de regroupement naturel présumé parmi les membres du ménage. Des données stratifiées les plus à jour sur la population par site et les données démographiques dérivées de HPAfrica seront utilisées pour générer les proportions pour l'ajustement des calculs d'incidence.

Les informations socio-économiques collectées seront utilisées pour construire des indices de richesse des populations étudiées en appliquant des analyses factorielles basées sur la méthode des composantes principales (38). De plus, des analyses de régression bi et multivariée seront effectuées pour identifier les facteurs de confusion potentiels ou la modification des effets en utilisant les scores factoriels.

Les analyses de régression bi et multivariée seront effectuées en utilisant les scores factoriels pour l'évaluation des variables suivantes:

- Données socioéconomiques telles que l'éducation, le revenu, l'assurance maladie, le logement et les biens des ménages
- Hygiène et assainissement tels que l'accès aux toilettes, les sources d'eau, la manipulation des aliments, l'élimination des déchets et les contacts avec les animaux
- Comportement sanitaire: *réel* (stratifié selon l'âge et le sexe, gravité, fréquence), *générique* (stratifié selon l'âge et le sexe, gravité, classement par choix)
- Modalités de déplacement vers les établissements de santé, stratifiées selon l'âge
- Saisonnalité liée à l'hygiène et à l'assainissement, comportement sanitaire, modalités de déplacement
- Facteurs culturels / religieux / ethnographiques, stratifiés selon l'âge
- Statut de vaccination parmi les enfants ≤ 6 ans (18, 19)
- Connaissance et perception des maladies courantes

For peer review only - http://bmjopen.bmj.com/site/about/guidelines.xhtml

Considérations éthiques

Il n'y a pas d'avantages directs pour les ménages inscrits et leurs membres. Les avantages indirects pour la population générale sur les sites seront les informations mises à jour sur le fardeau des maladies transmissibles, y compris les facteurs influençant la transmission de la maladie et l'infection. L'étude HPAfrica génère des évidences pour aider les décideurs à introduire des mesures appropriées pour la prévention et le contrôle des maladies.

Ce protocole a fait l'objet d'examens par le Comité de Révision Institutionnelle d'IVI et des comités locaux spécifiques aux sites, comme indiqué dans la section «Références éthiques». Pendant et après l'étude HPAfrica, toutes les données des sujets inscrits seront gardés avec une stricte confidentialité et ne seront pas divulguées à une tierce partie par aucun membre de l'équipe de recherche. La protection par mot de passe des appareils et de la base de données est utilisée pour une stricte confidentialité. Toutes les données sur support papier (formulaires de consentement) seront conservées dans un endroit sécurisé et verrouillé. Les informations confidentielles stockées sur des ordinateurs et des sources de données sur support papier seront seulement mises à la disposition des co-chercheurs et du personnel d'IVI directement impliqués dans les activités d'étude de HPAfrica.

Abbréviations

JSON: JavaScript Objective Notation: CSS: Cascading Style Sheets: JSP: Java Server Page; SQL: Structured Query Language

For peer review only - http://bmjopen.bmj.com/site/about/guidelines.xhtml

Définitions/Terminologie

**A**	
Automédication	Fait de se médicamenter soi-même ou de traiter sa propre maladie ou son état sans supervision médicale ou ordonnance.
**B**	
**C**	
Choléra	Maladie infectieuse de l'intestin grêle qui peut être mortelle causée par la bactérie *Vibrio cholerae* ; elle se propage par voie fécale-orale par l'ingestion d'eau et d'aliments contaminés et provoque fièvre, diarrhée aqueuse sévère, vomissements et déshydratation.
Confusion/Vertiges/Perte de connaissance	L'état de désorientation ou d'absence de clarté d'esprit ; l'état anormal de réponse aux stimuli dans l'environnement; faible, wooziness, faiblesse, instabilité ou tendance à tomber; l'interruption de la conscience de soi et de son environnement.
Convulsion	Mouvement soudain, violent et irrégulier d'un membre/d'une partie du corps ou de tout le corps causé par une contraction involontaire des muscles.
Coqueluche (Pertussis)	La coqueluche est une maladie bactérienne très contagieuse. Les symptômes d'un froid commun comme nez qui coule, fièvre et toux sont suivis par des semaines de toux sévère et un son aigu de la coqueluche ou haletant quand une personne malade inspire; toux sévère peut être suivie de vomissements, rupture des côtes ou fatigue extrême. Les très jeunes et les personnes âgées infectées peuvent avoir peu ou pas de toux, ou peuvent avoir des périodes de respiration interrompue pendant lesquelles elles ne respirent pas. La coqueluche est transmise par la bactérie *Bordetella pertussis* par toux et éternuements d'une personne infectée.
**D**	
Déshydration	Une condition de perte excessive d'eau/d'hydratation par le corps.
Diarrhée	Une condition d'évacuer les matières fécales du corps très fréquemment et sous forme plutôt liquide que solide.
Difficultés respiratoires/ Respiration sévère	Une condition de la respiration anormale accompagnée d'une respiration sifflante, d'une douleur thoracique, d'un tirage thoracique, d'un stridor, d'un gonflement de la poitrine ou d'une toux par exemple.
Diphthérie	Maladie hautement contagieuse, parfois mortelle, causée par la bactérie *Corynebacterium diphtheriae*. Elle se transmet de personne à personne, par l'air ou les objets contaminés et affecte les voies respiratoires supérieures. Les symptômes courants sont maux de gorge, gonflement des glandes/ ganglions lymphatiques, malaise, fièvre/frissons, substance membraneuse/matière grise et épaisse qui se forme dans la gorge/sur les amygdales et gêne/accélère la respiration (toux rauque) et gêne pour avaler. Une forme de diphtérie qui implique la peau, les yeux et les organes génitaux est également signalée et se caractérise par une peau douloureuse, rouge et enflée.
Douleur abdominale/ Douleur intestinale	Douleur (aiguë/courte durée ou chronique/longue durée) qui survient entre la poitrine et l'aine (souvent désignée comme la région de l'estomac/du ventre)
Douleur intestinale/Douleur abdominale	Douleur (aiguë/courte durée ou chronique/longue durée) qui survient entre la poitrine et l'aine (souvent désignée comme la région de l'estomac/du ventre)
Douleur/Maux	État caractérisé par une douleur sourde continue/prolongée dans une partie du corps ; la sensation/souffrance/inconfort physique causée par

For peer review only

For peer review only - http://bmjopen.bmj.com/site/about/guidelines.xhtml

	une maladie/une blessure/quelque chose qui fait mal au corps.
**E**	
Éducation formelle	Modèle d'éducation systématique et organisée structuré et administré selon un ensemble de lois et de normes ; les institutions d'éducation formelle sont organisées de façon administrative, physique et pédagogique et exigent des étudiants une assiduité minimum ; il implique des évaluations intermédiaires et finales afin d'accéder au niveau d'apprentissage suivant ; il délivre des certificats et des diplômes conformément à un ensemble de règles strictes.
Essoufflement/ Respiration rapide	Fait de respirer anormalement rapidement et profondément ; à une fréquence de >20 respirations par minute pour les adultes, >30 respirations par minute pour les enfants et >45 respirations par minute par les bébés.
Établissement de santé	Lieux ou institutions où des soins de santé sont prodigués ; dont les hôpitaux, les cliniques, les centres de santé, les postes de santé et les centres de soins spécialisé.
Éternuement	Fait d'expulser soudainement l'air par le nez et la bouche avec un bruit important caractéristique.
**F**	
Faiblesse/Malaise/Fatigue	État de se sentir fatigué/épuisé/à bout de forces ; sentiment général d'inconfort/malaise/de ne pas être en bonne santé ou heureux.
Fatigue/Malaise/ Faiblesse	État de se sentir fatigué/épuisé/à bout de forces ; sentiment général d'inconfort/malaise/de ne pas être en bonne santé ou heureux.
Fièvre	Température corporelle anormalement élevée (habituellement au-dessus de 38°C).
Fièvre <3 jours	Température corporelle anormalement élevée (habituellement au-dessus de 38°C) qui dure moins de 3 jours et apparaît en continu.
Fièvre >3 jours	Température corporelle anormalement élevée (habituellement au-dessus de 38°C) qui dure plus de 3 jours et apparaît en continu.
Fièvre jaune	Maladie infectieuse aiguë, pathogène pour l'homme et virale caractérisée par l'apparition soudaine de symptômes comme fièvre, frissons, perte d'appétit, nausée, douleurs musculaires et maux de tête qui disparaissent généralement en quelques jours ou sont suivis de symptômes plus graves comme jaunisse, fièvre forte et hémorragie. La maladie est causée par un virus du genre Flavivirus et est transmise par la piqûre d'un moustique infecté.
Fièvre typhoïde	Maladie infectieuse bactérienne potentiellement mortelle causée par *Salmonella typhi* ; elle se transmet par la voie fécale-orale par les aliments et l'eau contaminés ou de personne à personne. Les symptômes courants sont forte fièvre, maux de tête, douleur abdominale, constipation/diarrhée et éruption cutanée. Des complications graves (perforation de l'intestin) sont rapportées.
Frissons	Sensation de froid qui peut être accompagnée de frissons/tremblements du corps et de pâleur de la peau
**G**	
Gonflement/Œdème	État d'accumulation excessive de liquide aqueux/gonflement des cavités ou des tissus du corps ; couramment constaté dans les mains/bras/pieds/jambes ; augmentation du volume de tissu.
Grippe	Maladie infectieuse hautement contagieuse des voies respiratoires causée par le virus de la grippe ; elle se propage de personne à personne par les particules expulsées par la toux/les éternuements/la parole et les objets contaminés. Les symptômes courants sont fièvre,

For peer review only - http://bmjopen.bmj.com/site/about/guidelines.xhtml

	douleurs dans les muscles/bras/jambes, frissons/sueur, maux de tête, fatigue/faiblesse, nez qui coule/congestion nasale, éternuements, maux de gorge et toux sèche et persistante.
Guérisseur traditionnel	Personne qui cherche à maintenir la santé aussi bien qu'à prévenir, diagnostiquer et améliorer ou traiter les maladies en utilisant un ensemble de connaissances, compétences et pratiques basées sur des théories, des croyances et des expériences.
**H**	
Haemophilus influenzae de type B	Bactérie qui provoque une série de maladies et se propage par les particules expulsées par la toux ou les éternuements, et surviennent surtout chez l'enfant de moins de 5 ans ; les symptômes courants sont fièvre, maux de tête et raideur dans le cou jusqu'à l'état septique. Elle peut causer otites, cellulites (infection des tissus mous), arthrite, infections des voies respiratoires supérieures, pneumonies, méningites (avec dommages potentiels au cerveau) et épiglottites (avec obstruction des voies aériennes).
Hémorragie (interne/externe)/ Perte de sang	État caractérisé par une perte de sang du système vasculaire, soit de façon interne à l'intérieur du corps (hémorragie interne) soit de façon externe par un orifice naturel ou une lésion de la peau (hémorragie externe) ; sang qui s'échappe d'un vaisseau sanguin endommagé.
Hépatite B	Maladie infectieuse causée par le virus de l'hépatite B (VHB) qui affecte le foie (infection hépatique aiguë/chronique ; peut entraîner une insuffisance hépatique/un cancer/une cirrhose). Le virus se transmet de personne à personne par le sang/le sperme/les autres fluides corporels. Les symptômes courants sont fièvre, malaise/faiblesse/fatigue, perte d'appétit, nausée/vomissements, gêne/douleur abdominale, urines foncées, douleurs articulaires et jaunisse.
Hépatite E	Maladie infectieuse causée par le virus de l'hépatite E (VHE) qui affecte le foie (infection hépatique aiguë uniquement ; peut entraîner une insuffisance hépatique aiguë). Le virus se propage par les voies fécales-orales par l'ingestion d'eau ou d'aliments contaminés, les animaux contaminés (de façon zoonotique) et par le sang. Les symptômes courants sont fièvre, malaise, perte d'appétit, diarrhée, nausée, gêne abdominale, urines foncées, douleurs articulaires et jaunisse.
**I**	
**J**	
Jaunisse	Maladie qui fait jaunir la peau/le blanc des yeux.
**K/L**	
**M**	
Malaise/Fatigue/ Faiblesse	État de se sentir fatigué/épuisé/à bout de forces ; sentiment général d'inconfort/malaise/de ne pas être en bonne santé ou heureux.
Maux de tête	Douleur continue dans une région de la tête.
Maux/Douleur	État caractérisé par une douleur sourde continue/prolongée dans une partie du corps ; la sensation/souffrance/inconfort physique causée par une maladie/une blessure/quelque chose qui fait mal au corps.
Médecin	Une personne compétente/formée aux soins ; une personne éduquée, cliniquement expérimentée et autorisée à pratiquer la médecine.
Ménage	Personne ou groupe de personnes apparentées ou non qui vivent ensemble dans la même unité d'habitation, qui reconnaissent un adulte de sexe masculin ou féminin comme le chef du ménage, qui partagent

For peer review only

For peer review only - http://bmjopen.bmj.com/site/about/guidelines.xhtml

	les mêmes installations domestiques, qu'on considère comme formant une unité, et qui subviennent à leurs besoins en termes de nourriture et des autres éléments essentiels pour vivre. Un ménage peut être situé dans une unité d'habitation unique (bâtiment à un seul étage) ou dans un ensemble de logements collectifs (bâtiment à plusieurs étages).
Méningite	Inflammation virale/bactérienne/fongique infectieuse parfois mortelle des membranes (méninges) qui entourent le cerveau et la moelle épinière ; elle se propage de personne à personne par les particules expulsées par la toux/les éternuements/la parole et les objets contaminés. Les symptômes courants sont forte fièvre, maux de tête sévères, nausée/vomissements, confusion, convulsions ; perte d'appétit et de la soif, fatigue, sensibilité à la lumière, éruption cutanée et raideur au cou.
**N**	
Nausée/Vomissements	Sensation de malaise dans l'estomac avec tendance à vomir ; fait d'éjecter une partie/tout le contenu de l'estomac principalement par la bouche.
Nez qui coule	État résultant de la production par le nez (tissus nasaux et adjacents et vaisseaux sanguins) de mucus/drainage excessif, qui coule du nez pour retirer quelque chose.
**O**	
Œdème/Gonflement	État d'accumulation excessive de liquide aqueux/gonflement des cavités ou des tissus du corps ; couramment constaté dans les mains/bras/pieds/jambes ; augmentation du volume de tissu.
Oreillons	Maladie infectieuse virale causée par le virus des oreillons qui affecte principalement les glandes parotides qui sont situées sous et devant les oreilles ; elle se propage de personne à personne par la salive infectée/les particules de salive infectées expulsées par la toux/les éternuements/la parole et les objets contaminés. Les symptômes courants sont fièvre, gonflement d'une/des deux glandes parotides, maux de tête ; douleurs musculaires, faiblesse/fatigue, perte d'appétit et douleurs en mâchant/avalant ; une complication connue est une possible perte d'audition.
**P**	
Paludisme	Maladie infectieuse parfois mortelle causée par un parasite (*Plasmodium* spp.) transmis principalement par la piqûre de moustiques infectés, en dehors de la transmission mère-enfant et par le sang. Les symptômes courants sont attaques récurrentes de frissons, sueurs, forte fièvre, maux de tête, vomissements et diarrhée.
Personne interrogée	Membre adulte (à l'âge légal de majorité du pays concerné) du ménage qui est un décideur en ce qui concerne les soins de santé/l'utilisation des soins de santé pour le ménage entier à l'intérieur du site d'étude ; cette personne adulte parle ainsi au nom de tous les membres du ménage. Cette personne peut être identifiée par les autres membres du même ménage comme la personne qui est principalement impliquée dans les soins quotidiens des membres du ménage.
Perte de connaissance/ Vertiges/Confusion	L'état de désorientation ou d'absence de clarté d'esprit ; l'état anormal de réponse aux stimuli dans l'environnement; faible, wooziness, faiblesse, instabilité ou tendance à tomber; l'interruption de la conscience de soi et de son environnement.
Perte de poids	Baisse (volontaire/involontaire) du poids du corps.
Perte de sang/	État caractérisé par une perte de sang du système vasculaire, soit de

For peer review only - http://bmjopen.bmj.com/site/about/guidelines.xhtml

Hémorragie	façon interne à l'intérieur du corps (hémorrage interne) soit de façon externe par un orifice naturel ou une lésion de la peau (hémorragie externe) ; sang qui s'échappe d'un vaisseau sanguin endommagé.
Pharmacie	Endroit où seuls des médicaments (ordonnance requise/non requise) sont préparés, conservés, composés et dispensés.
Pneumocoque	Une inflammation infectieuse bactérienne des poumons parfois mortelle qui affecte souvent les personnes immunodéprimées et est causée par la bactérie *Streptococcus pneumoniae* ; elle se propage de personne à personne par les particules expulsées par la toux/les éternuements/la parole et les objets contaminés. Les symptômes courants sont fièvre, frissons, toux (avec mucosités/pus), douleurs thoraciques, difficulté à respirer/essoufflement, fatigue, nausée/vomissements et diarrhée ; des symptômes rares sont septicémie, épanchement pleural et empyème.
Polio	Maladie virale infectieuse, très contagieuse, parfois mortelle, causée par le poliovirus qui cause dans sa forme la plus sévère paralysie et difficultés à respirer ; il se transmet par la voie fécale-orale par ingestion d'eau et d'aliments contaminés et de personne à personne. Les symptômes courants sont fièvre, maux de gorge, maux de tête, vomissements, fatigue, maux de dos, de cou/raideur au cou, faiblesse des muscles/muscles douloureux, méningite, perte de réflexes, membres lourds/pendants.
Occupation	A job, or means of earning a wage/living; often requires the mastery of a complex set of knowledge, tasks, duties and skills acquired through formal education and/or practical experience.
**Q**	
**R**	
Respiration rapide/ Essoufflement	Fait de respirer anormalement rapidement et profondément ; à une fréquence de >20 respirations par minute pour les adultes, >30 respirations par minute pour les enfants et >45 respirations par minute par les bébés.
Respiration sévère/ Difficultés respiratoires	Une condition de la respiration anormale accompagnée d'une respiration sifflante, d'une douleur thoracique, d'un tirage thoracique, d'un stridor, d'un gonflement de la poitrine ou d'une toux par exemple.
Revenus/Salaire	Argent ou autre forme de paiement que quelqu'un reçoit périodiquement/régulièrement en échange de biens ou de services. Provient généralement d'un emploi/d'une profession.
Rotavirus	Infection virale causée par le rotavirus qui cause principalement des diarrhées pour les enfants de moins de 5 ans ; elle se propage par la voie fécale-orale, de personne à personne et par les objets contaminés. Les symptômes courants sont diarrhée aqueuse, fièvre, vomissements, douleurs abdominales et déshydratation.
Rougeole	Maladie infectieuse parfois mortelle des voies respiratoire (nez/gorge) causée par un virus qui affecte principalement les enfants de moins de 5 ans ; elle se propage de personne à personne par les particules expulsées par la toux/les éternuements/la parole et les objets contaminés. Les symptômes courants sont fièvre, toux sèche, nez qui coule, inflammation des yeux, maux de gorge, larges plaques sur la peau et points blancs dans la bouche et sur les joues.
Rubéole	Maladie infectieuse virale contagieuse connue pour son éruption cutanée rouge distinctive qui est causée par le virus de la rubéole ; elle se transmet de personne à personne (toux/éternuements/parole), de la mère à l'enfant par le sang et les objets contaminés. Les symptômes courants sont fièvre modérée, maux de tête, nez bouché/qui coule,

For peer review only

	yeux rouges/inflammation des yeux, ganglions lymphatiques gonflés et sensibles à la base du crâne/à l'arrière du cou/derrière les oreilles, douleurs articulaires et éruption cutanée rose qui se déplace du visage au torse, aux bras et aux jambes.
**S**	
Soins de santé - généraux	Soins de santé pour les maladies qui n'ont aucun lien avec l'apparition de maladies associées aux signes et symptômes ou les dates de début de maladies.
Soins de santé - réels	Soins de santé liés l'apparition de maladies associées aux signes et symptômes ou les dates de début de maladies.
**T**	
Témoin	Personne adulte (à l'âge légal de majorité du pays concerné), qui est indépendante de l'étude, qui ne peut pas être indûment influencée par le personnel de l'étude, qui participe au processus de consentement éclairé si le sujet/le représentant légal du sujet est analphabète, et qui lit (lettré) le formulaire de consentement éclairé et toute autre information écrite présentée au sujet.
Tétanos	Une maladie infectieuse caractérisée par des spasmes musculaires qui commencent généralement dans la mâchoire et progresse vers le reste du corps. Les spasmes durent généralement quelques minutes et se produisent fréquemment pendant les 3-4 semaines au début de la maladie, ils peuvent être si graves qu'ils peuvent causer des fractures osseuses ou la mort lorsqu'ils affectent les muscles respiratoires. Elle peut être caractérisée par d'autres symptômes comme fièvre, sueurs, maux de tête, difficultés à avaler, pression artérielle élevée et augmentation du rythme cardiaque. La maladie est causée par une infection par la bactérie *Clostridium tetani* qui se trouve dans sol, saliva, poussière et fumier. La bactérie entre par une lésion cutanée (par exemple, coupure, blessure par ponction) par un objet contaminé. Il produit des toxines qui interfèrent avec les contractions musculaires qui résultent des spasmes typiques.
Toux	Fait d'expulser de façon soudaine/rapide et bruyamment l'air des poumons ; souvent de façon involontaire afin de dégager les voies respiratoires des poumons de fluides/mucus/autres matières
Troubles cardiaques	Tout trouble que affecte le cœur ; maladies qui impliquent des vaisseaux sanguins rétrécis/bloqués qui peuvent entraîner une attaque cardiaque/douleur thoracique/accident vasculaire cérébral ; maladie qui affecte le muscle/les valves/le rythme du cœur.
Troubles de la pression artérielle	Pression artérielle élevée/faible ou pression artérielle qui présente des fluctuations excessives.
Tuberculose	Maladie infectieuse bactérienne (*Mycobacterium tuberculosum*), contagieuse, potentiellement grave qui affecte principalement les poumons en plus des reins et de la colonne vertébrale/du cerveau ; elle se transmet de personne à personne par les particules expulsées par la toux/les éternuements/la parole. Les symptômes courants d'une tuberculose active sont toux (avec/sans sang), douleur thoracique, perte de poids, fatigue, fièvre, sueurs nocturnes, frissons et perte d'appétit.
**U**	
**V**	
Vertiges/Confusion/Perte de connaissance	L'état de désorientation ou d'absence de clarté d'esprit ; l'état anormal de réponse aux stimuli dans l'environnement; faible, wooziness, faiblesse, instabilité ou tendance à tomber; l'interruption de la

For peer review only - http://bmjopen.bmj.com/site/about/guidelines.xhtml

	conscience de soi et de son environnement.
VIH/SIDA	Maladie infectieuse causée par le virus de l'immunodéficience humaine (VIH) qui affecte/endommage le système immunitaire et entraîne le syndrome immunodéficitaire acquis (SIDA), une maladie chronique et potentiellement mortelle ; il se transmet par les liquides corporels infectieux comme le sang/le sperme/les autres fluides corporels. Les symptômes courants d'une infection aiguë/précoce sont fièvre, fatigue, maux de tête, douleurs articulaires, éruption cutanée, maux de gorge, ganglions lymphatiques enflés, diarrhée, perte de poids, candidose orale et zona. La maladie non traitée évolue en SIDA ; les symptômes causés par un système immunitaire gravement endommagé sont sueurs, fièvre récurrente, diarrhée chronique, lésions sur la langue et dans la bouche, fatigue, perte de poids et éruptions cutanées.
Visiteur	Personne (apparentée ou non) qui rend visite à un ménage pour passer du temps avec les membres du ménage ; un visiteur ne vit pas avec les membres du ménage dans la même unité d'habitation, ne partage pas les mêmes installations domestiques, la même nourriture et autres éléments essentiels à la vie que les membres du ménage, et ne constitue pas une unité avec les membres du ménage.
Voisin	Personne (apparentée ou non) vivant dans l'habitation à côté de celle d'un ménage cible ; un voisin ne vit pas avec les membres d'un ménage (à côté) dans la même unité d'habitation, ne partage pas les mêmes installations domestiques, aliments ou autres éléments essentiels à la vie que les membres du ménage (à côté), et ne constitue pas une unité avec les membres du ménage (à côté).
Vomissements/Nausée	Sensation de malaise dans l'estomac avec tendance à vomir ; fait d'éjecter une partie/tout le contenu de l'estomac principalement par la bouche.
**W/X/Y/Z**	

For peer review only

For peer review only - http://bmjopen.bmj.com/site/about/guidelines.xhtml

Formulaires de l'étude

Site ___ ___ ___ Sous-zone ___ ___ ___ Numéro d'habitation ___ ___ ___ ___ Identifiant de l'enquêteur ___ __

FORMULAIRE 1 – COMPTE RENDU DES VISITES AUX MÉNAGES

Instructions pour l'enquêteur

- Compléter « Formulaire 1 – COMPTE RENDU DES VISITES AUX MÉNAGES » pour chaque ménage visité.
- Compléter « Foyer/Personne interrogée/Complété » une fois que toutes les parties de l'enquête ont été complétées ; si toutes les données n'ont pas été recueillies lors de la visite initiale et/ou d'une visite ultérieure (max. 3 visites), programmer une nouvelle visite.
- Tentative 1: Compléter le statut de la visite au ménage et utilisant les choix proposés ; pour « pas à la maison », « à la maison/ne répond pas » et « à la maison/répond/indisponible », programmer une nouvelle visite ; pour « à la maison/répond/refus » indiquez la raison de refus et visitez un ménage de remplacement au plus proche à droite ou à gauche de ce ménage; pour « inexistant/pas d' habitation », passer ce ménage et visiter un ménage de remplacement au plus proche à droite ou à gauche à la place de ce ménage.
- Tentative 2: Compléter le statut de la visite du ménage et utilisant les choix proposés ; pour « pas à la maison », « à la maison/ne répond pas » et « à la maison/répond/indisponible », programmer une nouvelle visite.
- Tentative 3: Compléter le statut de la visite au ménage et utilisant les choix proposés ; pour « pas à la maison », « à la maison/ne répond pas » et « à la maison/répond/indisponible », passer ce ménage et visiter un ménage de remplacement au plus proche à droite ou à gauche à la place de ce ménage.
- « Site » et « sous-zone » doivent être un numéro à trois chiffres, « numéro d'habitation » un numéro à quatre chiffres, et « identifiant de l'enquêteur » un numéro à deux chiffres (utiliser les listes préparées individuellement pour chaque site pour obtenir les codes) ; saisir « date » au format JJ/MM/AAAA et « heure » au format HH:MM et entourer MATIN ou APRÈS-MIDI. «Étiquette de l'étude» doit être une combinaison d'un code «site» à trois chiffres, d'un code «sous-zone» à trois chiffres et d'un code «numéro d'habitation» à quatre chiffres; le « identifiant de l'enquêteur » doit être un numéro à trois chiffres.

For peer review only

**Tentative 1**	Date ____/____/____ (JJ/MM/AA)	Heure _____/_____ MATIN/APRÈS-MIDI	Identifiant de l'enquêteur ___ ___
STATUT		Cocher un	
1=Pas à la maison		○	➡ Planifier nouvelle visite (date/heure)
2=À la maison/ Non-répondant		○	➡ Planifier nouvelle visite (date/heure)
3=À la maison/ répondant /Indisponible		○	➡ Planifier nouvelle visite (date/heure)
4= À la maison/ Répondant /Complété		○	
5= À la maison/ répondant /Refus		○	Notez la/les raison(s) du refus; cochez tout ce que s'applique:<br>○ 1=Pas de temps pour répondre aux questions<br>○ 2=Ne veulent pas participer/répondre aux questions<br>○ 3=Autre ______________________<br>○ 99=Pas de réponse ○ 98=Ne sait pas<br>➡ Visitez un ménage de remplacement le plus proche de ce ménage du côté droit ou gauche
6=Inexistant/Pas une habitation		○	➡ Visitez un ménage de remplacement le plus proche de ce ménage du côté droit ou gauche

For peer review only - http://bmjopen.bmj.com/site/about/guidelines.xhtml

**Tentative 2** Date ____/____/____ (JJ/MM/AA)	Heure _____/_____ MATIN/APRÈS-MIDI	Identifiant de l'enquêteur ___ ___
STATUT	Cocher un	
1=Pas à la maison	O	➡ Planifier nouvelle visite (date/heure)
2=À la maison/Ne répond pas	O	➡ Planifier nouvelle visite (date/heure)
3=À la maison/Répond/Indisponible	O	➡ Planifier nouvelle visite (date/heure)
4= À la maison/Répond/Complété	O	
5= À la maison/ répondant /Refus	O	Notez la/les raison(s) du refus; cochez tout ce que s'applique:<br>O 1=Pas de temps pour répondre aux questions<br>O 2=Ne veulent pas participer/répondre aux questions<br><br>O 3=Autre ______________________<br>O 99=Pas de réponse O 98=Ne sait pas<br><br>➡ Visitez un ménage de remplacement le plus proche de ce ménage du côté droit ou gauche
6=Inexistant/Pas une habitation	O	➡ Visitez un ménage de remplacement le plus proche de ce ménage du côté droit ou gauche
**Tentative 3** Date ____/____/____ (JJ/MM/AA)	Heure _____/_____ MATIN/APRÈS-MIDI	Identifiant de l'enquêteur ___ ___
STATUT	Cocher un	
1=Pas à la maison	O	➡ Visitez un ménage de remplacement le plus proche de ce ménage du côté droit ou gauche
2=À la maison/Ne répond pas	O	➡ Visitez un ménage de remplacement le plus proche de ce ménage du côté droit ou gauche
3=À la maison/Répond/Indisponible	O	➡ Visitez un ménage de remplacement le plus proche de ce ménage du côté droit ou gauche
4= À la maison/Répond/Complété	O	
5= À la maison/ répondant /Refus	O	Notez la/les raison(s) du refus; cochez tout ce que s'applique:<br>O 1=Pas de temps pour répondre aux questions<br>O 2=Ne veulent pas participer/répondre aux questions<br><br>O 3=Autre ______________________<br>O 99=Pas de réponse O 98=Ne sait pas<br><br>➡ Visitez un ménage de remplacement le plus proche de ce ménage du côté droit ou gauche
6=Inexistant/Pas une habitation	O	➡ Visitez un ménage de remplacement le plus proche de ce ménage du côté droit ou gauche

For peer review only

Site ___ ___ ___ Sous-zone ___ ___ ___ Numéro d'habitation ___ ___ ___ ___ Identifiant de l'enquêteur ___ __

FORMULAIRE 2 – FORMULAIRE DE CONSENTEMENT

Evaluation des soins de santé à [site SETA and –pays]

Instructions à l'enquêteur

- Lire le "Formulaire 2 – FORMULAIRE DE CONSENTEMENT" pour le répondant/demander au répondant de lire attentivement le "Formulaire 2 – FORMULAIRE DE CONSENTEMENT" s'il préfère.
- Le répondant tel que défini dans cette enquête est un adulte (ayant l'âge de la majorité spécifique au pays) membre du ménage et responsable des décisions journalières concernant la santé des membres du ménage; il ne doit pas par exemple être un voisin ou un visiteur.
- S'assurer que le répondant a pleinement compris l'objectif, les procédures, les risques et les bénéfices de cette étude avant d'obtenir son consentement à participer.
- Le répondant doit signer le "Formulaire 2 – FORMULAIRE DE CONSENTEMENT"; si le répondant est incapable de signer/analphabète, une empreinte digitale servira en lieu et place de la signature, et un adulte lettré indépendant de l'étude qui servira de témoin (un membre du même ménage ou un voisin par exemple) signera le "Formulaire 2 – FORMULAIRE DE CONSENTEMENT".
- Collecter les informations sur tous les membres du ménage indépendamment du fait qu'ils soient présents ou non au moment de l'entretien; Recueillir l'information sur seulement les individus présents au moment de l'entretien résultera en un biais dans les données.
- Remplir le "Formulaire 2 – FORMULAIRE DE CONSENTEMENT" pour chaque ménage consentant enrôlé dans l'étude.
- Expliquer au répondant qu'il est prévu de revisiter chaque ménage durant une période de 2 ans; a chaque visite, l'équipe de recherche obtiendra un nouveau consentement.
- «Site» et «sous-zone» doivent être un numéro à trois chiffres, «numéro d'habitation» un numéro à quatre chiffres, et «identifiant de l'enquêteur» un numéro à deux chiffres (utiliser les listes préparées individuellement pour chaque site pour obtenir les codes); saisir «date» au format JJ/MM/AAAA et «heure» au format HH:MM et entourer MATIN ou APRÈS-MIDI. «Étiquette de l'étude» doit être une combinaison d'un code «site» à trois chiffres, d'un code «sous-zone» à trois chiffres et d'un code «numéro d'habitation» à quatre chiffres; le «identifiant de l'enquêteur» doit être un numéro à trois chiffres.

Investigateur principal et co-investigateur de l'étude

[Ajouter les noms de l'investigateur principal et des co-investigateurs de l'étude ainsi que leurs affiliations respectives]

Introduction

L'Institut International des Vaccins (IVI) et [ajouter l'institution d'affiliation des investigateurs locaux] travaillent ensemble pour examiner les problèmes de santé et le comportement de santé dans votre communauté [ajouter le site SETA-pays], y compris le statu socio-économique, le comportement d'hygiène et d'assainissement et le statut vaccinal des enfants. Nous aimerions mieux comprendre où vous, votre famille et vos voisins cherchez des soins de santé en cas de maladies causant la fièvre/corps chaud et autres chante & symptômes. Cette information nous aidera à savoir à quelle fréquence vous souffrez de maladies causant de la fièvre et les autres liées à la fièvre, fournir une meilleure image des facteurs influençant la transmission de la maladie et l'infection, et à trouver des moyens pour les prévenir et traiter dans votre communauté. Vous, en tant que responsable principal pour le comportement de santé/l'utilisation des soins de santé quotidienne pour les membres de ce ménage, êtes invités à participer parce que vous vivez dans la localité où nous effectuons cette étude. Ce formulaire de consentement sera lu par vous ou pour vous et vous aurez le temps pour prendre votre décision d'accepter ou de refuser de participer à l'étude. S'il vous plaît, demandez aux membres de l'étude d'expliquer tout mot ou toute information que vous ne comprenez pas. On vous donnera en conséquence plus d'informations sur cette étude, y compris ses risques potentiels, les avantages et les inconvénients. Après vous avoir entièrement expliqué l'étude et si vous acceptez de participer, on vous demandera de signer ce formulaire de consentement au nom de tous les membres du ménage. Si vous ne pouvez pas signer le formulaire, nous vous demandons de fournir votre empreinte digitale, et une personne adulte indépendante de l'étude (membre de votre ménage/voisin) signera le formulaire en tant que témoin. Nous allons vous remettre une copie signée et datée du formulaire de consentement.

Objectif

L'objectif de cette étude est d'examiner les problèmes de santé et l'utilisation des soins de santé dans votre communauté [ajouter le site SETA-pays], y compris le statu socio-économique et le comportement d'hygiène et d'assainissement. Notre objectif est de mieux comprendre où vous, votre famille et vos voisins cherchez les soins de santé pour des maladies causant de la fièvre/corps chaud et autres chante & symptômes. Cette

For peer review only - http://bmjopen.bmj.com/site/about/guidelines.xhtml

information permettra de mieux estimer le fardeau des maladies communes et d'identifier les moyens pour la mise en place de mesures de prévention et de traitement pour les maladies.

Procédures

Nous visiterons environ [ajouter le nombre pour site d'étude et pays] ménages au total à [ajouter le site et pays] et poserons les mêmes questions dans chaque ménage. Votre ménage a été choisi pour cette étude simplement par hasard. Si vous acceptez de participer, nous vous poserons, au nom de tous les membres du ménage, quelques questions au sujet de votre ménage tels que les initiales, le sexe et l'âge de chaque membre. Nous aimerions savoir certaines informations relatives à l'éducation et l'occupation du chef de ménage, au revenu, à la possession de biens, au logement lui-même ainsi qu'à l'hygiène et à l'assainissement dans le contexte de l'hygiène personnelle, de la préparation et de la manipulation des aliments, des sources d'eau et de la manipulation, de l'élimination des déchets, de la manipulation des animaux de ferme et domestiques ainsi que de l'élevage de ce ménage. Nous allons également demander où les membres de votre ménage obtiennent les soins si quelqu'un a une maladie causant de la fièvre ou toute autre maladie et les facteurs qui peuvent influer sur l'utilisation des soins de santé des membres du ménage (c'est-à-dire les modalités de transport, la disponibilité d'assurance maladie, les facteurs culturels) en plus du statut vaccinal et de la perception de certaines maladies. Enfin, nous allons mesurer la position géographique de votre maison. Il n'y a pas de bonnes ou de mauvaises réponses. Vous êtes libre de choisir les questions à répondre. Somme toute, il devrait prendre environ 30-45 minutes pour répondre à toutes les questions. L'entretien peut prendre un peu plus de temps s'il y a beaucoup de membres dans ce ménage. En outre, nous visiterons votre ménage deux fois en différentes saisons (une fois pendant la saison sèche et une fois vers la fin de la saison des pluies) pendant la période totale de l'étude qui est de deux ans environ pour recueillir des données comme décrit dans ce paragraphe du formulaire de consentement.

Participation

Vous, le décideur principal pour le comportement de santé quotidienne et l'utilisation des soins de santé dans ce ménage, ainsi que les membres de votre ménage, êtes libres de choisir d'accepter ou de refuser de participer à l'étude. Il n'y a aucun problème si vous ne voulez pas participer. Il n'y a non plus aucun problème si vous voulez arrêter votre participation à tout moment, et cela ne causera aucun tord à vous ou aux membres de votre ménage. Donc, la participation à cette étude est volontaire, et vous êtes libre de refuser de participer à l'étude ou vous pouvez retirer votre consentement à tout moment sans donner de raisons et ceci n'entraînera aucune pénalité.

Risques et avantages

La participation à cette étude n'entraine pas de risque direct. Poser des questions au sujet de votre ménage peut mettre mal à l'aise vous ou les membres de votre ménage. Après avoir terminé les questions, l'équipe de recherche pourra fournir des informations sur les soins de santé communautaires pour les participants qui expriment de la détresse. Vous pouvez refuser de répondre à toute question à tout moment. Vous pouvez faire une pause ou arrêter de participer à cette étude à tout moment. Il n'y a pas d'avantages directs à être dans cette étude. Au cours des visites de votre ménage, notre équipe de recherche peut identifier les membres de votre ménage qui devraient chercher des soins de santé pour les maladies causant de la fièvre et autres. Par conséquent, elle peut aider à fournir de l'information sur l'offre de soins établie dans votre communauté. Les avantages indirects sont que toutes les informations collectées contribueront à fournir des données exactes sur les problèmes de santé, y compris le statu socio-économique, le comportement d'hygiène et d'assainissement et le statut vaccinal des enfants dans votre communauté. Cela aidera à donner une meilleure compréhension des facteurs influençant la transmission de la maladie et l'infection, et à estimer le fardeau des maladies courantes ainsi qu'à trouver des moyens de prévenir et de traiter les personnes dans votre communauté.

Coûts et Compensations

Il n'a pas de coûts pour les participants de cette étude. Ni vous ni aucune personne de votre ménage ne sera indemnisée d'avoir participé.

Confidentialité

Tous les fichiers de l'étude et de toutes les informations qui identifient vous et les membres de votre ménage seront gardées confidentielles. Tous les questionnaires sur papier seront gardés en toute sécurité et les fichiers informatisés seront protégés par un mot de passe; tous les fichiers de données (sur papier/informatisés) seront stockées pour un minimum de cinq ans; ces fichiers seront accessibles seulement à quelques personnes autorisées de l'étude. Ni votre nom ni aucun identifiant ne sera utilisé dans les publications ou les rapports de

For peer review only - http://bmjopen.bmj.com/site/about/guidelines.xhtml

cette étude. Les informations que nous collectons à votre sujet et au sujet des membres de votre ménage seront partagées uniquement avec le personnel de l'étude autorisé et des représentants du comité d'éthique.

Personnes de contact pour des questions
Si vous ou des membres de votre ménage avez des questions, s'il vous plaît, demandez maintenant à l'équipe de recherche ou à tout moment plus tard en contactant [ajouter le nom de l'investigateur principal et/ou du co-investigateur, leurs affiliations respectives et les numéros de téléphone]. Si vous avez des questions sur vos droits ou ceux des membres du ménage en tant que participant à l'étude, vous pouvez contacter [ajouter le nom et les coordonnées du comité d'éthique local].

Déclaration de consentement
Ce formulaire de consentement pour participer à l'évaluation des soins de santé et d'autres facteurs liés à la population à [ajouter le site et pays] a été bien lu par moi/pour moi. L'objectif, les procédures, les risques et les avantages ont m'ont été expliqués en détails. J'ai été autorisé à poser des questions à tout moment, et mes questions ont été répondues à ma satisfaction par l'équipe de recherche. On m'a dit la personne à contacter en cas de questions ou si je veux discuter de problèmes ou préoccupations. On m'a dit que je recevrai une copie signée et datée de ce formulaire de consentement. Je suis rassuré que toutes les informations obtenues par suite de cette étude seront gardées confidentielles et utilisées uniquement aux fins de cette étude, et seulement par les institutions participantes.

Moi, le répondant, consens, par la présente, volontairement à participer à cette étude. Je suivrai les instructions de l'équipe de recherche et donnerai mon entière collaboration. Je comprends que j'ai le droit de me retirer de l'étude à tout moment.

RÉPONDANT

Nom : ____
(prénom + deuxième prénom (si disponible) + nom de famille)

Signature : ____
(Représentation manuscrite du nom/ marque que la personne écrit sur un document comme une preuve d'identité)

Date ______/______/______________ (YY/MM/AAAA)

Empreinte du pouce

Si le répondant ne peut pas signer/est analphabète, mais accepte de participer, enregistre l'empreinte du pouce dans la boîte ci-dessus, et demande à un témoin adulte, alphabétisé et indépendant de l'étude (par exemple membre du même ménage ou un voisin), de signer ci-dessous :

TÉMOIN

Nom : ____
(prénom + deuxième prénom (si disponible) + nom de famille)

Signature : ____
(Représentation manuscrite du nom/ marque que la personne écrit sur un document comme une preuve d'identité)

Date ______/______/______________ (YY/MM/AAAA)

Moi, l'enquêteur, j'ai lu/expliqué l'étude au répondant nommé ci-dessus (témoin si le répondant est analphabète) dans une langue que le répondant comprend bien. Je suis certain que le répondant a compris l'information et accepte qu'on lui pose des questions.

INTERVIEWER

Nom : ____
(prénom + deuxième prénom (si disponible) + nom de famille)

Signature : ____
(Représentation manuscrite du nom/ marque que la personne écrit sur un document comme une preuve d'identité)

Date ______/______/______________ (YY/MM/AAAA)

For peer review only - http://bmjopen.bmj.com/site/about/guidelines.xhtml

Site ___ ___ ___ Sous-zone ___ ___ ___ Numéro d'habitation ___ ___ ___ ___ Identifiant de l'enquêteur ___ __

FORMULAIRE 3: INFORMATIONS GÉNÉRALES SUR LE MÉNAGE

Instructions pour l'enquêteur

- Compléter ce formulaire uniquement si la personne interrogée a consenti à participer (voir « Formulaire 2 — FORMULAIRE DE CONSENTEMENT ») ; la personne interrogée ainsi que définie pour cette enquête est un adulte (selon l'âge légal de majorité du pays), un membre du ménage et un décideur en ce qui concerne les soins de santé quotidiens et l'utilisation de soins de santé pour le ménage entier et ses membre ; il ne doit pas être, par exemple, un voisin ou un visiteur.
- Recueillir les données de tous les membres du ménage que tous les membres du ménage soient présents ou non au moment de l'entretien ; ne recueillir et n'inscrire les données que des personnes présentes au moment de l'entretien fausserait les données.
- Un ménage est défini/les membres d'un ménage sont définis comme une personne ou un groupe de personnes avec ou sans lien de parenté qui vivent ensemble dans la même unité d'habitation, qui reconnaissent un adulte de sexe masculin ou féminin comme chef de ménage, qui partagent les mêmes installations domestiques, qui sont considérés comme constituant une unité, et qui subviennent à leurs besoins en termes de nourriture et des autres éléments essentiels pour vivre.
- « Site » et « sous-zone » doivent être un numéro à trois chiffres, « numéro d'habitation » un numéro à quatre chiffres, et « identifiant de l'enquêteur » un numéro à deux chiffres (utiliser les listes préparées individuellement pour chaque site pour obtenir les codes) ; saisir « date » au format JJ/MM/AAAA et « heure » au format HH:MM et entourer MATIN ou APRÈS-MIDI. «Étiquette de l'étude» doit être une combinaison d'un code «site» à trois chiffres, d'un code «sous-zone» à trois chiffres et d'un code «numéro d'habitation» à quatre chiffres; le « identifiant de l'enquêteur » doit être un numéro à trois chiffres.

FORMULAIRE 3: INFORMATIONS GÉNÉRALES SUR LE MÉNAGE
PARTIE A – DONNÉES DÉMOGRAPHIQUES/ INFORMATIONS GÉNÉRALES SUR LE MÉNAGE

EMPLACEMENT DU DOMICILE

1 Numéros de site, de sous-zone et d'habitation

Instructions : Site (=site d'étude) et sous-zone (=plus petite unité du site d'étude) doivent être tous les deux des numéros à trois chiffres, et numéro d'habitation doit être un code à quatre chiffres ; utiliser les listes préparées individuellement pour obtenir les codes respectifs.

Site: ____ ____ ____ Sous-zone: ____ ____ ____ Numéro d'habitation: ____ ____ ____ ____

2 Coordonnées GPS

Instructions : Prendre les coordonnées GPS approximativement au centre de la maison. Inscrire l'altitude par un nombre à quatre chiffres (unité : mètre), et la latitude/longitude par un numéro à huit chiffres (unités : degrés, minutes décimales).

O 1=Ménage d'origine O 2=Ménage de remplacement (côte droit/gauche du ménage d'origine)

Altitude: ___ ___ ___ ___

Latitude: ___ ___°___ ___.___ ___ ___' Longitude: ___ ___°___ ___.___ ___ ___'

PERSONNE INTERROGÉE

3 Inscrire le nom complet de la personne interrogée.

Instructions : Inscrire le prénom, le deuxième nom (le cas échéant) et le nom de famille de la personne interrogée.

____

4 Inscrire l'âge de la personne interrogée.

Instruction : Utiliser un numéro à trois chiffres (unité: année). _____ _____ _____

5 Inscrire le sexe de la personne interrogée.

O 1=Masculin O 2=Féminin

6 Quel est le lien de la personne interrogée avec le ménage ?

O 1=Chef de ménage
O 7=Conjoint du chef de ménage
O 2=Fils/fille du chef de ménage
O 8=Conjoint du fils/fille du chef de ménage

For peer review only - http://bmjopen.bmj.com/site/about/guidelines.xhtml

- O 3=Frère/sœur du chef de ménage
- O 4=Mère/père du chef de ménage
- O 5=Petite-fille/petit-fils du chef de ménage
- O 6=Autre, préciser __________
- O 9=Conjoint du frère/sœur du chef de ménage
- O 10=Belle-mère/beau-père du chef de ménage
- O 11=Nièce/neveu du chef de ménage
- O 98=Pas de réponse

MEMBRES DU MÉNAGE

7 Combien de membres du ménage vivent dans ce domicile au total (au moment de la visite) **?**

Instruction : Utiliser un numéro à deux chiffres.

Nombre total de membres du ménage : ____ ____

8 Inscrire l'âge et le sexe de chaque membre du ménage.

Instructions : Inscrire les initiales, l'âge et le sexe de la personne interrogée en première position/ligne du tableau. Les initiales, l'âge et le sexe de tous les autres membres du ménage doivent être inscrits ensuite. Les initiales d'un membre du ménage consistent en les trois premières lettres du prénom et les trois première lettre du nom de famille (un éventuel deuxième nom ne fait pas partie des initiales). Inscrire l'âge de chaque membre du ménage avec un numéro à trois chiffres (unité : années) ; si l'âge d'un des membres du ménage ≤ 12 mois, inscrire 1 an comme âge. Merci de compléter un formulaire supplémentaire si le ménage a plus de vingt membres.

Membre du ménage Identifiant	Initiales [prénom/nom de famille]	Âge [années]	Sexe
1/Personne interrogée	____ ____ ____/____ ____ ____	____ ____ ____	O1=Masculin O2=Féminin
2	____ ____ ____/____ ____ ____	____ ____ ____	O1=Masculin O2=Féminin
3	____ ____ ____/____ ____ ____	____ ____ ____	O1=Masculin O2=Féminin
4	____ ____ ____/____ ____ ____	____ ____ ____	O1=Masculin O2=Féminin
5	____ ____ ____/____ ____ ____	____ ____ ____	O1=Masculin O2=Féminin
6	____ ____ ____/____ ____ ____	____ ____ ____	O1=Masculin O2=Féminin
7	____ ____ ____/____ ____ ____	____ ____ ____	O1=Masculin O2=Féminin
8	____ ____ ____/____ ____ ____	____ ____ ____	O1=Masculin O2=Féminin
9	____ ____ ____/____ ____ ____	____ ____ ____	O1=Masculin O2=Féminin
10	____ ____ ____/____ ____ ____	____ ____ ____	O1=Masculin O2=Féminin
11	____ ____ ____/____ ____ ____	____ ____ ____	O1=Masculin O2=Féminin
12	____ ____ ____/____ ____ ____	____ ____ ____	O1=Masculin O2=Féminin
13	____ ____ ____/____ ____ ____	____ ____ ____	O1=Masculin O2=Féminin
14	____ ____ ____/____ ____ ____	____ ____ ____	O1=Masculin O2=Féminin
15	____ ____ ____/____ ____ ____	____ ____ ____	O1=Masculin O2=Féminin
16	____ ____ ____/____ ____ ____	____ ____ ____	O1=Masculin O2=Féminin
17	____ ____ ____/____ ____ ____	____ ____ ____	O1=Masculin O2=Féminin
18	____ ____ ____/____ ____ ____	____ ____ ____	O1=Masculin O2=Féminin
19	____ ____ ____/____ ____ ____	____ ____ ____	O1=Masculin O2=Féminin
20	____ ____ ____/____ ____ ____	____ ____ ____	O1=Masculin O2=Féminin

For peer review only - http://bmjopen.bmj.com/site/about/guidelines.xhtml

FORMULAIRE 3: INFORMATIONS GÉNÉRALES SUR LE MÉNAGE
PARTIE B – DONNÉÉS SOCIO-ECONOMIQUES

ÉDUCATION

1 Le chef de ménage sait-il lire en [inscrire la langue officielle du site d'étude/pays] **?**

O 1=Oui O 2=Non O 99=Ne sait pas O 98=Pas de réponse

2 Le chef de ménage sait-il écrire en [inscrire la langue officielle du site d'étude/pays] **?**

O 1=Oui O 2=Non O 99=Ne sait pas O 98=Pas de réponse

3 Quel est le niveau d'éducation scolaire le plus élevé atteint par le chef de ménage ?

O 1=École primaire
O 2=Collège
O 3=Lycée
O 4=Plus élevé que le lycée
O 5= PAS D'ÉDUCATION
O 6=Autre, préciser ___________
O 99=Ne sait pas
O 98=Pas de réponse

PROFESSION/RESSOURCES FINANCIÈRES

4 Quelle est la profession principale du chef de ménage ?

Instructions : Inscrire le statut du jour de visite uniquement.

O 1=Groupe 1 : Cadres
(directeurs généraux, hauts fonctionnaires, législateurs ; cadres administratifs et commerciaux ; responsables de production et de services spécialisés ; responsables dans l'hôtellerie, la vente et d'autres services)

O 2=Groupe 2 : Professionnels
(scientifiques ou ingénieurs ; professionnels de santé ; professionnels de l'enseignement ; professionnels de l'administration et des affaires ; professionnels de l'information et des technologies de communication ; professionnels du droit, du social ou de la culture)

O 3=Groupe 3 : Techniciens et professions intermédiaires
(Professions intermédiaires des sciences et techniques ; professions intermédiaires de la santé ; professions intermédiaires de l'administration et des affaires ; professions intermédiaires du droit, du social, de la culture et assimilés ; techniciens de l'information et des communications)

O 4=Groupe 4 : Employés de bureau
(secrétaires et dactylographes ; employé de service clientèle ; employé de conservation des données et de la documentation ; autres employés de bureau)

O 5=Groupe 5 : Travailleurs des services et de la vente
(travailleurs du service aux personnes ; travailleurs de la vente ; travailleurs des soins aux personnes ; travailleurs des services de protection)

O 6=Groupe 6 : Travailleurs qualifiés de l'agriculture, la sylviculture et la pêche
(travailleurs qualifiés de l'agriculture orientés marché ; éleveurs d'animaux; travailleurs qualifiés de la sylviculture, la pêche et la chasse orientés marché ; agriculteurs, pêcheurs, chasseurs et cueilleurs de subsistance)

O 7=Groupe 7 : Travailleurs manuels et assimilés
(travailleurs de la construction et assimilés (à l'exception des électriciens) ; travailleurs de la métallurgie, sur machines et assimilés ; artisans et travailleurs de l'imprimerie ; travailleurs des métiers de l'électrique et de l'électronique ; travailleurs des métiers de la transformation des aliments, du bois, des vêtements et assimilés)

O 8=Groupe 8 : Opérateurs de machines et d'installations, et monteurs
(Opérateurs de machines et d'installations fixes ; monteurs ; conducteurs opérateurs d'installations mobiles)

O 9=Groupe 9 : Emplois non qualifiés
(agents d'entretien et assistants ; ouvriers de l'agricultures, la sylviculture et la pêche ; travailleurs des mines, de la construction, de l'industrie et des transports ; assistants à la préparation des aliments ; vendeurs et services de rue et assimilés ; travailleurs de la collecte des ordures et autres travailleurs non qualifiés)

O 10=Groupe 0 : Professions des forces armées
(officiers des forces armées ; sous-officiers des forces armées ; professions des forces armées, autres grades)

O 11=Autre, préciser __________
O 12=PAS DE PROFESSION O 99=Ne sait pas O 98=Pas de réponse

For peer review only

5 Quel est le montant moyen des revenus mensuels du chef de ménage en lien avec à la profession indiquée en question 4 ainsi que celui des autres membre du ménage ?

Instruction: Tick one answer per household head/enter one code per household member if applicable/if income available.

Chef de ménage	Membre 1	Membre 2	Membre 3
Code ____________	Code ____________	Code ____________	Code ____________
[1] <100USD<br>[2] ≥100 à <150USD	[3] ≥150 à <200USD<br>[4] ≥200 à <250USD	[5] ≥250USD<br>[6] PAS DE REVENU (chef de ménage; voir Q4)	[99] Ne sait pas<br>[98] Pas de réponse

6 Quels sont les autres sources ou les sources supplémentaires de revenus mensuels du ménage et quel en est le montant ?

Instruction : Cocher plusieurs cases si nécessaire ; il peut y avoir plus d'une réponse.

Autres sources/Sources supplémentaires	Revenus financiers mensuels globaux
O 1=Aides sociales<br>O 2=Assurances (publiques ou privées)<br>O 3=Prêt (d'amis, de membres de la famille, d'une banque)<br>O 4=Vente (bétail, récoltes, meubles, autres biens)<br>O 5=Économies personnelles<br>O 6=Autre, préciser ________________	O 1=<100 USD<br>O 2=≥100 à <150 USD<br>O 3=≥150 à <200 USD<br>O 4=≥200 à <250 USD<br>O 5=≥250 USD
O 7=PAS D'AUTRES SOURCES/DE SOURCES SUPPLÉMENTAIRES<br>O 99=Ne sait pas<br>O 98=Pas de réponse	

For peer review only

LOGEMENT/HABITATION

7 Les membres du ménage sont-ils propriétaires de l'habitation ?

O 1=Oui O 3=Non, logement fourni (par le gouvernement, un employeur) O 99=Ne sait pas
O 2=Non, locataires O 4=Non, autres, préciser ________ O 98=Pas de réponse

8 Combien de pièces ou structures individuelles de l'habitation sont utilisées pour dormir, vivre, faire la cuisine ou se laver ?

Instruction : N'indiquer qu'un code par catégorie.

Chambre	Salon	Cuisine	Salle de bain
Code ____________	Code ____________	Code ____________	Code ____________
[1] 1-2<br>[2] 3-4	[2] >4<br>[2] Aucune	[99] Ne sait pas<br>[98] Pas de réponse	

9 En quel matériau sont construits le sol, les murs et le toit des pièces/des structures individuelles de l'habitation ?

Instruction : Indiquer tous les codes par catégorie ; plus d'une réponse par catégorie est possible.

Sol	Murs	Toit
Code ____________	Code ____________	Code ____________
[1] Brique [3] Tuiles<br>[2] Ciment/Béton [4] Terre	[5] Bois [7] Métal/Étain<br>[6] Paille/feuilles [8] Autre, préciser_________	[99] Ne sait pas<br>[98] Pas de réponse

10 Les membres de ce ménage ont-ils accès à/utilisent-ils l'électricité dans cette habitation ?

O 1=Oui O 2=Non O 99=Ne sait pas O 98=Pas de réponse

11 À quelle source de lumière principale les membres de ce ménage ont-ils accès/Quelle source de lumière principale utilisent-ils ?

O 1=Lumière/ampoule(s) électrique(s) O 4=Lampe(s) à piles O 7= Autre, préciser_____
O 2=Lumière du soleil/Lampe(s) solaire(s) O 5=Bougie(s) O 99=Ne sait pas
O 3=Lampe(s) à pétrole/à huile O 6=PAS DE SOURCE DE LUMIÈRE O 98=Pas de réponse

For peer review only - http://bmjopen.bmj.com/site/about/guidelines.xhtml

BIENS DU MÉNAGE

12 Parmi les articles suivants, lesquels les membres du ménage possèdent-ils ?

Instructions : Cocher plusieurs cases si nécessaire ; il peut y avoir plus d'une réponse ; indiquer la quantité d'articles fonctionnels si nécessaire

O 1=Radio	Quantité ____	O Rien	O 11=Lecteur cassettes/CD	Quantité ____	O Rien
O 2=Télévision	Quantité ____	O Rien	O 12=Ordinateur de bureau/portable/tablette	Quantité ____	O Rien
O 3=Connexion internet	Quantité ____	O Rien	O 13=Téléphone portable	Quantité ____	O Rien
O 4=Voiture	Quantité ____	O Rien	O 14=Camion/bus	Quantité ____	O Rien
O 5=Moto	Quantité ____	O Rien	O 15=Vélo	Quantité ____	O Rien
O 6=Char à bœufs/ânes	Quantité ____	O Rien	O 16=Lit	Quantité ____	O Rien
O 7=Matelas	Quantité ____	O Rien	O 17=Moustiquaire	Quantité ____	O Rien
O 8=Table	Quantité ____	O Rien	O 18=Chaise	Quantité ____	O Rien
O 9=Réfrigérateur (4°C)	Quantité ____	O Rien	O 19=Congélateur (-20°C)	Quantité ____	O Rien
O 10=Ventilateur	Quantité ____	O Rien	O 20=Machine à coudre	Quantité ____	O Rien

For peer review only

For peer review only - http://bmjopen.bmj.com/site/about/guidelines.xhtml

FORMULAIRE 3: INFORMATIONS GÉNÉRALES SUR LE MÉNAGE
Partie C – Hygiène et installations sanitaires

TOILETTES

1 À quel type de toilettes les membres du ménage ont-ils habituellement accès ?

Instruction : À l'intérieur signifie à l'intérieur d'une pièce/d'une habitation individuelle/d'un bâtiment ; à l'extérieur signifie à l'extérieur d'une pièce/d'une habitation individuelle/d'un bâtiment.

- O 1=Toilettes avec chasse d'eau/Cabinets (à l'intérieur)
- O 2=Toilettes avec chasse d'eau/Cabinets (à l'extérieur)
- O 3=Latrines sans chasse/latrines à fosse (à l'intérieur)
- O 4=Latrines sans chasse/latrines à fosse (à l'extérieur)
- O 5=Latrines à fosse améliorées par une ventilation (KVIP, à l'intérieur)
- O 6=Latrines à fosse améliorées par une ventilation (KVIP, à l'extérieur)
- O 7=Toilettes à compostage (à l'intérieur)
- O 8=Toilettes à compostage (à l'extérieur)
- O 9=PAS de toilettes, utilisent un seau
- O 10=PAS de toilettes, creusent un trou
- O 11=PAS de toilettes, défécation libre/en plein air
- O 12=Autre, préciser __________
- O 99=Ne sait pas
- O 98=Pas de réponse

2 Les membres du ménage utilisent-ils habituellement les toilettes indiquées à la question 1 ?

- O 1=Non, les membres ne peuvent utiliser les toilettes de façon autonome (bébés, enfants en bas-âge)
- O 2=Non, les membres ne peuvent utiliser les toilettes de façon autonome (handicapés, malades chroniques/longue durée)
- O 3=Non, les membres ne peuvent utiliser les toilettes de façon autonome (personnes âgées)
- O 4=Non, accès limité (publiques, partagées avec d'autres ménages/communautés)
- O 5=Non, accès limité (publiques, partagées avec d'autres ménages/communautés)
- O 6= Non, mauvais état (cassé, pas propre)
- O 7=OUI
- O 8=Autre, préciser _________________
- O 99=Ne sait pas
- O 98=Pas de réponse

3 Où sont situées les toilettes indiquées à la question 1 ? Par combien de personnes sont-elles habituellement partagées ?

- O 1=Sur la propriété du ménage (privées, non partagées)
- O 2=Sur la propriété d'un ménage voisin (privées, partagées)
  - *Partagées par* O 1=<10 personnes O 2=11-24 personnes O 3=25-49 personnes O 4=>50 personnes O 99=Ne sait pas O 98=Pas de réponse
- O 3=Dans la communauté (publiques, partagées)
  - *Partagées par* O 1=<10 personnes O 2=11-24 personnes O 3=25-49 personnes O 4=>50 personnes O 99=Ne sait pas O 98=Pas de réponse
- O 4=Autre, préciser__________ O 99=Ne sait pas O 98=Pas de réponse

4 Comment les membres du ménage qui ont accès/n'ont pas accès aux toilettes se lavent-ils habituellement les mains tout de suite avant et après avoir uriné/déféqué ?

Instruction : Indiquer un code par catégorie uniquement.

Lavent les mains avant d'avoir uriné/déféqué	Lavent les mains après avoir uriné/déféqué
Code _____________	Code _____________
[1] Lavent les mains avec du savon et de l'eau<br>[2] Lavent les mains avec de l'eau uniquement<br>[3] Frottent les mains avec des feuilles/de la paille/de l'herbe<br>[4] Frottent les mains avec du sable<br>[5] Frottent les mains avec un chiffon/du tissu	[6] Ne lavent pas, pas besoin/pas sale<br>[7] Ne lavent pas, rien pour laver les mains<br>[8] Autre, préciser __________<br>[99] Ne sait pas<br>[98] Pas de réponse

CUISINE/MANIPULATION DES ALIMENTS

5 Les membres de ce ménage ont-ils une cuisine pour faire cuire/préparer la nourriture/les repas ? Où est-elle située ?

- O 1=Oui, cuisine intérieure/dans une pièce/structure individuelle d'habitation ; sur la propriété du ménage (privée, non partagée)
- O 2=Oui, cuisine extérieure/hors d'une pièce/structure individuelle d'habitation/ en plein air ; sur la propriété du ménage (privée, non partagée)

For peer review only - http://bmjopen.bmj.com/site/about/guidelines.xhtml

○ 3=Oui, cuisine intérieure/dans une pièce/structure individuelle d'habitation ; sur la propriété d'un ménage voisin (privée, partagée)
○ 4=Oui, cuisine extérieure/hors d'une pièce/structure individuelle d'habitation/ en plein air ; sur la propriété d'un ménage voisin (privée, partagée)
○ 5=PAS DE CUISINE
○ 6=Autre, préciser ___________ ○ 99=Ne sait pas ○ 98=Pas de réponse

6 Quelle source d'énergie les membres de ce ménage utilisent-ils habituellement pour cuisiner/préparer la nourriture/les repas ?

○ 1=Électricité ○ 3=Charbon de bois ○ 5=Excréments d'animaux ○ 99=Ne sait pas
○ 2=Gaz/pétrole ○ 4=Bois/paille/feuilles ○ 6=Autre, préciser ___________ ○ 98=Pas de réponse

7 Quelle moyen de cuisson les membres de ce ménage utilisent-ils habituellement pour cuisiner/préparer la nourriture/les repas ?

○ 1=Foyer ouvert ○ 3=Fourneau intérieur avec cheminée ○ 99=Ne sait pas
○ 2=Fourneau extérieur ○ 4=Autre, préciser ___________ ○ 98=Pas de réponse

8 Où les membres de ce ménage conservent-ils habituellement les produits alimentaires frais/crus (comme les légumes, la salade, les fruits, les produits laitiers, les œufs, la viande et le poisson) et les restes/les aliments/repas préparés ?

Instruction : Indiquer un code par catégorie uniquement. À l'intérieur signifie à l'intérieur d'une pièce/d'une habitation individuelle/d'un bâtiment ; à l'extérieur signifie à l'extérieur d'une pièce/d'une habitation individuelle/d'un bâtiment.

Des produits alimentaires frais/crus (légumes, salade, fruits, produits laitiers, œufs, viande et poisson)	Des restes/aliments/repas préparés
Code ______________	Code ______________

[1] À température ambiante (à l'intérieur, sur la propriété du ménage, privé)
[2] À température ambiante (à l'extérieur, sur la propriété du ménage, privé)
[3] À température ambiante (à l'intérieur, sur la propriété d'un ménage voisin, privé, partagé)
[4] À température ambiante (à l'extérieur, sur la propriété d'un ménage voisin, privé, partagé)
[5] Réfrigérateur (sur la propriété du ménage, privé)
[6] Réfrigérateur (sur la propriété d'un ménage voisin, privé, partagé)
[7] Congélateur (sur la propriété du ménage, privé)
[8] Congélateur (sur la propriété d'un ménage voisin, privé, partagé)
[9] Autre, préciser ________________ [10] PAS DE CONSERVATION [99] Ne sait pas [98] Pas de réponse

9 Si la réponse à la question 8 au sujet des produits alimentaires frais/crus et/ou des restes/aliments/repas préparés est «température ambiante», combien de temps les membres de ce ménage les conservent-ils habituellement?

Instruction: Indiquer un code par catégorie uniquement.

Des produits alimentaires frais/crus (légumes, salade, fruits, produits laitiers, œufs, viande et poisson)	Des restes/aliments/repas préparés
Code ______________	Code ______________

[1] <1 jour [3] 3-4 jours [5] Pas applicable [99] Ne sait pas
[2] 1-2 jours [4] >4 jours [98] Pas de réponse

10 Les membres de ce ménage couvrent-ils habituellement les restes/aliments/repas préparés? Réchauffent-ils/recuisent-ils les restes/aliments/repas préparés avant de les consommer?

Instruction : Indiquer un code par catégorie uniquement.

Couvrent les restes/les aliments/repas préparés	Réchauffent/Recuisent les restes/les aliments/repas préparés
Code ______________	Code ______________

[1] Oui [2] Non [99] Ne sait pas [98] Pas de réponse

For peer review only - http://bmjopen.bmj.com/site/about/guidelines.xhtml

11 Quel membre(s) de ce ménage cuisine(s)/prépare(s) la nourriture/les repas habituellement pour les autres membres de ce ménage ?

Instructions : Se référer à la question 9 du « Formulaire 3 Partie A – Données démographiques/Informations générales sur le ménage » et indiquer les initiales/ Identifiant (s) du membre.

O Membre du ménage, indiquer les initiales/identificateur(s) __________

O 99=Ne sait pas O 98=Pas de réponse

12 Comment le(s) membre(s) de la famille qui cuisine(nt)/prépare(nt) la nourriture/les repas habituellement pour les autres membres de ce ménage comme indiqué à la question 11 se prépare(nt)-t-il(s)/elle(s) habituellement avant et après la préparation des repas?

Instruction: Indiquer un code par catégorie uniquement.

Avant la préparation des repas	Après la préparation des repas
Code _____________	Code _____________
[1] Lavent les mains avec du savon et de l'eau<br>[2] Lavent les mains avec de l'eau uniquement<br>[3] Frottent les mains avec des feuilles/de la paille/de l'herbe<br>[4] Frottent les mains avec du sable<br>[5] Frottent les mains avec un chiffon/du tissu	[6] Ne lavent pas, pas besoin/pas sale<br>[7] Ne lavent pas, rien pour laver les mains<br>[8] Autre, préciser __________<br>[99] Ne sait pas<br>[98] Pas de réponse

13 Comment les membres de ce ménage se préparent-ils habituellement avant et après avoir mangé de la nourriture/des repas, y compris le membre du ménage qui cuisine/prépare la nourriture/les repas habituellement comme indiqué à la question 11?

Instruction : Indiquer un code par catégorie uniquement.

Avant la préparation des repas	Après la préparation des repas
Code _____________	Code _____________
[1] Lavent les mains avec du savon et de l'eau<br>[2] Lavent les mains avec de l'eau uniquement<br>[3] Frottent les mains avec des feuilles/de la paille/de l'herbe<br>[4] Frottent les mains avec du sable<br>[5] Frottent les mains avec un chiffon/du tissu	[6] Ne lavent pas, pas besoin/pas sale<br>[7] Ne lavent pas, rien pour laver les mains<br>[8] Autre, préciser __________<br>[99] Ne sait pas<br>[98] Pas de réponse

14 Comment les membres de ce ménage mangent-ils habituellement leur nourriture/leurs repas, y compris le membre du ménage qui cuisine/prépare la nourriture/les repas habituellement comme indiqué à la question 11?

O 1=Avec des couverts O 3=Autre, préciser __________ O 99=Ne sait pas

O 2=Avec les doints O 98=Pas de réponse

15 Les membres de ce ménage mangent-ils habituellement leur nourriture/leurs repas dans le même récipient, y compris le membre du ménage qui cuisine/prépare la nourriture/les repas habituellement comme indiqué à la question 11?

O 1=Oui O 2=Non O 99=Ne sait pas O 98=Pas de réponse

16 Où les membres de ce ménage obtiennent-ils habituellement leurs produits alimentaires frais/crus principaux comme les légumes, les fruits, les grains/céréales, les produits laitiers, les œufs, la viande et le poisson?

Instruction : Indiquer un code par catégorie uniquement.

Légumes, fruits, grains/céréales	Produits laitiers	Œufs, viande, poisson
Code _____________	Code _____________	Code _____________
[1] Agriculture vivrière (cour/arrière-cour)<br>[2] Supermarché/Épicerie<br>[3] Amis/famille<br>[4] Marché ouvert<br>[5] Vendeur de rue		[6] Agriculture vivrière (terre agricole)<br>[7] Boucher<br>[8] Autre, préciser ______<br>[99] Ne sait pas<br>[98] Pas de réponse

For peer review only - http://bmjopen.bmj.com/site/about/guidelines.xhtml

17 Les membres de ce ménage consomment/mangent-ils des produits alimentaires frais/crus (comme les légumes, la salade, les fruits, les produits laitiers, la glace, les œufs, la viande et le poisson) ? Si oui, lesquels?

Instruction : Cocher plusieurs cases si nécessaire ; plus d'une réponse est possible.

O 1=Légumes/salade (cultivés sur arbuste)
O 2=Légumes/salade (récolte sauvage sur arbuste)
O 3=Fruits (cultivés sur arbuste/arbre)
O 4=Fruits (récolte sauvage sur arbuste/arbre)
O 5=Produits laitiers (lait, fromage, yaourt d'animaux domestiques produisant du lait), préciser ____________
O 6=Œufs (de volailles domestiques), préciser _________
O 7=Sang animal (d'animaux domestiques), domestiques), préciser _________
O 8=Bœuf/Zébu/Buffle (domestiqué), préciser _________
O 9=Porc (domestiqué)
O 10=Mouton (domestiqué)
O 11=Viande d'animaux non-domestiqués (antilope, sanglier, volaille, rongeurs, poisson), préciser ________
O 12=PAS DE CONSOMMATION de produits frais/crus
O 13=Légumes/salade (cultivés au sol)
O 14=Légumes/salade (récolte sauvage sur le sol)
O 15=Fruits (cultivés au sol)
O 16=Fruits (récolte sauvage sur le sol)
O 17=Produits laitiers (lait, fromage, yaourt d'animaux non-domestiques produisant du lait), préciser ____________
O 18= Œufs (de volailles non-domestiques), préciser _________
O 19= Sang animal (d'animaux non- préciser _________
O 20=Volaille (domestiquée; poulet, canard, pigeon, oie, dinde), préciser _________
O 21=Chèvre (domestiquée)
O 22= Rongeurs (domestiqués), préciser ______
O 23=Poisson/fruits de mer, préciser ______
O 24=Glace
O 25=Autre, préciser _________
O 99=Ne sait pas O 98=Pas de réponse

18 Les membres de ce ménage consomment-ils habituellement des repas/produits alimentaires/boissons également en dehors de l'habitation ? Si oui, quel(s) membre(s) de ce ménage et où ?

Instructions : Se référer à la question 9 du « Formulaire 3 Partie A – Données démographiques/Informations générales sur le ménage » et indiquer les initiales du/des membre(s). Indiquer tous les codes nécessaires pour les membres du ménage respectifs.

Membre 1<br>Initiales/Identifiant : ___	Membre 2<br>Initiales/Identifiant : ___	Membre 3<br>Initiales/Identifiant : ___	Membre 4<br>Initiales/ Identifiant : ___
Code ____________	Code ____________	Code ____________	Code ____________
[1] Restaurant<br>[2] Chaîne de restauration rapide	[3] Cafétéria<br>[4] Vendeur de rue	[5] Autre, préciser ____<br>[6] Pas applicable	[99] Ne sait pas<br>[98] Pas de réponse

19 Quels/Quelles repas/produits alimentaires/boissons le/les membre(s) de ce ménage listé à la question 18 consomment-ils habituellement en dehors de l'habitation ?

Instructions : Se référer à la question 9 du « Formulaire 3 Partie A – Données démographiques/Informations générales sur le ménage » et indiquer/identifiant les initiales du/des membre(s). Indiquer jusqu'à 5 repas/produits alimentaires/boissons par membre du ménage. Vous pouvez vous référer aux produits alimentaires listés à la question 17. Passer cette question si aucun membre du ménage ne consomme les repas/les produits alimentaires/les boissons en dehord de la maison comme indiqué dans Q18.

Membre 1<br>Initiales/Identifiant : ____	Membre 2<br>Initiales/Identifiant : ____	Membre 3<br>Initiales/Identifiant : ____	Membre 4<br>Initiales/ Identifiant : ___
1: ______________<br>2: ______________<br>3: ______________<br>4: ______________<br>5: ______________	1: ______________<br>2: ______________<br>3: ______________<br>4: ______________<br>5: ______________	1: ______________<br>2: ______________<br>3: ______________<br>4: ______________<br>5: ______________	1: ______________<br>2: ______________<br>3: ______________<br>4: ______________<br>5: ______________

EAU

20 Quelle est la source d'approvisionnement en eau principale des membres de ce ménage pour la boisson, la cuisine/la préparation des aliments/repas, le bain et le ménage (de

For peer review only - http://bmjopen.bmj.com/site/about/guidelines.xhtml

l'habitation/de la/les structure(s) individuelle(s) d'habitation(s), les vêtements/les outils de cuisine) ?

Instruction : Indiquer un code par catégorie uniquement.

Boisson	Cuisine	Bain	Nettoyage
Code ____________	Code ____________	Code ____________	Code ____________

[1] Eau du robinet (à l'intérieur de l'habitation ; privée)
[2] Eau du robinet (à l'extérieur de l'habitation : cour/arrière-cour ; privée)
[3] Eau du robinet (à l'extérieur de l'habitation ; publique/partagée)
[4] Borne-fontaine/eau courante (à l'intérieur de l'habitation ; privée)
[5] Borne-fontaine/eau courante (à l'extérieur de l'habitation : cour/arrière-cour ; privée)
[6] Borne-fontaine/eau courante (à l'extérieur de l'habitation ; publique/partagée)
[7] Camion-citerne (public ; partagé)
[8] Eaux de surface (canal, canal d'irrigation)
[9] Eaux de surface (lac, étang, rivière, ruisseau)
[10] Eau de source
[11] Eau de pluie
[12] Puits/Puits de forage (en plein air/non couvert/non protégé)
[13] Puits/Puits de forage (couvert/protégé)
[14] Eau en bouteille
[15] Autre, préciser ________________ [99] Ne sait pas [98] Pas de réponse

21 Les membres de ce ménage traitent-ils habituellement l'eau pour la boisson, la cuisine/la préparation des aliments/des repas, le bain, et le nettoyage (de l'habitation/des/de la structure(s) individuelle(s) d'habitation(s), des vêtements/des outils de cuisine) avant la consommation ? Comment l'eau est-elle habituellement traitée ?

Instruction: Indiquer tous les codes nécessaires par catégorie ; plus d'une réponse par catégorie est possible.

Boisson	Cuisine	Bain	Nettoyage
Code ____________	Code ____________	Code ____________	Code ____________

[1] Non, pas du tout
[2] Non, seulement pour les malades du ménage
[3] Non, seulement selon la saison (saison sèche)
[4] Oui, par filtration (tissu, céramique, sable)
[5] Oui, par sédimentation
[6] Autre, préciser ______________
[7] Non, seulement pour les enfants du ménage
[8] Non, seulement selon la saison (saison humide)
[9] Oui, par ébullition
[10] Oui, par désinfection solaire
[11] Oui, par chloration
[99] Ne sait pas [98] Pas de réponse

22 Quel(s) membre(s) de ce ménage recueille(nt) habituellement l'eau potable comme indiqué à la question 20 pour les autres membres de ce ménage ?

Instructions : Se référer à la question 9 du « Formulaire 3 Partie A – Données démographiques/Informations générales sur le ménage » et indiquer/identifiant les initiales du membre.

O Membre du ménage, indiquer les initiales/identifiant(s) _________
O 99=Ne sait pas O 98=Pas de réponse

23 Comment les membres de ce ménage conservent-ils habituellement l'eau potable indiquée à la question 20?

O 1=Réservoir d'eau (non couvert)
O 2=Réservoir d'eau (couvert par chiffon/tissu, couvercle, feuilles)
O 3=Récipient (non couvert)
O 4=Récipient (couvert par chiffon/tissu, couvercle, feuilles)
O 5=Pot en terre (non couvert)
O 6=Pot en terre (couvert par chiffon/tissu, couvercle, feuilles)
O 7=Seau (non couvert)
O 10=Bol (couvert par chiffon/tissu, couvercle, feuilles)
O 11=Pot en bois (non couvert)
O 12= Pot en bois (couvert par chiffon/ tissu, couvercle, feuilles)
O 13= Récipient en peau (chèvre, mouton, bovins, non couvert)
O 14=Récipient en peau (chèvre, mouton, bovins ; couvert par un chiffon/du tissu, un couvercle, des feuilles
O 15=NON CONSERVÉE
O 16=Autre, préciser _________

For peer review only - http://bmjopen.bmj.com/site/about/guidelines.xhtml

O 8=Seau (couvert par chiffon/tissu, couvercle, feuilles) O 99=Ne sait pas
O 9=Bol (non couvert) O 98= Pas de réponse

24 Si les membres de ce ménage utilisent une source d'approvisionnement en eau potable partagée comme indiqué à la question 20, par combien de personnes cette eau potable est-elle partagée ?

O 1=<10 personnes O 3=25-49 personnes O 5=NON PARTAGÉE O 99=Ne sait pas
O 2=11-24 personnes O 4=>50 personnes O 98=Pas de réponse

TRAITEMENT DES DÉCHETS

25 Où les membres de ce ménage jettent-ils habituellement les eaux usées de, par exemple, la cuisine/la préparation des aliments/repas, le bain et/ou le ménage, et jettent-ils habituellement les déchets domestiques, les déchets humains/déjections ?

Instruction: Indiquer un code par catégorie uniquement.

Eaux usées de par exemple cuisine/préparation des aliments/ repas, bain et/ou ménage	Déchets domestiques	Déchets humains/déjections
Code _____________	Code _____________	Code _____________

[1] Sur le sol/surface libre proche de l'habitation (cour/arrière-cour, privé)
[2] Sur le sol/surface libre loin de l'habitation dans la communauté (partagé, public)
[3] Dans un trou proche de la maison (cour/arrière-cour, privé)
[4] Dans un trou loin de la maison dans la communauté (partagé, public)
[5] Versement dans des eaux de surface dans la communauté (lac, étang, rivière, ruisseau, public, partagé)
[6] Versement dans des eaux de surface dans la communauté (canal, canal d'irrigation, public, partagé)
[7] Versement dans de l'eau de source dans la communauté (public, partagé)
[8] Versement dans une fosse septique proche de l'habitation (cour/arrière-cour, proche de toilettes si disponibles, privé)
[9] Versement dans une fosse septique dans la communauté (partagé, public)
[10] Versement dans les égouts proches de la maison (cour/arrière-cour, privé)
[11] Versement dans les égouts dans la communauté (partagé, public)
[12] Autre, préciser _________________________ [99] Ne sait pas [98] Pas de réponse

26 Les membres de ce ménage traitent-ils habituellement les déchets domestiques de la maison au préalable avant de les jeter ?

O 1=Oui, incinération des déchets O 3=NON
O 2=Oui, séparation/tri des déchet dégradable et non dégradables O 4=Oui, Autre, préciser _________
O 99=Ne sait pas O 98=Pas de réponse

27 À quelle distance de la source d'approvisionnement en eau de ce ménage comme indiqué à la question 20 se trouve l'emplacement de l'élimination des eaux usées, des déchets domestiques et humains/excrétions de ce ménage comme indiqué à la question 25 ?

Instructions : Indiquer un code par catégorie uniquement. Indiquer la distance en mètres [m].

Eaux usées de, par exemple, cuisine/préparation des aliments/ repas, bain et/ou ménage	Déchets domestiques	Déchets humains/déjections
Code _____________	Code _____________	Code _____________

[1] <5 m [3] ≥25 à <50 m [5] ≥100 à <500 m [7] ≥1,000 m
[2] ≥5 à <25 m [4] ≥50 à <100 m [6] ≥500 à <1,000 m [99] Ne sait pas [98] Pas de réponse

28 À quelle distance de la source d'approvisionnement en eau principale de ce ménage pour la cuisine/la préparation des aliments/des repas comme indiqué à la question 20 se trouve l'emplacement de l'élimination des eaux usées, des déchets domestiques et humains/excrétions de ce ménage comme indiqué à la question 25 ?

Instructions : Indiquer un code par catégorie uniquement. Indiquer la distance en mètres [m].

Eaux usées de par exemple cuisine/préparation des aliments/ repas, bain et/ou ménage	Déchets domestiques	Déchets humains/excréments

For peer review only - http://bmjopen.bmj.com/site/about/guidelines.xhtml

Code ____________	Code ____________	Code ____________
[1] <5 m [3] ≥25 à <50 m<br>[2] ≥5 à <25 m [4] ≥50 à <100 m	[5] ≥100 à <500 m<br>[6] ≥500 à <1,000 m	[7] ≥1,000 m<br>[99] Ne sait pas [98] Pas de réponse

ANIMAUX DE FERME/DOMESTIQUES

29 Quel genre d'animaux/de bétail et d'animaux domestiques/de compagnie les membres de ce ménage possèdent-ils ?

Instruction : Cocher plusieurs cases par catégorie si nécessaire ; il peut y avoir plus d'une réponse par catégorie.

Animaux de ferme/bétail		Animaux domestiques/de compagnie
O 1=Bovin	O9=Chameau	O 1=Chien
O 2=Mouton	O 10=Rongeurs (lapin), préciser ____________	O 2=Rongeurs (lapin), préciser ____________
O 3=Âne	O 11=Chèvre	O 3=Chat
O 4= Volaille (i.e. poulet, canard, pigeon, oie, dinde), préciser ____________	O 12=Cheval	O 4=PAS D'ANIMAUX DOMESTIQUES/DE COMPAGNIE
O 5=Zébu	O 13=PAS D'ANIMAUX DE FERME/DE BÉTAIL	O 5=Autre, préciser ____________
O 6=Cochon	O 14=Autre, préciser ____________	O 99=Ne sait pas
O 7=Poisson	O 99=Ne sait pas	O 98=Pas de réponse
O 8=Buffle	O 98=Pas de réponse	

30 Où les membres de ce ménage gardent-ils habituellement les animaux de ferme/le bétail et les animaux domestiques/de compagnie de ce ménage comme indiqué à la question 29 ?

Instruction : Indiquer un code par catégorie uniquement.

Animaux de ferme/bétail	Animaux domestiques/de compagnie
O 1=Étable/Cage/Bassin d'eau sur la propriété de l'habitation (arrière-cour/cour)	O 1=Cage/Chaîne proche de l'habitation (arrière-cour/cour)
O 2=Étable/Cage/Bassin d'eau à l'extérieur de l'habitation dans la communauté	O 2= Cage/Chaîne à l'extérieur de l'habitation dans la communauté
O 3=Étable/Cage/Bassin d'eau à l'extérieur de la communauté	O 3= Cage/Chaîne à l'extérieur de la communauté (public)
O 4=Surface libre/Eaux de surface/Eau de source sur la propriété de l'habitation (habitation, arrière-cour/cour)	O 4=Surface libre proche de l'habitation (dans la maison, arrière-cour/cour)
O 5=Surface libre/Eaux de surface/Eau de source à l'extérieur de l'habitation dans la communauté	O 5=Surface libre à l'extérieur de l'habitation dans la communauté
O 6=Surface libre/Eaux de surface/Eau de source à l'extérieur de la communauté	O 6=Surface libre à l'extérieur de la communauté
O 7=Pas applicable (nulle part /pas d'animaux de ferme/bétail)	O 7=Pas applicable (nulle part/pas d'animaux domestiques/de compagnie)
O 8=Autre, préciser __________	O 8=Autre, préciser __________
O 99=Ne sait pas	O 99=Ne sait pas
O 98=Pas de réponse	O 98=Pas de réponse

31 Quelle est l'importance de l'élevage que les membres de ce ménage pratiquent ?

O 1=Pour leurs propres besoins («élevage d'arrière-cour»)
O 2=Pas applicable (pas d'élevage)
O 3=3=Demande massive/industrielle («élevage intensif»)
O 4=Autre, préciser __________
O 99=Ne sait pas
O 98=Pas de réponse

For peer review only - http://bmjopen.bmj.com/site/about/guidelines.xhtml

32 Quel approvisionnement en eau les membres de ce ménage utilisent-ils pour fournir de l'eau aux animaux de ferme/au bétail et aux animaux domestiques/de compagnie de ce ménage comme indiqué à la question 29 ?
Instruction : Indiquer un code par catégorie uniquement.

Animaux de ferme/bétail	Animaux domestiques/de compagnie
Code ____________	Code ____________

[1] Eau du robinet (à l'intérieur de l'habitation ; privée)
[2] Eau du robinet (à l'extérieur de l'habitation : cour/arrière-cour ; privée)
[3] Eau du robinet (à l'extérieur de l'habitation ; publique/partagée)
[4] Borne-fontaine/eau courante (à l'intérieur de l'habitation ; privée)
[5] Borne-fontaine/eau courante (à l'extérieur de l'habitation : cour/arrière-cour ; privée)
[6] Borne-fontaine/eau courante (à l'extérieur de l'habitation ; publique/partagée)
[7] Camion-citerne (public ; partagé)
[8] Eaux de surface (canal, canal d'irrigation)
[9] Eaux de surface (lac, étang, rivière, ruisseau)
[10] Eau de source
[11] Eau de pluie
[12] Puits/Puits de forage (en plein air/non couvert/non protégé)
[13] Puits/Puits de forage (couvert/protégé)
[14] Pas applicable (pas d'animaux de ferme/bétail; pas d'animaux domestiques/de compagnie)
[15] Autre, préciser ____________ [99] Ne sait pas [98] Pas de réponse

33 Les membres de ce ménage se lavent-ils habituellement les mains après avoir nourri, donné de l'eau et nettoyé l'étable/la cage/le bassin d'eau des animaux de ferme/du bétail de ce ménage comme indiqué à la question 29 ?
Instruction : Indiquer un code par catégorie uniquement.

Animaux de ferme/bétail	Animaux domestiques/de compagnie
Code ____________	Code ____________

[1] Lavent les mains avec du savon et de l'eau
[2] Lavent les mains avec de l'eau uniquement
[3] Frottent les mains avec des feuilles/de la paille/de l'herbe
[4] Frottent les mains avec du sable
[5] Frottent les mains avec un chiffon/du tissu
[6] Ne lavent pas, pas besoin/pas sale
[7] Ne lavent pas, rien pour laver les mains
[8] Autre, préciser __________
[9] Pas applicable (pas d'animaux de ferme/bétail ; pas d'animaux domestiques/de compagnie
[99] Ne sait pas [98] Pas de réponse

CULTURES AGRICOLES

34 Quel genre de cultures les membres de ce ménage font-ils pousser/cultivent-ils ?
Instruction : Cocher plusieurs cases si nécessaire ; il peut y avoir plus d'une réponse.

- O 1=Café
- O 2=Riz
- O 3=Fruits (cultivés sur arbustes/arbre)
- O 4=Fruits (cultivés au sol)
- O 5=Légumes/salade (cultivés sur arbustes/arbre)
- O 6=Légumes/salade (cultivés au sol)
- O 7=Céréales (blé, orge, avoine, maïs)
- O 8=Thé
- O 9=Noix de cajou
- O 10=Tabac
- O 11=Canne à Sucre
- O 12=Chad/Chat
- O 13=Bees/Honey
- O 14=Coton
- O 15=PAS DE CULTURES
- O 16=Autre, préciser __________
- O 99=Ne sait pas
- O 98=Pas de réponse

35 Où les champs/terres agricoles des membres de ce ménage se situent-ils/elles?

- O 1=On the property of the house (i.e. back-/courtyard)
- O 4=Outside the house in the community
- O 2=Outside the community
- O 5=Not applicable (no crop farming)
- O 3=Other, specify __________
- O 99=Don't know
- O 98=No response

For peer review only - http://bmjopen.bmj.com/site/about/guidelines.xhtml

36 Quelle est l'importance des cultures des membres de ce ménage?

- O 1=Leurs propres besoins (« agriculture d'arrière-cour »)
- O 2=2=Pas applicable (pas de cultures)
- O 3=Demande massive/industrielle (agriculture intensive)
- O 4=Autre, préciser __________
- O 99=Ne sait pas
- O 98= Pas de réponse

37 Les membres de ce ménage utilisent-ils habituellement des herbicides, pesticides et engrais pour les cultures indiquées à la question 34?

Instruction : Indiquer un code par catégorie uniquement.

Herbicides	Pesticides	Engrais
O 1=Oui, préciser l'herbicide ____________________	O 1=Oui, préciser le pesticide ____________________	O 1=Oui, préciser l'engrais ____________________
O 2=Pas applicable (pas de cultures)	O 2=Pas applicable (pas de cultures)	O 2=Pas applicable (pas de cultures)
O 3=Pas d'utilisation des herbicides	O 3=Pas d'utilisation des pesticides	O 3=Pas d'utilisation des engrais
O 99=Ne sait pas	O 99=Ne sait pas	O 99=Ne sait pas
O 98=Pas de réponse	O 98=Pas de réponse	O 98=Pas de réponse

38 Les membres de ce ménage se lavent-ils les mains après avoir travaillé dans les terres agricoles pour les cultures indiquées à la question 34 ?

- O 1=Oui, se lavent les mains avec du savon et de l'eau
- O 2=Oui, se frottent les mains avec des feuilles/de la paille/de l'herbe
- O 3=Oui, se frottent les mains avec des chiffons/du tissu
- O 4=Pas applicable (pas de cultures)
- O 5=Oui, se lavent les mains avec de l'eau uniquement
- O 6=Oui, se frottent les mains avec du sable
- O 7=Non, ne se lavent pas les mains
- O 8=Autre, préciser __________
- O 99=Ne sait pas
- O 98= Pas de réponse

For peer review only

For peer review only - http://bmjopen.bmj.com/site/about/guidelines.xhtml

Site ___ ___ ___ Sous-zone ___ ___ ___ Numéro d'habitation ___ ___ ___ ___ Identifiant de l'enquêteur ___ __

FORMULAIRE 4: ÉVALUATION DE L'UTILISATION GÉNÉRIQUE DES SOINS DE SANTÉ GLOBAUX, STRATIFIÉE SELON L'ÂGE ET LE SEXE

Partie A –Évaluation de l'utilisation *générique* des soins de santé

Instructions pour l'enquêteur

– Compléter ce formulaire uniquement si la personne interrogée a consenti à participer (voir « Formulaire 2 — FORMULAIRE DE CONSENTEMENT ») ; la personne interrogée ainsi que définie pour cette enquête est un adulte (selon l'âge légal de majorité du pays), un membre du ménage et un décideur en ce qui concerne les soins de santé quotidiens et l'utilisation de soins de santé pour le ménage entier et ses membres ; il ne doit pas être, par exemple, un voisin ou un visiteur.
– Se référer à « Formulaire 3 — Partie A — Informations générales sur le ménage » comme référence pour obtenir le nombre total de membres du ménage ainsi que l'âge et le sexe de chaque membre du ménage.
– Recueillir les données de tous les membres du ménage que tous les membre du ménage soient présents ou non au moment de l'entretien ; ne recueillir et n'inscrire les données que des personnes présentes au moment de l'entretien fausserait les données.
– Un ménage est défini/les membres d'un ménage sont définis comme une personne ou un groupe de personnes avec ou sans lien de parenté qui vivent ensemble dans la même unité d'habitation, qui reconnaissent un adulte de sexe masculin ou féminin comme chef de ménage, qui partagent les mêmes installations domestiques, qui sont considérés comme constituant une unité, et qui subviennent à leurs besoins en termes de nourriture et des autres éléments essentiels pour vivre.
– « Site » et « sous-zone » doivent être un numéro à trois chiffres, « numéro d'habitation » un numéro à quatre chiffres, et « identifiant de l'enquêteur » un numéro à deux chiffres (utiliser les listes préparées individuellement pour chaque site pour obtenir les codes) ; saisir « date » au format JJ/MM/AAAA et « heure » au format HH:MM et entourer MATIN ou APRÈS-MIDI. «Étiquette de l'étude» doit être une combinaison d'un code «site» à trois chiffres, d'un code «sous-zone» à trois chiffres et d'un code «numéro d'habitation» à quatre chiffres; le « identifiant de l'enquêteur » doit être un numéro à trois chiffres.
– Abréviation : EDS = Établissement de santé public/privé primaire/secondaire/tertiaire.

MEMBRES DU MÉNAGE

1 Indiquer le nombre total de membres du ménage de cette habitation (au jour de la visite).
Instructions : Le nombre total de membres du ménage doit être indiqué à l'aide d'un numéro à 2 chiffres ; voir également « Formulaire 3 — Partie A — INFORMATIONS GÉNÉRALES SUR LE MÉNAGE ».

Nombre total de membres du ménage : ____ ____

2 Indiquer le nombre de membres FÉMININS du ménage pour chacun des groupes d'âge suivants :
Instructions : Le nombre total de membres féminins du ménage par groupe d'âge doit être indiqué à l'aide d'un numéro à 2 chiffres ; voir également « Formulaire 3 — Partie A — INFORMATIONS GÉNÉRALES SUR LE MÉNAGE ».

<2 ans : ____ ____ ≥2 à <5 ans : ____ ____ ≥5 à <15 ans : ____ ____ ≥15 ans : ____ ____

3 Indiquer le nombre de membres MASCULINS du ménage pour chacun des groupes d'âge suivants :
Instructions : Le nombre total de membres masculins du ménage par groupe d'âge doit être indiqué à l'aide d'un numéro à 2 chiffres ; voir également « Formulaire 3 — Partie A — INFORMATIONS GÉNÉRALES SUR LE MÉNAGE ».

<2 ans : ____ ____ ≥2 à <5 ans : ____ ____ ≥5 à <15 ans : ____ ____ ≥15 ans : ____ ____

COMPORTEMENT DE RECHERCHE DE SOINS DE SANTÉ

4 Indiquer où les membres féminins/masculins du foyer d'âges <2 ans/≥2 à <5 ans/≥5 à <15 ans/≥15 ans recherchent habituellement des soins de santé pour les signes/symptômes suivants. Merci d'attribuer une note entre 1 et 4.

Instructions :
- Compléter la question 4 séparément pour les membres du ménage féminins et masculins d'âge <2 ans, ≥2 à <5 ans, ≥5 à <15 ans et ≥15 ans et entoure le sexe et le groupe d'âge pour lequel Q4 a été completée; passer la question 4 s'il n'y a aucun membre féminin/masculin du ménage d'âge <2 ans/ ≥2 à <5 ans/ ≥5 à <15 ans/ ≥15 ans.
- Indiquer une réponse pour chaque choix de soins de santé pour chaque signe/symptôme; attribuer une note de 1 à 4 (1=1er choix; 2=2ème choix; 3=3ème choix; 4=non choisi); indiquer un code s'il y a lieu en utilisant les listes préparées (utiliser listes préparées individuellement pour chaque site pour obtenir les codes); cocher «Ne sait pas» s'il y a lieu.

For peer review only - http://bmjopen.bmj.com/site/about/guidelines.xhtml

Signe/Symptôme	Choix de soins de santé									
	EDS SETA		Autre EDS		Médecin	Pharmacie	Guérisseur traditionnel	Nulle part Auto-médication	Nulle part Rien	Ne sait pas
	Note	Code	Note	Code	Note	Note	Note	Note	Note	Cocher
**1** [**A**] Fièvre	O=1<br>O=2<br>O=3<br>O=4		O=1<br>O=2<br>O=3<br>O=4		O=1<br>O=2<br>O=3<br>O=4	O=1<br>O=2<br>O=3<br>O=4	O=1<br>O=2<br>O=3<br>O=4	O=1<br>O=2<br>O=3<br>O=4	O=1<br>O=2<br>O=3<br>O=4	O
**2** Fièvre <3 jours (continus)	O=1<br>O=2<br>O=3<br>O=4		O=1<br>O=2<br>O=3<br>O=4		O=1<br>O=2<br>O=3<br>O=4	O=1<br>O=2<br>O=3<br>O=4	O=1<br>O=2<br>O=3<br>O=4	O=1<br>O=2<br>O=3<br>O=4	O=1<br>O=2<br>O=3<br>O=4	O
**3** Fièvre ≥3 jours (continus)	O=1<br>O=2<br>O=3<br>O=4		O=1<br>O=2<br>O=3<br>O=4		O=1<br>O=2<br>O=3<br>O=4	O=1<br>O=2<br>O=3<br>O=4	O=1<br>O=2<br>O=3<br>O=4	O=1<br>O=2<br>O=3<br>O=4	O=1<br>O=2<br>O=3<br>O=4	O
**4** Frissons Tremblements	O=1<br>O=2<br>O=3<br>O=4		O=1<br>O=2<br>O=3<br>O=4		O=1<br>O=2<br>O=3<br>O=4	O=1<br>O=2<br>O=3<br>O=4	O=1<br>O=2<br>O=3<br>O=4	O=1<br>O=2<br>O=3<br>O=4	O=1<br>O=2<br>O=3<br>O=4	O
**5** Convulsion	O=1<br>O=2<br>O=3<br>O=4		O=1<br>O=2<br>O=3<br>O=4		O=1<br>O=2<br>O=3<br>O=4	O=1<br>O=2<br>O=3<br>O=4	O=1<br>O=2<br>O=3<br>O=4	O=1<br>O=2<br>O=3<br>O=4	O=1<br>O=2<br>O=3<br>O=4	O
**6** Perte de poids	O=1<br>O=2<br>O=3<br>O=4		O=1<br>O=2<br>O=3<br>O=4		O=1<br>O=2<br>O=3<br>O=4	O=1<br>O=2<br>O=3<br>O=4	O=1<br>O=2<br>O=3<br>O=4	O=1<br>O=2<br>O=3<br>O=4	O=1<br>O=2<br>O=3<br>O=4	O
**7** Déshydratation	O=1<br>O=2<br>O=3<br>O=4		O=1<br>O=2<br>O=3<br>O=4		O=1<br>O=2<br>O=3<br>O=4	O=1<br>O=2<br>O=3<br>O=4	O=1<br>O=2<br>O=3<br>O=4	O=1<br>O=2<br>O=3<br>O=4	O=1<br>O=2<br>O=3<br>O=4	O
**8** Malaise Faiblesse/ Fatigue	O=1<br>O=2<br>O=3<br>O=4		O=1<br>O=2<br>O=3<br>O=4		O=1<br>O=2<br>O=3<br>O=4	O=1<br>O=2<br>O=3<br>O=4	O=1<br>O=2<br>O=3<br>O=4	O=1<br>O=2<br>O=3<br>O=4	O=1<br>O=2<br>O=3<br>O=4	O
**9** Douleurs (muscles/articulations /os)	O=1<br>O=2<br>O=3<br>O=4		O=1<br>O=2<br>O=3<br>O=4		O=1<br>O=2<br>O=3<br>O=4	O=1<br>O=2<br>O=3<br>O=4	O=1<br>O=2<br>O=3<br>O=4	O=1<br>O=2<br>O=3<br>O=4	O=1<br>O=2<br>O=3<br>O=4	O
**10** Maux de tête	O=1<br>O=2<br>O=3<br>O=4		O=1<br>O=2<br>O=3<br>O=4		O=1<br>O=2<br>O=3<br>O=4	O=1<br>O=2<br>O=3<br>O=4	O=1<br>O=2<br>O=3<br>O=4	O=1<br>O=2<br>O=3<br>O=4	O=1<br>O=2<br>O=3<br>O=4	O
**11** Vertiges/ Confusion Perte de connaissance	O=1<br>O=2<br>O=3<br>O=4		O=1<br>O=2<br>O=3<br>O=4		O=1<br>O=2<br>O=3<br>O=4	O=1<br>O=2<br>O=3<br>O=4	O=1<br>O=2<br>O=3<br>O=4	O=1<br>O=2<br>O=3<br>O=4	O=1<br>O=2<br>O=3<br>O=4	O
**12** Troubles de la pression artérielle	O=1<br>O=2<br>O=3<br>O=4		O=1<br>O=2<br>O=3<br>O=4		O=1<br>O=2<br>O=3<br>O=4	O=1<br>O=2<br>O=3<br>O=4	O=1<br>O=2<br>O=3<br>O=4	O=1<br>O=2<br>O=3<br>O=4	O=1<br>O=2<br>O=3<br>O=4	O
**13** Troubles cardiaques	O=1<br>O=2<br>O=3<br>O=4		O=1<br>O=2<br>O=3<br>O=4		O=1<br>O=2<br>O=3<br>O=4	O=1<br>O=2<br>O=3<br>O=4	O=1<br>O=2<br>O=3<br>O=4	O=1<br>O=2<br>O=3<br>O=4	O=1<br>O=2<br>O=3<br>O=4	O

For peer review only

For peer review only - http://bmjopen.bmj.com/site/about/guidelines.xhtml

Signe/Symptôme	Choix de soins de santé									
	EDS SETA		Autre EDS		Médecin	Pharmacie	Guérisseur traditionnel	Nulle part Auto-médication	Nulle part Rien	Ne sait pas
	Note	Code	Note	Code	Note	Note	Note	Note	Note	Cocher
**14** Nausée Vomissements	O=1<br>O=2<br>O=3<br>O=4		O=1<br>O=2<br>O=3<br>O=4		O=1<br>O=2<br>O=3<br>O=4	O=1<br>O=2<br>O=3<br>O=4	O=1<br>O=2<br>O=3<br>O=4	O=1<br>O=2<br>O=3<br>O=4	O=1<br>O=2<br>O=3<br>O=4	O
**15** Diarrhée (liquide/sanglante/ mucopurulente)	O=1<br>O=2<br>O=3<br>O=4		O=1<br>O=2<br>O=3<br>O=4		O=1<br>O=2<br>O=3<br>O=4	O=1<br>O=2<br>O=3<br>O=4	O=1<br>O=2<br>O=3<br>O=4	O=1<br>O=2<br>O=3<br>O=4	O=1<br>O=2<br>O=3<br>O=4	O
**16** Douleur abdominale Douleur intestinale	O=1<br>O=2<br>O=3<br>O=4		O=1<br>O=2<br>O=3<br>O=4		O=1<br>O=2<br>O=3<br>O=4	O=1<br>O=2<br>O=3<br>O=4	O=1<br>O=2<br>O=3<br>O=4	O=1<br>O=2<br>O=3<br>O=4	O=1<br>O=2<br>O=3<br>O=4	O
**17** Perte de sang/ Hémorragie (interne)	O=1<br>O=2<br>O=3<br>O=4		O=1<br>O=2<br>O=3<br>O=4		O=1<br>O=2<br>O=3<br>O=4	O=1<br>O=2<br>O=3<br>O=4	O=1<br>O=2<br>O=3<br>O=4	O=1<br>O=2<br>O=3<br>O=4	O=1<br>O=2<br>O=3<br>O=4	O
**18** Perte de sang/ Hémorragie (externe)	O=1<br>O=2<br>O=3<br>O=4		O=1<br>O=2<br>O=3<br>O=4		O=1<br>O=2<br>O=3<br>O=4	O=1<br>O=2<br>O=3<br>O=4	O=1<br>O=2<br>O=3<br>O=4	O=1<br>O=2<br>O=3<br>O=4	O=1<br>O=2<br>O=3<br>O=4	O
**19** Éternuements Nez qui coule	O=1<br>O=2<br>O=3<br>O=4		O=1<br>O=2<br>O=3<br>O=4		O=1<br>O=2<br>O=3<br>O=4	O=1<br>O=2<br>O=3<br>O=4	O=1<br>O=2<br>O=3<br>O=4	O=1<br>O=2<br>O=3<br>O=4	O=1<br>O=2<br>O=3<br>O=4	O
**20** Toux	O=1<br>O=2<br>O=3<br>O=4		O=1<br>O=2<br>O=3<br>O=4		O=1<br>O=2<br>O=3<br>O=4	O=1<br>O=2<br>O=3<br>O=4	O=1<br>O=2<br>O=3<br>O=4	O=1<br>O=2<br>O=3<br>O=4	O=1<br>O=2<br>O=3<br>O=4	O
**21** Respiration rapide/ essoufflement	O=1<br>O=2<br>O=3<br>O=4		O=1<br>O=2<br>O=3<br>O=4		O=1<br>O=2<br>O=3<br>O=4	O=1<br>O=2<br>O=3<br>O=4	O=1<br>O=2<br>O=3<br>O=4	O=1<br>O=2<br>O=3<br>O=4	O=1<br>O=2<br>O=3<br>O=4	O
**22** Difficultés respiratoires graves	O=1<br>O=2<br>O=3<br>O=4		O=1<br>O=2<br>O=3<br>O=4		O=1<br>O=2<br>O=3<br>O=4	O=1<br>O=2<br>O=3<br>O=4	O=1<br>O=2<br>O=3<br>O=4	O=1<br>O=2<br>O=3<br>O=4	O=1<br>O=2<br>O=3<br>O=4	O
**23** Inflammation de la peau (éruptions cutanées/ rougeurs/pus/ démangeaisons)	O=1<br>O=2<br>O=3<br>O=4		O=1<br>O=2<br>O=3<br>O=4		O=1<br>O=2<br>O=3<br>O=4	O=1<br>O=2<br>O=3<br>O=4	O=1<br>O=2<br>O=3<br>O=4	O=1<br>O=2<br>O=3<br>O=4	O=1<br>O=2<br>O=3<br>O=4	O
**24** Gonflement/ Œdème	O=1<br>O=2<br>O=3<br>O=4		O=1<br>O=2<br>O=3<br>O=4		O=1<br>O=2<br>O=3<br>O=4	O=1<br>O=2<br>O=3<br>O=4	O=1<br>O=2<br>O=3<br>O=4	O=1<br>O=2<br>O=3<br>O=4	O=1<br>O=2<br>O=3<br>O=4	O
**25** Jaunisse	O=1<br>O=2<br>O=3<br>O=4		O=1<br>O=2<br>O=3<br>O=4		O=1<br>O=2<br>O=3<br>O=4	O=1<br>O=2<br>O=3<br>O=4	O=1<br>O=2<br>O=3<br>O=4	O=1<br>O=2<br>O=3<br>O=4	O=1<br>O=2<br>O=3<br>O=4	O

For peer review only

For peer review only - http://bmjopen.bmj.com/site/about/guidelines.xhtml

Site ___ ___ ___ Sous-zone ___ ___ ___ Numéro d'habitation ___ ___ ___ ___ Identifiant de l'enquêteur ___ __

FORMULAIRE 4: ÉVALUATION DE L'UTILISATION GÉNÉRIQUE DES SOINS DE SANTÉ GLOBAUX, STRATIFIÉE SELON L'ÂGE ET LE SEXE

Partie B – Facteurs associés aux soins de santé/Vaccination/Perception de la maladie

DÉPLACEMENT À L'ÉTABLISSEMENT DE SANTÉ

1 Au cas où un établissement de santé (EDS) a été noté comme 1er choix de soins à la question 4A (fièvre), quel est habituellement le moyen de transport principal, le temps de trajet (selon le moyen de transport), le coût du trajet (selon le moyen de transport) et la distance du trajet (selon le moyen de transport) pour s'y rendre ?

Instructions : Indiquer uniquement un code par catégorie, sexe ainsi que groupe d'âge. Passer cette question s'il n'y a pas de membre féminin/masculin d'âge <2 ans/≥2 à <5 ans/≥5 à <15 ans/≥15 ans dans ce ménage (voir également questions 2 et 3 de ce formulaire Partie A). Passer cette question si aucun EDS n'a été noté comme 1er choix de soins à la question 4A pour les membres masculins/féminins d'âge <2 ans/≥2 à <5 ans/≥5 à <15 ans/≥15 ans. Indiquer le temps de trajet en minute(s) [min]/ heure(s) [h], le coût total en USD, y compris les cadeaux éventuels, les aides financières et les dons, et la distance totale en mètre(s) [m]/kilomètre(s) [km].

Membre féminin				Membre masculin			
<2 ans	≥2 à <5 ans	≥5 à <15 ans	≥15 ans	<2 ans	≥2 à <5 ans	≥5 à <15 ans	≥15 ans
**1.1 Moyen de transport**							
Code ____	Code ____	Code ____	Code ____	Code ____	Code ____	Code ____	Code ____
[1] Private transportation (i.e. motorbike, car, bus)<br>[2] Public transportation (i.e. motorbike, car, bus)				[3] Walking<br>[4] Bicycle	[5] Other, specify ________<br>[99] Don't know		[98] No response
**1.2 Temps de trajet**							
Code ____	Code ____	Code ____	Code ____	Code ____	Code ____	Code ____	Code ____
[1] <30 min<br>[2] ≥30 à <60 min		[3] ≥1.0 à <2.0 h<br>[4] ≥2.0 à <3.0 h		[5] ≥3.0 à <4.0 h<br>[6] ≥4.0h		[99] Ne sait pas<br>[98] Pas de réponse	
**1.3 Coût du transport**							
Code ____	Code ____	Code ____	Code ____	Code ____	Code ____	Code ____	Code ____
[1] <1.0 USD<br>[2] ≥1.0 à <3.0 USD		[3] ≥3.0 à <5.0 USD<br>[4] ≥5.0 à <10.0 USD		[5] ≥10.0 USD		[99] Ne sait pas<br>[98] Pas de réponse	
**1.4 Distance du trajet**							
Code ____	Code ____	Code ____	Code ____	Code ____	Code ____	Code ____	Code ____
[1] <500 m<br>[2] ≥500 m à <1.0 km		[3] ≥1.0 à <2.0 km<br>[4] ≥2.0 à <3.0 km		[5] ≥3.0 à <4.0 km<br>[6] ≥4.0 km		[99] Ne sait pas<br>[98] Pas de réponse	

ASSURANCE

2 Pour quels membres de ce ménage l'assurance couvre-t-elle les dépenses de santé ?

Instructions : Indiquer un code par catégorie. Passer cette question s'il n'y a aucun membre d'âge <2 ans/≥2 à <5 ans/≥5 à <15 ans/≥15 ans dans ce ménage (voir questions 2 et 3 de ce formulaire partie A).

<2 ans	≥2 à <5 ans	≥5 à <15 ans	≥15 ans
Code ____________	Code ____________	Code ____________	Code ____________
[1] Oui	[2] Non [99] Ne sait pas	[98] Pas de réponse	

3 Quels types de dépenses de santé sont habituellement couverts par l'assurance pour les membres de ce ménage ?

Instructions : Indiquer uniquement un code par catégorie et par groupe d'âge. Passer cette question s'il n'y a aucun membre d'âge <2 ans/≥2 à <5 ans/≥5 à <15 ans/≥15 ans dans ce ménage (voir également les questions 2 et 3 de ce formulaire partie

For peer review only - http://bmjopen.bmj.com/site/about/guidelines.xhtml

A). Passer cette question s'il n'y a aucune assurance qui couvre les dépenses de santé des membres d'âge <2 ans/≥2 à <5 ans/≥5 à <15 ans/≥15 ans.

	<2 ans	≥2 à <5 ans	≥5 à <15 ans	≥15 ans
	[1] Oui	[2] Non	[99] Ne sait pas	[98] Pas de réponse
**1** Inscription	Code _______	Code _______	Code _______	Code _______
**2** Consultation/Visite chez le médecin	Code _______	Code _______	Code _______	Code _______
**3** Examen physique - général	Code _______	Code _______	Code _______	Code _______
**4** Examen physique – spécifique, préciser_______	Code _______	Code _______	Code _______	Code _______
**5** Diagnostics - diagnostics généraux de laboratoire	Code _______	Code _______	Code _______	Code _______
**6** Diagnostics – diagnostics spécifiques de laboratoire, préciser__________	Code _______	Code _______	Code _______	Code _______
**7** Diagnostics Imagerie générale	Code _______	Code _______	Code _______	Code _______
**8** Diagnostics Imagerie spécifique, préciser__________	Code _______	Code _______	Code _______	Code _______
**9** Diagnostics Autre, préciser________	Code _______	Code _______	Code _______	Code _______
**10** Traitement Médication générale	Code _______	Code _______	Code _______	Code _______
**11** Traitement Médication spécifique, préciser________	Code _______	Code _______	Code _______	Code _______
**12** Traitement Autre médication, préciser__________	Code _______	Code _______	Code _______	Code _______
**13** Traitement Interventions (autres que chirurgie), préciser_______	Code _______	Code _______	Code _______	Code _______
**14** Traitement Chirurgie	Code _______	Code _______	Code _______	Code _______
**15** Hospitalisation <7 jours	Code _______	Code _______	Code _______	Code _______
**16** Hospitalisation ≥7 jours	Code _______	Code _______	Code _______	Code _______
**17** Services d'urgence	Code _______	Code _______	Code _______	Code _______
**18** Soins prénatals	Code _______	Code _______	Code _______	Code _______
**19** Soins postnatals	Code _______	Code _______	Code _______	Code _______
**20** Médecine préventive	Code _______	Code _______	Code _______	Code _______
**21** Vaccination	Code _______	Code _______	Code _______	Code _______
**22** Soins à domicile (personnes âgées, handicapés, malades chroniques/à long terme)	Code _______	Code _______	Code _______	Code _______
**23** Thérapie de soutien (physiothérapie, ergothérapie, réhabilitation)	Code _______	Code _______	Code _______	Code _______

For peer review only - http://bmjopen.bmj.com/site/about/guidelines.xhtml

ETHNOGRAPHIE

4 À quelle religion/groupe ethnique/tribu appartiennent les membres de ce ménage ?

Instructions : Donner une réponse par groupe d'âge uniquement. Passer cette question s'il n'y a aucun membre d'âge>2 ans/≥2 à <5 ans/≥5 à <15 ans/≥15 ans dans ce ménage (voir également questions 2 et 3 de ce formulaire Partie A).

	<2 ans	≥2 to <5 ans	≥5 to <15 ans	≥15 ans
Religion/Groupe ethnique/Tribu	______________	______________	______________	______________
	O 99=Ne sait pas<br>O 98=Pas de réponse	O 99=Ne sait pas<br>O 98=Pas de réponse	O 99=Ne sait pas<br>O 98=Pas de réponse	O 99=Ne sait pas<br>O 98=Pas de réponse

5 La religion/le groupe ethnique/la tribu comme indiqué à la question 4 influence-t-elle/il les différents aspects de l'utilisation des soins de santé pour les membres de ce ménage ?

Instructions : Indiquer un code par catégorie et groupe d'âge uniquement. Passer cette question s'il y a un membre d'âge >2 ans/≥2 à <5 ans/≥5 à <15 ans/≥15 ans dans ce ménage (voir également questions 2 t 3 de ce formulaire Partie A).

	<2 ans	≥2 à <5 ans	≥5 to <15 ans	≥15 ans
	[1] Oui	[2] Non	[99] Ne sait pas	[98] pas réponse
**1** Visite dans un établissement de santé (hôpital, centre de soins, poste de santé)	Code _____	Code _______	Code _______	Code _____
**2** Visite chez un médecin	Code _____	Code _______	Code _______	Code _____
**3** Visite dans une pharmacie	Code _____	Code _______	Code _______	Code _____
**4** Visite chez un guérisseur traditionnel	Code _____	Code _______	Code _______	Code _____
**5** Décision d'automédication	Code _____	Code _______	Code _______	Code _____
**6** Décision de non traitement	Code _____	Code _______	Code _______	Code _____
**7** Examen physique - général	Code _____	Code _______	Code _______	Code _____
**8** Examen physique – spécifique, préciser________	Code _____	Code _______	Code _______	Code _____
**9** Prélèvements d'échantillons (sang, urine, selles, frottis, écouvillons, liquide cérébro-spinal)	Code _____	Code _______	Code _______	Code _____
**10** Diagnostics - diagnostics généraux de laboratoire	Code _____	Code _______	Code _______	Code _____
**11** Diagnostics – diagnostics spécifiques de laboratoire, préciser__________	Code _____	Code _______	Code _______	Code _____
**12** Diagnostics Imagerie générale	Code _____	Code _______	Code _______	Code _____
**13** Diagnostics Imagerie spécifique, préciser__________	Code _____	Code _______	Code _______	Code _____
**14** Diagnostics Autre, préciser_________	Code _____	Code _______	Code _______	Code _____
**15** Traitement Médication générale	Code _____	Code _______	Code _______	Code _____
**16** Traitement Médication spécifique, préciser_________	Code _____	Code _______	Code _______	Code _____
**17** Traitement Autre médication, préciser__________	Code _____	Code _______	Code _______	Code _____

For peer review only - http://bmjopen.bmj.com/site/about/guidelines.xhtml

**18** Traitement<br>Interventions (autres que chirurgie), préciser________	Code _____	Code _______	Code _______	Code _____
**19** Traitement<br>Chirurgie	Code _____	Code _______	Code _______	Code _____
**20** Hospitalisation<br><7 jours	Code _____	Code _______	Code _______	Code _____
**21** Hospitalisation<br>≥7 jours	Code _____	Code _______	Code _______	Code _____
**22** Services d'urgence	Code _____	Code _______	Code _______	Code _____
**23** Soins prénatals	Code _____	Code _______	Code _______	Code _____
**24** Soins postnatals	Code _____	Code _______	Code _______	Code _____
**25** Médecine préventive	Code _____	Code _______	Code _______	Code _____
**26** Vaccination	Code _____	Code _______	Code _______	Code _____
**27** Soins à domicile (personnes âgées, handicapés, malades chroniques/à long terme)	Code _____	Code _______	Code _______	Code _____
**28** Thérapie de soutien (physiothérapie, ergothérapie, réhabilitation)	Code _____	Code _______	Code _______	Code _____

VACCINATION

6 Comment les enfants de ce ménage ont-ils été nourris pendant les 6 premiers mois de leur vie ?

Instructions : Passer cette question s'il n'y a pas d'enfants de sexe masculin ou féminin dans ce ménage (voir également questions 2 et 3 de ce formulaire partie A).

O 1=Lait maternisé exclusivement
O 2=Lait maternisé et autres aliments, préciser ______
O 3=Allaitement et autres aliments, préciser _________
O 4=Lait maternisé et allaitement
O 5=Allaitement exclusivement
O 6=Autre, préciser _____
O 99=Ne sait pas
O 98=Pas de réponse

7 Quelle source d'approvisionnement en eau était-elle habituellement utilisée pour préparer le lait maternisé ou le lait maternisé et d'autres aliments comme indiqué à la question 6 pour les enfants de ce ménage ?

Instructions : Passer cette question s'il n'y a pas d'enfants dans ce ménage (voir également les questions 2 et 3 de ce formulaire Partie A).

O 1=Eau du robinet (à l'intérieur de l'habitation, privé)
O 2=Eau du robinet (à l'extérieur de l'habitation, arrière- cour/cour, privé)
O 3=Eau du robinet (à l'extérieur de l'habitation, public, partagé)
O 4=Borne-fontaine/Eau courante (à l'intérieur de l'habitation, privé)
O 5=Borne-fontaine/Eau courante (à l'extérieur de l'habitation, arrière- cour/cour, privé)
O 6=Borne-fontaine/Eau courante (à l'extérieur de l'habitation, public, partagé)
O 7=Camion-citerne (public, partagé)
O 8=Eau en bouteille
O 9=Eaux de surface (lac, étang, rivière, ruisseau)
O 10=Eaux de surface (canal, canal d'irrigation)
O 11=Eau de source
O 12=Eau de pluie
O 13=Puits/Puits de forage (ouvert/non couvert/non protégé)
O 14=Puits/Puits de forage (couvert/protégé)
O 15=NO FORMULA/OTHER FEEDS PREPARED
O 16=Autre, préciser _____________ O 99=Ne sait pas O 98=Pas de réponse

8 Est-ce que les enfants de ce ménage ont été vaccinés depuis leur naissance, y compris les vaccins de naissance ?

For peer review only - http://bmjopen.bmj.com/site/about/guidelines.xhtml

Instructions : Indiquer un code par catégorie et par enfant uniquement. Passer cette question s'il n'y a aucun enfant dans ce ménage (voir également les questions 2 et 3 de ce formulaire PartieA). Se référer à la question 9 « Formulaire 3 Partie A – Données démographiques/Informations générales sur le ménage » et indiquer les initiales/identificateur du/des membre(s).

Enfant 1<br>Initiales/Identifiant : ____	Enfant 2<br>Initiales/Identifiant : ____	Enfant 3<br>Initiales/Identifiant : ____	Enfant 4<br>Initiales/Identifiant : ____
[1] OUI, vacciné [5] Non, manque de vaccins [9] Non, EDS débordé<br>[2] Non, parent/tuteur débordé [6] Non, enfant trop jeune/vieux [10] Non, enfant malade<br>[3] Non, parent/tuteur a décidé que pas nécessaire [7] Non, parent/tuteur a refusé/trop inquiet [11] Non, pas de carte de vaccination<br>[4] Non, ne sait pas où aller [8] Non, Autre, préciser ____________ [99] Don't know [98] No response			
**8.1 Diphtérie et tétanos et coqueluche et *Haemophilus influenza* et vaccin contre l'hépatite B / DTwPHibHepB** (par exemple 6/8, 10/12, 14/16 semaines)			
Code __________	Code __________	Code __________	Code __________
**8.2 Anatoxine tétanique (TT)** (par exemple, 1er contact, grossesse, 1, 6, 12/24 mois)			
Code __________	Code __________	Code __________	Code __________
**8.3 Anatoxine tétanique et diphtérique (TD)** (par exemple, grossesse avec un premier contact, 1, 6, 12 mois)			
Code __________	Code __________	Code __________	Code __________
**8.4 Tuberculose / BCG** (à la naissance)			
Code __________	Code __________	Code __________	Code __________
**8.5 Rotavirus / RV** (par exemple 6/8, 10/12, 16 semaines)			
Code __________	Code __________	Code __________	Code __________
**8.6 Rougeole / rougeole et rubéole (MR)** (par exemple 9, 15/18 mois)			
Code __________	Code __________	Code __________	Code __________
**8.7 Polio / VPO** (par exemple naissance, 6/8, 10/12, 14/16 semaines)			
Code __________	Code __________	Code __________	Code __________
**8.8 Pneumocoque / PCV** (par exemple 6/8, 10/12, 14/16 semaines)			
Code __________	Code __________	Code __________	Code __________
**8.9 Fièvre jaune (YF)** (par exemple 9 mois)			
Code __________	Code __________	Code __________	Code __________
**8.10 Autres vaccins**			
Préciser, __________	Préciser, __________	Préciser, __________	Préciser, __________
Préciser, __________	Préciser, __________	Préciser, __________	Préciser, __________
Préciser, __________	Préciser, __________	Préciser, __________	Préciser, __________
Préciser, __________	Préciser, __________	Préciser, __________	Préciser, __________

For peer review only - http://bmjopen.bmj.com/site/about/guidelines.xhtml

PERCEPTION DE LA MALADIE

9 Connaissance, perception, causes et prévention des maladies infectieuses communes

Maladie	**9.1 Maladie connue des membres du ménage ?** [1] Oui [2] Non [99] Ne sait pas [98] Pas de réponse	**9.2 Perception de la maladie par les membres du ménage** *Instruction :* Décrire brièvement si elle est connue.	**9.3 Causes de la maladie connue des membres du ménage ?** *Instructions :* Indiquer tous les codes (liste A) qui s'appliquent; plus d'une réponse est possible	**9.4 Mesures pour prévenir la maladie connues des membres du ménage ?** *Instructions :* Indiquer tous les codes (liste B) qui s'appliquent ; plus d'une réponse est possible.	**9.5 Mesures pour prévenir la maladie utilisées par les membres du foyer ?** *Instructions :* Indiquer tous les codes (liste B) qui s'appliquent ; il peut y avoir plus d'une réponse.
**A.** Fièvre typhoïde	Code ___________		Code ___________	Code ___________	Code ___________
**B.** Choléra	Code ___________		Code ___________	Code ___________	Code ___________
**C.** Paludisme	Code ___________		Code ___________	Code ___________	Code ___________
**D.** Grippe	Code ___________		Code ___________	Code ___________	Code ___________
**E.** Méningite	Code ___________		Code ___________	Code ___________	Code ___________
**F.** Hépatite E	Code ___________		Code ___________	Code ___________	Code ___________
**G.** Tuberculose	Code ___________		Code ___________	Code ___________	Code ___________
**H.** VIH/SIDA	Code ___________		Code ___________	Code ___________	Code ___________

Liste A: Causes possibles	Liste B: Mesures préventives possibles
[1] Consommation d'alcool<br>[2] Temps/sorcellerie	[1] Pas de consommation d'alcool<br>[2] Dormir sous une moustiquaire

For peer review only - http://bmjopen.bmj.com/site/about/guidelines.xhtml

[3] Piqûre de moustique/d'insecte<br>[4] Morsure de chien<br>[5] Boire de l'eau non traitée de surface/de source (lac, étang, rivière, ruisseau, canal, canal d'irrigation)<br>[6] Contact physique étroit/baiser/embrassade<br>[7] Manger des produits alimentaires crus comme des légumes/de la salade, des fruits, des produits laitiers, des œufs, de la viande ou du poisson<br>[8] Ne pas se laver les mains correctement en préparant la nourriture/les repas<br>[9] Ne pas se laver les mains correctement avant/après être allé aux toilettes<br>[10] Contact avec du sang humain (injection, transfusion, chirurgie)<br>[11] Consommation de sang animal cru<br>[12] Autre, préciser ____________________<br>[99] Ne sait pas<br>[98] Pas de réponse	[3] Utiliser des sprays insecticides/anti-moustiques<br>[4] Maintenir la maison et ses environs immédiats propres<br>[5] Avoir une pratique correcte de l'élimination des déchets humains et domestiques<br>[6] Éviter le contact rapproché avec des chiens<br>[7] Traiter l'eau (faire bouillir, filtration, désinfection solaire, sédimentation, chloration)<br>[8] Ne pas cuisiner/préparer la nourriture/les repas avec de l'eau non traitée<br>[9] Ne pas laver les produits alimentaires à consommer crus avec de l'eau non traitée<br>[10] Faire bouillir/chauffer à nouveau les restes/les aliments/repas préparés avant de les consommer<br>[11] Ne pas manger des produits alimentaires crus comme légumes/salade, fruits, produits laitiers, œufs, viande ou poisson<br>[12] Couvrir les produits alimentaires/repas<br>[13] Protéger les produits alimentaires/repas contre les mouches<br>[14] Éviter la nourriture/les boissons des vendeurs de rue<br>[15] Éviter le contact rapproché avec les personnes malades<br>[16] Pas de contact physique étroit/baisers/embrassades<br>[17] Se couvrir le nez/la bouche en éternuant/toussant<br>[18] Pas de contact/consommation de sang humain/animal<br>[19] Avoir une bonne hygiène des mains en préparant la nourriture/les repas (se laver les mains avec du savon et de l'eau, se laver les mains par frottement)<br>[20] Avoir une bonne hygiène des mains avant/après être allé aux toilettes (se laver les mains avec du savon et de l'eau, se laver les mains par frottement)<br>[21] Autre, préciser ____________________<br>[99] Ne sait pas<br>[98] Pas de réponse

For peer review only

For peer review only - http://bmjopen.bmj.com/site/about/guidelines.xhtml

10 Pour quels membres de ce ménage les mesures préventives listées à la questions 9.5 contre la fièvre typhoïde sont-elles utilisées ?

Instructions : Cocher plusieurs cases si nécessaire ; il peut y avoir plus d'une réponse.

O 1=<2 ans	O 3=≥5 à <15 ans	O 5=PAS UTILISÉ	O 99=Ne sait pas
O 2=≥2 à < 5 ans	O 4=≥15 ans		O 98=Pas de réponse

11 Nommer les membres du ménage pour lesquels une fièvre typhoïde a été diagnostiquée dans les 3 derniers mois. Quels éléments de diagnostic ont été utilisés pour confirmer la fièvre typhoïde?

Instructions : Indiquer plusieurs codes si nécessaire pour chaque membre du ménage ; il peut y avoir plus d'une réponse.

Membre 1 Initiales/Identifiant: __	Membre 2 Initiales/Identifiant: __	Membre 3 Initiales/Identifiant: __	Membre 4 Initiales/Identifiant: __
[1] Hémoculture [3] Test sanguin/de Widal [5] Pas applicable (pas diagnostiqué avec la fièvre typhoïde)<br>[2] Coproculture [4] Autre test, préciser ____________ [99] Ne sait pas [98] Pas de réponse			
Code __________	Code __________	Code __________	Code __________

12 (Où les membres de ce ménage indiqués à la question 11 ont-ils été diagnostiqués pour la fièvre typhoïde?) Où les membres de ce ménage ont-ils recherché des soins de santé?

Instructions : Indiquer un code par membre du ménage uniquement.

Membre 1 Initiales/Identifiant: __	Membre 2 Initiales/Identifiant: __	Membre 3 Initiales/Identifiant: __	Membre 4 Initiales/Identifiant: __
[1] Établissement de santé, préciser ___________<br>[2] Médecin<br>[3] Nulle part, automédication<br>[4] Guérisseur traditionnel<br>[5] Pharmacien		[6] Nulle part, pas de recherche de soins<br>[7] Autre, préciser ___________<br>[8] Pas applicable<br>[99] Ne sait pas<br>[98] Pas de réponse	
Code __________	Code __________	Code __________	Code __________

13 (Les membres de ce ménage pour lesquels une fièvre typhoïde a été diagnostiquée comme indiqué à la question 11 ont-ils reçu un traitement?) Quel traitement ont-ils reçu?

Instructions : Cocher une réponse par membre du ménage uniquement.

Membre 1 Initiales/Identifiant: __	Membre 2 Initiales/Identifiant: __	Membre 3 Initiales/Identifiant: __	Membre 4 Initiales/Identifiant: __
O1=Traitement, préciser ______________<br>O 2=Pas applicable<br>O 99=Ne sait pas<br>O 98=Pas de réponse	O1=Traitement, préciser ______________<br>O 2=Pas applicable<br>O 99=Ne sait pas<br>O 98=Pas de réponse	O1=Traitement, préciser ______________<br>O 2=Pas applicable<br>O 99=Ne sait pas<br>O 98=Pas de réponse	O1=Traitement, préciser ______________<br>O 2=Pas applicable<br>O 99=Ne sait pas<br>O 98=Pas de réponse

For peer review only - http://bmjopen.bmj.com/site/about/guidelines.xhtml

Site ___ ___ ___ Sous-zone ___ ___ ___ Numéro d'habitation ___ ___ ___ ___ Identifiant de l'enquêteur ___ __

FORMULAIRE 5: ÉVALUATION DE L'UTILISATION RÉELLE DES SOINS DE SANTÉ GLOBAUX, STRATIFIÉE SELON L'ÂGE ET LE SEXE

Partie A: Évaluation de l'utilisation *réelle* des soins de santé

COMPORTEMENT DE RECHERCHE DE SOINS

1 Consigner ci-dessous l'apparition de signe(s) ou symptôme(s) (voir liste 1) et préciser où (voir liste 2) et comment le(s) membre(s) a/ont recherché les soins de santé durant les 3 derniers mois ?

Instructions : voir « formulaire 3- partie A-INFORMATIONS GENERALES SUR LE MENAGE » pour obtenir les initiaux/identifiants de chacun des membres du ménage. Indiquer seulement un signe/symptome par ligne. Commencer avec une nouvelle ligne pour tous nouveaux soins de santé sélectionnés pour chaque signe/symptome recurrent. Indiquer le nombre des apparitions de chaque signe/symptômes durant les 3 derniers mois. Si une différente option de soins de santé a été sélectionnée pour un signe/symptôme recurrent, indiquer cela dans une nouvelle ligne. Completer s'il vous plaît, un formulaire supplémentaire s'il y a eu plus de 20 signes/symptômes apparus durant les 3 derniers mois.

**Liste 1: Signe/Symptôme**		**Liste 2: Option de soins de santé**
[A] Fièvre	[N] Nausée/Vomissements	[1] EDS-SETA, Code _______________
[B] Fièvre <3 jours (continus)	[O] Diarrhée (liquide/sanglante/mucopurulente)	[2] Autre EDS Code _______________
[C] Fièvre ≥3 jours (continus)	[P] Douleur abdominale/Douleur intestinale	[3] Médecin
[D] Frissons/Tremblements	[Q] Perte de sang/Hémorragie (interne)	[4] Pharmacie
[E] Convulsion	[R] Perte de sang/Hémorragie (externe)	[5] Guérisseur traditionnel
[F] Perte de poids	[S] Éternuements Nez qui coule	[6] Nulle part Auto-médication
[G] Déshydratation	[T] Toux	[7] Nulle part Rien
[H] Malaise/Faiblees/Fatigue	[U] Respiration rapide/Essoufflement	
[I] Douleurs (muscles/articulations/os)	[V] Difficultés respiratoires graves	
[J] Maux de tête	[W] Inflammation de la peau (éruptions cutanées/ rougeurs/pus/démangeaisons)	
[K] Vertiges/Confusion/ Perte de connaissance	[X] Gonflement/Œdème	
[L] Troubles de la pression sanguine	[Y] Jaunisse	
[M] Troubles cardiaques		

**No.**	**Membre du ménage** Enregistrer les initiaux/identifiants du membre du ménage ; voir formulaire 3 – partie A- INFORMATIONS GENERALES SUR LE MENAGE »	**Signes/ Symptômes** Utiliser la Liste 1 pour sélectionner le code	**Options de soins de santé**		**Fréquence** Indiquer le nombre d'incidences pour le signe/symptôme listé
			Utiliser la Liste 2 pour sélectionner le code	EDS Code	
1					
2					
3					
4					
5					

For peer review only - http://bmjopen.bmj.com/site/about/guidelines.xhtml

6				
7				
8				
9				
10				
11				
12				
13				
14				
15				
16				
17				
18				
19				
20				

For peer review only

For peer review only - http://bmjopen.bmj.com/site/about/guidelines.xhtml

Appendix 5/Supplementary file 7: System diagram of the data collection and data management platform: the Android application "HPA Collect" and the platform "HPA Web"

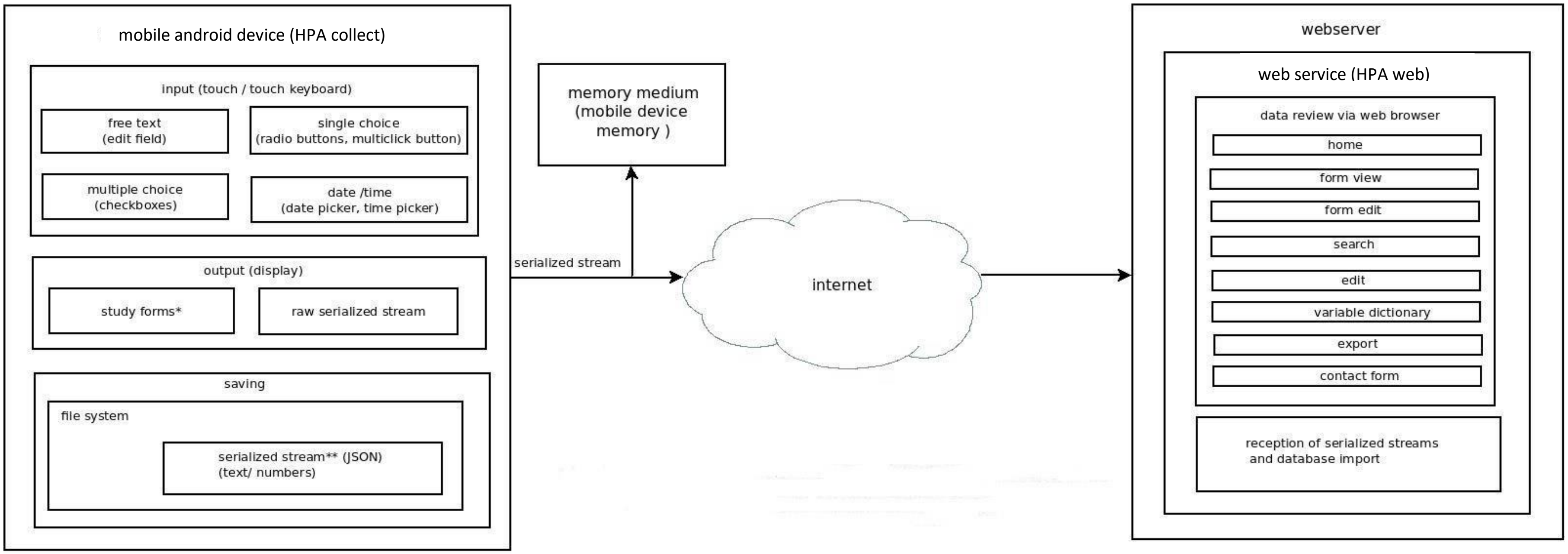

Note. *study forms: Form 1, Form 3 Part A to C, Form 4 Part A to B, Form 5; **serialized stream: two- or more-dimensional data (e.g. a table) is brought to a one-dimensional form; the application uses the JSON (Java Script Object Notation) format.

BMJ Open

The HPAfrica Study Protocol: Assessment of health behavior and population-based socio-economic, hygiene behavioral factors - a standardized repeated cross-sectional study in multiple cohorts in sub-Saharan Africa

Journal:	*BMJ Open*
Manuscript ID	bmjopen-2017-021438.R1
Article Type:	Protocol
Date Submitted by the Author:	06-Apr-2018

Complete List of Authors:	Pak, GiDeok; International Vaccine Institute, Biostatistics & Data Management<br>Haselbeck, Andrea; International Vaccine Institute, Epidemiology<br>Seo, Hyeong-Won; International Vaccine Institute, Biostatistics & Data Management<br>Osei, Isaac; Kumasi Centre for Collaborative Research in Tropical Medicine, Kwame Nkrumah University of Science and Technology (KNUST)<br>Amuasi, John; Kumasi Centre for Collaborative Research in Tropical Medicine, Kwame Nkrumah University of Science and Technology (KNUST)<br>Breiman, Robert; Emory University , Global Health Institute<br>Cruz Espinosa, Ligia Maria; International Vaccine Institute, Epidemiology<br>Im, Justin; International Vaccine Institute, Epidemiology<br>Jang, Geun-Hyeog; International Vaccine Institute, Biostatistics & Data Management<br>Jeon, Hyonjin; International Vaccine Institute, Epidemiology<br>Luby, Stephen; Stanford University Division of Infectious Diseases and Geographic Medicine<br>Lunguya-Metila, Octavie; Institut National de Recherche Biomedicales; Service de Microbiologie, Cliniques Universitaires de Kinshasa<br>MacWright, William; Global Health Institute, Emory University<br>Mogeni, Ondari; International Vaccine Institute, Epidemiology<br>Okeke, Iruka; University of Ibadan<br>Owusu-Dabo, Ellis; Kwame Nkrumah Univeristy of Science and Technology, School of Public Health<br>Park, Jin Kyung; International Vaccine Institute, Biostatistics & Data Management<br>Park, SeEun; International Vaccine Institute, Epidemiology<br>Popoola, Femi; University of Ibadan<br>Seo, Hye-jin; International Vaccine Institute, Epidemiology<br>Soura, Abdramane ; Institut Superieur des Sciences de la Population, University of Ouagadougou<br>Teferi, Mekonnen; Armauer Hansen Research Institute, ALERT Campus<br>Toy, Trevor; International Vaccine Institute, Epidemiology<br>Chon, Yun; International Vaccine Institute, Biostatistics & Data Management<br>Rahaniminina, Lydia; Universite d'Antananarivo

	Rakotozandrindrainy, Raphael; Universite d'Antananarivo<br>Meyer, Christian; Institute of Tropical Medicine, Eberhard Karls University; Faculty of Medicine, Duy Tan University<br>Marks, Florian ; International Vaccine Institute, Epidemiology; The Department of Medicine, The University of Cambridge<br>Panzner, Ursula; International Vaccine Institute, Epidemiology

**Primary Subject Heading**:	Epidemiology
Secondary Subject Heading:	Epidemiology, Global health, Health services research, Infectious diseases, Public health
Keywords:	health/hygiene behavior, sanitation, socio-economic, geospatial random selection, sub-Saharan Africa, HPAfrica study

SCHOLARONE™
Manuscripts

The HPAfrica Study Protocol: Assessment of health behavior and population-based socio-economic, hygiene behavioral factors - a standardized repeated cross-sectional study in multiple cohorts in sub-Saharan Africa

Short Title

The **H**ealth **P**opulation **Africa** (HPAfrica) study

Authors

Gi-Deok Pak[1#], Andrea Haselbeck[1#], Hyeong-Won Seo[1], Isaac Osei[2], John Amuasi[2], Robert Breiman[3], Ligia Maria Cruz Espinoza[1], Justin Im[1], Geun-Hyeog Jang[1], Hyon-Jin Jeon[1], Stephen Luby[4], Octavie Lunguya-Metila[5,6], William MacWright[3], Ondari D. Mogeni[1], Iruka N. Okeke[7], Ellis Owusu-Dabo[8], Jin-Kyung Park[1], Se Eun Park[1], Oluwafemi Popoola[7], Hye-Jin Seo[1], Abdramane Bassiahi Soura[9], Mekonnen Teferi[10], Trevor Toy[1], Yun Chon[1], Lydia Beithine Rahaniminina[11], Raphael Rakotozandrindrainy[11], Christian G. Meyer[12,13], Florian Marks[1,14*] and Ursula Panzner[1]

Affiliations

1. International Vaccine Institute, Seoul, Republic of Korea
2. Kumasi Centre for Collaborative Research in Tropical Medicine, Kwame Nkrumah University of Science and Technology (KNUST), Kumasi, Ghana
3. Global Health Institute, Emory University, Atlanta, United States
4. Infectious Diseases and Geographic Medicine, Stanford University, Stanford, United States
5. Institut National de Recherche Biom´edicales, Kinshasa, Democratic Republic of Congo
6. Service de Microbiologie, Cliniques Universitaires de Kinshasa, Kinshasa, Democratic Republic of the Congo
7. University of Ibadan, Ibadan, Nigeria
8. School of Public Health, Kwame Nkrumah Univeristy of Science and Technology, Kumasi, Ghana
9. Institut Supérieur des Sciences de la Population, University of Ouagadougou, Ouagadougou, Burkina Faso
10. Armauer Hansen Research Institute, Jimma Road, ALERT Campus, Addis Ababa, Ethiopia
11. University of Antananarivo, Antananarivo, Madagascar
12. Institute of Tropical Medicine, Eberhard Karls University, Tübingen, Germany
13. Faculty of Medicine, Duy Tan University, Da Nang, Vietnam
14. The Department of Medicine, The University of Cambridge, Cambridge, United Kingdom

# Contributed equally

*Corresponding Author:

Florian Marks, MPH, PhD, International Vaccine Institute, SNU Research Park, 1 Gwanak-ro, Gwanak-gu, Seoul, 08826, Republic of Korea, Phone: +82-2-881-1133, Fax: +82-2-881-1164, E-mail: fmarks@ivi.int

For peer review only - http://bmjopen.bmj.com/site/about/guidelines.xhtml

Abstract

Introduction

The objective of the **H**ealth **P**opulation **Africa** (HPAfrica) study is to determine health behavior and other population-based factors, including socio-economic, ethnographic, hygiene and sanitation factors, at sites of the Severe Typhoid Fever in Africa (SETA) program. SETA aims to investigate healthcare facility-based fever surveillance in Burkina Faso, the Democratic Republic of the Congo (DRC), Ethiopia, Ghana, Madagascar, and Nigeria. Meaningful disease burden estimates require adjustment for health behavior patterns, which are assumed to vary among a study population.

Methods and Analysis

For the minimum sample size required, the assumptions of an infinite population, a design effect, and age- and sex-stratification are considered. In the absence of a sampling frame or household list, a geospatial approach will be used to generate geographic random points with an Aeronautical Reconnaissance Coverage Geographic Information System (ArcGIS) tool. Printouts of Google Earth Pro® satellite imagery visualize these points. Data of interest will be assessed in different seasons by applying simple, population weighted-stratified sampling. An Android-based application and a web service have been developed for electronic data capturing and synchronization with the database server in real time. Descriptive data analyses will be performed in order to assess baseline information of each study population and age- and sex-stratified health behavior. This will allow adjusting disease burden estimates. In addition, multivariate analyses will be applied to look into associations between health behavior, population-based factors, and the disease burden as determined in the SETA study.

For peer review only - http://bmjopen.bmj.com/site/about/guidelines.xhtml

Strengths and limitations of this study

- Standardization in community-based multi-country/-site research requires cautiousness considering variations in setting, language and culture.
- Large-scale public health assessments will complement disease burden data by investigating sources of transmission and infection.
- Current population data in study sites and their administratively-defined subareas are assumed to be limited.
- Electronic data collection may cause technical problems related to damage, functional failure, or loss of devices.
- Unstable wireless internet connection may limit data teams to update the Android-based application, communicate with study managers in real time, and synchronize collected data with the server.

Registration: Not required

Keywords: health/hygiene behavior, sanitation, socio-economic, geospatial random selection, sub-Saharan Africa, HPAfrica study

For peer review only
For peer review only - http://bmjopen.bmj.com/site/about/guidelines.xhtml

Introduction

Assessment of health behavior, including healthcare utilization, is an important determinant for the generation of accurate disease burden estimates among target populations. This is particularly critical for studies which utilize observational, healthcare facility-based surveillance designs as substantial variation in health behavior patterns strongly influences disease burden estimates[1 2]. The adjustment of disease burden calculations for variation in health behavior will increase the accuracy of assessing prevalences of diseases caused by bacterial, fungal, viral, and parasitic pathogens among populations under investigation. Furthermore, the influence of a large variety of population-based factors on both health behavior and disease burden has been recognized. This includes socioeconomic factors such as education, occupation, income, the availability of household assets[3 4], ethnical/religious and cultural factors[5 6], but also hygiene and sanitation facilities[7-11], food handling[12-14], and animal contacts[10].

The **H**ealth **P**opulation **Africa** (HPAfrica) study will be conducted in Burkina Faso, Democratic Republic of the Congo (DRC), Ethiopia, Ghana, Madagascar, and Nigeria. Experiences from the Typhoid Fever Surveillance in Africa Program (TSAP) will be transferred to the Severe Typhoid in Africa (SETA) program[15 16]. In this program, standardized, healthcare facility-based fever surveillance is performed at selected study sites in participating countries over a two-years period[17]. SETA primarily aims to systematically collect information on the burden of severe *Salmonella* infections. Subjects living in defined catchment areas with either an acute fever episode, a fever history of ≥3 consecutive days, clinically-suspected typhoid fever or clinically-diagnosed gastrointestinal perforations due to typhoid fever will be eligible. In addition, the severity of *Salmonella* caused illnesses and the natural history of *Salmonella* infection will be recorded with further analyses on host immune responses and chronic carriage.

Objectives and Outcomes

The goal of the HPAfrica study is to assess the *generic* and *actual* age- and sex-stratified health behavior. *Generic* health behavior pertains to behavior in conditions associated with fever and other

For peer review only - http://bmjopen.bmj.com/site/about/guidelines.xhtml

signs and symptoms unrelated to onsets, whereas *actual* health behavior is related to onsets (Appendix 1/Supplementary file 1; Appendix 2/Supplementary file 2: Form 4 Part A, Form 5; Appendix 3/Supplementary file 3). These data will be used to adjust age- and sex-stratified incidences of infectious pathogens in the proportion of a study population not captured by activities of HPAfrica. Data documented for various signs and symptoms or a combination of these will be used for a gradual classification of healthcare utilization based on disease severity.

The HPAfrica study will, moreover, collect data on healthcare-associated factors such as travel modalities, possession of a health insurance, cultural/ethnographic factors, immunization status among children[18 19], and the frequency of occurrence and individual perception of selected diseases (Appendix 2/Supplementary file 2: Form 4 Part B; Appendix 3/Supplementary file 3:Form 4 Part B). Demographic data (Appendix 2/ Supplementary file 2: Form 3 Part A; Appendix 3/Supplementary file 3, Form 3 Part A) will be used for age- and sex-stratification of the population surveyed, which is required to compute adjustment factors for disease incidences. Socio-economic data (Appendix 2/ Supplementary file 2: Form 3 Part B; Appendix 3/ Supplementary file 3: Form 3 Part B) will be utilized to calculate wealth indices for descriptive and analytical approaches of the populations studied. Information on hygiene and sanitation facilities (Appendix 2/ Supplementary file 2: Form 3 Part C; Appendix 3/Supplementary file 3: Form 3 Part C) may provide a better understanding of the occurrence and frequency of pathogens identified in the populations studied.

Methods

Study sites

The boundaries of each site will be defined using pre-existing information set by statistical authorities or ministries of health (Table 1). A retrospective review of records of recruitment healthcare facilities from the past two years will support the re-definement of site limits. It is expected that this review will reveal the residences or at least the broader administrative residential areas such as communities, districts or villages of patients who sought healthcare for any reason. Maps and satellite imagery may

For peer review only

133 be used to better visualize patients' residences and the overall boundaries, including the
134 geographically or administratively defined subareas, of each site. Where boundaries cannot be clearly
135 defined, additional factors such as the distance to a recruitment healthcare facility may be considered.

For peer review only

For peer review only - http://bmjopen.bmj.com/site/about/guidelines.xhtml

Table 1: Background data of participating study sites of target countries

Country	Study site	Setting*	Approximate total site population (year)	Approximate age-stratified site population				Approximate sex-stratified site population	
				<2y	≥2y to <5y	≥5y to <15y	≥15y	Male	Female
Burkina Faso	Nioko II[1]	Urban	19,251 (2017)	2,394	1,977	4,657	10,223	9,321	9,930
	Polesgo[1]	Rural	7,897 (2017)	934	893	1,808	4,262	3,856	4,041
	Ouagadougou[1]	Urban	2,532,311 (2015)	421,429		693,723	1,417,159	1,271,302	1,261,009
DRC	Kisantu[2]	Urban	291,252 (2017)	48,043		243,209		91,598[#]	99,231[#]
Ethiopia	Wolayita Zone[3]	Urban/ Rural	1,968,735 (2017)	100,800	206,520	635,015	1,026300	964,577	1,004,058
	Wolayita Sodo[3]	Urban/ Rural	120,288 (2017)	6,161	12,617	38,807	62,703	59,898	60,390
	East Shewa & Arsi Zone[3]	Urban/ Rural	3,249,722 (2017)	104,408	422,939	1,016,917	1,705,458	1,671,699	1,578,023
	Adama Wenji[3]	Urban/ Rural	53,540 (2017)	3,330	7,204	17,028	25,978	27,199	26,341
Ghana	Asante Akim North & Central[4]	Urban/ Rural	140,694 (2010)	11,606	8,363	35,618	85,107	67,673	73,021
	Kumasi (Metropolis)[4,5]	Urban	1,730,249 (2010)	52,516	178,575	421,834	1,077,324	826,479	903,770
Madagascar	Antananarivo[6] (Renivohitra)	Urban	1,247,025 (2009)	N/A[$]	N/A[$]	N/A[$]	N/A[$]	N/A[$]	N/A[$]
	Imerintsiatosika[7]	Rural	44,669 (2016)	3,582	4,449	7,610	29,028	N/A[$]	N/A[$]
Nigeria	Ibadan[8]	Urban	1,343,147 (2006)	176,110		305,656	861,381	661,818	681,329

Note. Table 1 shows population data that were available at the time of the HPAfrica protocol writing; population data and boundaries of geographically and/or administratively defined study sites may be subject to changes during the course of the study. *The classification of sites by country is based on best local knowledge; #The sex-stratification is based on a total population of 190,829; $Population data not available at the time of protocol writing; y= years of age.

Source of population data: **Burkina Faso** ([1]http://www.insd.bf/n/; [2]http://www.indepth-network.org/member-centres/ouagadougou-hdss); **DRC** ([2]Kisantu Central Health Zone Office report, 2016); **Ethiopia** ([3]Health Management Information System of the Ethiopian Ministry of Health (zonal and district health offices); **Ghana** ([4]Ghana Statistical Service, 2010 Population & Housing Census, Asante Akim Central Municipality; [5]Ghana Statistical Service, 2010 Population & Housing Census, Summary report of final results); **Madagascar** ([6]Population par Fokotany selon la declaration des Chefs Fokotany: Source: Donnee de la cartographie censitaire mises-a-jour en juillet 2009 - INSTAT/DDSS; [7]University of Antananarivo); **Nigeria** ([8]Federal Republic of Nigeria 2006 Population and Housing Census (Table DS5), National Population Commission, Abuja, Nigeria).

For peer review only

For peer review only - http://bmjopen.bmj.com/site/about/guidelines.xhtml

Sample size

All available sources will be used to gather most up-to-date age- and sex-stratified population data by study site and its administratively or geographically defined subareas. This may include latest demographic information from a population census or a Demographic Surveillance System (DSS)/Health and Demographic Surveillance System (HDSS). Population summary figures and population growth factors, if available, may also be used.

The minimum number of randomly selected households to be interviewed will be calculated using the precision-based equation (Equation-I)[20-23] assuming an infinite population and considering a design effect (DEFF), including its accompanying assumptions:

Equation-I: precision-based sample size calculation for an infinite population[20-23]:

$$n_0 = DEFF \cdot \left[z^2{}_{1-\alpha/2} \cdot \frac{4 \cdot p \cdot (1-p)}{d^2} \right]$$

<u>Assumptions Equation-I</u>

n_0	Minimum number of households to be interviewed in a study area assuming an infinite population
DEFF	Design effect; set at 1.5
$z_{1-\alpha/2}$	Normal deviation corresponding to a 95% confidence interval (1.96 for alpha of 0.05)
D	Precision (acceptable error); point estimation; set at 0.2
P	Proportion of the study population expected to visit a recruitment healthcare facility for conditions associated with fever and other signs and symptoms (proportion captured); set at 0.2
1-p	Proportion of the study population expected to not visit a recruitment healthcare facility for conditions associated with fever and other signs and symptoms (proportion not captured)

The design effect is an adjustment factor for natural clustering of health behavior among household members[21]. It is arbitrarily set at 1.5, depending on the household size, as no exact estimates are available[24 25]. A proportion p of 0.2 may be assumed if no other estimates are available or p may be based on experiences during the TSAP study: Madagascar, Isotry: p=0.01, Burkina Faso, Polesgo: p=0.9[26]. Combining all assumptions into Equation-I will result in a total minimum number (n_0) of 92 household interviews at each site.

In addition, n_0 will be accounted for the age- and sex-distribution of a study population reflected by $\hat{p}$,

For peer review only

For peer review only - http://bmjopen.bmj.com/site/about/guidelines.xhtml

the assumed minimum proportion of stratification per age group (<5 years, ≥5 to <15 years, ≥15 years). Factor $\hat{p}$ may be arbitrarily set at 0.2 or may be based on previous experiences: Ethiopia, Butajira: $\hat{p}$=0.09, Burkina Faso, Polesgo: $\hat{p}$=0.23[26]. Applying $\hat{p}$ to Equation-I for a binomial distribution, including its accompanying assumptions, will result in the age-stratified minimum number of household interviews ($\overline{n_0}$) of 461 (Equation-II):

Equation-II: precision-based sample size calculation for an infinite population considering age-stratification:

$$\overline{n_0} = \left(DEFF \cdot \left[z^2_{1-\frac{\alpha}{2}} \cdot \frac{4 \cdot p \cdot (1-p)}{d^2}\right]\right)/\hat{p}$$

Assumptions Equation-II

$\overline{n_0}$	Minimum number of households to be interviewed in the study area assuming an infinite population and age-stratification
DEFF	Design effect; set at 1.5
$z_{1-\alpha/2}$	Normal deviation corresponding to a 95% confidence interval (1.96 for alpha of 0.05)
D	Precision (acceptable error); point estimation; set at 0.2
P	Proportion of the study population expected to visit a recruitment healthcare facility for conditions associated with fever and other signs and symptoms (proportion captured); set at 0.2
1-p	Proportion of the study population expected to not visit a recruitment healthcare facility for conditions associated with fever and other signs and symptoms (proportion not captured)
$\hat{p}$	The minimum proportion of stratification per age group; set at 0.2

Health behavior will be assessed twice at the same households if possible or at alternative households in case the study population is instable due to migration and/or high birth/death rates. Additional households, assumed to be 20% on average, (Table 2) may be added to account for possible loss to follow-up between the two assessments. Eventually, $\overline{n_0}$ of 553 will be divided by the number of subareas and weighted proportionally to the population size by applying weighted-stratified sampling[27].

Table 2: Sample sizes considering infinite population and age-stratification applying Equation-II:

Proportion (p)	Total minimum number of households without DEFF	Total minimum number of households (n_0) with DEFF=1.5	Total minimum number of households ($\overline{n_0}$) with DEFF=1.5 and $\hat{p}$=0.2	$\overline{n_0}$ adjusted for loss to follow-up
0.1	35	52	259	311
0.2	62	92	461	553
0.3	81	121	605	726
0.4	92	138	691	830

For peer review only

For peer review only - http://bmjopen.bmj.com/site/about/guidelines.xhtml

0.5	96	144	720	864
0.6	92	138	691	830
0.7	81	121	605	726
0.8	62	92	461	553
0.9	35	52	259	311

Abbreviations
p=Proportion of the study population expected to visit a recruitment healthcare facility for conditions associated with fever and other signs and symptoms (proportion captured); set at 0.2
DEFF=Design effect; set at 1.5
n_0=Total minimum number of households to be interviewed in a study area assuming an infinite population
$\overline{n}_0$=Total minimum number of households to be interviewed in the study area assuming an infinite population and age-stratification
$\hat{p}$=Minimum proportion of stratification per age group; set at 0.2

Sampling

If comprehensive up-to-date sampling frames or household lists exist (through DSS/HDSS or available census), computerized random selection will be done using SAS (Statistical Analysis System, version 9.4, SAS Institute, Cary, NC) applying serial simple random selection without replacement. If no sampling frame exists, it can be constructed using satellite imagery or aerial maps in order to enumerate all structures[26], rastering[28], or grid cells[29 30].

However, we apply a geospatial technique for the creation of points generated by the ArcGIS (Aeronautical Reconnaissance Coverage Geographic Information System; Redlands, CA, USA, version 10.2) random point generation tool with the application of simple, population weighted-stratified sampling[27 31]. The tool randomly places specified numbers of points inside the features of a polygon that corresponds to an administrative subarea. X- and Y-coordinates define one point until the calculated sample size is reached. ArcGIS-generated random points are converted into an image file of the Keyhole Markup Language (KML) or Keyhole Markup language Zipped (KMZ) format and imported into Google Earth Pro® (Google Inc., Mountain View, CA, USA, version 6.2.2.6613). A unique numerical identifier and horizontal geographic coordinates are assigned to each point. The location of each point is verified on an up-to-date Google Earth Pro® satellite imagery. Appendix 4/Supplementary file 4 illustrates our approach when utilizing examples from Ghana and Madagascar.

For peer review only

For peer review only - http://bmjopen.bmj.com/site/about/guidelines.xhtml

Geographic points labeled with identifiers are pictured on poster-size (60x60 to 60x90 cm) printouts of Google Earth Pro® satellite imagery with high resolution up to the structure level. The satellite maps help to locate selected points on the ground. Alternatively to printouts, an open source application for offline/online automated navigation and mapping may be used. Global Positioning System (GPS) receivers (Garmin-eTrex; Garmin Ltd., Lenexa, KS, USA) allow verifying the locations. The receivers will be positioned closely to a located point, in a static position and an open area to assure barrier-free reading. In case of refusal, absence of the respondent or locating of a non-residential point, a residential point closest to the right or left side will be visited instead[32].

Frequency of data collection

Residential points will be visited twice for household interviews during the SETA program to assess seasonal influences on health behavior[33-37]. Interviews will take place in differing seasons – one at the end of the dry season or at least one month after its beginning and one towards at end of the wet season or at least one month after its beginning (Table 3). Currently, the HPAfrica study is ongoing in Burkina Faso, Ghana and Madagascar and is anticipated to be continued in the remaining countries during the upcoming months.

Table 3: Seasonality in participating countries

Country	Period of Wet Season	Period of Dry Season
Burkina Faso	May/June - September/October	October/November - April/May
DRC	November - March	April - October
Ethiopia	June - August	October - May
Ghana	April – July, September - November	December - March
Madagascar	November - March/April	April/May - October
Nigeria	May - August	October - April

Note. The seasonality by country is based on best local knowledge.

Inclusion and exclusion criteria

Household members of all ages and both sexes living in a study area at the day of the interview will be eligible for inclusion. A household will be excluded if the designated respondent declines participation or is unavailable after three consecutive visits. All visitors and individuals with unknown

For peer review only - http://bmjopen.bmj.com/site/about/guidelines.xhtml

residence or residence outside the study area will be excluded.

For the purpose of this investigation, a household is defined as a person or a group of related or unrelated persons living in the same dwelling unit, acknowledging one adult individual as the household head, sharing the same housekeeping arrangements, and independently procuring food and other essential for living[26].

The interview will exclusively be held with the respondent, who is an adult household member at the country-specific legal age of majority. This person may be identified as decision-maker by members of the same household and serves as a proxy for an entire household[15]. Further relevant definitions are explained in Appendix 1/Supplementary file 1 and Appendix 3/Supplementary file 3.

Data collection

On-site interviewers will be trained on locating the geographic points, identification of respondents, informed consent procedures with emphasis on voluntary participation, and the deployment of standardized, pre-tested study forms (Appendix 2/Supplementary file 2 and Appendix 3/Supplementary file 3) prior to the initiation of HPAfrica. Informed consent and study forms will be translated into the country-specific official language and back-translated into English to account for translation errors and to make corrections, where required.

Data management

Management of data will depend on the mode of collection, which will be primarily electronic rather than paper-based. Past experiences showed following disadvantages of paper-based data collection: a high probability of errors while filling study forms, and the need of subsequent data digitalization, which is an extra, labor- and time-intensive and error-prone step. Electronic data capturing using a smartphone or tablet application minimizes these limiting factors to the data quality.

For peer review only - http://bmjopen.bmj.com/site/about/guidelines.xhtml

There may be a pilot period during which both paper-based and paperless data will be collected prior to full implementation of electronic data collection. The latter utilizes the Android application "HPA Collect" (Google Android 5.0.1 9API 23; available at https://play.google.com/store/apps/details?id=anint.ivi.hpa) and the platform "HPA Web" (CSS, JavaScript and JSP; available at http://hpa.ivi.int/), which are both accompanied by a variable dictionary containing codes, meaning and properties of variables in at least English and French. Stable wireless internet connectivity using default browsers is required to assure a contemporaneous, attributable, original and accurate synchronization of data between "HPA Collect" and "HPA Web". Access to both "HPA Collect" and "HPA Web" is password-protected. Appendix 5/Supplementary file 5 displays the system diagram, which has the following features:

Structure or features of "HPA Collect"

- **User management:** A simple user interface provides different privileges for users to enter, edit (except for study labels), and export or transfer data. It establishes an audit trail that records when users enter data into the server.
- **Data input:** Data are entered directly into the smartphone or tablet device. Entry fields limit possible answers to plausible ones only by offering different input methods:
  - Edit Text: free text
  - Radio group/Multi click button: single choice
  - Check box: multiple choice
  - Date and time picker: entering date and time
  - Spinner: drop-down button

  Additional features allow the surveyor to save time by transferring data collected at an earlier stage to data fields required at a later stage of the interview by performing plausibility checks for diverse data input fields, and by easily recognizing skip patterns. Error checks, missing data notifications, code errors, and logical errors are also included.

For peer review only - http://bmjopen.bmj.com/site/about/guidelines.xhtml

- **Data view:** Raw, originally entered data can be seen in the same, human- and machine-readable format, namely the JSON format, as they will be transferred to a database server (Windows, MySQL). Additional functions like "clear" to clear all records, "reset" to return back to the main data entry page, and "search" to search for a study label are available.
- **Settings:** The ability to switch the system language between English and French is provided.
- **About:** The currently installed version of "HPA Collect" is displayed.

Structure or features of "HPA Web"

- **Home:** An introduction to the platform is given and a tutorial in French and English will be accessible.
- **Form View:** The study forms are displayed separately showing every question and the corresponding input data as defined variables.
- **Form Edit:** This feature allows the correction of the uploaded data. It is only accessible to data managers in each participating country and at IVI.
- **Search:** Specific study labels given to each study form can be filtered.
- **Variable dictionary:** The variable dictionary can be downloaded.
- **Export:** Data can be converted from the server into a transferrable excel file.
- **Contact Us:** Quick and easy correspondence between users and the data manager at IVI is given.

Data will be reviewed by key study personnel on a day-to-day basis and checked for consistency and accuracy prior to data analysis. The quality of data may be checked by selecting a subset of 5% for validation against the original, paper-based data if possible. Names of household members will not be linked to study forms of HPAfrica and will not be recorded in the database in order to ensure confidentiality. Access to the database will be restricted to authorized study personnel only and data

For peer review only - http://bmjopen.bmj.com/site/about/guidelines.xhtml

will be kept in a locked, protected location. Periodic interim backup files and at least three final backup files of the database stored in a secure, locked place will avoid loss of data and ensure data safety. All data will be stored for a minimum of five years.

Data analysis

Descriptive analyses such as absolute and relative frequencies, medians or arithmetic means will be conducted to construct a contemporary baseline population description.

Basic analyses will include the calculation of age- and sex-stratified proportions of *generic* and *actual* health behavior, including healthcare utilization, for various signs and symptoms or combinations of signs and symptoms. These analyses will allow for a gradual classification based on disease severity, including 95% confidence interval. Health behavior will be adjusted for actual clustering among household members. Most up-to-date stratified population data by site and demographic data derived from HPAfrica will be used to generate the proportions for the adjustment of incidence calculations.

Socio-economic information collected will be used to construct wealth indices of studied populations by applying factor analyses based on the principal component method. In addition, bi- and multivariate regression analyses will be performed to identify potential confounding factors or effect modification using factor scores.

Bi- and multivariate regression analyses will be carried out using factor scores for the assessment of the following variables:

- Socioeconomic data such as education, occupation[38], health insurance, housing, and household assets
- Hygiene and sanitation such as toilet access, water sources, food handling, waste disposal, and animal contacts
- Health behavior: *actual* (age- and sex-stratified, severity, frequency), *generic* (age- and

For peer review only - http://bmjopen.bmj.com/site/about/guidelines.xhtml

sex-stratified, severity, rating by choice)

- Travel modalities to health facilities, age-stratified
- Seasonality related to hygiene & sanitation, health behavior, travel modalities
- Cultural/religious/ethnographic factors, age-stratified
- Vaccination status among children[18 19]
- Knowledge and perception of common diseases

Ethical considerations

All study participants are required to provide written informed consent. There are no direct benefits for enrolled households and their members. Indirect benefits for the general population at sites will be the updated information on the burden of communicable diseases, including factors influencing disease transmission and infection. The HPAfrica study generates evidence to support decision-makers on the introduction of appropriate measures for disease prevention and control.

This protocol was first approved by the Institutional Review Board of the International Vaccine Institute in October 2016 and undergoes annual review and renewal also by Ethics Committees of collaborating institutions (Ethics References are listed in Appendix1/Supplementary file1).

During and after the HPAfrica study, all data of enrolled subjects will be kept in strict confidence and will not be disclosed to a third party by any member of the research team. Password protection of devices and the database is used for strict confidence. All paper-based data (consent forms) will be kept in a secure, locked location. Confidential information stored on computers and paper-based data sources will only be made available to co-investigators and IVI staff directly involved in study activities of HPAfrica.

Patient and Public Involvement

No patients but household members in selected study sites will be involved. They did not participate

For peer review only - http://bmjopen.bmj.com/site/about/guidelines.xhtml

in the development of the research question, outcome measures and the study design. However, we attempt to balance well between answering our study objectives and benefiting the general population at the sites by disseminating information on the disease burden and factors influencing disease transmission and infection derived from our study. Household members will contribute to the study conduct by direct participating in the gathering of data. They will not be involved in recruitment procedures. The households selected for participation will be identified purely by chance due to the random sampling applied. Study findings will be disseminated to study populations directly through collaborating institutions and indirectly through publications in peer-reviewed journals.

Dissemination

We plan to disseminate the study findings by reporting descriptive as well as analytic and stratified data through publications in peer-reviewed journals and collaborating institutions.

Authors' contributions

GDP, JKP and UP conceptualized the initial study concept of the HPAfrica study. HWS and GHJ developed the tools "HPA Collect" and "HPA Web" for electronic data collection with primary support of UP and AH. IO and JA supported the development and finalizing of the data collection tools "HPA Collect" and "HPA Web". LBR and RR supported the development and finalization of the HPAfrica study forms. The appendices were conceptualized by GDP, JKP, YC, JI, TT, AH and UP. IO, JA, RB, LMCE, JI, HJJ, SL, OLM, WMW, ODM, INO, EOD, SP, OP, HJS, ABS, MT, TT, LBR and RR supported the further development of the initial study protocol and appendices. FM, YC and CGM participated in the finalization of the study protocol, including appendices. AH wrote the first draft of the manuscript. GDP, HWS, IO, JA, RB, LMCE, JI, GHJ, HJJ, SL, OLM, WMW, ODM, INO, EOD, JKP, SP, OP, HJS, ABS, MT, TT, YC, LBR, RR, CGM and FM critically reviewed and revised the manuscript draft. GDP, AH, HWS, IO, JA, RB, LMCE, JI, GHJ, HJJ, SL, OLM, WMW, ODM, INO, EOD, JKP, SP, OP, HJS, ABS, MT, TT, YC, LBR, RR, CGM, FM and UP agreed and approved the final manuscript as submitted.

For peer review only - http://bmjopen.bmj.com/site/about/guidelines.xhtml

Acknowledgement

We thank David Kauderer for the support and advice in computer engineering, Dr. Paul M. Tshiminyi for the support in proofreading French study materials, and Soo Young Kwon and Ji Hyun Han for administrative support. We are grateful to community leaders and site representatives for their assistance and facilitation in properly approaching household members for our study.

Funding statement

This study was supported by the Bill & Melinda Gates Foundation (OPP1127988). The funders had no role in study design, data collection, data analysis, data interpretation, or writing of the report; the conclusions and findings presented are those of the authors and do not necessarily reflect positions or policies of the Bill & Melinda Gates Foundation or the International Vaccine Institute (IVI). The IVI acknowledges its donors, including the Republic of Korea and the Swedish International Development Cooperation Agency (Sida).

Competing interests

The authors declare that they have no competing interests.

Abbreviations

JSON: JavaScript Objective Notation; CSS: Cascading Style Sheets; JSP: Java Server Page; SQL: Structured Query Language

Appendices

Appendix1 (Supplementary file 1)

Ethic References

Definitions/Terminology (English)

For peer review only - http://bmjopen.bmj.com/site/about/guidelines.xhtml

STROBE Statement—Checklist of items that should be included in reports of cross-sectional studies

Appendix 2 (Supplementary file 2)

Study Forms (English)

Form 1: Record of household visits

Form 2: Consent Form

Form 3: General household information

Part A: Demographic data/General household information

Part B: Socio-economic data

Part C: Hygiene and sanitation

Form 4: Assessment of age- and sex-stratified *generic* healthcare utilization

Part A: Assessment of *generic* healthcare utilization

Part B: Healthcare-associated factors/Vaccination/Disease perception

Form 5: Assessment of age- and sex-stratified *actual* healthcare utilization

Appendix 3 (Supplementary file 3)

Protocol, Definitions/Terminology and Study Forms (French)

Appendix 4 (Supplementary file 4)

Geospatial random selection (examples of Ghana and Madagascar)

Appendix 5 (Supplementary file 5)

System diagram of the data collection and data management: the Android application "HPA Collect" and the platform "HPA Web".

References

1. Bigogo G, Audi A, Aura B, et al. Health-seeking patterns among participants of population-based morbidity surveillance in rural western Kenya: implications for calculating disease rates. *Int J Infect Dis* 2010;14(11):e967-73. doi: 10.1016/j.ijid.2010.05.016

2. Jordan HT, Prapasiri P, Areerat P, et al. A comparison of population-based pneumonia surveillance and health-seeking behavior in two provinces in rural Thailand. *Int J Infect Dis* 2009;13(3):355-61. doi: 10.1016/j.ijid.2008.07.014

For peer review only - http://bmjopen.bmj.com/site/about/guidelines.xhtml

3. Saeed BI, Xicang Z, Yawson AE, et al. Impact of socioeconomic status and medical conditions on health and healthcare utilization among aging Ghanaians. *BMC public health* 2015;15:276. doi: 10.1186/s12889-015-1603-y [published Online First: 2015/04/18]

4. Thoa NT, Thanh NX, Chuc NT, et al. The impact of economic growth on health care utilization: a longitudinal study in rural Vietnam. *International journal for equity in health* 2013;12:19. doi: 10.1186/1475-9276-12-19 [published Online First: 2013/03/19]

5. Okafor IP, Sekoni AO, Ezeiru SS, et al. Orthodox versus unorthodox care: A qualitative study on where rural women seek healthcare during pregnancy and childbirth in Southwest, Nigeria. *Malawi medical journal : the journal of Medical Association of Malawi* 2014;26(2):45-9. [published Online First: 2014/08/27]

6. Farag TH, Kotloff KL, Levine MM, et al. Seeking care for pediatric diarrheal illness from traditional healers in Bamako, Mali. *The American journal of tropical medicine and hygiene* 2013;89(1 Suppl):21-8. doi: 10.4269/ajtmh.12-0753 [published Online First: 2013/05/01]

7. Akullian A, Ng'eno E, Matheson AI, et al. Environmental Transmission of Typhoid Fever in an Urban Slum. *PLoS Negl Trop Dis* 2015;9(12):e0004212. doi: 10.1371/journal.pntd.0004212 [published Online First: 2015/12/04]

8. Steele AD, Hay Burgess DC, Diaz Z, et al. Challenges and Opportunities for Typhoid Fever Control: A Call for Coordinated Action. *Clinical infectious diseases : an official publication of the Infectious Diseases Society of America* 2016;62 Suppl 1:S4-8. doi: 10.1093/cid/civ976 [published Online First: 2016/03/05]

9. Tran HH, Bjune G, Nguyen BM, et al. Risk factors associated with typhoid fever in Son La province, northern Vietnam. *Transactions of the Royal Society of Tropical Medicine and Hygiene* 2005;99(11):819-26. doi: 10.1016/j.trstmh.2005.05.007 [published Online First: 2005/08/16]

10. Rweyemamu M, Kambarage D, Karimuribo E, et al. Development of a One Health National Capacity in Africa : the Southern African Centre for Infectious Disease Surveillance (SACIDS) One Health Virtual Centre Model. *Current topics in microbiology and immunology* 2013;366:73-91. doi: 10.1007/82_2012_244 [published Online First: 2012/07/24]

11. Slayton RB, Date KA, Mintz ED. Vaccination for typhoid fever in sub-Saharan Africa. *Human vaccines & immunotherapeutics* 2013;9(4):903-6. doi: 10.4161/hv.23007 [published Online First: 2013/04/09]

12. Sharma PK, Ramakrishnan R, Hutin Y, et al. Risk factors for typhoid in Darjeeling, West Bengal, India: evidence for practical action. *Tropical medicine & international health : TM & IH* 2009;14(6):696-702. doi: 10.1111/j.1365-3156.2009.02283.x [published Online First: 2009/04/28]

13. Eguale T, Gebreyes WA, Asrat D, et al. Non-typhoidal Salmonella serotypes, antimicrobial resistance and co-infection with parasites among patients with diarrhea and other

For peer review only

For peer review only - http://bmjopen.bmj.com/site/about/guidelines.xhtml

gastrointestinal complaints in Addis Ababa, Ethiopia. *BMC infectious diseases* 2015;15:497. doi: 10.1186/s12879-015-1235-y [published Online First: 2015/11/06]

14. Paudyal N, Anihouvi V, Hounhouigan J, et al. Prevalence of foodborne pathogens in food from selected African countries - A meta-analysis. *International journal of food microbiology* 2017;249:35-43. doi: 10.1016/j.ijfoodmicro.2017.03.002 [published Online First: 2017/03/09]
15. Panzner U, Pak GD, Aaby P, et al. Utilization of Healthcare in the Typhoid Fever Surveillance in Africa Program. *Clinical infectious diseases : an official publication of the Infectious Diseases Society of America* 2016;62 Suppl 1:S56-68. doi: 10.1093/cid/civ891
16. von Kalckreuth V, Konings F, Aaby P, et al. The Typhoid Fever Surveillance in Africa Program (TSAP): Clinical, Diagnostic, and Epidemiological Methodologies. *Clinical infectious diseases : an official publication of the Infectious Diseases Society of America* 2016;62 Suppl 1:S9-S16. doi: 10.1093/cid/civ693
17. Park SE, Toy T, Adu-Sarkodie Y, et al. The Severe Typhoid in Africa Program: a multicentre population-based surveillance program with a nested case-control and cohort study. under submission
18. WHO. WHO vaccine-preventable diseases: monitoring system. 2017 global summary 2017 [Available from: http://apps.who.int/immunization_monitoring/globalsummary/schedules/ accessed December 17 2017.
19. WHO. WHO recommendations for routine immunization - summary tables, 2017.
20. Fleiss JL, Levin, B., Paik, M.C. Statistical Methods for Rates and Proportions. 3rd ed. New York: John. Wiley & Sons 2003.
21. Henderson RH, Sundaresan T. Cluster sampling to assess immunization coverage: a review of experience with a simplified sampling method. *Bulletin of the World Health Organization* 1982;60(2):253-60. [published Online First: 1982/01/01]
22. Henderson RH, Davis H, Eddins DL, et al. Assessment of vaccination coverage, vaccination scar rates, and smallpox scarring in five areas of West Africa. *Bulletin of the World Health Organization* 1973;48(2):183-94. [published Online First: 1973/01/01]
23. Newcombe RG. Two-sided confidence intervals for the single proportion: comparison of seven methods. *Statistics in medicine* 1998;17(8):857-72. [published Online First: 1998/05/22]
24. Katz J, Zeger SL. Estimation of design effects in cluster surveys. *Ann Epidemiol* 1994;4(4):295-301.
25. Bostoen K, Chalabi Z. Optimization of household survey sampling without sample frames. *Int J Epidemiol* 2006;35(3):751-5. doi: 10.1093/ije/dyl019
26. Panzner U, Ali M, Meyer CG, et al. Geospatial sampling frames for household-based studies: Lessons learned from a multi-country surveillance network in Senegal, South Africa and Sudan. 2017 (submitted)
27. Lohr SL. Sampling: Design and Analysis. 2nd edition ed. Pacific Grove, CA: Duxbury Press 2010.

For peer review only

For peer review only - http://bmjopen.bmj.com/site/about/guidelines.xhtml

28. Lister AJ, Scott CT. Use of space-filling curves to select sample locations in natural resource monitoring studies. *Environ Monit Assess* 2009;149(1-4):71-80. doi: 10.1007/s10661-008-0184-y

29. Keating J, MacIntyre K, Mbogo C, et al. A geographic sampling strategy for studying relationships between human activity and malaria vectors in urban Africa. *Am J Trop Med Hyg* 2003;68(3):357-65.

30. Keating J, Macintyre K, Mbogo CM, et al. Characterization of potential larval habitats for Anopheles mosquitoes in relation to urban land-use in Malindi, Kenya. *Int J Health Geogr* 2004;3(1):9. doi: 10.1186/1476-072X-3-9

31. ArcGIS. [Available from: http://pro.arcgis.com/en/pro-app/tool-reference/data-management/create-random-points.htm accessed October 2017.

32. Kondo MC, Bream KD, Barg FK, et al. A random spatial sampling method in a rural developing nation. *BMC public health* 2014;14:338. doi: 10.1186/1471-2458-14-338 [published Online First: 2014/04/11]

33. Kasper MR, Blair PJ, Touch S, et al. Infectious etiologies of acute febrile illness among patients seeking health care in south-central Cambodia. *The American journal of tropical medicine and hygiene* 2012;86(2):246-53. doi: 10.4269/ajtmh.2012.11-0409 [published Online First: 2012/02/04]

34. Dewan AM, Corner R, Hashizume M, et al. Typhoid Fever and its association with environmental factors in the Dhaka Metropolitan Area of Bangladesh: a spatial and time-series approach. *PLoS Negl Trop Dis* 2013;7(1):e1998. doi: 10.1371/journal.pntd.0001998 [published Online First: 2013/01/30]

35. Naeem Khan M, Shafee M, Hussain K, et al. Typhoid fever in paediatric patients in Quetta, Balochistan, Pakistan. *Pakistan journal of medical sciences* 2013;29(4):929-32. [published Online First: 2013/12/20]

36. Crump JA, Youssef FG, Luby SP, et al. Estimating the incidence of typhoid fever and other febrile illnesses in developing countries. *Emerging infectious diseases* 2003;9(5):539-44. [published Online First: 2003/05/10]

37. Osei-Tutu B, Anto F. Trends of reported foodborne diseases at the Ridge Hospital, Accra, Ghana: a retrospective review of routine data from 2009-2013. *BMC infectious diseases* 2016;16:139. doi: 10.1186/s12879-016-1472-8 [published Online First: 2016/03/26]

38. (ILO) ILO. International Standard Classification of Occupations ISCO-08, 2012 [Available from: http://www.ilo.org/wcmsp5/groups/public/---dgreports/---dcomm/---publ/documents/publication/wcms_172572.pdf.

For peer review only - http://bmjopen.bmj.com/site/about/guidelines.xhtml

Appendix 1/Supplementary file 1:
Ethic References
Definitions/Terminology (English)
STROBE Statement—Checklist of items that should be included in reports of *cross-sectional studies*

Ethics References

- IVI Institutional Review Board (IRB), No. 2016-003
- Institute of Tropical Medicine Antwerp Institutional Review Board, Belgium
- Universiteit Antwerpen, Comite voor medische Ethiek, Belgium
- Ministère de la Santé du Burkina Faso – Comité d'Ethique pour la Recherche en Santé, Burkina Faso
- Comité d'Ethique de l'Ecole de Santé Publique de l'Université de Kinshasa, Democratic Republic of Congo
- National Research Ethics Review committee (NRERC) Ministry of Science and Technology, Federal Democratic Republic of Ethiopia
- AHRI-ALERT Ethics Review Committee (AAERC), Ethiopia
- Kwame Nkrumah University of Science and Technology, School of Medical Sciences/Komfo Anokye Teaching Hospital, Committee on Human Research, Publication and Ethics, Ghana
- Ministère de la Santé du Repoblikan'l Madagaskar – Comité d'Ethique
- University of Ibadan/University College Hospital Ethics Committee, Ibadan, Nigeria

Definitions/Terminology (English)

**A**	
Abdominal pain/ Intestinal pain	Pain (acute/short-lived or chronic/long-lived) that occurs anywhere between the chest and the groin (often referred to as the stomach region/belly).
Aches/Pain	A condition of having continuous/prolonged dull pain in a part of the body; the physical feeling, suffering, or discomfort caused by an illness or injury.
**B**	
Bleeding (internal/external)/ Blood loss	The condition of losing blood from the vascular system, either internally into the body (internal bleeding) or externally through a natural orifice or break in the skin (external bleeding); blood escaping from an injured blood vessel.
Blood loss/ Bleeding	The condition of losing blood from the vascular system, either internally into the body (internal bleeding) or externally through a natural orifice or break in the skin (external bleeding); blood escaping from an injured blood vessel.
Blood pressure disorders	A high/low blood pressure or a blood pressure that shows excessive fluctuation.
**C**	
Chills/Shivering	The sensation of coldness that could be accompanied by shivering or shaking of the body and pallor of the skin.
Cholera	An infectious, sometimes fatal disease of the small intestine caused by the bacterium *Vibrio cholerae*; it is spread via the fecal-oral route from the ingestion of contaminated water and food and causes chills, shivering, severe watery diarrhea, vomiting, and dehydration.
Confusion/Dizziness/ Unconsciousness	The state of being bewildered or unclear in mind; an abnormal state of response to stimuli in the environment; faint, wooziness, weakness, unsteadiness or a tendency to fall; the interruption of the awareness of oneself and one's surroundings.
Convulsion	The sudden, violent, irregular movement of a limb/part of the body or of the entire body which is caused by involuntary contraction of muscles.
Cough	The condition of expelling air from the lungs suddenly/rapidly and with a harsh noise; often involuntarily in order to clear the lung airways of fluids/mucus/other material.
**D**	

For peer review only - http://bmjopen.bmj.com/site/about/guidelines.xhtml

Dehydration	A condition of excessive loss of water/moisture from the body.
Diarrhea	A condition of passing feces from the body very frequently and in a liquid rather than solid form.
Diphtheria	A highly contagious, sometimes fatal disease caused by the bacterium *Corynebacterium diphtheriae*. It is spread from person-to-person, through the air or contaminated objects and affects the upper respiratory tract. Common symptoms are sore throat, swollen glands/ lymph nodes, malaise, fever/chills, a membranous substance/thick gray material that forms in the throat/tonsils and makes it difficult to breathe/ rapid breathing ("barking cough") and swallow. A form of diphtheria that involves the skin, eyes, or genitals is also reported and is characterized by pain, redness, and swelling of the skin.
Dizziness/Confusion/ Unconsciousness	The state of being bewildered or unclear in mind; an abnormal state of response to stimuli in the environment; faint, wooziness, weakness, unsteadiness or a tendency to fall; the interruption of the awareness of oneself and one's surroundings.
**E**	
Edema/Swelling	The excess collection of a watery fluid/swelling in the cavities or tissues of the body; it's commonly noticed in the hands/arms/feet/legs; volume increase of tissue.
Education - formal	A systematic, organized education model that is structured and administered according to a given set of laws and norms; formal education institutions are administratively, physically and curricularly organized and require a minimum classroom attendance from students; it involves intermediate and final assessments in order to advance to the next learning stage; it confers degrees and diplomas pursuant to a quite strict set of regulations.
**F**	
Fatigue/Malaise/ Weakness	The state of feeling tired, exhausted, or weak; a general feeling of discomfort, uneasiness, unhealthiness, or unhappiness.
Fever, any	An abnormally high body temperature (usually above 38°C).
Fever <3 days	An abnormally high body temperature (usually above 38°C) that appears continuously for less than 3 days.
Fever >3 days	An abnormally high body temperature (usually above 38°C) that lasts longer than 3 days and appears continuously.
**G**	
**H**	
Headache	Any kind of pain in any region of the head.
Healthcare - actual	Healthcare that is related to specific sign/symptom-associated disease onsets or disease onset dates.
Healthcare - general	Healthcare that is unrelated to specific sign/symptom-associated disease onsets or disease onset dates.
Healthcare facility	Places or institutions where healthcare is provided; including hospitals, clinics, healthcare centers, health posts, and specialized care centers.
Haemophilus influenzae type B	A bacterium that causes a range of illnesses and is spread by droplets through coughs and sneezing, and occurs primarily among children <5years of age; common signs are fever, headache, and stiff neck up to sepsis. It can cause ear infections, cellulitis (soft tissue infection), arthritis, upper respiratory infections, pneumonia, meningitis (potential brain damage) and epiglottitis (with airway obstructions).
Heart disorders	Any disorder that affects or is caused by the heart; conditions that involve narrowed/blocked blood vessels that can lead to heart attack, chest pain, or stroke; condition that may affect one's heart muscle, valves, rhythm, or blood pressure.
Hepatitis B	An infectious disease caused by the hepatitis B virus (HBV) that affects the liver (acute/chronic liver infection; may result in liver failure, cancer, or cirrhosis). The virus is spread from person-to-person through blood, semen, or other bodily fluids. Common symptoms are fever, malaise/weakness/fatigue, loss of appetite, nausea/ vomiting, abdominal discomfort/pain, dark-colored urine, joint pain and jaundice.
Hepatitis E	An infectious disease caused by the hepatitis E virus (HEV) that affects the liver (only acute liver infection; may result in acute liver failure). The virus is spread via the fecal-oral route through the ingestion of contaminated food and water, infected

For peer review only - http://bmjopen.bmj.com/site/about/guidelines.xhtml

	animals (zoonotically) and blood. Common symptoms are fever, malaise, loss of appetite, diarrhea, nausea, abdominal discomfort, dark-colored urine, joint pain and jaundice.
HIV/AIDS	An infectious disease caused by the human immunodeficiency virus (HIV) that affects/damages the immune system and results in the acquired immunodeficiency syndrome (AIDS), a chronic, potentially life-threatening condition; it is transmitted through infectious body fluids such as blood, semen, and other bodily fluids. Common symptoms of an acute/early infection are fever, fatigue, headache, aches/joint pain, rash, sore throat, swollen glands/lymph nodes, diarrhea, weight loss, oral yeast infection and shingles. The untreated disease progresses to AIDS; symptoms caused by the severely damaged immune system are night sweats, recurring fever, chronic diarrhea, lesions on tongue and in the mouth, fatigue, weight loss, and skin rushes.
Household	A person or a group of related or unrelated persons that live together in the same dwelling unit, that acknowledge one male or female adult as the head of the household, that share the same housekeeping arrangements, that are considered to constitute one unit, and that provide themselves with food or other essentials for living. A household may be located in a single housing unit (single-story building) or in a set of collective living quarters (multi-story building).
**I**	
Income/Wage	Money or another form of payment that someone receives periodically in exchange for providing a good or service. It is usually received from a job/occupation.
Influenza	An infectious, highly contagious disease of the respiratory tract that is caused by flu viruses; it is spread from person-to-person via infected saliva droplets and contaminated objects. Common symptoms are fever, aches in muscles/arms/legs, chills/sweats, headache, fatigue/weakness, runny nose/nasal congestion, sneezing, sore throat and dry persistent cough.
Intestinal pain/ Abdominal pain	Pain (acute/short-lived or chronic/long-lived) that occurs anywhere between the chest and the groin (often referred to as the stomach region/belly).
**J**	
Jaundice	A condition of yellowing of the skin/whites of the eyes.
**K/L**	
**M**	
Malaise/Fatigue/ Weakness	The state of feeling tired, exhausted, or weak; a general feeling of discomfort, uneasiness, unhealthiness, or unhappiness.
Malaria	An infectious, sometimes fatal disease caused by a parasite (*Plasmodium* spp.) that is transmitted primarily by the bite of infected mosquitoes; infection may also spread from mother-to-child prenatally or during delivery, or through contact with contaminated blood. Common symptoms are recurrent attacks of chills, sweating, high fever, headache, vomiting and diarrhea.
Measles	An infectious, sometimes fatal disease of the respiratory tract (nose/throat) that is caused by a virus that affects mainly children <5years of age; it is spread from person-to-person via infected saliva droplets and contaminated objects. Common symptoms are fever, dry cough, runny nose, inflamed eyes, sore throat, large flat spots on the skin, and white spots in the mouth and on the cheek.
Meningitis	A viral/bacterial/fungal, sometimes life-threatening infectious inflammation of the membranes (meninges) surrounding the brain and spinal cord; it is spread from person-to-person via infected saliva droplets and contaminated objects. Common symptoms are high fever, severe headache, nausea/vomiting, confusion, seizures, loss of appetite and thirst, fatigues, sensitivity to light, skin rush and stiff neck.
Mumps	A viral infectious disease caused by the mumps virus that affects mainly the parotid glands that are situated below and in front of the ears; it is spread from person-to-person via infected saliva droplets and contaminated objects. Common symptoms are fever, swelling of one/both parotid glands, headache, muscle aches, weakness/fatigue, loss of appetite and pain while shewing/swallowing; a known complication may be hearing loss.

For peer review only - http://bmjopen.bmj.com/site/about/guidelines.xhtml

**N**	
Nausea/Vomiting	A feeling of sickness in the stomach with an inclination to vomit; a condition of ejecting part or all of the contents of the stomach, primarily through the mouth.
Neighbor	A person (related or unrelated) living next door to a household; a neighbor does not live together with members of a household (next door) in the same dwelling unit, does not share the same housekeeping arrangements, food, or other living essentials with members of a household (next door), and does not constitute one unit with members of a household (next door).
**O**	
Occupation	A job, or means of earning a wage/living; often requires the mastery of a complex set of knowledge, tasks, duties and skills acquired through formal education and/or practical experience.
**P**	
Pain/Aches	A condition of having continuous/prolonged dull pain in a part of the body; the physical feeling, suffering, or discomfort caused by an illness or injury.
Pertussis	Pertussis, also known as whooping cough, is a highly contagious bacterial disease. Symptoms similar to a common cold like runny nose, fever and cough are followed by weeks of sever coughing and a high-pitched whooping sound or gasping when a diseased person breathes in; severe coughing my even be followed by vomiting, breaking of ribs, or extreme fatigue. Infected very young and old people may have little or no cough, or they may have periods of interrupted breathing during which they do not breath are all. Pertussis is transmitted by the bacterium *Bordetella pertussis* through coughs and sneezes of an infected person.
Pharmacy	A place where medicines (prescription or nonprescription) are prepared, preserved, compounded and dispensed.
Physician	A person skilled/trained in healing; a person educated, clinically experienced, and licensed to practice medicine.
Pneumococcus	A bacterial infection caused by the bacterium *Streptococcus pneumoniae*; a sometimes fatal inflammation of the lungs that often affects immunocompromised people; it is spread from person-to-person via infected saliva droplets and contaminated objects. Common symptoms are fever, chills, cough (with phlegm/pus), chest pain, difficulty in breathing/shortness of breath, fatigue, nausea/vomiting and diarrhea; rare symptoms are sepsis, pleural effusion, and empyema.
Polio	A viral, highly contagious, sometimes fatal infectious disease caused by the poliovirus; in its most severe form causes paralysis and difficulties in breathing; it is transmitted via the fecal-oral route through ingestion of contaminated water and food, and from person-to-person contact. Common symptoms are fever, sore throat, headache, vomiting, fatigue, back pain/neck pain/stiffness, muscle weakness/aches, meningitis, loss of reflexes, and loose/floppy limbs.
**Q**	
**R**	
Rapid breathing/ Shortness of breath	The condition of abnormally fast breathing; a rate of >20 breaths per minute for adults, >30 breaths per minute for children and >45 breaths per minute for babies.
Respondent	An adult (at the country-specific legal age of majority) household member who is a decision-maker for the entire household within the study site; thus, this adult person serves as a proxy for all household members. This person may be identified by other members of the same household as the person who is primarily involved in the daily healthcare of household members.
Rotavirus	A viral infectious disease caused by rotavirus that results predominantly in diarrhea among children <5years of age; it spreads through the fecal-oral route from person-to-person contact and contaminated objects. Common symptoms are watery diarrhea, fever, vomiting, abdominal pain and dehydration.
Rubella	A contagious, viral infectious disease caused by the rubella virus; known by its distinctive red rash; it is spread from person-to-person via infected saliva droplets), from mother-to-child prenatally via blood, and contaminated objects. Common

For peer review only - http://bmjopen.bmj.com/site/about/guidelines.xhtml

	symptoms are mild fever, headache, stuffy/runny nose, inflamed red eyes, enlarged tender lymph nodes at the base of the skull/back of the neck/behind the ears, joint pain and a fine pink rash that moves from the face to the trunk, arms and legs.
Runny nose	The condition when the nose (nasal and adjacent tissues and blood vessels) produces extra mucus/excess drainage.
**S**	
Self-treatment	The medication of oneself or treatment of one's own disease or condition without medical supervision or prescription.
Severe breathing/ Difficulties in breathing	The condition of abnormal breathing that may be accompanied by wheezing, chest pain, chest in-drawing, stridor, swelling in the chest, or cough for instance.
Shivering/Chills	The sensation of coldness that could be accompanied by shivering or shaking of the body and pallor of the skin.
Shortness of breath/ Rapid breathing	The condition of abnormally fast and deep breathing; a rate of >20 breaths per minute for adults, >30 breaths per minute for children and >45 breaths per minute for babies.
Sneezing	The condition of suddenly forcing air out through nose and mouth with a usual loud noise.
Swelling/Edema	A condition of an excess collection of a watery fluid/swelling in the cavities or tissues of the body; it's commonly noticed in the hands/arms/feet/legs; volume increase of tissue.
**T**	
Tetanus	Tetanus is an infection characterized by muscle spasms which commonly begins in the jaw and progresses to the rest of the body. The spasms usually last for a few minutes only and occur frequently during 3-4 weeks of the illness but can be so severe that they may cause bone fractures or death when they affect the respiratory muscles. Tetanus may be characterized by further symptoms like fever, sweating, headache, difficulties in swallowing, high blood pressure, and an increased heart rate. The disease is caused by an infection with the bacterium *Clostridium tetani* that is found in soil, salvia, dust and manure. The bacterium enters through a skin injury (e.g. cut, puncture wound) by a contaminated object. It produces toxins that interfere with muscle contractions that result in the typical spasms.
Traditional healer	A person that aims to maintain health as well as to prevent, diagnose, and improve or treat illnesses by using a sum of knowledge, skills and practices based on theories, beliefs and experiences.
Tuberculosis	A contagious, potentially serious infectious disease caused by the bacteria *Mycobacterium tuberculosis*; primarily affects the lungs, but can also affect the kidneys and spine/brain; it is transmitted from person-to-person via infected saliva droplets. Common symptoms of active tuberculosis are cough (with or without blood), chest pain, weight loss, fatigue, fever, night sweats, chills and loss of appetite.
Typhoid fever	A bacterial, potentially life-threatening infectious disease caused by *Salmonella typhi*; it is transmitted via the fecal-oral route through contaminated food and water, or from person-to-person contact. Common symptoms are high fever, headache, abdominal pain, constipation/diarrhea, and rash. Serious complications (i.e. perforation of the gut) are reported.
**U**	
Unconsciousness/ Dizziness/Confusion	The state of being bewildered or unclear in mind; an abnormal state of response to stimuli in the environment; faint, wooziness, weakness, unsteadiness or a tendency to fall; the interruption of the awareness of oneself and one's surroundings.
**V**	
Varicella	A viral, highly contagious infectious disease, commonly known as chickenpox, that primarily affects children and is caused by the varicella-zoster virus; it is spread from person-to-person by contact with infected blisters or saliva droplets. Common symptoms are mild fever, headache, backache, loss of appetite, and vesicular skin eruptions/rash/red spots on the back and chest accompanied by severe itching.

For peer review only - http://bmjopen.bmj.com/site/about/guidelines.xhtml

Visitor	A person (related or unrelated) who comes to a household to spend some time with household members; a visitor does not live together with household members in the same dwelling unit, does not share the same housekeeping arrangements, food, or other living essentials with household members, and does not constitute one unit with household members.
Vomiting/Nausea	A feeling of sickness in the stomach with an inclination to vomit; a condition of ejecting part or all of the contents of the stomach, primarily through the mouth.
**W**	
Weakness/Malaise/ Fatigue	The state of feeling tired, exhausted, or weak; a general feeling of discomfort, uneasiness, unhealthiness, or unhappiness.
Weight loss	The (voluntary or involuntary) decrease in body weight.
Witness	A person who is an adult (at the country-specific legal age of majority), who is independent of the study, who cannot be unfairly influenced by study staff, who attends the informed consent process if the subject or subject's legally acceptable representative is illiterate, and who reads (literate) the informed consent form and any other written information supplied to the subject.
**X/Y/Z**	
Yellow fever	An acute infectious, human pathogenic, viral disease that is marked by the sudden onset of symptoms like fever, chills, loss of appetite, nausea, muscle pain, and headache, that typically resolve within a few days or are followed by more serious symptoms such as jaundice, high fever and hemorrhage. The disease is caused by a virus of the genus Flavivirus and is transmitted by the bite of an infected mosquito.

For peer review only

STROBE Statement—Checklist of items that should be included in reports of ***cross-sectional studies***

	Item No	Recommendation	
**Title and abstract**	1	(*a*) Indicate the study's design with a commonly used term in the title or the abstract	√
		(*b*) Provide in the abstract an informative and balanced summary of what was done and what was found	√
**Introduction**			
Background/rationale	2	Explain the scientific background and rationale for the investigation being reported	√
Objectives	3	State specific objectives, including any prespecified hypotheses	√
**Methods**			
Study design	4	Present key elements of study design early in the paper	√
Setting	5	Describe the setting, locations, and relevant dates, including periods of recruitment, exposure, follow-up, and data collection	√
Participants	6	(*a*) Give the eligibility criteria, and the sources and methods of selection of participants	√
Variables	7	Clearly define all outcomes, exposures, predictors, potential confounders, and effect modifiers. Give diagnostic criteria, if applicable	√
Data sources/ measurement	8*	For each variable of interest, give sources of data and details of methods of assessment (measurement). Describe comparability of assessment methods if there is more than one group	√
Bias	9	Describe any efforts to address potential sources of bias	√
Study size	10	Explain how the study size was arrived at	√
Quantitative variables	11	Explain how quantitative variables were handled in the analyses. If applicable, describe which groupings were chosen and why	√
Statistical methods	12	(*a*) Describe all statistical methods, including those used to control for confounding	√
		(*b*) Describe any methods used to examine subgroups and interactions	√
		(*c*) Explain how missing data were addressed	Not

For peer review only

			applicable
		(*d*) If applicable, describe analytical methods taking account of sampling strategy	Not applicable
		(*e*) Describe any sensitivity analyses	Not applicable
**Results**			
Participants	13*	(a) Report numbers of individuals at each stage of study—eg numbers potentially eligible, examined for eligibility, confirmed eligible, included in the study, completing follow-up, and analysed	Not applicable
		(b) Give reasons for non-participation at each stage	Not applicable
		(c) Consider use of a flow diagram	Not applicable
Descriptive data	14*	(a) Give characteristics of study participants (eg demographic, clinical, social) and information on exposures and potential confounders	Not applicable
		(b) Indicate number of participants with missing data for each variable of interest	Not applicable
Outcome data	15*	Report numbers of outcome events or summary measures	Not applicable
Main results	16	(*a*) Give unadjusted estimates and, if applicable, confounder-adjusted estimates and their precision (eg, 95% confidence interval). Make clear which confounders were adjusted for and why they were included	Not applicable
		(*b*) Report category boundaries when continuous variables were categorized	Not applicable
		(*c*) If relevant, consider translating estimates of relative risk into absolute risk for a meaningful time period	Not applicable
Other analyses	17	Report other analyses done—eg analyses of subgroups and interactions, and sensitivity analyses	Not applicable

For peer review only

**Discussion**			
Key results	18	Summarise key results with reference to study objectives	Not applicable
Limitations	19	Discuss limitations of the study, taking into account sources of potential bias or imprecision. Discuss both direction and magnitude of any potential bias	Not applicable
Interpretation	20	Give a cautious overall interpretation of results considering objectives, limitations, multiplicity of analyses, results from similar studies, and other relevant evidence	Not applicable
Generalisability	21	Discuss the generalisability (external validity) of the study results	Not applicable
**Other information**			
Funding	22	Give the source of funding and the role of the funders for the present study and, if applicable, for the original study on which the present article is based	√

For peer review only

Site ___ ___ ___ Subarea ___ ___ ___ House number ___ ___ ___ ___ Interviewer ID ___ ___

Appendix 2/Supplementary file 2: Study forms (English)

FORM 1 – RECORD OF HOUSEHOLD VISITS

Instructions to the interviewer

– Complete "Form 1 – RECORD OF HOUSEHOLD VISITS" for <u>every household</u> visited.
– Complete "Home/Respondent/Completed" once <u>all survey parts</u> have been completed; if not all data haven been collected at the initial and/or a consecutive visit (max. 3 visits), schedule a revisit.
– <u>Attempt 1:</u> complete the status of the household visit using given options; for "Not Home", "Home/Non-Respondent" and "Home/Respondent/Unavailable" schedule a revisit; for "Home/Respondent/Refusal" state the reason for refusal and visit a replacement household to the nearest on the right or left side of this household; for ~~and~~ "Non-existing/Non-residential" skip this household and visit a replacement household to the nearest on the right or left side of this household instead.
– <u>Attempt 2:</u> complete the status of the household visit using given options; for "Not Home", "Home/Non-Respondent" and "Home/Respondent/Unavailable" schedule a revisit.
– <u>Attempt 3:</u> complete the status of the household visit using given options; for "Not Home", "Home/Non-Respondent", "Home/Respondent/Unavailable" skip this household and visit a replacement household to the nearest on the right or left side of this household instead.
– "Site" and "Subarea" must be entered each as a 3-digit, "House number" as a 4-digit, and "Interviewer ID" as a 2-digit code (use lists individually prepared for each site to obtain codes); enter "Date" in the format DD/MM/YY and "Time" in the format HH:MM and circle AM or PM. "Study label" must be a combination of a 3-digit "Site" code, a 3-digit "Subarea" code and a 4-digit "House number" code; the "Interviewer ID" must be a entered as a 3-digit code.

**Attempt 1** Date ____/____/____ (DD/MM/YY)	Time _____/_____ AM/PM	Interviewer ID ___ ___
STATUS	Tick one	
1=Not home	O	➧ Schedule revisit (Date/Time)
2=Home/Non-Respondent	O	➧ Schedule revisit (Date/Time)
3=Home/Respondent/Unavailable	O	➧ Schedule revisit (Date/Time)
4=Home/Respondent/Completed	O	
5=Home/Respondent/Refusal	O	Record the reason(s) for refusal; tick all that applies<br>O 1=No time to answer questions<br>O 2=Don't want to participate/answer questions<br>O 3=Other ______________________________<br>O 98=No response O 99=Don't know<br>➧ Visit a replacement household to the nearest right or left side of this household
6=Non-existing/Non-residential	O	➧ Visit a replacement household to the nearest right or left side of this household
**Attempt 2** Date ____/____/____ (DD/MM/YY)	Time _____/_____ AM/PM	Interviewer ID ___ ___
STATUS	Tick one	
1=Not home	O	➧ Schedule revisit (Date/Time)
2=Home/Non-Respondent	O	➧ Schedule revisit (Date/Time)
3=Home/Respondent/Unavailable	O	➧ Schedule revisit (Date/Time)

For peer review only - http://bmjopen.bmj.com/site/about/guidelines.xhtml

Site ___ ___ ___ Subarea ___ ___ ___ House number ___ ___ ___ ___ Interviewer ID ___ ___

4=Home/Respondent/Completed	O	
5=Home/Respondent/Refusal	O	Record the reason(s) for refusal; tick all that applies<br>O 1=No time to answer questions<br>O 2=Don't want to participate/answer questions<br>O 3=Other ________________<br>O 98=No response O 99=Don't know<br>➡ Visit a replacement household to the nearest right or left side of this household
6=Non-existing/Non-residential	O	➡ Visit a replacement household to the nearest right or left side of this household
**Attempt 3** Date ____/____/____ (DD/MM/YY)	Time _____/_____ AM/PM	Interviewer ID ___ ___
STATUS	Tick one	
1=Not home	O	➡ Visit a replacement household to the nearest right or left side of this household
2=Home/Non-Respondent	O	➡ Visit a replacement household to the nearest right or left side of this household
3=Home/Respondent/Unavailable	O	➡ Visit a replacement household to the nearest right or left side of this household
4=Home/Respondent/Completed	O	
5=Home/Respondent/Refusal	O	Record the reason(s) for refusal; tick all that applies<br>O 1=No time to answer questions<br>O 2=Don't want to participate/answer questions<br>O 3=Other ________________<br>O 98=No response O 99=Don't know<br>➡ Visit a replacement household to the nearest right or left side of this household
6=Non-existing/Non-residential	O	➡ Visit a replacement household to the nearest right or left side of this household

For peer review only

For peer review only - http://bmjopen.bmj.com/site/about/guidelines.xhtml

Site ___ ___ ___ Subarea ___ ___ ___ House number ___ ___ ___ ___ Interviewer ID ___ ___

FORM 2 – CONSENT FORM

Healthcare assessment in [add study site and -country]

Instructions to the interviewer

- Read "Form 2 – CONSENT FORM" to the respondent/ask the respondent to carefully read through "Form 2 – CONSENT FORM" if preferred.
- The respondent as defined for this investigation is an adult (at the country-specific legal age of majority) household member and decision-maker with respect to daily healthcare/healthcare utilization for the entire household and its members; it must not be e.g. a neighbour or a visitor.
- Assure that the respondent has fully understood the purpose, procedures, risks and benefits of this study prior to obtaining the respondent's consent to participate.
- The respondent must sign "Form 2 – CONSENT FORM"; if the respondent is unable to sign/illiterate, a thumbprint will serve in place of a signature and a study-independent literate adult witness (e.g. member of same household/neighbour) will sign "Form 2 – CONSENT FORM".
- Collect data about all household members regardless of whether all household members are present at the time of the interview; collecting and recording data only about individuals present at the time of the interview will result in biased data.
- Complete "Form 2 – CONSENT FORM" once for every household consented/enrolled in the study.
- Explain to the respondent that it is anticipated to revisit every household during a 2-year period; the study team will obtain a new consent for participation during each visit.
- "Site" and "Subarea" must be entered each as a 3-digit, "House number" as a 4-digit, "Interviewer ID" as a 2-digit code (use lists individually prepared for each site to obtain codes), and "Date" in the format DD/MM/YYYY. "Study label" must be a combination of a 3-digit "Site" code, a 3-digit "Subarea" code and a 4-digit "House number" code; the "Interviewer ID" must be entered as a 3-digit code.

Study principal- and co-investigators

[add names of study principal- and co-investigators and respective affiliations]

Introduction

The International Vaccine Institute (IVI) and the [add affiliation(s) of local investigators] are working together to look into health problems and healthcare seeking behaviour in your community [add study site and -country], including the socio-economic status, hygiene and sanitation behaviour, and the vaccination status among children. We would like to better understand where you, your family and your neighbours seek healthcare in case of medical conditions associated with fever/hot body and other signs & symptoms. This information will help us to find out how common fever-related and other conditions are, provide a better picture of influencing factors involved in disease transmission and infection, and to find ways to prevent and treat illnesses in your community. You, the primary decision-maker for daily healthcare/healthcare utilization for this household and its members, are invited to participate because you live in the area where we are conducting this study. This consent form will be read by you/to you and you should take your time to make your decision to accept or decline the participation in the study. Please ask members of the study team to explain any word(s) or information that you do not understand. Further information about this study, including its potential risks, benefits and inconveniences will be explained subsequently. After the study has been fully explained to you and if you agree to participate, you will be asked to sign this consent form on behalf of all household members. If you are unable to sign the form, we will ask you to provide your thumbprint, and a study-independent adult person (member of your household/neighbour) will sign the form as a witness. We will give you a signed and dated copy of the consent form.

Purpose

The purpose of this study is to look into health problems and utilization of healthcare in your community [add study site and -country], including the socio-economic status and hygiene and sanitation behaviour. We aim to better understand where you, your family and your neighbours seek healthcare for conditions associated with fever/hot body or other sings & symptoms. This information will help better estimate the burden of illnesses common in your community, and to identify ways to introduce measures to prevent and treat them.

For peer review only - http://bmjopen.bmj.com/site/about/guidelines.xhtml

Site ___ ___ ___ Subarea ___ ___ ___ House number ___ ___ ___ ___ Interviewer ID ___ ___

Procedures

We will visit about [add number for study site and -country] households in total in [add study site and -country] and will ask the same questions to each household. Your household was chosen for this study simply by chance. If you agree to participate, we will ask you on behalf of all household members a few questions about your household such as the initials, sex and age of each member. We would like to know some information related to the education and occupation of the household head, and income, possession of household items, the housing itself as well as hygiene and sanitation in the context of personal hygiene, food preparation and handling, water sources and handling, waste disposal, handling of farm and domestic animals as well as farming of this household. We will also ask where members of your household get help if anyone is sick due to fever-related and other conditions and about factors that may influence healthcare seeking behaviour of household members (i.e. travel modalities, availability of health insurance, cultural/ethnographic factors) besides the immunization status of children of this house and the perception of some diseases. Finally, we will measure the geographic location of your house. There are no right or wrong answers. You are free to choose which questions to answer. All in all, it should take about 30-45 minutes to answer all questions. The interview may take a bit longer if there are many members in this household. Also, we will visit your household two times in different seasons (once during the dry season and once towards the end of the rainy season) during the overall study period of about two years to collect data as described in this paragraph of the consent form.

Participation

You, the primary decision-maker for daily healthcare/healthcare utilization for this household and its members, and members of your household are free to choose to accept or decline to be part of the study. If you do not want to participate, that is ok. If you want to stop your participation at any time, that is ok, and there will be no harm to you or members of your household. So, participation in this study is voluntary, and you are free to refuse to participate in the study or you can withdraw your consent at any time without giving reasons and this will not involve any penalty.

Risks & Benefits

There are no direct risks for participating in this study. Asking questions about your household may make you or household members feel uncomfortable. Study teams may help providing information on established community healthcare resources for participants that express distress after completing the questions. You can refuse to answer any question at any time. You may take a break or stop participating in this study at any time. There are no direct benefits for being in this study. During visits of your household, our study teams may identify members of your household that should seek healthcare for fever-associated conditions and others. Hence, they may help providing information on established healthcare resources in your community. Indirect benefits are that all information collected will provide exact information on health problems and healthcare utilization for various conditions, including vaccination for young children, the socio-economic status as well as hygiene and sanitation behaviour in your community. This will help to provide a better picture of influencing factors involved in disease transmission and infection, and to estimate the burden of common diseases as well as to find ways to prevent and treat those in your community.

Costs & Compensation

There are no costs for participants of this study. You and anyone from your household will not be compensated for your participation.

Confidentiality

All study records and all collected information that identify you and members of your household will be kept confidential. All paper-based files will be locked safely and computerized files will be password-protected; all data files (paper-based/computerized) will be stored for a minimum of five years; these files will be accessible to authorized few study staff only. Your name or any identifier will not be used in any publication or reports from this study. Information that we collect about you and members of your household will only be shared with authorized study members such as authorized study staff and officials from ethics committees.

For peer review only - http://bmjopen.bmj.com/site/about/guidelines.xhtml

Site ___ ___ ___ Subarea ___ ___ ___ House number ___ ___ ___ ___ Interviewer ID ___ ___

Contact point for questions

If you or members of your household have any questions, please ask them now to the study team or at any time later by contacting [add name of local principal- and/or co-investigator, respective affiliations, and phone numbers]. If you have questions as a study participant about your/your household members' rights, you can contact the [add name and contact details of the local ethics committee].

Statement of consent

This consent form for participating in the assessment of health behaviour and other population-based factors in [add study site and -country] has been read by me/to me carefully. The purpose, procedures, risks & benefits have been explained to me in detail. I have been allowed to ask questions at any time, and my questions have been answered to my satisfaction by the study team. I have been told whom to contact if I have questions, want to discuss problems or concerns. I have been told that I will be given a signed and dated copy of this consent form. I have been reassured that all information obtained as result of this study will be kept confidential and used only for the purpose of this study and only by participating institutions.

I, the respondent, consent hereby voluntarily to participate in this study. I will follow the directions of the study team and give them my full cooperation. I understand that I have the right to withdraw from the study at any time.

RESPONDENT

Name: ______________________________

(given name + middle name (if available) + family name)

Signature: ______________________________

(handwritten depiction of person's name/mark that the person writes on a document as a proof of identity)

Date ______/______/______________ (DD/MM/YYYY)

Thumbprint

If the respondent is unable to sign/illiterate but agrees to participate, record the respondent's thumb print in the above box, and have an adult study-independent literate witness (e.g. member of the same household or a neighbor) sign below:

WITNESS

Name: ______________________________

(given name + middle name (if available) + family name)

Signature: ______________________________

(handwritten depiction of person's name/mark that the person writes on a document as a proof of identity)

Date ______/______/______________ (DD/MM/YYYY)

I, the interviewer, have read/explained the study to the above named respondent (witness if respondent is illiterate) in a language that the respondent understands well. I am certain that the respondent has understood the information and he/she agrees to be asked some questions.

INTERVIEWER

Name: ______________________________

(given name + middle name (if available) + family name)

Signature: ______________________________

(handwritten depiction of person's name/mark that the person writes on a document as a proof of identity)

Date ______/______/______________ (DD/MM/YYYY)

For peer review only - http://bmjopen.bmj.com/site/about/guidelines.xhtml

Site ___ ___ ___ Subarea ___ ___ ___ House number ___ ___ ___ ___ Interviewer ID ___ ___

FORM 3: GENERAL HOUSEHOLD INFORMATION

Instructions to the interviewer

- Complete this form only if the respondent consented to participate (see "Form 2 – CONSENT FORM"); the respondent as defined for this investigation is an adult (at the country-specific legal age of majority) household member and decision-maker with respect to daily healthcare/healthcare utilization for the entire household and its members; it must not be e.g. a neighbour or a visitor.
- Collect data about all household members regardless of whether all household members are present at the time of the interview; collecting and recording data only about individuals present at the time of the interview will result in biased data.
- A household is/household members are defined as a person or a group of related or unrelated persons that live together in the same dwelling unit, that acknowledge one male or female adult as the head of the household, that share the same housekeeping arrangements, that are considered to constitute one unit, and that provide themselves with food or other essentials for living.
- "Site" and "Subarea" must be entered each as a 3-digit, "House number" as a 4-digit, and "Interviewer ID" as a 2-digit code (use lists individually prepared for each site to obtain codes). "Study label" must be a combination of a 3-digit "Site" code, a 3-digit "Subarea" code and a 4-digit "House number" code; the "Interviewer ID" must be entered as a 3-digit code.

FORM 3: GENERAL HOUSEHOLD INFORMATION
PART A – DEMOGRAPHIC DATA/GENERAL HOUSEHOLD INFORMATION

HOUSE LOCATION

1 Site, subarea and house number
Instructions: Site (=study site) and subarea (=smallest unit of the study site) must be recorded each as a 3-digit, and house number as a 4-digit code; use individually prepared lists to obtain the respective codes.

Site: ____ ____ ____ Subarea: ____ ____ ____ House number: ____ ____ ____ ____

2 GPS coordinates
Instructions: Take GPS coordinates at the approx. center of the house. Record altitude as a 4-digit number (unit: meter), and latitude/longitude as a 7-digit number (unit: degrees, decimal minutes).

O 1=Original household O 2=Replacement household (right/left side of original household)

Altitude: ___ ___ ___ ___

Latitude: ___ ___°___ ___.___ ___ ___' Longitude: ___ ___°___ ___.___ ___ ___'

RESPONDENT

3 Provide the full name of the respondent.
Instructions: Record given name, middle name (if available) and family name of the respondent.

____

4 Provide the age of the respondent.
Instruction: Recorded as a 3-digit number (unit: year). ____ ____ ____

5 Provide the sex of the respondent.
O 1=Male O 2=Female

6 What is the respondent's relationship to this household?
O 1=Household head
O 2=Son/daughter of household head
O 3=Brother/sister of household head
O 4=Mother/father of household head
O 5=Granddaughter/grandson of household head
O 6=Other, specify __________
O 7=Spouse of household head
O 8=Spouse of son/daughter of household head
O 9=Spouse of brother/sister of household head
O 10=Mother/father in law of household head
O 11=Niece/nephew of household head
O 98=No response

For peer review only - http://bmjopen.bmj.com/site/about/guidelines.xhtml

Site ___ ___ ___ Subarea ___ ___ ___ House number ___ ___ ___ ___ Interviewer ID ___ ___

HOUSEHOLD MEMBERS

7 How many household members are living in this house in total (at the day of visit)**?**
Instruction: Recorded as a 2-digit number.
Total number of household members: ____ ____

8 Record age and sex of each household member.
Instructions: Record the respondent's initials, age and sex at the first position/row of the table. Initials, age and sex of all other household members must be recorded subsequently. The initials of a household member consist of the first three letters of the given name and the first three letters of the family name (a potential middle name is not part of the initials). Record the age of each household member as a 3-digit number (unit: year); if a household member's age is ≤12 months, record the age as 1 year. Please complete an additional form if a household has >20 members.

**Household member identifier**	**Initials** [given name/family name]	**Age** [year]	**Sex**
1/Respondent	____ ____ ____/____ ____ ____	____ ____ ____	O1=Male O2=Female
2	____ ____ ____/____ ____ ____	____ ____ ____	O1=Male O2=Female
3	____ ____ ____/____ ____ ____	____ ____ ____	O1=Male O2=Female
4	____ ____ ____/____ ____ ____	____ ____ ____	O1=Male O2=Female
5	____ ____ ____/____ ____ ____	____ ____ ____	O1=Male O2=Female
6	____ ____ ____/____ ____ ____	____ ____ ____	O1=Male O2=Female
7	____ ____ ____/____ ____ ____	____ ____ ____	O1=Male O2=Female
8	____ ____ ____/____ ____ ____	____ ____ ____	O1=Male O2=Female
9	____ ____ ____/____ ____ ____	____ ____ ____	O1=Male O2=Female
10	____ ____ ____/____ ____ ____	____ ____ ____	O1=Male O2=Female
11	____ ____ ____/____ ____ ____	____ ____ ____	O1=Male O2=Female
12	____ ____ ____/____ ____ ____	____ ____ ____	O1=Male O2=Female
13	____ ____ ____/____ ____ ____	____ ____ ____	O1=Male O2=Female
14	____ ____ ____/____ ____ ____	____ ____ ____	O1=Male O2=Female
15	____ ____ ____/____ ____ ____	____ ____ ____	O1=Male O2=Female
16	____ ____ ____/____ ____ ____	____ ____ ____	O1=Male O2=Female
17	____ ____ ____/____ ____ ____	____ ____ ____	O1=Male O2=Female
18	____ ____ ____/____ ____ ____	____ ____ ____	O1=Male O2=Female
19	____ ____ ____/____ ____ ____	____ ____ ____	O1=Male O2=Female
20	____ ____ ____/____ ____ ____	____ ____ ____	O1=Male O2=Female

For peer review only

For peer review only - http://bmjopen.bmj.com/site/about/guidelines.xhtml

Site ___ ___ ___ Subarea ___ ___ ___ House number ___ ___ ___ ___ Interviewer ID ___ ___

FORM 3: GENERAL HOUSEHOLD INFORMATION
PART B – SOCIO-ECONOMIC DATA

EDUCATION

1 Can the head of household read in [add the official language of the study site/country]**?**
O 1=Yes O 2=No O 99=Don't know O 98=No response

2 Can the head of household write in [add the official language of the study site/country]**?**
O 1=Yes O 2=No O 99=Don't know O 98=No response

3 What is the highest completed level of formal school education of the head of household?
O 1=Primary school O 4=Higher than secondary school O 99=Don't know
O 2=Middle school O 5=NO EDUCATION O 98=No response
O 3=Secondary school O 6=Other, specify ___________

OCCUPATION/INCOME

4 What is the main occupation of the household head?
Instructions: Record the status of the day of visit only.

O 1=Group 1: Managers
(chief executives, senior officials, legislators; administrative and commercial managers; production and specialized services managers; hospitality, retail and other services managers)

O 2=Group 2: Professional
(science and engineering professionals; health professionals; teaching professionals; business and administration professionals; information and communications technology professionals; legal, social and cultural professionals)

O 3=Group 3: Technicians and Associate Professionals
(science and engineering associate professionals; health associate professionals; business and administration associate professionals; legal, social, cultural and related associate professionals; information and communications technicians)

O 4=Group 4: Clerical Support Workers
(general and keyboard clerks; customer services clerks; numerical and material recording clerks; other clerical support workers)

O 5=Group 5: Services and Sales Workers
(personal services workers; sales workers; personal care workers; protective services workers)

O 6=Group 6: Skilled Agricultural, Forestry, and Fishery Workers
(market-oriented skilled agricultural workers; animal producers; marked-oriented skilled forestry, fishery and hunting workers; subsistence farmers, fishers, hunters and gatherers)

O 7=Group 7: Craft and Related Trades Workers
(building and related trades workers (excluding electricians); metal, machinery and related trades workers; handicraft and printing workers; electrical and electronics trades workers; food processing, woodworking, garment and other craft and related trades workers)

O 8=Group 8: Plant and Machine Operators, and Assemblers
(stationary plant and machine operators; assemblers; drivers and mobile plant operators)

O 9=Group 9: Elementary Occupations
(cleaners and helpers; agricultural, forestry and fishery labourers; labourers in mining, construction, manufacturing and transport; food preparation assistants; street and related sales and services workers; refuse workers and other elementary workers)

O 10=Group 0: Armed Forces Occupations
(commissioned armed forces officers; non-commissioned armed forces officers; armed forces occupations, other ranks)

O 11=Other, specify __________

O 12=NO OCCUPATION O 99=Don't' know O 98=No response

For peer review only - http://bmjopen.bmj.com/site/about/guidelines.xhtml

Site ___ ___ ___ Subarea ___ ___ ___ House number ___ ___ ___ ___ Interviewer ID ___ ___

5 What is the average monthly income of the household head based on the occupation stated in question 4 and of other household members?
Instruction: Tick one answer per household head/enter one code per household member if applicable/if income available.

Head of household	Member 1	Member 2	Member 3
Code ____________	Code ____________	Code ____________	Code ____________

[1] <100USD [3] ≥150 to <200USD [5] ≥250USD [99] Don't know
[2] ≥100 to <150USD [4] ≥200 to <250USD [6] Head of household NO INCOME (see Q4) [98] No response

6 Which and how much other/additional source(s) of monthly monetary earnings has this household?
Instruction: Tick all that applies; more than one answer is possible.

Other/additional sources	Overall monthly monetary earnings
O 1=Social grants<br>O 2=Insurance (i.e. private, public)<br>O 3=Loan (i.e. from friends, family, bank)<br>O 4=Selling (i.e. livestock, crops, furniture, other goods)<br>O 5=Personal savings<br>O 6=Other, specify ________________	O 1=<100 USD<br>O 2=≥100 to <150 USD<br>O 3=≥150 to <200 USD<br>O 4=≥200 to <250 USD<br>O 5=≥250 USD
O 7=NO OTHER/ADDITIONAL SOURCES<br>O 99=Don't know<br>O 98=No response	

HOUSING/DWELLING

7 Is the house owned by the members of this household?
O 1=Yes O 3=No, supplied (i.e. government, employer) O 99=Don't know
O 2=No, rented O 4=No, other, specify _________ O 98=No response

8 How many room(s)/individual house structure(s) of this household are used for sleeping, living, cooking and bathing?
Instruction: Enter one code per category only.

Bedroom	Living room	Kitchen	Bathroom
Code ____________	Code ____________	Code ____________	Code ____________

[1] 1-2 [3] >4 [99] Don't know
[2] 3-4 [4] None [98] No response

9 What is the construction material of the floor, the walls and the roof of room(s)/individual house structure(s) of this house?
Instruction: Enter all codes that applies per category; more than one answer per category is possible.

Floor	Walls	Roof
Code ____________	Code ____________	Code ____________

[1] Brick [3] Tiles [5] Wood [7] Metal/Tin [99] Don't know
[2] Cement/Concrete [4] Mud [6] Straw/Leaves [8] Other, specify _________ [98] No response

10 Do members of this household have access to/utilize electricity in this household?
O 1=Yes O 2=No O 99=Don't know O 98=No response

11 Which main source of light have members of this household access to/are utilizing?
O 1=Electric light/Light bulb(s) O 4=Battery-powered lamp(s) O 7=Other, specify _________
O 2=Solar light/Solar lamp(s) O 5=Candle(s) O 99=Don't know
O 3=Paraffin lamp(s)/Oil lamp(s) O 6=NO SOURCE OF LIGHT O 98=No response

For peer review only - http://bmjopen.bmj.com/site/about/guidelines.xhtml

Site ___ ___ ___ Subarea ___ ___ ___ House number ___ ___ ___ ___ Interviewer ID ___ ___

HOUSEHOLD ASSETS

12 Which of the following items do members of this household own?

Instructions: Tick all that applies; more than one answer is possible; record the quantity of functional items where applicable.

O 1=Radio	Quantity ____	O None	O 11=Tape/CD player	Quantity ____	O None
O 2=Television	Quantity ____	O None	O 12=Computer/Laptop	Quantity ____	O None
O 3=Internet connection	Quantity ____	O None	O 13=Mobile/Cell phone	Quantity ____	O None
O 4=Car	Quantity ____	O None	O 14=Truck/Bus	Quantity ____	O None
O 5=Motorbike	Quantity ____	O None	O 15=Bicycle	Quantity ____	O None
O 6=Oxcart/Donkey	Quantity ____	O None	O 16=Bed	Quantity ____	O None
O 7=Mattress	Quantity ____	O None	O 17=Bed net	Quantity ____	O None
O 8=Table	Quantity ____	O None	O 18=Chair	Quantity ____	O None
O 9=Refrigerator (4°C)	Quantity ____	O None	O 19=Freezer (-20°C)	Quantity ____	O None
O 10=Fan	Quantity ____	O None	O 20=Sewing machine	Quantity ____	O None

For peer review only

Site ___ ___ ___ Subarea ___ ___ ___ House number ___ ___ ___ ___ Interviewer ID ___ ___

FORM 3: GENERAL HOUSEHOLD INFORMATION
Part C – Hygiene and sanitation

TOILET FACILITY

1 To what type of toilet facility have members of this household usually access?
Instruction: Inside means inside a room/individual house structure/building; outside means outside a room/individual house structure/building.

O 1=Flush toilet/Water closet (inside)
O 2=Flush toilet/Water closet (outside)
O 3=Non-flush latrine/pit latrine (inside)
O 4=Non-flush latrine/pit latrine (outside)
O 5=Ventilated Improved Pit Latrine (KVIP, inside)
O 6=Ventilated Improved Pit Latrine (KVIP, outside)
O 7=Composting toilet (inside)
O 8=Composting toilet (outside)
O 9=NO toilet facility, use a bucket
O 10=NO toilet facility, dig a hole
O 11=NO toilet facility, free range/open defecation
O 12=Other, specify __________
O 99=Don't know
O 98=No response

2 Do members of this household usually utilize the toilet facility stated in question 1?

O 1=No, members cannot utilize the facility independently (i.e. infants, toddlers)
O 2=No, members cannot utilize the facility independently (i.e. disabled, long-term/chronically diseased)
O 3=No, members cannot utilize the facility independently (i.e. seniors)
O 4=No, limited access (i.e. public, shared with other households/community)
O 5=No, too far away (i.e. public, shared with other households/community)
O 6=No, bad condition (i.e. broken, not clean)
O 7=YES
O 8=Other, specify _________________
O 99=Don't know
O 98=No response

3 Where is the toilet facility stated in question 1 located? With how many other people is it usually shared?

O 1=On the property of this household (private, not shared)
O 2=On the property of a neighbouring household (private, shared)

Shared O 1=<10 people O 2=11-24 people O 3=25-49 people O 4=>50 people O 99=Don't know O 98=No response

O 3=In the community (public, shared)

Shared O 1=<10 people O 2=11-24 people O 3=25-49 people O 4=>50 people O 99=Don't know O 98=No response

O 4=Other, specify __________ O 99=Don't know O 98=No response

4 How do members of this household that have/don't have access to a toilet facility usually clean their hands directly before and after urination/defection?
Instruction: Enter one code per category only.

Clean hands before urination/defection	Clean hands after urination/defection
Code _____________	Code _____________
[1] Wash hands with soap and water	[6] Don't clean, not needed/not dirty
[2] Wash hands with water only	[7] Don't clean, nothing to clean hands nearby/on hands
[3] Rub hands clean with some leaves/straw/ grass	[8] Other, specify __________
[4] Rub hands clean with some sand	[99] Don't know
[5] Rub hands clean with some cloth/fabric	[98] No response

KITCHEN/FOOD HANDLING

5 Do members of this household have a kitchen for cooking/preparing food/meals? Where is it located?

O 1=Yes, inside kitchen/in a room/individual house structure; on the property of this household (private, not shared)
O 2=Yes, outside kitchen/outside a room/individual house structure/ open air; on the property of this

For peer review only - http://bmjopen.bmj.com/site/about/guidelines.xhtml

Site ___ ___ ___ Subarea ___ ___ ___ House number ___ ___ ___ ___ Interviewer ID ___ ___

household (private, not shared)
- O 3=Yes, inside kitchen/in a room/individual house structure; on the property of a neighbouring household (private, shared)
- O 4=Yes, outside kitchen/outside a room/individual house structure/ open air; on the property of a neighbouring household (private, shared)
- O 5=NO KITCHEN
- O 6=Other, specify __________ O 99=Don't know O98=No response

6 What energy source do members of this household usually use for cooking/preparing food/meals?

O 1=Electricity O 3=Charcoal O 5=Animal dung O 99=Don't know
O 2=Gas/Kerosene O 4=Wood/straw/leaves O 6=Other, specify _____ O 98=No response

7 What cooking spot do members of this household usually use for cooking/preparing food/meals?

O 1=Open fire O 3=Closed stove with chimney O 99=Don't know
O 2=Open stove O 4=Other, specify __________ O 98=No response

8 Where do members of this household usually store fresh/raw food items (e.g. vegetables, lettuce, fruits, dairy products, eggs, meat and fish) and left-over/prepared food/meals?

Instruction: Enter one code per category only. Inside means inside a room/individual house structure/building; outside means outside a room/individual house structure/building.

Fresh/raw food items (i.e. vegetables, lettuce, fruits, dairy products, eggs, meat and fish)	Left-over/prepared food/meals
Code _____________	Code _____________

[1] At room temperature (inside, on the property of this household, private)
[2] At room temperature (outside, on the property of this household, private)
[3] At room temperature (inside, on the property of a neighboring household, private, shared)
[4] At room temperature (outside, on the property of a neighboring household, private, shared)
[5] Refrigerator (on the property of this household, private)
[6] Refrigerator (on the property of a neighboring household, private, shared)
[7] Freezer (on the property of this household, private)
[8] Freezer (on the property of a neighboring household, private, shared)
[9] Other, specify ______________
[10] NO STORAGE [99] Don't know [98] No response

9 If the answer is "Room temperature" for fresh/raw food items and/or left-over/ prepared food/meals in question 8, how long do members of this household usually store them?

Instruction: Enter one code per category only.

Fresh/raw food items (i.e. vegetables, lettuce, fruits, dairy products, eggs, meat and fish)	Left-over/prepared food/meals
Code _____________	Code _____________

[1] <1 day [3] 3-4 days [5] Not applicable [99] Don't know
[2] 1-2 days [4] >4 days [98] No response

10 Do members of this household usually cover left-over/prepared food/meals? Do they re-heat/re-cook left-over/prepared food/meals prior to consumption?

Instruction: Enter one code per category only.

Cover left-over/prepared food/meals	Re-heat/Re-cook left-over/prepared food/meals
Code _____________	Code _____________

[1] Yes [2] No [99] Don't know [98] No response

For peer review only - http://bmjopen.bmj.com/site/about/guidelines.xhtml

Site ___ ___ ___ Subarea ___ ___ ___ House number ___ ___ ___ ___ Interviewer ID ___ ___

11 Which member(s) of this household usually cook(s)/prepare(s) food/meals for other members of this household?
Instructions: Refer to question 9 of "Form 3 Part A – Demographic data/General household information" and record the initials/identifier(s) of the member.
O Household member, record initials/identifier(s) __________ O 99=Don't know O 98=No response

12 How does/do the household member(s) that usually cook(s)/prepare(s) food/meals for other members of this household as stated in question 11 usually prepare(s) herself/himself/themselves before and after the preparation of meals?
Instruction: Enter one code per category only.

Before the preparation of meals	After the preparation of meals
Code _____________	Code _____________
[1] Wash hands with soap and water<br>[2] Wash hands with water only<br>[3] Rub hands clean with some leaves/straw/ grass<br>[4] Rub hands clean with some sand<br>[5] Rub hands clean with some cloth/fabric	[6] Don't clean, not needed/not dirty<br>[7] Don't clean, nothing to clean hands nearby/on hands<br>[8] Other, specify __________<br>[99] Don't know<br>[98] No response

13 How do members of this household usually prepare themselves before and after eating food/meals, including the household member that usually cooks/prepares food/meals as stated in question 11?
Instruction: Enter one code per category only.

Before the eating of food/meals	After the eating of food/meals
Code _____________	Code _____________
[1] Wash hands with soap and water<br>[2] Wash hands with water only<br>[3] Rub hands clean with some leaves/straw/ grass<br>[4] Rub hands clean with some sand<br>[5] Rub hands clean with some cloth/fabric	[6] Don't clean, not needed/not dirty<br>[7] Don't clean, nothing to clean hands nearby/on hands<br>[8] Other, specify __________<br>[99] Don't know<br>[98] No response

14 How do members of this household usually eat their food/meals, including the household member that usually cooks/prepares food/meals as stated in question 11?
O 1=With cutlery O 3=Other, specify _____________ O 99=Don't know
O 2=With fingers O 98=No response

15 Do members of this household usually eat their food/meals from the same pot, including the household member that usually cooks/prepares food/meals as stated in question 11?
O 1=Yes O 2=No O 99=Don't know O 98=No response

16 Where do members of this household usually get their main fresh/raw food items such as vegetables, fruits and grains/cereals, dairy products, eggs, meat and fish?
Instruction: Enter one code per category only.

Vegetables, fruits, grains/ cereals	Dairy products	Eggs, meat, fish
Code _____________	Code _____________	Code _____________
[1] Self-farming (i.e. back-/courtyard)<br>[2] Supermarket/Grocery store<br>[3] Friend/Family<br>[4] Open market<br>[5] Street vendor	[6] Self-farming (i.e. agricultural land)<br>[7] Butcher<br>[8] Other, specify _____________<br>[99] Don't know<br>[98] No response	

17 Do members of this household usually consume/eat fresh/raw food items (e.g. vegetables, lettuce, fruits, dairy products, ice cream, eggs, meat and fish)? If so, which ones?

For peer review only - http://bmjopen.bmj.com/site/about/guidelines.xhtml

Site ___ ___ ___ Subarea ___ ___ ___ House number ___ ___ ___ ___ Interviewer ID ___ ___

Instruction: Tick all that applies; more than one answer is possible.

- O 1=Vegetables/Lettuce (cultivated on bush)
- O 2=Vegetables/Lettuce (wild harvesting from bush)
- O 3=Fruits (cultivated on bush/tree)
- O 4=Fruits (wild harvesting from bush/tree)
- O 5=Dairy products (i.e. milk, cheese, yoghurt from domesticated milk producing animals), specify ____________
- O 6=Eggs (from domesticated poultry), specify _________
- O 7=Animal blood (from domesticated animals), specify _________
- O 8=Beef/Zebu/Buffalo (domesticated), specify _________
- O 9=Pork (domesticated)
- O 10=Sheep (domesticated)
- O 11=Meat from non-domesticated animals (i.e. antelope, boar, poultry, rodents, fish), specify _________
- O 12=NO CONSUMPTION of fresh/raw food items
- O 13=Vegetables/Lettuce (cultivated on ground)
- O 14=Vegetables/Lettuce (wild harvesting from ground)
- O 15=Fruits (cultivated on ground)
- O 16=Fruits (wild harvesting from ground)
- O 17=Dairy products (i.e. milk, cheese, yoghurt from non-domesticated milk producing animals), specify ____________
- O 18=Eggs (from non-domesticated poultry), specify _________
- O 19=Animal blood (from non-domesticated animals), specify _________
- O 20=Poultry (domesticated; i.e. chicken, duck, pigeon, goose, turkey), specify _________
- O 21=Goat (domesticated)
- O 22=Rodents (domesticated), specify _________
- O 23=Fish/Seafood, specify _________
- O 24=Ice cream
- O 25=Other, specify _________
- O 99=Don't know O 98=No response

18 Do members of this household consume meals/food items/beverages usually also outside the house? If so, which member(s) of this household and where?

Instructions: Refer to question 9 of "Form 3 Part A – Demographic data/General household information" and record the initials/identifier of the member(s). Enter all codes that apply for the respective household member.

Member 1<br>Initials/Identifier: _____	Member 2<br>Initials/Identifier: _____	Member 3<br>Initials/Identifier: _____	Member 4<br>Initials/Identifier: _____
Code ____________	Code ____________	Code ____________	Code ____________
[1] Restaurant<br>[2] Fast food chain	[3] Cafeteria<br>[4] Street vendor	[5] Other, specify ____<br>[6] Not applicable	[99] Don't know<br>[98] No response

19 Which meals/food items/beverages do the member(s) of this household listed in question 18 usually consume outside the house?

Instructions: Refer to question 9 of "Form 3 Part A – Demographic data/General household information" and record the initials/identifier of the member(s). List up to 5 meals/food items/beverages per household member. You can refer to food items listed in question 17. Skip this question of no household member consumes meals/food items/beverages outside the house as stated in Q18.

Member 1<br>Initials/Identifier: _____	Member 2<br>Initials/Identifier: _____	Member 3<br>Initials/Identifier: _____	Member 4<br>Initials/Identifier: _____
1: ______________<br>2: ______________<br>3: ______________<br>4: ______________<br>5: ______________	1: ______________<br>2: ______________<br>3: ______________<br>4: ______________<br>5: ______________	1: ______________<br>2: ______________<br>3: ______________<br>4: ______________<br>5: ______________	1: ______________<br>2: ______________<br>3: ______________<br>4: ______________<br>5: ______________

WATER

20 What is the main water supply for members of this household for drinking, cooking/ preparing food/meals, bathing, and cleaning (of house/individual house structure(s), clothing/kitchen tools)?

Instruction: Enter one code per category only.

Drinking	Cooking	Bathing	Cleaning
Code ____________	Code ____________	Code ____________	Code ____________
[1] Tap water (inside the house; private)<br>[2] Tap water (outside the house i.e. back-/courtyard; private)			

For peer review only - http://bmjopen.bmj.com/site/about/guidelines.xhtml

Site ___ ___ ___ Subarea ___ ___ ___ House number ___ ___ ___ ___ Interviewer ID ___ ___

[3] Tap water (outside the house; public/shared)
[4] Standpipe/Piped water (inside the house; private)
[5] Standpipe/Piped water (outside the house i.e. back-/ courtyard; private)
[6] Standpipe/Piped water (outside the house; public/shared)
[7] Water truck (public; shared)
[8] Surface water (i.e. canal, irrigation canal)
[9] Surface water (i.e. lake, pond, river, stream)
[10] Spring water
[11] Rain water
[12] Well/Borehole (open/uncovered/unprotected)
[13] Well/Borehole (covered/protected)
[14] Bottled water
[15] Other, specify ________________ [99] Don't know [98] No response

21 Do members of this household usually treat water for drinking, cooking/ preparing food/meals, bathing, and cleaning (of house/individual house structure(s), clothing/ kitchen tools) prior to consumption? How is water usually treated?

Instruction: Enter all codes that applies per category; more than one answer per category is possible.

Drinking	Cooking	Bathing	Cleaning
Code ____________	Code ____________	Code ____________	Code ____________

[1] No, not at all
[2] No, only for sick members of this household
[3] No, only depending on the season (dry season)
[4] Yes, filtration (i.e. cloth, ceramic, sand)
[5] Yes, sedimentation
[6] Other, specify ________________
[7] No, only for children of this household
[8] No, only depending on the season (rainy season)
[9] Yes, boiling
[10] Yes, solar disinfection
[11] Yes, chlorination
[99] Don't know [98] No response

22 Which member(s) of this household usually collect(s) the drinking water as stated in question 20 for other members of this household?

Instructions: Refer to question 9 of "Form 3 Part A – Demographic data/General household information" and record the initials/identifier of the member.

O Household member, record initials/identifier(s) __________ O 99=Don't know O 98=No response

23 How do members of this household usually store the drinking water stated in question 20?

O 1=Water tank (uncovered)
O 2=Water tank (covered with i.e. cloth/fabric, lid, leaves)
O 3=Container (uncovered)
O 4=Container (covered with i.e. cloth/fabric, lid, leaves)
O 5=Clay pot (uncovered)
O 6=Clay pot (covered with i.e. cloth/fabric, lid, leaves)
O 7=Bucket (uncovered)
O 8=Bucket (covered with i.e. cloth/fabric, lid, leaves)
O 9=Bowl (uncovered)
O 10=Bowl (covered with i.e. cloth/fabric, lid, leaves)
O 11=Wooden pot (uncovered)
O 12=Wooden pot (covered with i.e. cloth/fabric, lid, leaves)
O 13=Skin vessel (i.e. goat, sheep, cattle; uncovered)
O 14=Skin vessel (i.e. goat, sheep, cattle; covered with i.e. cloth/fabric, lid, leaves)
O 15=NOT STORED
O 16=Other, specify __________
O 99=Don't know
O 98=No response

24 If members of this household use a shared drinking water supply as stated in question 20, with how many other people is this drinking water supply usually shared?

O 1=<10 people
O 2=11-24 people
O 3=25-49 people
O 4=>50 people
O 5=NOT SHARED
O 99=Don't know
O 98=No response

For peer review only - http://bmjopen.bmj.com/site/about/guidelines.xhtml

Site ___ ___ ___ Subarea ___ ___ ___ House number ___ ___ ___ ___ Interviewer ID ___ ___

WASTE DISPOSAL

25 Where do members of this household usually dispose waste water from e.g. cooking/ preparation of food/meals, bathing and/or cleaning, dispose domestic waste, human waste/excreta?

Instruction: Enter one code per category only.

Waste water from (e.g. cooking/ preparation of food/ meals, bathing and/or cleaning)	Domestic waste	Human waste/excreta
Code ____________	Code ____________	Code ____________

[1] On the ground/free range near the house (i.e. back-/courtyard, private)
[2] On the ground/free range away from the house in the community (shared, public)
[3] In a hole near the house (i.e. back-/courtyard, private)
[4] In a hole away from the house in the community (shared, public)
[5] Pour in surface water in the community (i.e. lake, pond, river, stream, public, shared)
[6] Pour in surface water in the community (i.e. canal, irrigation canal, public, shared)
[7] Pour in spring water in the community (public, shared)
[8] Pour in a septic tank near the house (i.e. back-/courtyard, near toilet facility if available, private)
[9] Pour in a septic tank in the community (shared, public)
[10] Pour in the sewer near the house (i.e. back-/courtyard, private)
[11] Pour in the sewer in the community (shared, public)
[12] Other, specify ________________________ [99] Don't know [98] No response

26 Do members of this household usually pre-process domestic waste from this house prior to disposing it?

- O 1=Yes, burning garbage
- O 2=Yes, separate/sort out degradable from non-degradable garbage
- O 3=NO
- O 4=Yes, other, specify _________
- O 99=Don't know
- O 98=No response

27 How far from the main drinking water supply of this household as stated in question 20 is the location for disposal of waste water, domestic waste and human waste/excreta of this household as stated in question 25?

Instructions: Enter one code per category only. Record the distance as meter [m].

Waste water from (e.g. cooking/ preparation of food/ meals, bathing and/or cleaning)	Domestic waste	Human waste/excreta
Code ____________	Code ____________	Code ____________

[1] <5 m [3] ≥25 to <50 m [5] ≥100 to <500 m [7] ≥1,000 m
[2] ≥5 to <25 m [4] ≥50 to <100 m [6] ≥500 to <1,000 m [99] Don't know [98] No response

28 How far from the main water supply for cooking/preparing food/meals of this household as stated in question 20 is the location for disposal of waste water, domestic waste and human waste/excreta of this household as stated in question 25?

Instructions: Enter one code per category only. Record the distance as meter [m].

Waste water from (e.g. cooking/preparation of food/ meals, bathing and/or cleaning)	Domestic waste	Human waste/excreta
Code ____________	Code ____________	Code ____________

[1] <5 m [3] ≥25 to <50 m [5] ≥100 to <500 m [7] ≥1,000 m
[2] ≥5 to <25 m [4] ≥50 to <100 m [6] ≥500 to <1,000 m [99] Don't know [98] No response

FARM/DOMESTIC ANIMALS

29 What kind of farm animals/livestock and domestic animals/pets do members of this household have?

Instruction: Tick all that applies per category; more than one answer per category is possible.

For peer review only - http://bmjopen.bmj.com/site/about/guidelines.xhtml

Site ___ ___ ___ Subarea ___ ___ ___ House number ___ ___ ___ ___ Interviewer ID ___ ___

Farm animals/livestock		Domestic animals/pets
O 1=Cattle	O 9=Camel	O 1=Dog
O 2=Sheep	O 10= Rodents (i.e. rabbit), specify ______________	O 2=Rodents (i.e. rabbit), specify ______________
O 3=Donkey	O 11=Goat	O 3=Cat
O 4=Poultry (i.e. chicken, duck, pigeon, goose, turkey), specify ______________	O 12=Horse	O 4=NO DOMESTIC ANIMALS/ PETS
O 5=Zebu	O 13=NO FARM ANIMALS/ LIVESTOCK	O 5=Other, specify ______________
O 6=Pig	O 14=Other, specify ______________	O 99=Don't know
O 7=Fish	O 99=Don't know	O 98=No response
O 8=Buffalo	O 98=No response	

30 Where do members of this household usually keep farm animals/livestock and domestic animals/pets of this household as stated in question 29?
Instruction: Tick one answer per category only.

Farm animals/livestock	Domestic animals/pets
O 1=Stable/Cage/Water basin on the property of the house (i.e. back-/courtyard)<br>O 2=Stable/Cage/Water basin outside the house in the community<br>O 3=Stable/Cage/Water basin outside the community<br>O 4=Free range/Surface water/Spring water on the property of the house (i.e. house, back-/courtyard)<br>O 5=Free range/Surface water/Spring water outside the house in the community<br>O 6=Free range/Surface water/Spring water outside the community<br>O 7=Not applicable (nowhere/no farm animals/livestock)<br>O 8=Other, specify __________<br>O 99=Don't know<br>O 98=No response	O 1=Cage/Chain near the house (i.e. back-/courtyard)<br>O 2=Cage/Chain outside the house in the community<br>O 3=Cage/Chain outside the community (public)<br>O 4=Free range near the house (i.e. in the house, back-/courtyard)<br>O 5=Free range outside the house in the community<br>O 6=Free range outside the community<br>O 7=Not applicable (nowhere/no domestic animals/pets)<br>O 8=Other, specify __________<br>O 99=Don't know<br>O 98=No response

31 What is the extent of the livestock farming that members of this household practice?

O 1=Own demand ("backyard farming")
O 2=Not applicable (no livestock farming)
O 3=Mass/Industrial demand ("mass animal farming")
O 4=Other, specify __________
O 99=Don't know
O 98=No response

32 Which water supply do members of this household usually use to water farm animals/livestock and domestic animals/pets of this household as stated in question 29?
Instruction: Enter one code per category only.

Farm animals/livestock	Domestic animals/pets
Code ______________	Code ______________

[1] Tap water (inside the house; private)
[2] Tap water (outside the house i.e. back-/courtyard; private)
[3] Tap water (outside the house; public/shared)
[4] Standpipe/Piped water (inside the house; private)
[5] Standpipe/Piped water (outside the house i.e. back-/ courtyard; private)
[6] Standpipe/Piped water (outside the house; public/shared)

For peer review only - http://bmjopen.bmj.com/site/about/guidelines.xhtml

Site ___ ___ ___ Subarea ___ ___ ___ House number ___ ___ ___ ___ Interviewer ID ___ ___

[7] Water truck (public; shared)
[8] Surface water (i.e. canal, irrigation canal)
[9] Surface water (i.e. lake, pond, river, stream)
[10] Spring water
[11] Rain water
[12] Well/Borehole (open/uncovered/unprotected)
[13] Well/Borehole (covered/protected)
[14] Not applicable (no farm animals/livestock; no domestic animals/pets)
[15] Other, specify ____________________ [99] Don't know [98] No response

33 Do members of this household usually wash their hands after they fed & watered and cleaned the stable/cage/water basin of the farm animals/livestock and domestic animals/pets of this household as stated in question 29?
Instruction: Enter one code per category only.

Farm animals/livestock	Domestic animals/pets
Code ____________	Code ____________

[1] Wash hands with soap and water
[2] Wash hands with water only
[3] Rub hands clean with some leaves/straw/ grass
[4] Rub hands clean with some sand
[5] Rub hands clean with some cloth/fabric
[6] Don't clean, not needed/not dirty
[7] Don't clean, nothing to clean hands nearby/on hands
[8] Other, specify _________
[9] Not applicable (no farm animals/livestock; no domestic animals/pets)
[99] Don't know
[98] No response

CROP FARMING

34 What kind of crops do members of this household grow/cultivate?
Instruction: Tick all that applies; more than one answer is possible.

O 1=Coffee
O 2=Rice
O 3=Fruits (cultivated on bush/tree)
O 4=Fruits (cultivated on the ground)
O 5=Vegetables/Lettuce (cultivated on bush/tree)
O 6=Vegetables/Lettuce (cultivated on the ground)
O 7=Grains (i.e. wheat, barley, oat, corn)
O 8=Tea
O 9=Cashew
O 10=Tobacco
O 11=Sugar cane
O 12=Chad/Chat
O 13=Bees/Honey
O 14=Cotton
O 15=NO CROPS
O 16=Other, specify __________
O 99=Don't know
O 98=No response

35 Where do members of this household have the farmland/agricultural land for crop farming?

O 1=On the property of the house (i.e. back-/courtyard)
O 4=Outside the house in the community
O 2=Outside the community
O 5=Not applicable (no crop farming)
O 3=Other, specify __________
O 99=Don't know
O 98=No response

36 What is the extent of the crop farming that members of this household practice?

O 1=Own demand ("backyard farming")
O 2=Not applicable (no crop farming)
O 3=Mass/Industrial demand ("mass crop farming")
O 4=Other, specify __________
O 99=Don't know
O 98=No response

37 Do members of this household usually use herbicides, pesticides and fertilizers for the crop farming as stated in question 34?
Instruction: Tick one answer per category only.

Herbicides	Pesticides	Fertilizers
O 1=Yes, specify herbicide ____________________	O 1=Yes, specify pesticide ____________________	O 1=Yes, specify fertilizer ____________________

For peer review only - http://bmjopen.bmj.com/site/about/guidelines.xhtml

Site ___ ___ ___ Subarea ___ ___ ___ House number ___ ___ ___ ___ Interviewer ID ___ ___

O 2=Not applicable (no crop farming)<br>O 3=No use of herbicides<br>O 99=Don't know<br>O 98=No response	O 2=Not applicable (no crop farming)<br>O 3=No use of pesticides<br>O 99=Don't know<br>O 98=No response	O 2=Not applicable (no crop farming)<br>O 3=No use of fertilizers<br>O 99=Don't know<br>O 98=No response

38 Do members of this household usually wash their hands after the fieldwork for the crop farming as stated in question 34?

- O 1=Yes, wash hands with soap and water
- O 2=Yes, rub hands clean with some leaves/straw/grass
- O 3=Yes, rub hands clean with some cloth/fabric
- O 4=Not applicable (no crop farming)
- O 5=Yes, wash hands with water only
- O 6=Yes, rub hands clean with some sand
- O 7=No, don't clean my hands
- O 8=Other, specify __________
- O 99=Don't know
- O 98=No response

For peer review only

For peer review only - http://bmjopen.bmj.com/site/about/guidelines.xhtml

Site ___ ___ ___ Subarea ___ ___ ___ House number ___ ___ ___ ___ Interviewer ID ___ ___

FORM 4: ASSESSMENT OF OVERALL, AGE- AND SEX-STRATIFIED GENERIC HEALTHCARE UTILIZATION
Part A – Assessment of *generic* healthcare utilization

Instructions to the interviewer

- Complete this form only if the respondent consented to participate (see "Form 2 - CONSENT FORM"); the respondent as defined for this investigation is an adult (at the country-specific legal age of majority) household member and decision-maker with respect to daily healthcare/healthcare utilization for the entire household and its members; it must not be e.g. a neighbour or a visitor.
- Refer to "Form 3 – Part A - GENERAL HOUSEHOLD INFORMATION" as a reference to obtain the total number of household members as well as age and sex of each household member.
- Collect data about all household members regardless of whether all household members are present at the time of the interview; collecting and recording data only about individuals present at the time of the interview will result in biased data.
- A household is/household members are defined as a person or a group of related or unrelated persons that live together in the same dwelling unit, that acknowledge one male or female adult as the head of the household, that share the same housekeeping arrangements, that are considered to constitute one unit, and that provide themselves with food or other essentials for living.
- "Site" and "Subarea" must be entered each as a 3-digit, "House number" as a 4-digit, and "Interviewer ID" as a 2-digit code (use lists individually prepared for each site to obtain codes). "Study label" must be a combination of a 3-digit "Site" code, a 3-digit "Subarea" code and a 4-digit "House number" code; the "Interviewer ID" must be entered as a 3-digit code.
- Abbreviation: HCF=private/public primary/secondary/tertiary healthcare facility.

HOUSEHOLD MEMBERS

1 Record the total number of household members of this house (at the day of visit)**.**
Instructions: The total number of household members must be recorded as a 2-digit number; see also "Form 3 – Part A - GENERAL HOUSEHOLD INFORMATION".

Total number of household members: ____ ____

2 Record the number of FEMALE household members by each of the following age groups:
Instructions: The total number of female household members by age group must be recorded as a 2-digit number; see also "Form 3 – Part A - GENERAL HOUSEHOLD INFORMATION".

<2years: ____ ____ ≥2 to <5years: ____ ____ ≥5 to <15years: ____ ____ ≥15years: ____ ____

3 Record the number of MALE household members by each of the following age groups:
Instructions: The total number of male household members by age group must be recorded as a 2-digit number; see also "Form 3 – Part A - GENERAL HOUSEHOLD INFORMATION".

<2years: ____ ____ ≥2 to <5years: ____ ____ ≥5 to <15years: ____ ____ ≥15years: ____ ____

HEALTHCARE SEEKING BEHAVIOR

4 Record where female/male household members <2years/≥2 to <5years/≥5 to <15years/≥15years of age usually seek healthcare for the following signs/symptoms. Please rate using a range from 1-4.

Instructions:
- Complete question 4 separately for female and male household members <2years, ≥2 to <5years, ≥5 to <15years and ≥15years of age and circle the sex and age group Q4 was completed for; skip question 4 if there is no female/male household member <2years/ ≥2 to <5years/ ≥5 to <15years/ ≥15years of age.
- Provide an answer to each healthcare option for each sign/symptom; use range 1-4 for rating (1=1st choice; 2=2nd choice; 3=3rd choice; 4=not chosen); enter a code where applicable by using prepared lists (use lists individually prepared for each site to obtain codes); tick "Don't know" if applicable.

For peer review only - http://bmjopen.bmj.com/site/about/guidelines.xhtml

Site ___ ___ ___ Subarea ___ ___ ___ House number ___ ___ ___ ___ Interviewer ID ___ ___

Sign/Symptom	Healthcare options									
	Study-HCF		Other HCF		Physician	Pharmacy	Traditional healer	Nowhere Self-treatment	Nowhere Nothing	Don't know
	Rate	Code	Rate	Code	Rate	Rate	Rate	Rate	Rate	Tick
**1** [**A**] Fever, any	O=1<br>O=2<br>O=3<br>O=4		O=1<br>O=2<br>O=3<br>O=4		O=1<br>O=2<br>O=3<br>O=4	O=1<br>O=2<br>O=3<br>O=4	O=1<br>O=2<br>O=3<br>O=4	O=1<br>O=2<br>O=3<br>O=4	O=1<br>O=2<br>O=3<br>O=4	O
**2** Fever <3days (continuous)	O=1<br>O=2<br>O=3<br>O=4		O=1<br>O=2<br>O=3<br>O=4		O=1<br>O=2<br>O=3<br>O=4	O=1<br>O=2<br>O=3<br>O=4	O=1<br>O=2<br>O=3<br>O=4	O=1<br>O=2<br>O=3<br>O=4	O=1<br>O=2<br>O=3<br>O=4	O
**3** Fever ≥3days (continuous)	O=1<br>O=2<br>O=3<br>O=4		O=1<br>O=2<br>O=3<br>O=4		O=1<br>O=2<br>O=3<br>O=4	O=1<br>O=2<br>O=3<br>O=4	O=1<br>O=2<br>O=3<br>O=4	O=1<br>O=2<br>O=3<br>O=4	O=1<br>O=2<br>O=3<br>O=4	O
**4** Chills Shivering	O=1<br>O=2<br>O=3<br>O=4		O=1<br>O=2<br>O=3<br>O=4		O=1<br>O=2<br>O=3<br>O=4	O=1<br>O=2<br>O=3<br>O=4	O=1<br>O=2<br>O=3<br>O=4	O=1<br>O=2<br>O=3<br>O=4	O=1<br>O=2<br>O=3<br>O=4	O
**5** Convulsion	O=1<br>O=2<br>O=3<br>O=4		O=1<br>O=2<br>O=3<br>O=4		O=1<br>O=2<br>O=3<br>O=4	O=1<br>O=2<br>O=3<br>O=4	O=1<br>O=2<br>O=3<br>O=4	O=1<br>O=2<br>O=3<br>O=4	O=1<br>O=2<br>O=3<br>O=4	O
**6** Weight loss	O=1<br>O=2<br>O=3<br>O=4		O=1<br>O=2<br>O=3<br>O=4		O=1<br>O=2<br>O=3<br>O=4	O=1<br>O=2<br>O=3<br>O=4	O=1<br>O=2<br>O=3<br>O=4	O=1<br>O=2<br>O=3<br>O=4	O=1<br>O=2<br>O=3<br>O=4	O
**7** Dehydration	O=1<br>O=2<br>O=3<br>O=4		O=1<br>O=2<br>O=3<br>O=4		O=1<br>O=2<br>O=3<br>O=4	O=1<br>O=2<br>O=3<br>O=4	O=1<br>O=2<br>O=3<br>O=4	O=1<br>O=2<br>O=3<br>O=4	O=1<br>O=2<br>O=3<br>O=4	O
**8** Malaise Weakness/ Fatigue	O=1<br>O=2<br>O=3<br>O=4		O=1<br>O=2<br>O=3<br>O=4		O=1<br>O=2<br>O=3<br>O=4	O=1<br>O=2<br>O=3<br>O=4	O=1<br>O=2<br>O=3<br>O=4	O=1<br>O=2<br>O=3<br>O=4	O=1<br>O=2<br>O=3<br>O=4	O
**9** Aches/ Pain (muscle/joint/bone)	O=1<br>O=2<br>O=3<br>O=4		O=1<br>O=2<br>O=3<br>O=4		O=1<br>O=2<br>O=3<br>O=4	O=1<br>O=2<br>O=3<br>O=4	O=1<br>O=2<br>O=3<br>O=4	O=1<br>O=2<br>O=3<br>O=4	O=1<br>O=2<br>O=3<br>O=4	O
**10** Headache	O=1<br>O=2<br>O=3<br>O=4		O=1<br>O=2<br>O=3<br>O=4		O=1<br>O=2<br>O=3<br>O=4	O=1<br>O=2<br>O=3<br>O=4	O=1<br>O=2<br>O=3<br>O=4	O=1<br>O=2<br>O=3<br>O=4	O=1<br>O=2<br>O=3<br>O=4	O
**11** Dizziness/ Confusion Unconsciousness	O=1<br>O=2<br>O=3<br>O=4		O=1<br>O=2<br>O=3<br>O=4		O=1<br>O=2<br>O=3<br>O=4	O=1<br>O=2<br>O=3<br>O=4	O=1<br>O=2<br>O=3<br>O=4	O=1<br>O=2<br>O=3<br>O=4	O=1<br>O=2<br>O=3<br>O=4	O
**12** Blood pressure disorders	O=1<br>O=2<br>O=3<br>O=4		O=1<br>O=2<br>O=3<br>O=4		O=1<br>O=2<br>O=3<br>O=4	O=1<br>O=2<br>O=3<br>O=4	O=1<br>O=2<br>O=3<br>O=4	O=1<br>O=2<br>O=3<br>O=4	O=1<br>O=2<br>O=3<br>O=4	O

For peer review only

For peer review only - http://bmjopen.bmj.com/site/about/guidelines.xhtml

Site ___ ___ ___ Subarea ___ ___ ___ House number ___ ___ ___ ___ Interviewer ID ___ ___

Sign/Symptom	Healthcare options									
	Study-HCF		Other HCF		Physician	Pharmacy	Traditional healer	Nowhere Self-treatment	Nowhere Nothing	Don't know
	Rate	Code	Rate	Code	Rate	Rate	Rate	Rate	Rate	Code
**13** Heart disorders	O=1<br>O=2<br>O=3<br>O=4		O=1<br>O=2<br>O=3<br>O=4		O=1<br>O=2<br>O=3<br>O=4	O=1<br>O=2<br>O=3<br>O=4	O=1<br>O=2<br>O=3<br>O=4	O=1<br>O=2<br>O=3<br>O=4	O=1<br>O=2<br>O=3<br>O=4	O
**14** Nausea Vomitting	O=1<br>O=2<br>O=3<br>O=4		O=1<br>O=2<br>O=3<br>O=4		O=1<br>O=2<br>O=3<br>O=4	O=1<br>O=2<br>O=3<br>O=4	O=1<br>O=2<br>O=3<br>O=4	O=1<br>O=2<br>O=3<br>O=4	O=1<br>O=2<br>O=3<br>O=4	O
**15** Diarrhea (watery/ bloody/ mucopurulent)	O=1<br>O=2<br>O=3<br>O=4		O=1<br>O=2<br>O=3<br>O=4		O=1<br>O=2<br>O=3<br>O=4	O=1<br>O=2<br>O=3<br>O=4	O=1<br>O=2<br>O=3<br>O=4	O=1<br>O=2<br>O=3<br>O=4	O=1<br>O=2<br>O=3<br>O=4	O
**16** Abdominal pain Intestinal pain	O=1<br>O=2<br>O=3<br>O=4		O=1<br>O=2<br>O=3<br>O=4		O=1<br>O=2<br>O=3<br>O=4	O=1<br>O=2<br>O=3<br>O=4	O=1<br>O=2<br>O=3<br>O=4	O=1<br>O=2<br>O=3<br>O=4	O=1<br>O=2<br>O=3<br>O=4	O
**17** Blood loss/ Bleeding (internal)	O=1<br>O=2<br>O=3<br>O=4		O=1<br>O=2<br>O=3<br>O=4		O=1<br>O=2<br>O=3<br>O=4	O=1<br>O=2<br>O=3<br>O=4	O=1<br>O=2<br>O=3<br>O=4	O=1<br>O=2<br>O=3<br>O=4	O=1<br>O=2<br>O=3<br>O=4	O
**18** Blood loss/ Bleeding (external)	O=1<br>O=2<br>O=3<br>O=4		O=1<br>O=2<br>O=3<br>O=4		O=1<br>O=2<br>O=3<br>O=4	O=1<br>O=2<br>O=3<br>O=4	O=1<br>O=2<br>O=3<br>O=4	O=1<br>O=2<br>O=3<br>O=4	O=1<br>O=2<br>O=3<br>O=4	O
**19** Sneezing Runny nose	O=1<br>O=2<br>O=3<br>O=4		O=1<br>O=2<br>O=3<br>O=4		O=1<br>O=2<br>O=3<br>O=4	O=1<br>O=2<br>O=3<br>O=4	O=1<br>O=2<br>O=3<br>O=4	O=1<br>O=2<br>O=3<br>O=4	O=1<br>O=2<br>O=3<br>O=4	O
**20** Cough	O=1<br>O=2<br>O=3<br>O=4		O=1<br>O=2<br>O=3<br>O=4		O=1<br>O=2<br>O=3<br>O=4	O=1<br>O=2<br>O=3<br>O=4	O=1<br>O=2<br>O=3<br>O=4	O=1<br>O=2<br>O=3<br>O=4	O=1<br>O=2<br>O=3<br>O=4	O
**21** Rapid breathing/ shortness of breath	O=1<br>O=2<br>O=3<br>O=4		O=1<br>O=2<br>O=3<br>O=4		O=1<br>O=2<br>O=3<br>O=4	O=1<br>O=2<br>O=3<br>O=4	O=1<br>O=2<br>O=3<br>O=4	O=1<br>O=2<br>O=3<br>O=4	O=1<br>O=2<br>O=3<br>O=4	O
**22** Severe breathing/ Difficulties in breathing	O=1<br>O=2<br>O=3<br>O=4		O=1<br>O=2<br>O=3<br>O=4		O=1<br>O=2<br>O=3<br>O=4	O=1<br>O=2<br>O=3<br>O=4	O=1<br>O=2<br>O=3<br>O=4	O=1<br>O=2<br>O=3<br>O=4	O=1<br>O=2<br>O=3<br>O=4	O
**23** Skin inflammation (rashes/ redness/ pus/ itching)	O=1<br>O=2<br>O=3<br>O=4		O=1<br>O=2<br>O=3<br>O=4		O=1<br>O=2<br>O=3<br>O=4	O=1<br>O=2<br>O=3<br>O=4	O=1<br>O=2<br>O=3<br>O=4	O=1<br>O=2<br>O=3<br>O=4	O=1<br>O=2<br>O=3<br>O=4	O
**24** Swelling/ Edema	O=1<br>O=2<br>O=3<br>O=4		O=1<br>O=2<br>O=3<br>O=4		O=1<br>O=2<br>O=3<br>O=4	O=1<br>O=2<br>O=3<br>O=4	O=1<br>O=2<br>O=3<br>O=4	O=1<br>O=2<br>O=3<br>O=4	O=1<br>O=2<br>O=3<br>O=4	O
**25** Jaundice	O=1<br>O=2<br>O=3<br>O=4		O=1<br>O=2<br>O=3<br>O=4		O=1<br>O=2<br>O=3<br>O=4	O=1<br>O=2<br>O=3<br>O=4	O=1<br>O=2<br>O=3<br>O=4	O=1<br>O=2<br>O=3<br>O=4	O=1<br>O=2<br>O=3<br>O=4	O

For peer review only

Site ___ ___ ___ Subarea ___ ___ ___ House number ___ ___ ___ ___ Interviewer ID ___ ___

FORM 4: ASSESSMENT OF OVERALL, AGE- AND SEX-STRATIFED GENERIC HEALTHCARE UTILIZATION

Part B – Healthcare-associated factors/Vaccination/Disease perception

TRAVEL HEALTHCARE FACILITY

1 In case a healthcare facility (HCF) was rated the 1st choice of care in question 4A (fever), what is the main mode of transportation, travel time (depending on mode of transportation), travel cost (depending on mode of transportation) and travel distance (depending on mode of transportation) that is usually taken to get there?

Instructions: Enter one code per category and sex as well as age group only. Skip this question if there is no female/male member <2years/≥2 to <5years/≥5 to <15years/≥15years of age in this household (see also question 2 and 3 of this form PartA). Skip this question if no HCF was rated the 1st choice of care in question 4A for female/male member <2years/≥2 to <5years/≥5 to <15years/≥15years of age. Record the travel time as minute(s) [min]/ hour(s) [hr], the total costs in USD, including potential gifts, favours and tips, and the total distance as meter(s) [m]/kilometre(s) [km].

Female				Male			
<2 years	≥2 to <5 years	≥5 to <15 years	≥15 years	<2 years	≥2 to <5 years	≥5 to <15 years	≥15 years
**1.1 Transportation mode**							
Code ___	Code ___	Code ___	Code ___	Code ___	Code ___	Code ___	Code ___
[1] Private transportation (i.e. motorbike, car, bus) [2] Public transportation (i.e. motorbike, car, bus)				[3] Walking [4] Bicycle	[5] Other, specify _________ [99] Don't know		[98] No response
**1.2 Travel time**							
Code ___	Code ___	Code ___	Code ___	Code ___	Code ___	Code ___	Code ___
[1] <30 mins [2] ≥30 to <60 mins		[3] ≥1.0 to <2.0 hrs [4] ≥2.0 to <3.0 hrs		[5] ≥3.0 to <4.0 hrs [6] ≥4.0 hrs		[99] Don't know [98] No response	
**1.3 Travel cost**							
Code ___	Code ___	Code ___	Code ___	Code ___	Code ___	Code ___	Code ___
[1] <1.0 USD [2] ≥1.0 to <3.0 USD		[3] ≥3.0 to <5.0 USD [4] ≥5.0 to <10.0 USD		[5] ≥10.0 USD		[99] Don't know [98] No response	
**1.4 Travel distance**							
Code ___	Code ___	Code ___	Code ___	Code ___	Code ___	Code ___	Code ___
[1] <500 m [2] ≥500 m to <1.0 km		[3] ≥1.0 to <2.0 km [4] ≥2.0 to <3.0 km		[5] ≥3.0 to <4.0 km [6] ≥4.0 km		[99] Don't know [98] No response	

INSURANCE

2 For which members of this household does the insurance cover health expenses?

Instructions: Enter one code per category. Skip this question if there is no member <2years/≥2 to <5years/≥5 to <15years/≥15years of age in this household (see question 2 and 3 of this form PartA).

<2 years	≥2 to <5 years	≥5 to <15 years	≥15 years
Code ____________	Code ____________	Code ____________	Code ____________
[1] Yes	[2] No	[99] Don't know	[98] No response

For peer review only - http://bmjopen.bmj.com/site/about/guidelines.xhtml

Site ___ ___ ___ Subarea ___ ___ ___ House number ___ ___ ___ ___ Interviewer ID ___ ___

3 What kind of health expenses are usually covered by the insurance for members of this household?

Instructions: Enter one code by category and age group only. Skip this question if there is no member <2years/≥2 to <5years/≥5 to <15years/≥15years of age in this household (see also question 2 and 3 of this form PartA). Skip this question if there is no insurance that covers health expenses for <2years/≥2 to <5years/≥5 to <15years/≥15years of age.

	<2 years	≥2 to <5years	≥5 to <15years	≥15years
	[1] Yes	[2] No	[99] Don't know	[98] No response
**1** Registration	Code ______	Code _______	Code _______	Code _____
**2** Consultation/ Doctor's visit	Code ______	Code _______	Code _______	Code _____
**3** Physical examination - general	Code ______	Code _______	Code _______	Code _____
**4** Physical examination – specific, specify_______	Code ______	Code _______	Code _______	Code _____
**5** Diagnostics - general laboratory diagnostics	Code ______	Code _______	Code _______	Code _____
**6** Diagnostics - specific laboratory diagnostics, specify__________	Code ______	Code _______	Code _______	Code _____
**7** Diagnostics - general imaging	Code ______	Code _______	Code _______	Code _____
**8** Diagnostics - specific imaging, specify__________	Code ______	Code _______	Code _______	Code _____
**9** Diagnostics - other, specify________	Code ______	Code _______	Code _______	Code _____
**10** Treatment - general medication	Code ______	Code _______	Code _______	Code _____
**11** Treatment - specific medication, specify________	Code ______	Code _______	Code _______	Code _____
**12** Treatment - other medication, specify__________	Code ______	Code _______	Code _______	Code _____
**13** Treatment - interventions (other than surgery), specify_______	Code ______	Code _______	Code _______	Code _____
**14** Treatment Surgery	Code ______	Code _______	Code _______	Code _____
**15** Hospitalization <7days	Code ______	Code _______	Code _______	Code _____
**16** Hospitalization ≥7days	Code ______	Code _______	Code _______	Code _____
**17** Emergency services	Code ______	Code _______	Code _______	Code _____
**18** Antenatal care	Code ______	Code _______	Code _______	Code _____
**19** Postnatal care	Code ______	Code _______	Code _______	Code _____
**20** Preventive medicine	Code ______	Code _______	Code _______	Code _____
**21** Vaccination	Code ______	Code _______	Code _______	Code _____
**22** Home healthcare (i.e. elderly, disabled, chronic/long-term diseased)	Code ______	Code _______	Code _______	Code _____
**23** Supportive therapy (i.e. physiotherapy, ergotherapy, rehabilitation)	Code ______	Code _______	Code _______	Code _____

For peer review only - http://bmjopen.bmj.com/site/about/guidelines.xhtml

Site ___ ___ ___ Subarea ___ ___ ___ House number ___ ___ ___ ___ Interviewer ID ___ ___

ETHNOGRAPHY

4 To which religion/ethnic group/tribe belong members of this household?

Instructions: Give one answer by age group only. Skip this question if there is no member >2years/≥2 to <5years/≥5 to <15years/≥15years of age in this household (see also question 2 and 3 of this form PartA).

	<2 years	≥2 to <5years	≥5 to <15years	≥15years
Religion/Ethnic group/Tribe	______________ O 99=Don't know O 98=No response	______________ O 99=Don't know O 98=No response	______________ O 99=Don't know O 98=No response	______________ O 99=Don't know O 98=No response

5 Does the religion/ethnic group/tribe as stated in question 4 influence the various aspects of utilization of healthcare for members of this household?

Instructions: Enter one code by category and age group only. Skip this question if there is member >2years/≥2 to <5years/≥5 to <15years/≥15years of age in this household (see also question 2 and 3 of this form PartA).

	<2 years	≥2 to <5years	≥5 to <15years	≥15years
	[1] Yes	[2] No	[99] Don't know	[98] No response
**1** Visiting a healthcare facility (i.e. hospital, health care center, health post)	Code _____	Code _______	Code _______	Code _____
**2** Visiting a physician	Code _____	Code _______	Code _______	Code _____
**3** Visiting a pharmacy	Code _____	Code _______	Code _______	Code _____
**4** Visiting a traditional healer	Code _____	Code _______	Code _______	Code _____
**5** Deciding for self-treatment	Code _____	Code _______	Code _______	Code _____
**6** Deciding for non-treatment	Code _____	Code _______	Code _______	Code _____
**7** Physical examination – general	Code _____	Code _______	Code _______	Code _____
**8** Physical examination – specific, specify________	Code _____	Code _______	Code _______	Code _____
**9** Collection of specimen (i.e. blood, urine, stool, smear, swab, cerebrospinal fluid)	Code _____	Code _______	Code _______	Code _____
**10** Diagnostics – general laboratory diagnostics	Code _____	Code _______	Code _______	Code _____
**11** Diagnostics – specific laboratory diagnostics, specify___________	Code _____	Code _______	Code _______	Code _____
**12** Diagnostics - general imaging	Code _____	Code _______	Code _______	Code _____
**13** Diagnostics - specific imaging, specify_________	Code _____	Code _______	Code _______	Code _____
**14** Diagnostics - other, specify_________	Code _____	Code _______	Code _______	Code _____
**15** Treatment - general medication	Code _____	Code _______	Code _______	Code _____
**16** Treatment - specific medication, specify________	Code _____	Code _______	Code _______	Code _____
**17** Treatment - other medication, specify___________	Code _____	Code _______	Code _______	Code _____

For peer review only - http://bmjopen.bmj.com/site/about/guidelines.xhtml

Site ___ ___ ___ Subarea ___ ___ ___ House number ___ ___ ___ ___ Interviewer ID ___ ___

**18** Treatment - interventions (other than surgery), specify________________	Code _____	Code _______	Code _______	Code _____
**19** Treatment - Surgery	Code _____	Code _______	Code _______	Code _____
**20** Hospitalization <7days	Code _____	Code _______	Code _______	Code _____
**21** Hospitalization ≥7days	Code _____	Code _______	Code _______	Code _____
**22** Emergency services	Code _____	Code _______	Code _______	Code _____
**23** Antenatal care	Code _____	Code _______	Code _______	Code _____
**24** Postnatal care	Code _____	Code _______	Code _______	Code _____
**25** Preventive medicine	Code _____	Code _______	Code _______	Code _____
**26** Vaccination	Code _____	Code _______	Code _______	Code _____
**27** Home healthcare (i.e. elderly, disabled, chronic/long-term diseased)	Code _____	Code _______	Code _______	Code _____
**28** Supportive therapy (i.e. physiotherapy, ergotherapy, rehabilitation)	Code _____	Code _______	Code _______	Code _____

VACCINATION

6 How were children of this household fed during the first six months of life?

Instructions: Skip this question if there are no male/female children in this household (see also question 2 and 3 of this form PartA).

- O 1=Breastfed exclusively
- O 2=Breastfed and other feeds, specify _______
- O 3=Formula and other feeds, specify _________
- O 4=Breastfed and formula
- O 5=Formula exclusively
- O 6=Other, specify _________________
- O 99=Don't know
- O 98=No response

7 Which water source was usually used to prepare formula or formula and other feeds as stated in question 6 for children of this household?

Instructions: Skip this question if there are no children in this household (see also question 2 and 3 of this form PartA).

- O 1=Tap water (inside the house; private)
- O 2=Tap water (outside the house i.e. back-/courtyard; private)
- O 3=Tap water (outside the house; public, shared)
- O 4=Standpipe/Piped water (inside the house; private)
- O 5=Standpipe/Piped water (outside the house i.e. back-/courtyard; private)
- O 6=Standpipe/Piped water (outside the house; public, shared)
- O 7=Water truck (public; shared)
- O 8=Bottled water
- O 9=Surface water (i.e. lake, pond, river, stream)
- O 10=Surface water (i.e. canal, irrigation canal)
- O 11=Spring water
- O 12=Rain water
- O 13=Well/Borehole (open/uncovered/unprotected)
- O 14=Well/Borehole (covered/protected)
- O 15=NO FORMULA/OTHER FEEDS PREPARED
- O 16=Other, specify _____________
- O 99=Don't know
- O 98=No response

8 Did children of this household receive any vaccines since birth, including the birth vaccines?

Instructions: Enter one code by category and child only. Skip this question if there are no children in this household (see also question 2 and 3 of this form PartA). Refer to question 9 of "Form 3 Part A – Demographic data/General household information" and record the initials/identifier of the member(s).

Child 1	Child 2	Child 3	Child 4
Initials/Identifier: _____	Initials/Identifier: _____	Initials/Identifier: _____	Initials/Identifier: _____

For peer review only - http://bmjopen.bmj.com/site/about/guidelines.xhtml

Site ___ ___ ___ Subarea ___ ___ ___ House number ___ ___ ___ ___ Interviewer ID ___ ___

[1] YES, vaccinated	[5] No, lack of vaccine	[9] No, HCF busy
[2] No, caretaker busy	[6] No, child too old/young	[10] No, child sick
[3] No, caretaker decided no need	[7] No, caretaker refused/too worried	[11] No, no vaccination card
[4] No, unknown where to go	[8] No, other, specify ___________	[99] Don't know [98] No response

**8.1 Diphtheria and Tetanus and Pertussis and *Haemophilus influenza* and Hepatitis B vaccine/DTwPHibHepB** (e.g. 6/8, 10/12, 14/16 weeks)			
Code ___________	Code ___________	Code ___________	Code ___________
**8.2 Tetanus toxoid (TT)** (e.g. 1st contact pregnancy, 1, 6, 12/24 months)			
Code ___________	Code ___________	Code ___________	Code ___________
**8.3 Tetanus and diphtheria toxoid (TD)** (e.g. 1st contact pregnancy, 1, 6, 12 months)			
Code ___________	Code ___________	Code ___________	Code ___________
**8.4 Tuberculosis/BCG** (at birth)			
Code ___________	Code ___________	Code ___________	Code ___________
**8.5 Rotavirus/RV** (e.g. 6/8, 10/12, 16 weeks)			
Code ___________	Code ___________	Code ___________	Code ___________
**8.6 Measles/Measles and Rubella (MR)** (e.g. 9, 15/18months)			
Code ___________	Code ___________	Code ___________	Code ___________
**8.7 Polio/OPV** (e.g. birth, 6/8, 10/12, 14/16 weeks)			
Code ___________	Code ___________	Code ___________	Code ___________
**8.8 Pneumococcus/PCV** (e.g. 6/8, 10/12, 14/16 weeks)			
Code ___________	Code ___________	Code ___________	Code ___________
**8.9 Yellow fever (YF)** (e.g. 9 months)			
Code ___________	Code ___________	Code ___________	Code ___________
**8.10 Other Vaccines**			
Specify, ___________	Specify, ___________	Specify, ___________	Specify, ___________
Specify, ___________	Specify, ___________	Specify, ___________	Specify, ___________
Specify, ___________	Specify, ___________	Specify, ___________	Specify, ___________
Specify, ___________	Specify, ___________	Specify, ___________	Specify, ___________

For peer review only

For peer review only - http://bmjopen.bmj.com/site/about/guidelines.xhtml

Site ___ ___ ___ Subarea ___ ___ ___ House number ___ ___ ___ ___ Interviewer ID ___ ___

DISEASE PERCEPTION

9 Knowledge, perception, causes and prevention of common infectious diseases

**Disease**	**9.1 Disease known by household members?** [1] Yes [2] No [99] Don't know [98] No response	**9.2 Perception of disease by household members?** *Instruction:* Describe briefly if known.	**9.3 Causes for disease known by household members?** *Instructions:* Enter all codes (list A) that apply; more than one answer is possible.	**9.4 Measures to prevent disease known by household members?** *Instructions:* Enter all codes (list B) that apply; more than one answer is possible.	**9.5 Measures to prevent disease utilized by household members?** *Instructions:* Enter all codes (list B) that apply; more than one answer is possible.
**A.** Typhoid fever	Code __________		Code __________	Code __________	Code __________
**B.** Cholera	Code __________		Code __________	Code __________	Code __________
**C.** Malaria	Code __________		Code __________	Code __________	Code __________
**D.** Influenza	Code __________		Code __________	Code __________	Code __________
**E.** Meningitis	Code __________		Code __________	Code __________	Code __________
**F.** Hepatitis E	Code __________		Code __________	Code __________	Code __________
**G.** Tuberculosis	Code __________		Code __________	Code __________	Code __________
**H.** HIV/AIDS	Code __________		Code __________	Code __________	Code __________

List A: Possible causes	List B: Possible preventive measures
[1] Alcohol consumption<br>[2] Weather/witchcraft<br>[3] Mosquito/insect bite<br>[4] Dog bite	[1] No alcohol consumption<br>[2] Sleeping under bed net<br>[3] Using mosquito/insect spray<br>[4] Maintain a clean house and immediate surroundings of the house

For peer review only - http://bmjopen.bmj.com/site/about/guidelines.xhtml

Site ___ ___ ___ Subarea ___ ___ ___ House number ___ ___ ___ ___ Interviewer ID ___ ___

[5] Drinking untreated surface/spring water (i.e. lake, pond, river, stream, canal/irrigation canal)<br>[6] Close body contact/kissing/hugging<br>[7] Eating raw food items such as vegetables/lettuce, fruits, dairy products, eggs, meat or fish<br>[8] Not cleaning hands properly when preparing food/meals<br>[9] Not cleaning hands properly before/after visiting a toilet<br>[10] Contact to human blood (i.e. injection, transfusion, surgery)<br>[11] Consuming raw animal blood<br>[12] Other, specify _______________<br>[99] Don't know<br>[98] No response	[5] Practice proper disposal of human and domestic waste<br>[6] Avoiding close contact to dogs<br>[7] Treating water (i.e. boiling, filtration, solar disinfection, sedimentation, chlorination)<br>[8] Not cooking/preparing food/meals with untreated water<br>[9] Not washing food items to be consumed raw with untreated water<br>[10] Re-boiling/re-heating left-over/prepared food/meals prior to consumption<br>[11] Not eating raw food items such as vegetables/lettuce, fruits, dairy products, eggs, meat or fish<br>[12] Cover food items/meals<br>[13] Protect food items/meals from flies<br>[14] Avoid food/beverage from street vendors<br>[15] Avoid close contact to sick people<br>[16] No close body contact/kissing/hugging<br>[17] Covering nose/mouth while sneezing/coughing<br>[18] No contact/consumption of human/animal blood<br>[19] Practicing proper hand hygiene when preparing food/meals (i.e. washing hands with soap and water, rub hands clean)<br>[20] Practicing proper hand hygiene before/after visiting a toilet (i.e. washing hands with soap and water, rub hands clean)<br>[21] Other, specify ____________________<br>[99] Don't know<br>[98] No response

For peer review only

Site ___ ___ ___ Subarea ___ ___ ___ House number ___ ___ ___ ___ Interviewer ID ___ ___

10 For which members of this household are the preventive measures as listed in question 9.5 against typhoid fever used?

Instructions: Tick all that applies; more than one answer is possible.

O 1=<2years O 3=≥5 to <15years O 5=NOT USED O 99=Don't know
O 2=≥2 to < 5years O 4=≥15years O 98=No response

11 Name the household members that were diagnosed with typhoid fever during the past 3 months. Which diagnostics were used to confirm typhoid fever?

Instructions: Enter all codes that apply for each household member; more than one answer is possible.

Member 1 Initials/Identifier: ______	Member 2 Initials/Identifier: ______	Member 3 Initials/Identifier: ______	Member 4 Initials/Identifier: ______
[1] Blood culture [2] Stool culture	[3] Blood/Widal test [4] Other test, specify______________	[5] Not applicable (not diagnosed with typhoid fever) [99] Don't know	[98] No response
Code ___________	Code ___________	Code ___________	Code ___________

12 (Where were members of this household recorded in question 11 diagnosed for typhoid fever?) Where did members of this household seek care?

Instructions: Enter one code by household member only.

Member 1 Initials/Identifier: ______	Member 2 Initials/Identifier: ______	Member 3 Initials/Identifier: ______	Member 4 Initials/Identifier: ______
[1] Healthcare facility, specify ____________ [2] Physician [3] Nowhere, self-treatment [4] Traditional healer [5] Pharmacy		[6] Nowhere, did not seek care [7] Other, specify ____________ [8] Not applicable [99] Don't know [98] No response	
Code ___________	Code ___________	Code ___________	Code ___________

13 (Did members of this household that were diagnosed with typhoid fever as recorded in question 11 get any treatment?) Which treatment did they get?

Instructions: Tick one answer by household member only.

Member 1 Initials/Identifier: ______	Member 2 Initials/Identifier: ______	Member 3 Initials/Identifier: ______	Member 4 Initials/Identifier: ______
O 1=Treatment, specify _______________ O 2=Not applicable O 99=Don't know O 98=No response	O 1=Treatment, specify _______________ O 2=Not applicable O 99=Don't know O 98=No response	O 1=Treatment, specify _______________ O 2=Not applicable O 99=Don't know O 98=No response	O 1=Treatment, specify _______________ O 2=Not applicable O 99=Don't know O 98=No response

For peer review only - http://bmjopen.bmj.com/site/about/guidelines.xhtml

Site ___ ___ ___ Subarea ___ ___ ___ House number ___ ___ ___ ___ Interviewer ID ___ ___

FORM 5: ASSESSMENT OF OVERALL, AGE-AND SEX-STRATIFIED ACTUAL HEALTHCARE UTILIZATION

HEALTHCARE SEEKING BEHAVIOR

1 Record below any occurrence of a sign(s)/ a symptom(s) (see List 1) and specify where (see List 2) and how often a member/members sought healthcare during the past 3 months?

Instructions: See "Form 3 - Part A - GENERAL HOUSEHOLD INFORMATION" to obtain the initials/identifier of each household member. Indicate one sign/symptom per line only. Start a new line if different healthcare options were selected for recurring signs/symptoms. Indicate the number of occurrences for each sign/symptom during the past 3 months. If a different healthcare option was taken for a recurring sign/symptom, indicate this on a new line. Please complete an additional form if >20 occurrences took place during the past 3 months.

**List 1: Sign/Symptom**		**List 2: Healthcare option**
[A] Fever, any<br>[B] Fever <3days (continuous)<br>[C] Fever ≥3days (continuous)<br>[D] Chills/ Shivering<br>[E] Convulsion<br>[F] Weight loss<br>[G] Dehydration<br>[H] Malaise/ Weakness/ Fatigue<br>[I] Aches/Pain (muscle/joint/bone)<br>[J] Headache<br>[K] Dizziness/Confusion/ Unconsciousness<br>[L] Blood pressure disorders<br>[M] Heart disorders	[N] Nausea/ Vomiting<br>[O] Diarrhea (watery/bloody/mucopurulent)<br>[P] Abdominal pain/ Intestinal pain<br>[Q] Blood loss/ Bleeding (internal)<br>[R] Blood loss/ Bleeding (external)<br>[S] Sneezing/ Runny nose<br>[T] Cough<br>[U] Rapid breathing/ Shortness of breath<br>[V] Severe breathing/ Difficulties in breathing<br>[W] Skin inflammation (rashes/redness/ pus/itching)<br>[X] Swelling/ Edema<br>[Y] Jaundice	[1] Study-HCF, Code ________<br>[2] Other HCF, Code ________<br>[3] Physician<br>[4] Pharmacy<br>[5] Traditional healer<br>[6] Nowhere/Self-treatment<br>[7] Nowhere/Nothing

**No.**	**Household member** Record the initials/identifier of a household member; see "Form 3 - Part A - GENERAL HOUSEHOLD INFORMATION"	**Sign/ Symptom** Use List 1 to select a code	**Healthcare option** Use List 2 to select a code	HCF code	**Frequency** Indicate the number of incidences for the sign/symptom listed
1					
2					
3					
4					
5					
6					
7					
8					
9					

For peer review only - http://bmjopen.bmj.com/site/about/guidelines.xhtml

Site ___ ___ ___ Subarea ___ ___ ___ House number ___ ___ ___ ___ Interviewer ID ___ ___

10					
11					
12					
13					
14					
15					
16					
17					
18					
19					
20					

For peer review only

For peer review only - http://bmjopen.bmj.com/site/about/guidelines.xhtml

Appendix 3/Supplementary file 3: Protocol, Definitions/Terminology and Study Forms (French)

Protocol
Évaluation du comportement de santé et d'autres facteurs démographiques: une étude standardisée dans plusieurs cohortes en Afrique subsaharienne

Abstrait
Introduction L'objectif de l'étude HPAfrica est de déterminer les comportements sanitaires et autres facteurs démographiques, y compris les facteurs socio-économiques, ethnographiques aussi bien que d'hygiène et d'assainissement sur les sites d'étude du programme Fièvre Typhoïde Sévère en Afrique (SETA). L'objectif du programme SETA est d'enquêter sur la surveillance de la fièvre au niveau des établissements de soins de santé au Burkina Faso, en République Démocratique du Congo (RDC), en Éthiopie, au Ghana, à Madagascar et au Nigéria. Les estimations précises de la charge de morbidité nécessitent un ajustement des styles de comportements sanitaires, qui sont supposés varier au sein d'une population d'étude.

Methodes Pour la taille d'échantillon minimale requise, les hypothèses d'une population infinie, d'un effet de plan et d'une stratification par âge et sexe sont considérées. En l'absence d'un cadre d'échantillonnage ou d'une liste de ménages, une approche géospatiale sera utilisée pour générer des points aléatoires géographiques avec un outil ArcGIS (Aeronautical Reconnaissance Coverage Geographic Information System). Les impressions de l'imagerie satellite Google Earth Pro® visualisent ces points. Les données d'intérêt seront évaluées au niveau des ménages en différentes saisons en appliquant un échantillonnage simple, stratifié en fonction du poids. Une application basée sur Android et un service Web ont été mis au point pour la capture de données électroniques et la synchronisation avec le serveur de base de données sur Internet en temps réel. Cela permet la documentation de données simultanée.

Analyse Des analyses de données descriptives seront effectuées afin d'évaluer les informations de base sur / à propos de chaque population d'étude HPAfrica et le comportement santé stratifié par âge et par sexe. Ceci permettra d'ajuster les estimations de la charge de morbidité. En outre, des analyses multivariées seront appliquées pour examiner les associations entre le comportement santé, les facteurs demographiques et la charge de morbidité (fardeau de la maladie) trouvée dans l'étude SETA.

Défis attendus
- La normalisation dans la recherche multi-pays /-site à base communautaire nécessite de la prudence compte tenu des variations de cadre, de langue et de culture.
- Les données de population actuelles dans les sites d'étude et leurs sous-zones définies administrativement sont supposées être limitées.
- L'accessibilité aux ménages peut être compliqué par des facteurs environnementaux.
- La collecte de données électroniques peut entraîner problèmes techniques liés aux dommages, aux pannes fonctionnelles ou à la perte de dispositifs.
- Une connexion Internet sans fil instable peut limiter les équipes de données à mettre à jour l'application basée sur Android, communiquer en temps réel avec les responsables de l'étude et à synchroniser les données collectées avec le serveur.

Ethics References
- Comité d'Examen Institutionnel d'IVI, No. 2015-006
- Institute de Médecine Tropicale d'Antwerp, Comité d'Examen, Belgique
- Universiteit Antwerpen, Comite voor medische Ethiek, Belgique
- Ministère de la Santé du Burkina Faso – Comité d'Ethique pour la Recherche en Santé, Burkina Faso
- Comité d'Ethique de l'Ecole de Santé Publique de l'Université de Kinshasa, République Démocratique du Congo

For peer review only - http://bmjopen.bmj.com/site/about/guidelines.xhtml

- Comité National d'Examen de l'Éthique de la Recherche, Ministère des Sciences et de la Technologie, République Fédérale Démocratique d'Éthopie
- Comité d'Éthique de l'AHRI-ALERT, République Fédérale Démocratique d'Éthopie
- Kwame Nkrumah Université ded Sciences et de la Technologie, École des Sciences Médicales/Hôpital Universitaire Komfo Anokye, Comité de la Recherche humaines, de la Publication et de l'Éthique, Ghana
- Ministère de la Santé du Repoblikan Madagaskar – Comité d'Ethique
- Comité d'Éthique de l'Université d'Idadan/ Hôpital du Collège Universitaire/Comité de l'Éthique, Ibadan, Nigéria

Mots clés: comportement santé/hygiénique, assainissement, socio-économique, sélection aléatoire geospatiale, Afrique subsaharienne, HPAfrica étude

Contexte
L'évaluation du comportement de santé, y compris l'utilisation des soins de santé, est un déterminant important pour la production d'estimations précises du fardeau de la maladie parmi les populations cibles. Ceci est particulièrement important pour les études qui utilisent un modèle de surveillance observationnelle basé sur les établissements de santé, car une variation substantielle des comportements de santé influence fortement les estimations de la maladie (1, 2). L'ajustement des calculs de la maladie pour des variations du comportement en matière de santé augmentera l'exactitude de la prévalence des maladies causée par des pathogènes bactériens, fongiques, viraux ou parasitaires parmi la population étudiée. De plus, l'influence d'une grande variété de facteurs basés sur la population sur le comportement en matière de santé et le fardeau de la maladie a été reconnue. Cela inclut des facteurs socioéconomiques tels que l'éducation, la profession, le revenu, la disponibilité des biens des ménages (3, 4), les facteurs ethniques / religieux et culturels (5, 6), mais aussi l'hygiène et l'assainissement (7-11) - 14), et les contacts avec des animaux (10).

L'étude **H**ealth **P**opulation **Africa** (HPAfrica) sera menée au Burkina Faso, en République démocratique du Congo (RDC), en Éthopie, au Ghana, à Madagascar et au Nigéria (Tableau 1). Les expériences du programme Typhoid Fever Surveillance in Africa Program (TSAP) seront reportées sur le programme Severe Typhoid in Africa (SETA) (15, 16). Dans ce programme, une surveillance standardisée de la fièvre, basée sur les établissements de santé, est effectuée sur des sites d'étude sélectionnés dans les pays participants sur une période de deux ans (17). SETA vise principalement à collecter systématiquement des informations sur le fardeau des infections graves à *Salmonella*. Les sujets vivant dans la zone the chalandise définie, avec un épisode de fièvre aiguë, des antécédents de fièvre ≥ 3 jours consécutifs, une fièvre typhoïde cliniquement soupçonnée ou des perforations gastro-intestinales cliniquement diagnostiquées dues à la fièvre typhoïde seront éligibles. En outre, la sévérité des maladies provoquées par *Salmonella* et l'histoire naturelle de l'infection à *Salmonella* seront enregistrées avec des analyses supplémentaires sur les réponses immunitaires de l'hôte et le portage chronique.

Objectifs et Résultats
L'objectif de l'étude HPAfrica est d'évaluer le comportement de santé générique et réel stratifié par âge et par sexe. Le comportement générique se rapporte au comportement associé à la fièvre et à d'autres signes et symptômes sans l'apparition, alors que le comportement réel est lié à l'apparition (Formulaire 4 Partie A, Formulaire 5). Les données sont utilisées pour ajuster les incidences d'agents pathogènes infectieux stratifiés selon l'âge et le sexe pour la proportion d'une population étudiée non capturée par les activités de HPAfrica. Les données documentaires pour divers signes et symptômes ou une combinaison de ceux-ci sont utilisés pour une classification progressive de l'utilisation des soins de santé en fonction de la gravité de la maladie.

L'étude HPAfrica recueillera des données sur les facteurs associés aux soins de santé, tels que les modalités de voyage, la possession d'une assurance maladie, les facteurs culturels/ethnographiques, l'état vaccinal des enfants (18, 19) et la fréquence et la perception individuelle des maladies (Formulaire

For peer review only - http://bmjopen.bmj.com/site/about/guidelines.xhtml

4 Partie B). Les données démographiques (Formulaire 3 Partie A) seront utilisés pour la stratification par âge et sexe de la population étudiée qui est nécessaire pour calculer les facteurs d'ajustement pour les incidences de la maladie. Les données socio-économiques (Formulaire 3 Partie B) seront utilisées pour calculer les indices de richesse pour les approches descriptives et analytiques des populations étudiées. Les informations sur l'hygiène et l'assainissement (Formulaire 3 Partie C) peuvent permettre une meilleure compréhension de l'occurrence et de la fréquence des agents pathogènes identifiés parmi les populations étudiées.

Méthodes

Sites d'étude

Les limites de chaque site seront définies en utilisant des indications préexistantes qui avaient été déterminées par les autorités statistiques ou ministères de la santé (Tableau 1). L'examen rétrospectif des dossiers (registres/archives) de des deux dernières années aux établissements de soins de santé de recrutement permettra de redéfinir les limites du site. On s'attend à ce que cette revue révèle les résidences ou au moins les zones résidentielles administratives plus larges telles que des communautés, des districts ou des villages de patients qui ont cherché des soins de santé pour une raison quelconque. Des cartes et des images satellites peuvent être utilisées pour mieux visualiser les résidences des patients et les limites générales, y compris les sous-zones définies géographiquement ou administrativement de chaque site. Là où les limites ne peuvent être clairement définies, des facteurs supplémentaires, tels que la distance à un établissement de soins de santé de recrutement, peuvent être pris en compte.

Taille d'échantillons

Toutes les sources disponibles seront utilisées pour recueillir des données sur la population le plus à jour stratifiées selon l'âge et le sexe par site d'étude et par les sous-zones définies administrativement ou géographiquement. Cela peut inclure les dernières informations démographiques issues d'un recensement de la population ou d'un DSS (Demographic Surveillance System) / HDSS (Health and Demographic Surveillance System). Les chiffres de population et les facteurs de croissance de la population, s'ils sont disponibles, peuvent également être utilisés.

Le nombre minimum de ménages sélectionnés au hasard sera calculé en utilisant l'équation basée de précision (équation-I) (20-23) en supposant une population infinie et en considérant un effet de design (design effect, DEFF), y compris les hypothèses qui l'accompagnent:

Equation-I: Calcul de la taille d'échantillon basé sur la précision pour une population infinie (20-23):

$$n_0 = DEFF \cdot \left[z^2{}_{1-\alpha/2} \cdot \frac{4 \cdot p \cdot (1-p)}{d^2} \right]$$

<u>Hypothèses d'Equation-I</u>

n_0	Nombre minimum de ménages à interroger dans une zone d'étude en supposant une population infinie
DEFF	Effect de design; fixé à 1.5
$z_{1-\alpha/2}$	Déviation Standard correspondant à un intervalle de confiance de 95% (1.96 pour alpha de 0.05)
d	Précision (erreur acceptable); estimation de point; fixé à 0.2
p	Proportion de la population de l'étude devant se rendre dans un établissement de soins de santé de recrutement pour des conditions associées à de la fièvre et d'autres signes et symptômes (proportion capturée); fixé à 0.2
1-p	Proportion de la population de l'étude ne devant se rendre dans un établissement de soins de santé de recrutement pour des conditions associées à de la fièvre et d'autres signes et symptômes (proportion non capturée)

For peer review only - http://bmjopen.bmj.com/site/about/guidelines.xhtml

L'effet de design est un facteur d'ajustement pour le regroupement naturel des comportements de santé au sein des membres d'un ménage (21). Il est défini de façon arbitraire à 1,5 selon la taille du ménage puisqu'aucune estimation exacte n'est disponible (24, 25). Une proportion p de 0,2 pourra être considérée si aucune autre estimation n'est disponible ou p pourra être basé sur les expériences au cours de l'étude TSAP: Madagascar, Isotry : p=0,01, Burkina Faso, Polesgo: p=0,9 (26). Combiner tous les hypothèses dans l'équation-I va avoir pour résultat un nombre global minimum (n_0) de 92 ménages à interroger à chaque site.

En outre, n_0 pourra être pris en compte pour la répartition par âge et sexe de chaque population de l'étude représentée par $\hat{p}$, la proportion minimum supposée de stratification par groupe d'âge (<5 ans, ≥5 à <15 ans, ≥15 ans). Le facteur $\hat{p}$ pourra être défini de façon arbitraire à 0,2 ou être basé sur les expériences précédentes: Éthopie, Butajira : $\hat{p}$ =0,09, Burkina Faso, Polesgo : $\hat{p}$ =0,23 (26). Appliquer $\hat{p}$ à l'Equation-I pour une distribution binomiale, y compris ses postulats connexes, aura pour résultat le nombre minimum de ménages à interroger stratifié selon l'âge (Equation-II):

Equation-II: Calcul de la taille d'échantillon basé sur la précision pour une population infinie avec stratification selon l'âge:

$$\overline{n_0} = \left(DEFF \cdot \left[z^2{}_{1-\frac{\alpha}{2}} \cdot \frac{4 \cdot p \cdot (1-p)}{d^2}\right]\right)/\hat{p}$$

Hypothèses d'Equation-II

$\overline{n_0}$	Nombre minimum de ménages à interroger dans une zone d'étude en supposant une population infinie et une stratification selon l'âge
DEFF	Effect de design; fixé à 1.5
$z_{1-\alpha/2}$	Déviation Standard correspondant à un intervalle de confiance de 95% (1.96 pour alpha de 0.05)
d	Précision (erreur acceptable); estimation de point; fixé à 0.2
p	Proportion de la population de l'étude devant se rendre dans un établissement de soins de santé de recrutement pour des conditions associées à de la fièvre et d'autres signes et symptômes (proportion capturée); fixé à 0.2
1-p	Proportion de la population de l'étude ne devant se rendre dans un établissement de soins de santé de recrutement pour des conditions associées à de la fièvre et d'autres signes et symptômes (proportion non capturée)
$\hat{p}$	Proportion minimum de stratification par groupe d'âge, définie à 0,2

Les comportements de santé seront évalués deux fois dans les mêmes ménages si possible ou dans des ménages alternatifs, au cas où la population de l'étude serait instable en raison de la migration et / ou de taux de natalité / mortalité élevés. Des ménages supplémentaires, supposés être de 20% en moyenne (Tableau 2), peuvent être ajoutés pour tenir compte de la possible perte de suivi entre les deux évaluations. Eventuellement, $\overline{n_0}$ sera divisé par le nombre de sous-zones et pondéré proportionnellement à la taille de la population en appliquant un échantillonnage stratifié selon le poids (16).

Echantillonnage

Si une base d'échantillonnage ou une liste de ménages compréhensif et à jour existe (par DSS / HDSS ou recensement disponible), une sélection aléatoire informatisée sera effectuée en utilisant SAS (Système d'analyse statistique, version 9.4, SAS Institute, Cary NC) en appliquant une sélection aléatoire simple en série sans remplacement. S'il n'existe aucune base de sondage, celle-ci peut être construite en utilisant l'imagerie satellitaire ou les cartes aériennes pour dénombrer toutes les structures (26), raster (27) ou cellules de grille (28, 29).

Cependant, nous utilisons une technique géospatiale pour la création de points générés par l'outil de génération de points ArcGIS (Aeronautical Reconnaissance Coverage Geographic Information System, Redlands, CA, USA, version 10.2) avec l'application d'un échantillonnage simple stratifié selon le poids

For peer review only - http://bmjopen.bmj.com/site/about/guidelines.xhtml

(30, 31). L'outil place de façon aléatoire des nombres de points spécifiés dans les entités d'un polygone correspondant à une sous-zone administrative. Les coordonnées X et Y définissent un point jusqu'à ce que la taille d'échantillon soit atteinte. Les points aléatoires géographiques générés par ArcGIS sont convertis en un fichier image au format KML (Keyhole Markup Language) ou KMZ (Keyhole Markup landguage Zipped) et importés dans Google Earth Pro® (Google Inc., Mountain View, CA, États-Unis, version 6.2.2.6613). Un identifiant numérique unique et des coordonnées géographiques horizontales sont affectés à chaque point. L'emplacement de chaque point est vérifié sur une imagerie satellite Google Earth Pro® mise à jour. L'annexe 4 illustre notre approche en utilisant des exemples du Ghana et de Madagascar.

Les points géographiques étiquetés avec des identifiants sont représentés sur des impressions de taille d'affiche (60 x 60 à 60 x 90 cm) d'images satellite Google Earth Pro avec une haute résolution jusqu'au niveau de la structure. Ces cartes satellites aideront à localiser précisément les points sélectionnés sur le terrain. Des récepteurs GPS (Global Positioning System) portables (Garmin-eTrex, Garmin Ltd., Lenexa, KS, USA) aide à vérifier les emplacements. Les récepteurs seront positionnés près d'un point localisé, dans une position statique et en plein air pour assurer une lecture sans obstacle. En cas de refus, d'absence du répondant ou de localisation d'un point non résidentiel, un point résidentiel le plus proche de la droite ou de la gauche sera visité (32).

Fréquence de la collecte de données

Les points résidentiels seront visités deux fois pour des entrevues au cours du programme SETA pour prendre en compte un possible impact lié à la saison, sur le comportement sanitaire (33-37). Les enquêtes auront lieu à différentes saisons - une à la fin de la saison sèche ou au moins un mois après son début et une vers la fin de la saison des pluies ou au moins un mois après son début (Tableau 3). Actuellement, l'étude HPAfrica est en cours au Burkina Faso, au Ghana et à Madagascar et devrait se poursuivre dans les autres pays au cours des prochains mois.

Critères d'inclusion et d'exclusion

Les membres du ménage de tout âge et des deux sexes qui vivent dans la zone d'étude au jour de l'interview seront éligibles pour l'inclusion. Un ménage sera exclu si le répondant désigne refuse de participer ou n'est pas disponible après trois visites consécutives. Tous les visiteurs et individus dont le lieu de résidence n'est pas connu ou est en-dehors de la zone d'étude seront exclus.

Pour raison de cette enquête, un ménage est défini comme une personne ou un groupe de personnes apparentées ou non apparentées vivant dans la même unité d'habitation, reconnaissant un individu adulte comme chef du ménage, partageant les mêmes installations domestiques, et subvenant indépendamment à leurs besoins en termes de nourriture et des autres éléments essentiels pour vivre (26).

L'interview aura lieu exclusivement avec le répondant, qui est un membre adulte du ménage à l'âge de majorité légale spécifique au pays. Cette personne peut être identifiée comme décideur par les membres du même ménage et sert de substitut pour un ménage entier (15). D'autres définitions pertinentes sont expliquées subséquemment.

Collecte de données

Les intervieweurs de site recevront une formation sur la localisation des points géographiques, l'identification des répondants, les procédures de consentement éclairé en mettant l'accent sur la participation volontaire, et sur le déploiement de formulaires d'étude standardisés et pré-testés avant le lancement de HPAfrica. Les formulaires de consentement éclairé et d'étude seront traduits dans la langue officielle du pays et retraduits en anglais pour prendre en compte des erreurs de traduction et apporter des corrections, le cas échéant.

Gestion de données

For peer review only - http://bmjopen.bmj.com/site/about/guidelines.xhtml

La gestion des données dépendra du mode de collecte, qui sera principalement électronique plutôt que sur support papier. Les expériences passées ont montré les inconvénients suivants de la collecte de données sur papier: une forte probabilité d'erreurs lors du remplissage des formulaires d'étude, et le besoin de numérisation des données, qui est une étape supplémentaire, laborieuse, qui consomme du temps et sujette aux erreurs. La capture électronique de données à l'aide d'une application pour smartphone ou tablette réduit ces facteurs limitants à la qualité des données.

Il peut y avoir une période pilote au cours de laquelle des données sur support papier et sans papier seront collectées avant la mise en œuvre complète de la collecte de données électroniques. Ce dernier utilise l'application Android "HPA Collect" (Google Android 5.0.1 9API 23, disponible à l'adresse https://play.google.com/store/apps/details?id=anint.ivi.hpa) et la plate-forme "HPA Web" (CSS, JavaScript et JSP, disponible sur http://hpa.ivi.int/), qui sont tous deux accompagnés d'un dictionnaire de variables contenant les codes, la signification et les propriétés des variables au moins en anglais et en français. Une connectivité internet sans fil stable utilisant des navigateurs par défaut est nécessaire pour assurer une synchronisation simultanée, attribuable, originale et précise des données entre "HPA Collect" et "HPA Web". L'accès à "HPA Collect" et "HPA Web" est protégé par un mot de passe. L'annexe 5 affiche le diagramme du système, qui a les caractéristiques suivantes:

Structure ou fonctionnalités de "HPA Collect"

- **Gestion des utilisateurs:** Une interface utilisateur simple fournit différents privilèges aux utilisateurs pour entrer, modifier (sauf pour les étiquettes d'étude) et exporter ou transférer des données. Il établit une piste d'audit qui enregistre lorsque les utilisateurs entrent des données dans le serveur.
- **Saisie de données:** Les données sont entrées directement dans le smartphone ou la tablette. Les champs de saisie limitent les réponses possibles à des réponses plausibles uniquement en offrant différentes méthodes de saisie:
  - Modifier le texte: texte libre
  - Groupe radio / bouton Multi-clic: choix unique (radio group/Multi click button)
  - Case à cocher: choix multiple
  - Sélecteur de date et d'heure: date et heure d'entrée
  - Spinner: bouton déroulant

  Des fonctionnalités supplémentaires permettent à l'enquêteur de gagner du temps en transférant les données collectées plus tôt aux champs de données requis à un stade ultérieur de l'interview en effectuant des vérifications de vraisemblance pour divers champs de saisie de données et en reconnaissant facilement les modèles de saut. Les vérifications d'erreurs, les notifications de données manquantes, les erreurs de code et les erreurs logiques sont également incluses.
- **Vue des données:** Les données brutes entrées à l'origine peuvent être vues dans le même format lisible par l'homme et la machine, à savoir le format JSON, étant donné qu'elles seront transférées vers un serveur de base de données (Windows, MySQL). Des fonctions supplémentaires telles que «effacer» pour effacer tous les enregistrements, «réinitialiser» pour revenir à la page d'entrée de données principale, et «rechercher» pour rechercher une étiquette d'étude sont disponibles.
- **Paramètres:** la possibilité de changer la langue du système entre l'anglais et le français est fournie.
- **À propos de:** La version actuellement installée de «HPA Collect» est affichée.

Structure ou caractéristiques de "HPA Web"

- **Accueil:** une introduction à la plateforme est donnée et un tutoriel en français et en anglais sera accessible.
- **Aperçu du Formulaire:** Les formulaires d'étude sont affichés séparément montrant chaque question et les données d'entrée correspondantes en tant que variables définies.

For peer review only - http://bmjopen.bmj.com/site/about/guidelines.xhtml

- **Modifier le formulaire:** cette fonction permet la correction des données téléchargées. Il est uniquement accessible aux gestionnaires de données de chaque pays participant et à IVI.
- **Cherche:** Les étiquettes d'étude spécifiques données à chaque formulaire d'étude peuvent être filtrées.
- **Dictionnaire des variables:** Le dictionnaire variable peut être téléchargé.
- **Exporter:** Les données peuvent être converties à partir du serveur dans un fichier Excel transférable.
- **Contactez-nous:** Une correspondance rapide et facile entre les utilisateurs et le gestionnaire de données à IVI est donnée.

Les données seront examinées quotidiennement par le personnel clé de l'étude et vérifiées en termes de consistance et d'exactitude avant l'analyse des données. La qualité des données peut être vérifiée en sélectionnant un sous-ensemble de 5% pour la validation par rapport aux données originales sur support papier, si possible. Les noms des membres du ménage ne seront pas liés aux formulaires d'étude de HPAfrica et ne seront pas enregistrés dans la base de données afin d'assurer la confidentialité. L'accès à la base de données sera limité au personnel d'étude autorisé uniquement et les données seront conservées dans un endroit sécurisé et protégé. Des fichiers de sauvegarde provisoires périodiques et au moins trois fichiers de sauvegarde définitifs de la base de données stockés dans un endroit sécurisé et verrouillé éviteront la perte de données et garantiront la sécurité des données. Toutes les données seront conservées pendant au moins cinq ans.

L'analyse des données

Des analyses descriptives telles que les fréquences absolues et relatives, les médianes ou les moyennes arithmétiques seront effectuées pour construire une description contemporaine de la population de base.

Les analyses de base comprendront le calcul des proportions stratifiées selon l'âge et le sexe, des comportements sanitaires *génériques* et *réels*, y compris l'utilisation des soins de santé, pour divers signes et symptômes ou combinaisons de signes et de symptômes. Ces analyses permettront une classification graduelle basée sur la sévérité de la maladie, incluant un intervalle de confiance de 95%. Le comportement de santé sera ajusté pour un effet de regroupement naturel présumé parmi les membres du ménage. Des données stratifiées les plus à jour sur la population par site et les données démographiques dérivées de HPAfrica seront utilisées pour générer les proportions pour l'ajustement des calculs d'incidence.

Les informations socio-économiques collectées seront utilisées pour construire des indices de richesse des populations étudiées en appliquant des analyses factorielles basées sur la méthode des composantes principales (38). De plus, des analyses de régression bi et multivariée seront effectuées pour identifier les facteurs de confusion potentiels ou la modification des effets en utilisant les scores factoriels.

Les analyses de régression bi et multivariée seront effectuées en utilisant les scores factoriels pour l'évaluation des variables suivantes:

- Données socioéconomiques telles que l'éducation, le revenu, l'assurance maladie, le logement et les biens des ménages
- Hygiène et assainissement tels que l'accès aux toilettes, les sources d'eau, la manipulation des aliments, l'élimination des déchets et les contacts avec les animaux
- Comportement sanitaire: *réel* (stratifié selon l'âge et le sexe, gravité, fréquence), *générique* (stratifié selon l'âge et le sexe, gravité, classement par choix)
- Modalités de déplacement vers les établissements de santé, stratifiées selon l'âge
- Saisonnalité liée à l'hygiène et à l'assainissement, comportement sanitaire, modalités de déplacement
- Facteurs culturels / religieux / ethnographiques, stratifiés selon l'âge
- Statut de vaccination parmi les enfants ≤ 6 ans (18, 19)
- Connaissance et perception des maladies courantes

For peer review only - http://bmjopen.bmj.com/site/about/guidelines.xhtml

Considérations éthiques
Il n'y a pas d'avantages directs pour les ménages inscrits et leurs membres. Les avantages indirects pour la population générale sur les sites seront les informations mises à jour sur le fardeau des maladies transmissibles, y compris les facteurs influençant la transmission de la maladie et l'infection. L'étude HPAfrica génère des évidences pour aider les décideurs à introduire des mesures appropriées pour la prévention et le contrôle des maladies.

Ce protocole a fait l'objet d'examens par le Comité de Révision Institutionnelle d'IVI et des comités locaux spécifiques aux sites, comme indiqué dans la section «Références éthiques». Pendant et après l'étude HPAfrica, toutes les données des sujets inscrits seront gardés avec une stricte confidentialité et ne seront pas divulguées à une tierce partie par aucun membre de l'équipe de recherche. La protection par mot de passe des appareils et de la base de données est utilisée pour une stricte confidentialité. Toutes les données sur support papier (formulaires de consentement) seront conservées dans un endroit sécurisé et verrouillé. Les informations confidentielles stockées sur des ordinateurs et des sources de données sur support papier seront seulement mises à la disposition des co-chercheurs et du personnel d'IVI directement impliqués dans les activités d'étude de HPAfrica.

Abbréviations
JSON: JavaScript Objective Notation: CSS: Cascading Style Sheets: JSP: Java Server Page; SQL: Structured Query Language

For peer review only

For peer review only - http://bmjopen.bmj.com/site/about/guidelines.xhtml

Définitions/Terminologie

**A**	
Automédication	Fait de se médicamenter soi-même ou de traiter sa propre maladie ou son état sans supervision médicale ou ordonnance.
**B**	
**C**	
Choléra	Maladie infectieuse de l'intestin grêle qui peut être mortelle causée par la bactérie *Vibrio cholerae* ; elle se propage par voie fécale-orale par l'ingestion d'eau et d'aliments contaminés et provoque fièvre, diarrhée aqueuse sévère, vomissements et déshydratation.
Confusion/Vertiges/Perte de connaissance	L'état de désorientation ou d'absence de clarté d'esprit ; l'état anormal de réponse aux stimuli dans l'environnement; faible, wooziness, faiblesse, instabilité ou tendance à tomber; l'interruption de la conscience de soi et de son environnement.
Convulsion	Mouvement soudain, violent et irrégulier d'un membre/d'une partie du corps ou de tout le corps causé par une contraction involontaire des muscles.
Coqueluche (Pertussis)	La coqueluche est une maladie bactérienne très contagieuse. Les symptômes d'un froid commun comme nez qui coule, fièvre et toux sont suivis par des semaines de toux sévère et un son aigu de la coqueluche ou haletant quand une personne malade inspire; toux sévère peut être suivie de vomissements, rupture des côtes ou fatigue extrême. Les très jeunes et les personnes âgées infectées peuvent avoir peu ou pas de toux, ou peuvent avoir des périodes de respiration interrompue pendant lesquelles elles ne respirent pas. La coqueluche est transmise par la bactérie *Bordetella pertussis* par toux et éternuements d'une personne infectée.
**D**	
Déshydration	Une condition de perte excessive d'eau/d'hydratation par le corps.
Diarrhée	Une condition d'évacuer les matières fécales du corps très fréquemment et sous forme plutôt liquide que solide.
Difficultés respiratoires/ Respiration sévère	Une condition de la respiration anormale accompagnée d'une respiration sifflante, d'une douleur thoracique, d'un tirage thoracique, d'un stridor, d'un gonflement de la poitrine ou d'une toux par exemple.
Diphthérie	Maladie hautement contagieuse, parfois mortelle, causée par la bactérie *Corynebacterium diphtheriae*. Elle se transmet de personne à personne, par l'air ou les objets contaminés et affecte les voies respiratoires supérieures. Les symptômes courants sont maux de gorge, gonflement des glandes/ ganglions lymphatiques, malaise, fièvre/frissons, substance membraneuse/matière grise et épaisse qui se forme dans la gorge/sur les amygdales et gêne/accélère la respiration (toux rauque) et gêne pour avaler. Une forme de diphtérie qui implique la peau, les yeux et les organes génitaux est également signalée et se caractérise par une peau douloureuse, rouge et enflée.
Douleur abdominale/ Douleur intestinale	Douleur (aiguë/courte durée ou chronique/longue durée) qui survient entre la poitrine et l'aine (souvent désignée comme la région de l'estomac/du ventre)
Douleur intestinale/Douleur abdominale	Douleur (aiguë/courte durée ou chronique/longue durée) qui survient entre la poitrine et l'aine (souvent désignée comme la région de l'estomac/du ventre)
Douleur/Maux	État caractérisé par une douleur sourde continue/prolongée dans une partie du corps ; la sensation/souffrance/inconfort physique causée par

For peer review only - http://bmjopen.bmj.com/site/about/guidelines.xhtml

	une maladie/une blessure/quelque chose qui fait mal au corps.
**E**	
Éducation formelle	Modèle d'éducation systématique et organisée structuré et administré selon un ensemble de lois et de normes ; les institutions d'éducation formelle sont organisées de façon administrative, physique et pédagogique et exigent des étudiants une assiduité minimum ; il implique des évaluations intermédiaires et finales afin d'accéder au niveau d'apprentissage suivant ; il délivre des certificats et des diplômes conformément à un ensemble de règles strictes.
Essoufflement/ Respiration rapide	Fait de respirer anormalement rapidement et profondément ; à une fréquence de >20 respirations par minute pour les adultes, >30 respirations par minute pour les enfants et >45 respirations par minute par les bébés.
Établissement de santé	Lieux ou institutions où des soins de santé sont prodigués ; dont les hôpitaux, les cliniques, les centres de santé, les postes de santé et les centres de soins spécialisé.
Éternuement	Fait d'expulser soudainement l'air par le nez et la bouche avec un bruit important caractéristique.
**F**	
Faiblesse/Malaise/Fatigue	État de se sentir fatigué/épuisé/à bout de forces ; sentiment général d'inconfort/malaise/de ne pas être en bonne santé ou heureux.
Fatigue/Malaise/ Faiblesse	État de se sentir fatigué/épuisé/à bout de forces ; sentiment général d'inconfort/malaise/de ne pas être en bonne santé ou heureux.
Fièvre	Température corporelle anormalement élevée (habituellement au-dessus de 38°C).
Fièvre <3 jours	Température corporelle anormalement élevée (habituellement au-dessus de 38°C) qui dure moins de 3 jours et apparaît en continu.
Fièvre >3 jours	Température corporelle anormalement élevée (habituellement au-dessus de 38°C) qui dure plus de 3 jours et apparaît en continu.
Fièvre jaune	Maladie infectieuse aiguë, pathogène pour l'homme et virale caractérisée par l'apparition soudaine de symptômes comme fièvre, frissons, perte d'appétit, nausée, douleurs musculaires et maux de tête qui disparaissent généralement en quelques jours ou sont suivis de symptômes plus graves comme jaunisse, fièvre forte et hémorragie. La maladie est causée par un virus du genre Flavivirus et est transmise par la piqûre d'un moustique infecté.
Fièvre typhoïde	Maladie infectieuse bactérienne potentiellement mortelle causée par *Salmonella typhi* ; elle se transmet par la voie fécale-orale par les aliments et l'eau contaminés ou de personne à personne. Les symptômes courants sont forte fièvre, maux de tête, douleur abdominale, constipation/diarrhée et éruption cutanée. Des complications graves (perforation de l'intestin) sont rapportées.
Frissons	Sensation de froid qui peut être accompagnée de frissons/tremblements du corps et de pâleur de la peau
**G**	
Gonflement/Œdème	État d'accumulation excessive de liquide aqueux/gonflement des cavités ou des tissus du corps ; couramment constaté dans les mains/bras/pieds/jambes ; augmentation du volume de tissu.
Grippe	Maladie infectieuse hautement contagieuse des voies respiratoires causée par le virus de la grippe ; elle se propage de personne à personne par les particules expulsées par la toux/les éternuements/la parole et les objets contaminés. Les symptômes courants sont fièvre,

For peer review only - http://bmjopen.bmj.com/site/about/guidelines.xhtml

	douleurs dans les muscles/bras/jambes, frissons/sueur, maux de tête, fatigue/faiblesse, nez qui coule/congestion nasale, éternuements, maux de gorge et toux sèche et persistante.
Guérisseur traditionnel	Personne qui cherche à maintenir la santé aussi bien qu'à prévenir, diagnostiquer et améliorer ou traiter les maladies en utilisant un ensemble de connaissances, compétences et pratiques basées sur des théories, des croyances et des expériences.
**H**	
Haemophilus influenzae de type B	Bactérie qui provoque une série de maladies et se propage par les particules expulsées par la toux ou les éternuements, et surviennent surtout chez l'enfant de moins de 5 ans ; les symptômes courants sont fièvre, maux de tête et raideur dans le cou jusqu'à l'état septique. Elle peut causer otites, cellulites (infection des tissus mous), arthrite, infections des voies respiratoires supérieures, pneumonies, méningites (avec dommages potentiels au cerveau) et épiglottites (avec obstruction des voies aériennes).
Hémorragie (interne/externe)/ Perte de sang	État caractérisé par une perte de sang du système vasculaire, soit de façon interne à l'intérieur du corps (hémorragie interne) soit de façon externe par un orifice naturel ou une lésion de la peau (hémorragie externe) ; sang qui s'échappe d'un vaisseau sanguin endommagé.
Hépatite B	Maladie infectieuse causée par le virus de l'hépatite B (VHB) qui affecte le foie (infection hépatique aiguë/chronique ; peut entraîner une insuffisance hépatique/un cancer/une cirrhose). Le virus se transmet de personne à personne par le sang/le sperme/les autres fluides corporels. Les symptômes courants sont fièvre, malaise/faiblesse/fatigue, perte d'appétit, nausée/vomissements, gêne/douleur abdominale, urines foncées, douleurs articulaires et jaunisse.
Hépatite E	Maladie infectieuse causée par le virus de l'hépatite E (VHE) qui affecte le foie (infection hépatique aiguë uniquement ; peut entraîner une insuffisance hépatique aiguë). Le virus se propage par les voies fécales-orales par l'ingestion d'eau ou d'aliments contaminés, les animaux contaminés (de façon zoonotique) et par le sang. Les symptômes courants sont fièvre, malaise, perte d'appétit, diarrhée, nausée, gêne abdominale, urines foncées, douleurs articulaires et jaunisse.
**I**	
**J**	
Jaunisse	Maladie qui fait jaunir la peau/le blanc des yeux.
**K/L**	
**M**	
Malaise/Fatigue/ Faiblesse	État de se sentir fatigué/épuisé/à bout de forces ; sentiment général d'inconfort/malaise/de ne pas être en bonne santé ou heureux.
Maux de tête	Douleur continue dans une région de la tête.
Maux/Douleur	État caractérisé par une douleur sourde continue/prolongée dans une partie du corps ; la sensation/souffrance/inconfort physique causée par une maladie/une blessure/quelque chose qui fait mal au corps.
Médecin	Une personne compétente/formée aux soins ; une personne éduquée, cliniquement expérimentée et autorisée à pratiquer la médecine.
Ménage	Personne ou groupe de personnes apparentées ou non qui vivent ensemble dans la même unité d'habitation, qui reconnaissent un adulte de sexe masculin ou féminin comme le chef du ménage, qui partagent

For peer review only - http://bmjopen.bmj.com/site/about/guidelines.xhtml

	les mêmes installations domestiques, qu'on considère comme formant une unité, et qui subviennent à leurs besoins en termes de nourriture et des autres éléments essentiels pour vivre. Un ménage peut être situé dans une unité d'habitation unique (bâtiment à un seul étage) ou dans un ensemble de logements collectifs (bâtiment à plusieurs étages).
Méningite	Inflammation virale/bactérienne/fongique infectieuse parfois mortelle des membranes (méninges) qui entourent le cerveau et la moelle épinière ; elle se propage de personne à personne par les particules expulsées par la toux/les éternuements/la parole et les objets contaminés. Les symptômes courants sont forte fièvre, maux de tête sévères, nausée/vomissements, confusion, convulsions ; perte d'appétit et de la soif, fatigue, sensibilité à la lumière, éruption cutanée et raideur au cou.
**N**	
Nausée/Vomissements	Sensation de malaise dans l'estomac avec tendance à vomir ; fait d'éjecter une partie/tout le contenu de l'estomac principalement par la bouche.
Nez qui coule	État résultant de la production par le nez (tissus nasaux et adjacents et vaisseaux sanguins) de mucus/drainage excessif, qui coule du nez pour retirer quelque chose.
**O**	
Œdème/Gonflement	État d'accumulation excessive de liquide aqueux/gonflement des cavités ou des tissus du corps ; couramment constaté dans les mains/bras/pieds/jambes ; augmentation du volume de tissu.
Oreillons	Maladie infectieuse virale causée par le virus des oreillons qui affecte principalement les glandes parotides qui sont situées sous et devant les oreilles ; elle se propage de personne à personne par la salive infectée/les particules de salive infectées expulsées par la toux/les éternuements/la parole et les objets contaminés. Les symptômes courants sont fièvre, gonflement d'une/des deux glandes parotides, maux de tête ; douleurs musculaires, faiblesse/fatigue, perte d'appétit et douleurs en mâchant/avalant ; une complication connue est une possible perte d'audition.
**P**	
Paludisme	Maladie infectieuse parfois mortelle causée par un parasite (*Plasmodium* spp.) transmis principalement par la piqûre de moustiques infectés, en dehors de la transmission mère-enfant et par le sang. Les symptômes courants sont attaques récurrentes de frissons, sueurs, forte fièvre, maux de tête, vomissements et diarrhée.
Personne interrogée	Membre adulte (à l'âge légal de majorité du pays concerné) du ménage qui est un décideur en ce qui concerne les soins de santé/l'utilisation des soins de santé pour le ménage entier à l'intérieur du site d'étude ; cette personne adulte parle ainsi au nom de tous les membres du ménage. Cette personne peut être identifiée par les autres membres du même ménage comme la personne qui est principalement impliquée dans les soins quotidiens des membres du ménage.
Perte de connaissance/ Vertiges/Confusion	L'état de désorientation ou d'absence de clarté d'esprit ; l'état anormal de réponse aux stimuli dans l'environnement; faible, wooziness, faiblesse, instabilité ou tendance à tomber; l'interruption de la conscience de soi et de son environnement.
Perte de poids	Baisse (volontaire/involontaire) du poids du corps.
Perte de sang/	État caractérisé par une perte de sang du système vasculaire, soit de

For peer review only - http://bmjopen.bmj.com/site/about/guidelines.xhtml

Hémorragie	façon interne à l'intérieur du corps (hémorrage interne) soit de façon externe par un orifice naturel ou une lésion de la peau (hémorragie externe) ; sang qui s'échappe d'un vaisseau sanguin endommagé.
Pharmacie	Endroit où seuls des médicaments (ordonnance requise/non requise) sont préparés, conservés, composés et dispensés.
Pneumocoque	Une inflammation infectieuse bactérienne des poumons parfois mortelle qui affecte souvent les personnes immunodéprimées et est causée par la bactérie *Streptococcus pneumoniae* ; elle se propage de personne à personne par les particules expulsées par la toux/les éternuements/la parole et les objets contaminés. Les symptômes courants sont fièvre, frissons, toux (avec mucosités/pus), douleurs thoraciques, difficulté à respirer/essoufflement, fatigue, nausée/vomissements et diarrhée ; des symptômes rares sont septicémie, épanchement pleural et empyème.
Polio	Maladie virale infectieuse, très contagieuse, parfois mortelle, causée par le poliovirus qui cause dans sa forme la plus sévère paralysie et difficultés à respirer ; il se transmet par la voie fécale-orale par ingestion d'eau et d'aliments contaminés et de personne à personne. Les symptômes courants sont fièvre, maux de gorge, maux de tête, vomissements, fatigue, maux de dos, de cou/raideur au cou, faiblesse des muscles/muscles douloureux, méningite, perte de réflexes, membres lourds/pendants.
Occupation	A job, or means of earning a wage/living; often requires the mastery of a complex set of knowledge, tasks, duties and skills acquired through formal education and/or practical experience.
**Q**	
**R**	
Respiration rapide/ Essoufflement	Fait de respirer anormalement rapidement et profondément ; à une fréquence de >20 respirations par minute pour les adultes, >30 respirations par minute pour les enfants et >45 respirations par minute par les bébés.
Respiration sévère/ Difficultés respiratoires	Une condition de la respiration anormale accompagnée d'une respiration sifflante, d'une douleur thoracique, d'un tirage thoracique, d'un stridor, d'un gonflement de la poitrine ou d'une toux par exemple.
Revenus/Salaire	Argent ou autre forme de paiement que quelqu'un reçoit périodiquement/régulièrement en échange de biens ou de services. Provient généralement d'un emploi/d'une profession.
Rotavirus	Infection virale causée par le rotavirus qui cause principalement des diarrhées pour les enfants de moins de 5 ans ; elle se propage par la voie fécale-orale, de personne à personne et par les objets contaminés. Les symptômes courants sont diarrhée aqueuse, fièvre, vomissements, douleurs abdominales et déshydratation.
Rougeole	Maladie infectieuse parfois mortelle des voies respiratoire (nez/gorge) causée par un virus qui affecte principalement les enfants de moins de 5 ans ; elle se propage de personne à personne par les particules expulsées par la toux/les éternuements/la parole et les objets contaminés. Les symptômes courants sont fièvre, toux sèche, nez qui coule, inflammation des yeux, maux de gorge, larges plaques sur la peau et points blancs dans la bouche et sur les joues.
Rubéole	Maladie infectieuse virale contagieuse connue pour son éruption cutanée rouge distinctive qui est causée par le virus de la rubéole ; elle se transmet de personne à personne (toux/éternuements/parole), de la mère à l'enfant par le sang et les objets contaminés. Les symptômes courants sont fièvre modérée, maux de tête, nez bouché/qui coule,

For peer review only - http://bmjopen.bmj.com/site/about/guidelines.xhtml

	yeux rouges/inflammation des yeux, ganglions lymphatiques gonflés et sensibles à la base du crâne/à l'arrière du cou/derrière les oreilles, douleurs articulaires et éruption cutanée rose qui se déplace du visage au torse, aux bras et aux jambes.
**S**	
Soins de santé - généraux	Soins de santé pour les maladies qui n'ont aucun lien avec l'apparition de maladies associées aux signes et symptômes ou les dates de début de maladies.
Soins de santé - réels	Soins de santé liés l'apparition de maladies associées aux signes et symptômes ou les dates de début de maladies.
**T**	
Témoin	Personne adulte (à l'âge légal de majorité du pays concerné), qui est indépendante de l'étude, qui ne peut pas être indûment influencée par le personnel de l'étude, qui participe au processus de consentement éclairé si le sujet/le représentant légal du sujet est analphabète, et qui lit (lettré) le formulaire de consentement éclairé et toute autre information écrite présentée au sujet.
Tétanos	Une maladie infectieuse caractérisée par des spasmes musculaires qui commencent généralement dans la mâchoire et progresse vers le reste du corps. Les spasmes durent généralement quelques minutes et se produisent fréquemment pendant les 3-4 semaines au début de la maladie, ils peuvent être si graves qu'ils peuvent causer des fractures osseuses ou la mort lorsqu'ils affectent les muscles respiratoires. Elle peut être caractérisée par d'autres symptômes comme fièvre, sueurs, maux de tête, difficultés à avaler, pression artérielle élevée et augmentation du rythme cardiaque. La maladie est causée par une infection par la bactérie *Clostridium tetani* qui se trouve dans sol, saliva, poussière et fumier. La bactérie entre par une lésion cutanée (par exemple, coupure, blessure par ponction) par un objet contaminé. Il produit des toxines qui interfèrent avec les contractions musculaires qui résultent des spasmes typiques.
Toux	Fait d'expulser de façon soudaine/rapide et bruyamment l'air des poumons ; souvent de façon involontaire afin de dégager les voies respiratoires des poumons de fluides/mucus/autres matières
Troubles cardiaques	Tout trouble que affecte le cœur ; maladies qui impliquent des vaisseaux sanguins rétrécis/bloqués qui peuvent entraîner une attaque cardiaque/douleur thoracique/accident vasculaire cérébral ; maladie qui affecte le muscle/les valves/le rythme du cœur.
Troubles de la pression artérielle	Pression artérielle élevée/faible ou pression artérielle qui présente des fluctuations excessives.
Tuberculose	Maladie infectieuse bactérienne (*Mycobacterium tuberculosum*), contagieuse, potentiellement grave qui affecte principalement les poumons en plus des reins et de la colonne vertébrale/du cerveau ; elle se transmet de personne à personne par les particules expulsées par la toux/les éternuements/la parole. Les symptômes courants d'une tuberculose active sont toux (avec/sans sang), douleur thoracique, perte de poids, fatigue, fièvre, sueurs nocturnes, frissons et perte d'appétit.
**U**	
**V**	
Vertiges/Confusion/Perte de connaissance	L'état de désorientation ou d'absence de clarté d'esprit ; l'état anormal de réponse aux stimuli dans l'environnement; faible, wooziness, faiblesse, instabilité ou tendance à tomber; l'interruption de la

For peer review only - http://bmjopen.bmj.com/site/about/guidelines.xhtml

	conscience de soi et de son environnement.
VIH/SIDA	Maladie infectieuse causée par le virus de l'immunodéficience humaine (VIH) qui affecte/endommage le système immunitaire et entraîne le syndrome immunodéficitaire acquis (SIDA), une maladie chronique et potentiellement mortelle ; il se transmet par les liquides corporels infectieux comme le sang/le sperme/les autres fluides corporels. Les symptômes courants d'une infection aiguë/précoce sont fièvre, fatigue, maux de tête, douleurs articulaires, éruption cutanée, maux de gorge, ganglions lymphatiques enflés, diarrhée, perte de poids, candidose orale et zona. La maladie non traitée évolue en SIDA ; les symptômes causés par un système immunitaire gravement endommagé sont sueurs, fièvre récurrente, diarrhée chronique, lésions sur la langue et dans la bouche, fatigue, perte de poids et éruptions cutanées.
Visiteur	Personne (apparentée ou non) qui rend visite à un ménage pour passer du temps avec les membres du ménage ; un visiteur ne vit pas avec les membres du ménage dans la même unité d'habitation, ne partage pas les mêmes installations domestiques, la même nourriture et autres éléments essentiels à la vie que les membres du ménage, et ne constitue pas une unité avec les membres du ménage.
Voisin	Personne (apparentée ou non) vivant dans l'habitation à côté de celle d'un ménage cible ; un voisin ne vit pas avec les membres d'un ménage (à côté) dans la même unité d'habitation, ne partage pas les mêmes installations domestiques, aliments ou autres éléments essentiels à la vie que les membres du ménage (à côté), et ne constitue pas une unité avec les membres du ménage (à côté).
Vomissements/Nausée	Sensation de malaise dans l'estomac avec tendance à vomir ; fait d'éjecter une partie/tout le contenu de l'estomac principalement par la bouche.
**W/X/Y/Z**	

For peer review only

Formulaires de l'étude

Site ___ ___ ___ Sous-zone ___ ___ ___ Numéro d'habitation ___ ___ ___ ___ Identifiant de l'enquêteur ___ __

FORMULAIRE 1 – COMPTE RENDU DES VISITES AUX MÉNAGES

Instructions pour l'enquêteur

- Compléter « Formulaire 1 – COMPTE RENDU DES VISITES AUX MÉNAGES » pour chaque ménage visité.
- Compléter « Foyer/Personne interrogée/Complété » une fois que toutes les parties de l'enquête ont été complétées ; si toutes les données n'ont pas été recueillies lors de la visite initiale et/ou d'une visite ultérieure (max. 3 visites), programmer une nouvelle visite.
- <u>Tentative 1:</u> Compléter le statut de la visite au ménage et utilisant les choix proposés ; pour « pas à la maison », « à la maison/ne répond pas » et « à la maison/répond/indisponible », programmer une nouvelle visite ; pour « à la maison/répond/refus » indiquez la raison de refus et visitez un ménage de remplacement au plus proche à droite ou à gauche de ce ménage; pour « inexistant/pas d' habitation », passer ce ménage et visiter un ménage de remplacement au plus proche à droite ou à gauche à la place de ce ménage.
- <u>Tentative 2:</u> Compléter le statut de la visite du ménage et utilisant les choix proposés ; pour « pas à la maison », « à la maison/ne répond pas » et « à la maison/répond/indisponible », programmer une nouvelle visite.
- <u>Tentative 3:</u> Compléter le statut de la visite au ménage et utilisant les choix proposés ; pour « pas à la maison », « à la maison/ne répond pas » et « à la maison/répond/indisponible », passer ce ménage et visiter un ménage de remplacement au plus proche à droite ou à gauche à la place de ce ménage.
- « Site » et « sous-zone » doivent être un numéro à trois chiffres, « numéro d'habitation » un numéro à quatre chiffres, et « identifiant de l'enquêteur » un numéro à deux chiffres (utiliser les listes préparées individuellement pour chaque site pour obtenir les codes) ; saisir « date » au format JJ/MM/AAAA et « heure » au format HH:MM et entourer MATIN ou APRÈS-MIDI. «Étiquette de l'étude» doit être une combinaison d'un code «site» à trois chiffres, d'un code «sous-zone» à trois chiffres et d'un code «numéro d'habitation» à quatre chiffres; le « identifiant de l'enquêteur » doit être un numéro à trois chiffres.

**Tentative 1**	Date ____/____/____ (JJ/MM/AA)	Heure _____/_____ MATIN/APRÈS-MIDI	Identifiant de l'enquêteur ___ ___
STATUT		Cocher un	
1=Pas à la maison		O	➡ Planifier nouvelle visite (date/heure)
2=À la maison/ Non-répondant		O	➡ Planifier nouvelle visite (date/heure)
3=À la maison/ répondant /Indisponible		O	➡ Planifier nouvelle visite (date/heure)
4= À la maison/ Répondant /Complété		O	
5= À la maison/ répondant /Refus		O	Notez la/les raison(s) du refus; cochez tout ce que s'applique:<br>O 1=Pas de temps pour répondre aux questions<br>O 2=Ne veulent pas participer/répondre aux questions<br>O 3=Autre ______________________<br>O 99=Pas de réponse O 98=Ne sait pas<br>➡ Visitez un ménage de remplacement le plus proche de ce ménage du côté droit ou gauche
6=Inexistant/Pas une habitation		O	➡ Visitez un ménage de remplacement le plus proche de ce ménage du côté droit ou gauche

For peer review only - http://bmjopen.bmj.com/site/about/guidelines.xhtml

Tentative 2	Date ____/____/____ (JJ/MM/AA)	Heure _____/_____ MATIN/APRÈS-MIDI	Identifiant de l'enquêteur ___ ___
STATUT		Cocher un	
1=Pas à la maison		○	➡ Planifier nouvelle visite (date/heure)
2=À la maison/Ne répond pas		○	➡ Planifier nouvelle visite (date/heure)
3=À la maison/Répond/Indisponible		○	➡ Planifier nouvelle visite (date/heure)
4= À la maison/Répond/Complété		○	
5= À la maison/ répondant /Refus		○	Notez la/les raison(s) du refus; cochez tout ce que s'applique:<br>○ 1=Pas de temps pour répondre aux questions<br>○ 2=Ne veulent pas participer/répondre aux questions<br><br>○ 3=Autre ______________________________<br>○ 99=Pas de réponse ○ 98=Ne sait pas<br><br>➡ Visitez un ménage de remplacement le plus proche de ce ménage du côté droit ou gauche
6=Inexistant/Pas une habitation		○	➡ Visitez un ménage de remplacement le plus proche de ce ménage du côté droit ou gauche
**Tentative 3**	Date ____/____/____ (JJ/MM/AA)	Heure _____/_____ MATIN/APRÈS-MIDI	Identifiant de l'enquêteur ___ ___
STATUT		Cocher un	
1=Pas à la maison		○	➡ Visitez un ménage de remplacement le plus proche de ce ménage du côté droit ou gauche
2=À la maison/Ne répond pas		○	➡ Visitez un ménage de remplacement le plus proche de ce ménage du côté droit ou gauche
3=À la maison/Répond/Indisponible		○	➡ Visitez un ménage de remplacement le plus proche de ce ménage du côté droit ou gauche
4= À la maison/Répond/Complété		○	
5= À la maison/ répondant /Refus		○	Notez la/les raison(s) du refus; cochez tout ce que s'applique:<br>○ 1=Pas de temps pour répondre aux questions<br>○ 2=Ne veulent pas participer/répondre aux questions<br><br>○ 3=Autre ______________________________<br>○ 99=Pas de réponse ○ 98=Ne sait pas<br><br>➡ Visitez un ménage de remplacement le plus proche de ce ménage du côté droit ou gauche
6=Inexistant/Pas une habitation		○	➡ Visitez un ménage de remplacement le plus proche de ce ménage du côté droit ou gauche

For peer review only

Site ___ ___ ___ Sous-zone ___ ___ ___ Numéro d'habitation ___ ___ ___ ___ Identifiant de l'enquêteur ___ __

FORMULAIRE 2 – FORMULAIRE DE CONSENTEMENT

Evaluation des soins de santé à [site SETA and –pays]

Instructions à l'enquêteur

- Lire le "Formulaire 2 – FORMULAIRE DE CONSENTEMENT" pour le répondant/demander au répondant de lire attentivement le "Formulaire 2 – FORMULAIRE DE CONSENTEMENT" s'il préfère.
- Le répondant tel que défini dans cette enquête est un adulte (ayant l'âge de la majorité spécifique au pays) membre du ménage et responsable des décisions journalières concernant la santé des membres du ménage; il ne doit pas par exemple être un voisin ou un visiteur.
- S'assurer que le répondant a pleinement compris l'objectif, les procédures, les risques et les bénéfices de cette étude avant d'obtenir son consentement à participer.
- Le répondant doit signer le "Formulaire 2 – FORMULAIRE DE CONSENTEMENT"; si le répondant est incapable de signer/analphabète, une empreinte digitale servira en lieu et place de la signature, et un adulte lettré indépendant de l'étude qui servira de témoin (un membre du même ménage ou un voisin par exemple) signera le "Formulaire 2 – FORMULAIRE DE CONSENTEMENT".
- Collecter les informations sur tous les membres du ménage indépendamment du fait qu'ils soient présents ou non au moment de l'entretien; Recueillir l'information sur seulement les individus présents au moment de l'entretien résultera en un biais dans les données.
- Remplir le "Formulaire 2 – FORMULAIRE DE CONSENTEMENT" pour chaque ménage consentant enrôlé dans l'étude.
- Expliquer au répondant qu'il est prévu de revisiter chaque ménage durant une période de 2 ans; a chaque visite, l'équipe de recherche obtiendra un nouveau consentement.
- «Site» et «sous-zone» doivent être un numéro à trois chiffres, «numéro d'habitation» un numéro à quatre chiffres, et «identifiant de l'enquêteur» un numéro à deux chiffres (utiliser les listes préparées individuellement pour chaque site pour obtenir les codes); saisir «date» au format JJ/MM/AAAA et «heure» au format HH:MM et entourer MATIN ou APRÈS-MIDI. «Étiquette de l'étude» doit être une combinaison d'un code «site» à trois chiffres, d'un code «sous-zone» à trois chiffres et d'un code «numéro d'habitation» à quatre chiffres; le «identifiant de l'enquêteur» doit être un numéro à trois chiffres.

Investigateur principal et co-investigateur de l'étude

[Ajouter les noms de l'investigateur principal et des co-investigateurs de l'étude ainsi que leurs affiliations respectives]

Introduction

L'Institut International des Vaccins (IVI) et [ajouter l'institution d'affiliation des investigateurs locaux] travaillent ensemble pour examiner les problèmes de santé et le comportement de santé dans votre communauté [ajouter le site SETA-pays], y compris le statu socio-économique, le comportement d'hygiène et d'assainissement et le statut vaccinal des enfants. Nous aimerions mieux comprendre où vous, votre famille et vos voisins cherchez des soins de santé en cas de maladies causant la fièvre/corps chaud et autres chante & symptômes. Cette information nous aidera à savoir à quelle fréquence vous souffrez de maladies causant de la fièvre et les autres liées à la fièvre, fournir une meilleure image des facteurs influençant la transmission de la maladie et l'infection, et à trouver des moyens pour les prévenir et traiter dans votre communauté. Vous, en tant que responsable principal pour le comportement de santé/l'utilisation des soins de santé quotidienne pour les membres de ce ménage, êtes invités à participer parce que vous vivez dans la localité où nous effectuons cette étude. Ce formulaire de consentement sera lu par vous ou pour vous et vous aurez le temps pour prendre votre décision d'accepter ou de refuser de participer à l'étude. S'il vous plaît, demandez aux membres de l'étude d'expliquer tout mot ou toute information que vous ne comprenez pas. On vous donnera en conséquence plus d'informations sur cette étude, y compris ses risques potentiels, les avantages et les inconvénients. Après vous avoir entièrement expliqué l'étude et si vous acceptez de participer, on vous demandera de signer ce formulaire de consentement au nom de tous les membres du ménage. Si vous ne pouvez pas signer le formulaire, nous vous demandons de fournir votre empreinte digitale, et une personne adulte indépendante de l'étude (membre de votre ménage/voisin) signera le formulaire en tant que témoin. Nous allons vous remettre une copie signée et datée du formulaire de consentement.

Objectif

L'objectif de cette étude est d'examiner les problèmes de santé et l'utilisation des soins de santé dans votre communauté [ajouter le site SETA-pays], y compris le statu socio-économique et le comportement d'hygiène et d'assainissement. Notre objectif est de mieux comprendre où vous, votre famille et vos voisins cherchez les soins de santé pour des maladies causant de la fièvre/corps chaud et autres chante & symptômes. Cette

For peer review only - http://bmjopen.bmj.com/site/about/guidelines.xhtml

information permettra de mieux estimer le fardeau des maladies communes et d'identifier les moyens pour la mise en place de mesures de prévention et de traitement pour les maladies.

Procédures

Nous visiterons environ [ajouter le nombre pour site d'étude et pays] ménages au total à [ajouter le site et pays] et poserons les mêmes questions dans chaque ménage. Votre ménage a été choisi pour cette étude simplement par hasard. Si vous acceptez de participer, nous vous poserons, au nom de tous les membres du ménage, quelques questions au sujet de votre ménage tels que les initiales, le sexe et l'âge de chaque membre. Nous aimerions savoir certaines informations relatives à l'éducation et l'occupation du chef de ménage, au revenu, à la possession de biens, au logement lui-même ainsi qu'à l'hygiène et à l'assainissement dans le contexte de l'hygiène personnelle, de la préparation et de la manipulation des aliments, des sources d'eau et de la manipulation, de l'élimination des déchets, de la manipulation des animaux de ferme et domestiques ainsi que de l'élevage de ce ménage. Nous allons également demander où les membres de votre ménage obtiennent les soins si quelqu'un a une maladie causant de la fièvre ou toute autre maladie et les facteurs qui peuvent influer sur l'utilisation des soins de santé des membres du ménage (c'est-à-dire les modalités de transport, la disponibilité d'assurance maladie, les facteurs culturels) en plus du statut vaccinal et de la perception de certaines maladies. Enfin, nous allons mesurer la position géographique de votre maison. Il n'y a pas de bonnes ou de mauvaises réponses. Vous êtes libre de choisir les questions à répondre. Somme toute, il devrait prendre environ 30-45 minutes pour répondre à toutes les questions. L'entretien peut prendre un peu plus de temps s'il y a beaucoup de membres dans ce ménage. En outre, nous visiterons votre ménage deux fois en différentes saisons (une fois pendant la saison sèche et une fois vers la fin de la saison des pluies) pendant la période totale de l'étude qui est de deux ans environ pour recueillir des données comme décrit dans ce paragraphe du formulaire de consentement.

Participation

Vous, le décideur principal pour le comportement de santé quotidienne et l'utilisation des soins de santé dans ce ménage, ainsi que les membres de votre ménage, êtes libres de choisir d'accepter ou de refuser de participer à l'étude. Il n'y a aucun problème si vous ne voulez pas participer. Il n'y a non plus aucun problème si vous voulez arrêter votre participation à tout moment, et cela ne causera aucun tord à vous ou aux membres de votre ménage. Donc, la participation à cette étude est volontaire, et vous êtes libre de refuser de participer à l'étude ou vous pouvez retirer votre consentement à tout moment sans donner de raisons et ceci n'entraînera aucune pénalité.

Risques et avantages

La participation à cette étude n'entraine pas de risque direct. Poser des questions au sujet de votre ménage peut mettre mal à l'aise vous ou les membres de votre ménage. Après avoir terminé les questions, l'équipe de recherche pourra fournir des informations sur les soins de santé communautaires pour les participants qui expriment de la détresse. Vous pouvez refuser de répondre à toute question à tout moment. Vous pouvez faire une pause ou arrêter de participer à cette étude à tout moment. Il n'y a pas d'avantages directs à être dans cette étude. Au cours des visites de votre ménage, notre équipe de recherche peut identifier les membres de votre ménage qui devraient chercher des soins de santé pour les maladies causant de la fièvre et autres. Par conséquent, elle peut aider à fournir de l'information sur l'offre de soins établie dans votre communauté. Les avantages indirects sont que toutes les informations collectées contribueront à fournir des données exactes sur les problèmes de santé, y compris le statu socio-économique, le comportement d'hygiène et d'assainissement et le statut vaccinal des enfants dans votre communauté. Cela aidera à donner une meilleure compréhension des facteurs influençant la transmission de la maladie et l'infection, et à estimer le fardeau des maladies courantes ainsi qu'à trouver des moyens de prévenir et de traiter les personnes dans votre communauté.

Coûts et Compensations

Il n'a pas de coûts pour les participants de cette étude. Ni vous ni aucune personne de votre ménage ne sera indemnisée d'avoir participé.

Confidentialité

Tous les fichiers de l'étude et de toutes les informations qui identifient vous et les membres de votre ménage seront gardées confidentielles. Tous les questionnaires sur papier seront gardés en toute sécurité et les fichiers informatisés seront protégés par un mot de passe; tous les fichiers de données (sur papier/informatisés) seront stockées pour un minimum de cinq ans; ces fichiers seront accessibles seulement à quelques personnes autorisées de l'étude. Ni votre nom ni aucun identifiant ne sera utilisé dans les publications ou les rapports de

For peer review only - http://bmjopen.bmj.com/site/about/guidelines.xhtml

cette étude. Les informations que nous collectons à votre sujet et au sujet des membres de votre ménage seront partagées uniquement avec le personnel de l'étude autorisé et des représentants du comité d'éthique.

Personnes de contact pour des questions
Si vous ou des membres de votre ménage avez des questions, s'il vous plaît, demandez maintenant à l'équipe de recherche ou à tout moment plus tard en contactant [ajouter le nom de l'investigateur principal et/ou du co-investigateur, leurs affiliations respectives et les numéros de téléphone]. Si vous avez des questions sur vos droits ou ceux des membres du ménage en tant que participant à l'étude, vous pouvez contacter [ajouter le nom et les coordonnées du comité d'éthique local].

Déclaration de consentement
Ce formulaire de consentement pour participer à l'évaluation des soins de santé et d'autres facteurs liés à la population à [ajouter le site et pays] a été bien lu par moi/pour moi. L'objectif, les procédures, les risques et les avantages ont m'ont été expliqués en détails. J'ai été autorisé à poser des questions à tout moment, et mes questions ont été répondues à ma satisfaction par l'équipe de recherche. On m'a dit la personne à contacter en cas de questions ou si je veux discuter de problèmes ou préoccupations. On m'a dit que je recevrai une copie signée et datée de ce formulaire de consentement. Je suis rassuré que toutes les informations obtenues par suite de cette étude seront gardées confidentielles et utilisées uniquement aux fins de cette étude, et seulement par les institutions participantes.

Moi, le répondant, consens, par la présente, volontairement à participer à cette étude. Je suivrai les instructions de l'équipe de recherche et donnerai mon entière collaboration. Je comprends que j'ai le droit de me retirer de l'étude à tout moment.

RÉPONDANT

Nom : ____

(prénom + deuxième prénom (si disponible) + nom de famille)

Signature : ____

(Représentation manuscrite du nom/ marque que la personne écrit sur un document comme une preuve d'identité)

Date ______/______/______________ (YY/MM/AAAA)

Empreinte du pouce

Si le répondant ne peut pas signer/est analphabète, mais accepte de participer, enregistre l'empreinte du pouce dans la boîte ci-dessus, et demande à un témoin adulte, alphabétisé et indépendant de l'étude (par exemple membre du même ménage ou un voisin), de signer ci-dessous :

TÉMOIN

Nom : ____

(prénom + deuxième prénom (si disponible) + nom de famille)

Signature : ____

(Représentation manuscrite du nom/ marque que la personne écrit sur un document comme une preuve d'identité)

Date ______/______/______________ (YY/MM/AAAA)

Moi, l'enquêteur, j'ai lu/expliqué l'étude au répondant nommé ci-dessus (témoin si le répondant est analphabète) dans une langue que le répondant comprend bien. Je suis certain que le répondant a compris l'information et accepte qu'on lui pose des questions.

INTERVIEWER

Nom : ____

(prénom + deuxième prénom (si disponible) + nom de famille)

Signature : ____

(Représentation manuscrite du nom/ marque que la personne écrit sur un document comme une preuve d'identité)

Date ______/______/______________ (YY/MM/AAAA)

For peer review only - http://bmjopen.bmj.com/site/about/guidelines.xhtml

Site ___ ___ ___ Sous-zone ___ ___ ___ Numéro d'habitation ___ ___ ___ ___ Identifiant de l'enquêteur ___ __

FORMULAIRE 3: INFORMATIONS GÉNÉRALES SUR LE MÉNAGE

Instructions pour l'enquêteur

- Compléter ce formulaire uniquement si la personne interrogée a consenti à participer (voir « Formulaire 2 — FORMULAIRE DE CONSENTEMENT ») ; la personne interrogée ainsi que définie pour cette enquête est un adulte (selon l'âge légal de majorité du pays), un membre du ménage et un décideur en ce qui concerne les soins de santé quotidiens et l'utilisation de soins de santé pour le ménage entier et ses membre ; il ne doit pas être, par exemple, un voisin ou un visiteur.
- Recueillir les données de tous les membres du ménage que tous les membres du ménage soient présents ou non au moment de l'entretien ; ne recueillir et n'inscrire les données que des personnes présentes au moment de l'entretien fausserait les données.
- Un ménage est défini/les membres d'un ménage sont définis comme une personne ou un groupe de personnes avec ou sans lien de parenté qui vivent ensemble dans la même unité d'habitation, qui reconnaissent un adulte de sexe masculin ou féminin comme chef de ménage, qui partagent les mêmes installations domestiques, qui sont considérés comme constituant une unité, et qui subviennent à leurs besoins en termes de nourriture et des autres éléments essentiels pour vivre.
- « Site » et « sous-zone » doivent être un numéro à trois chiffres, « numéro d'habitation » un numéro à quatre chiffres, et « identifiant de l'enquêteur » un numéro à deux chiffres (utiliser les listes préparées individuellement pour chaque site pour obtenir les codes) ; saisir « date » au format JJ/MM/AAAA et « heure » au format HH:MM et entourer MATIN ou APRÈS-MIDI. «Étiquette de l'étude» doit être une combinaison d'un code «site» à trois chiffres, d'un code «sous-zone» à trois chiffres et d'un code «numéro d'habitation» à quatre chiffres; le « identifiant de l'enquêteur » doit être un numéro à trois chiffres.

FORMULAIRE 3: INFORMATIONS GÉNÉRALES SUR LE MÉNAGE
PARTIE A – DONNÉES DÉMOGRAPHIQUES/ INFORMATIONS GÉNÉRALES SUR LE MÉNAGE

EMPLACEMENT DU DOMICILE

1 Numéros de site, de sous-zone et d'habitation

Instructions : Site (=site d'étude) et sous-zone (=plus petite unité du site d'étude) doivent être tous les deux des numéros à trois chiffres, et numéro d'habitation doit être un code à quatre chiffres ; utiliser les listes préparées individuellement pour obtenir les codes respectifs.

Site: ____ ____ ____ Sous-zone: ____ ____ ____ Numéro d'habitation: ____ ____ ____ ____

2 Coordonnées GPS

Instructions : Prendre les coordonnées GPS approximativement au centre de la maison. Inscrire l'altitude par un nombre à quatre chiffres (unité : mètre), et la latitude/longitude par un numéro à huit chiffres (unités : degrés, minutes décimales).

O 1=Ménage d'origine O 2=Ménage de remplacement (côte droit/gauche du ménage d'origine)

Altitude: ___ ___ ___ ___

Latitude: ___ ___°___ ___.___ ___ ___' Longitude: ___ ___°___ ___.___ ___ ___'

PERSONNE INTERROGÉE

3 Inscrire le nom complet de la personne interrogée.

Instructions : Inscrire le prénom, le deuxième nom (le cas échéant) et le nom de famille de la personne interrogée.

____

4 Inscrire l'âge de la personne interrogée.

Instruction : Utiliser un numéro à trois chiffres (unité: année). _____ _____ _____

5 Inscrire le sexe de la personne interrogée.

O 1=Masculin O 2=Féminin

6 Quel est le lien de la personne interrogée avec le ménage ?

O 1=Chef de ménage
O 2=Fils/fille du chef de ménage
O 7=Conjoint du chef de ménage
O 8=Conjoint du fils/fille du chef de ménage

For peer review only - http://bmjopen.bmj.com/site/about/guidelines.xhtml

- O 3=Frère/sœur du chef de ménage
- O 4=Mère/père du chef de ménage
- O 5=Petite-fille/petit-fils du chef de ménage
- O 6=Autre, préciser __________
- O 9=Conjoint du frère/sœur du chef de ménage
- O 10=Belle-mère/beau-père du chef de ménage
- O 11=Nièce/neveu du chef de ménage
- O 98=Pas de réponse

MEMBRES DU MÉNAGE

7 Combien de membres du ménage vivent dans ce domicile au total (au moment de la visite) **?**

Instruction : Utiliser un numéro à deux chiffres.

Nombre total de membres du ménage : ____ ____

8 Inscrire l'âge et le sexe de chaque membre du ménage.

Instructions : Inscrire les initiales, l'âge et le sexe de la personne interrogée en première position/ligne du tableau. Les initiales, l'âge et le sexe de tous les autres membres du ménage doivent être inscrits ensuite. Les initiales d'un membre du ménage consistent en les trois premières lettres du prénom et les trois première lettre du nom de famille (un éventuel deuxième nom ne fait pas partie des initiales). Inscrire l'âge de chaque membre du ménage avec un numéro à trois chiffres (unité : années) ; si l'âge d'un des membres du ménage ≤ 12 mois, inscrire 1 an comme âge. Merci de compléter un formulaire supplémentaire si le ménage a plus de vingt membres.

Membre du ménage Identifiant	Initiales [prénom/nom de famille]	Âge [années]	Sexe
1/Personne interrogée	____ ____ ____/____ ____ ____	____ ____ ____	O1=Masculin O2=Féminin
2	____ ____ ____/____ ____ ____	____ ____ ____	O1=Masculin O2=Féminin
3	____ ____ ____/____ ____ ____	____ ____ ____	O1=Masculin O2=Féminin
4	____ ____ ____/____ ____ ____	____ ____ ____	O1=Masculin O2=Féminin
5	____ ____ ____/____ ____ ____	____ ____ ____	O1=Masculin O2=Féminin
6	____ ____ ____/____ ____ ____	____ ____ ____	O1=Masculin O2=Féminin
7	____ ____ ____/____ ____ ____	____ ____ ____	O1=Masculin O2=Féminin
8	____ ____ ____/____ ____ ____	____ ____ ____	O1=Masculin O2=Féminin
9	____ ____ ____/____ ____ ____	____ ____ ____	O1=Masculin O2=Féminin
10	____ ____ ____/____ ____ ____	____ ____ ____	O1=Masculin O2=Féminin
11	____ ____ ____/____ ____ ____	____ ____ ____	O1=Masculin O2=Féminin
12	____ ____ ____/____ ____ ____	____ ____ ____	O1=Masculin O2=Féminin
13	____ ____ ____/____ ____ ____	____ ____ ____	O1=Masculin O2=Féminin
14	____ ____ ____/____ ____ ____	____ ____ ____	O1=Masculin O2=Féminin
15	____ ____ ____/____ ____ ____	____ ____ ____	O1=Masculin O2=Féminin
16	____ ____ ____/____ ____ ____	____ ____ ____	O1=Masculin O2=Féminin
17	____ ____ ____/____ ____ ____	____ ____ ____	O1=Masculin O2=Féminin
18	____ ____ ____/____ ____ ____	____ ____ ____	O1=Masculin O2=Féminin
19	____ ____ ____/____ ____ ____	____ ____ ____	O1=Masculin O2=Féminin
20	____ ____ ____/____ ____ ____	____ ____ ____	O1=Masculin O2=Féminin

For peer review only - http://bmjopen.bmj.com/site/about/guidelines.xhtml

FORMULAIRE 3: INFORMATIONS GÉNÉRALES SUR LE MÉNAGE
PARTIE B – DONNÉÉS SOCIO-ECONOMIQUES

ÉDUCATION

1 Le chef de ménage sait-il lire en [inscrire la langue officielle du site d'étude/pays] **?**
O 1=Oui O 2=Non O 99=Ne sait pas O 98=Pas de réponse

2 Le chef de ménage sait-il écrire en [inscrire la langue officielle du site d'étude/pays] **?**
O 1=Oui O 2=Non O 99=Ne sait pas O 98=Pas de réponse

3 Quel est le niveau d'éducation scolaire le plus élevé atteint par le chef de ménage ?
O 1=École primaire O 4=Plus élevé que le lycée O 99=Ne sait pas
O 2=Collège O 5= PAS D'ÉDUCATION O 98=Pas de réponse
O 3=Lycée O 6=Autre, préciser ___________

PROFESSION/RESSOURCES FINANCIÈRES

4 Quelle est la profession principale du chef de ménage ?
Instructions : Inscrire le statut du jour de visite uniquement.

O 1=Groupe 1 : Cadres
(directeurs généraux, hauts fonctionnaires, législateurs ; cadres administratifs et commerciaux ; responsables de production et de services spécialisés ; responsables dans l'hôtellerie, la vente et d'autres services)

O 2=Groupe 2 : Professionnels
(scientifiques ou ingénieurs ; professionnels de santé ; professionnels de l'enseignement ; professionnels de l'administration et des affaires ; professionnels de l'information et des technologies de communication ; professionnels du droit, du social ou de la culture)

O 3=Groupe 3 : Techniciens et professions intermédiaires
(Professions intermédiaires des sciences et techniques ; professions intermédiaires de la santé ; professions intermédiaires de l'administration et des affaires ; professions intermédiaires du droit, du social, de la culture et assimilés ; techniciens de l'information et des communications)

O 4=Groupe 4 : Employés de bureau
(secrétaires et dactylographes ; employé de service clientèle ; employé de conservation des données et de la documentation ; autres employés de bureau)

O 5=Groupe 5 : Travailleurs des services et de la vente
(travailleurs du service aux personnes ; travailleurs de la vente ; travailleurs des soins aux personnes ; travailleurs des services de protection)

O 6=Groupe 6 : Travailleurs qualifiés de l'agriculture, la sylviculture et la pêche
(travailleurs qualifiés de l'agriculture orientés marché ; éleveurs d'animaux; travailleurs qualifiés de la sylviculture, la pêche et la chasse orientés marché ; agriculteurs, pêcheurs, chasseurs et cueilleurs de subsistance)

O 7=Groupe 7 : Travailleurs manuels et assimilés
(travailleurs de la construction et assimilés (à l'exception des électriciens) ; travailleurs de la métallurgie, sur machines et assimilés ; artisans et travailleurs de l'imprimerie ; travailleurs des métiers de l'électrique et de l'électronique ; travailleurs des métiers de la transformation des aliments, du bois, des vêtements et assimilés)

O 8=Groupe 8 : Opérateurs de machines et d'installations, et monteurs
(Opérateurs de machines et d'installations fixes ; monteurs ; conducteurs opérateurs d'installations mobiles)

O 9=Groupe 9 : Emplois non qualifiés
(agents d'entretien et assistants ; ouvriers de l'agricultures, la sylviculture et la pêche ; travailleurs des mines, de la construction, de l'industrie et des transports ; assistants à la préparation des aliments ; vendeurs et services de rue et assimilés ; travailleurs de la collecte des ordures et autres travailleurs non qualifiés)

O 10=Groupe 0 : Professions des forces armées
(officiers des forces armées ; sous-officiers des forces armées ; professions des forces armées, autres grades)

O 11=Autre, préciser __________
O 12=PAS DE PROFESSION O 99=Ne sait pas O 98=Pas de réponse

For peer review only - http://bmjopen.bmj.com/site/about/guidelines.xhtml

5 Quel est le montant moyen des revenus mensuels du chef de ménage en lien avec à la profession indiquée en question 4 ainsi que celui des autres membre du ménage ?

Instruction: Tick one answer per household head/enter one code per household member if applicable/if income available.

Chef de ménage	Membre 1	Membre 2	Membre 3
Code ____________	Code ____________	Code ____________	Code ____________
[1] <100USD<br>[2] ≥100 à <150USD	[3] ≥150 à <200USD<br>[4] ≥200 à <250USD	[5] ≥250USD<br>[6] PAS DE REVENU (chef de ménage; voir Q4)	[99] Ne sait pas<br>[98] Pas de réponse

6 Quels sont les autres sources ou les sources supplémentaires de revenus mensuels du ménage et quel en est le montant ?

Instruction : Cocher plusieurs cases si nécessaire ; il peut y avoir plus d'une réponse.

Autres sources/Sources supplémentaires	Revenus financiers mensuels globaux
O 1=Aides sociales<br>O 2=Assurances (publiques ou privées)<br>O 3=Prêt (d'amis, de membres de la famille, d'une banque)<br>O 4=Vente (bétail, récoltes, meubles, autres biens)<br>O 5=Économies personnelles<br>O 6=Autre, préciser ________________	O 1=<100 USD<br>O 2=≥100 à <150 USD<br>O 3=≥150 à <200 USD<br>O 4=≥200 à <250 USD<br>O 5=≥250 USD
O 7=PAS D'AUTRES SOURCES/DE SOURCES SUPPLÉMENTAIRES<br>O 99=Ne sait pas<br>O 98=Pas de réponse	

For peer review only

LOGEMENT/HABITATION

7 Les membres du ménage sont-ils propriétaires de l'habitation ?

O 1=Oui O 3=Non, logement fourni (par le gouvernement, un employeur) O 99=Ne sait pas

O 2=Non, locataires O 4=Non, autres, préciser ________ O 98=Pas de réponse

8 Combien de pièces ou structures individuelles de l'habitation sont utilisées pour dormir, vivre, faire la cuisine ou se laver ?

Instruction : N'indiquer qu'un code par catégorie.

Chambre	Salon	Cuisine	Salle de bain
Code ____________	Code ____________	Code ____________	Code ____________
[1] 1-2<br>[2] 3-4	[2] >4<br>[2] Aucune	[99] Ne sait pas<br>[98] Pas de réponse	

9 En quel matériau sont construits le sol, les murs et le toit des pièces/des structures individuelles de l'habitation ?

Instruction : Indiquer tous les codes par catégorie ; plus d'une réponse par catégorie est possible.

Sol	Murs	Toit
Code ____________	Code ____________	Code ____________
[1] Brique [3] Tuiles<br>[2] Ciment/Béton [4] Terre	[5] Bois<br>[6] Paille/feuilles	[7] Métal/Étain [99] Ne sait pas<br>[8] Autre, préciser_________ [98] Pas de réponse

10 Les membres de ce ménage ont-ils accès à/utilisent-ils l'électricité dans cette habitation ?

O 1=Oui O 2=Non O 99=Ne sait pas O 98=Pas de réponse

11 À quelle source de lumière principale les membres de ce ménage ont-ils accès/Quelle source de lumière principale utilisent-ils ?

O 1=Lumière/ampoule(s) électrique(s) O 4=Lampe(s) à piles O 7= Autre, préciser_____

O 2=Lumière du soleil/Lampe(s) solaire(s) O 5=Bougie(s) O 99=Ne sait pas

O 3=Lampe(s) à pétrole/à huile O 6=PAS DE SOURCE DE LUMIÈRE O 98=Pas de réponse

For peer review only - http://bmjopen.bmj.com/site/about/guidelines.xhtml

BIENS DU MÉNAGE

12 Parmi les articles suivants, lesquels les membres du ménage possèdent-ils ?

Instructions : Cocher plusieurs cases si nécessaire ; il peut y avoir plus d'une réponse ; indiquer la quantité d'articles fonctionnels si nécessaire

O 1=Radio	Quantité ____	O Rien	O 11=Lecteur cassettes/CD	Quantité ____	O Rien
O 2=Télévision	Quantité ____	O Rien	O 12=Ordinateur de bureau/portable/tablette	Quantité ____	O Rien
O 3=Connexion internet	Quantité ____	O Rien	O 13=Téléphone portable	Quantité ____	O Rien
O 4=Voiture	Quantité ____	O Rien	O 14=Camion/bus	Quantité ____	O Rien
O 5=Moto	Quantité ____	O Rien	O 15=Vélo	Quantité ____	O Rien
O 6=Char à bœufs/ânes	Quantité ____	O Rien	O 16=Lit	Quantité ____	O Rien
O 7=Matelas	Quantité ____	O Rien	O 17=Moustiquaire	Quantité ____	O Rien
O 8=Table	Quantité ____	O Rien	O 18=Chaise	Quantité ____	O Rien
O 9=Réfrigérateur (4°C)	Quantité ____	O Rien	O 19=Congélateur (-20°C)	Quantité ____	O Rien
O 10=Ventilateur	Quantité ____	O Rien	O 20=Machine à coudre	Quantité ____	O Rien

For peer review only

FORMULAIRE 3: INFORMATIONS GÉNÉRALES SUR LE MÉNAGE
Partie C – Hygiène et installations sanitaires

TOILETTES

1 À quel type de toilettes les membres du ménage ont-ils habituellement accès ?

Instruction : À l'intérieur signifie à l'intérieur d'une pièce/d'une habitation individuelle/d'un bâtiment ; à l'extérieur signifie à l'extérieur d'une pièce/d'une habitation individuelle/d'un bâtiment.

- O 1=Toilettes avec chasse d'eau/Cabinets (à l'intérieur)
- O 2=Toilettes avec chasse d'eau/Cabinets (à l'extérieur)
- O 3=Latrines sans chasse/latrines à fosse (à l'intérieur)
- O 4=Latrines sans chasse/latrines à fosse (à l'extérieur)
- O 5=Latrines à fosse améliorées par une ventilation (KVIP, à l'intérieur)
- O 6=Latrines à fosse améliorées par une ventilation (KVIP, à l'extérieur)
- O 7=Toilettes à compostage (à l'intérieur)
- O 8=Toilettes à compostage (à l'extérieur)
- O 9=PAS de toilettes, utilisent un seau
- O 10=PAS de toilettes, creusent un trou
- O 11=PAS de toilettes, défécation libre/en plein air
- O 12=Autre, préciser __________
- O 99=Ne sait pas
- O 98=Pas de réponse

2 Les membres du ménage utilisent-ils habituellement les toilettes indiquées à la question 1 ?

- O 1=Non, les membres ne peuvent utiliser les toilettes de façon autonome (bébés, enfants en bas-âge)
- O 2=Non, les membres ne peuvent utiliser les toilettes de façon autonome (handicapés, malades chroniques/longue durée)
- O 3=Non, les membres ne peuvent utiliser les toilettes de façon autonome (personnes âgées)
- O 4=Non, accès limité (publiques, partagées avec d'autres ménages/communautés)
- O 5=Non, accès limité (publiques, partagées avec d'autres ménages/communautés)
- O 6= Non, mauvais état (cassé, pas propre)
- O 7=OUI
- O 8=Autre, préciser _________________
- O 99=Ne sait pas
- O 98=Pas de réponse

3 Où sont situées les toilettes indiquées à la question 1 ? Par combien de personnes sont-elles habituellement partagées ?

- O 1=Sur la propriété du ménage (privées, non partagées)
- O 2=Sur la propriété d'un ménage voisin (privées, partagées)
  - *Partagées par* O 1=<10 personnes O 2=11-24 personnes O 3=25-49 personnes O 4=>50 personnes O 99=Ne sait pas O 98=Pas de réponse
- O 3=Dans la communauté (publiques, partagées)
  - *Partagées par* O 1=<10 personnes O 2=11-24 personnes O 3=25-49 personnes O 4=>50 personnes O 99=Ne sait pas O 98=Pas de réponse
- O 4=Autre, préciser__________ O 99=Ne sait pas O 98=Pas de réponse

4 Comment les membres du ménage qui ont accès/n'ont pas accès aux toilettes se lavent-ils habituellement les mains tout de suite avant et après avoir uriné/déféqué ?

Instruction : Indiquer un code par catégorie uniquement.

Lavent les mains avant d'avoir uriné/déféqué	Lavent les mains après avoir uriné/déféqué
Code _____________	Code _____________
[1] Lavent les mains avec du savon et de l'eau<br>[2] Lavent les mains avec de l'eau uniquement<br>[3] Frottent les mains avec des feuilles/de la paille/de l'herbe<br>[4] Frottent les mains avec du sable<br>[5] Frottent les mains avec un chiffon/du tissu	[6] Ne lavent pas, pas besoin/pas sale<br>[7] Ne lavent pas, rien pour laver les mains<br>[8] Autre, préciser __________<br>[99] Ne sait pas<br>[98] Pas de réponse

CUISINE/MANIPULATION DES ALIMENTS

5 Les membres de ce ménage ont-ils une cuisine pour faire cuire/préparer la nourriture/les repas ? Où est-elle située ?

- O 1=Oui, cuisine intérieure/dans une pièce/structure individuelle d'habitation ; sur la propriété du ménage (privée, non partagée)
- O 2=Oui, cuisine extérieure/hors d'une pièce/structure individuelle d'habitation/ en plein air ; sur la propriété du ménage (privée, non partagée)

For peer review only - http://bmjopen.bmj.com/site/about/guidelines.xhtml

O 3=Oui, cuisine intérieure/dans une pièce/structure individuelle d'habitation ; sur la propriété d'un ménage voisin (privée, partagée)
O 4=Oui, cuisine extérieure/hors d'une pièce/structure individuelle d'habitation/ en plein air ; sur la propriété d'un ménage voisin (privée, partagée)
O 5=PAS DE CUISINE
O 6=Autre, préciser __________ O 99=Ne sait pas O 98=Pas de réponse

6 Quelle source d'énergie les membres de ce ménage utilisent-ils habituellement pour cuisiner/préparer la nourriture/les repas ?

O 1=Électricité O 3=Charbon de bois O 5=Excréments d'animaux O 99=Ne sait pas
O 2=Gaz/pétrole O 4=Bois/paille/feuilles O 6=Autre, préciser __________ O 98=Pas de réponse

7 Quelle moyen de cuisson les membres de ce ménage utilisent-ils habituellement pour cuisiner/préparer la nourriture/les repas ?

O 1=Foyer ouvert O 3=Fourneau intérieur avec cheminée O 99=Ne sait pas
O 2=Fourneau extérieur O 4=Autre, préciser __________ O 98=Pas de réponse

8 Où les membres de ce ménage conservent-ils habituellement les produits alimentaires frais/crus (comme les légumes, la salade, les fruits, les produits laitiers, les œufs, la viande et le poisson) et les restes/les aliments/repas préparés ?

Instruction : Indiquer un code par catégorie uniquement. À l'intérieur signifie à l'intérieur d'une pièce/d'une habitation individuelle/d'un bâtiment ; à l'extérieur signifie à l'extérieur d'une pièce/d'une habitation individuelle/d'un bâtiment.

Des produits alimentaires frais/crus (légumes, salade, fruits, produits laitiers, œufs, viande et poisson)	Des restes/aliments/repas préparés
Code _____________	Code _____________

[1] À température ambiante (à l'intérieur, sur la propriété du ménage, privé)
[2] À température ambiante (à l'extérieur, sur la propriété du ménage, privé)
[3] À température ambiante (à l'intérieur, sur la propriété d'un ménage voisin, privé, partagé)
[4] À température ambiante (à l'extérieur, sur la propriété d'un ménage voisin, privé, partagé)
[5] Réfrigérateur (sur la propriété du ménage, privé)
[6] Réfrigérateur (sur la propriété d'un ménage voisin, privé, partagé)
[7] Congélateur (sur la propriété du ménage, privé)
[8] Congélateur (sur la propriété d'un ménage voisin, privé, partagé)
[9] Autre, préciser _______________ [10] PAS DE CONSERVATION [99] Ne sait pas [98] Pas de réponse

9 Si la réponse à la question 8 au sujet des produits alimentaires frais/crus et/ou des restes/aliments/repas préparés est «température ambiante», combien de temps les membres de ce ménage les conservent-ils habituellement?

Instruction: Indiquer un code par catégorie uniquement.

Des produits alimentaires frais/crus (légumes, salade, fruits, produits laitiers, œufs, viande et poisson)	Des restes/aliments/repas préparés
Code _____________	Code _____________

[1] <1 jour [3] 3-4 jours [5] Pas applicable [99] Ne sait pas
[2] 1-2 jours [4] >4 jours [98] Pas de réponse

10 Les membres de ce ménage couvrent-ils habituellement les restes/aliments/repas préparés? Réchauffent-ils/recuisent-ils les restes/aliments/repas préparés avant de les consommer?

Instruction : Indiquer un code par catégorie uniquement.

Couvrent les restes/les aliments/repas préparés	Réchauffent/Recuisent les restes/les aliments/repas préparés
Code _____________	Code _____________

[1] Oui [2] Non [99] Ne sait pas [98] Pas de réponse

For peer review only - http://bmjopen.bmj.com/site/about/guidelines.xhtml

11 Quel membre(s) de ce ménage cuisine(s)/prépare(s) la nourriture/les repas habituellement pour les autres membres de ce ménage ?

Instructions : Se référer à la question 9 du « Formulaire 3 Partie A – Données démographiques/Informations générales sur le ménage » et indiquer les initiales/ Identifiant (s) du membre.

O Membre du ménage, indiquer les initiales/identificateur(s) __________

O 99=Ne sait pas O 98=Pas de réponse

12 Comment le(s) membre(s) de la famille qui cuisine(nt)/prépare(nt) la nourriture/les repas habituellement pour les autres membres de ce ménage comme indiqué à la question 11 se prépare(nt)-t-il(s)/elle(s) habituellement avant et après la préparation des repas?

Instruction: Indiquer un code par catégorie uniquement.

Avant la préparation des repas	Après la préparation des repas
Code _____________	Code _____________
[1] Lavent les mains avec du savon et de l'eau<br>[2] Lavent les mains avec de l'eau uniquement<br>[3] Frottent les mains avec des feuilles/de la paille/de l'herbe<br>[4] Frottent les mains avec du sable<br>[5] Frottent les mains avec un chiffon/du tissu	[6] Ne lavent pas, pas besoin/pas sale<br>[7] Ne lavent pas, rien pour laver les mains<br>[8] Autre, préciser __________<br>[99] Ne sait pas<br>[98] Pas de réponse

13 Comment les membres de ce ménage se préparent-ils habituellement avant et après avoir mangé de la nourriture/des repas, y compris le membre du ménage qui cuisine/prépare la nourriture/les repas habituellement comme indiqué à la question 11?

Instruction : Indiquer un code par catégorie uniquement.

Avant la préparation des repas	Après la préparation des repas
Code _____________	Code _____________
[1] Lavent les mains avec du savon et de l'eau<br>[2] Lavent les mains avec de l'eau uniquement<br>[3] Frottent les mains avec des feuilles/de la paille/de l'herbe<br>[4] Frottent les mains avec du sable<br>[5] Frottent les mains avec un chiffon/du tissu	[6] Ne lavent pas, pas besoin/pas sale<br>[7] Ne lavent pas, rien pour laver les mains<br>[8] Autre, préciser __________<br>[99] Ne sait pas<br>[98] Pas de réponse

14 Comment les membres de ce ménage mangent-ils habituellement leur nourriture/leurs repas, y compris le membre du ménage qui cuisine/prépare la nourriture/les repas habituellement comme indiqué à la question 11?

O 1=Avec des couverts O 3=Autre, préciser __________ O 99=Ne sait pas

O 2=Avec les doints O 98=Pas de réponse

15 Les membres de ce ménage mangent-ils habituellement leur nourriture/leurs repas dans le même récipient, y compris le membre du ménage qui cuisine/prépare la nourriture/les repas habituellement comme indiqué à la question 11?

O 1=Oui O 2=Non O 99=Ne sait pas O 98=Pas de réponse

16 Où les membres de ce ménage obtiennent-ils habituellement leurs produits alimentaires frais/crus principaux comme les légumes, les fruits, les grains/céréales, les produits laitiers, les œufs, la viande et le poisson?

Instruction : Indiquer un code par catégorie uniquement.

Légumes, fruits, grains/céréales	Produits laitiers	Œufs, viande, poisson
Code _____________	Code _____________	Code _____________

[1] Agriculture vivrière (cour/arrière-cour)<br>[2] Supermarché/Épicerie<br>[3] Amis/famille<br>[4] Marché ouvert<br>[5] Vendeur de rue	[6] Agriculture vivrière (terre agricole)<br>[7] Boucher<br>[8] Autre, préciser ______<br>[99] Ne sait pas<br>[98] Pas de réponse

For peer review only - http://bmjopen.bmj.com/site/about/guidelines.xhtml

17 Les membres de ce ménage consomment/mangent-ils des produits alimentaires frais/crus (comme les légumes, la salade, les fruits, les produits laitiers, la glace, les œufs, la viande et le poisson) ? Si oui, lesquels?

Instruction : Cocher plusieurs cases si nécessaire ; plus d'une réponse est possible.

O 1=Légumes/salade (cultivés sur arbuste)
O 2=Légumes/salade (récolte sauvage sur arbuste)
O 3=Fruits (cultivés sur arbuste/arbre)
O 4=Fruits (récolte sauvage sur arbuste/arbre)
O 5=Produits laitiers (lait, fromage, yaourt d'animaux domestiques produisant du lait), préciser ____________
O 6=Œufs (de volailles domestiques), préciser _________
O 7=Sang animal (d'animaux domestiques), domestiques), préciser _________
O 8=Bœuf/Zébu/Buffle (domestiqué), préciser _________
O 9=Porc (domestiqué)
O 10=Mouton (domestiqué)
O 11=Viande d'animaux non-domestiqués (antilope, sanglier, volaille, rongeurs, poisson), préciser ________
O 12=PAS DE CONSOMMATION de produits frais/crus
O 13=Légumes/salade (cultivés au sol)
O 14=Légumes/salade (récolte sauvage sur le sol)
O 15=Fruits (cultivés au sol)
O 16=Fruits (récolte sauvage sur le sol)
O 17=Produits laitiers (lait, fromage, yaourt d'animaux non-domestiques produisant du lait), préciser ____________
O 18= Œufs (de volailles non-domestiques), préciser _________
O 19= Sang animal (d'animaux non- préciser _________
O 20=Volaille (domestiquée; poulet, canard, pigeon, oie, dinde), préciser _________
O 21=Chèvre (domestiquée)
O 22= Rongeurs (domestiqués), préciser ______
O 23=Poisson/fruits de mer, préciser ______
O 24=Glace
O 25=Autre, préciser _________
O 99=Ne sait pas O 98=Pas de réponse

18 Les membres de ce ménage consomment-ils habituellement des repas/produits alimentaires/boissons également en dehors de l'habitation ? Si oui, quel(s) membre(s) de ce ménage et où ?

Instructions : Se référer à la question 9 du « Formulaire 3 Partie A – Données démographiques/Informations générales sur le ménage » et indiquer les initiales du/des membre(s). Indiquer tous les codes nécessaires pour les membres du ménage respectifs.

Membre 1 Initiales/Identifiant : ___	Membre 2 Initiales/Identifiant : ___	Membre 3 Initiales/Identifiant : ___	Membre 4 Initiales/ Identifiant : ___
Code ____________	Code ____________	Code ____________	Code ____________
[1] Restaurant<br>[2] Chaîne de restauration rapide	[3] Cafétéria<br>[4] Vendeur de rue	[5] Autre, préciser ____<br>[6] Pas applicable	[99] Ne sait pas<br>[98] Pas de réponse

19 Quels/Quelles repas/produits alimentaires/boissons le/les membre(s) de ce ménage listé à la question 18 consomment-ils habituellement en dehors de l'habitation ?

Instructions : Se référer à la question 9 du « Formulaire 3 Partie A – Données démographiques/Informations générales sur le ménage » et indiquer/identifiant les initiales du/des membre(s). Indiquer jusqu'à 5 repas/produits alimentaires/boissons par membre du ménage. Vous pouvez vous référer aux produits alimentaires listés à la question 17. Passer cette question si aucun membre du ménage ne consomme les repas/les produits alimentaires/les boissons en dehord de la maison comme indiqué dans Q18.

Membre 1 Initiales/Identifiant : ____	Membre 2 Initiales/Identifiant : ____	Membre 3 Initiales/Identifiant : ____	Membre 4 Initiales/ Identifiant : ___
1: ______________<br>2: ______________<br>3: ______________<br>4: ______________<br>5: ______________	1: ______________<br>2: ______________<br>3: ______________<br>4: ______________<br>5: ______________	1: ______________<br>2: ______________<br>3: ______________<br>4: ______________<br>5: ______________	1: ______________<br>2: ______________<br>3: ______________<br>4: ______________<br>5: ______________

EAU

20 Quelle est la source d'approvisionnement en eau principale des membres de ce ménage pour la boisson, la cuisine/la préparation des aliments/repas, le bain et le ménage (de

For peer review only - http://bmjopen.bmj.com/site/about/guidelines.xhtml

l'habitation/de la/les structure(s) individuelle(s) d'habitation(s), les vêtements/les outils de cuisine) ?
Instruction : Indiquer un code par catégorie uniquement.

Boisson	Cuisine	Bain	Nettoyage
Code ____________	Code ____________	Code ____________	Code ____________

[1] Eau du robinet (à l'intérieur de l'habitation ; privée)
[2] Eau du robinet (à l'extérieur de l'habitation : cour/arrière-cour ; privée)
[3] Eau du robinet (à l'extérieur de l'habitation ; publique/partagée)
[4] Borne-fontaine/eau courante (à l'intérieur de l'habitation ; privée)
[5] Borne-fontaine/eau courante (à l'extérieur de l'habitation : cour/arrière-cour ; privée)
[6] Borne-fontaine/eau courante (à l'extérieur de l'habitation ; publique/partagée)
[7] Camion-citerne (public ; partagé)
[8] Eaux de surface (canal, canal d'irrigation)
[9] Eaux de surface (lac, étang, rivière, ruisseau)
[10] Eau de source
[11] Eau de pluie
[12] Puits/Puits de forage (en plein air/non couvert/non protégé)
[13] Puits/Puits de forage (couvert/protégé)
[14] Eau en bouteille
[15] Autre, préciser ________________ [99] Ne sait pas [98] Pas de réponse

21 Les membres de ce ménage traitent-ils habituellement l'eau pour la boisson, la cuisine/la préparation des aliments/des repas, le bain, et le nettoyage (de l'habitation/des/de la structure(s) individuelle(s) d'habitation(s), des vêtements/des outils de cuisine) avant la consommation ? Comment l'eau est-elle habituellement traitée ?
Instruction: Indiquer tous les codes nécessaires par catégorie ; plus d'une réponse par catégorie est possible.

Boisson	Cuisine	Bain	Nettoyage
Code ____________	Code ____________	Code ____________	Code ____________

[1] Non, pas du tout
[2] Non, seulement pour les malades du ménage
[3] Non, seulement selon la saison (saison sèche)
[4] Oui, par filtration (tissu, céramique, sable)
[5] Oui, par sédimentation
[6] Autre, préciser ______________
[7] Non, seulement pour les enfants du ménage
[8] Non, seulement selon la saison (saison humide)
[9] Oui, par ébullition
[10] Oui, par désinfection solaire
[11] Oui, par chloration
[99] Ne sait pas [98] Pas de réponse

22 Quel(s) membre(s) de ce ménage recueille(nt) habituellement l'eau potable comme indiqué à la question 20 pour les autres membres de ce ménage ?
Instructions : Se référer à la question 9 du « Formulaire 3 Partie A – Données démographiques/Informations générales sur le ménage » et indiquer/identifiant les initiales du membre.
○ Membre du ménage, indiquer les initiales/identifiant(s) _________
○ 99=Ne sait pas ○ 98=Pas de réponse

23 Comment les membres de ce ménage conservent-ils habituellement l'eau potable indiquée à la question 20?
○ 1=Réservoir d'eau (non couvert)
○ 2=Réservoir d'eau (couvert par chiffon/tissu, couvercle, feuilles)
○ 3=Récipient (non couvert)
○ 4=Récipient (couvert par chiffon/tissu, couvercle, feuilles)
○ 5=Pot en terre (non couvert)
○ 6=Pot en terre (couvert par chiffon/tissu, couvercle, feuilles)
○ 7=Seau (non couvert)
○ 10=Bol (couvert par chiffon/tissu, couvercle, feuilles)
○ 11=Pot en bois (non couvert)
○ 12= Pot en bois (couvert par chiffon/ tissu, couvercle, feuilles)
○ 13= Récipient en peau (chèvre, mouton, bovins, non couvert)
○ 14=Récipient en peau (chèvre, mouton, bovins ; couvert par un chiffon/du tissu, un couvercle, des feuilles
○ 15=NON CONSERVÉE
○ 16=Autre, préciser _________

For peer review only - http://bmjopen.bmj.com/site/about/guidelines.xhtml

O 8=Seau (couvert par chiffon/tissu, couvercle, feuilles) O 99=Ne sait pas
O 9=Bol (non couvert) O 98= Pas de réponse

24 Si les membres de ce ménage utilisent une source d'approvisionnement en eau potable partagée comme indiqué à la question 20, par combien de personnes cette eau potable est-elle partagée ?

O 1=<10 personnes O 3=25-49 personnes O 5=NON PARTAGÉE O 99=Ne sait pas
O 2=11-24 personnes O 4=>50 personnes O 98=Pas de réponse

TRAITEMENT DES DÉCHETS

25 Où les membres de ce ménage jettent-ils habituellement les eaux usées de, par exemple, la cuisine/la préparation des aliments/repas, le bain et/ou le ménage, et jettent-ils habituellement les déchets domestiques, les déchets humains/déjections ?

Instruction: Indiquer un code par catégorie uniquement.

Eaux usées de par exemple cuisine/préparation des aliments/ repas, bain et/ou ménage	Déchets domestiques	Déchets humains/déjections
Code _____________	Code _____________	Code _____________

[1] Sur le sol/surface libre proche de l'habitation (cour/arrière-cour, privé)
[2] Sur le sol/surface libre loin de l'habitation dans la communauté (partagé, public)
[3] Dans un trou proche de la maison (cour/arrière-cour, privé)
[4] Dans un trou loin de la maison dans la communauté (partagé, public)
[5] Versement dans des eaux de surface dans la communauté (lac, étang, rivière, ruisseau, public, partagé)
[6] Versement dans des eaux de surface dans la communauté (canal, canal d'irrigation, public, partagé)
[7] Versement dans de l'eau de source dans la communauté (public, partagé)
[8] Versement dans une fosse septique proche de l'habitation (cour/arrière-cour, proche de toilettes si disponibles, privé)
[9] Versement dans une fosse septique dans la communauté (partagé, public)
[10] Versement dans les égouts proches de la maison (cour/arrière-cour, privé)
[11] Versement dans les égouts dans la communauté (partagé, public)
[12] Autre, préciser ___________________________ [99] Ne sait pas [98] Pas de réponse

26 Les membres de ce ménage traitent-ils habituellement les déchets domestiques de la maison au préalable avant de les jeter ?

O 1=Oui, incinération des déchets O 3=NON
O 2=Oui, séparation/tri des déchet dégradable et non dégradables O 4=Oui, Autre, préciser _________
O 99=Ne sait pas O 98=Pas de réponse

27 À quelle distance de la source d'approvisionnement en eau de ce ménage comme indiqué à la question 20 se trouve l'emplacement de l'élimination des eaux usées, des déchets domestiques et humains/excrétions de ce ménage comme indiqué à la question 25 ?

Instructions : Indiquer un code par catégorie uniquement. Indiquer la distance en mètres [m].

Eaux usées de, par exemple, cuisine/préparation des aliments/ repas, bain et/ou ménage	Déchets domestiques	Déchets humains/déjections
Code _____________	Code _____________	Code _____________

[1] <5 m [3] ≥25 à <50 m [5] ≥100 à <500 m [7] ≥1,000 m
[2] ≥5 à <25 m [4] ≥50 à <100 m [6] ≥500 à <1,000 m [99] Ne sait pas [98] Pas de réponse

28 À quelle distance de la source d'approvisionnement en eau principale de ce ménage pour la cuisine/la préparation des aliments/des repas comme indiqué à la question 20 se trouve l'emplacement de l'élimination des eaux usées, des déchets domestiques et humains/excrétions de ce ménage comme indiqué à la question 25 ?

Instructions : Indiquer un code par catégorie uniquement. Indiquer la distance en mètres [m].

Eaux usées de par exemple cuisine/préparation des aliments/ repas, bain et/ou ménage	Déchets domestiques	Déchets humains/excréments

For peer review only - http://bmjopen.bmj.com/site/about/guidelines.xhtml

Code ____________	Code ____________	Code ____________
[1] <5 m [3] ≥25 à <50 m<br>[2] ≥5 à <25 m [4] ≥50 à <100 m	[5] ≥100 à <500 m<br>[6] ≥500 à <1,000 m	[7] ≥1,000 m<br>[99] Ne sait pas [98] Pas de réponse

ANIMAUX DE FERME/DOMESTIQUES

29 Quel genre d'animaux/de bétail et d'animaux domestiques/de compagnie les membres de ce ménage possèdent-ils ?

Instruction : Cocher plusieurs cases par catégorie si nécessaire ; il peut y avoir plus d'une réponse par catégorie.

Animaux de ferme/bétail		Animaux domestiques/de compagnie
O 1=Bovin	O9=Chameau	O 1=Chien
O 2=Mouton	O 10=Rongeurs (lapin), préciser ____________	O 2=Rongeurs (lapin), préciser ____________
O 3=Âne	O 11=Chèvre	O 3=Chat
O 4= Volaille (i.e. poulet, canard, pigeon, oie, dinde), préciser ____________	O 12=Cheval	O 4=PAS D'ANIMAUX DOMESTIQUES/DE COMPAGNIE
O 5=Zébu	O 13=PAS D'ANIMAUX DE FERME/DE BÉTAIL	O 5=Autre, préciser ____________
O 6=Cochon	O 14=Autre, préciser ____________	O 99=Ne sait pas
O 7=Poisson	O 99=Ne sait pas	O 98=Pas de réponse
O 8=Buffle	O 98=Pas de réponse	

30 Où les membres de ce ménage gardent-ils habituellement les animaux de ferme/le bétail et les animaux domestiques/de compagnie de ce ménage comme indiqué à la question 29 ?

Instruction : Indiquer un code par catégorie uniquement.

Animaux de ferme/bétail	Animaux domestiques/de compagnie
O 1=Étable/Cage/Bassin d'eau sur la propriété de l'habitation (arrière-cour/cour)	O 1=Cage/Chaîne proche de l'habitation (arrière-cour/cour)
O 2=Étable/Cage/Bassin d'eau à l'extérieur de l'habitation dans la communauté	O 2= Cage/Chaîne à l'extérieur de l'habitation dans la communauté
O 3=Étable/Cage/Bassin d'eau à l'extérieur de la communauté	O 3= Cage/Chaîne à l'extérieur de la communauté (public)
O 4=Surface libre/Eaux de surface/Eau de source sur la propriété de l'habitation (habitation, arrière-cour/cour)	O 4=Surface libre proche de l'habitation (dans la maison, arrière-cour/cour)
O 5=Surface libre/Eaux de surface/Eau de source à l'extérieur de l'habitation dans la communauté	O 5=Surface libre à l'extérieur de l'habitation dans la communauté
O 6=Surface libre/Eaux de surface/Eau de source à l'extérieur de la communauté	O 6=Surface libre à l'extérieur de la communauté
O 7=Pas applicable (nulle part /pas d'animaux de ferme/bétail)	O 7=Pas applicable (nulle part/pas d'animaux domestiques/de compagnie)
O 8=Autre, préciser __________	O 8=Autre, préciser __________
O 99=Ne sait pas	O 99=Ne sait pas
O 98=Pas de réponse	O 98=Pas de réponse

31 Quelle est l'importance de l'élevage que les membres de ce ménage pratiquent ?

O 1=Pour leurs propres besoins («élevage d'arrière-cour»)
O 2=Pas applicable (pas d'élevage)
O 3=3=Demande massive/industrielle («élevage intensif»)
O 4=Autre, préciser __________
O 99=Ne sait pas
O 98=Pas de réponse

For peer review only - http://bmjopen.bmj.com/site/about/guidelines.xhtml

32 Quel approvisionnement en eau les membres de ce ménage utilisent-ils pour fournir de l'eau aux animaux de ferme/au bétail et aux animaux domestiques/de compagnie de ce ménage comme indiqué à la question 29 ?

Instruction : Indiquer un code par catégorie uniquement.

Animaux de ferme/bétail	Animaux domestiques/de compagnie
Code ____________	Code ____________

[1] Eau du robinet (à l'intérieur de l'habitation ; privée)
[2] Eau du robinet (à l'extérieur de l'habitation : cour/arrière-cour ; privée)
[3] Eau du robinet (à l'extérieur de l'habitation ; publique/partagée)
[4] Borne-fontaine/eau courante (à l'intérieur de l'habitation ; privée)
[5] Borne-fontaine/eau courante (à l'extérieur de l'habitation : cour/arrière-cour ; privée)
[6] Borne-fontaine/eau courante (à l'extérieur de l'habitation ; publique/partagée)
[7] Camion-citerne (public ; partagé)
[8] Eaux de surface (canal, canal d'irrigation)
[9] Eaux de surface (lac, étang, rivière, ruisseau)
[10] Eau de source
[11] Eau de pluie
[12] Puits/Puits de forage (en plein air/non couvert/non protégé)
[13] Puits/Puits de forage (couvert/protégé)
[14] Pas applicable (pas d'animaux de ferme/bétail; pas d'animaux domestiques/de compagnie)
[15] Autre, préciser ________________ [99] Ne sait pas [98] Pas de réponse

33 Les membres de ce ménage se lavent-ils habituellement les mains après avoir nourri, donné de l'eau et nettoyé l'étable/la cage/le bassin d'eau des animaux de ferme/du bétail de ce ménage comme indiqué à la question 29 ?

Instruction : Indiquer un code par catégorie uniquement.

Animaux de ferme/bétail	Animaux domestiques/de compagnie
Code ____________	Code ____________

[1] Lavent les mains avec du savon et de l'eau
[2] Lavent les mains avec de l'eau uniquement
[3] Frottent les mains avec des feuilles/de la paille/de l'herbe
[4] Frottent les mains avec du sable
[5] Frottent les mains avec un chiffon/du tissu
[6] Ne lavent pas, pas besoin/pas sale
[7] Ne lavent pas, rien pour laver les mains
[8] Autre, préciser __________
[9] Pas applicable (pas d'animaux de ferme/bétail ; pas d'animaux domestiques/de compagnie
[99] Ne sait pas [98] Pas de réponse

CULTURES AGRICOLES

34 Quel genre de cultures les membres de ce ménage font-ils pousser/cultivent-ils ?

Instruction : Cocher plusieurs cases si nécessaire ; il peut y avoir plus d'une réponse.

- O 1=Café
- O 2=Riz
- O 3=Fruits (cultivés sur arbustes/arbre)
- O 4=Fruits (cultivés au sol)
- O 5=Légumes/salade (cultivés sur arbustes/arbre)
- O 6=Légumes/salade (cultivés au sol)
- O 7=Céréales (blé, orge, avoine, maïs)
- O 8=Thé
- O 9=Noix de cajou
- O 10=Tabac
- O 11=Canne à Sucre
- O 12=Chad/Chat
- O 13=Bees/Honey
- O 14=Coton
- O 15=PAS DE CULTURES
- O 16=Autre, préciser __________
- O 99=Ne sait pas
- O 98=Pas de réponse

35 Où les champs/terres agricoles des membres de ce ménage se situent-ils/elles?

- O 1=On the property of the house (i.e. back-/courtyard)
- O 4=Outside the house in the community
- O 2=Outside the community
- O 5=Not applicable (no crop farming)
- O 3=Other, specify __________
- O 99=Don't know
- O 98=No response

For peer review only - http://bmjopen.bmj.com/site/about/guidelines.xhtml

36 Quelle est l'importance des cultures des membres de ce ménage?

- O 1=Leurs propres besoins (« agriculture d'arrière-cour »)
- O 2=2=Pas applicable (pas de cultures)
- O 3=Demande massive/industrielle (agriculture intensive)
- O 4=Autre, préciser __________
- O 99=Ne sait pas
- O 98= Pas de réponse

37 Les membres de ce ménage utilisent-ils habituellement des herbicides, pesticides et engrais pour les cultures indiquées à la question 34?

Instruction : Indiquer un code par catégorie uniquement.

Herbicides	Pesticides	Engrais
O 1=Oui, préciser l'herbicide ____________	O 1=Oui, préciser le pesticide ____________	O 1=Oui, préciser l'engrais ____________
O 2=Pas applicable (pas de cultures)	O 2=Pas applicable (pas de cultures)	O 2=Pas applicable (pas de cultures)
O 3=Pas d'utilisation des herbicides	O 3=Pas d'utilisation des pesticides	O 3=Pas d'utilisation des engrais
O 99=Ne sait pas	O 99=Ne sait pas	O 99=Ne sait pas
O 98=Pas de réponse	O 98=Pas de réponse	O 98=Pas de réponse

38 Les membres de ce ménage se lavent-ils les mains après avoir travaillé dans les terres agricoles pour les cultures indiquées à la question 34 ?

- O 1=Oui, se lavent les mains avec du savon et de l'eau
- O 2=Oui, se frottent les mains avec des feuilles/de la paille/de l'herbe
- O 3=Oui, se frottent les mains avec des chiffons/du tissu
- O 4=Pas applicable (pas de cultures)
- O 5=Oui, se lavent les mains avec de l'eau uniquement
- O 6=Oui, se frottent les mains avec du sable
- O 7=Non, ne se lavent pas les mains
- O 8=Autre, préciser __________
- O 99=Ne sait pas
- O 98= Pas de réponse

For peer review only - http://bmjopen.bmj.com/site/about/guidelines.xhtml

Site ___ ___ ___ Sous-zone ___ ___ ___ Numéro d'habitation ___ ___ ___ ___ Identifiant de l'enquêteur ___ __

FORMULAIRE 4: ÉVALUATION DE L'UTILISATION GÉNÉRIQUE DES SOINS DE SANTÉ GLOBAUX, STRATIFIÉE SELON L'ÂGE ET LE SEXE

Partie A –Évaluation de l'utilisation *générique* des soins de santé

Instructions pour l'enquêteur

- Compléter ce formulaire uniquement si la personne interrogée a consenti à participer (voir « Formulaire 2 — FORMULAIRE DE CONSENTEMENT ») ; la personne interrogée ainsi que définie pour cette enquête est un adulte (selon l'âge légal de majorité du pays), un membre du ménage et un décideur en ce qui concerne les soins de santé quotidiens et l'utilisation de soins de santé pour le ménage entier et ses membres ; il ne doit pas être, par exemple, un voisin ou un visiteur.
- Se référer à « Formulaire 3 — Partie A — Informations générales sur le ménage » comme référence pour obtenir le nombre total de membres du ménage ainsi que l'âge et le sexe de chaque membre du ménage.
- Recueillir les données de tous les membres du ménage que tous les membre du ménage soient présents ou non au moment de l'entretien ; ne recueillir et n'inscrire les données que des personnes présentes au moment de l'entretien fausserait les données.
- Un ménage est défini/les membres d'un ménage sont définis comme une personne ou un groupe de personnes avec ou sans lien de parenté qui vivent ensemble dans la même unité d'habitation, qui reconnaissent un adulte de sexe masculin ou féminin comme chef de ménage, qui partagent les mêmes installations domestiques, qui sont considérés comme constituant une unité, et qui subviennent à leurs besoins en termes de nourriture et des autres éléments essentiels pour vivre.
- « Site » et « sous-zone » doivent être un numéro à trois chiffres, « numéro d'habitation » un numéro à quatre chiffres, et « identifiant de l'enquêteur » un numéro à deux chiffres (utiliser les listes préparées individuellement pour chaque site pour obtenir les codes) ; saisir « date » au format JJ/MM/AAAA et « heure » au format HH:MM et entourer MATIN ou APRÈS-MIDI. «Étiquette de l'étude» doit être une combinaison d'un code «site» à trois chiffres, d'un code «sous-zone» à trois chiffres et d'un code «numéro d'habitation» à quatre chiffres; le « identifiant de l'enquêteur » doit être un numéro à trois chiffres.
- Abréviation : EDS = Établissement de santé public/privé primaire/secondaire/tertiaire.

MEMBRES DU MÉNAGE

1 Indiquer le nombre total de membres du ménage de cette habitation (au jour de la visite).
Instructions : Le nombre total de membres du ménage doit être indiqué à l'aide d'un numéro à 2 chiffres ; voir également « Formulaire 3 — Partie A — INFORMATIONS GÉNÉRALES SUR LE MÉNAGE ».

Nombre total de membres du ménage : ____ ____

2 Indiquer le nombre de membres FÉMININS du ménage pour chacun des groupes d'âge suivants :
Instructions : Le nombre total de membres féminins du ménage par groupe d'âge doit être indiqué à l'aide d'un numéro à 2 chiffres ; voir également « Formulaire 3 — Partie A — INFORMATIONS GÉNÉRALES SUR LE MÉNAGE ».

<2 ans : ____ ____ ≥2 à <5 ans : ____ ____ ≥5 à <15 ans : ____ ____ ≥15 ans : ____ ____

3 Indiquer le nombre de membres MASCULINS du ménage pour chacun des groupes d'âge suivants :
Instructions : Le nombre total de membres masculins du ménage par groupe d'âge doit être indiqué à l'aide d'un numéro à 2 chiffres ; voir également « Formulaire 3 — Partie A — INFORMATIONS GÉNÉRALES SUR LE MÉNAGE ».

<2 ans : ____ ____ ≥2 à <5 ans : ____ ____ ≥5 à <15 ans : ____ ____ ≥15 ans : ____ ____

COMPORTEMENT DE RECHERCHE DE SOINS DE SANTÉ

4 Indiquer où les membres féminins/masculins du foyer d'âges <2 ans/≥2 à <5 ans/≥5 à <15 ans/≥15 ans recherchent habituellement des soins de santé pour les signes/symptômes suivants. Merci d'attribuer une note entre 1 et 4.

Instructions :
- Compléter la question 4 séparément pour les membres du ménage féminins et masculins d'âge <2 ans, ≥2 à <5 ans, ≥5 à <15 ans et ≥15 ans et entoure le sexe et le groupe d'âge pour lequel Q4 a été completée; passer la question 4 s'il n'y a aucun membre féminin/masculin du ménage d'âge <2 ans/ ≥2 à <5 ans/ ≥5 à <15 ans/ ≥15 ans.
- Indiquer une réponse pour chaque choix de soins de santé pour chaque signe/symptôme; attribuer une note de 1 à 4 (1=1er choix; 2=2ème choix; 3=3ème choix; 4=non choisi); indiquer un code s'il y a lieu en utilisant les listes préparées (utiliser listes préparées individuellement pour chaque site pour obtenir les codes); cocher «Ne sait pas» s'il y a lieu.

For peer review only - http://bmjopen.bmj.com/site/about/guidelines.xhtml

Signe/Symptôme	Choix de soins de santé									
	EDS SETA		Autre EDS		Médecin	Pharmacie	Guérisseur traditionnel	Nulle part Auto-médication	Nulle part Rien	Ne sait pas
	Note	Code	Note	Code	Note	Note	Note	Note	Note	Cocher
**1** [**A**] Fièvre	O=1<br>O=2<br>O=3<br>O=4		O=1<br>O=2<br>O=3<br>O=4		O=1<br>O=2<br>O=3<br>O=4	O=1<br>O=2<br>O=3<br>O=4	O=1<br>O=2<br>O=3<br>O=4	O=1<br>O=2<br>O=3<br>O=4	O=1<br>O=2<br>O=3<br>O=4	O
**2** Fièvre <3 jours (continus)	O=1<br>O=2<br>O=3<br>O=4		O=1<br>O=2<br>O=3<br>O=4		O=1<br>O=2<br>O=3<br>O=4	O=1<br>O=2<br>O=3<br>O=4	O=1<br>O=2<br>O=3<br>O=4	O=1<br>O=2<br>O=3<br>O=4	O=1<br>O=2<br>O=3<br>O=4	O
**3** Fièvre ≥3 jours (continus)	O=1<br>O=2<br>O=3<br>O=4		O=1<br>O=2<br>O=3<br>O=4		O=1<br>O=2<br>O=3<br>O=4	O=1<br>O=2<br>O=3<br>O=4	O=1<br>O=2<br>O=3<br>O=4	O=1<br>O=2<br>O=3<br>O=4	O=1<br>O=2<br>O=3<br>O=4	O
**4** Frissons Tremblements	O=1<br>O=2<br>O=3<br>O=4		O=1<br>O=2<br>O=3<br>O=4		O=1<br>O=2<br>O=3<br>O=4	O=1<br>O=2<br>O=3<br>O=4	O=1<br>O=2<br>O=3<br>O=4	O=1<br>O=2<br>O=3<br>O=4	O=1<br>O=2<br>O=3<br>O=4	O
**5** Convulsion	O=1<br>O=2<br>O=3<br>O=4		O=1<br>O=2<br>O=3<br>O=4		O=1<br>O=2<br>O=3<br>O=4	O=1<br>O=2<br>O=3<br>O=4	O=1<br>O=2<br>O=3<br>O=4	O=1<br>O=2<br>O=3<br>O=4	O=1<br>O=2<br>O=3<br>O=4	O
**6** Perte de poids	O=1<br>O=2<br>O=3<br>O=4		O=1<br>O=2<br>O=3<br>O=4		O=1<br>O=2<br>O=3<br>O=4	O=1<br>O=2<br>O=3<br>O=4	O=1<br>O=2<br>O=3<br>O=4	O=1<br>O=2<br>O=3<br>O=4	O=1<br>O=2<br>O=3<br>O=4	O
**7** Déshydratation	O=1<br>O=2<br>O=3<br>O=4		O=1<br>O=2<br>O=3<br>O=4		O=1<br>O=2<br>O=3<br>O=4	O=1<br>O=2<br>O=3<br>O=4	O=1<br>O=2<br>O=3<br>O=4	O=1<br>O=2<br>O=3<br>O=4	O=1<br>O=2<br>O=3<br>O=4	O
**8** Malaise Faiblesse/ Fatigue	O=1<br>O=2<br>O=3<br>O=4		O=1<br>O=2<br>O=3<br>O=4		O=1<br>O=2<br>O=3<br>O=4	O=1<br>O=2<br>O=3<br>O=4	O=1<br>O=2<br>O=3<br>O=4	O=1<br>O=2<br>O=3<br>O=4	O=1<br>O=2<br>O=3<br>O=4	O
**9** Douleurs (muscles/articulations /os)	O=1<br>O=2<br>O=3<br>O=4		O=1<br>O=2<br>O=3<br>O=4		O=1<br>O=2<br>O=3<br>O=4	O=1<br>O=2<br>O=3<br>O=4	O=1<br>O=2<br>O=3<br>O=4	O=1<br>O=2<br>O=3<br>O=4	O=1<br>O=2<br>O=3<br>O=4	O
**10** Maux de tête	O=1<br>O=2<br>O=3<br>O=4		O=1<br>O=2<br>O=3<br>O=4		O=1<br>O=2<br>O=3<br>O=4	O=1<br>O=2<br>O=3<br>O=4	O=1<br>O=2<br>O=3<br>O=4	O=1<br>O=2<br>O=3<br>O=4	O=1<br>O=2<br>O=3<br>O=4	O
**11** Vertiges/ Confusion Perte de connaissance	O=1<br>O=2<br>O=3<br>O=4		O=1<br>O=2<br>O=3<br>O=4		O=1<br>O=2<br>O=3<br>O=4	O=1<br>O=2<br>O=3<br>O=4	O=1<br>O=2<br>O=3<br>O=4	O=1<br>O=2<br>O=3<br>O=4	O=1<br>O=2<br>O=3<br>O=4	O
**12** Troubles de la pression artérielle	O=1<br>O=2<br>O=3<br>O=4		O=1<br>O=2<br>O=3<br>O=4		O=1<br>O=2<br>O=3<br>O=4	O=1<br>O=2<br>O=3<br>O=4	O=1<br>O=2<br>O=3<br>O=4	O=1<br>O=2<br>O=3<br>O=4	O=1<br>O=2<br>O=3<br>O=4	O
**13** Troubles cardiaques	O=1<br>O=2<br>O=3<br>O=4		O=1<br>O=2<br>O=3<br>O=4		O=1<br>O=2<br>O=3<br>O=4	O=1<br>O=2<br>O=3<br>O=4	O=1<br>O=2<br>O=3<br>O=4	O=1<br>O=2<br>O=3<br>O=4	O=1<br>O=2<br>O=3<br>O=4	O

For peer review only

For peer review only - http://bmjopen.bmj.com/site/about/guidelines.xhtml

Signe/Symptôme	Choix de soins de santé									
	EDS SETA		Autre EDS		Médecin	Pharmacie	Guérisseur traditionnel	Nulle part Auto-médication	Nulle part Rien	Ne sait pas
	Note	Code	Note	Code	Note	Note	Note	Note	Note	Cocher
**14** Nausée Vomissements	O=1<br>O=2<br>O=3<br>O=4		O=1<br>O=2<br>O=3<br>O=4		O=1<br>O=2<br>O=3<br>O=4	O=1<br>O=2<br>O=3<br>O=4	O=1<br>O=2<br>O=3<br>O=4	O=1<br>O=2<br>O=3<br>O=4	O=1<br>O=2<br>O=3<br>O=4	O
**15** Diarrhée (liquide/sanglante/ mucopurulente)	O=1<br>O=2<br>O=3<br>O=4		O=1<br>O=2<br>O=3<br>O=4		O=1<br>O=2<br>O=3<br>O=4	O=1<br>O=2<br>O=3<br>O=4	O=1<br>O=2<br>O=3<br>O=4	O=1<br>O=2<br>O=3<br>O=4	O=1<br>O=2<br>O=3<br>O=4	O
**16** Douleur abdominale Douleur intestinale	O=1<br>O=2<br>O=3<br>O=4		O=1<br>O=2<br>O=3<br>O=4		O=1<br>O=2<br>O=3<br>O=4	O=1<br>O=2<br>O=3<br>O=4	O=1<br>O=2<br>O=3<br>O=4	O=1<br>O=2<br>O=3<br>O=4	O=1<br>O=2<br>O=3<br>O=4	O
**17** Perte de sang/ Hémorragie (interne)	O=1<br>O=2<br>O=3<br>O=4		O=1<br>O=2<br>O=3<br>O=4		O=1<br>O=2<br>O=3<br>O=4	O=1<br>O=2<br>O=3<br>O=4	O=1<br>O=2<br>O=3<br>O=4	O=1<br>O=2<br>O=3<br>O=4	O=1<br>O=2<br>O=3<br>O=4	O
**18** Perte de sang/ Hémorragie (externe)	O=1<br>O=2<br>O=3<br>O=4		O=1<br>O=2<br>O=3<br>O=4		O=1<br>O=2<br>O=3<br>O=4	O=1<br>O=2<br>O=3<br>O=4	O=1<br>O=2<br>O=3<br>O=4	O=1<br>O=2<br>O=3<br>O=4	O=1<br>O=2<br>O=3<br>O=4	O
**19** Éternuements Nez qui coule	O=1<br>O=2<br>O=3<br>O=4		O=1<br>O=2<br>O=3<br>O=4		O=1<br>O=2<br>O=3<br>O=4	O=1<br>O=2<br>O=3<br>O=4	O=1<br>O=2<br>O=3<br>O=4	O=1<br>O=2<br>O=3<br>O=4	O=1<br>O=2<br>O=3<br>O=4	O
**20** Toux	O=1<br>O=2<br>O=3<br>O=4		O=1<br>O=2<br>O=3<br>O=4		O=1<br>O=2<br>O=3<br>O=4	O=1<br>O=2<br>O=3<br>O=4	O=1<br>O=2<br>O=3<br>O=4	O=1<br>O=2<br>O=3<br>O=4	O=1<br>O=2<br>O=3<br>O=4	O
**21** Respiration rapide/ essoufflement	O=1<br>O=2<br>O=3<br>O=4		O=1<br>O=2<br>O=3<br>O=4		O=1<br>O=2<br>O=3<br>O=4	O=1<br>O=2<br>O=3<br>O=4	O=1<br>O=2<br>O=3<br>O=4	O=1<br>O=2<br>O=3<br>O=4	O=1<br>O=2<br>O=3<br>O=4	O
**22** Difficultés respiratoires graves	O=1<br>O=2<br>O=3<br>O=4		O=1<br>O=2<br>O=3<br>O=4		O=1<br>O=2<br>O=3<br>O=4	O=1<br>O=2<br>O=3<br>O=4	O=1<br>O=2<br>O=3<br>O=4	O=1<br>O=2<br>O=3<br>O=4	O=1<br>O=2<br>O=3<br>O=4	O
**23** Inflammation de la peau (éruptions cutanées/ rougeurs/pus/ démangeaisons)	O=1<br>O=2<br>O=3<br>O=4		O=1<br>O=2<br>O=3<br>O=4		O=1<br>O=2<br>O=3<br>O=4	O=1<br>O=2<br>O=3<br>O=4	O=1<br>O=2<br>O=3<br>O=4	O=1<br>O=2<br>O=3<br>O=4	O=1<br>O=2<br>O=3<br>O=4	O
**24** Gonflement/ Œdème	O=1<br>O=2<br>O=3<br>O=4		O=1<br>O=2<br>O=3<br>O=4		O=1<br>O=2<br>O=3<br>O=4	O=1<br>O=2<br>O=3<br>O=4	O=1<br>O=2<br>O=3<br>O=4	O=1<br>O=2<br>O=3<br>O=4	O=1<br>O=2<br>O=3<br>O=4	O
**25** Jaunisse	O=1<br>O=2<br>O=3<br>O=4		O=1<br>O=2<br>O=3<br>O=4		O=1<br>O=2<br>O=3<br>O=4	O=1<br>O=2<br>O=3<br>O=4	O=1<br>O=2<br>O=3<br>O=4	O=1<br>O=2<br>O=3<br>O=4	O=1<br>O=2<br>O=3<br>O=4	O

For peer review only

Site ___ ___ ___ Sous-zone ___ ___ ___ Numéro d'habitation ___ ___ ___ ___ Identifiant de l'enquêteur ___ __

FORMULAIRE 4: ÉVALUATION DE L'UTILISATION GÉNÉRIQUE DES SOINS DE SANTÉ GLOBAUX, STRATIFIÉE SELON L'ÂGE ET LE SEXE

Partie B – Facteurs associés aux soins de santé/Vaccination/Perception de la maladie

<u>DÉPLACEMENT À L'ÉTABLISSEMENT DE SANTÉ</u>

1 Au cas où un établissement de santé (EDS) a été noté comme 1er choix de soins à la question 4A (fièvre), quel est habituellement le moyen de transport principal, le temps de trajet (selon le moyen de transport), le coût du trajet (selon le moyen de transport) et la distance du trajet (selon le moyen de transport) pour s'y rendre ?

Instructions : Indiquer uniquement un code par catégorie, sexe ainsi que groupe d'âge. <u>Passer</u> cette question s'il n'y a <u>pas de membre féminin/masculin d'âge <2 ans/≥2 à <5 ans/≥5 à <15 ans/≥15 ans</u> dans ce ménage (voir également questions 2 et 3 de ce formulaire Partie A). <u>Passer</u> cette question si <u>aucun EDS n'a été noté comme 1er choix de soins à la question 4A pour les membres masculins/féminins d'âge <2 ans/≥2 à <5 ans/≥5 à <15 ans/≥15 ans.</u> Indiquer le temps de trajet en minute(s) [min]/ heure(s) [h], le coût total en USD, y compris les cadeaux éventuels, les aides financières et les dons, et la distance totale en mètre(s) [m]/kilomètre(s) [km].

Membre féminin				Membre masculin			
<2 ans	≥2 à <5 ans	≥5 à <15 ans	≥15 ans	<2 ans	≥2 à <5 ans	≥5 à <15 ans	≥15 ans
**1.1 Moyen de transport**							
Code ____	Code ____	Code ____	Code ____	Code ____	Code ____	Code ____	Code ____
[1] Private transportation (i.e. motorbike, car, bus)<br>[2] Public transportation (i.e. motorbike, car, bus)				[3] Walking<br>[4] Bicycle	[5] Other, specify ________<br>[99] Don't know		[98] No response
**1.2 Temps de trajet**							
Code ____	Code ____	Code ____	Code ____	Code ____	Code ____	Code ____	Code ____
[1] <30 min<br>[2] ≥30 à <60 min		[3] ≥1.0 à <2.0 h<br>[4] ≥2.0 à <3.0 h		[5] ≥3.0 à <4.0 h<br>[6] ≥4.0h		[99] Ne sait pas<br>[98] Pas de réponse	
**1.3 Coût du transport**							
Code ____	Code ____	Code ____	Code ____	Code ____	Code ____	Code ____	Code ____
[1] <1.0 USD<br>[2] ≥1.0 à <3.0 USD		[3] ≥3.0 à <5.0 USD<br>[4] ≥5.0 à <10.0 USD		[5] ≥10.0 USD		[99] Ne sait pas<br>[98] Pas de réponse	
**1.4 Distance du trajet**							
Code ____	Code ____	Code ____	Code ____	Code ____	Code ____	Code ____	Code ____
[1] <500 m<br>[2] ≥500 m à <1.0 km		[3] ≥1.0 à <2.0 km<br>[4] ≥2.0 à <3.0 km		[5] ≥3.0 à <4.0 km<br>[6] ≥4.0 km		[99] Ne sait pas<br>[98] Pas de réponse	

<u>ASSURANCE</u>

2 Pour quels membres de ce ménage l'assurance couvre-t-elle les dépenses de santé ?

Instructions : Indiquer un code par catégorie. <u>Passer</u> cette question s'il n'y a <u>aucun membre d'âge <2 ans/≥2 à <5 ans/≥5 à <15 ans/≥15 ans</u> dans ce ménage (voir questions 2 et 3 de ce formulaire partie A).

<2 ans	≥2 à <5 ans	≥5 à <15 ans	≥15 ans
Code ____________	Code ____________	Code ____________	Code ____________
[1] Oui	[2] Non	[99] Ne sait pas	[98] Pas de réponse

3 Quels types de dépenses de santé sont habituellement couverts par l'assurance pour les membres de ce ménage ?

Instructions : Indiquer uniquement un code par catégorie et par groupe d'âge. <u>Passer</u> cette question s'il n'y a <u>aucun membre d'âge <2 ans/≥2 à <5 ans/≥5 à <15 ans/≥15 ans</u> dans ce ménage (voir également les questions 2 et 3 de ce formulaire partie

For peer review only - http://bmjopen.bmj.com/site/about/guidelines.xhtml

A). Passer cette question s'il n'y a aucune assurance qui couvre les dépenses de santé des membres d'âge <2 ans/≥2 à <5 ans/≥5 à <15 ans/≥15 ans.

	<2 ans	≥2 à <5 ans	≥5 à <15 ans	≥15 ans
	[1] Oui	[2] Non	[99] Ne sait pas	[98] Pas de réponse
**1** Inscription	Code _______	Code _______	Code _______	Code _______
**2** Consultation/Visite chez le médecin	Code _______	Code _______	Code _______	Code _______
**3** Examen physique - général	Code _______	Code _______	Code _______	Code _______
**4** Examen physique – spécifique, préciser_______	Code _______	Code _______	Code _______	Code _______
**5** Diagnostics - diagnostics généraux de laboratoire	Code _______	Code _______	Code _______	Code _______
**6** Diagnostics – diagnostics spécifiques de laboratoire, préciser__________	Code _______	Code _______	Code _______	Code _______
**7** Diagnostics Imagerie générale	Code _______	Code _______	Code _______	Code _______
**8** Diagnostics Imagerie spécifique, préciser__________	Code _______	Code _______	Code _______	Code _______
**9** Diagnostics Autre, préciser________	Code _______	Code _______	Code _______	Code _______
**10** Traitement Médication générale	Code _______	Code _______	Code _______	Code _______
**11** Traitement Médication spécifique, préciser________	Code _______	Code _______	Code _______	Code _______
**12** Traitement Autre médication, préciser__________	Code _______	Code _______	Code _______	Code _______
**13** Traitement Interventions (autres que chirurgie), préciser_______	Code _______	Code _______	Code _______	Code _______
**14** Traitement Chirurgie	Code _______	Code _______	Code _______	Code _______
**15** Hospitalisation <7 jours	Code _______	Code _______	Code _______	Code _______
**16** Hospitalisation ≥7 jours	Code _______	Code _______	Code _______	Code _______
**17** Services d'urgence	Code _______	Code _______	Code _______	Code _______
**18** Soins prénatals	Code _______	Code _______	Code _______	Code _______
**19** Soins postnatals	Code _______	Code _______	Code _______	Code _______
**20** Médecine préventive	Code _______	Code _______	Code _______	Code _______
**21** Vaccination	Code _______	Code _______	Code _______	Code _______
**22** Soins à domicile (personnes âgées, handicapés, malades chroniques/à long terme)	Code _______	Code _______	Code _______	Code _______
**23** Thérapie de soutien (physiothérapie, ergothérapie, réhabilitation)	Code _______	Code _______	Code _______	Code _______

For peer review only - http://bmjopen.bmj.com/site/about/guidelines.xhtml

ETHNOGRAPHIE

4 À quelle religion/groupe ethnique/tribu appartiennent les membres de ce ménage ?

Instructions : Donner une réponse par groupe d'âge uniquement. Passer cette question s'il n'y a aucun membre d'âge>2 ans/≥2 à <5 ans/≥5 à <15 ans/≥15 ans dans ce ménage (voir également questions 2 et 3 de ce formulaire Partie A).

	<2 ans	≥2 to <5 ans	≥5 to <15 ans	≥15 ans
Religion/Groupe ethnique/Tribu	______________	______________	______________	______________
	O 99=Ne sait pas<br>O 98=Pas de réponse	O 99=Ne sait pas<br>O 98=Pas de réponse	O 99=Ne sait pas<br>O 98=Pas de réponse	O 99=Ne sait pas<br>O 98=Pas de réponse

5 La religion/le groupe ethnique/la tribu comme indiqué à la question 4 influence-t-elle/il les différents aspects de l'utilisation des soins de santé pour les membres de ce ménage ?

Instructions : Indiquer un code par catégorie et groupe d'âge uniquement. Passer cette question s'il y a un membre d'âge >2 ans/≥2 à <5 ans/≥5 à <15 ans/≥15 ans dans ce ménage (voir également questions 2 t 3 de ce formulaire Partie A).

	<2 ans	≥2 à <5 ans	≥5 to <15 ans	≥15 ans
	[1] Oui	[2] Non	[99] Ne sait pas	[98] pas réponse
**1** Visite dans un établissement de santé (hôpital, centre de soins, poste de santé)	Code _____	Code _______	Code _______	Code _____
**2** Visite chez un médecin	Code _____	Code _______	Code _______	Code _____
**3** Visite dans une pharmacie	Code _____	Code _______	Code _______	Code _____
**4** Visite chez un guérisseur traditionnel	Code _____	Code _______	Code _______	Code _____
**5** Décision d'automédication	Code _____	Code _______	Code _______	Code _____
**6** Décision de non traitement	Code _____	Code _______	Code _______	Code _____
**7** Examen physique - général	Code _____	Code _______	Code _______	Code _____
**8** Examen physique – spécifique, préciser_______	Code _____	Code _______	Code _______	Code _____
**9** Prélèvements d'échantillons (sang, urine, selles, frottis, écouvillons, liquide cérébro-spinal)	Code _____	Code _______	Code _______	Code _____
**10** Diagnostics - diagnostics généraux de laboratoire	Code _____	Code _______	Code _______	Code _____
**11** Diagnostics – diagnostics spécifiques de laboratoire, préciser__________	Code _____	Code _______	Code _______	Code _____
**12** Diagnostics Imagerie générale	Code _____	Code _______	Code _______	Code _____
**13** Diagnostics Imagerie spécifique, préciser__________	Code _____	Code _______	Code _______	Code _____
**14** Diagnostics Autre, préciser________	Code _____	Code _______	Code _______	Code _____
**15** Traitement Médication générale	Code _____	Code _______	Code _______	Code _____
**16** Traitement Médication spécifique, préciser________	Code _____	Code _______	Code _______	Code _____
**17** Traitement Autre médication, préciser__________	Code _____	Code _______	Code _______	Code _____

For peer review only - http://bmjopen.bmj.com/site/about/guidelines.xhtml

**18** Traitement Interventions (autres que chirurgie), préciser________	Code _____	Code _______	Code _______	Code _____
**19** Traitement Chirurgie	Code _____	Code _______	Code _______	Code _____
**20** Hospitalisation <7 jours	Code _____	Code _______	Code _______	Code _____
**21** Hospitalisation ≥7 jours	Code _____	Code _______	Code _______	Code _____
**22** Services d'urgence	Code _____	Code _______	Code _______	Code _____
**23** Soins prénatals	Code _____	Code _______	Code _______	Code _____
**24** Soins postnatals	Code _____	Code _______	Code _______	Code _____
**25** Médecine préventive	Code _____	Code _______	Code _______	Code _____
**26** Vaccination	Code _____	Code _______	Code _______	Code _____
**27** Soins à domicile (personnes âgées, handicapés, malades chroniques/à long terme)	Code _____	Code _______	Code _______	Code _____
**28** Thérapie de soutien (physiothérapie, ergothérapie, réhabilitation)	Code _____	Code _______	Code _______	Code _____

VACCINATION

6 Comment les enfants de ce ménage ont-ils été nourris pendant les 6 premiers mois de leur vie ?

Instructions : Passer cette question s'il n'y a pas d'enfants de sexe masculin ou féminin dans ce ménage (voir également questions 2 et 3 de ce formulaire partie A).

- O 1=Lait maternisé exclusivement
- O 2=Lait maternisé et autres aliments, préciser ______
- O 3=Allaitement et autres aliments, préciser ________
- O 4=Lait maternisé et allaitement
- O 5=Allaitement exclusivement
- O 6=Autre, préciser _____
- O 99=Ne sait pas
- O 98=Pas de réponse

7 Quelle source d'approvisionnement en eau était-elle habituellement utilisée pour préparer le lait maternisé ou le lait maternisé et d'autres aliments comme indiqué à la question 6 pour les enfants de ce ménage ?

Instructions : Passer cette question s'il n'y a pas d'enfants dans ce ménage (voir également les questions 2 et 3 de ce formulaire Partie A).

- O 1=Eau du robinet (à l'intérieur de l'habitation, privé)
- O 2=Eau du robinet (à l'extérieur de l'habitation, arrière- cour/cour, privé)
- O 3=Eau du robinet (à l'extérieur de l'habitation, public, partagé)
- O 4=Borne-fontaine/Eau courante (à l'intérieur de l'habitation, privé)
- O 5=Borne-fontaine/Eau courante (à l'extérieur de l'habitation, arrière- cour/cour, privé)
- O 6=Borne-fontaine/Eau courante (à l'extérieur de l'habitation, public, partagé)
- O 7=Camion-citerne (public, partagé)
- O 8=Eau en bouteille
- O 9=Eaux de surface (lac, étang, rivière, ruisseau)
- O 10=Eaux de surface (canal, canal d'irrigation)
- O 11=Eau de source
- O 12=Eau de pluie
- O 13=Puits/Puits de forage (ouvert/non couvert/non protégé)
- O 14=Puits/Puits de forage (couvert/protégé)
- O 15=NO FORMULA/OTHER FEEDS PREPARED
- O 16=Autre, préciser _____________ O 99=Ne sait pas O 98=Pas de réponse

8 Est-ce que les enfants de ce ménage ont été vaccinés depuis leur naissance, y compris les vaccins de naissance ?

For peer review only - http://bmjopen.bmj.com/site/about/guidelines.xhtml

Instructions : Indiquer un code par catégorie et par enfant uniquement. Passer cette question s'il n'y a aucun enfant dans ce ménage (voir également les questions 2 et 3 de ce formulaire PartieA). Se référer à la question 9 « Formulaire 3 Partie A – Données démographiques/Informations générales sur le ménage » et indiquer les initiales/identificateur du/des membre(s).

Enfant 1<br>Initiales/Identifiant : ____	Enfant 2<br>Initiales/Identifiant : ____	Enfant 3<br>Initiales/Identifiant : ____	Enfant 4<br>Initiales/Identifiant : ____
[1] OUI, vacciné [5] Non, manque de vaccins [9] Non, EDS débordé<br>[2] Non, parent/tuteur débordé [6] Non, enfant trop jeune/vieux [10] Non, enfant malade<br>[3] Non, parent/tuteur a décidé que pas nécessaire [7] Non, parent/tuteur a refusé/trop inquiet [11] Non, pas de carte de vaccination<br>[4] Non, ne sait pas où aller [8] Non, Autre, préciser ___________ [99] Don't know [98] No response			
**8.1 Diphtérie et tétanos et coqueluche et *Haemophilus influenza* et vaccin contre l'hépatite B / DTwPHibHepB** (par exemple 6/8, 10/12, 14/16 semaines)			
Code __________	Code __________	Code __________	Code __________
**8.2 Anatoxine tétanique (TT)** (par exemple, 1er contact, grossesse, 1, 6, 12/24 mois)			
Code __________	Code __________	Code __________	Code __________
**8.3 Anatoxine tétanique et diphtérique (TD)** (par exemple, grossesse avec un premier contact, 1, 6, 12 mois)			
Code __________	Code __________	Code __________	Code __________
**8.4 Tuberculose / BCG** (à la naissance)			
Code __________	Code __________	Code __________	Code __________
**8.5 Rotavirus / RV** (par exemple 6/8, 10/12, 16 semaines)			
Code __________	Code __________	Code __________	Code __________
**8.6 Rougeole / rougeole et rubéole (MR)** (par exemple 9, 15/18 mois)			
Code __________	Code __________	Code __________	Code __________
**8.7 Polio / VPO** (par exemple naissance, 6/8, 10/12, 14/16 semaines)			
Code __________	Code __________	Code __________	Code __________
**8.8 Pneumocoque / PCV** (par exemple 6/8, 10/12, 14/16 semaines)			
Code __________	Code __________	Code __________	Code __________
**8.9 Fièvre jaune (YF)** (par exemple 9 mois)			
Code __________	Code __________	Code __________	Code __________
**8.10 Autres vaccins**			
Préciser, __________	Préciser, __________	Préciser, __________	Préciser, __________
Préciser, __________	Préciser, __________	Préciser, __________	Préciser, __________
Préciser, __________	Préciser, __________	Préciser, __________	Préciser, __________
Préciser, __________	Préciser, __________	Préciser, __________	Préciser, __________

For peer review only - http://bmjopen.bmj.com/site/about/guidelines.xhtml

PERCEPTION DE LA MALADIE

9 Connaissance, perception, causes et prévention des maladies infectieuses communes

Maladie	**9.1 Maladie connue des membres du ménage ?** [1] Oui [2] Non [99] Ne sait pas [98] Pas de réponse	**9.2 Perception de la maladie par les membres du ménage** *Instruction :* Décrire brièvement si elle est connue.	**9.3 Causes de la maladie connue des membres du ménage ?** *Instructions :* Indiquer tous les codes (liste A) qui s'appliquent; plus d'une réponse est possible	**9.4 Mesures pour prévenir la maladie connues des membres du ménage ?** *Instructions :* Indiquer tous les codes (liste B) qui s'appliquent ; plus d'une réponse est possible.	**9.5 Mesures pour prévenir la maladie utilisées par les membres du foyer ?** *Instructions :* Indiquer tous les codes (liste B) qui s'appliquent ; il peut y avoir plus d'une réponse.
**A.** Fièvre typhoïde	Code __________		Code __________	Code __________	Code __________
**B.** Choléra	Code __________		Code __________	Code __________	Code __________
**C.** Paludisme	Code __________		Code __________	Code __________	Code __________
**D.** Grippe	Code __________		Code __________	Code __________	Code __________
**E.** Méningite	Code __________		Code __________	Code __________	Code __________
**F.** Hépatite E	Code __________		Code __________	Code __________	Code __________
**G.** Tuberculose	Code __________		Code __________	Code __________	Code __________
**H.** VIH/SIDA	Code __________		Code __________	Code __________	Code __________

Liste A: Causes possibles	Liste B: Mesures préventives possibles
[1] Consommation d'alcool<br>[2] Temps/sorcellerie	[1] Pas de consommation d'alcool<br>[2] Dormir sous une moustiquaire

For peer review only - http://bmjopen.bmj.com/site/about/guidelines.xhtml

[3] Piqûre de moustique/d'insecte<br>[4] Morsure de chien<br>[5] Boire de l'eau non traitée de surface/de source (lac, étang, rivière, ruisseau, canal, canal d'irrigation)<br>[6] Contact physique étroit/baiser/embrassade<br>[7] Manger des produits alimentaires crus comme des légumes/de la salade, des fruits, des produits laitiers, des œufs, de la viande ou du poisson<br>[8] Ne pas se laver les mains correctement en préparant la nourriture/les repas<br>[9] Ne pas se laver les mains correctement avant/après être allé aux toilettes<br>[10] Contact avec du sang humain (injection, transfusion, chirurgie)<br>[11] Consommation de sang animal cru<br>[12] Autre, préciser ____________________<br>[99] Ne sait pas<br>[98] Pas de réponse	[3] Utiliser des sprays insecticides/anti-moustiques<br>[4] Maintenir la maison et ses environs immédiats propres<br>[5] Avoir une pratique correcte de l'élimination des déchets humains et domestiques<br>[6] Éviter le contact rapproché avec des chiens<br>[7] Traiter l'eau (faire bouillir, filtration, désinfection solaire, sédimentation, chloration)<br>[8] Ne pas cuisiner/préparer la nourriture/les repas avec de l'eau non traitée<br>[9] Ne pas laver les produits alimentaires à consommer crus avec de l'eau non traitée<br>[10] Faire bouillir/chauffer à nouveau les restes/les aliments/repas préparés avant de les consommer<br>[11] Ne pas manger des produits alimentaires crus comme légumes/salade, fruits, produits laitiers, œufs, viande ou poisson<br>[12] Couvrir les produits alimentaires/repas<br>[13] Protéger les produits alimentaires/repas contre les mouches<br>[14] Éviter la nourriture/les boissons des vendeurs de rue<br>[15] Éviter le contact rapproché avec les personnes malades<br>[16] Pas de contact physique étroit/baisers/embrassades<br>[17] Se couvrir le nez/la bouche en éternuant/toussant<br>[18] Pas de contact/consommation de sang humain/animal<br>[19] Avoir une bonne hygiène des mains en préparant la nourriture/les repas (se laver les mains avec du savon et de l'eau, se laver les mains par frottement)<br>[20] Avoir une bonne hygiène des mains avant/après être allé aux toilettes (se laver les mains avec du savon et de l'eau, se laver les mains par frottement)<br>[21] Autre, préciser ____________________<br>[99] Ne sait pas<br>[98] Pas de réponse

For peer review only - http://bmjopen.bmj.com/site/about/guidelines.xhtml

10 Pour quels membres de ce ménage les mesures préventives listées à la questions 9.5 contre la fièvre typhoïde sont-elles utilisées ?

Instructions : Cocher plusieurs cases si nécessaire ; il peut y avoir plus d'une réponse.

O 1=<2 ans
O 2=≥2 à < 5 ans
O 3=≥5 à <15 ans
O 4=≥15 ans
O 5=PAS UTILISÉ
O 99=Ne sait pas
O 98=Pas de réponse

11 Nommer les membres du ménage pour lesquels une fièvre typhoïde a été diagnostiquée dans les 3 derniers mois. Quels éléments de diagnostic ont été utilisés pour confirmer la fièvre typhoïde?

Instructions : Indiquer plusieurs codes si nécessaire pour chaque membre du ménage ; il peut y avoir plus d'une réponse.

Membre 1<br>Initiales/Identifiant: __	Membre 2<br>Initiales/Identifiant: __	Membre 3<br>Initiales/Identifiant: __	Membre 4<br>Initiales/Identifiant: __
[1] Hémoculture [3] Test sanguin/de Widal [5] Pas applicable (pas diagnostiqué avec la fièvre typhoïde)<br>[2] Coproculture [4] Autre test, préciser ______________ [99] Ne sait pas [98] Pas de réponse			
Code ___________	Code ___________	Code ___________	Code ___________

12 (Où les membres de ce ménage indiqués à la question 11 ont-ils été diagnostiqués pour la fièvre typhoïde?) Où les membres de ce ménage ont-ils recherché des soins de santé?

Instructions : Indiquer un code par membre du ménage uniquement.

Membre 1<br>Initiales/Identifiant: __	Membre 2<br>Initiales/Identifiant: __	Membre 3<br>Initiales/Identifiant: __	Membre 4<br>Initiales/Identifiant: __
[1] Établissement de santé, préciser _____________<br>[2] Médecin<br>[3] Nulle part, automédication<br>[4] Guérisseur traditionnel<br>[5] Pharmacien		[6] Nulle part, pas de recherche de soins<br>[7] Autre, préciser _____________<br>[8] Pas applicable<br>[99] Ne sait pas<br>[98] Pas de réponse	
Code ___________	Code ___________	Code ___________	Code ___________

13 (Les membres de ce ménage pour lesquels une fièvre typhoïde a été diagnostiquée comme indiqué à la question 11 ont-ils reçu un traitement?) Quel traitement ont-ils reçu?

Instructions : Cocher une réponse par membre du ménage uniquement.

Membre 1<br>Initiales/Identifiant: __	Membre 2<br>Initiales/Identifiant: __	Membre 3<br>Initiales/Identifiant: __	Membre 4<br>Initiales/Identifiant: __
O1=Traitement, préciser<br>________________<br>O 2=Pas applicable<br>O 99=Ne sait pas<br>O 98=Pas de réponse	O1=Traitement, préciser<br>________________<br>O 2=Pas applicable<br>O 99=Ne sait pas<br>O 98=Pas de réponse	O1=Traitement, préciser<br>________________<br>O 2=Pas applicable<br>O 99=Ne sait pas<br>O 98=Pas de réponse	O1=Traitement, préciser<br>________________<br>O 2=Pas applicable<br>O 99=Ne sait pas<br>O 98=Pas de réponse

For peer review only

For peer review only - http://bmjopen.bmj.com/site/about/guidelines.xhtml

Site ___ ___ ___ Sous-zone ___ ___ ___ Numéro d'habitation ___ ___ ___ ___ Identifiant de l'enquêteur ___ __

FORMULAIRE 5: ÉVALUATION DE L'UTILISATION RÉELLE DES SOINS DE SANTÉ GLOBAUX, STRATIFIÉE SELON L'ÂGE ET LE SEXE

Partie A: Évaluation de l'utilisation *réelle* des soins de santé

COMPORTEMENT DE RECHERCHE DE SOINS

1 Consigner ci-dessous l'apparition de signe(s) ou symptôme(s) (voir liste 1) et préciser où (voir liste 2) et comment le(s) membre(s) a/ont recherché les soins de santé durant les 3 derniers mois ?

Instructions : voir « formulaire 3- partie A-INFORMATIONS GENERALES SUR LE MENAGE » pour obtenir les initiaux/identifiants de chacun des membres du ménage. Indiquer seulement un signe/symptome par ligne. Commencer avec une nouvelle ligne pour tous nouveaux soins de santé sélectionnés pour chaque signe/symptome recurrent. Indiquer le nombre des apparitions de chaque signe/symptômes durant les 3 derniers mois. Si une différente option de soins de santé a été sélectionnée pour un signe/symptôme recurrent, indiquer cela dans une nouvelle ligne. Completer s'il vous plaît, un formulaire supplémentaire s'il y a eu plus de 20 signes/symptômes apparus durant les 3 derniers mois.

**Liste 1: Signe/Symptôme**		**Liste 2: Option de soins de santé**
[A] Fièvre<br>[B] Fièvre <3 jours (continus)<br>[C] Fièvre ≥3 jours (continus)<br>[D] Frissons/Tremblements<br>[E] Convulsion<br>[F] Perte de poids<br>[G] Déshydratation<br>[H] Malaise/Faiblees/Fatigue<br>[I] Douleurs (muscles/articulations/os)<br>[J] Maux de tête<br>[K] Vertiges/Confusion/ Perte de connaissance<br>[L] Troubles de la pression sanguine<br>[M] Troubles cardiaques	[N] Nausée/Vomissements<br>[O] Diarrhée (liquide/sanglante/mucopurulente)<br>[P] Douleur abdominale/Douleur intestinale<br>[Q] Perte de sang/Hémorragie (interne)<br>[R] Perte de sang/Hémorragie (externe)<br>[S] Éternuements Nez qui coule<br>[T] Toux<br>[U] Respiration rapide/Essoufflement<br>[V] Difficultés respiratoires graves<br>[W] Inflammation de la peau (éruptions cutanées/ rougeurs/pus/démangeaisons)<br>[X] Gonflement/Œdème<br>[Y] Jaunisse	[1] EDS-SETA, Code _______________<br>[2] Autre EDS Code _______________<br>[3] Médecin<br>[4] Pharmacie<br>[5] Guérisseur traditionnel<br>[6] Nulle part Auto-médication<br>[7] Nulle part Rien

**No.**	**Membre du ménage** Enregistrer les initiaux/identifiants du membre du ménage ; voir formulaire 3 – partie A- INFORMATIONS GENERALES SUR LE MENAGE »	**Signes/ Symptômes** Utiliser la Liste 1 pour sélectionner le code	**Options de soins de santé**		**Fréquence** Indiquer le nombre d'incidences pour le signe/symptôme listé
			Utiliser la Liste 2 pour sélectionner le code	EDS Code	
1					
2					
3					
4					
5					

For peer review only - http://bmjopen.bmj.com/site/about/guidelines.xhtml

6				
7				
8				
9				
10				
11				
12				
13				
14				
15				
16				
17				
18				
19				
20				

For peer review only

For peer review only - http://bmjopen.bmj.com/site/about/guidelines.xhtml

Appendix 4/Supplementary file 4: Geospatial random selection with application of simple, weighted-stratified sampling (examples of Ghana and Madagascar)

Figure 1 Geographic random points generated by ArcGIS for Asante Akim North and Central, Ghana

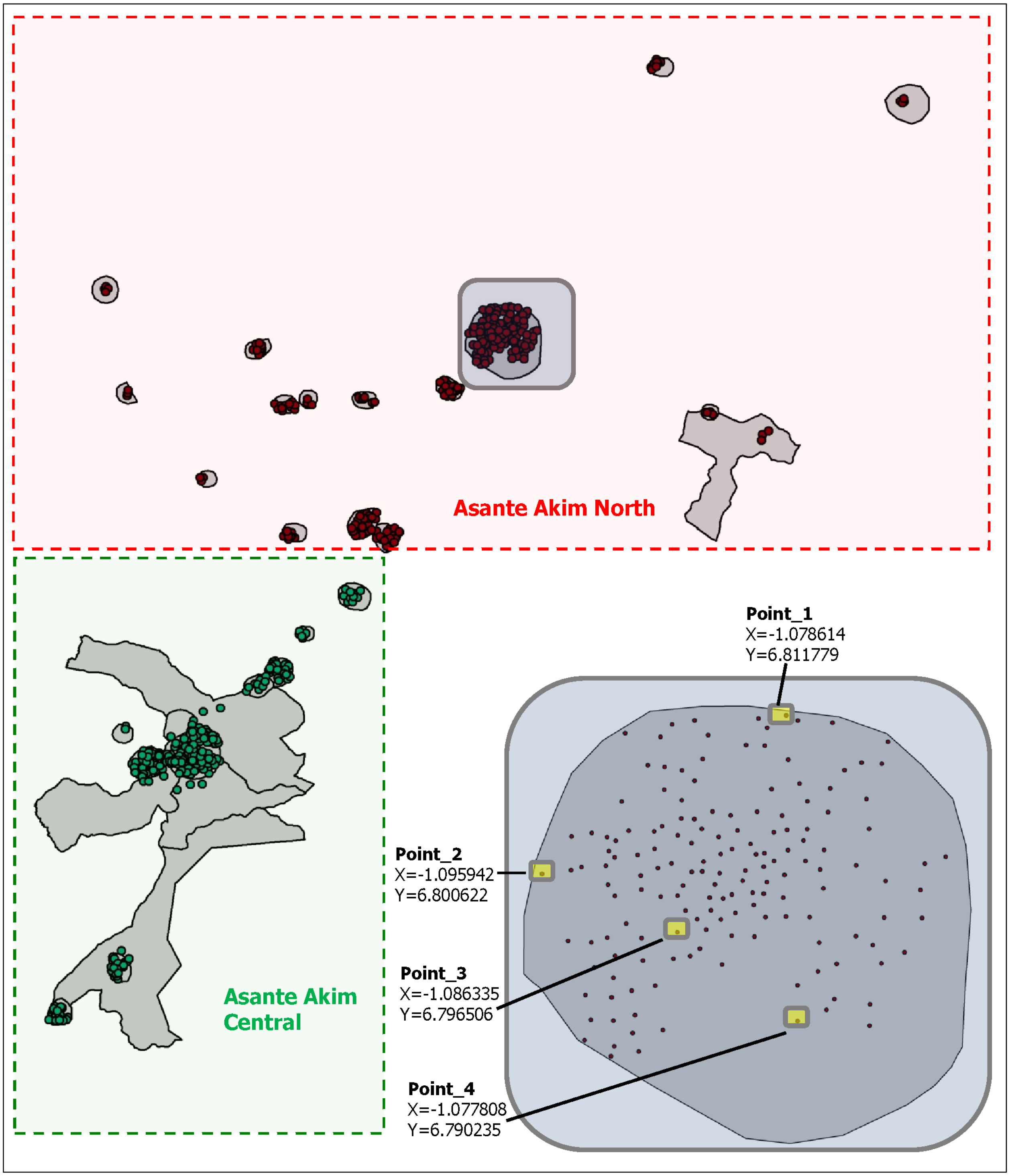

The study area of Asante Akim North (light red rectangle) and Asante Akim Central (light green rectangle) is highlighted in grey. The geographic random points are shown as red and green points for Asante Akim North and Asante Akim Central, respectively. The illustration at the bottom, right highlighted in light blue shows the enlarged area that is indicated as a small central light blue highlighted rectangle. The X- and Y-data of four geographic random points are given as examples; points are indicated as yellow highlighted rectangles. ArcGIS-generated geographic random points converted and visualized in Google Earth Pro® for Asante Akim North and Central, Ghana, can be shared on request.

Figure 2 Geographic random points generated by ArcGIS for Renivohitra/Antananarivo, Madagascar

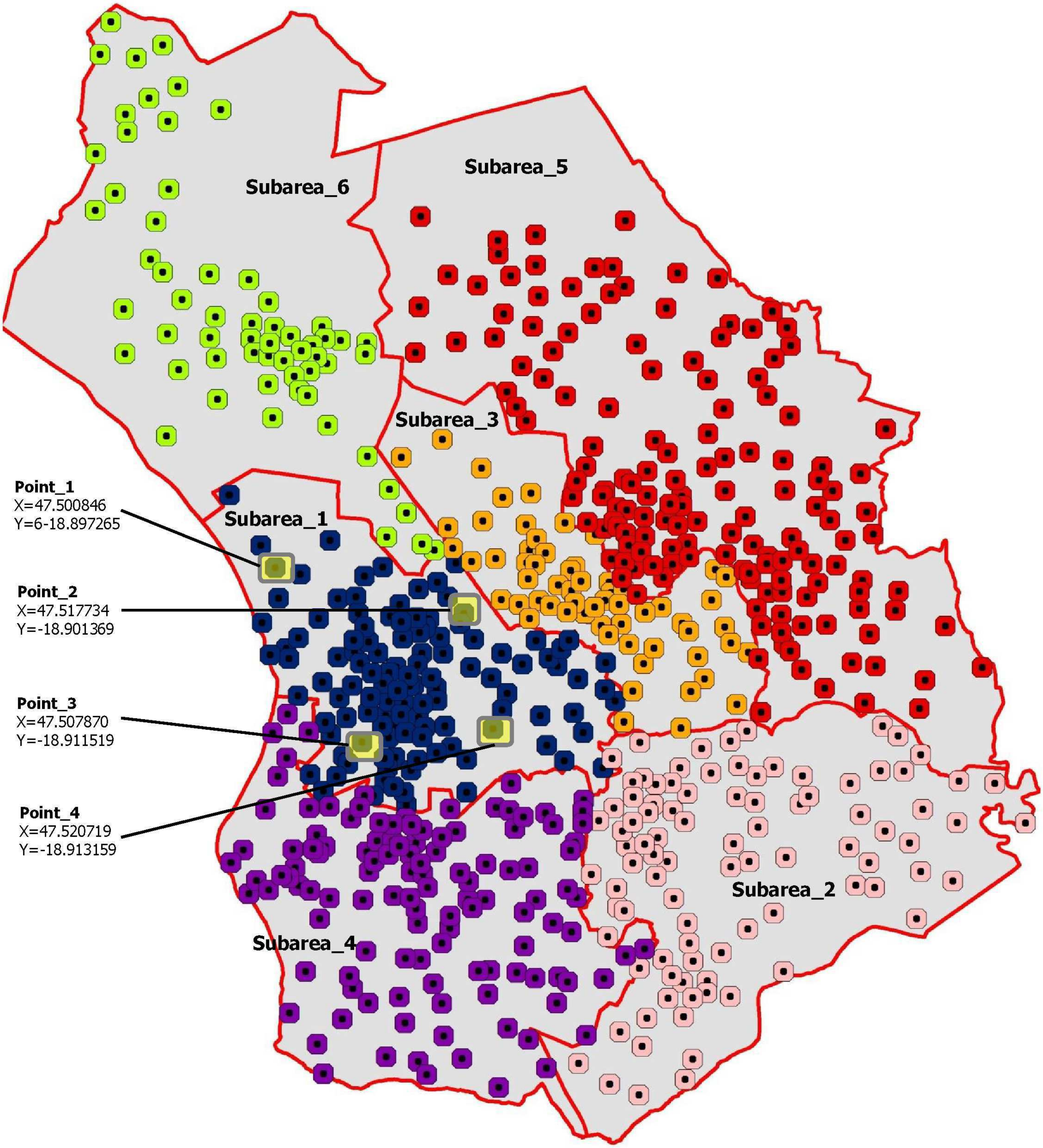

The study area of Renivohitra/Antananarivo, which is subdivided into six administrative subareas, is highlighted in grey. The geographic random points are shown as blue (subarea_1), pink (subarea_2), orange (subarea_3), purple (subarea_4), red (subarea_5) and green (subarea_6) points. The X- and Y-data of four geographic random points are given as examples; points are indicated as yellow highlighted rectangles. ArcGIS-generated geographic random points converted and visualized in Google Earth Pro® for Renivohitra/Antananarivo, Madagascar, can be shared on request.

Figure 3 ArcGIS-generated geographic random points converted and visualized in Google Earth Pro® for Asante Akim North and Central, Ghana (online, supplementary material)

Figure 3A
Random points by smallest administrative subarea visualized in Google Earth Pro® for Asante Akim Central (northern), Ghana

Figure 3B
Random points by smallest administrative subarea visualized in Google Earth Pro® for Asante Akim Central (southern), Ghana

Figure 3C
Random points by smallest administrative subarea visualized in Google Earth Pro® for Asante Akim North (western), Ghana

Figure 3D
Random points by smallest administrative subarea visualized in Google Earth Pro® for Asante Akim North (eastern), Ghana

Figure 4 ArcGIS-generated geographic random points converted and visualized in Google Earth Pro® for Renivohitra/Antananarivo, Madagascar (online, supplementary material)

Figure 4A
The Latitude and Longitude of four geographic random points shown as examples are given as degrees, decimal minutes; points are indicated as yellow highlighted rectangles.

Figure 4B
Random points visualized in Google Earth Pro® for Subarea 1, 3 and 4, Renivohitra/Antananarivo, Madagascar

Figure 4C
Random points visualized in Google Earth Pro® for Subarea 2, Renivohitra/Antananarivo, Madagascar

Figure 4D
Random points visualized in Google Earth Pro® for Subarea 5, Renivohitra/Antananarivo, Madagascar

Figure 4E
Random points visualized in Google Earth Pro® for Subarea 6, Renivohitra/Antananarivo, Madagascar

For peer review only

Appendix 5/Supplementary file ~~5~~7: System diagram of the data collection and data management platform: the Android application "HPA Collect" and the platform "HPA Web"

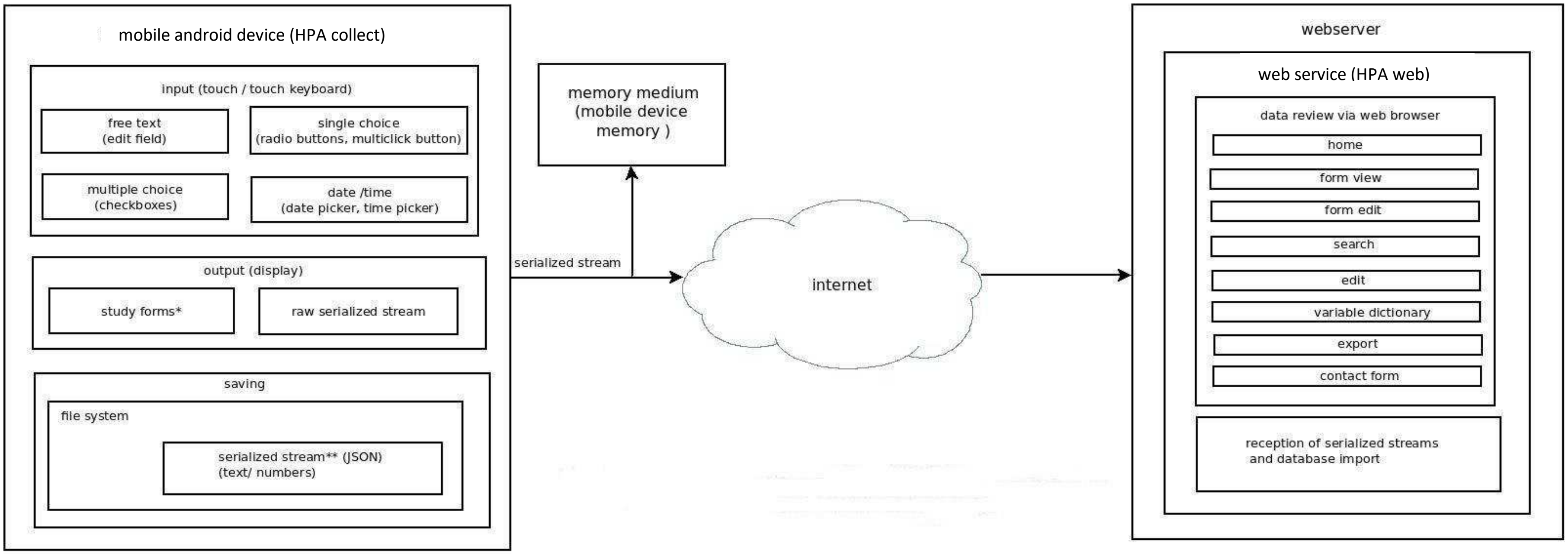

Note. *study forms: Form 1, Form 3 Part A to C, Form 4 Part A to B, Form 5; **serialized stream: two- or more-dimensional data (e.g. a table) is brought to a one-dimensional form; the application uses the JSON (Java Script Object Notation) format.

For peer review only - http://bmjopen.bmj.com/site/about/guidelines.xhtml

BMJ Open

The HPAfrica Study Protocol: Assessment of health behavior and population-based socio-economic, hygiene behavioral factors - a standardized repeated cross-sectional study in multiple cohorts in sub-Saharan Africa

Journal:	*BMJ Open*
Manuscript ID	bmjopen-2017-021438.R2
Article Type:	Protocol
Date Submitted by the Author:	27-Jun-2018
Complete List of Authors:	Pak, GiDeok; International Vaccine Institute, Biostatistics & Data Management<br>Haselbeck, Andrea; International Vaccine Institute, Epidemiology<br>Seo, Hyeong-Won; International Vaccine Institute, Biostatistics & Data Management<br>Osei, Isaac; Kumasi Centre for Collaborative Research in Tropical Medicine, Kwame Nkrumah University of Science and Technology (KNUST)<br>Amuasi, John; Kumasi Centre for Collaborative Research in Tropical Medicine, Kwame Nkrumah University of Science and Technology (KNUST)<br>Breiman, Robert; Emory University , Global Health Institute<br>Cruz Espinosa, Ligia Maria; International Vaccine Institute, Epidemiology<br>Holm, Marianne; International Vaccine Institute, Epidemiology<br>Im, Justin; International Vaccine Institute, Epidemiology<br>Jang, Geun-Hyeog; International Vaccine Institute, Biostatistics & Data Management<br>Jeon, Hyonjin; International Vaccine Institute, Epidemiology<br>Luby, Stephen; Stanford University Division of Infectious Diseases and Geographic Medicine<br>Lunguya-Metila, Octavie; Institut National de Recherche Biomedicales; Service de Microbiologie, Cliniques Universitaires de Kinshasa<br>MacWright, William; Global Health Institute, Emory University<br>Mogeni, Ondari; International Vaccine Institute, Epidemiology<br>Okeke, Iruka; University of Ibadan<br>Owusu-Dabo, Ellis; Kwame Nkrumah Univeristy of Science and Technology, School of Public Health<br>Park, Jin Kyung; International Vaccine Institute, Biostatistics & Data Management<br>Park, SeEun; International Vaccine Institute, Epidemiology<br>Popoola, Femi; University of Ibadan<br>Seo, Hye-jin; International Vaccine Institute, Epidemiology<br>Soura, Abdramane ; Institut Superieur des Sciences de la Population, University of Ouagadougou<br>Teferi, Mekonnen; Armauer Hansen Research Institute, ALERT Campus<br>Toy, Trevor; International Vaccine Institute, Epidemiology<br>Chon, Yun; International Vaccine Institute, Biostatistics & Data Management

	Rafindrakalia, Mathilde; Universite d'Antananarivo<br>Rakotozandrindrainy, Raphael; Universite d'Antananarivo<br>Meyer, Christian; Institute of Tropical Medicine, Eberhard Karls University; Faculty of Medicine, Duy Tan University<br>Marks, Florian ; International Vaccine Institute, Epidemiology; The Department of Medicine, The University of Cambridge<br>Panzner, Ursula; International Vaccine Institute, Epidemiology
**Primary Subject Heading**:	Epidemiology
Secondary Subject Heading:	Epidemiology, Global health, Health services research, Infectious diseases, Public health
Keywords:	health/hygiene behavior, sanitation, socio-economic, sub-Saharan Africa, HPAfrica study, population sampling frame/spatial sampling

SCHOLARONE™
Manuscripts

The HPAfrica Study Protocol: Assessment of health behavior and population-based socio-economic, hygiene behavioral factors - a standardized repeated cross-sectional study in multiple cohorts in sub-Saharan Africa

Short Title

The **H**ealth **P**opulation **Africa** (HPAfrica) study

Authors

Gi-Deok Pak[1#], Andrea Haselbeck[1#], Hyeong-Won Seo[1], Isaac Osei[2], John Amuasi[2], Robert F. Breiman[3], Ligia Maria Cruz Espinoza[1], Marianne Holm[1], Justin Im[1], Geun-Hyeog Jang[1], Hyon-Jin Jeon[1], Stephen Luby[4], Octavie Lunguya-Metila[5,6], William MacWright[3], Ondari D. Mogeni[1], Iruka N. Okeke[7], Ellis Owusu-Dabo[8], Jin-Kyung Park[1], Se Eun Park[1], Oluwafemi Popoola[7], Hye-Jin Seo[1], Abdramane Bassiahi Soura[9], Mekonnen Teferi[10], Trevor Toy[1], Yun Chon[1], Mathilde Rafindrakalia[11], Raphael Rakotozandrindrainy[11], Christian G. Meyer[12,13], Florian Marks[1,14*] and Ursula Panzner[1]

Affiliations

1. International Vaccine Institute, Seoul, Republic of Korea
2. Kumasi Centre for Collaborative Research in Tropical Medicine, Kwame Nkrumah University of Science and Technology (KNUST), Kumasi, Ghana
3. Global Health Institute, Emory University, Atlanta, United States
4. Infectious Diseases and Geographic Medicine, Stanford University, Stanford, United States
5. Institut National de Recherche Biom ́edicales, Kinshasa, Democratic Republic of the Congo
6. Service de Microbiologie, Cliniques Universitaires de Kinshasa, Kinshasa, Democratic Republic of the Congo
7. University of Ibadan, Ibadan, Nigeria
8. School of Public Health, Kwame Nkrumah Univeristy of Science and Technology, Kumasi, Ghana
9. Institut Supérieur des Sciences de la Population, University of Ouagadougou, Ouagadougou, Burkina Faso
10. Armauer Hansen Research Institute, Jimma Road, ALERT Campus, Addis Ababa, Ethiopia
11. University of Antananarivo, Antananarivo, Madagascar
12. Institute of Tropical Medicine, Eberhard Karls University, Tübingen, Germany
13. Faculty of Medicine, Duy Tan University, Da Nang, Vietnam
14. The Department of Medicine, The University of Cambridge, Cambridge, United Kingdom

# Contributed equally

*Corresponding Author:

Florian Marks, MPH, PhD, International Vaccine Institute, SNU Research Park, 1 Gwanak-ro, Gwanak-gu, Seoul, 08826, Republic of Korea, Phone: +82-2-881-1133, Fax: +82-2-881-1164, E-mail: fmarks@ivi.int

For peer review only - http://bmjopen.bmj.com/site/about/guidelines.xhtml

Abstract

Introduction

The objective of the **H**ealth **P**opulation **Africa** (HPAfrica) study is to determine health behavior and population-based factors, including socio-economic, ethnographic, hygiene and sanitation factors, at sites of the Severe Typhoid Fever in Africa (SETA) program. SETA aims to investigate healthcare facility-based fever surveillance in Burkina Faso, the Democratic Republic of the Congo, Ethiopia, Ghana, Madagascar, and Nigeria. Meaningful disease burden estimates require adjustment for health behavior patterns, which are assumed to vary among a study population.

Methods and Analysis

For the minimum sample size of household interviews required, the assumptions of an infinite population, a design effect, and age- and sex-stratification are considered. In the absence of a population sampling frame or household list, a spatial approach will be used to generate geographic random points with an Aeronautical Reconnaissance Coverage Geographic Information System (ArcGIS) tool. Printouts of Google Earth Pro® satellite imagery visualize these points. Data of interest will be assessed in different seasons by applying population-weighted stratified sampling. An Android-based application and a web service will be developed for electronic data capturing and synchronization with the database server in real time. Sampling weights will be computed to adjust for possible differences in selection probabilities. Descriptive data analyses will be performed in order to assess baseline information of each study population and age- and sex-stratified health behavior. This will allow adjusting disease burden estimates. In addition, multivariate analyses will be applied to look into associations between health behavior, population-based factors, and the disease burden as determined in the SETA study.

For peer review only - http://bmjopen.bmj.com/site/about/guidelines.xhtml

Strengths and limitations of this study

- Standardization in community-based multi-country/-site research requires cautiousness considering variations in setting, language and culture.
- Large-scale public health assessments will complement disease burden data by investigating sources of transmission and infection.
- Current population data in study sites and their administratively-defined subareas and population sampling frames are assumed to be limited.
- Electronic data collection may cause technical problems related to damage, functional failure, or loss of devices.
- Unstable wireless internet connection may limit data teams to update the Android-based application, communicate with study managers in real time, and synchronize collected data with the server.

Registration: Not required

Keywords: health/hygiene behavior, sanitation, socio-economic, population sampling frame/spatial sampling, sub-Saharan Africa, HPAfrica study

For peer review only

For peer review only - http://bmjopen.bmj.com/site/about/guidelines.xhtml

Introduction

Assessment of health behavior, including healthcare utilization, is an important determinant for the generation of accurate disease burden estimates among target populations. This is particularly critical for studies which utilize observational, healthcare facility-based surveillance designs as substantial variation in health behavior patterns strongly influences disease burden estimates.[1] The adjustment of disease burden calculations for variation in health behavior will increase the accuracy of measures of disease occurrence caused by bacterial, fungal, viral, and parasitic pathogens among populations under investigation. Furthermore, the influence of a large variety of population-based factors on both health behavior and disease burden has been recognized. This includes socioeconomic factors such as education, occupation, income, the availability of household assets,[2, 3] ethnical/religious and cultural factors,[4, 5] but also hygiene and sanitation facilities,[6-10] food handling,[11-14] and animal contacts.[9]

The **H**ealth **P**opulation **Africa** (HPAfrica) study will be conducted in Burkina Faso, the Democratic Republic of the Congo (DRC), Ethiopia, Ghana, Madagascar, and Nigeria. Experiences from the Typhoid Fever Surveillance in Africa Program (TSAP) will be transferred to the Severe Typhoid in Africa (SETA) program.[15, 16] In this program, standardized, healthcare facility-based fever surveillance is performed at selected study sites in participating countries over a two-year period.[17] SETA primarily aims to systematically collect information on the burden of severe *Salmonella* infections. Subjects living in defined catchment areas with either an acute fever episode, a fever history of ≥3 consecutive days, clinically-suspected typhoid fever or clinically-diagnosed gastrointestinal perforations due to typhoid fever will be eligible. In addition, the severity of *Salmonella* caused illnesses and the natural history of *Salmonella* infection will be recorded with further analyses on host immune responses and chronic carriage.

Objectives and Outcomes

The goal of the HPAfrica study is to assess the *generic* and *actual* age- and sex-stratified health

For peer review only - http://bmjopen.bmj.com/site/about/guidelines.xhtml

behavior. *Generic* health behavior pertains to behavior in conditions associated with fever and other signs and symptoms unrelated to onsets, whereas *actual* health behavior is related to onsets (Appendix 1/Supplementary file 1; Appendix 2/Supplementary file 2: Form 4 Part A, Form 5; Appendix 3/Supplementary file 3). These data will be used to adjust age- and sex-stratified measures of disease occurrence like incidences of infectious pathogens in the proportion of a study population not captured by the SETA surveillance program. Data documented for various signs and symptoms or a combination of these will be used for a gradual classification of healthcare utilization based on disease severity.

The HPAfrica study will, moreover, collect data on healthcare-associated factors such as travel modalities, possession of a health insurance, cultural/ethnographic factors, immunization status among children,[18, 19] and the frequency of occurrence and individual perception of selected diseases (Appendix 2/Supplementary file 2: Form 4 Part B; Appendix 3/Supplementary file 3:Form 4 Part B). Demographic data (Appendix 2/ Supplementary file 2: Form 3 Part A; Appendix 3/Supplementary file 3, Form 3 Part A) will be used for age- and sex-stratification of the population surveyed, which is required to compute adjustment factors for measures of disease occurrence. Socio-economic data (Appendix 2/ Supplementary file 2: Form 3 Part B; Appendix 3/ Supplementary file 3: Form 3 Part B) will be utilized to calculate wealth indices for descriptive and analytical approaches. Information on hygiene and sanitation facilities (Appendix 2/ Supplementary file 2: Form 3 Part C; Appendix 3/Supplementary file 3: Form 3 Part C) may provide a better understanding of the occurrence and frequency of pathogens identified among study populations.

Methods

Study sites

The boundaries of each site will be defined using pre-existing information set by statistical authorities or ministries of health (Table 1) coupled with open-source high-resolution geospatial data. A retrospective review of records of SETA recruitment healthcare facilities from the past two years will

For peer review only - http://bmjopen.bmj.com/site/about/guidelines.xhtml

support the re-definement of site limits. It is expected that this review will reveal the residences or at least the broader administrative residential areas such as communities, districts or villages of patients who sought healthcare for any reason. Maps and satellite imagery may be used to better visualize patients' residences and the overall boundaries, including the geographically- or administratively-defined subareas, census enumeration areas or strata, of each site. Where boundaries cannot be clearly defined, additional factors such as the distance to a recruitment healthcare facility may be considered.

For peer review only

Table 1: Background data of participating study sites of target countries

Country	Study site	Setting*	Approximate total site population (year)	Approximate age-stratified site population				Approximate sex-stratified site population	
				<2y	≥2y to <5y	≥5y to <15y	≥15y	Male	Female
Burkina Faso	Nioko II[1]	Urban	19,251 (2017)	2,394	1,977	4,657	10,223	9,321	9,930
	Polesgo[1]	Rural	7,897 (2017)	934	893	1,808	4,262	3,856	4,041
	Ouagadougou[1]	Urban	2,532,311 (2015)	421,429		693,723	1,417,159	1,271,302	1,261,009
DRC	Kisantu[2]	Urban	291,252 (2017)	48,043		243,209		91,598[#]	99,231[#]
Ethiopia	Wolayita Zone[3]	Urban/ Rural	1,968,735 (2017)	100,800	206,520	635,015	1,026300	964,577	1,004,058
	Wolayita Sodo[3]	Urban/ Rural	120,288 (2017)	6,161	12,617	38,807	62,703	59,898	60,390
	East Shewa & Arsi Zone[3]	Urban/ Rural	3,249,722 (2017)	104,408	422,939	1,016,917	1,705,458	1,671,699	1,578,023
	Adama Wenji[3]	Urban/ Rural	53,540 (2017)	3,330	7,204	17,028	25,978	27,199	26,341
Ghana	Asante Akim North & Central[4]	Urban/ Rural	140,694 (2010)	11,606	8,363	35,618	85,107	67,673	73,021
	Kumasi (Metropolis)[4,5]	Urban	1,730,249 (2010)	52,516	178,575	421,834	1,077,324	826,479	903,770
Madagascar	Antananarivo[6] (Renivohitra)	Urban	1,247,025 (2009)	N/A[$]	N/A[$]	N/A[$]	N/A[$]	N/A[$]	N/A[$]
	Imerintsiatosika[7]	Rural	44,669 (2016)	3,582	4,449	7,610	29,028	N/A[$]	N/A[$]
Nigeria	Ibadan[8]	Urban	1,343,147 (2006)	176,110		305,656	861,381	661,818	681,329

Note. Table 1 shows population data that were available at the time of the HPAfrica protocol writing; population data and boundaries of geographically and/or administratively defined study sites may be subject to changes during the course of the study. *The classification of sites by country is based on best local knowledge; #The sex-stratification is based on a total population of 190,829; $Population data not available at the time of protocol writing; y= years of age.

Source of population data: **Burkina Faso** ([1]http://www.insd.bf/n/; [2]http://www.indepth-network.org/member-centres/ouagadougou-hdss); **DRC** ([2]Kisantu Central Health Zone Office report, 2016); **Ethiopia** ([3]Health Management Information System of the Ethiopian Ministry of Health (zonal and district health offices); **Ghana** ([4]Ghana Statistical Service, 2010 Population & Housing Census, Asante Akim Central Municipality; [5]Ghana Statistical Service, 2010 Population & Housing Census, Summary report of final results); **Madagascar** ([6]Population par Fokotany selon la declaration des Chefs Fokotany: Source: Donnee de la cartographie censitaire mises-a-jour en juillet 2009 - INSTAT/DDSS; [7]University of Antananarivo); **Nigeria** ([8]Federal Republic of Nigeria 2006 Population and Housing Census (Table DS5), National Population Commission, Abuja, Nigeria).

For peer review only - http://bmjopen.bmj.com/site/about/guidelines.xhtml

Sample size

All available sources will be used to gather most up-to-date age- and sex-stratified population data by study site and its administratively- or geographically-defined subareas or strata. This may include latest demographic information from census population totals or a Demographic Surveillance System (DSS)/Health and Demographic Surveillance System (HDSS). Population summary figures and population growth factors, if available, and open-access sampling tools coupled with population data sources like density-based gridded population data may also be used.[20-24]

All strata per study site will be included. A household as defined for the HPAfrica study constitutes the primary sampling unit (PSU). The minimum number of randomly selected households to be interviewed will be calculated using the precision-based equation (Equation-I) assuming an infinite population and considering a design effect (DEFF),[25-27] including its accompanying assumptions:

Equation-I: Precision-based sample size calculation by study site for an infinite population [25-31]

$$n_0 = DEFF \cdot \left[z^2{}_{1-\frac{\alpha}{2}} \cdot \frac{4 \cdot p \cdot (1-p)}{d^2} \right]$$

<u>Assumptions Equation-I</u>

n_0	Minimum total number of households to be interviewed in a study area assuming an infinite population
$DEFF$	Design effect; set at 1.5<br>≤1.0=negative correlation of the outcome(s) of interest between household members; 1.0=no correlation of the outcome(s) of interest between household members; ≥1.0=positive correlation of the outcome(s) of interest between household members
$z_{1-\frac{\alpha}{2}}$	Normal deviation corresponding to a 95% confidence interval (1.96 for alpha of 0.05)
d	Precision (acceptable error); point estimation; set at 0.2
p	Proportion of the study population expected to visit a recruitment healthcare facility for conditions associated with fever and other signs and symptoms (proportion captured); set at 0.2
$1-p$	Proportion of the study population expected to not visit a recruitment healthcare facility for conditions associated with fever and other signs and symptoms (proportion not captured)

The design effect is defined as an adjustment factor for the natural clustering of health behavior as the main outcome of interest among household members. It accounts for greater statistical variance and, therefore, lower precision compared to simple random sampling.[25, 29, 32, 33] For HPAfrica, the DEFF may be set conservatively at 1.5 based on an estimate of 1.42 resulting from an average household size of seven and an Intracluster Correlation Coefficient (ICC) of 0.07 from the TSAP

For peer review only

For peer review only - http://bmjopen.bmj.com/site/about/guidelines.xhtml

study.[15, 30, 31, 34] A proportion p of 0.2 may be assumed if no other estimates are available, or p may be based on more precise information available by site or on experiences taken from the TSAP study (Table 2): Madagascar, Isotry: p =0.01, Burkina Faso, Polesgo: p =0.9.[35] Combining all assumptions into Equation-I will result in a total minimum number (n_0) of 92 household interviews per site.

In addition, n_0 will be accounted for the age- and sex-distribution of a study population reflected by q, the assumed minimum proportion of stratification per age group (<5 years, ≥5 to <15 years, ≥15 years). Factor q may be arbitrarily set at 0.2 or may be based on previous experiences: Ethiopia, Butajira: q=0.09, Burkina Faso, Polesgo: q=0.23.[15] Applying q to Equation-II for a binomial distribution, including its accompanying assumptions, will result in the age-stratified minimum number of household interviews ($\overline{n_0}$) of 461 (Equation-II):

Equation-II: Precision-based sample size calculation by study site for an infinite population considering age-stratification and population-weight by subarea or stratum:

$$\overline{n_0} = \left(DEFF \cdot \left[z^2_{1-\frac{\alpha}{2}} \cdot \frac{4 \cdot p \cdot (1-p)}{d^2}\right]\right)/q \qquad \overline{n_{0w}} = \overline{n_0} \cdot (n/N)$$

<u>Assumptions Equation-II</u>

$\overline{n_0}$	Minimum total number of households to be interviewed in the study area assuming an infinite population and age-stratification
$\overline{n_{0w}}$	Minimum number of households to be interviewed by subarea or stratum
n/N	Population size of subarea or stratum divided by the population size of the total study area
$DEFF$	Design effect; set at 1.5
$z_{1-\frac{\alpha}{2}}$	Normal deviation corresponding to a 95% confidence interval (1.96 for alpha of 0.05)
d	Precision (acceptable error); point estimation; set at 0.2
p	Proportion of the study population expected to visit a recruitment healthcare facility for conditions associated with fever and other signs and symptoms (proportion captured); set at 0.2
$1-p$	Proportion of the study population expected to not visit a recruitment healthcare facility for conditions associated with fever and other signs and symptoms (proportion not captured)
q	The minimum proportion of stratification per age group; set at 0.2

Health behavior will be assessed twice at the same households if possible or at alternative households in case the study population is instable due to migration and/or high birth/death rates. Additional households, assumed to be 20% on average, (Table 2) may be added to account for possible loss to follow-up between the two assessments. Eventually, population-weighted stratified sampling with

For peer review only

For peer review only - http://bmjopen.bmj.com/site/about/guidelines.xhtml

probability proportional to population size (PPPS) to account for unequal selection probability due to inhomogeneous population distributions between strata (population-weight (n/N) by stratum) will be applied to $\overline{n_0}$ of 553.[36]

Table 2: Sample sizes considering infinite population, differing estimates for p, age-stratification and loss to follow-up by study site applying Equation-II:

Proportion (p)	Total minimum number of households without DEFF	Total minimum number of households (n_0) with DEFF=1.5	Total minimum number of households ($\overline{n_0}$) with DEFF=1.5 and q=0.2	$\overline{n_0}$ adjusted for loss to follow-up
0.1	35	52	259	311
0.2	62	92	461	553
0.3	81	121	605	726
0.4	92	138	691	830
0.5	96	144	720	864
0.6	92	138	691	830
0.7	81	121	605	726
0.8	62	92	461	553
0.9	35	52	259	311

Abbreviations
p=Proportion of the study population expected to visit a recruitment healthcare facility for conditions associated with fever and other signs and symptoms (proportion captured); set at 0.2
DEFF=Design effect; set at 1.5
n_0=Total minimum number of households to be interviewed in a study area assuming an infinite population
$\overline{n_0}$=Total minimum number of households to be interviewed in the study area assuming an infinite population and age-stratification
q=Minimum proportion of stratification per age group; set at 0.2

Sampling with and without population sampling frame

If a comprehensive up-to-date population sampling frame or household list exists through DSS/HDSS or census, computerized selection of households as PSUs will be performed using SAS (Statistical Analysis System, version 9.4, SAS Institute, Cary, NC) applying PPPS population-weighted stratified serial simple random selection without replacement. Selected households labeled with identifiers are visualized using DSS/HDSS or census tools. In case of refusal, absence of a respondent or locating of an abandoned household, interviewers are trained to visit an alternative household closest to the right or left side of an original household. This follows the principle of closest proximity as seen in previous research.[37-40] To limit, furthermore, chances of unequal selection probability, thus chances of selection bias by interviewers, we pre-choose an arbitrary number of five alternative

For peer review only - http://bmjopen.bmj.com/site/about/guidelines.xhtml

households from the population sampling frame that require to be in closest proximity to the original household rather than applying for instance a lottery-based or clockwise spiral outward selection.[36, 37, 40] In addition, scoring from one (=closest) to five (=furthest) is applied to alternative households and interviewers are instructed to strictly follow the scoring sequence during the household selection procedure. Compliance of interviewers with the rules given for selecting original and alternative households will be verified on a daily basis. Deviations will be investigated and re-training provided to interviewers as required.

If no population sampling frame exists we will apply a PPPS population-weighted stratified spatial sampling technique.[36] Guided by previous research conducted, random spatial points are generated using the ArcGIS (Aeronautical Reconnaissance Coverage Geographic Information System; Redlands, CA, USA, version 10.2) random point generation tool.[21, 23, 36, 39, 41-46] The tool randomly places a number of points inside the feature of a polygon that corresponds to an administrative subarea or stratum. X- and Y-coordinates define one point until the calculated sample size by stratum is reached. ArcGIS-generated points are converted into an image file of the Keyhole Markup Language (KML) or Keyhole Markup language Zipped (KMZ) format and imported into Google Earth Pro®. A unique numerical identifier and geographic coordinates are assigned to each point. Appendix 4/Supplementary file 4 illustrates our approach utilizing examples from Ghana and Madagascar. Spatial points labeled with identifiers are pictured on poster-sized printouts of (60x60 to 60x90 cm) of Google Earth Pro® satellite imagery with high resolution or alternatively using an open-source application for offline/online automated navigation and mapping to locate points on the ground. Global Positioning System (GPS) receivers (Garmin-eTrex; Garmin Ltd., Lenexa, KS, USA) allow verifying the locations. The receivers will be positioned closely to a located point, in a static position and an open area to assure barrier-free reading. Interviewers assess spatial points for the presence of a household as the PSUs.[24] In case of refusal, absence of a respondent or locating of a non-residential point, interviewers are trained to visit a pre-selected alternative spatial point closest to the right or left side of an original point and assess it for the presence of a household.[37-39, 47] To limit chances of

For peer review only - http://bmjopen.bmj.com/site/about/guidelines.xhtml

unequal selection probability, we pre-select at least five alternative spatial points in closest proximity to the original point that represent a structure of appropriate size and rectangular or square shape from the Google Earth Pro® satellite imagery using the in-built distance measurement tool. Same as for sites with a population sampling frame, scoring from one to five is applied to alternative spatial points and interviewers are instructed to follow the scoring sequence. Compliance of interviewers with the rules given for selecting original and alternative households will be verified on a daily basis. Deviations will be investigated and re-training provided to interviewers as required. In the event that a spatial point is placed in equidistance to two (or more) structures, the interviewer skips the original spatial point and chooses a pre-selected alternative spatial point following the scoring sequence. In case two (or more) original spatial points are placed on the same structure, the interviewer assesses the structure for the presence of a household and chooses a second (or more) pre-selected alternative spatial point following the scoring sequence.

Irrespective of the presence or absence of a population sampling frame, a single-family, single-story household will be approached directly for study participation. However, one household only is enrolled at a multi-family, single-story structure based on following procedures: the interviewer enters the structure and assesses the total number of households; the first household on the right/left side (depending on the inner construction) of the structure is approached; if the first household does not participate, the second household on the right/left side of the same structure is approached. The interviewer continues until one household by structure is enrolled. Similarly, one household only is enrolled at a multi-family, multi-story structure. After assessing the total number of households present, the interviewer approaches the first household nearest to the entrance; if the first household does not participate, the second household closest to the entrance on the same/subsequent floor (depending on the inner construction) is approached; the interviewer continues until one household by structure is enrolled. The interviewer chooses a pre-selected alternative spatial point following the scoring sequence in case no household can be enrolled.

For peer review only - http://bmjopen.bmj.com/site/about/guidelines.xhtml

Frequency of data collection

Residential points will be visited twice for household interviews during the SETA program to assess seasonal influences on health behavior.[48-52] Interviews will take place in differing seasons – one at the end of the dry season or at least one month after its beginning and one towards the end of the wet season or at least one month after its beginning (Table 3). Currently, the HPAfrica study is ongoing in Burkina Faso, Ghana and Madagascar and is anticipated to be continued in the remaining countries during the upcoming months.

Table 3: Seasonality in participating countries

Country	Period of Wet Season	Period of Dry Season
Burkina Faso	May/June - September/October	October/November - April/May
DRC	November – March	April - October
Ethiopia	June – August	October - May
Ghana	April – July, September – November	December - March
Madagascar	November - March/April	April/May - October
Nigeria	May – August	October - April

Note. The seasonality by country is based on best local knowledge.

Inclusion and exclusion criteria

Household members of all ages and both sexes living in a study area at the day of the interview will be eligible for inclusion. A household will be excluded if the designated respondent declines participation or is unavailable after three consecutive visits. All visitors and individuals with unknown residence or residence outside the study area will be excluded.

For the purpose of this investigation, a household is defined as a person or a group of related or unrelated persons living in the same dwelling unit, acknowledging one adult individual as the household head, sharing the same housekeeping arrangements, and independently procuring food and other essential for living.[35]

The interview will exclusively be held with the respondent, who is an adult household member at the country-specific legal age of majority. This person may be identified as decision-maker by members

For peer review only - http://bmjopen.bmj.com/site/about/guidelines.xhtml

of the same household and serves as a proxy for an entire household.[15] Further relevant definitions are explained in Appendix 1/Supplementary file 1 and Appendix 3/Supplementary file 3.

Data collection

On-site interviewers will be trained on locating the geographic points, identification of respondents, informed consent procedures with emphasis on voluntary participation, and the deployment of standardized, pre-tested study forms (Appendix 2/Supplementary file 2 and Appendix 3/Supplementary file 3) prior to the initiation of HPAfrica. Informed consent and study forms will be forward-translated into the country-specific official language by two independent bilingual translators fluent in English and native speakers of the target language who are familiar with the concept and terminology of the forms. Both translations will be compared for discrepancies together with the translators and a coordinator, and consensus will be sought. The synthesized forward translation will be back-translated from the target language into English by two independent translators blinded to the original forms. Consensus on discrepancies of translations and the original forms will be sought together with the translators and a coordinator. Translated forms will be pilot-tested among a convenience sample of households of the target population prior to their finalization to assure cross-cultural comprehensibility and semantic, idiomatic, experiential and conceptual equivalence. A translation report will be prepared.[53, 54]

Data management

Management of data will depend on the mode of collection, which will be primarily electronic rather than paper-based. Past experiences showed following disadvantages of paper-based data collection: a high probability of errors while filling study forms, and the need of subsequent data digitalization, which is an extra, labor- and time-intensive and error-prone step. Electronic data capturing using an application for a mobile device like smartphone or tablet minimizes these limiting factors to the data quality.

For peer review only - http://bmjopen.bmj.com/site/about/guidelines.xhtml

There may be a pilot period during which both paper-based and paperless data will be collected prior to full implementation of electronic data collection. The latter utilizes the Android application "HPA Collect" (Google Android 5.0.1 9API 23; available at https://play.google.com/store/apps/details?id=anint.ivi.hpa) and the platform "HPA Web" (CSS, JavaScript and JSP; available at http://hpa.ivi.int/), which are accompanied by a variable dictionary containing codes, meaning and properties of variables in at least English and French. Both, "HPA Collect" and "HPA Web", will be designed specifically for the purpose of this study. IVI's expertise in computer engineering allows the fundamental construction, configuration and development of components required. Different from many open-source programs, a server installation feature enables the storage of all original data collected on the institutional server besides extra features for deep-error checking, logical relations between variables and forms, search functions, and analyzing and displaying of data collected. Stable wireless internet connectivity using default browsers is required to assure a contemporaneous, attributable, original and accurate synchronization of data between "HPA Collect" and "HPA Web". Access to both "HPA Collect" and "HPA Web" is password-protected. Appendix 5/Supplementary file 5 displays the system diagram, which has the following features:

Structure or features of "HPA Collect"

- **User management:** A simple user interface provides different privileges for users to enter, edit (except for study labels), and export or transfer data. It establishes an audit trail that records when users enter data into the server.
- **Data input:** Data are entered directly into the smartphone or tablet device. Entry fields limit possible answers to plausible ones only by offering different input methods:
    - Edit Text: free text
    - Radio group/Multi click button: single choice
    - Check box: multiple choice
    - Date and time picker: entering date and time
    - Spinner: drop-down button

For peer review only

For peer review only - http://bmjopen.bmj.com/site/about/guidelines.xhtml

Additional features allow the surveyor to save time by transferring data collected at an earlier stage to data fields required at a later stage of the interview by performing plausibility checks for diverse data input fields, and by easily recognizing skip patterns. Error checks, missing data notifications, code errors, and logical errors are also included.

- **Data view:** Raw, originally entered data can be seen in the same, human- and machine-readable format, namely the JSON format, as they will be transferred to a database server (Windows, MySQL). Additional functions like "clear" to clear all records, "reset" to return back to the main data view page, and "search" to search for a study label are available.
- **Export:** All entered data will be sent to the IVI server through wireless internet connection. Each form record having a unique study label of the household enrolled is transformed into a SQL insertion query and inserted onto the server only if there is no duplicated study label found. Insertion of data will be ignored in case of duplicated study labels.
- **Export to local:** All data entered will be backed-up threefold on a public storage folder of the mobile device as JSON and txt formats. Txt files contain data of each individual form. One version of JSON files contain data by each individual form and a second version of JSON files contain all data of a household enrolled. Data in the JSON format can be extracted by USB or wireless internet connection.
- **Report:** This feature allows a user to report any query to the leading "HPA Collect" administrator at IVI by e-mail or a third-party application.
- **Settings:** The ability to switch the system language between English and French is provided.
- **About:** The currently installed version of "HPA Collect" is displayed.

Structure or features of "HPA Web"

- **Home:** An introduction to the platform is given and a tutorial in French and English will

For peer review only - http://bmjopen.bmj.com/site/about/guidelines.xhtml

be accessible.

- **Form View:** The study forms are displayed separately showing every question and the corresponding input data as defined variables.
- **Form Edit:** This feature allows the correction of the uploaded data. It is only accessible to data managers in each participating country and at IVI.
- **Search:** Specific study labels given to each study form can be filtered.
- **Variable dictionary:** The variable dictionary can be downloaded.
- **Export:** Data can be converted from the server into a transferrable excel file.
- **Contact Us:** Quick and easy correspondence between users and the data manager at IVI is given.

Data will be reviewed by key study personnel on a day-to-day basis and checked for consistency and accuracy prior to data analysis. The quality of data may be checked by selecting a subset of 5% for validation against the original, paper-based data if possible. Names of household members will not be linked to study forms of HPAfrica and will not be recorded in the database in order to ensure confidentiality. Access to the database will be restricted to authorized study personnel only and data will be kept in a locked, protected location. Periodic interim backup files and at least three final backup files of the database stored in a secure, locked place will avoid loss of data and ensure data safety. All data will be stored for a minimum of five years.

Data analysis

Combined sampling weights will be computed to adjust for possible differences in selection probabilities and to increase the accuracy of study estimates.[22, 55] Weights are generated in a multi-level approach to consider selection probabilities of e.g. households, inverse weights within households (i.e., multi-family single-story structure, multi-family multi-story structure) and inverse weights within household members participating in the HPAfrica study.[21, 39] Open-source tools, census data and HPAfrica-generated data such as the population size, the number of households or

For peer review only

For peer review only - http://bmjopen.bmj.com/site/about/guidelines.xhtml

structures, the number of households within multi-family/multi-story structures and the household size by stratum will be used to compute sampling weights.[56, 57]

Descriptive analyses such as absolute and relative frequencies, medians or arithmetic means will be conducted to construct a contemporary baseline population description. Basic analyses will include the calculation of age- and sex-stratified proportions of *generic* and *actual* health behavior, including healthcare utilization, for various signs and symptoms or combinations of signs and symptoms. These analyses will allow for a gradual classification based on disease severity, including 95% confidence interval. Health behavior will be adjusted for actual clustering among household members. Most up-to-date stratified population data by site and demographic data derived from HPAfrica will be used to generate the proportions for the adjustment of incidence calculations.

Socio-economic information collected will be used to construct wealth indices of studied populations by applying factor analyses based on the principal component method. In addition, bi- and multivariate regression analyses will be performed to identify potential confounding factors or effect modification using factor scores.

Bi- and multivariate regression analyses will be carried out using factor scores for the assessment of the following variables:

- Socioeconomic data such as education, occupation,[58] health insurance, housing, and household assets
- Hygiene and sanitation such as toilet access, water sources, food handling, waste disposal, and animal contacts
- Health behavior: *actual* (age- and sex-stratified, severity, frequency), *generic* (age- and sex-stratified, severity, rating by choice)
- Travel modalities to health facilities, age-stratified
- Seasonality related to hygiene & sanitation, health behavior, travel modalities

For peer review only - http://bmjopen.bmj.com/site/about/guidelines.xhtml

- Cultural/religious/ethnographic factors, age-stratified
- Vaccination status among children[18, 19]
- Knowledge and perception of common diseases

Ethical considerations

All study participants are required to provide written informed consent. There are no direct benefits for enrolled households and their members. Indirect benefits for the general population at sites will be the updated information on the burden of communicable diseases, including factors influencing disease transmission and infection. The HPAfrica study generates evidence to support decision-makers on the introduction of appropriate measures for disease prevention and control.

This protocol was first approved by the Institutional Review Board of the International Vaccine Institute in October 2016 and undergoes annual review and renewal also by Ethics Committees of collaborating institutions (Ethics References are listed in Appendix1/Supplementary file1).

During and after the HPAfrica study, all data of enrolled subjects will be kept in strict confidence and will not be disclosed to a third party by any member of the research team. Password protection of devices and the database is used for strict confidence. All paper-based data (consent forms) will be kept in a secure, locked location. Confidential information stored on computers and paper-based data sources will only be made available to co-investigators and IVI staff directly involved in study activities of HPAfrica.

Patient and Public Involvement

No patients but household members in selected study sites will be involved. They did not participate in the development of the research question, outcome measures and the study design. However, we attempt to balance well between answering our study objectives and benefiting the general population at the sites by disseminating information on the disease burden and factors influencing disease

For peer review only - http://bmjopen.bmj.com/site/about/guidelines.xhtml

transmission and infection derived from our study. Household members will contribute to the study conduct by direct participating in the gathering of data. They will not be involved in recruitment procedures. The households selected for participation will be identified purely by chance due to the random sampling applied. Study findings will be disseminated to study populations directly through collaborating institutions and indirectly through publications in peer-reviewed journals.

Dissemination

We plan to disseminate the study findings by reporting descriptive as well as analytic and stratified data through publications in peer-reviewed journals and collaborating institutions.

Authors' contributions

GDP, JKP and UP conceptualized the initial study concept of the HPAfrica study. HWS and GHJ developed the tools "HPA Collect" and "HPA Web" for electronic data collection with primary support of UP and AH. IO and JA supported the development and finalizing of the data collection tools "HPA Collect" and "HPA Web". MR and RR supported the development and finalization of the HPAfrica study forms. The appendices were conceptualized by GDP, JKP, YC, JI, TT, AH and UP. IO, JA, RB, LMCE, JI, HJJ, SL, OLM, WMW, ODM, INO, EOD, SP, OP, HJS, ABS, MT, TT, MR and RR supported the further development of the initial study protocol and appendices. FM, YC and CGM participated in the finalization of the study protocol, including appendices. AH wrote the first draft of the manuscript. GDP, HWS, IO, JA, RB, LMCE, MH, JI, GHJ, HJJ, SL, OLM, WMW, ODM, INO, EOD, JKP, SP, OP, HJS, ABS, MT, TT, YC, MR, RR, CGM and FM critically reviewed and revised the manuscript draft. GDP, AH, HWS, IO, JA, RB, LMCE, MH, JI, GHJ, HJJ, SL, OLM, WMW, ODM, INO, EOD, JKP, SP, OP, HJS, ABS, MT, TT, YC, MR, RR, CGM, FM and UP agreed and approved the final manuscript as submitted.

Acknowledgement

For peer review only - http://bmjopen.bmj.com/site/about/guidelines.xhtml

We thank David Kauderer for the support and advice in computer engineering, Dr. Paul M. Tshiminyi for the support in proofreading French study materials, and Soo Young Kwon and Ji Hyun Han for administrative support. We are grateful to community leaders and site representatives for their assistance and facilitation in properly approaching household members for our study.

Funding statement

This study was supported by the Bill & Melinda Gates Foundation (OPP1127988). The funders had no role in study design, data collection, data analysis, data interpretation, or writing of the report; the conclusions and findings presented are those of the authors and do not necessarily reflect positions or policies of the Bill & Melinda Gates Foundation or the International Vaccine Institute (IVI). The IVI acknowledges its donors, including the Republic of Korea and the Swedish International Development Cooperation Agency (Sida).

Competing interests

The authors declare that they have no competing interests.

Abbreviations

JSON: JavaScript Objective Notation; CSS: Cascading Style Sheets; JSP: Java Server Page; SQL: Structured Query Language

Appendices

Appendix1 (Supplementary file 1)

Ethic References

Definitions/Terminology (English)

STROBE Statement—Checklist of items that should be included in reports of cross-sectional studies

Appendix 2 (Supplementary file 2)

For peer review only

517 Study Forms (English)

518 Form 1: Record of household visits

519 Form 2: Consent Form

520 Form 3: General household information

521 Part A: Demographic data/General household information

522 Part B: Socio-economic data

523 Part C: Hygiene and sanitation

524 Form 4: Assessment of age- and sex-stratified *generic* healthcare utilization

525 Part A: Assessment of *generic* healthcare utilization

526 Part B: Healthcare-associated factors/Vaccination/Disease perception

527 Form 5: Assessment of age- and sex-stratified *actual* healthcare utilization

528 **Appendix 3** (Supplementary file 3)

529 Protocol, Definitions/Terminology and Study Forms (French)

530 **Appendix 4** (Supplementary file 4)

531 Geospatial random selection (examples of Ghana and Madagascar)

532 **Appendix 5** (Supplementary file 5)

533 System diagram of the data collection and data management: the Android application "HPA Collect" and the platform "HPA Web".

535

536 **References**

538 1. Bigogo, G., et al., *Health-seeking patterns among participants of population-based morbidity surveillance in rural western Kenya: implications for calculating disease rates.* Int J Infect Dis, 2010. **14**(11): p. e967-73.

541 2. Jordan, H.T., et al., *A comparison of population-based pneumonia surveillance and health-seeking behavior in two provinces in rural Thailand.* Int J Infect Dis, 2009. **13**(3): p. 355-61.

543 3. Saeed, B.I., et al., *Impact of socioeconomic status and medical conditions on health and healthcare utilization among aging Ghanaians.* BMC Public Health, 2015. **15**: p. 276.

545 4. Thoa, N.T., et al., *The impact of economic growth on health care utilization: a longitudinal study in rural Vietnam.* Int J Equity Health, 2013. **12**: p. 19.

For peer review only - http://bmjopen.bmj.com/site/about/guidelines.xhtml

5. Okafor, I.P., et al., *Orthodox versus unorthodox care: A qualitative study on where rural women seek healthcare during pregnancy and childbirth in Southwest, Nigeria.* Malawi Med J, 2014. **26**(2): p. 45-9.

6. Farag, T.H., et al., *Seeking care for pediatric diarrheal illness from traditional healers in Bamako, Mali.* Am J Trop Med Hyg, 2013. **89**(1 Suppl): p. 21-8.

7. Akullian, A., et al., *Environmental Transmission of Typhoid Fever in an Urban Slum.* PLoS Negl Trop Dis, 2015. **9**(12): p. e0004212.

8. Steele, A.D., et al., *Challenges and Opportunities for Typhoid Fever Control: A Call for Coordinated Action.* Clin Infect Dis, 2016. **62 Suppl 1**: p. S4-8.

9. Tran, H.H., et al., *Risk factors associated with typhoid fever in Son La province, northern Vietnam.* Trans R Soc Trop Med Hyg, 2005. **99**(11): p. 819-26.

10. Rweyemamu, M., et al., *Development of a One Health National Capacity in Africa : the Southern African Centre for Infectious Disease Surveillance (SACIDS) One Health Virtual Centre Model.* Curr Top Microbiol Immunol, 2013. **366**: p. 73-91.

11. Slayton, R.B., K.A. Date, and E.D. Mintz, *Vaccination for typhoid fever in sub-Saharan Africa.* Hum Vaccin Immunother, 2013. **9**(4): p. 903-6.

12. Sharma, P.K., et al., *Risk factors for typhoid in Darjeeling, West Bengal, India: evidence for practical action.* Trop Med Int Health, 2009. **14**(6): p. 696-702.

13. Eguale, T., et al., *Non-typhoidal Salmonella serotypes, antimicrobial resistance and co-infection with parasites among patients with diarrhea and other gastrointestinal complaints in Addis Ababa, Ethiopia.* BMC Infect Dis, 2015. **15**: p. 497.

14. Paudyal, N., et al., *Prevalence of foodborne pathogens in food from selected African countries - A meta-analysis.* Int J Food Microbiol, 2017. **249**: p. 35-43.

15. Panzner, U., et al., *Utilization of Healthcare in the Typhoid Fever Surveillance in Africa Program.* Clin Infect Dis, 2016. **62 Suppl 1**: p. S56-68.

16. von Kalckreuth, V., et al., *The Typhoid Fever Surveillance in Africa Program (TSAP): Clinical, Diagnostic, and Epidemiological Methodologies.* Clin Infect Dis, 2016. **62 Suppl 1**: p. S9-S16.

17. Park, S.E., et al., *The Severe Typhoid in Africa Program: a multicentre population-based surveillance program with a nested case-control and cohort study.* under submission.

18. WHO. *WHO vaccine-preventable diseases: monitoring system. 2017 global summary* 2017 [cited 2017 December 17]; Available from: http://apps.who.int/immunization_monitoring/globalsummary/schedules/.

19. WHO, *WHO recommendations for routine immunization - summary tables*, 2017.

20. *GridSample*. accessed June 2018]; Available from: www.gridsample.org.

21. Thomson, D.R., et al., *Modelling strategic interventions in a population with a total fertility rate of 8.3: a cross-sectional study of Idjwi Island, DRC.* BMC Public Health, 2012. **12**: p. 959.

For peer review only

For peer review only - http://bmjopen.bmj.com/site/about/guidelines.xhtml

22. Thomson, D.R., et al., *GridSample: an R package to generate household survey primary sampling units (PSUs) from gridded population data.* Int J Health Geogr, 2017. **16**(1): p. 25.

23. Galway, L., et al., *A two-stage cluster sampling method using gridded population data, a GIS, and Google Earth(TM) imagery in a population-based mortality survey in Iraq.* Int J Health Geogr, 2012. **11**: p. 12.

24. Muñoz, J. and W. Langeraar, *A census-independent sampling strategy for a household survey in Myanmar. .* 2013.

25. Fleiss, J.L., Levin, B., Paik, M.C, *Statistical Methods for Rates and Proportions*. 3rd ed2003, New York: John. Wiley & Sons.

26. Henderson, R.H., et al., *Assessment of vaccination coverage, vaccination scar rates, and smallpox scarring in five areas of West Africa.* Bull World Health Organ, 1973. **48**(2): p. 183-94.

27. Henderson, R.H. and T. Sundaresan, *Cluster sampling to assess immunization coverage: a review of experience with a simplified sampling method.* Bull World Health Organ, 1982. **60**(2): p. 253-60.

28. Chakraborty, H., et al., *A simulation based technique to estimate intracluster correlation for a binary variable.* Contemp Clin Trials, 2009. **30**(1): p. 71-80.

29. Segura-Correa, J.C., et al., *Intraherd correlation coefficients and design effects for bovine viral diarrhoea, infectious bovine rhinotracheitis, leptospirosis and neosporosis in cow-calf system herds in North-eastern Mexico.* Prev Vet Med, 2010. **96**(3-4): p. 272-5.

30. Katz, J. and S.L. Zeger, *Estimation of design effects in cluster surveys.* Ann Epidemiol, 1994. **4**(4): p. 295-301.

31. van Breukelen, G.J. and M.J. Candel, *Calculating sample sizes for cluster randomized trials: we can keep it simple and efficient!* J Clin Epidemiol, 2012. **65**(11): p. 1212-8.

32. Kaiser, R., et al., *Using design effects from previous cluster surveys to guide sample size calculation in emergency settings.* Disasters, 2006. **30**(2): p. 199-211.

33. Katz, J., et al., *Estimation of design effects and diarrhea clustering within households and villages.* Am J Epidemiol, 1993. **138**(11): p. 994-1006.

34. Newcombe, R.G., *Two-sided confidence intervals for the single proportion: comparison of seven methods.* Stat Med, 1998. **17**(8): p. 857-72.

35. Panzner, U., et al., *Geospatial sampling frames for household-based studies: Lessons learned from a multi-country surveillance network in Senegal, South Africa and Sudan.* 2018 (submitted).

36. Kumar, N., *Spatial Sampling Design for a Demographic and Health Survey.* Population Research and Policy Review, 2007. **26**(5/6): p. 581-599.

37. Siri, J.G., et al., *A census-weighted, spatially-stratified household sampling strategy for urban malaria epidemiology.* Malar J, 2008. **7**: p. 39.

For peer review only

For peer review only - http://bmjopen.bmj.com/site/about/guidelines.xhtml

38. Sollom, R., et al., *Health and human rights in Chin State, Western Burma: a population-based assessment using multistaged household cluster sampling.* PLoS Med, 2011. **8**(2): p. e1001007.

39. Shannon, H.S., et al., *Choosing a survey sample when data on the population are limited: a method using Global Positioning Systems and aerial and satellite photographs.* Emerg Themes Epidemiol, 2012. **9**(1): p. 5.

40. Grais, R.F., A.M. Rose, and J.P. Guthmann, *Don't spin the pen: two alternative methods for second-stage sampling in urban cluster surveys.* Emerg Themes Epidemiol, 2007. **4**: p. 8.

41. Lohr, S.L., *Sampling: Design and Analysis*. 2nd edition ed2010, Pacific Grove, CA: Duxbury Press.

42. Lister, A.J. and C.T. Scott, *Use of space-filling curves to select sample locations in natural resource monitoring studies.* Environ Monit Assess, 2009. **149**(1-4): p. 71-80.

43. Keating, J., et al., *A geographic sampling strategy for studying relationships between human activity and malaria vectors in urban Africa.* Am J Trop Med Hyg, 2003. **68**(3): p. 357-65.

44. Keating, J., et al., *Characterization of potential larval habitats for Anopheles mosquitoes in relation to urban land-use in Malindi, Kenya.* Int J Health Geogr, 2004. **3**(1): p. 9.

45. ArcGIS. October 2017]; Available from: http://pro.arcgis.com/en/pro-app/tool-reference/data-management/create-random-points.htm.

46. Wong, W.C., et al., *Prevalence and risk factors of chlamydia infection in Hong Kong: A population-based geospatial household survey and testing.* PLoS One, 2017. **12**(2): p. e0172561.

47. Kondo, M.C., et al., *A random spatial sampling method in a rural developing nation.* BMC public health, 2014. **14**: p. 338.

48. Kasper, M.R., et al., *Infectious etiologies of acute febrile illness among patients seeking health care in south-central Cambodia.* Am J Trop Med Hyg, 2012. **86**(2): p. 246-53.

49. Dewan, A.M., et al., *Typhoid Fever and its association with environmental factors in the Dhaka Metropolitan Area of Bangladesh: a spatial and time-series approach.* PLoS Negl Trop Dis, 2013. **7**(1): p. e1998.

50. Naeem Khan, M., et al., *Typhoid fever in paediatric patients in Quetta, Balochistan, Pakistan.* Pak J Med Sci, 2013. **29**(4): p. 929-32.

51. Crump, J.A., et al., *Estimating the incidence of typhoid fever and other febrile illnesses in developing countries.* Emerg Infect Dis, 2003. **9**(5): p. 539-44.

52. Osei-Tutu, B. and F. Anto, *Trends of reported foodborne diseases at the Ridge Hospital, Accra, Ghana: a retrospective review of routine data from 2009-2013.* BMC Infect Dis, 2016. **16**: p. 139.

53. Koller, M., et al., *Translation procedures for standardised quality of life questionnaires: The European Organisation for Research and Treatment of Cancer (EORTC) approach.* Eur J Cancer, 2007. **43**(12): p. 1810-20.

For peer review only

54. Acquadro, C., et al., *Literature review of methods to translate health-related quality of life questionnaires for use in multinational clinical trials.* Value Health, 2008. **11**(3): p. 509-21.

55. Milligan, P., A. Njie, and S. Bennett, *Comparison of two cluster sampling methods for health surveys in developing countries.* Int J Epidemiol, 2004. **33**(3): p. 469-76.

56. Kamanga, A., et al., *Open-source satellite enumeration to map households: planning and targeting indoor residual spraying for malaria.* Malar J, 2015. **14**: p. 345.

57. Elsey, H., et al., *Addressing Inequities in Urban Health: Do Decision-Makers Have the Data They Need? Report from the Urban Health Data Special Session at International Conference on Urban Health Dhaka 2015.* J Urban Health, 2016. **93**(3): p. 526-37.

58. (ILO), I.L.O. *International Standard Classification of Occupations ISCO-08, 2012.* [cited accessed December 2017; Available from: http://www.ilo.org/wcmsp5/groups/public/---dgreports/---dcomm/---publ/documents/publication/wcms_172572.pdf.

For peer review only

For peer review only - http://bmjopen.bmj.com/site/about/guidelines.xhtml

Appendix 1/Supplementary file 1:
Ethic References
Definitions/Terminology (English)
STROBE Statement—Checklist of items that should be included in reports of *cross-sectional studies*

Ethics References

- IVI Institutional Review Board (IRB), No. 2016-003
- Institute of Tropical Medicine Antwerp Institutional Review Board, Belgium
- Universiteit Antwerpen, Comite voor medische Ethiek, Belgium
- Ministère de la Santé du Burkina Faso – Comité d'Ethique pour la Recherche en Santé, Burkina Faso
- Comité d'Ethique de l'Ecole de Santé Publique de l'Université de Kinshasa, Democratic Republic of Congo
- National Research Ethics Review committee (NRERC) Ministry of Science and Technology, Federal Democratic Republic of Ethiopia
- AHRI-ALERT Ethics Review Committee (AAERC), Ethiopia
- Kwame Nkrumah University of Science and Technology, School of Medical Sciences/Komfo Anokye Teaching Hospital, Committee on Human Research, Publication and Ethics, Ghana
- Ministère de la Santé du Repoblikan'l Madagaskar – Comité d'Ethique
- University of Ibadan/University College Hospital Ethics Committee, Ibadan, Nigeria

Definitions/Terminology (English)

**A**	
Abdominal pain/ Intestinal pain	Pain (acute/short-lived or chronic/long-lived) that occurs anywhere between the chest and the groin (often referred to as the stomach region/belly).
Aches/Pain	A condition of having continuous/prolonged dull pain in a part of the body; the physical feeling, suffering, or discomfort caused by an illness or injury.
**B**	
Bleeding (internal/external)/ Blood loss	The condition of losing blood from the vascular system, either internally into the body (internal bleeding) or externally through a natural orifice or break in the skin (external bleeding); blood escaping from an injured blood vessel.
Blood loss/ Bleeding	The condition of losing blood from the vascular system, either internally into the body (internal bleeding) or externally through a natural orifice or break in the skin (external bleeding); blood escaping from an injured blood vessel.
Blood pressure disorders	A high/low blood pressure or a blood pressure that shows excessive fluctuation.
**C**	
Chills/Shivering	The sensation of coldness that could be accompanied by shivering or shaking of the body and pallor of the skin.
Cholera	An infectious, sometimes fatal disease of the small intestine caused by the bacterium *Vibrio cholerae*; it is spread via the fecal-oral route from the ingestion of contaminated water and food and causes chills, shivering, severe watery diarrhea, vomiting, and dehydration.
Confusion/Dizziness/ Unconsciousness	The state of being bewildered or unclear in mind; an abnormal state of response to stimuli in the environment; faint, wooziness, weakness, unsteadiness or a tendency to fall; the interruption of the awareness of oneself and one's surroundings.
Convulsion	The sudden, violent, irregular movement of a limb/part of the body or of the entire body which is caused by involuntary contraction of muscles.
Cough	The condition of expelling air from the lungs suddenly/rapidly and with a harsh noise; often involuntarily in order to clear the lung airways of fluids/mucus/other material.
**D**	

For peer review only - http://bmjopen.bmj.com/site/about/guidelines.xhtml

Dehydration	A condition of excessive loss of water/moisture from the body.
Diarrhea	A condition of passing feces from the body very frequently and in a liquid rather than solid form.
Diphtheria	A highly contagious, sometimes fatal disease caused by the bacterium *Corynebacterium diphtheriae*. It is spread from person-to-person, through the air or contaminated objects and affects the upper respiratory tract. Common symptoms are sore throat, swollen glands/ lymph nodes, malaise, fever/chills, a membranous substance/thick gray material that forms in the throat/tonsils and makes it difficult to breathe/ rapid breathing ("barking cough") and swallow. A form of diphtheria that involves the skin, eyes, or genitals is also reported and is characterized by pain, redness, and swelling of the skin.
Dizziness/Confusion/ Unconsciousness	The state of being bewildered or unclear in mind; an abnormal state of response to stimuli in the environment; faint, wooziness, weakness, unsteadiness or a tendency to fall; the interruption of the awareness of oneself and one's surroundings.
**E**	
Edema/Swelling	The excess collection of a watery fluid/swelling in the cavities or tissues of the body; it's commonly noticed in the hands/arms/feet/legs; volume increase of tissue.
Education - formal	A systematic, organized education model that is structured and administered according to a given set of laws and norms; formal education institutions are administratively, physically and curricularly organized and require a minimum classroom attendance from students; it involves intermediate and final assessments in order to advance to the next learning stage; it confers degrees and diplomas pursuant to a quite strict set of regulations.
**F**	
Fatigue/Malaise/ Weakness	The state of feeling tired, exhausted, or weak; a general feeling of discomfort, uneasiness, unhealthiness, or unhappiness.
Fever, any	An abnormally high body temperature (usually above 38°C).
Fever <3 days	An abnormally high body temperature (usually above 38°C) that appears continuously for less than 3 days.
Fever >3 days	An abnormally high body temperature (usually above 38°C) that lasts longer than 3 days and appears continuously.
**G**	
**H**	
Headache	Any kind of pain in any region of the head.
Healthcare - actual	Healthcare that is related to specific sign/symptom-associated disease onsets or disease onset dates.
Healthcare - general	Healthcare that is unrelated to specific sign/symptom-associated disease onsets or disease onset dates.
Healthcare facility	Places or institutions where healthcare is provided; including hospitals, clinics, healthcare centers, health posts, and specialized care centers.
Haemophilus influenzae type B	A bacterium that causes a range of illnesses and is spread by droplets through coughs and sneezing, and occurs primarily among children <5years of age; common signs are fever, headache, and stiff neck up to sepsis. It can cause ear infections, cellulitis (soft tissue infection), arthritis, upper respiratory infections, pneumonia, meningitis (potential brain damage) and epiglottitis (with airway obstructions).
Heart disorders	Any disorder that affects or is caused by the heart; conditions that involve narrowed/blocked blood vessels that can lead to heart attack, chest pain, or stroke; condition that may affect one's heart muscle, valves, rhythm, or blood pressure.
Hepatitis B	An infectious disease caused by the hepatitis B virus (HBV) that affects the liver (acute/chronic liver infection; may result in liver failure, cancer, or cirrhosis). The virus is spread from person-to-person through blood, semen, or other bodily fluids. Common symptoms are fever, malaise/weakness/fatigue, loss of appetite, nausea/ vomiting, abdominal discomfort/pain, dark-colored urine, joint pain and jaundice.
Hepatitis E	An infectious disease caused by the hepatitis E virus (HEV) that affects the liver (only acute liver infection; may result in acute liver failure). The virus is spread via the fecal-oral route through the ingestion of contaminated food and water, infected

For peer review only - http://bmjopen.bmj.com/site/about/guidelines.xhtml

	animals (zoonotically) and blood. Common symptoms are fever, malaise, loss of appetite, diarrhea, nausea, abdominal discomfort, dark-colored urine, joint pain and jaundice.
HIV/AIDS	An infectious disease caused by the human immunodeficiency virus (HIV) that affects/damages the immune system and results in the acquired immunodeficiency syndrome (AIDS), a chronic, potentially life-threatening condition; it is transmitted through infectious body fluids such as blood, semen, and other bodily fluids. Common symptoms of an acute/early infection are fever, fatigue, headache, aches/joint pain, rash, sore throat, swollen glands/lymph nodes, diarrhea, weight loss, oral yeast infection and shingles. The untreated disease progresses to AIDS; symptoms caused by the severely damaged immune system are night sweats, recurring fever, chronic diarrhea, lesions on tongue and in the mouth, fatigue, weight loss, and skin rushes.
Household	A person or a group of related or unrelated persons that live together in the same dwelling unit, that acknowledge one male or female adult as the head of the household, that share the same housekeeping arrangements, that are considered to constitute one unit, and that provide themselves with food or other essentials for living. A household may be located in a single housing unit (single-story building) or in a set of collective living quarters (multi-story building).
**I**	
Income/Wage	Money or another form of payment that someone receives periodically in exchange for providing a good or service. It is usually received from a job/occupation.
Influenza	An infectious, highly contagious disease of the respiratory tract that is caused by flu viruses; it is spread from person-to-person via infected saliva droplets and contaminated objects. Common symptoms are fever, aches in muscles/arms/legs, chills/sweats, headache, fatigue/weakness, runny nose/nasal congestion, sneezing, sore throat and dry persistent cough.
Intestinal pain/ Abdominal pain	Pain (acute/short-lived or chronic/long-lived) that occurs anywhere between the chest and the groin (often referred to as the stomach region/belly).
**J**	
Jaundice	A condition of yellowing of the skin/whites of the eyes.
**K/L**	
**M**	
Malaise/Fatigue/ Weakness	The state of feeling tired, exhausted, or weak; a general feeling of discomfort, uneasiness, unhealthiness, or unhappiness.
Malaria	An infectious, sometimes fatal disease caused by a parasite (*Plasmodium* spp.) that is transmitted primarily by the bite of infected mosquitoes; infection may also spread from mother-to-child prenatally or during delivery, or through contact with contaminated blood. Common symptoms are recurrent attacks of chills, sweating, high fever, headache, vomiting and diarrhea.
Measles	An infectious, sometimes fatal disease of the respiratory tract (nose/throat) that is caused by a virus that affects mainly children <5years of age; it is spread from person-to-person via infected saliva droplets and contaminated objects. Common symptoms are fever, dry cough, runny nose, inflamed eyes, sore throat, large flat spots on the skin, and white spots in the mouth and on the cheek.
Meningitis	A viral/bacterial/fungal, sometimes life-threatening infectious inflammation of the membranes (meninges) surrounding the brain and spinal cord; it is spread from person-to-person via infected saliva droplets and contaminated objects. Common symptoms are high fever, severe headache, nausea/vomiting, confusion, seizures, loss of appetite and thirst, fatigues, sensitivity to light, skin rush and stiff neck.
Mumps	A viral infectious disease caused by the mumps virus that affects mainly the parotid glands that are situated below and in front of the ears; it is spread from person-to-person via infected saliva droplets and contaminated objects. Common symptoms are fever, swelling of one/both parotid glands, headache, muscle aches, weakness/fatigue, loss of appetite and pain while shewing/swallowing; a known complication may be hearing loss.

For peer review only - http://bmjopen.bmj.com/site/about/guidelines.xhtml

**N**	
Nausea/Vomiting	A feeling of sickness in the stomach with an inclination to vomit; a condition of ejecting part or all of the contents of the stomach, primarily through the mouth.
Neighbor	A person (related or unrelated) living next door to a household; a neighbor does not live together with members of a household (next door) in the same dwelling unit, does not share the same housekeeping arrangements, food, or other living essentials with members of a household (next door), and does not constitute one unit with members of a household (next door).
**O**	
Occupation	A job, or means of earning a wage/living; often requires the mastery of a complex set of knowledge, tasks, duties and skills acquired through formal education and/or practical experience.
**P**	
Pain/Aches	A condition of having continuous/prolonged dull pain in a part of the body; the physical feeling, suffering, or discomfort caused by an illness or injury.
Pertussis	Pertussis, also known as whooping cough, is a highly contagious bacterial disease. Symptoms similar to a common cold like runny nose, fever and cough are followed by weeks of sever coughing and a high-pitched whooping sound or gasping when a diseased person breathes in; severe coughing my even be followed by vomiting, breaking of ribs, or extreme fatigue. Infected very young and old people may have little or no cough, or they may have periods of interrupted breathing during which they do not breath are all. Pertussis is transmitted by the bacterium *Bordetella pertussis* through coughs and sneezes of an infected person.
Pharmacy	A place where medicines (prescription or nonprescription) are prepared, preserved, compounded and dispensed.
Physician	A person skilled/trained in healing; a person educated, clinically experienced, and licensed to practice medicine.
Pneumococcus	A bacterial infection caused by the bacterium *Streptococcus pneumoniae*; a sometimes fatal inflammation of the lungs that often affects immunocompromised people; it is spread from person-to-person via infected saliva droplets and contaminated objects. Common symptoms are fever, chills, cough (with phlegm/pus), chest pain, difficulty in breathing/shortness of breath, fatigue, nausea/vomiting and diarrhea; rare symptoms are sepsis, pleural effusion, and empyema.
Polio	A viral, highly contagious, sometimes fatal infectious disease caused by the poliovirus; in its most severe form causes paralysis and difficulties in breathing; it is transmitted via the fecal-oral route through ingestion of contaminated water and food, and from person-to-person contact. Common symptoms are fever, sore throat, headache, vomiting, fatigue, back pain/neck pain/stiffness, muscle weakness/aches, meningitis, loss of reflexes, and loose/floppy limbs.
**Q**	
**R**	
Rapid breathing/ Shortness of breath	The condition of abnormally fast breathing; a rate of >20 breaths per minute for adults, >30 breaths per minute for children and >45 breaths per minute for babies.
Respondent	An adult (at the country-specific legal age of majority) household member who is a decision-maker for the entire household within the study site; thus, this adult person serves as a proxy for all household members. This person may be identified by other members of the same household as the person who is primarily involved in the daily healthcare of household members.
Rotavirus	A viral infectious disease caused by rotavirus that results predominantly in diarrhea among children <5years of age; it spreads through the fecal-oral route from person-to-person contact and contaminated objects. Common symptoms are watery diarrhea, fever, vomiting, abdominal pain and dehydration.
Rubella	A contagious, viral infectious disease caused by the rubella virus; known by its distinctive red rash; it is spread from person-to-person via infected saliva droplets), from mother-to-child prenatally via blood, and contaminated objects. Common

For peer review only - http://bmjopen.bmj.com/site/about/guidelines.xhtml

	symptoms are mild fever, headache, stuffy/runny nose, inflamed red eyes, enlarged tender lymph nodes at the base of the skull/back of the neck/behind the ears, joint pain and a fine pink rash that moves from the face to the trunk, arms and legs.
Runny nose	The condition when the nose (nasal and adjacent tissues and blood vessels) produces extra mucus/excess drainage.
**S**	
Self-treatment	The medication of oneself or treatment of one's own disease or condition without medical supervision or prescription.
Severe breathing/ Difficulties in breathing	The condition of abnormal breathing that may be accompanied by wheezing, chest pain, chest in-drawing, stridor, swelling in the chest, or cough for instance.
Shivering/Chills	The sensation of coldness that could be accompanied by shivering or shaking of the body and pallor of the skin.
Shortness of breath/ Rapid breathing	The condition of abnormally fast and deep breathing; a rate of >20 breaths per minute for adults, >30 breaths per minute for children and >45 breaths per minute for babies.
Sneezing	The condition of suddenly forcing air out through nose and mouth with a usual loud noise.
Swelling/Edema	A condition of an excess collection of a watery fluid/swelling in the cavities or tissues of the body; it's commonly noticed in the hands/arms/feet/legs; volume increase of tissue.
**T**	
Tetanus	Tetanus is an infection characterized by muscle spasms which commonly begins in the jaw and progresses to the rest of the body. The spasms usually last for a few minutes only and occur frequently during 3-4 weeks of the illness but can be so severe that they may cause bone fractures or death when they affect the respiratory muscles. Tetanus may be characterized by further symptoms like fever, sweating, headache, difficulties in swallowing, high blood pressure, and an increased heart rate. The disease is caused by an infection with the bacterium *Clostridium tetani* that is found in soil, salvia, dust and manure. The bacterium enters through a skin injury (e.g. cut, puncture wound) by a contaminated object. It produces toxins that interfere with muscle contractions that result in the typical spasms.
Traditional healer	A person that aims to maintain health as well as to prevent, diagnose, and improve or treat illnesses by using a sum of knowledge, skills and practices based on theories, beliefs and experiences.
Tuberculosis	A contagious, potentially serious infectious disease caused by the bacteria *Mycobacterium tuberculosis*; primarily affects the lungs, but can also affect the kidneys and spine/brain; it is transmitted from person-to-person via infected saliva droplets. Common symptoms of active tuberculosis are cough (with or without blood), chest pain, weight loss, fatigue, fever, night sweats, chills and loss of appetite.
Typhoid fever	A bacterial, potentially life-threatening infectious disease caused by *Salmonella typhi*; it is transmitted via the fecal-oral route through contaminated food and water, or from person-to-person contact. Common symptoms are high fever, headache, abdominal pain, constipation/diarrhea, and rash. Serious complications (i.e. perforation of the gut) are reported.
**U**	
Unconsciousness/ Dizziness/Confusion	The state of being bewildered or unclear in mind; an abnormal state of response to stimuli in the environment; faint, wooziness, weakness, unsteadiness or a tendency to fall; the interruption of the awareness of oneself and one's surroundings.
**V**	
Varicella	A viral, highly contagious infectious disease, commonly known as chickenpox, that primarily affects children and is caused by the varicella-zoster virus; it is spread from person-to-person by contact with infected blisters or saliva droplets. Common symptoms are mild fever, headache, backache, loss of appetite, and vesicular skin eruptions/rash/red spots on the back and chest accompanied by severe itching.

For peer review only - http://bmjopen.bmj.com/site/about/guidelines.xhtml

Visitor	A person (related or unrelated) who comes to a household to spend some time with household members; a visitor does not live together with household members in the same dwelling unit, does not share the same housekeeping arrangements, food, or other living essentials with household members, and does not constitute one unit with household members.
Vomiting/Nausea	A feeling of sickness in the stomach with an inclination to vomit; a condition of ejecting part or all of the contents of the stomach, primarily through the mouth.
**W**	
Weakness/Malaise/ Fatigue	The state of feeling tired, exhausted, or weak; a general feeling of discomfort, uneasiness, unhealthiness, or unhappiness.
Weight loss	The (voluntary or involuntary) decrease in body weight.
Witness	A person who is an adult (at the country-specific legal age of majority), who is independent of the study, who cannot be unfairly influenced by study staff, who attends the informed consent process if the subject or subject's legally acceptable representative is illiterate, and who reads (literate) the informed consent form and any other written information supplied to the subject.
**X/Y/Z**	
Yellow fever	An acute infectious, human pathogenic, viral disease that is marked by the sudden onset of symptoms like fever, chills, loss of appetite, nausea, muscle pain, and headache, that typically resolve within a few days or are followed by more serious symptoms such as jaundice, high fever and hemorrhage. The disease is caused by a virus of the genus Flavivirus and is transmitted by the bite of an infected mosquito.

For peer review only

STROBE Statement—Checklist of items that should be included in reports of ***cross-sectional studies***

	Item No	Recommendation	
**Title and abstract**	1	(*a*) Indicate the study's design with a commonly used term in the title or the abstract	√
		(*b*) Provide in the abstract an informative and balanced summary of what was done and what was found	√
**Introduction**			
Background/rationale	2	Explain the scientific background and rationale for the investigation being reported	√
Objectives	3	State specific objectives, including any prespecified hypotheses	√
**Methods**			
Study design	4	Present key elements of study design early in the paper	√
Setting	5	Describe the setting, locations, and relevant dates, including periods of recruitment, exposure, follow-up, and data collection	√
Participants	6	(*a*) Give the eligibility criteria, and the sources and methods of selection of participants	√
Variables	7	Clearly define all outcomes, exposures, predictors, potential confounders, and effect modifiers. Give diagnostic criteria, if applicable	√
Data sources/ measurement	8*	For each variable of interest, give sources of data and details of methods of assessment (measurement). Describe comparability of assessment methods if there is more than one group	√
Bias	9	Describe any efforts to address potential sources of bias	√
Study size	10	Explain how the study size was arrived at	√
Quantitative variables	11	Explain how quantitative variables were handled in the analyses. If applicable, describe which groupings were chosen and why	√
Statistical methods	12	(*a*) Describe all statistical methods, including those used to control for confounding	√
		(*b*) Describe any methods used to examine subgroups and interactions	√
		(*c*) Explain how missing data were addressed	Not

For peer review only

			applicable
		(*d*) If applicable, describe analytical methods taking account of sampling strategy	Not applicable
		(*e*) Describe any sensitivity analyses	Not applicable
**Results**			
Participants	13*	(a) Report numbers of individuals at each stage of study—eg numbers potentially eligible, examined for eligibility, confirmed eligible, included in the study, completing follow-up, and analysed	Not applicable
		(b) Give reasons for non-participation at each stage	Not applicable
		(c) Consider use of a flow diagram	Not applicable
Descriptive data	14*	(a) Give characteristics of study participants (eg demographic, clinical, social) and information on exposures and potential confounders	Not applicable
		(b) Indicate number of participants with missing data for each variable of interest	Not applicable
Outcome data	15*	Report numbers of outcome events or summary measures	Not applicable
Main results	16	(*a*) Give unadjusted estimates and, if applicable, confounder-adjusted estimates and their precision (eg, 95% confidence interval). Make clear which confounders were adjusted for and why they were included	Not applicable
		(*b*) Report category boundaries when continuous variables were categorized	Not applicable
		(*c*) If relevant, consider translating estimates of relative risk into absolute risk for a meaningful time period	Not applicable
Other analyses	17	Report other analyses done—eg analyses of subgroups and interactions, and sensitivity analyses	Not applicable

For peer review only - http://bmjopen.bmj.com/site/about/guidelines.xhtml

**Discussion**			
Key results	18	Summarise key results with reference to study objectives	Not applicable
Limitations	19	Discuss limitations of the study, taking into account sources of potential bias or imprecision. Discuss both direction and magnitude of any potential bias	Not applicable
Interpretation	20	Give a cautious overall interpretation of results considering objectives, limitations, multiplicity of analyses, results from similar studies, and other relevant evidence	Not applicable
Generalisability	21	Discuss the generalisability (external validity) of the study results	Not applicable
**Other information**			
Funding	22	Give the source of funding and the role of the funders for the present study and, if applicable, for the original study on which the present article is based	√

For peer review only

For peer review only - http://bmjopen.bmj.com/site/about/guidelines.xhtml

Site ___ ___ ___ Subarea ___ ___ ___ House number ___ ___ ___ ___ Interviewer ID ___ ___

Appendix 2/Supplementary file 2: Study forms (English)

FORM 1 – RECORD OF HOUSEHOLD VISITS

Instructions to the interviewer

- Complete "Form 1 – RECORD OF HOUSEHOLD VISITS" for every household visited.
- Complete "Home/Respondent/Completed" once all survey parts have been completed; if not all data haven been collected at the initial and/or a consecutive visit (max. 3 visits), schedule a revisit.
- Attempt 1: complete the status of the household visit using given options; for "Not Home", "Home/Non-Respondent" and "Home/Respondent/Unavailable" schedule a revisit; for "Home/Respondent/Refusal" state the reason for refusal and visit a replacement household to the nearest on the right or left side of this household; for ~~and~~ "Non-existing/Non-residential" skip this household and visit a replacement household to the nearest on the right or left side of this household instead.
- Attempt 2: complete the status of the household visit using given options; for "Not Home", "Home/Non-Respondent" and "Home/Respondent/Unavailable" schedule a revisit.
- Attempt 3: complete the status of the household visit using given options; for "Not Home", "Home/Non-Respondent", "Home/Respondent/Unavailable" skip this household and visit a replacement household to the nearest on the right or left side of this household instead.
- "Site" and "Subarea" must be entered each as a 3-digit, "House number" as a 4-digit, and "Interviewer ID" as a 2-digit code (use lists individually prepared for each site to obtain codes); enter "Date" in the format DD/MM/YY and "Time" in the format HH:MM and circle AM or PM. "Study label" must be a combination of a 3-digit "Site" code, a 3-digit "Subarea" code and a 4-digit "House number" code; the "Interviewer ID" must be a entered as a 3-digit code.

**Attempt 1**	Date ____/____/____ (DD/MM/YY)	Time _____/_____ AM/PM	Interviewer ID ___ ___
STATUS		Tick one	
1=Not home		O	➡ Schedule revisit (Date/Time)
2=Home/Non-Respondent		O	➡ Schedule revisit (Date/Time)
3=Home/Respondent/Unavailable		O	➡ Schedule revisit (Date/Time)
4=Home/Respondent/Completed		O	
5=Home/Respondent/Refusal		O	Record the reason(s) for refusal; tick all that applies<br>O 1=No time to answer questions<br>O 2=Don't want to participate/answer questions<br>O 3=Other ____________________<br>O 98=No response O 99=Don't know<br>➡ Visit a replacement household to the nearest right or left side of this household
6=Non-existing/Non-residential		O	➡ Visit a replacement household to the nearest right or left side of this household
**Attempt 2**	Date ____/____/____ (DD/MM/YY)	Time _____/_____ AM/PM	Interviewer ID ___ ___
STATUS		Tick one	
1=Not home		O	➡ Schedule revisit (Date/Time)
2=Home/Non-Respondent		O	➡ Schedule revisit (Date/Time)
3=Home/Respondent/Unavailable		O	➡ Schedule revisit (Date/Time)

For peer review only

For peer review only - http://bmjopen.bmj.com/site/about/guidelines.xhtml

Site ___ ___ ___ Subarea ___ ___ ___ House number ___ ___ ___ ___ Interviewer ID ___ ___

4=Home/Respondent/Completed	O	
5=Home/Respondent/Refusal	O	Record the reason(s) for refusal; tick all that applies<br>O 1=No time to answer questions<br>O 2=Don't want to participate/answer questions<br><br>O 3=Other ________________<br>O 98=No response O 99=Don't know<br><br>➡ Visit a replacement household to the nearest right or left side of this household
6=Non-existing/Non-residential	O	➡ Visit a replacement household to the nearest right or left side of this household
**Attempt 3** Date ____/____/____ (DD/MM/YY)	Time _____/_____ AM/PM	Interviewer ID ___ ___
STATUS	Tick one	
1=Not home	O	➡ Visit a replacement household to the nearest right or left side of this household
2=Home/Non-Respondent	O	➡ Visit a replacement household to the nearest right or left side of this household
3=Home/Respondent/Unavailable	O	➡ Visit a replacement household to the nearest right or left side of this household
4=Home/Respondent/Completed	O	
5=Home/Respondent/Refusal	O	Record the reason(s) for refusal; tick all that applies<br>O 1=No time to answer questions<br>O 2=Don't want to participate/answer questions<br><br>O 3=Other ________________<br>O 98=No response O 99=Don't know<br><br>➡ Visit a replacement household to the nearest right or left side of this household
6=Non-existing/Non-residential	O	➡ Visit a replacement household to the nearest right or left side of this household

For peer review only

For peer review only - http://bmjopen.bmj.com/site/about/guidelines.xhtml

Site ___ ___ ___ Subarea ___ ___ ___ House number ___ ___ ___ ___ Interviewer ID ___ ___

FORM 2 – CONSENT FORM

Healthcare assessment in [add study site and -country]

Instructions to the interviewer

- Read "Form 2 – CONSENT FORM" to the respondent/ask the respondent to carefully read through "Form 2 – CONSENT FORM" if preferred.
- The respondent as defined for this investigation is an adult (at the country-specific legal age of majority) household member and decision-maker with respect to daily healthcare/healthcare utilization for the entire household and its members; it must not be e.g. a neighbour or a visitor.
- Assure that the respondent has fully understood the purpose, procedures, risks and benefits of this study prior to obtaining the respondent's consent to participate.
- The respondent must sign "Form 2 – CONSENT FORM"; if the respondent is unable to sign/illiterate, a thumbprint will serve in place of a signature and a study-independent literate adult witness (e.g. member of same household/neighbour) will sign "Form 2 – CONSENT FORM".
- Collect data about all household members regardless of whether all household members are present at the time of the interview; collecting and recording data only about individuals present at the time of the interview will result in biased data.
- Complete "Form 2 – CONSENT FORM" once for every household consented/enrolled in the study.
- Explain to the respondent that it is anticipated to revisit every household during a 2-year period; the study team will obtain a new consent for participation during each visit.
- "Site" and "Subarea" must be entered each as a 3-digit, "House number" as a 4-digit, "Interviewer ID" as a 2-digit code (use lists individually prepared for each site to obtain codes), and "Date" in the format DD/MM/YYYY. "Study label" must be a combination of a 3-digit "Site" code, a 3-digit "Subarea" code and a 4-digit "House number" code; the "Interviewer ID" must be entered as a 3-digit code.

Study principal- and co-investigators

[add names of study principal- and co-investigators and respective affiliations]

Introduction

The International Vaccine Institute (IVI) and the [add affiliation(s) of local investigators] are working together to look into health problems and healthcare seeking behaviour in your community [add study site and -country], including the socio-economic status, hygiene and sanitation behaviour, and the vaccination status among children. We would like to better understand where you, your family and your neighbours seek healthcare in case of medical conditions associated with fever/hot body and other signs & symptoms. This information will help us to find out how common fever-related and other conditions are, provide a better picture of influencing factors involved in disease transmission and infection, and to find ways to prevent and treat illnesses in your community. You, the primary decision-maker for daily healthcare/healthcare utilization for this household and its members, are invited to participate because you live in the area where we are conducting this study. This consent form will be read by you/to you and you should take your time to make your decision to accept or decline the participation in the study. Please ask members of the study team to explain any word(s) or information that you do not understand. Further information about this study, including its potential risks, benefits and inconveniences will be explained subsequently. After the study has been fully explained to you and if you agree to participate, you will be asked to sign this consent form on behalf of all household members. If you are unable to sign the form, we will ask you to provide your thumbprint, and a study-independent adult person (member of your household/neighbour) will sign the form as a witness. We will give you a signed and dated copy of the consent form.

Purpose

The purpose of this study is to look into health problems and utilization of healthcare in your community [add study site and -country], including the socio-economic status and hygiene and sanitation behaviour. We aim to better understand where you, your family and your neighbours seek healthcare for conditions associated with fever/hot body or other sings & symptoms. This information will help better estimate the burden of illnesses common in your community, and to identify ways to introduce measures to prevent and treat them.

For peer review only - http://bmjopen.bmj.com/site/about/guidelines.xhtml

Site ___ ___ ___ Subarea ___ ___ ___ House number ___ ___ ___ ___ Interviewer ID ___ ___

Procedures
We will visit about [add number for study site and -country] households in total in [add study site and -country] and will ask the same questions to each household. Your household was chosen for this study simply by chance. If you agree to participate, we will ask you on behalf of all household members a few questions about your household such as the initials, sex and age of each member. We would like to know some information related to the education and occupation of the household head, and income, possession of household items, the housing itself as well as hygiene and sanitation in the context of personal hygiene, food preparation and handling, water sources and handling, waste disposal, handling of farm and domestic animals as well as farming of this household. We will also ask where members of your household get help if anyone is sick due to fever-related and other conditions and about factors that may influence healthcare seeking behaviour of household members (i.e. travel modalities, availability of health insurance, cultural/ethnographic factors) besides the immunization status of children of this house and the perception of some diseases. Finally, we will measure the geographic location of your house. There are no right or wrong answers. You are free to choose which questions to answer. All in all, it should take about 30-45 minutes to answer all questions. The interview may take a bit longer if there are many members in this household. Also, we will visit your household two times in different seasons (once during the dry season and once towards the end of the rainy season) during the overall study period of about two years to collect data as described in this paragraph of the consent form.

Participation
You, the primary decision-maker for daily healthcare/healthcare utilization for this household and its members, and members of your household are free to choose to accept or decline to be part of the study. If you do not want to participate, that is ok. If you want to stop your participation at any time, that is ok, and there will be no harm to you or members of your household. So, participation in this study is voluntary, and you are free to refuse to participate in the study or you can withdraw your consent at any time without giving reasons and this will not involve any penalty.

Risks & Benefits
There are no direct risks for participating in this study. Asking questions about your household may make you or household members feel uncomfortable. Study teams may help providing information on established community healthcare resources for participants that express distress after completing the questions. You can refuse to answer any question at any time. You may take a break or stop participating in this study at any time. There are no direct benefits for being in this study. During visits of your household, our study teams may identify members of your household that should seek healthcare for fever-associated conditions and others. Hence, they may help providing information on established healthcare resources in your community. Indirect benefits are that all information collected will provide exact information on health problems and healthcare utilization for various conditions, including vaccination for young children, the socio-economic status as well as hygiene and sanitation behaviour in your community. This will help to provide a better picture of influencing factors involved in disease transmission and infection, and to estimate the burden of common diseases as well as to find ways to prevent and treat those in your community.

Costs & Compensation
There are no costs for participants of this study. You and anyone from your household will not be compensated for your participation.

Confidentiality
All study records and all collected information that identify you and members of your household will be kept confidential. All paper-based files will be locked safely and computerized files will be password-protected; all data files (paper-based/computerized) will be stored for a minimum of five years; these files will be accessible to authorized few study staff only. Your name or any identifier will not be used in any publication or reports from this study. Information that we collect about you and members of your household will only be shared with authorized study members such as authorized study staff and officials from ethics committees.

For peer review only - http://bmjopen.bmj.com/site/about/guidelines.xhtml

Site ___ ___ ___ Subarea ___ ___ ___ House number ___ ___ ___ ___ Interviewer ID ___ ___

Contact point for questions

If you or members of your household have any questions, please ask them now to the study team or at any time later by contacting [add name of local principal- and/or co-investigator, respective affiliations, and phone numbers]. If you have questions as a study participant about your/your household members' rights, you can contact the [add name and contact details of the local ethics committee].

Statement of consent

This consent form for participating in the assessment of health behaviour and other population-based factors in [add study site and -country] has been read by me/to me carefully. The purpose, procedures, risks & benefits have been explained to me in detail. I have been allowed to ask questions at any time, and my questions have been answered to my satisfaction by the study team. I have been told whom to contact if I have questions, want to discuss problems or concerns. I have been told that I will be given a signed and dated copy of this consent form. I have been reassured that all information obtained as result of this study will be kept confidential and used only for the purpose of this study and only by participating institutions.

I, the respondent, consent hereby voluntarily to participate in this study. I will follow the directions of the study team and give them my full cooperation. I understand that I have the right to withdraw from the study at any time.

RESPONDENT

Name: ____

(given name + middle name (if available) + family name)

Signature: ____

(handwritten depiction of person's name/mark that the person writes on a document as a proof of identity)

Date ______/______/______________ (DD/MM/YYYY)

Thumbprint

If the respondent is unable to sign/illiterate but agrees to participate, record the respondent's thumb print in the above box, and have an adult study-independent literate witness (e.g. member of the same household or a neighbor) sign below:

WITNESS

Name: ____

(given name + middle name (if available) + family name)

Signature: ____

(handwritten depiction of person's name/mark that the person writes on a document as a proof of identity)

Date ______/______/______________ (DD/MM/YYYY)

I, the interviewer, have read/explained the study to the above named respondent (witness if respondent is illiterate) in a language that the respondent understands well. I am certain that the respondent has understood the information and he/she agrees to be asked some questions.

INTERVIEWER

Name: ____

(given name + middle name (if available) + family name)

Signature: ____

(handwritten depiction of person's name/mark that the person writes on a document as a proof of identity)

Date ______/______/______________ (DD/MM/YYYY)

For peer review only - http://bmjopen.bmj.com/site/about/guidelines.xhtml

Site ___ ___ ___ Subarea ___ ___ ___ House number ___ ___ ___ ___ Interviewer ID ___ ___

FORM 3: GENERAL HOUSEHOLD INFORMATION

Instructions to the interviewer

- Complete this form only if the respondent consented to participate (see "Form 2 – CONSENT FORM"); the respondent as defined for this investigation is an adult (at the country-specific legal age of majority) household member and decision-maker with respect to daily healthcare/healthcare utilization for the entire household and its members; it must not be e.g. a neighbour or a visitor.
- Collect data about all household members regardless of whether all household members are present at the time of the interview; collecting and recording data only about individuals present at the time of the interview will result in biased data.
- A household is/household members are defined as a person or a group of related or unrelated persons that live together in the same dwelling unit, that acknowledge one male or female adult as the head of the household, that share the same housekeeping arrangements, that are considered to constitute one unit, and that provide themselves with food or other essentials for living.
- "Site" and "Subarea" must be entered each as a 3-digit, "House number" as a 4-digit, and "Interviewer ID" as a 2-digit code (use lists individually prepared for each site to obtain codes). "Study label" must be a combination of a 3-digit "Site" code, a 3-digit "Subarea" code and a 4-digit "House number" code; the "Interviewer ID" must be entered as a 3-digit code.

FORM 3: GENERAL HOUSEHOLD INFORMATION
PART A – DEMOGRAPHIC DATA/GENERAL HOUSEHOLD INFORMATION

HOUSE LOCATION

1 Site, subarea and house number

Instructions: Site (=study site) and subarea (=smallest unit of the study site) must be recorded each as a 3-digit, and house number as a 4-digit code; use individually prepared lists to obtain the respective codes.

Site: ____ ____ ____ Subarea: ____ ____ ____ House number: ____ ____ ____ ____

2 GPS coordinates

Instructions: Take GPS coordinates at the approx. center of the house. Record altitude as a 4-digit number (unit: meter), and latitude/longitude as a 7-digit number (unit: degrees, decimal minutes).

O 1=Original household O 2=Replacement household (right/left side of original household)

Altitude: ___ ___ ___ ___

Latitude: ___ ___°___ ___.___ ___ ___' Longitude: ___ ___°___ ___.___ ___ ___'

RESPONDENT

3 Provide the full name of the respondent.

Instructions: Record given name, middle name (if available) and family name of the respondent.

____

4 Provide the age of the respondent.

Instruction: Recorded as a 3-digit number (unit: year). ____ ____ ____

5 Provide the sex of the respondent.

O 1=Male O 2=Female

6 What is the respondent's relationship to this household?

O 1=Household head
O 2=Son/daughter of household head
O 3=Brother/sister of household head
O 4=Mother/father of household head
O 5=Granddaughter/grandson of household head
O 6=Other, specify __________
O 7=Spouse of household head
O 8=Spouse of son/daughter of household head
O 9=Spouse of brother/sister of household head
O 10=Mother/father in law of household head
O 11=Niece/nephew of household head
O 98=No response

For peer review only - http://bmjopen.bmj.com/site/about/guidelines.xhtml

Site ___ ___ ___ Subarea ___ ___ ___ House number ___ ___ ___ ___ Interviewer ID ___ ___

HOUSEHOLD MEMBERS

7 How many household members are living in this house in total (at the day of visit)**?**

Instruction: Recorded as a 2-digit number.

Total number of household members: ____ ____

8 Record age and sex of each household member.

Instructions: Record the respondent's initials, age and sex at the first position/row of the table. Initials, age and sex of all other household members must be recorded subsequently. The initials of a household member consist of the first three letters of the given name and the first three letters of the family name (a potential middle name is not part of the initials). Record the age of each household member as a 3-digit number (unit: year); if a household member's age is ≤12 months, record the age as 1 year. Please complete an additional form if a household has >20 members.

**Household member identifier**	**Initials** [given name/family name]	**Age** [year]	**Sex**
1/Respondent	____ ____ ____/____ ____ ____	____ ____ ____	O1=Male O2=Female
2	____ ____ ____/____ ____ ____	____ ____ ____	O1=Male O2=Female
3	____ ____ ____/____ ____ ____	____ ____ ____	O1=Male O2=Female
4	____ ____ ____/____ ____ ____	____ ____ ____	O1=Male O2=Female
5	____ ____ ____/____ ____ ____	____ ____ ____	O1=Male O2=Female
6	____ ____ ____/____ ____ ____	____ ____ ____	O1=Male O2=Female
7	____ ____ ____/____ ____ ____	____ ____ ____	O1=Male O2=Female
8	____ ____ ____/____ ____ ____	____ ____ ____	O1=Male O2=Female
9	____ ____ ____/____ ____ ____	____ ____ ____	O1=Male O2=Female
10	____ ____ ____/____ ____ ____	____ ____ ____	O1=Male O2=Female
11	____ ____ ____/____ ____ ____	____ ____ ____	O1=Male O2=Female
12	____ ____ ____/____ ____ ____	____ ____ ____	O1=Male O2=Female
13	____ ____ ____/____ ____ ____	____ ____ ____	O1=Male O2=Female
14	____ ____ ____/____ ____ ____	____ ____ ____	O1=Male O2=Female
15	____ ____ ____/____ ____ ____	____ ____ ____	O1=Male O2=Female
16	____ ____ ____/____ ____ ____	____ ____ ____	O1=Male O2=Female
17	____ ____ ____/____ ____ ____	____ ____ ____	O1=Male O2=Female
18	____ ____ ____/____ ____ ____	____ ____ ____	O1=Male O2=Female
19	____ ____ ____/____ ____ ____	____ ____ ____	O1=Male O2=Female
20	____ ____ ____/____ ____ ____	____ ____ ____	O1=Male O2=Female

For peer review only

For peer review only - http://bmjopen.bmj.com/site/about/guidelines.xhtml

Site ___ ___ ___ Subarea ___ ___ ___ House number ___ ___ ___ ___ Interviewer ID ___ ___

FORM 3: GENERAL HOUSEHOLD INFORMATION
PART B – SOCIO-ECONOMIC DATA

EDUCATION

1 Can the head of household read in [add the official language of the study site/country]**?**

O 1=Yes O 2=No O 99=Don't know O 98=No response

2 Can the head of household write in [add the official language of the study site/country]**?**

O 1=Yes O 2=No O 99=Don't know O 98=No response

3 What is the highest completed level of formal school education of the head of household?

O 1=Primary school O 4=Higher than secondary school O 99=Don't know
O 2=Middle school O 5=NO EDUCATION O 98=No response
O 3=Secondary school O 6=Other, specify ___________

For peer review only

OCCUPATION/INCOME

4 What is the main occupation of the household head?

Instructions: Record the status of the day of visit only.

O 1=Group 1: Managers
(chief executives, senior officials, legislators; administrative and commercial managers; production and specialized services managers; hospitality, retail and other services managers)

O 2=Group 2: Professional
(science and engineering professionals; health professionals; teaching professionals; business and administration professionals; information and communications technology professionals; legal, social and cultural professionals)

O 3=Group 3: Technicians and Associate Professionals
(science and engineering associate professionals; health associate professionals; business and administration associate professionals; legal, social, cultural and related associate professionals; information and communications technicians)

O 4=Group 4: Clerical Support Workers
(general and keyboard clerks; customer services clerks; numerical and material recording clerks; other clerical support workers)

O 5=Group 5: Services and Sales Workers
(personal services workers; sales workers; personal care workers; protective services workers)

O 6=Group 6: Skilled Agricultural, Forestry, and Fishery Workers
(market-oriented skilled agricultural workers; animal producers; marked-oriented skilled forestry, fishery and hunting workers; subsistence farmers, fishers, hunters and gatherers)

O 7=Group 7: Craft and Related Trades Workers
(building and related trades workers (excluding electricians); metal, machinery and related trades workers; handicraft and printing workers; electrical and electronics trades workers; food processing, woodworking, garment and other craft and related trades workers)

O 8=Group 8: Plant and Machine Operators, and Assemblers
(stationary plant and machine operators; assemblers; drivers and mobile plant operators)

O 9=Group 9: Elementary Occupations
(cleaners and helpers; agricultural, forestry and fishery labourers; labourers in mining, construction, manufacturing and transport; food preparation assistants; street and related sales and services workers; refuse workers and other elementary workers)

O 10=Group 0: Armed Forces Occupations
(commissioned armed forces officers; non-commissioned armed forces officers; armed forces occupations, other ranks)

O 11=Other, specify __________

O 12=NO OCCUPATION O 99=Don't' know O 98=No response

For peer review only - http://bmjopen.bmj.com/site/about/guidelines.xhtml

Site ___ ___ ___ Subarea ___ ___ ___ House number ___ ___ ___ ___ Interviewer ID ___ ___

5 What is the average monthly income of the household head based on the occupation stated in question 4 and of other household members?

Instruction: Tick one answer per household head/enter one code per household member if applicable/if income available.

Head of household	Member 1	Member 2	Member 3
Code ____________	Code ____________	Code ____________	Code ____________

[1] <100USD [3] ≥150 to <200USD [5] ≥250USD [99] Don't know
[2] ≥100 to <150USD [4] ≥200 to <250USD [6] Head of household NO INCOME (see Q4) [98] No response

6 Which and how much other/additional source(s) of monthly monetary earnings has this household?

Instruction: Tick all that applies; more than one answer is possible.

Other/additional sources	Overall monthly monetary earnings
O 1=Social grants<br>O 2=Insurance (i.e. private, public)<br>O 3=Loan (i.e. from friends, family, bank)<br>O 4=Selling (i.e. livestock, crops, furniture, other goods)<br>O 5=Personal savings<br>O 6=Other, specify ________________	O 1=<100 USD<br>O 2=≥100 to <150 USD<br>O 3=≥150 to <200 USD<br>O 4=≥200 to <250 USD<br>O 5=≥250 USD
O 7=NO OTHER/ADDITIONAL SOURCES<br>O 99=Don't know<br>O 98=No response	

HOUSING/DWELLING

7 Is the house owned by the members of this household?

O 1=Yes O 3=No, supplied (i.e. government, employer) O 99=Don't know
O 2=No, rented O 4=No, other, specify _________ O 98=No response

8 How many room(s)/individual house structure(s) of this household are used for sleeping, living, cooking and bathing?

Instruction: Enter one code per category only.

Bedroom	Living room	Kitchen	Bathroom
Code ____________	Code ____________	Code ____________	Code ____________

[1] 1-2 [3] >4 [99] Don't know
[2] 3-4 [4] None [98] No response

9 What is the construction material of the floor, the walls and the roof of room(s)/individual house structure(s) of this house?

Instruction: Enter all codes that applies per category; more than one answer per category is possible.

Floor	Walls	Roof
Code ____________	Code ____________	Code ____________

[1] Brick [3] Tiles [5] Wood [7] Metal/Tin [99] Don't know
[2] Cement/Concrete [4] Mud [6] Straw/Leaves [8] Other, specify _________ [98] No response

10 Do members of this household have access to/utilize electricity in this household?

O 1=Yes O 2=No O 99=Don't know O 98=No response

11 Which main source of light have members of this household access to/are utilizing?

O 1=Electric light/Light bulb(s) O 4=Battery-powered lamp(s) O 7=Other, specify _________
O 2=Solar light/Solar lamp(s) O 5=Candle(s) O 99=Don't know
O 3=Paraffin lamp(s)/Oil lamp(s) O 6=NO SOURCE OF LIGHT O 98=No response

For peer review only - http://bmjopen.bmj.com/site/about/guidelines.xhtml

Site ___ ___ ___ Subarea ___ ___ ___ House number ___ ___ ___ ___ Interviewer ID ___ ___

HOUSEHOLD ASSETS

12 Which of the following items do members of this household own?
Instructions: Tick all that applies; more than one answer is possible; record the quantity of functional items where applicable.

O 1=Radio	Quantity ____	O None	O 11=Tape/CD player	Quantity ____	O None
O 2=Television	Quantity ____	O None	O 12=Computer/Laptop	Quantity ____	O None
O 3=Internet connection	Quantity ____	O None	O 13=Mobile/Cell phone	Quantity ____	O None
O 4=Car	Quantity ____	O None	O 14=Truck/Bus	Quantity ____	O None
O 5=Motorbike	Quantity ____	O None	O 15=Bicycle	Quantity ____	O None
O 6=Oxcart/Donkey	Quantity ____	O None	O 16=Bed	Quantity ____	O None
O 7=Mattress	Quantity ____	O None	O 17=Bed net	Quantity ____	O None
O 8=Table	Quantity ____	O None	O 18=Chair	Quantity ____	O None
O 9=Refrigerator (4°C)	Quantity ____	O None	O 19=Freezer (-20°C)	Quantity ____	O None
O 10=Fan	Quantity ____	O None	O 20=Sewing machine	Quantity ____	O None

For peer review only

For peer review only - http://bmjopen.bmj.com/site/about/guidelines.xhtml

Site ___ ___ ___ Subarea ___ ___ ___ House number ___ ___ ___ ___ Interviewer ID ___ ___

FORM 3: GENERAL HOUSEHOLD INFORMATION
Part C – Hygiene and sanitation

TOILET FACILITY

1 To what type of toilet facility have members of this household usually access?

Instruction: Inside means inside a room/individual house structure/building; outside means outside a room/individual house structure/building.

- O 1=Flush toilet/Water closet (inside)
- O 2=Flush toilet/Water closet (outside)
- O 3=Non-flush latrine/pit latrine (inside)
- O 4=Non-flush latrine/pit latrine (outside)
- O 5=Ventilated Improved Pit Latrine (KVIP, inside)
- O 6=Ventilated Improved Pit Latrine (KVIP, outside)
- O 7=Composting toilet (inside)
- O 8=Composting toilet (outside)
- O 9=NO toilet facility, use a bucket
- O 10=NO toilet facility, dig a hole
- O 11=NO toilet facility, free range/open defecation
- O 12=Other, specify __________
- O 99=Don't know
- O 98=No response

2 Do members of this household usually utilize the toilet facility stated in question 1?

- O 1=No, members cannot utilize the facility independently (i.e. infants, toddlers)
- O 2=No, members cannot utilize the facility independently (i.e. disabled, long-term/chronically diseased)
- O 3=No, members cannot utilize the facility independently (i.e. seniors)
- O 4=No, limited access (i.e. public, shared with other households/community)
- O 5=No, too far away (i.e. public, shared with other households/community)
- O 6=No, bad condition (i.e. broken, not clean)
- O 7=YES
- O 8=Other, specify _________________
- O 99=Don't know
- O 98=No response

3 Where is the toilet facility stated in question 1 located? With how many other people is it usually shared?

- O 1=On the property of this household (private, not shared)
- O 2=On the property of a neighbouring household (private, shared)
  - *Shared* O 1=<10 people O 2=11-24 people O 3=25-49 people O 4=>50 people O 99=Don't know O 98=No response
- O 3=In the community (public, shared)
  - *Shared* O 1=<10 people O 2=11-24 people O 3=25-49 people O 4=>50 people O 99=Don't know O 98=No response
- O 4=Other, specify __________ O 99=Don't know O 98=No response

4 How do members of this household that have/don't have access to a toilet facility usually clean their hands directly before and after urination/defection?

Instruction: Enter one code per category only.

Clean hands before urination/defection	Clean hands after urination/defection
Code _____________	Code _____________
[1] Wash hands with soap and water<br>[2] Wash hands with water only<br>[3] Rub hands clean with some leaves/straw/ grass<br>[4] Rub hands clean with some sand<br>[5] Rub hands clean with some cloth/fabric	[6] Don't clean, not needed/not dirty<br>[7] Don't clean, nothing to clean hands nearby/on hands<br>[8] Other, specify __________<br>[99] Don't know<br>[98] No response

KITCHEN/FOOD HANDLING

5 Do members of this household have a kitchen for cooking/preparing food/meals? Where is it located?

- O 1=Yes, inside kitchen/in a room/individual house structure; on the property of this household (private, not shared)
- O 2=Yes, outside kitchen/outside a room/individual house structure/ open air; on the property of this

For peer review only - http://bmjopen.bmj.com/site/about/guidelines.xhtml

Site ___ ___ ___ Subarea ___ ___ ___ House number ___ ___ ___ ___ Interviewer ID ___ ___

household (private, not shared)
O 3=Yes, inside kitchen/in a room/individual house structure; on the property of a neighbouring household (private, shared)
O 4=Yes, outside kitchen/outside a room/individual house structure/ open air; on the property of a neighbouring household (private, shared)
O 5=NO KITCHEN
O 6=Other, specify __________ O 99=Don't know O98=No response

6 What energy source do members of this household usually use for cooking/preparing food/meals?
O 1=Electricity O 3=Charcoal O 5=Animal dung O 99=Don't know
O 2=Gas/Kerosene O 4=Wood/straw/leaves O 6=Other, specify _____ O 98=No response

7 What cooking spot do members of this household usually use for cooking/preparing food/meals?
O 1=Open fire O 3=Closed stove with chimney O 99=Don't know
O 2=Open stove O 4=Other, specify __________ O 98=No response

8 Where do members of this household usually store fresh/raw food items (e.g. vegetables, lettuce, fruits, dairy products, eggs, meat and fish) and left-over/prepared food/meals?
Instruction: Enter one code per category only. Inside means inside a room/individual house structure/building; outside means outside a room/individual house structure/building.

Fresh/raw food items (i.e. vegetables, lettuce, fruits, dairy products, eggs, meat and fish)	Left-over/prepared food/meals
Code _____________	Code _____________

[1] At room temperature (inside, on the property of this household, private)
[2] At room temperature (outside, on the property of this household, private)
[3] At room temperature (inside, on the property of a neighboring household, private, shared)
[4] At room temperature (outside, on the property of a neighboring household, private, shared)
[5] Refrigerator (on the property of this household, private)
[6] Refrigerator (on the property of a neighboring household, private, shared)
[7] Freezer (on the property of this household, private)
[8] Freezer (on the property of a neighboring household, private, shared)
[9] Other, specify ______________
[10] NO STORAGE [99] Don't know [98] No response

9 If the answer is "Room temperature" for fresh/raw food items and/or left-over/ prepared food/meals in question 8, how long do members of this household usually store them?
Instruction: Enter one code per category only.

Fresh/raw food items (i.e. vegetables, lettuce, fruits, dairy products, eggs, meat and fish)	Left-over/prepared food/meals
Code _____________	Code _____________

[1] <1 day [3] 3-4 days [5] Not applicable [99] Don't know
[2] 1-2 days [4] >4 days [98] No response

10 Do members of this household usually cover left-over/prepared food/meals? Do they re-heat/re-cook left-over/prepared food/meals prior to consumption?
Instruction: Enter one code per category only.

Cover left-over/prepared food/meals	Re-heat/Re-cook left-over/prepared food/meals
Code _____________	Code _____________

[1] Yes [2] No [99] Don't know [98] No response

For peer review only - http://bmjopen.bmj.com/site/about/guidelines.xhtml

Site ___ ___ ___ Subarea ___ ___ ___ House number ___ ___ ___ ___ Interviewer ID ___ ___

11 Which member(s) of this household usually cook(s)/prepare(s) food/meals for other members of this household?

Instructions: Refer to question 9 of "Form 3 Part A – Demographic data/General household information" and record the initials/identifier(s) of the member.

O Household member, record initials/identifier(s) __________ O 99=Don't know O 98=No response

12 How does/do the household member(s) that usually cook(s)/prepare(s) food/meals for other members of this household as stated in question 11 usually prepare(s) herself/himself/themselves before and after the preparation of meals?

Instruction: Enter one code per category only.

Before the preparation of meals	After the preparation of meals
Code _____________	Code _____________
[1] Wash hands with soap and water<br>[2] Wash hands with water only<br>[3] Rub hands clean with some leaves/straw/ grass<br>[4] Rub hands clean with some sand<br>[5] Rub hands clean with some cloth/fabric	[6] Don't clean, not needed/not dirty<br>[7] Don't clean, nothing to clean hands nearby/on hands<br>[8] Other, specify __________<br>[99] Don't know<br>[98] No response

13 How do members of this household usually prepare themselves before and after eating food/meals, including the household member that usually cooks/prepares food/meals as stated in question 11?

Instruction: Enter one code per category only.

Before the eating of food/meals	After the eating of food/meals
Code _____________	Code _____________
[1] Wash hands with soap and water<br>[2] Wash hands with water only<br>[3] Rub hands clean with some leaves/straw/ grass<br>[4] Rub hands clean with some sand<br>[5] Rub hands clean with some cloth/fabric	[6] Don't clean, not needed/not dirty<br>[7] Don't clean, nothing to clean hands nearby/on hands<br>[8] Other, specify __________<br>[99] Don't know<br>[98] No response

14 How do members of this household usually eat their food/meals, including the household member that usually cooks/prepares food/meals as stated in question 11?

O 1=With cutlery O 3=Other, specify _____________ O 99=Don't know
O 2=With fingers O 98=No response

15 Do members of this household usually eat their food/meals from the same pot, including the household member that usually cooks/prepares food/meals as stated in question 11?

O 1=Yes O 2=No O 99=Don't know O 98=No response

16 Where do members of this household usually get their main fresh/raw food items such as vegetables, fruits and grains/cereals, dairy products, eggs, meat and fish?

Instruction: Enter one code per category only.

Vegetables, fruits, grains/ cereals	Dairy products	Eggs, meat, fish
Code _____________	Code _____________	Code _____________

[1] Self-farming (i.e. back-/courtyard)
[2] Supermarket/Grocery store
[3] Friend/Family
[4] Open market
[5] Street vendor
[6] Self-farming (i.e. agricultural land)
[7] Butcher
[8] Other, specify _____________
[99] Don't know
[98] No response

17 Do members of this household usually consume/eat fresh/raw food items (e.g. vegetables, lettuce, fruits, dairy products, ice cream, eggs, meat and fish)? If so, which ones?

For peer review only - http://bmjopen.bmj.com/site/about/guidelines.xhtml

Site ___ ___ ___ Subarea ___ ___ ___ House number ___ ___ ___ ___ Interviewer ID ___ ___

Instruction: Tick all that applies; more than one answer is possible.

- O 1=Vegetables/Lettuce (cultivated on bush)
- O 2=Vegetables/Lettuce (wild harvesting from bush)
- O 3=Fruits (cultivated on bush/tree)
- O 4=Fruits (wild harvesting from bush/tree)
- O 5=Dairy products (i.e. milk, cheese, yoghurt from domesticated milk producing animals), specify ____________
- O 6=Eggs (from domesticated poultry), specify _________
- O 7=Animal blood (from domesticated animals), specify _________
- O 8=Beef/Zebu/Buffalo (domesticated), specify _________
- O 9=Pork (domesticated)
- O 10=Sheep (domesticated)
- O 11=Meat from non-domesticated animals (i.e. antelope, boar, poultry, rodents, fish), specify _________
- O 12=NO CONSUMPTION of fresh/raw food items
- O 13=Vegetables/Lettuce (cultivated on ground)
- O 14=Vegetables/Lettuce (wild harvesting from ground)
- O 15=Fruits (cultivated on ground)
- O 16=Fruits (wild harvesting from ground)
- O 17=Dairy products (i.e. milk, cheese, yoghurt from non-domesticated milk producing animals), specify ____________
- O 18=Eggs (from non-domesticated poultry), specify _________
- O 19=Animal blood (from non-domesticated animals), specify _________
- O 20=Poultry (domesticated; i.e. chicken, duck, pigeon, goose, turkey), specify _________
- O 21=Goat (domesticated)
- O 22=Rodents (domesticated), specify _________
- O 23=Fish/Seafood, specify _________
- O 24=Ice cream
- O 25=Other, specify _________
- O 99=Don't know O 98=No response

18 Do members of this household consume meals/food items/beverages usually also outside the house? If so, which member(s) of this household and where?

Instructions: Refer to question 9 of "Form 3 Part A – Demographic data/General household information" and record the initials/identifier of the member(s). Enter all codes that apply for the respective household member.

Member 1<br>Initials/Identifier: _____	Member 2<br>Initials/Identifier: _____	Member 3<br>Initials/Identifier: _____	Member 4<br>Initials/Identifier: _____
Code ____________	Code ____________	Code ____________	Code ____________
[1] Restaurant<br>[2] Fast food chain	[3] Cafeteria<br>[4] Street vendor	[5] Other, specify ____<br>[6] Not applicable	[99] Don't know<br>[98] No response

19 Which meals/food items/beverages do the member(s) of this household listed in question 18 usually consume outside the house?

Instructions: Refer to question 9 of "Form 3 Part A – Demographic data/General household information" and record the initials/identifier of the member(s). List up to 5 meals/food items/beverages per household member. You can refer to food items listed in question 17. Skip this question of no household member consumes meals/food items/beverages outside the house as stated in Q18.

Member 1<br>Initials/Identifier: _____	Member 2<br>Initials/Identifier: _____	Member 3<br>Initials/Identifier: _____	Member 4<br>Initials/Identifier: _____
1: ______________<br>2: ______________<br>3: ______________<br>4: ______________<br>5: ______________	1: ______________<br>2: ______________<br>3: ______________<br>4: ______________<br>5: ______________	1: ______________<br>2: ______________<br>3: ______________<br>4: ______________<br>5: ______________	1: ______________<br>2: ______________<br>3: ______________<br>4: ______________<br>5: ______________

WATER

20 What is the main water supply for members of this household for drinking, cooking/ preparing food/meals, bathing, and cleaning (of house/individual house structure(s), clothing/kitchen tools)?

Instruction: Enter one code per category only.

Drinking	Cooking	Bathing	Cleaning
Code ____________	Code ____________	Code ____________	Code ____________
[1] Tap water (inside the house; private)<br>[2] Tap water (outside the house i.e. back-/courtyard; private)			

For peer review only - http://bmjopen.bmj.com/site/about/guidelines.xhtml

Site ___ ___ ___ Subarea ___ ___ ___ House number ___ ___ ___ ___ Interviewer ID ___ ___

[3] Tap water (outside the house; public/shared)
[4] Standpipe/Piped water (inside the house; private)
[5] Standpipe/Piped water (outside the house i.e. back-/ courtyard; private)
[6] Standpipe/Piped water (outside the house; public/shared)
[7] Water truck (public; shared)
[8] Surface water (i.e. canal, irrigation canal)
[9] Surface water (i.e. lake, pond, river, stream)
[10] Spring water
[11] Rain water
[12] Well/Borehole (open/uncovered/unprotected)
[13] Well/Borehole (covered/protected)
[14] Bottled water
[15] Other, specify ________________ [99] Don't know [98] No response

21 Do members of this household usually treat water for drinking, cooking/ preparing food/meals, bathing, and cleaning (of house/individual house structure(s), clothing/ kitchen tools) prior to consumption? How is water usually treated?

Instruction: Enter all codes that applies per category; more than one answer per category is possible.

Drinking	Cooking	Bathing	Cleaning
Code ____________	Code ____________	Code ____________	Code ____________

[1] No, not at all
[2] No, only for sick members of this household
[3] No, only depending on the season (dry season)
[4] Yes, filtration (i.e. cloth, ceramic, sand)
[5] Yes, sedimentation
[6] Other, specify ________________
[7] No, only for children of this household
[8] No, only depending on the season (rainy season)
[9] Yes, boiling
[10] Yes, solar disinfection
[11] Yes, chlorination
[99] Don't know [98] No response

22 Which member(s) of this household usually collect(s) the drinking water as stated in question 20 for other members of this household?

Instructions: Refer to question 9 of "Form 3 Part A – Demographic data/General household information" and record the initials/identifier of the member.

O Household member, record initials/identifier(s) __________ O 99=Don't know O 98=No response

23 How do members of this household usually store the drinking water stated in question 20?

O 1=Water tank (uncovered)
O 2=Water tank (covered with i.e. cloth/fabric, lid, leaves)
O 3=Container (uncovered)
O 4=Container (covered with i.e. cloth/fabric, lid, leaves)
O 5=Clay pot (uncovered)
O 6=Clay pot (covered with i.e. cloth/fabric, lid, leaves)
O 7=Bucket (uncovered)
O 8=Bucket (covered with i.e. cloth/fabric, lid, leaves)
O 9=Bowl (uncovered)
O 10=Bowl (covered with i.e. cloth/fabric, lid, leaves)
O 11=Wooden pot (uncovered)
O 12=Wooden pot (covered with i.e. cloth/fabric, lid, leaves)
O 13=Skin vessel (i.e. goat, sheep, cattle; uncovered)
O 14=Skin vessel (i.e. goat, sheep, cattle; covered with i.e. cloth/fabric, lid, leaves)
O 15=NOT STORED
O 16=Other, specify __________
O 99=Don't know
O 98=No response

24 If members of this household use a shared drinking water supply as stated in question 20, with how many other people is this drinking water supply usually shared?

O 1=<10 people
O 2=11-24 people
O 3=25-49 people
O 4=>50 people
O 5=NOT SHARED
O 99=Don't know
O 98=No response

For peer review only - http://bmjopen.bmj.com/site/about/guidelines.xhtml

Site ___ ___ ___ Subarea ___ ___ ___ House number ___ ___ ___ ___ Interviewer ID ___ ___

WASTE DISPOSAL

25 Where do members of this household usually dispose waste water from e.g. cooking/ preparation of food/meals, bathing and/or cleaning, dispose domestic waste, human waste/excreta?

Instruction: Enter one code per category only.

Waste water from (e.g. cooking/ preparation of food/ meals, bathing and/or cleaning)	Domestic waste	Human waste/excreta
Code ____________	Code ____________	Code ____________

[1] On the ground/free range near the house (i.e. back-/courtyard, private)
[2] On the ground/free range away from the house in the community (shared, public)
[3] In a hole near the house (i.e. back-/courtyard, private)
[4] In a hole away from the house in the community (shared, public)
[5] Pour in surface water in the community (i.e. lake, pond, river, stream, public, shared)
[6] Pour in surface water in the community (i.e. canal, irrigation canal, public, shared)
[7] Pour in spring water in the community (public, shared)
[8] Pour in a septic tank near the house (i.e. back-/courtyard, near toilet facility if available, private)
[9] Pour in a septic tank in the community (shared, public)
[10] Pour in the sewer near the house (i.e. back-/courtyard, private)
[11] Pour in the sewer in the community (shared, public)
[12] Other, specify ________________________ [99] Don't know [98] No response

26 Do members of this household usually pre-process domestic waste from this house prior to disposing it?

O 1=Yes, burning garbage
O 2=Yes, separate/sort out degradable from non-degradable garbage
O 3=NO
O 4=Yes, other, specify _________
O 99=Don't know O 98=No response

27 How far from the main drinking water supply of this household as stated in question 20 is the location for disposal of waste water, domestic waste and human waste/excreta of this household as stated in question 25?

Instructions: Enter one code per category only. Record the distance as meter [m].

Waste water from (e.g. cooking/ preparation of food/ meals, bathing and/or cleaning)	Domestic waste	Human waste/excreta
Code ____________	Code ____________	Code ____________

[1] <5 m [3] ≥25 to <50 m [5] ≥100 to <500 m [7] ≥1,000 m
[2] ≥5 to <25 m [4] ≥50 to <100 m [6] ≥500 to <1,000 m [99] Don't know [98] No response

28 How far from the main water supply for cooking/preparing food/meals of this household as stated in question 20 is the location for disposal of waste water, domestic waste and human waste/excreta of this household as stated in question 25?

Instructions: Enter one code per category only. Record the distance as meter [m].

Waste water from (e.g. cooking/preparation of food/ meals, bathing and/or cleaning)	Domestic waste	Human waste/excreta
Code ____________	Code ____________	Code ____________

[1] <5 m [3] ≥25 to <50 m [5] ≥100 to <500 m [7] ≥1,000 m
[2] ≥5 to <25 m [4] ≥50 to <100 m [6] ≥500 to <1,000 m [99] Don't know [98] No response

FARM/DOMESTIC ANIMALS

29 What kind of farm animals/livestock and domestic animals/pets do members of this household have?

Instruction: Tick all that applies per category; more than one answer per category is possible.

For peer review only - http://bmjopen.bmj.com/site/about/guidelines.xhtml

Site ___ ___ ___ Subarea ___ ___ ___ House number ___ ___ ___ ___ Interviewer ID ___ ___

Farm animals/livestock		Domestic animals/pets
O 1=Cattle	O 9=Camel	O 1=Dog
O 2=Sheep	O 10= Rodents (i.e. rabbit), specify ____________________	O 2=Rodents (i.e. rabbit), specify ____________________
O 3=Donkey	O 11=Goat	O 3=Cat
O 4=Poultry (i.e. chicken, duck, pigeon, goose, turkey), specify ______________	O 12=Horse	O 4=NO DOMESTIC ANIMALS/ PETS
O 5=Zebu	O 13=NO FARM ANIMALS/ LIVESTOCK	O 5=Other, specify ____________________
O 6=Pig	O 14=Other, specify ______________	O 99=Don't know
O 7=Fish	O 99=Don't know	O 98=No response
O 8=Buffalo	O 98=No response	

30 Where do members of this household usually keep farm animals/livestock and domestic animals/pets of this household as stated in question 29?
Instruction: Tick one answer per category only.

Farm animals/livestock	Domestic animals/pets
O 1=Stable/Cage/Water basin on the property of the house (i.e. back-/courtyard)<br>O 2=Stable/Cage/Water basin outside the house in the community<br>O 3=Stable/Cage/Water basin outside the community<br>O 4=Free range/Surface water/Spring water on the property of the house (i.e. house, back-/courtyard)<br>O 5=Free range/Surface water/Spring water outside the house in the community<br>O 6=Free range/Surface water/Spring water outside the community<br>O 7=Not applicable (nowhere/no farm animals/livestock)<br>O 8=Other, specify __________<br>O 99=Don't know<br>O 98=No response	O 1=Cage/Chain near the house (i.e. back-/courtyard)<br>O 2=Cage/Chain outside the house in the community<br>O 3=Cage/Chain outside the community (public)<br>O 4=Free range near the house (i.e. in the house, back-/courtyard)<br>O 5=Free range outside the house in the community<br>O 6=Free range outside the community<br>O 7=Not applicable (nowhere/no domestic animals/pets)<br>O 8=Other, specify __________<br>O 99=Don't know<br>O 98=No response

For peer review only

31 What is the extent of the livestock farming that members of this household practice?

O 1=Own demand ("backyard farming")
O 2=Not applicable (no livestock farming)
O 3=Mass/Industrial demand ("mass animal farming")
O 4=Other, specify __________
O 99=Don't know
O 98=No response

32 Which water supply do members of this household usually use to water farm animals/livestock and domestic animals/pets of this household as stated in question 29?
Instruction: Enter one code per category only.

Farm animals/livestock	Domestic animals/pets
Code ____________	Code ____________

[1] Tap water (inside the house; private)
[2] Tap water (outside the house i.e. back-/courtyard; private)
[3] Tap water (outside the house; public/shared)
[4] Standpipe/Piped water (inside the house; private)
[5] Standpipe/Piped water (outside the house i.e. back-/ courtyard; private)
[6] Standpipe/Piped water (outside the house; public/shared)

For peer review only - http://bmjopen.bmj.com/site/about/guidelines.xhtml

Site ___ ___ ___ Subarea ___ ___ ___ House number ___ ___ ___ ___ Interviewer ID ___ ___

[7] Water truck (public; shared)
[8] Surface water (i.e. canal, irrigation canal)
[9] Surface water (i.e. lake, pond, river, stream)
[10] Spring water
[11] Rain water
[12] Well/Borehole (open/uncovered/unprotected)
[13] Well/Borehole (covered/protected)
[14] Not applicable (no farm animals/livestock; no domestic animals/pets)
[15] Other, specify ___________________ [99] Don't know [98] No response

33 Do members of this household usually wash their hands after they fed & watered and cleaned the stable/cage/water basin of the farm animals/livestock and domestic animals/pets of this household as stated in question 29?

Instruction: Enter one code per category only.

Farm animals/livestock	Domestic animals/pets
Code ____________	Code ____________
[1] Wash hands with soap and water	[7] Don't clean, nothing to clean hands nearby/on hands
[2] Wash hands with water only	[8] Other, specify _________
[3] Rub hands clean with some leaves/straw/ grass	[9] Not applicable (no farm animals/livestock; no domestic animals/pets)
[4] Rub hands clean with some sand	
[5] Rub hands clean with some cloth/fabric	[99] Don't know
[6] Don't clean, not needed/not dirty	[98] No response

CROP FARMING

34 What kind of crops do members of this household grow/cultivate?

Instruction: Tick all that applies; more than one answer is possible.

- O 1=Coffee
- O 2=Rice
- O 3=Fruits (cultivated on bush/tree)
- O 4=Fruits (cultivated on the ground)
- O 5=Vegetables/Lettuce (cultivated on bush/tree)
- O 6=Vegetables/Lettuce (cultivated on the ground)
- O 7=Grains (i.e. wheat, barley, oat, corn)
- O 8=Tea
- O 9=Cashew
- O 10=Tobacco
- O 11=Sugar cane
- O 12=Chad/Chat
- O 13=Bees/Honey
- O 14=Cotton
- O 15=NO CROPS
- O 16=Other, specify __________
- O 99=Don't know
- O 98=No response

35 Where do members of this household have the farmland/agricultural land for crop farming?

- O 1=On the property of the house (i.e. back-/courtyard)
- O 4=Outside the house in the community
- O 2=Outside the community
- O 5=Not applicable (no crop farming)
- O 3=Other, specify __________
- O 99=Don't know
- O 98=No response

36 What is the extent of the crop farming that members of this household practice?

- O 1=Own demand ("backyard farming")
- O 2=Not applicable (no crop farming)
- O 3=Mass/Industrial demand ("mass crop farming")
- O 4=Other, specify __________
- O 99=Don't know
- O 98=No response

37 Do members of this household usually use herbicides, pesticides and fertilizers for the crop farming as stated in question 34?

Instruction: Tick one answer per category only.

Herbicides	Pesticides	Fertilizers
O 1=Yes, specify herbicide ____________________	O 1=Yes, specify pesticide ____________________	O 1=Yes, specify fertilizer ____________________

For peer review only - http://bmjopen.bmj.com/site/about/guidelines.xhtml

Site ___ ___ ___ Subarea ___ ___ ___ House number ___ ___ ___ ___ Interviewer ID ___ ___

O 2=Not applicable (no crop farming)<br>O 3=No use of herbicides<br>O 99=Don't know<br>O 98=No response	O 2=Not applicable (no crop farming)<br>O 3=No use of pesticides<br>O 99=Don't know<br>O 98=No response	O 2=Not applicable (no crop farming)<br>O 3=No use of fertilizers<br>O 99=Don't know<br>O 98=No response

38 Do members of this household usually wash their hands after the fieldwork for the crop farming as stated in question 34?

O 1=Yes, wash hands with soap and water
O 2=Yes, rub hands clean with some leaves/straw/grass
O 3=Yes, rub hands clean with some cloth/fabric
O 4=Not applicable (no crop farming)
O 5=Yes, wash hands with water only
O 6=Yes, rub hands clean with some sand
O 7=No, don't clean my hands
O 8=Other, specify __________
O 99=Don't know
O 98=No response

For peer review only

For peer review only - http://bmjopen.bmj.com/site/about/guidelines.xhtml

Site ___ ___ ___ Subarea ___ ___ ___ House number ___ ___ ___ ___ Interviewer ID ___ ___

FORM 4: ASSESSMENT OF OVERALL, AGE- AND SEX-STRATIFIED GENERIC HEALTHCARE UTILIZATION

Part A – Assessment of *generic* healthcare utilization

Instructions to the interviewer

- Complete this form only if the respondent consented to participate (see "Form 2 - CONSENT FORM"); the respondent as defined for this investigation is an adult (at the country-specific legal age of majority) household member and decision-maker with respect to daily healthcare/healthcare utilization for the entire household and its members; it must not be e.g. a neighbour or a visitor.
- Refer to "Form 3 – Part A - GENERAL HOUSEHOLD INFORMATION" as a reference to obtain the total number of household members as well as age and sex of each household member.
- Collect data about all household members regardless of whether all household members are present at the time of the interview; collecting and recording data only about individuals present at the time of the interview will result in biased data.
- A household is/household members are defined as a person or a group of related or unrelated persons that live together in the same dwelling unit, that acknowledge one male or female adult as the head of the household, that share the same housekeeping arrangements, that are considered to constitute one unit, and that provide themselves with food or other essentials for living.
- "Site" and "Subarea" must be entered each as a 3-digit, "House number" as a 4-digit, and "Interviewer ID" as a 2-digit code (use lists individually prepared for each site to obtain codes). "Study label" must be a combination of a 3-digit "Site" code, a 3-digit "Subarea" code and a 4-digit "House number" code; the "Interviewer ID" must be entered as a 3-digit code.
- Abbreviation: HCF=private/public primary/secondary/tertiary healthcare facility.

HOUSEHOLD MEMBERS

1 Record the total number of household members of this house (at the day of visit)**.**

Instructions: The total number of household members must be recorded as a 2-digit number; see also "Form 3 – Part A - GENERAL HOUSEHOLD INFORMATION".

Total number of household members: ____ ____

2 Record the number of FEMALE household members by each of the following age groups:

Instructions: The total number of female household members by age group must be recorded as a 2-digit number; see also "Form 3 – Part A - GENERAL HOUSEHOLD INFORMATION".

<2years: ____ ____ ≥2 to <5years: ____ ____ ≥5 to <15years: ____ ____ ≥15years: ____ ____

3 Record the number of MALE household members by each of the following age groups:

Instructions: The total number of male household members by age group must be recorded as a 2-digit number; see also "Form 3 – Part A - GENERAL HOUSEHOLD INFORMATION".

<2years: ____ ____ ≥2 to <5years: ____ ____ ≥5 to <15years: ____ ____ ≥15years: ____ ____

HEALTHCARE SEEKING BEHAVIOR

4 Record where female/male household members <2years/≥2 to <5years/≥5 to <15years/≥15years of age usually seek healthcare for the following signs/symptoms. Please rate using a range from 1-4.

Instructions:

- Complete question 4 separately for female and male household members <2years, ≥2 to <5years, ≥5 to <15years and ≥15years of age and circle the sex and age group Q4 was completed for; skip question 4 if there is no female/male household member <2years/ ≥2 to <5years/ ≥5 to <15years/ ≥15years of age.
- Provide an answer to each healthcare option for each sign/symptom; use range 1-4 for rating (1=1st choice; 2=2nd choice; 3=3rd choice; 4=not chosen); enter a code where applicable by using prepared lists (use lists individually prepared for each site to obtain codes); tick "Don't know" if applicable.

For peer review only - http://bmjopen.bmj.com/site/about/guidelines.xhtml

Site ___ ___ ___ Subarea ___ ___ ___ House number ___ ___ ___ ___ Interviewer ID ___ ___

Sign/Symptom	Healthcare options									
	Study-HCF		Other HCF		Physician	Pharmacy	Traditional healer	Nowhere Self-treatment	Nowhere Nothing	Don't know
	Rate	Code	Rate	Code	Rate	Rate	Rate	Rate	Rate	Tick
**1** [**A**] Fever, any	O=1<br>O=2<br>O=3<br>O=4		O=1<br>O=2<br>O=3<br>O=4		O=1<br>O=2<br>O=3<br>O=4	O=1<br>O=2<br>O=3<br>O=4	O=1<br>O=2<br>O=3<br>O=4	O=1<br>O=2<br>O=3<br>O=4	O=1<br>O=2<br>O=3<br>O=4	O
**2** Fever <3days (continuous)	O=1<br>O=2<br>O=3<br>O=4		O=1<br>O=2<br>O=3<br>O=4		O=1<br>O=2<br>O=3<br>O=4	O=1<br>O=2<br>O=3<br>O=4	O=1<br>O=2<br>O=3<br>O=4	O=1<br>O=2<br>O=3<br>O=4	O=1<br>O=2<br>O=3<br>O=4	O
**3** Fever ≥3days (continuous)	O=1<br>O=2<br>O=3<br>O=4		O=1<br>O=2<br>O=3<br>O=4		O=1<br>O=2<br>O=3<br>O=4	O=1<br>O=2<br>O=3<br>O=4	O=1<br>O=2<br>O=3<br>O=4	O=1<br>O=2<br>O=3<br>O=4	O=1<br>O=2<br>O=3<br>O=4	O
**4** Chills Shivering	O=1<br>O=2<br>O=3<br>O=4		O=1<br>O=2<br>O=3<br>O=4		O=1<br>O=2<br>O=3<br>O=4	O=1<br>O=2<br>O=3<br>O=4	O=1<br>O=2<br>O=3<br>O=4	O=1<br>O=2<br>O=3<br>O=4	O=1<br>O=2<br>O=3<br>O=4	O
**5** Convulsion	O=1<br>O=2<br>O=3<br>O=4		O=1<br>O=2<br>O=3<br>O=4		O=1<br>O=2<br>O=3<br>O=4	O=1<br>O=2<br>O=3<br>O=4	O=1<br>O=2<br>O=3<br>O=4	O=1<br>O=2<br>O=3<br>O=4	O=1<br>O=2<br>O=3<br>O=4	O
**6** Weight loss	O=1<br>O=2<br>O=3<br>O=4		O=1<br>O=2<br>O=3<br>O=4		O=1<br>O=2<br>O=3<br>O=4	O=1<br>O=2<br>O=3<br>O=4	O=1<br>O=2<br>O=3<br>O=4	O=1<br>O=2<br>O=3<br>O=4	O=1<br>O=2<br>O=3<br>O=4	O
**7** Dehydration	O=1<br>O=2<br>O=3<br>O=4		O=1<br>O=2<br>O=3<br>O=4		O=1<br>O=2<br>O=3<br>O=4	O=1<br>O=2<br>O=3<br>O=4	O=1<br>O=2<br>O=3<br>O=4	O=1<br>O=2<br>O=3<br>O=4	O=1<br>O=2<br>O=3<br>O=4	O
**8** Malaise Weakness/ Fatigue	O=1<br>O=2<br>O=3<br>O=4		O=1<br>O=2<br>O=3<br>O=4		O=1<br>O=2<br>O=3<br>O=4	O=1<br>O=2<br>O=3<br>O=4	O=1<br>O=2<br>O=3<br>O=4	O=1<br>O=2<br>O=3<br>O=4	O=1<br>O=2<br>O=3<br>O=4	O
**9** Aches/ Pain (muscle/joint/bone)	O=1<br>O=2<br>O=3<br>O=4		O=1<br>O=2<br>O=3<br>O=4		O=1<br>O=2<br>O=3<br>O=4	O=1<br>O=2<br>O=3<br>O=4	O=1<br>O=2<br>O=3<br>O=4	O=1<br>O=2<br>O=3<br>O=4	O=1<br>O=2<br>O=3<br>O=4	O
**10** Headache	O=1<br>O=2<br>O=3<br>O=4		O=1<br>O=2<br>O=3<br>O=4		O=1<br>O=2<br>O=3<br>O=4	O=1<br>O=2<br>O=3<br>O=4	O=1<br>O=2<br>O=3<br>O=4	O=1<br>O=2<br>O=3<br>O=4	O=1<br>O=2<br>O=3<br>O=4	O
**11** Dizziness/ Confusion Unconsciousness	O=1<br>O=2<br>O=3<br>O=4		O=1<br>O=2<br>O=3<br>O=4		O=1<br>O=2<br>O=3<br>O=4	O=1<br>O=2<br>O=3<br>O=4	O=1<br>O=2<br>O=3<br>O=4	O=1<br>O=2<br>O=3<br>O=4	O=1<br>O=2<br>O=3<br>O=4	O
**12** Blood pressure disorders	O=1<br>O=2<br>O=3<br>O=4		O=1<br>O=2<br>O=3<br>O=4		O=1<br>O=2<br>O=3<br>O=4	O=1<br>O=2<br>O=3<br>O=4	O=1<br>O=2<br>O=3<br>O=4	O=1<br>O=2<br>O=3<br>O=4	O=1<br>O=2<br>O=3<br>O=4	O

For peer review only

For peer review only - http://bmjopen.bmj.com/site/about/guidelines.xhtml

Site ___ ___ ___ Subarea ___ ___ ___ House number ___ ___ ___ ___ Interviewer ID ___ ___

Sign/Symptom	Healthcare options									
	Study-HCF		Other HCF		Physician	Pharmacy	Traditional healer	Nowhere Self-treatment	Nowhere Nothing	Don't know
	Rate	Code	Rate	Code	Rate	Rate	Rate	Rate	Rate	Code
**13** Heart disorders	O=1<br>O=2<br>O=3<br>O=4		O=1<br>O=2<br>O=3<br>O=4		O=1<br>O=2<br>O=3<br>O=4	O=1<br>O=2<br>O=3<br>O=4	O=1<br>O=2<br>O=3<br>O=4	O=1<br>O=2<br>O=3<br>O=4	O=1<br>O=2<br>O=3<br>O=4	O
**14** Nausea Vomitting	O=1<br>O=2<br>O=3<br>O=4		O=1<br>O=2<br>O=3<br>O=4		O=1<br>O=2<br>O=3<br>O=4	O=1<br>O=2<br>O=3<br>O=4	O=1<br>O=2<br>O=3<br>O=4	O=1<br>O=2<br>O=3<br>O=4	O=1<br>O=2<br>O=3<br>O=4	O
**15** Diarrhea (watery/ bloody/ mucopurulent)	O=1<br>O=2<br>O=3<br>O=4		O=1<br>O=2<br>O=3<br>O=4		O=1<br>O=2<br>O=3<br>O=4	O=1<br>O=2<br>O=3<br>O=4	O=1<br>O=2<br>O=3<br>O=4	O=1<br>O=2<br>O=3<br>O=4	O=1<br>O=2<br>O=3<br>O=4	O
**16** Abdominal pain Intestinal pain	O=1<br>O=2<br>O=3<br>O=4		O=1<br>O=2<br>O=3<br>O=4		O=1<br>O=2<br>O=3<br>O=4	O=1<br>O=2<br>O=3<br>O=4	O=1<br>O=2<br>O=3<br>O=4	O=1<br>O=2<br>O=3<br>O=4	O=1<br>O=2<br>O=3<br>O=4	O
**17** Blood loss/ Bleeding (internal)	O=1<br>O=2<br>O=3<br>O=4		O=1<br>O=2<br>O=3<br>O=4		O=1<br>O=2<br>O=3<br>O=4	O=1<br>O=2<br>O=3<br>O=4	O=1<br>O=2<br>O=3<br>O=4	O=1<br>O=2<br>O=3<br>O=4	O=1<br>O=2<br>O=3<br>O=4	O
**18** Blood loss/ Bleeding (external)	O=1<br>O=2<br>O=3<br>O=4		O=1<br>O=2<br>O=3<br>O=4		O=1<br>O=2<br>O=3<br>O=4	O=1<br>O=2<br>O=3<br>O=4	O=1<br>O=2<br>O=3<br>O=4	O=1<br>O=2<br>O=3<br>O=4	O=1<br>O=2<br>O=3<br>O=4	O
**19** Sneezing Runny nose	O=1<br>O=2<br>O=3<br>O=4		O=1<br>O=2<br>O=3<br>O=4		O=1<br>O=2<br>O=3<br>O=4	O=1<br>O=2<br>O=3<br>O=4	O=1<br>O=2<br>O=3<br>O=4	O=1<br>O=2<br>O=3<br>O=4	O=1<br>O=2<br>O=3<br>O=4	O
**20** Cough	O=1<br>O=2<br>O=3<br>O=4		O=1<br>O=2<br>O=3<br>O=4		O=1<br>O=2<br>O=3<br>O=4	O=1<br>O=2<br>O=3<br>O=4	O=1<br>O=2<br>O=3<br>O=4	O=1<br>O=2<br>O=3<br>O=4	O=1<br>O=2<br>O=3<br>O=4	O
**21** Rapid breathing/ shortness of breath	O=1<br>O=2<br>O=3<br>O=4		O=1<br>O=2<br>O=3<br>O=4		O=1<br>O=2<br>O=3<br>O=4	O=1<br>O=2<br>O=3<br>O=4	O=1<br>O=2<br>O=3<br>O=4	O=1<br>O=2<br>O=3<br>O=4	O=1<br>O=2<br>O=3<br>O=4	O
**22** Severe breathing/ Difficulties in breathing	O=1<br>O=2<br>O=3<br>O=4		O=1<br>O=2<br>O=3<br>O=4		O=1<br>O=2<br>O=3<br>O=4	O=1<br>O=2<br>O=3<br>O=4	O=1<br>O=2<br>O=3<br>O=4	O=1<br>O=2<br>O=3<br>O=4	O=1<br>O=2<br>O=3<br>O=4	O
**23** Skin inflammation (rashes/ redness/ pus/ itching)	O=1<br>O=2<br>O=3<br>O=4		O=1<br>O=2<br>O=3<br>O=4		O=1<br>O=2<br>O=3<br>O=4	O=1<br>O=2<br>O=3<br>O=4	O=1<br>O=2<br>O=3<br>O=4	O=1<br>O=2<br>O=3<br>O=4	O=1<br>O=2<br>O=3<br>O=4	O
**24** Swelling/ Edema	O=1<br>O=2<br>O=3<br>O=4		O=1<br>O=2<br>O=3<br>O=4		O=1<br>O=2<br>O=3<br>O=4	O=1<br>O=2<br>O=3<br>O=4	O=1<br>O=2<br>O=3<br>O=4	O=1<br>O=2<br>O=3<br>O=4	O=1<br>O=2<br>O=3<br>O=4	O
**25** Jaundice	O=1<br>O=2<br>O=3<br>O=4		O=1<br>O=2<br>O=3<br>O=4		O=1<br>O=2<br>O=3<br>O=4	O=1<br>O=2<br>O=3<br>O=4	O=1<br>O=2<br>O=3<br>O=4	O=1<br>O=2<br>O=3<br>O=4	O=1<br>O=2<br>O=3<br>O=4	O

For peer review only - http://bmjopen.bmj.com/site/about/guidelines.xhtml

Site ___ ___ ___ Subarea ___ ___ ___ House number ___ ___ ___ ___ Interviewer ID ___ ___

FORM 4: ASSESSMENT OF OVERALL, AGE- AND SEX-STRATIFED GENERIC HEALTHCARE UTILIZATION

Part B – Healthcare-associated factors/Vaccination/Disease perception

TRAVEL HEALTHCARE FACILITY

1 In case a healthcare facility (HCF) was rated the 1st choice of care in question 4A (fever), what is the main mode of transportation, travel time (depending on mode of transportation), travel cost (depending on mode of transportation) and travel distance (depending on mode of transportation) that is usually taken to get there?

Instructions: Enter one code per category and sex as well as age group only. Skip this question if there is no female/male member <2years/≥2 to <5years/≥5 to <15years/≥15years of age in this household (see also question 2 and 3 of this form PartA). Skip this question if no HCF was rated the 1st choice of care in question 4A for female/male member <2years/≥2 to <5years/≥5 to <15years/≥15years of age. Record the travel time as minute(s) [min]/ hour(s) [hr], the total costs in USD, including potential gifts, favours and tips, and the total distance as meter(s) [m]/kilometre(s) [km].

Female				Male			
<2 years	≥2 to <5 years	≥5 to <15 years	≥15 years	<2 years	≥2 to <5 years	≥5 to <15 years	≥15 years
**1.1 Transportation mode**							
Code ___	Code ___	Code ___	Code ___	Code ___	Code ___	Code ___	Code ___
[1] Private transportation (i.e. motorbike, car, bus) [2] Public transportation (i.e. motorbike, car, bus) [3] Walking [4] Bicycle [5] Other, specify _________ [99] Don't know [98] No response							
**1.2 Travel time**							
Code ___	Code ___	Code ___	Code ___	Code ___	Code ___	Code ___	Code ___
[1] <30 mins [2] ≥30 to <60 mins [3] ≥1.0 to <2.0 hrs [4] ≥2.0 to <3.0 hrs [5] ≥3.0 to <4.0 hrs [6] ≥4.0 hrs [99] Don't know [98] No response							
**1.3 Travel cost**							
Code ___	Code ___	Code ___	Code ___	Code ___	Code ___	Code ___	Code ___
[1] <1.0 USD [2] ≥1.0 to <3.0 USD [3] ≥3.0 to <5.0 USD [4] ≥5.0 to <10.0 USD [5] ≥10.0 USD [99] Don't know [98] No response							
**1.4 Travel distance**							
Code ___	Code ___	Code ___	Code ___	Code ___	Code ___	Code ___	Code ___
[1] <500 m [2] ≥500 m to <1.0 km [3] ≥1.0 to <2.0 km [4] ≥2.0 to <3.0 km [5] ≥3.0 to <4.0 km [6] ≥4.0 km [99] Don't know [98] No response							

INSURANCE

2 For which members of this household does the insurance cover health expenses?

Instructions: Enter one code per category. Skip this question if there is no member <2years/≥2 to <5years/≥5 to <15years/≥15years of age in this household (see question 2 and 3 of this form PartA).

<2 years	≥2 to <5 years	≥5 to <15 years	≥15 years
Code ___________	Code ___________	Code ___________	Code ___________
[1] Yes [2] No [99] Don't know [98] No response			

For peer review only - http://bmjopen.bmj.com/site/about/guidelines.xhtml

Site ___ ___ ___ Subarea ___ ___ ___ House number ___ ___ ___ ___ Interviewer ID ___ ___

3 What kind of health expenses are usually covered by the insurance for members of this household?

Instructions: Enter one code by category and age group only. Skip this question if there is no member <2years/≥2 to <5years/≥5 to <15years/≥15years of age in this household (see also question 2 and 3 of this form PartA). Skip this question if there is no insurance that covers health expenses for <2years/≥2 to <5years/≥5 to <15years/≥15years of age.

	<2 years	≥2 to <5years	≥5 to <15years	≥15years
	[1] Yes	[2] No	[99] Don't know	[98] No response
**1** Registration	Code ______	Code _______	Code _______	Code _____
**2** Consultation/ Doctor's visit	Code ______	Code _______	Code _______	Code _____
**3** Physical examination - general	Code ______	Code _______	Code _______	Code _____
**4** Physical examination – specific, specify_______	Code ______	Code _______	Code _______	Code _____
**5** Diagnostics - general laboratory diagnostics	Code ______	Code _______	Code _______	Code _____
**6** Diagnostics - specific laboratory diagnostics, specify__________	Code ______	Code _______	Code _______	Code _____
**7** Diagnostics - general imaging	Code ______	Code _______	Code _______	Code _____
**8** Diagnostics - specific imaging, specify__________	Code ______	Code _______	Code _______	Code _____
**9** Diagnostics - other, specify________	Code ______	Code _______	Code _______	Code _____
**10** Treatment - general medication	Code ______	Code _______	Code _______	Code _____
**11** Treatment - specific medication, specify________	Code ______	Code _______	Code _______	Code _____
**12** Treatment - other medication, specify__________	Code ______	Code _______	Code _______	Code _____
**13** Treatment - interventions (other than surgery), specify_______	Code ______	Code _______	Code _______	Code _____
**14** Treatment Surgery	Code ______	Code _______	Code _______	Code _____
**15** Hospitalization <7days	Code ______	Code _______	Code _______	Code _____
**16** Hospitalization ≥7days	Code ______	Code _______	Code _______	Code _____
**17** Emergency services	Code ______	Code _______	Code _______	Code _____
**18** Antenatal care	Code ______	Code _______	Code _______	Code _____
**19** Postnatal care	Code ______	Code _______	Code _______	Code _____
**20** Preventive medicine	Code ______	Code _______	Code _______	Code _____
**21** Vaccination	Code ______	Code _______	Code _______	Code _____
**22** Home healthcare (i.e. elderly, disabled, chronic/long-term diseased)	Code ______	Code _______	Code _______	Code _____
**23** Supportive therapy (i.e. physiotherapy, ergotherapy, rehabilitation)	Code ______	Code _______	Code _______	Code _____

For peer review only - http://bmjopen.bmj.com/site/about/guidelines.xhtml

Site ___ ___ ___ Subarea ___ ___ ___ House number ___ ___ ___ ___ Interviewer ID ___ ___

ETHNOGRAPHY

4 To which religion/ethnic group/tribe belong members of this household?

Instructions: Give one answer by age group only. Skip this question if there is no member >2years/≥2 to <5years/≥5 to ≤15years/≥15years of age in this household (see also question 2 and 3 of this form PartA).

	<2 years	≥2 to <5years	≥5 to <15years	≥15years
Religion/Ethnic group/Tribe	___________	___________	___________	___________
	O 99=Don't know<br>O 98=No response	O 99=Don't know<br>O 98=No response	O 99=Don't know<br>O 98=No response	O 99=Don't know<br>O 98=No response

5 Does the religion/ethnic group/tribe as stated in question 4 influence the various aspects of utilization of healthcare for members of this household?

Instructions: Enter one code by category and age group only. Skip this question if there is member >2years/≥2 to <5years/≥5 to <15years/≥15years of age in this household (see also question 2 and 3 of this form PartA).

	<2 years	≥2 to <5years	≥5 to <15years	≥15years
	[1] Yes	[2] No	[99] Don't know	[98] No response
**1** Visiting a healthcare facility (i.e. hospital, health care center, health post)	Code _____	Code _______	Code _______	Code _____
**2** Visiting a physician	Code _____	Code _______	Code _______	Code _____
**3** Visiting a pharmacy	Code _____	Code _______	Code _______	Code _____
**4** Visiting a traditional healer	Code _____	Code _______	Code _______	Code _____
**5** Deciding for self-treatment	Code _____	Code _______	Code _______	Code _____
**6** Deciding for non-treatment	Code _____	Code _______	Code _______	Code _____
**7** Physical examination – general	Code _____	Code _______	Code _______	Code _____
**8** Physical examination – specific, specify________	Code _____	Code _______	Code _______	Code _____
**9** Collection of specimen (i.e. blood, urine, stool, smear, swab, cerebrospinal fluid)	Code _____	Code _______	Code _______	Code _____
**10** Diagnostics – general laboratory diagnostics	Code _____	Code _______	Code _______	Code _____
**11** Diagnostics – specific laboratory diagnostics, specify___________	Code _____	Code _______	Code _______	Code _____
**12** Diagnostics - general imaging	Code _____	Code _______	Code _______	Code _____
**13** Diagnostics - specific imaging, specify_________	Code _____	Code _______	Code _______	Code _____
**14** Diagnostics - other, specify_________	Code _____	Code _______	Code _______	Code _____
**15** Treatment - general medication	Code _____	Code _______	Code _______	Code _____
**16** Treatment - specific medication, specify________	Code _____	Code _______	Code _______	Code _____
**17** Treatment - other medication, specify___________	Code _____	Code _______	Code _______	Code _____

For peer review only - http://bmjopen.bmj.com/site/about/guidelines.xhtml

Site ___ ___ ___ Subarea ___ ___ ___ House number ___ ___ ___ ___ Interviewer ID ___ ___

**18** Treatment - interventions (other than surgery), specify________________	Code _____	Code _______	Code _______	Code _____
**19** Treatment - Surgery	Code _____	Code _______	Code _______	Code _____
**20** Hospitalization <7days	Code _____	Code _______	Code _______	Code _____
**21** Hospitalization ≥7days	Code _____	Code _______	Code _______	Code _____
**22** Emergency services	Code _____	Code _______	Code _______	Code _____
**23** Antenatal care	Code _____	Code _______	Code _______	Code _____
**24** Postnatal care	Code _____	Code _______	Code _______	Code _____
**25** Preventive medicine	Code _____	Code _______	Code _______	Code _____
**26** Vaccination	Code _____	Code _______	Code _______	Code _____
**27** Home healthcare (i.e. elderly, disabled, chronic/long-term diseased)	Code _____	Code _______	Code _______	Code _____
**28** Supportive therapy (i.e. physiotherapy, ergotherapy, rehabilitation)	Code _____	Code _______	Code _______	Code _____

VACCINATION

6 How were children of this household fed during the first six months of life?

Instructions: Skip this question if there are no male/female children in this household (see also question 2 and 3 of this form PartA).

- O 1=Breastfed exclusively
- O 2=Breastfed and other feeds, specify ______
- O 3=Formula and other feeds, specify ________
- O 4=Breastfed and formula
- O 5=Formula exclusively
- O 6=Other, specify ________________
- O 99=Don't know
- O 98=No response

7 Which water source was usually used to prepare formula or formula and other feeds as stated in question 6 for children of this household?

Instructions: Skip this question if there are no children in this household (see also question 2 and 3 of this form PartA).

- O 1=Tap water (inside the house; private)
- O 2=Tap water (outside the house i.e. back-/courtyard; private)
- O 3=Tap water (outside the house; public, shared)
- O 4=Standpipe/Piped water (inside the house; private)
- O 5=Standpipe/Piped water (outside the house i.e. back-/courtyard; private)
- O 6=Standpipe/Piped water (outside the house; public, shared)
- O 7=Water truck (public; shared)
- O 8=Bottled water
- O 9=Surface water (i.e. lake, pond, river, stream)
- O 10=Surface water (i.e. canal, irrigation canal)
- O 11=Spring water
- O 12=Rain water
- O 13=Well/Borehole (open/uncovered/unprotected)
- O 14=Well/Borehole (covered/protected)
- O 15=NO FORMULA/OTHER FEEDS PREPARED
- O 16=Other, specify _____________
- O 99=Don't know
- O 98=No response

8 Did children of this household receive any vaccines since birth, including the birth vaccines?

Instructions: Enter one code by category and child only. Skip this question if there are no children in this household (see also question 2 and 3 of this form PartA). Refer to question 9 of "Form 3 Part A – Demographic data/General household information" and record the initials/identifier of the member(s).

Child 1	Child 2	Child 3	Child 4
Initials/Identifier: _____	Initials/Identifier: _____	Initials/Identifier: _____	Initials/Identifier: _____

For peer review only - http://bmjopen.bmj.com/site/about/guidelines.xhtml

Site ___ ___ ___ Subarea ___ ___ ___ House number ___ ___ ___ ___ Interviewer ID ___ ___

[1] YES, vaccinated	[5] No, lack of vaccine	[9] No, HCF busy
[2] No, caretaker busy	[6] No, child too old/young	[10] No, child sick
[3] No, caretaker decided no need	[7] No, caretaker refused/too worried	[11] No, no vaccination card
[4] No, unknown where to go	[8] No, other, specify ___________	[99] Don't know [98] No response

**8.1 Diphtheria and Tetanus and Pertussis and *Haemophilus influenza* and Hepatitis B vaccine/DTwPHibHepB** (e.g. 6/8, 10/12, 14/16 weeks)			
Code ___________	Code ___________	Code ___________	Code ___________
**8.2 Tetanus toxoid (TT)** (e.g. 1st contact pregnancy, 1, 6, 12/24 months)			
Code ___________	Code ___________	Code ___________	Code ___________
**8.3 Tetanus and diphtheria toxoid (TD)** (e.g. 1st contact pregnancy, 1, 6, 12 months)			
Code ___________	Code ___________	Code ___________	Code ___________
**8.4 Tuberculosis/BCG** (at birth)			
Code ___________	Code ___________	Code ___________	Code ___________
**8.5 Rotavirus/RV** (e.g. 6/8, 10/12, 16 weeks)			
Code ___________	Code ___________	Code ___________	Code ___________
**8.6 Measles/Measles and Rubella (MR)** (e.g. 9, 15/18months)			
Code ___________	Code ___________	Code ___________	Code ___________
**8.7 Polio/OPV** (e.g. birth, 6/8, 10/12, 14/16 weeks)			
Code ___________	Code ___________	Code ___________	Code ___________
**8.8 Pneumococcus/PCV** (e.g. 6/8, 10/12, 14/16 weeks)			
Code ___________	Code ___________	Code ___________	Code ___________
**8.9 Yellow fever (YF)** (e.g. 9 months)			
Code ___________	Code ___________	Code ___________	Code ___________
**8.10 Other Vaccines**			
Specify, ___________	Specify, ___________	Specify, ___________	Specify, ___________
Specify, ___________	Specify, ___________	Specify, ___________	Specify, ___________
Specify, ___________	Specify, ___________	Specify, ___________	Specify, ___________
Specify, ___________	Specify, ___________	Specify, ___________	Specify, ___________

For peer review only

For peer review only - http://bmjopen.bmj.com/site/about/guidelines.xhtml

Site ___ ___ ___ Subarea ___ ___ ___ House number ___ ___ ___ ___ Interviewer ID ___ ___

DISEASE PERCEPTION

9 Knowledge, perception, causes and prevention of common infectious diseases

Disease	**9.1 Disease known by household members?** [1] Yes [2] No [99] Don't know [98] No response	**9.2 Perception of disease by household members?** *Instruction:* Describe briefly if known.	**9.3 Causes for disease known by household members?** *Instructions:* Enter all codes (list A) that apply; more than one answer is possible.	**9.4 Measures to prevent disease known by household members?** *Instructions:* Enter all codes (list B) that apply; more than one answer is possible.	**9.5 Measures to prevent disease utilized by household members?** *Instructions:* Enter all codes (list B) that apply; more than one answer is possible.
**A.** Typhoid fever	Code ___________		Code ___________	Code ___________	Code ___________
**B.** Cholera	Code ___________		Code ___________	Code ___________	Code ___________
**C.** Malaria	Code ___________		Code ___________	Code ___________	Code ___________
**D.** Influenza	Code ___________		Code ___________	Code ___________	Code ___________
**E.** Meningitis	Code ___________		Code ___________	Code ___________	Code ___________
**F.** Hepatitis E	Code ___________		Code ___________	Code ___________	Code ___________
**G.** Tuberculosis	Code ___________		Code ___________	Code ___________	Code ___________
**H.** HIV/AIDS	Code ___________		Code ___________	Code ___________	Code ___________

List A: Possible causes	List B: Possible preventive measures
[1] Alcohol consumption<br>[2] Weather/witchcraft<br>[3] Mosquito/insect bite<br>[4] Dog bite	[1] No alcohol consumption<br>[2] Sleeping under bed net<br>[3] Using mosquito/insect spray<br>[4] Maintain a clean house and immediate surroundings of the house

For peer review only

For peer review only - http://bmjopen.bmj.com/site/about/guidelines.xhtml

Site ___ ___ ___ Subarea ___ ___ ___ House number ___ ___ ___ ___ Interviewer ID ___ ___

[5] Drinking untreated surface/spring water (i.e. lake, pond, river, stream, canal/irrigation canal)<br>[6] Close body contact/kissing/hugging<br>[7] Eating raw food items such as vegetables/lettuce, fruits, dairy products, eggs, meat or fish<br>[8] Not cleaning hands properly when preparing food/meals<br>[9] Not cleaning hands properly before/after visiting a toilet<br>[10] Contact to human blood (i.e. injection, transfusion, surgery)<br>[11] Consuming raw animal blood<br>[12] Other, specify _______________<br>[99] Don't know<br>[98] No response	[5] Practice proper disposal of human and domestic waste<br>[6] Avoiding close contact to dogs<br>[7] Treating water (i.e. boiling, filtration, solar disinfection, sedimentation, chlorination)<br>[8] Not cooking/preparing food/meals with untreated water<br>[9] Not washing food items to be consumed raw with untreated water<br>[10] Re-boiling/re-heating left-over/prepared food/meals prior to consumption<br>[11] Not eating raw food items such as vegetables/lettuce, fruits, dairy products, eggs, meat or fish<br>[12] Cover food items/meals<br>[13] Protect food items/meals from flies<br>[14] Avoid food/beverage from street vendors<br>[15] Avoid close contact to sick people<br>[16] No close body contact/kissing/hugging<br>[17] Covering nose/mouth while sneezing/coughing<br>[18] No contact/consumption of human/animal blood<br>[19] Practicing proper hand hygiene when preparing food/meals (i.e. washing hands with soap and water, rub hands clean)<br>[20] Practicing proper hand hygiene before/after visiting a toilet (i.e. washing hands with soap and water, rub hands clean)<br>[21] Other, specify ___________________<br>[99] Don't know<br>[98] No response

For peer review only

For peer review only - http://bmjopen.bmj.com/site/about/guidelines.xhtml

Site ___ ___ ___ Subarea ___ ___ ___ House number ___ ___ ___ ___ Interviewer ID ___ ___

10 For which members of this household are the preventive measures as listed in question 9.5 against typhoid fever used?

Instructions: Tick all that applies; more than one answer is possible.

O 1=<2years O 3=≥5 to <15years O 5=NOT USED O 99=Don't know
O 2=≥2 to < 5years O 4=≥15years O 98=No response

11 Name the household members that were diagnosed with typhoid fever during the past 3 months. Which diagnostics were used to confirm typhoid fever?

Instructions: Enter all codes that apply for each household member; more than one answer is possible.

Member 1<br>Initials/Identifier: ______	Member 2<br>Initials/Identifier: ______	Member 3<br>Initials/Identifier: ______	Member 4<br>Initials/Identifier: ______
[1] Blood culture<br>[2] Stool culture	[3] Blood/Widal test<br>[4] Other test, specify_____________	[5] Not applicable (not diagnosed with typhoid fever)<br>[99] Don't know	[98] No response
Code ___________	Code ___________	Code ___________	Code ___________

12 (Where were members of this household recorded in question 11 diagnosed for typhoid fever?) Where did members of this household seek care?

Instructions: Enter one code by household member only.

Member 1<br>Initials/Identifier: ______	Member 2<br>Initials/Identifier: ______	Member 3<br>Initials/Identifier: ______	Member 4<br>Initials/Identifier: ______
[1] Healthcare facility, specify ___________<br>[2] Physician<br>[3] Nowhere, self-treatment<br>[4] Traditional healer<br>[5] Pharmacy		[6] Nowhere, did not seek care<br>[7] Other, specify ____________<br>[8] Not applicable<br>[99] Don't know<br>[98] No response	
Code ___________	Code ___________	Code ___________	Code ___________

13 (Did members of this household that were diagnosed with typhoid fever as recorded in question 11 get any treatment?) Which treatment did they get?

Instructions: Tick one answer by household member only.

Member 1<br>Initials/Identifier: ______	Member 2<br>Initials/Identifier: ______	Member 3<br>Initials/Identifier: ______	Member 4<br>Initials/Identifier: ______
O 1=Treatment, specify<br>_______________<br>O 2=Not applicable<br>O 99=Don't know<br>O 98=No response	O 1=Treatment, specify<br>_______________<br>O 2=Not applicable<br>O 99=Don't know<br>O 98=No response	O 1=Treatment, specify<br>_______________<br>O 2=Not applicable<br>O 99=Don't know<br>O 98=No response	O 1=Treatment, specify<br>_______________<br>O 2=Not applicable<br>O 99=Don't know<br>O 98=No response

For peer review only

For peer review only - http://bmjopen.bmj.com/site/about/guidelines.xhtml

Site ___ ___ ___ Subarea ___ ___ ___ House number ___ ___ ___ ___ Interviewer ID ___ ___

FORM 5: ASSESSMENT OF OVERALL, AGE-AND SEX-STRATIFIED ACTUAL HEALTHCARE UTILIZATION

HEALTHCARE SEEKING BEHAVIOR

1 Record below any occurrence of a sign(s)/ a symptom(s) (see List 1) and specify where (see List 2) and how often a member/members sought healthcare during the past 3 months?

Instructions: See "Form 3 - Part A - GENERAL HOUSEHOLD INFORMATION" to obtain the initials/identifier of each household member. Indicate one sign/symptom per line only. Start a new line if different healthcare options were selected for recurring signs/symptoms. Indicate the number of occurrences for each sign/symptom during the past 3 months. If a different healthcare option was taken for a recurring sign/symptom, indicate this on a new line. Please complete an additional form if >20 occurrences took place during the past 3 months.

**List 1: Sign/Symptom**		**List 2: Healthcare option**
[A] Fever, any<br>[B] Fever <3days (continuous)<br>[C] Fever ≥3days (continuous)<br>[D] Chills/ Shivering<br>[E] Convulsion<br>[F] Weight loss<br>[G] Dehydration<br>[H] Malaise/ Weakness/ Fatigue<br>[I] Aches/Pain (muscle/joint/bone)<br>[J] Headache<br>[K] Dizziness/Confusion/ Unconsciousness<br>[L] Blood pressure disorders<br>[M] Heart disorders	[N] Nausea/ Vomiting<br>[O] Diarrhea (watery/bloody/mucopurulent)<br>[P] Abdominal pain/ Intestinal pain<br>[Q] Blood loss/ Bleeding (internal)<br>[R] Blood loss/ Bleeding (external)<br>[S] Sneezing/ Runny nose<br>[T] Cough<br>[U] Rapid breathing/ Shortness of breath<br>[V] Severe breathing/ Difficulties in breathing<br>[W] Skin inflammation (rashes/redness/ pus/itching)<br>[X] Swelling/ Edema<br>[Y] Jaundice	[1] Study-HCF, Code __________<br>[2] Other HCF, Code __________<br>[3] Physician<br>[4] Pharmacy<br>[5] Traditional healer<br>[6] Nowhere/Self-treatment<br>[7] Nowhere/Nothing

**No.**	**Household member** Record the initials/identifier of a household member; see "Form 3 - Part A - GENERAL HOUSEHOLD INFORMATION"	**Sign/ Symptom** Use List 1 to select a code	**Healthcare option** Use List 2 to select a code	HCF code	**Frequency** Indicate the number of incidences for the sign/symptom listed
1					
2					
3					
4					
5					
6					
7					
8					
9					

For peer review only - http://bmjopen.bmj.com/site/about/guidelines.xhtml

Site ___ ___ ___ Subarea ___ ___ ___ House number ___ ___ ___ ___ Interviewer ID ___ ___

10					
11					
12					
13					
14					
15					
16					
17					
18					
19					
20					

For peer review only

For peer review only - http://bmjopen.bmj.com/site/about/guidelines.xhtml

Appendix 3/Supplementary file 3: Protocol, Definitions/Terminology and Study Forms (French)

Protocol

Le protocole d’étude HPAfrica: Évaluation du comportement de santé et des facteurs démographiques socio-économiques, comportement d’hygiène - une étude standardisée répétée transversale dans plusieurs cohortes en Afrique subsaharienne

Abstrait

Introduction L'objectif de l'étude HPAfrica est de déterminer les comportements sanitaires et~~ autres~~ facteurs démographiques, y compris les facteurs socio-économiques, ethnographiques aussi bien que d'hygiène et d'assainissement, sur les sites d'étude du programme Fièvre Typhoïde Sévère en Afrique (SETA). L'objectif du programme SETA est d'enquêter sur la surveillance de la fièvre au niveau des établissements de soins de santé au Burkina Faso, en République Démocratique du Congo, en Éthiopie, au Ghana, à Madagascar et au Nigéria. Les estimations précises de la charge de morbidité nécessitent un ajustement des styles de comportements de santé, qui sont supposés varier au sein d'une population d'étude.

Methodes et Analyses Pour la taille d'échantillon minimale d’interviews de ménages requise, les hypothèses d'une population infinie, d'un effet de design et d'une stratification par âge et par sexe sont considérées. En l'absence d'un cadre d'échantillonnage de la population ou d'une liste de ménages, une approche ~~géo~~spatiale sera utilisée pour générer des points aléatoires géographiques avec un outil ArcGIS (Aeronautical Reconnaissance Coverage Geographic Information System). Les impressions de l'imagerie satellite de Google Earth Pro® visualisent ces points. Les données d'intérêt seront évaluées au niveau des ménages en différentes saisons en appliquant un échantillonnage ~~simple, ~~stratifié en fonction de la population. Une application basée sur Android et un service Web ~~ont été mis au point ~~sera développé pour la capture de données électroniques et la synchronisation avec le serveur de base de données sur Internet en temps réel. Les poids d’échantillonnage seront calculés pour tenir compte des differences possibles dans les probabilitiés de selection. Des analyses de données descriptives seront effectuées afin d'évaluer l’informations de base de chaque population étudée et le comportement santé stratifié par âge et par sexe. Ceci permettra d'ajuster les estimations de la charge de morbidité. En outre, des analyses multivariées seront appliquées pour examiner les associations entre le comportement santé, les facteurs demographiques et le fardeau de la maladie trouvée dans l'étude SETA.

Les forces et les limites de cette étude

- La normalisation dans la recherche multi-pays /-site à base communautaire nécessite de la prudence compte tenu des variations de cadre, de langue et de culture.
- Les évaluations de santé publique à grande échelle complèteront les données sur la charge de morbidité en étudiant les sources de transmission et d’infection.
- Les données de population actuelles dans les sites d’étude et leurs sous-zones définies administrativement et les bases de sondage de population sont supposées être limitées.
- La collecte de données électroniques peut entraîner problèmes techniques liés aux dommages, aux pannes fonctionnelles ou à la perte de dispositifs.
- Une connexion Internet sans fil instable peut limiter les équipes de données à mettre à jour l'application basée sur Android, communiquer en temps réel avec les responsables de l'étude et à synchroniser les données collectées avec le serveur.

Inscription: non requise

Mots clés: comportement sanité/hygiénique, assainissement, socio-économique, cadre d’échantillonnage de la population/l’échantillonnage ~~sélection aléatoire geo~~spatiale, Afrique subsaharienne, HPAfrica étude

For peer review only - http://bmjopen.bmj.com/site/about/guidelines.xhtml

Contexte

L'évaluation du comportement de santé, y compris l'utilisation des soins de santé, est un déterminant important pour la production d'estimations précises du fardeau de la maladie parmi les populations cibles. Ceci est particulièrement important pour les études qui utilisent un modèle de surveillance observationnelle basé sur les établissements de santé, car une variation substantielle des comportements de santé influence fortement les estimations de la maladie (1). L'ajustement des calculs de la maladie pour des variations du comportement de santé augmentera l'exactitude de l’évaluation des mesuresla prévalence des maladies de la fréquence des maladies causées par des pathogènes bactériens, fongiques, viraux ou parasitaires parmi les populations étudiées. De plus, l'influence d'une grande variété de facteurs basés sur la population sur le comportement de santé et le fardeau de la maladie a été reconnue. Cela inclut des facteurs socioéconomiques tels que l'éducation, la profession, le revenu, la disponibilité des biens des ménages (2, 3), les facteurs ethniques / religieux et culturels (4, 5), mais aussi l'hygiène et l'assainissement (6-10), la manipulation des aliments (11-14) et les contacts avec des animaux (9).

L'étude **H**ealth **P**opulation **Africa** (HPAfrica) sera menée au Burkina Faso, en République démocratique du Congo (RDC), en Éthopie, au Ghana, à Madagascar et au Nigéria. Les expériences du programme Typhoid Fever Surveillance in Africa Program (TSAP) seront reportées sur le programme Severe Typhoid in Africa (SETA) (15, 16). Dans ce programme, une surveillance standardisée de la fièvre, basée sur les établissements de santé, est effectuée sur des sites d'étude sélectionnés dans les pays participants sur une période de deux ans (17). SETA vise principalement à collecter systématiquement des informations sur le fardeau des infections graves à *Salmonella*. Les sujets vivant dans la zone the chalandise définie, avec un épisode de fièvre aiguë, des antécédents de fièvre ≥ 3 jours consécutifs, une fièvre typhoïde cliniquement soupçonnée ou des perforations gastro-intestinales cliniquement diagnostiquées dues à la fièvre typhoïde seront éligibles. En outre, la sévérité des maladies provoquées par *Salmonella* et l'histoire naturelle de l'infection à *Salmonella* seront enregistrées avec des analyses supplémentaires sur les réponses immunitaires de l'hôte et le portage chronique.

Objectifs et Résultats

L'objectif de l'étude HPAfrica est d'évaluer le comportement de santé générique et réel stratifié par âge et par sexe. Le comportement générique se rapporte au comportement associé à la fièvre et à d'autres signes et symptômes sans l'apparition, alors que le comportement réel est lié à l'apparition (Formulaire 4 Partie A, Formulaire 5). Les données sont utilisées pour ajuster les mesures de l’occurrence de la maladie comme l’incidence d'agents pathogènes infectieux stratifiés selon l'âge et le sexe pour la proportion d'une population étudiée non capturée par les activités de HPAfrica dans le programme de surveillance SETA. Les données documentaires pour divers signes et symptômes ou une combinaison de ceux-ci sont utilisés pour une classification progressive de l'utilisation des soins de santé en fonction de la gravité de la maladie.

L’étude HPAfrica recueillera des données sur les facteurs associés aux soins de santé, tels que les modalités de voyage, la possession d'une assurance maladie, les facteurs culturels/ethnographiques, l'état vaccinal des enfants (18, 19) et la fréquence et la perception individuelle des maladies (Formulaire 4 Partie B). Les données démographiques (Formulaire 3 Partie A) seront utilisées pour la stratification par âge et par sexe de la population étudiée qui est nécessaire pour calculer les facteurs d'ajustement pour les incidences mesures de l’occurrence de la maladie. Les données socio-économiques (Formulaire 3 Partie B) seront utilisées pour calculer les indices de richesse pour les approches descriptives et analytiques des populations étudiées. Les informations sur l'hygiène et l'assainissement (Formulaire 3 Partie C) peuvent permettre une meilleure compréhension de l'occurrence et de la fréquence des agents pathogènes identifiés parmi les populations étudiées.

Méthodes

Sites d’étude

For peer review only - http://bmjopen.bmj.com/site/about/guidelines.xhtml

Les limites de chaque site seront définies en utilisant des indications préexistantes qui avaient été déterminées par les autorités statistiques ou ministères de la santé (Tableau 1), couplées à des données géospatiales à haute résolution de source ouverte. L'examen rétrospectif des registres de des deux dernières années aux établissements de soins de santé de recrutement SETA permettra de redéfinir les limites du site. On s'attend à ce que cette revue révèle les résidences ou au moins les zones résidentielles administratives plus larges telles que des communautés, des districts ou des villages de patients qui ont cherché des soins de santé pour une raison quelconque. Des cartes et des images satellites peuvent être utilisées pour mieux visualiser les résidences des patients et les limites générales, y compris les sous-zones définies géographiquement ou administrativement, les secteurs de recensement ou les strates de chaque site. Là où les limites ne peuvent être clairement définies, des facteurs supplémentaires, tels que la distance à un établissement de soins de santé de recrutement, peuvent être pris en compte.

Taille d'échantillons

Toutes les sources disponibles seront utilisées pour recueillir des données sur la population le plus à jour stratifiées selon l'âge et le sexe par site d'étude et par les sous-zones définies administrativement ou géographiquement ou les strates. Cela peut inclure les dernières informations démographiques issues d'un recensement d'une total de la population ou d'un DSS (Demographic Surveillance System) / HDSS (Health and Demographic Surveillance System). Les chiffres sommaires sur la population et les facteurs de croissance de la population, s'ils sont disponibles, ainsi que les outils d'échantillonnage en accès libre couplés à des sources de données sur la population, comme les données sur la population quadrillée basées sur la densité, peuvent également être utilisés (20-24).

Toutes les strates par site d'étude seront incluses. Un ménage tel que défini pour l'étude HPAfrica constitue l'unité d'échantillonnage primaire (PSU). Le nombre minimum de ménages sélectionnés au hasard sera calculé en utilisant l'équation basée de précision (équation-I) en supposant une population infinie et en considérant un effet de design (DEFF) (25-27), y compris les hypothèses qui l'accompagnent:

Equation-I: Calcul de la taille d'échantillon basé sur la précision par site d'étude pour une population infinie (20-23):

$$n_0 = DEFF \cdot \left[z^2{}_{1-\alpha/2} \cdot \frac{4 \cdot p \cdot (1-p)}{d^2} \right]$$

Hypothèses d'Equation-I

n_0	Nombre minimum total de ménages à interroger dans une zone d'étude en supposant une population infinie
$DEFF$	Effect de design; fixé à 1.5<br>≤1.0=negative correlation of the outcome(s) of interest between household members; 1.0=no correlation of the outcome(s) of interest between household members; ≥1.0=positive correlation of the outcome(s) of interest between household members
$z_{1-\frac{\alpha}{2}}$	Déviation Standard correspondant à un intervalle de confiance de 95% (1.96 pour alpha de 0.05)
d	Précision (erreur acceptable); estimation de point; fixé à 0.2
p	Proportion de la population de l'étude devant se rendre dans un établissement de soins de santé de recrutement pour des conditions associées à de la fièvre et d'autres signes et symptômes (proportion capturée); fixé à 0.2
$1-p$	Proportion de la population de l'étude ne devant se rendre dans un établissement de soins de santé de recrutement pour des conditions associées à de la fièvre et d'autres signes et symptômes (proportion non capturée)

L'effet de design est défini comme ~~est~~ un facteur d'ajustement pour le regroupement naturel des comportements de santé comme principal résultat d'intérêt parmi les ~~au sein des~~ membres du ménage. Il rend compte d'une plus grande variance statistique et, par conséquent, d'une plus faible précision par rapport à l'échantillonnage aléatoire simple (25, 29, 32, 33). Pour HPAfrica, le DEFF peut être fixé de manière conservatrice à 1,5 sur la base d'une estimation de 1,42 résultant d'une taille moyenne de

For peer review only - http://bmjopen.bmj.com/site/about/guidelines.xhtml

ménage de sept et d'un c'efficient de corrélation intracluster (ICC) de 0,07 provenant de l'étude TSAP (15, 30, 31, 34). Une proportion p de 0,2 pourra être considérée si aucune autre estimation n'est disponible ou p peut être basé sur des informations plus précises disponibles par site ou p pourra être basé ou sur les expériences au cours de l'étude TSAP (Tableau 2): Madagascar, Isotry : p=0,01, Burkina Faso, Polesgo: p=0,9 (3526). Combiner tous les hypothèses dans l'équation-I va avoir pour résultat un nombre global minimum (n_0) de 92 ménages à interroger par à chaque site.

En outre, n_0 pourra être pris en compte pour la répartition par âge et par sexe de chaque population de l'étude représentée par q $\hat{p}$, la proportion minimum supposée de stratification par groupe d'âge (<5 ans, ≥5 à <15 ans, ≥15 ans). Le facteur q $\hat{p}$ pourra être défini de façon arbitraire à 0,2 ou être basé sur les expériences précédentes: Éthopie, Butajira : q $\hat{p}$=0,09, Burkina Faso, Polesgo : q $\hat{p}$=0,23 (15). Appliquer q $\hat{p}$ à l'Equation-I pour une distribution binomiale, y compris ses postulats connexes, aura pour résultat le nombre minimum de ménages à interroger ($\overline{n_0}$) de 461 stratifié selon l'âge (Equation-II):

Equation-II: Calcul de la taille d'échantillon basé sur la précision par site d'étude pour une population infinie avec stratification selon l'âge et population-poids par sous-zone ou strate:

$$\overline{n_0} = \left(DEFF \cdot \left[z^2_{1-\frac{\alpha}{2}} \cdot \frac{4 \cdot p \cdot (1-p)}{d^2} \right]\right)/q \qquad \overline{n_{0w}} = \overline{n_0} \cdot (n/N)$$

Hypothèses d'Equation-II

$\overline{n_0}$	Nombre total minimum de ménages à interroger dans une zone d'étude en supposant une population infinie et une stratification selon l'âge
$\overline{n_{0w}}$	Minimum number of households to be interviewed by subarea or stratum
n/N	Population size of subarea or stratum divided by the population size of the total study area
$DEFF$	Effect de design; fixé à 1.5
$z_{1-\frac{\alpha}{2}}$	Déviation Standard correspondant à un intervalle de confiance de 95% (1.96 pour alpha de 0.05)
d	Précision (erreur acceptable); estimation de point; fixé à 0.2
p	Proportion de la population de l'étude devant se rendre dans un établissement de soins de santé de recrutement pour des conditions associées à de la fièvre et d'autres signes et symptômes (proportion capturée); fixé à 0.2
$1-p$	Proportion de la population de l'étude ne devant se rendre dans un établissement de soins de santé de recrutement pour des conditions associées à de la fièvre et d'autres signes et symptômes (proportion non capturée)
$q\hat{p}$	Proportion minimum de stratification par groupe d'âge, définie à 0,2

Les comportements de santé seront évalués deux fois dans les mêmes ménages si possible ou dans des ménages alternatifs, au cas où la population de l'étude serait instable en raison de la migration et / ou de taux de natalité / mortalité élevés. Des ménages supplémentaires, supposés être de 20% en moyenne (Tableau 2), peuvent être ajoutés pour tenir compte de la possible perte de suivi entre les deux évaluations. Eventuellement, l'échantillonnage stratifié pondéré en fonction de la population avec probabilité proportionnelle à la taille de la population (PPPS) pour tenir compte de la probabilité de sélection inégale aux distributions de population hétérogènes entre les strates (poids de la population (n/N) par strate) sera appliqué à $\overline{n_0}$ de 553 sera divisé par le nombre de sous-zones et pondéré proportionnellement à la taille de la population en appliquant un échantillonnage stratifié selon le poids (36).

Echantillonnage avec et sans cadre d'échantillonnage de population

Si une base d'échantillonnage de la population ou une liste de ménages compréhensif et à jour existe (par DSS / HDSS ou recensement disponible), une sélection aléatoire informatisée des ménages en tant

For peer review only - http://bmjopen.bmj.com/site/about/guidelines.xhtml

que PSU sera effectuée en utilisant SAS (Système d'analyse statistique, version 9.4, SAS Institute, Cary NC) en appliquant le PPPS stratifié en fonction de la population une sélection aléatoire simple en série sans remplacement. Les ménages sélectionnés avec des identifiants sont visualisés en utilisant des DDS / HDSS ou des outils de recensement. En cas de refus, d'absence d'un répondant ou de localisation d'un ménage abandonné, les enquêteurs sont formés pour visiter un ménage alternatif le plus proche du la côte droit ou gauche d'un ménage d'origine. Cela suit le principe de la proximité la plus proche comme vu dans la recherche précédente (37-40). Pour limiter, en outre, les probabilités de sélection inégale, donc les probabilités de sélection par les enquêteurs, nous présélectionnons un nombre arbitraire de cinq ménages alternatifs d'échantillonnage de population qui doivent être plus proches du ménage d'original plutôt que de demander par exemple une sélection en base de loterie ou une sélection dans le sens des aiguilles d'une montre (36, 37, 40). En outre, la notation de l'un (=le plus proche) à cinq (=le plus éloigné) est appliquée aux ménages alternatifs et les enquêteurs sont invités à suivre strictement la séquence de notation au cours de la procédure de sélection du ménage. La conformité des enquêteurs avec les règles de sélection des ménages originaux et alternatifs sera vérifiée quotidiennement. Les écarts seront étudiés et une formation complémentaire sera fournie aux enquêteurs au besoin.

S'il n'existe pas de cadre d'échantillonnage de la population, nous appliquerons une technique d'échantillonnage spatial stratifié PPPS pondérée selon la population (36). Guidés par des recherches antérieures, des points spatiaux aléatoires sont générés à l'aide de l'outil de génération de points aléatoires ArcGIS (Aeronautical Reconnaissance Coverage Geographic Information System, Redlands, CA, USA, version 10.2) (21, 23, 36, 39, 41-46). L'outil place de la manière façon aléatoire des nombres spécifiés de points spécifiés dans les entités d'un polygone correspondant à une sous-zone administrative ou à une strate. Les coordonnées X et Y définissent un point jusqu'à ce que la taille d'échantillon calculée par strate soit atteinte. Les points aléatoires générés par ArcGIS sont convertis en un fichier image au format KML (Keyhole Markup Language) ou KMZ (Keyhole Markup landguage Zipped) et importés dans Google Earth Pro® (Google Inc., Mountain View, CA, États-Unis, version 6.2.2.6613). Un identifiant numérique unique et des coordonnées géographiques horizontales sont affectés à chaque point. L'emplacement de chaque point est vérifié sur une imagerie satellite Google Earth Pro® mise à jour. L'annexe 4 illustre notre approche en utilisant des exemples du Ghana et de Madagascar. Les points spatiaux géographiques étiquetés avec des identifiants sont représentés sur des impressions de la taille d'une affiche de (60 x 60 à 60 x 90 cm) d'images satellite Google Earth Pro avec une haute résolution jusqu'au niveau de la structure ou alternativement en utilisant une application de source ouverte pour la navigation et la cartographie automatiques hors ligne / en ligne pour localiser les points sélectionnés sur le terrain. Ces cartes satellites aideront à localiser précisément les points sélectionnés sur le terrain. Des récepteurs GPS (Global Positioning System) portables (Garmin-eTrex, Garmin Ltd., Lenexa, KS, USA) aident à vérifier les emplacements. Les récepteurs seront positionnés près d'un point localisé, dans une position statique et en plein air pour assurer une lecture sans obstacle. Les enquêteurs évaluent les points spatiaux de la présence d'un ménage en tant que PSU (24). En cas de refus, d'absence du répondant ou de localisation d'un point non résidentiel, les enquêteurs sont formés pour visiter un point spatialrésidentiel alternatif présélectionné le plus proche de la droite ou de la gauche d'un point original et l'évaluer pour la présence d'un ménagesera visité (37-39, 47). Pour limiter les chances de probabilité de sélection inégale, nous présélectionnons au moins cinq points spatiaux alternatifs à proximité immédiate du point d'origine qui représentent une structure de taille appropriée et de forme rectangulaire ou carrée à partir de l'imagerie satellite Google Earth Pro outil de mesure de distance. Commue pour les sites ayant un cadre d'échantillonnage de la population, la notation d'un à cinq est appliquée aux points spatiaux alternatifs et les enquêteurs doivent suivre la séquence de notation. La conformité des enquêteurs avec les règles de sélection des ménages originaux et alternatifs sera vérifiée quotidiennement. Les écarts seront étudiés et une formation complémentaire sera fournie aux enquêteurs au besoin. Dans le cas où un point spatial est placé dans l'équidistance de deux structures (ou plus), l'intervieweur saute le point spatial original et choisit un point spatial alternatif présélectionné suivant la séquence de notation. Dans le cas où deux points spatiaux originaux (ou plus) sont placés sur

For peer review only - http://bmjopen.bmj.com/site/about/guidelines.xhtml

la même structure, l'intervieweur évalue la structure de la présence d'un ménage et choisit un second point spatial alternatif (ou plus) présélectionné après la séquence de notation.

Indépendamment de la présence ou de l'absence d'un cadre d'échantillonnage de la population, un ménage unifamilial à un étage sera directement contacté pour participer à l'étude. Cependant, un ménage seulement est inscrit dans une structure multifamiliale à un seul étage selon les procédures suivants : l'enquêteur entre dans la structure et évalue le nombre total de ménage : le premier ménage du côte droit / gauche (selon la construction intérieure) de la structure est approché ; si le premier ménage ne participe pas, le second ménage du côte droit / gauche de la même structure est approché. L'enquêteur continue jusqu'à ce qu'un ménage par structure soit inscrit. De même, un ménage seulement est inscrit dans une structure multifamiliale à plusieurs étages. Après avoir évalué le nombre total de ménage présents, l'enquêteur se rapproche du premier ménage ne participe pas, le deuxième ménage le plus proche de l'entrée sur le même étage / suivant (en fonction de la construction intérieure) est approché ; l'enquêteur continue jusqu'à ce qu'un ménage par structure soit inscrit. L'enquêteur choisit un point spatial alternatif présélectionné après la séquence de notation au cas où aucun ménage ne peut être inscrit.

Fréquence de la collecte de données
Les points résidentiels seront visités deux fois pour des entrevues au cours du programme SETA pour prendre en compte un possible impact lié à la saison, sur le comportement sanitaire (48-52). Les enquêtes auront lieu à différentes saisons - une à la fin de la saison sèche ou au moins un mois après son début et une vers la fin de la saison des pluies ou au moins un mois après son début (Tableau 3). Actuellement, l'étude HPAfrica est en cours au Burkina Faso, au Ghana et à Madagascar et devrait se poursuivre dans les autres pays au cours des prochains mois.

Critères d'inclusion et d'exclusion
Les membres du ménage de tout âge et des deux sexes qui vivent dans la zone d'étude au jour de l'interview seront éligibles pour l'inclusion. Un ménage sera exclu si le répondant désigne refuse de participer ou n'est pas disponible après trois visites consécutives. Tous les visiteurs et individus dont le lieu de résidence n'est pas connu ou est en-dehors de la zone d'étude seront exclus.

Pour raison de cette enquête, un ménage est défini comme une personne ou un groupe de personnes apparentées ou non apparentées vivant dans la même unité d'habitation, reconnaissant un individu adulte comme chef du ménage, partageant les mêmes installations domestiques, et subvenant indépendamment à leurs besoins en termes de nourriture et des autres éléments essentiels pour vivre (35).

L'interview aura lieu exclusivement avec le répondant, qui est un membre adulte du ménage à l'âge de majorité légale spécifique au pays. Cette personne peut être identifiée comme décideur par les membres du même ménage et sert de substitut pour un ménage entier (15). D'autres définitions pertinentes sont expliquées subséquemment.

Collecte de données
Les intervieweurs de site recevront une formation sur la localisation des points géographiques, l'identification des répondants, les procédures de consentement éclairé en mettant l'accent sur la participation volontaire, et sur le déploiement de formulaires d'étude standardisés et pré-testés avant le lancement de HPAfrica.

Les formulaires de consentement éclairé et d'étude seront traduits dans la langue officielle du pays par deux traducteurs bilingue indépendants parlant couramment l'anglais et les locuteurs natifs de la langue cible qui connaissent le concept et la terminologie des formulaires.~~et retraduits en anglais pour prendre en compte des erreurs de traduction et apporter des corrections, le cas échéant.~~ Les deux traductions seront comparées pour les divergences avec les traducteurs et un coordinateur, et un consensus sera

For peer review only - http://bmjopen.bmj.com/site/about/guidelines.xhtml

recherché. La traduction avancée synthétisée sera traduite et traduite de la langue cible vers l'anglais par deux traducteurs indépendants, aveugles aux formes originales. Un consensus sur les écarts de traduction et les formulaires originaux seront recherchés avec les traducteurs et un coordinateur. Les formes traduites feront l'objet d'un test pilote parmi un échantillon de commodité des ménages de la population cible avant leur finalisation pour assurer la compréhensibilité interculturelle et l'équivalence sémantique, idiomatique, expérientielle et conceptuelle. Un rapport de traduction sera préparé (53, 54).

Gestion de données

La gestion des données dépendra du mode de collecte, qui sera principalement électronique plutôt que sur support papier. Les expériences passées ont montré les inconvénients suivants de la collecte de données sur papier: une forte probabilité d'erreurs lors du remplissage des formulaires d'étude, et le besoin de numérisation des données, qui est une étape supplémentaire, laborieuse, qui consomme du temps et sujette aux erreurs. La capture des données électroniques ~~de données~~ à l'aide d'une application pour un appareil mobile, comme un smartphone ou une tablette, réduit ces facteurs limitants à la qualité des données.

Il peut y avoir une période pilote au cours de laquelle des données sur support papier et sans papier seront collectées avant la mise en œuvre complète de la collecte de données électroniques. Ce dernier utilise l'application Android « HPA Collect » (Google Android 5.0.1 9API 23, disponible à l'adresse https://play.google.com/store/apps/details?id=anint.ivi.hpa) et la plate-forme « HPA Web » (CSS, JavaScript et JSP, disponible sur http://hpa.ivi.int/), qui sont ~~tous deux~~ accompagnés d'un dictionnaire de variables contenant les codes, la signification et les propriétés des variables au moins en anglais et en français. Les deux, « HPA Collect » et « HPA Web », seront conçus spécifiquement pour les besoins de cette étude. L'expertise d'IVI en ingénierie informatique permet la construction fondamentale, la configuration et le développement des composants nécessaires. Différent de nombreux programmes de source ouverte, une fonction d'installation serveur permet le stockage de toutes les données originales collectées sur le serveur institutionnel en plus des fonctionnalités supplémentaires pour la vérification des erreurs profondes, les relations logiques entre variables et formulaires, les fonctions de recherche et l'analyse des données collectées. Une connectivité Internet sans fil stable utilisant des navigateurs par défaut est nécessaire pour assurer une synchronisation simultanée, attribuable, originale et précise des données entre « HPA Collect » et « HPA Web ». L'accès à « HPA Collect » et « HPA Web » est protégé par un mot de passe. L'annexe 5 affiche le diagramme du système, qui a les caractéristiques suivantes:

Structure ou fonctionnalités de « HPA Collect »

- **Gestion des utilisateurs:** Une interface utilisateur simple fournit différents privilèges aux utilisateurs pour entrer, modifier (sauf pour les étiquettes d'étude) et exporter ou transférer des données. Il établit une piste d'audit qui enregistre lorsque les utilisateurs entrent des données dans le serveur.
- **Saisie de données:** Les données sont entrées directement dans le smartphone ou la tablette. Les champs de saisie limitent les réponses possibles à des réponses plausibles uniquement en offrant différentes méthodes de saisie:
  - Modifier le texte: texte libre
  - Groupe radio / bouton Multi-clic: choix unique (radio group/Multi click button)
  - Case à cocher: choix multiple
  - Sélecteur de date et d'heure: date et heure d'entrée
  - Spinner: bouton déroulant

  Des fonctionnalités supplémentaires permettent à l'enquêteur de gagner du temps en transférant les données collectées plus tôt aux champs de données requis à un stade ultérieur de l'interview en effectuant des vérifications de vraisemblance pour divers champs de saisie de données et en reconnaissant facilement les modèles de saut. Les vérifications d'erreurs, les notifications de données manquantes, les erreurs de code et les erreurs logiques sont également incluses.

For peer review only - http://bmjopen.bmj.com/site/about/guidelines.xhtml

- **Vue des données:** Les données brutes entrées à l'origine peuvent être vues dans le même format lisible par l'homme et la machine, à savoir le format JSON, étant donné qu'elles seront transférées vers un serveur de base de données (Windows, MySQL). Des fonctions supplémentaires telles que «effacer» pour effacer tous les enregistrements, «réinitialiser» pour revenir à la page de vue'entrée de données principale, et «rechercher» pour rechercher une étiquette d'étude sont disponibles.
- **Exporter :** Toutes les données entrées seront envoyées au serveur IVI via une connexion Internet sans fil. Chaque enregistrement de formulaire ayant une étiquette d'étude unique du ménage inscrit est transformé en une requête d'insertion SQL et inséré sur le serveur uniquement s'il n'y a pas d'étiquette d'étude en double trouvée. L'insertion de données sera ignorée dans le cas d'étiquettes d'étude dupliquées.
- **Exporter ers local :** Tous les données entrées seront sauvegardées trois fois dans un dossier de stockage public de l'appareil mobile au format JSON et txt. Les fichiers txt contiennent des données de chaque formulaire individuel. Une version de fichiers JSON contient des données par chaque formulaire individuel et une deuxième version de fichiers JSON contient toutes les données d'un ménage inscrit. Les données au format JSON peuvent être extraites par USB ou connexion Internet sans fil.
- **Rapport :** Cette fonctionnalité permet à un utilisateur de signaler toute requête à l'administrateur principal de « HPA Collect » à IVI par e-mail our par une application tierce.
- **Paramètres:** la possibilité de changer la langue du système entre l'anglais et le français est fournie.
- **À propos de:** La version actuellement installée de «HPA Collect» est affichée.

Structure ou caractéristiques de « HPA Web »

- **Accueil:** une introduction à la plateforme est donnée et un tutoriel en français et en anglais sera accessible.
- **Aperçu du Formulaire:** Les formulaires d'étude sont affichés séparément montrant chaque question et les données d'entrée correspondantes en tant que variables définies.
- **Modifier le formulaire:** cette fonction permet la correction des données téléchargées. Il est uniquement accessible aux gestionnaires de données de chaque pays participant et à IVI.
- **Cherche:** Les étiquettes d'étude spécifiques données à chaque formulaire d'étude peuvent être filtrées.
- **Dictionnaire des variables:** Le dictionnaire variable peut être téléchargé.
- **Exporter:** Les données peuvent être converties à partir du serveur dans un fichier Excel transférable.
- **Contactez-nous:** Une correspondance rapide et facile entre les utilisateurs et le gestionnaire de données à IVI est donnée.

Les données seront examinées quotidiennement par le personnel clé de l'étude et vérifiées en termes de consistance et d'exactitude avant l'analyse des données. La qualité des données peut être vérifiée en sélectionnant un sous-ensemble de 5% pour la validation par rapport aux données originales sur support papier, si possible. Les noms des membres du ménage ne seront pas liés aux formulaires d'étude de HPAfrica et ne seront pas enregistrés dans la base de données afin d'assurer la confidentialité. L'accès à la base de données sera limité au personnel d'étude autorisé uniquement et les données seront conservées dans un endroit sécurisé et protégé. Des fichiers de sauvegarde provisoires périodiques et au moins trois fichiers de sauvegarde définitifs de la base de données stockés dans un endroit sécurisé et verrouillé éviteront la perte de données et garantiront la sécurité des données. Toutes les données seront conservées pendant au moins cinq ans.

L'analyse des données

Les poids d'échantillonnage combinés seront calculés pour tenir compte des différences possibles dans les probabilités de sélection et pour accroître la précision des estimations de l'étude (22, 55). Les poids sont générés dans une approche à plusieurs niveaux pour prendre en compte des probabilités de sélection de

For peer review only - http://bmjopen.bmj.com/site/about/guidelines.xhtml

par exemple les ménages, les poids inverses au sein des ménages (c'est-à-dire la structure à plusieurs étages à plusieurs familles, la structure à plusieurs étages multifamiliale) et les poids inverses parmi les membres du ménage participant à l'étude HPAfrica (21, 39). Les outils de source ouverte, les données de recensement et les données générées par HPAfrica telles que la taille de la population, le nombre de ménages ou de structures, le nombre de ménages dans les structures multifamiliales / multi-étages et la taille des ménages par strate seront utilisés pour calculer poids (56, 57).

Des analyses descriptives telles que les fréquences absolues et relatives, les médianes ou les moyennes arithmétiques seront effectuées pour construire une description contemporaine de la population de base. Les analyses de base comprendront le calcul des proportions stratifiées selon l'âge et le sexe, des comportements sanitaires *génériques* et *réels*, y compris l'utilisation des soins de santé, pour divers signes et symptômes ou combinaisons de signes et de symptômes. Ces analyses permettront une classification graduelle basée sur la sévérité de la maladie, incluant un intervalle de confiance de 95%. Le comportement de santé sera ajusté pour un effet de regroupement naturel présumé parmi les membres du ménage. Des données stratifiées les plus à jour sur la population par site et les données démographiques dérivées de HPAfrica seront utilisées pour générer les proportions pour l'ajustement des calculs d'incidence.

Les informations socio-économiques collectées seront utilisées pour construire des indices de richesse des populations étudiées en appliquant des analyses factorielles basées sur la méthode des composantes principales (38). De plus, des analyses de régression bi et multivariée seront effectuées pour identifier les facteurs de confusion potentiels ou la modification des effets en utilisant les scores factoriels.

Les analyses de régression bi et multivariée seront effectuées en utilisant les scores factoriels pour l'évaluation des variables suivantes:

- Données socioéconomiques telles que l'éducation, le revenu, l'assurance maladie, le logement et les biens des ménages
- Hygiène et assainissement tels que l'accès aux toilettes, les sources d'eau, la manipulation des aliments, l'élimination des déchets et les contacts avec les animaux
- Comportement sanitaire: *réel* (stratifié selon l'âge et le sexe, gravité, fréquence), *générique* (stratifié selon l'âge et le sexe, gravité, classement par choix)
- Modalités de déplacement vers les établissements de santé, stratifiées selon l'âge
- Saisonnalité liée à l'hygiène et à l'assainissement, comportement sanitaire, modalités de déplacement
- Facteurs culturels / religieux / ethnographiques, stratifiés selon l'âge
- Statut de vaccination parmi les enfants ≤ 6 ans (18, 19)
- Connaissance et perception des maladies courantes

Considérations éthiques
Il n'y a pas d'avantages directs pour les ménages inscrits et leurs membres. Les avantages indirects pour la population générale sur les sites seront les informations mises à jour sur le fardeau des maladies transmissibles, y compris les facteurs influençant la transmission de la maladie et l'infection. L'étude HPAfrica génère des évidences pour aider les décideurs à introduire des mesures appropriées pour la prévention et le contrôle des maladies.

Ce protocole a fait l'objet d'examens par le Comité de Révision Institutionnelle d'IVI et des comités locaux spécifiques aux sites, comme indiqué dans la section «Références éthiques». Pendant et après l'étude HPAfrica, toutes les données des sujets inscrits seront gardés avec une stricte confidentialité et ne seront pas divulguées à une tierce partie par aucun membre de l'équipe de recherche. La protection par mot de passe des appareils et de la base de données est utilisée pour une stricte confidentialité. Toutes les données sur support papier (formulaires de consentement) seront conservées dans un endroit sécurisé et verrouillé. Les informations confidentielles stockées sur des ordinateurs et des sources de données sur

For peer review only - http://bmjopen.bmj.com/site/about/guidelines.xhtml

support papier seront seulement mises à la disposition des co-chercheurs et du personnel d'IVI directement impliqués dans les activités d'étude de HPAfrica.

Abbréviations
JSON: JavaScript Objective Notation: CSS: Cascading Style Sheets: JSP: Java Server Page; SQL: Structured Query Language

For peer review only

For peer review only - http://bmjopen.bmj.com/site/about/guidelines.xhtml

Définitions/Terminologie

**A**	
Automédication	Fait de se médicamenter soi-même ou de traiter sa propre maladie ou son état sans supervision médicale ou ordonnance.
**B**	
**C**	
Choléra	Maladie infectieuse de l'intestin grêle qui peut être mortelle causée par la bactérie *Vibrio cholerae* ; elle se propage par voie fécale-orale par l'ingestion d'eau et d'aliments contaminés et provoque fièvre, diarrhée aqueuse sévère, vomissements et déshydratation.
Confusion/Vertiges/Perte de connaissance	L'état de désorientation ou d'absence de clarté d'esprit ; l'état anormal de réponse aux stimuli dans l'environnement; faible, wooziness, faiblesse, instabilité ou tendance à tomber; l'interruption de la conscience de soi et de son environnement.
Convulsion	Mouvement soudain, violent et irrégulier d'un membre/d'une partie du corps ou de tout le corps causé par une contraction involontaire des muscles.
Coqueluche (Pertussis)	La coqueluche est une maladie bactérienne très contagieuse. Les symptômes d'un froid commun comme nez qui coule, fièvre et toux sont suivis par des semaines de toux sévère et un son aigu de la coqueluche ou haletant quand une personne malade inspire; toux sévère peut être suivie de vomissements, rupture des côtes ou fatigue extrême. Les très jeunes et les personnes âgées infectées peuvent avoir peu ou pas de toux, ou peuvent avoir des périodes de respiration interrompue pendant lesquelles elles ne respirent pas. La coqueluche est transmise par la bactérie *Bordetella pertussis* par toux et éternuements d'une personne infectée.
**D**	
Déshydration	Une condition de perte excessive d'eau/d'hydratation par le corps.
Diarrhée	Une condition d'évacuer les matières fécales du corps très fréquemment et sous forme plutôt liquide que solide.
Difficultés respiratoires/ Respiration sévère	Une condition de la respiration anormale accompagnée d'une respiration sifflante, d'une douleur thoracique, d'un tirage thoracique, d'un stridor, d'un gonflement de la poitrine ou d'une toux par exemple.
Diphthérie	Maladie hautement contagieuse, parfois mortelle, causée par la bactérie *Corynebacterium diphtheriae*. Elle se transmet de personne à personne, par l'air ou les objets contaminés et affecte les voies respiratoires supérieures. Les symptômes courants sont maux de gorge, gonflement des glandes/ ganglions lymphatiques, malaise, fièvre/frissons, substance membraneuse/matière grise et épaisse qui se forme dans la gorge/sur les amygdales et gêne/accélère la respiration (toux rauque) et gêne pour avaler. Une forme de diphtérie qui implique la peau, les yeux et les organes génitaux est également signalée et se caractérise par une peau douloureuse, rouge et enflée.
Douleur abdominale/ Douleur intestinale	Douleur (aiguë/courte durée ou chronique/longue durée) qui survient entre la poitrine et l'aine (souvent désignée comme la région de l'estomac/du ventre)
Douleur intestinale/Douleur abdominale	Douleur (aiguë/courte durée ou chronique/longue durée) qui survient entre la poitrine et l'aine (souvent désignée comme la région de l'estomac/du ventre)
Douleur/Maux	État caractérisé par une douleur sourde continue/prolongée dans une partie du corps ; la sensation/souffrance/inconfort physique causée par

For peer review only

	une maladie/une blessure/quelque chose qui fait mal au corps.
**E**	
Éducation formelle	Modèle d'éducation systématique et organisée structuré et administré selon un ensemble de lois et de normes ; les institutions d'éducation formelle sont organisées de façon administrative, physique et pédagogique et exigent des étudiants une assiduité minimum ; il implique des évaluations intermédiaires et finales afin d'accéder au niveau d'apprentissage suivant ; il délivre des certificats et des diplômes conformément à un ensemble de règles strictes.
Essoufflement/ Respiration rapide	Fait de respirer anormalement rapidement et profondément ; à une fréquence de >20 respirations par minute pour les adultes, >30 respirations par minute pour les enfants et >45 respirations par minute par les bébés.
Établissement de santé	Lieux ou institutions où des soins de santé sont prodigués ; dont les hôpitaux, les cliniques, les centres de santé, les postes de santé et les centres de soins spécialisé.
Éternuement	Fait d'expulser soudainement l'air par le nez et la bouche avec un bruit important caractéristique.
**F**	
Faiblesse/Malaise/Fatigue	État de se sentir fatigué/épuisé/à bout de forces ; sentiment général d'inconfort/malaise/de ne pas être en bonne santé ou heureux.
Fatigue/Malaise/ Faiblesse	État de se sentir fatigué/épuisé/à bout de forces ; sentiment général d'inconfort/malaise/de ne pas être en bonne santé ou heureux.
Fièvre	Température corporelle anormalement élevée (habituellement au-dessus de 38°C).
Fièvre <3 jours	Température corporelle anormalement élevée (habituellement au-dessus de 38°C) qui dure moins de 3 jours et apparaît en continu.
Fièvre >3 jours	Température corporelle anormalement élevée (habituellement au-dessus de 38°C) qui dure plus de 3 jours et apparaît en continu.
Fièvre jaune	Maladie infectieuse aiguë, pathogène pour l'homme et virale caractérisée par l'apparition soudaine de symptômes comme fièvre, frissons, perte d'appétit, nausée, douleurs musculaires et maux de tête qui disparaissent généralement en quelques jours ou sont suivis de symptômes plus graves comme jaunisse, fièvre forte et hémorragie. La maladie est causée par un virus du genre Flavivirus et est transmise par la piqûre d'un moustique infecté.
Fièvre typhoïde	Maladie infectieuse bactérienne potentiellement mortelle causée par *Salmonella typhi* ; elle se transmet par la voie fécale-orale par les aliments et l'eau contaminés ou de personne à personne. Les symptômes courants sont forte fièvre, maux de tête, douleur abdominale, constipation/diarrhée et éruption cutanée. Des complications graves (perforation de l'intestin) sont rapportées.
Frissons	Sensation de froid qui peut être accompagnée de frissons/tremblements du corps et de pâleur de la peau
**G**	
Gonflement/Œdème	État d'accumulation excessive de liquide aqueux/gonflement des cavités ou des tissus du corps ; couramment constaté dans les mains/bras/pieds/jambes ; augmentation du volume de tissu.
Grippe	Maladie infectieuse hautement contagieuse des voies respiratoires causée par le virus de la grippe ; elle se propage de personne à personne par les particules expulsées par la toux/les éternuements/la parole et les objets contaminés. Les symptômes courants sont fièvre,

For peer review only - http://bmjopen.bmj.com/site/about/guidelines.xhtml

	douleurs dans les muscles/bras/jambes, frissons/sueur, maux de tête, fatigue/faiblesse, nez qui coule/congestion nasale, éternuements, maux de gorge et toux sèche et persistante.
Guérisseur traditionnel	Personne qui cherche à maintenir la santé aussi bien qu'à prévenir, diagnostiquer et améliorer ou traiter les maladies en utilisant un ensemble de connaissances, compétences et pratiques basées sur des théories, des croyances et des expériences.
**H**	
Haemophilus influenzae de type B	Bactérie qui provoque une série de maladies et se propage par les particules expulsées par la toux ou les éternuements, et surviennent surtout chez l'enfant de moins de 5 ans ; les symptômes courants sont fièvre, maux de tête et raideur dans le cou jusqu'à l'état septique. Elle peut causer otites, cellulites (infection des tissus mous), arthrite, infections des voies respiratoires supérieures, pneumonies, méningites (avec dommages potentiels au cerveau) et épiglottites (avec obstruction des voies aériennes).
Hémorragie (interne/externe)/ Perte de sang	État caractérisé par une perte de sang du système vasculaire, soit de façon interne à l'intérieur du corps (hémorragie interne) soit de façon externe par un orifice naturel ou une lésion de la peau (hémorragie externe) ; sang qui s'échappe d'un vaisseau sanguin endommagé.
Hépatite B	Maladie infectieuse causée par le virus de l'hépatite B (VHB) qui affecte le foie (infection hépatique aiguë/chronique ; peut entraîner une insuffisance hépatique/un cancer/une cirrhose). Le virus se transmet de personne à personne par le sang/le sperme/les autres fluides corporels. Les symptômes courants sont fièvre, malaise/faiblesse/fatigue, perte d'appétit, nausée/vomissements, gêne/douleur abdominale, urines foncées, douleurs articulaires et jaunisse.
Hépatite E	Maladie infectieuse causée par le virus de l'hépatite E (VHE) qui affecte le foie (infection hépatique aiguë uniquement ; peut entraîner une insuffisance hépatique aiguë). Le virus se propage par les voies fécales-orales par l'ingestion d'eau ou d'aliments contaminés, les animaux contaminés (de façon zoonotique) et par le sang. Les symptômes courants sont fièvre, malaise, perte d'appétit, diarrhée, nausée, gêne abdominale, urines foncées, douleurs articulaires et jaunisse.
**I**	
**J**	
Jaunisse	Maladie qui fait jaunir la peau/le blanc des yeux.
**K/L**	
**M**	
Malaise/Fatigue/ Faiblesse	État de se sentir fatigué/épuisé/à bout de forces ; sentiment général d'inconfort/malaise/de ne pas être en bonne santé ou heureux.
Maux de tête	Douleur continue dans une région de la tête.
Maux/Douleur	État caractérisé par une douleur sourde continue/prolongée dans une partie du corps ; la sensation/souffrance/inconfort physique causée par une maladie/une blessure/quelque chose qui fait mal au corps.
Médecin	Une personne compétente/formée aux soins ; une personne éduquée, cliniquement expérimentée et autorisée à pratiquer la médecine.
Ménage	Personne ou groupe de personnes apparentées ou non qui vivent ensemble dans la même unité d'habitation, qui reconnaissent un adulte de sexe masculin ou féminin comme le chef du ménage, qui partagent

For peer review only - http://bmjopen.bmj.com/site/about/guidelines.xhtml

	les mêmes installations domestiques, qu'on considère comme formant une unité, et qui subviennent à leurs besoins en termes de nourriture et des autres éléments essentiels pour vivre. Un ménage peut être situé dans une unité d'habitation unique (bâtiment à un seul étage) ou dans un ensemble de logements collectifs (bâtiment à plusieurs étages).
Méningite	Inflammation virale/bactérienne/fongique infectieuse parfois mortelle des membranes (méninges) qui entourent le cerveau et la moelle épinière ; elle se propage de personne à personne par les particules expulsées par la toux/les éternuements/la parole et les objets contaminés. Les symptômes courants sont forte fièvre, maux de tête sévères, nausée/vomissements, confusion, convulsions ; perte d'appétit et de la soif, fatigue, sensibilité à la lumière, éruption cutanée et raideur au cou.
**N**	
Nausée/Vomissements	Sensation de malaise dans l'estomac avec tendance à vomir ; fait d'éjecter une partie/tout le contenu de l'estomac principalement par la bouche.
Nez qui coule	État résultant de la production par le nez (tissus nasaux et adjacents et vaisseaux sanguins) de mucus/drainage excessif, qui coule du nez pour retirer quelque chose.
**O**	
Œdème/Gonflement	État d'accumulation excessive de liquide aqueux/gonflement des cavités ou des tissus du corps ; couramment constaté dans les mains/bras/pieds/jambes ; augmentation du volume de tissu.
Oreillons	Maladie infectieuse virale causée par le virus des oreillons qui affecte principalement les glandes parotides qui sont situées sous et devant les oreilles ; elle se propage de personne à personne par la salive infectée/les particules de salive infectées expulsées par la toux/les éternuements/la parole et les objets contaminés. Les symptômes courants sont fièvre, gonflement d'une/des deux glandes parotides, maux de tête ; douleurs musculaires, faiblesse/fatigue, perte d'appétit et douleurs en mâchant/avalant ; une complication connue est une possible perte d'audition.
**P**	
Paludisme	Maladie infectieuse parfois mortelle causée par un parasite (*Plasmodium* spp.) transmis principalement par la piqûre de moustiques infectés, en dehors de la transmission mère-enfant et par le sang. Les symptômes courants sont attaques récurrentes de frissons, sueurs, forte fièvre, maux de tête, vomissements et diarrhée.
Personne interrogée	Membre adulte (à l'âge légal de majorité du pays concerné) du ménage qui est un décideur en ce qui concerne les soins de santé/l'utilisation des soins de santé pour le ménage entier à l'intérieur du site d'étude ; cette personne adulte parle ainsi au nom de tous les membres du ménage. Cette personne peut être identifiée par les autres membres du même ménage comme la personne qui est principalement impliquée dans les soins quotidiens des membres du ménage.
Perte de connaissance/ Vertiges/Confusion	L'état de désorientation ou d'absence de clarté d'esprit ; l'état anormal de réponse aux stimuli dans l'environnement; faible, wooziness, faiblesse, instabilité ou tendance à tomber; l'interruption de la conscience de soi et de son environnement.
Perte de poids	Baisse (volontaire/involontaire) du poids du corps.
Perte de sang/	État caractérisé par une perte de sang du système vasculaire, soit de

For peer review only - http://bmjopen.bmj.com/site/about/guidelines.xhtml

Hémorragie	façon interne à l'intérieur du corps (hémorrage interne) soit de façon externe par un orifice naturel ou une lésion de la peau (hémorragie externe) ; sang qui s'échappe d'un vaisseau sanguin endommagé.
Pharmacie	Endroit où seuls des médicaments (ordonnance requise/non requise) sont préparés, conservés, composés et dispensés.
Pneumocoque	Une inflammation infectieuse bactérienne des poumons parfois mortelle qui affecte souvent les personnes immunodéprimées et est causée par la bactérie *Streptococcus pneumoniae* ; elle se propage de personne à personne par les particules expulsées par la toux/les éternuements/la parole et les objets contaminés. Les symptômes courants sont fièvre, frissons, toux (avec mucosités/pus), douleurs thoraciques, difficulté à respirer/essoufflement, fatigue, nausée/vomissements et diarrhée ; des symptômes rares sont septicémie, épanchement pleural et empyème.
Polio	Maladie virale infectieuse, très contagieuse, parfois mortelle, causée par le poliovirus qui cause dans sa forme la plus sévère paralysie et difficultés à respirer ; il se transmet par la voie fécale-orale par ingestion d'eau et d'aliments contaminés et de personne à personne. Les symptômes courants sont fièvre, maux de gorge, maux de tête, vomissements, fatigue, maux de dos, de cou/raideur au cou, faiblesse des muscles/muscles douloureux, méningite, perte de réflexes, membres lourds/pendants.
Occupation	A job, or means of earning a wage/living; often requires the mastery of a complex set of knowledge, tasks, duties and skills acquired through formal education and/or practical experience.
**Q**	
**R**	
Respiration rapide/ Essoufflement	Fait de respirer anormalement rapidement et profondément ; à une fréquence de >20 respirations par minute pour les adultes, >30 respirations par minute pour les enfants et >45 respirations par minute par les bébés.
Respiration sévère/ Difficultés respiratoires	Une condition de la respiration anormale accompagnée d'une respiration sifflante, d'une douleur thoracique, d'un tirage thoracique, d'un stridor, d'un gonflement de la poitrine ou d'une toux par exemple.
Revenus/Salaire	Argent ou autre forme de paiement que quelqu'un reçoit périodiquement/régulièrement en échange de biens ou de services. Provient généralement d'un emploi/d'une profession.
Rotavirus	Infection virale causée par le rotavirus qui cause principalement des diarrhées pour les enfants de moins de 5 ans ; elle se propage par la voie fécale-orale, de personne à personne et par les objets contaminés. Les symptômes courants sont diarrhée aqueuse, fièvre, vomissements, douleurs abdominales et déshydratation.
Rougeole	Maladie infectieuse parfois mortelle des voies respiratoire (nez/gorge) causée par un virus qui affecte principalement les enfants de moins de 5 ans ; elle se propage de personne à personne par les particules expulsées par la toux/les éternuements/la parole et les objets contaminés. Les symptômes courants sont fièvre, toux sèche, nez qui coule, inflammation des yeux, maux de gorge, larges plaques sur la peau et points blancs dans la bouche et sur les joues.
Rubéole	Maladie infectieuse virale contagieuse connue pour son éruption cutanée rouge distinctive qui est causée par le virus de la rubéole ; elle se transmet de personne à personne (toux/éternuements/parole), de la mère à l'enfant par le sang et les objets contaminés. Les symptômes courants sont fièvre modérée, maux de tête, nez bouché/qui coule,

For peer review only - http://bmjopen.bmj.com/site/about/guidelines.xhtml

	yeux rouges/inflammation des yeux, ganglions lymphatiques gonflés et sensibles à la base du crâne/à l'arrière du cou/derrière les oreilles, douleurs articulaires et éruption cutanée rose qui se déplace du visage au torse, aux bras et aux jambes.
**S**	
Soins de santé - généraux	Soins de santé pour les maladies qui n'ont aucun lien avec l'apparition de maladies associées aux signes et symptômes ou les dates de début de maladies.
Soins de santé - réels	Soins de santé liés l'apparition de maladies associées aux signes et symptômes ou les dates de début de maladies.
**T**	
Témoin	Personne adulte (à l'âge légal de majorité du pays concerné), qui est indépendante de l'étude, qui ne peut pas être indûment influencée par le personnel de l'étude, qui participe au processus de consentement éclairé si le sujet/le représentant légal du sujet est analphabète, et qui lit (lettré) le formulaire de consentement éclairé et toute autre information écrite présentée au sujet.
Tétanos	Une maladie infectieuse caractérisée par des spasmes musculaires qui commencent généralement dans la mâchoire et progresse vers le reste du corps. Les spasmes durent généralement quelques minutes et se produisent fréquemment pendant les 3-4 semaines au début de la maladie, ils peuvent être si graves qu'ils peuvent causer des fractures osseuses ou la mort lorsqu'ils affectent les muscles respiratoires. Elle peut être caractérisée par d'autres symptômes comme fièvre, sueurs, maux de tête, difficultés à avaler, pression artérielle élevée et augmentation du rythme cardiaque. La maladie est causée par une infection par la bactérie *Clostridium tetani* qui se trouve dans sol, saliva, poussière et fumier. La bactérie entre par une lésion cutanée (par exemple, coupure, blessure par ponction) par un objet contaminé. Il produit des toxines qui interfèrent avec les contractions musculaires qui résultent des spasmes typiques.
Toux	Fait d'expulser de façon soudaine/rapide et bruyamment l'air des poumons ; souvent de façon involontaire afin de dégager les voies respiratoires des poumons de fluides/mucus/autres matières
Troubles cardiaques	Tout trouble que affecte le cœur ; maladies qui impliquent des vaisseaux sanguins rétrécis/bloqués qui peuvent entraîner une attaque cardiaque/douleur thoracique/accident vasculaire cérébral ; maladie qui affecte le muscle/les valves/le rythme du cœur.
Troubles de la pression artérielle	Pression artérielle élevée/faible ou pression artérielle qui présente des fluctuations excessives.
Tuberculose	Maladie infectieuse bactérienne (*Mycobacterium tuberculosum*), contagieuse, potentiellement grave qui affecte principalement les poumons en plus des reins et de la colonne vertébrale/du cerveau ; elle se transmet de personne à personne par les particules expulsées par la toux/les éternuements/la parole. Les symptômes courants d'une tuberculose active sont toux (avec/sans sang), douleur thoracique, perte de poids, fatigue, fièvre, sueurs nocturnes, frissons et perte d'appétit.
**U**	
**V**	
Vertiges/Confusion/Perte de connaissance	L'état de désorientation ou d'absence de clarté d'esprit ; l'état anormal de réponse aux stimuli dans l'environnement; faible, wooziness, faiblesse, instabilité ou tendance à tomber; l'interruption de la

For peer review only - http://bmjopen.bmj.com/site/about/guidelines.xhtml

	conscience de soi et de son environnement.
VIH/SIDA	Maladie infectieuse causée par le virus de l'immunodéficience humaine (VIH) qui affecte/endommage le système immunitaire et entraîne le syndrome immunodéficitaire acquis (SIDA), une maladie chronique et potentiellement mortelle ; il se transmet par les liquides corporels infectieux comme le sang/le sperme/les autres fluides corporels. Les symptômes courants d'une infection aiguë/précoce sont fièvre, fatigue, maux de tête, douleurs articulaires, éruption cutanée, maux de gorge, ganglions lymphatiques enflés, diarrhée, perte de poids, candidose orale et zona. La maladie non traitée évolue en SIDA ; les symptômes causés par un système immunitaire gravement endommagé sont sueurs, fièvre récurrente, diarrhée chronique, lésions sur la langue et dans la bouche, fatigue, perte de poids et éruptions cutanées.
Visiteur	Personne (apparentée ou non) qui rend visite à un ménage pour passer du temps avec les membres du ménage ; un visiteur ne vit pas avec les membres du ménage dans la même unité d'habitation, ne partage pas les mêmes installations domestiques, la même nourriture et autres éléments essentiels à la vie que les membres du ménage, et ne constitue pas une unité avec les membres du ménage.
Voisin	Personne (apparentée ou non) vivant dans l'habitation à côté de celle d'un ménage cible ; un voisin ne vit pas avec les membres d'un ménage (à côté) dans la même unité d'habitation, ne partage pas les mêmes installations domestiques, aliments ou autres éléments essentiels à la vie que les membres du ménage (à côté), et ne constitue pas une unité avec les membres du ménage (à côté).
Vomissements/Nausée	Sensation de malaise dans l'estomac avec tendance à vomir ; fait d'éjecter une partie/tout le contenu de l'estomac principalement par la bouche.
**W/X/Y/Z**	

For peer review only - http://bmjopen.bmj.com/site/about/guidelines.xhtml

Formulaires de l'étude

Site ___ ___ ___ Sous-zone ___ ___ ___ Numéro d'habitation ___ ___ ___ ___ Identifiant de l'enquêteur ___ __

FORMULAIRE 1 – COMPTE RENDU DES VISITES AUX MÉNAGES

Instructions pour l'enquêteur

– Compléter « Formulaire 1 – COMPTE RENDU DES VISITES AUX MÉNAGES » pour chaque ménage visité.
– Compléter « Foyer/Personne interrogée/Complété » une fois que toutes les parties de l'enquête ont été complétées ; si toutes les données n'ont pas été recueillies lors de la visite initiale et/ou d'une visite ultérieure (max. 3 visites), programmer une nouvelle visite.
– Tentative 1: Compléter le statut de la visite au ménage et utilisant les choix proposés ; pour « pas à la maison », « à la maison/ne répond pas » et « à la maison/répond/indisponible », programmer une nouvelle visite ; pour « à la maison/répond/refus » indiquez la raison de refus et visitez un ménage de remplacement au plus proche à droite ou à gauche de ce ménage; pour « inexistant/pas d' habitation », passer ce ménage et visiter un ménage de remplacement au plus proche à droite ou à gauche à la place de ce ménage.
– Tentative 2: Compléter le statut de la visite du ménage et utilisant les choix proposés ; pour « pas à la maison », « à la maison/ne répond pas » et « à la maison/répond/indisponible », programmer une nouvelle visite.
– Tentative 3: Compléter le statut de la visite au ménage et utilisant les choix proposés ; pour « pas à la maison », « à la maison/ne répond pas » et « à la maison/répond/indisponible », passer ce ménage et visiter un ménage de remplacement au plus proche à droite ou à gauche à la place de ce ménage.
– « Site » et « sous-zone » doivent être un numéro à trois chiffres, « numéro d'habitation » un numéro à quatre chiffres, et « identifiant de l'enquêteur » un numéro à deux chiffres (utiliser les listes préparées individuellement pour chaque site pour obtenir les codes) ; saisir « date » au format JJ/MM/AAAA et « heure » au format HH:MM et entourer MATIN ou APRÈS-MIDI. «Étiquette de l'étude» doit être une combinaison d'un code «site» à trois chiffres, d'un code «sous-zone» à trois chiffres et d'un code «numéro d'habitation» à quatre chiffres; le « identifiant de l'enquêteur » doit être un numéro à trois chiffres.

**Tentative 1**	Date ____/____/____ (JJ/MM/AA)	Heure _____/_____ MATIN/APRÈS-MIDI	Identifiant de l'enquêteur ___ ___
STATUT		Cocher un	
1=Pas à la maison		O	➡ Planifier nouvelle visite (date/heure)
2=À la maison/ Non-répondant		O	➡ Planifier nouvelle visite (date/heure)
3=À la maison/ répondant /Indisponible		O	➡ Planifier nouvelle visite (date/heure)
4= À la maison/ Répondant /Complété		O	
5= À la maison/ répondant /Refus		O	Notez la/les raison(s) du refus; cochez tout ce que s'applique:<br>O 1=Pas de temps pour répondre aux questions<br>O 2=Ne veulent pas participer/répondre aux questions<br>O 3=Autre ______________________________<br>O 99=Pas de réponse O 98=Ne sait pas<br>➡ Visitez un ménage de remplacement le plus proche de ce ménage du côté droit ou gauche
6=Inexistant/Pas une habitation		O	➡ Visitez un ménage de remplacement le plus proche de ce ménage du côté droit ou gauche

For peer review only - http://bmjopen.bmj.com/site/about/guidelines.xhtml

**Tentative 2**	Date ____/____/____ (JJ/MM/AA)	Heure _____/_____ MATIN/APRÈS-MIDI	Identifiant de l'enquêteur ___ ___
STATUT		Cocher un	
1=Pas à la maison		O	➡ Planifier nouvelle visite (date/heure)
2=À la maison/Ne répond pas		O	➡ Planifier nouvelle visite (date/heure)
3=À la maison/Répond/Indisponible		O	➡ Planifier nouvelle visite (date/heure)
4= À la maison/Répond/Complété		O	
5= À la maison/ répondant /Refus		O	Notez la/les raison(s) du refus; cochez tout ce que s'applique:<br>O 1=Pas de temps pour répondre aux questions<br>O 2=Ne veulent pas participer/répondre aux questions<br><br>O 3=Autre ______________________________<br>O 99=Pas de réponse O 98=Ne sait pas<br><br>➡ Visitez un ménage de remplacement le plus proche de ce ménage du côté droit ou gauche
6=Inexistant/Pas une habitation		O	➡ Visitez un ménage de remplacement le plus proche de ce ménage du côté droit ou gauche

**Tentative 3**	Date ____/____/____ (JJ/MM/AA)	Heure _____/_____ MATIN/APRÈS-MIDI	Identifiant de l'enquêteur ___ ___
STATUT		Cocher un	
1=Pas à la maison		O	➡ Visitez un ménage de remplacement le plus proche de ce ménage du côté droit ou gauche
2=À la maison/Ne répond pas		O	➡ Visitez un ménage de remplacement le plus proche de ce ménage du côté droit ou gauche
3=À la maison/Répond/Indisponible		O	➡ Visitez un ménage de remplacement le plus proche de ce ménage du côté droit ou gauche
4= À la maison/Répond/Complété		O	
5= À la maison/ répondant /Refus		O	Notez la/les raison(s) du refus; cochez tout ce que s'applique:<br>O 1=Pas de temps pour répondre aux questions<br>O 2=Ne veulent pas participer/répondre aux questions<br><br>O 3=Autre ______________________________<br>O 99=Pas de réponse O 98=Ne sait pas<br><br>➡ Visitez un ménage de remplacement le plus proche de ce ménage du côté droit ou gauche
6=Inexistant/Pas une habitation		O	➡ Visitez un ménage de remplacement le plus proche de ce ménage du côté droit ou gauche

For peer review only

Site ___ ___ ___ Sous-zone ___ ___ ___ Numéro d'habitation ___ ___ ___ ___ Identifiant de l'enquêteur ___ __

FORMULAIRE 2 – FORMULAIRE DE CONSENTEMENT

Evaluation des soins de santé à [site SETA and –pays]

Instructions à l'enquêteur

- Lire le "Formulaire 2 – FORMULAIRE DE CONSENTEMENT" pour le répondant/demander au répondant de lire attentivement le "Formulaire 2 – FORMULAIRE DE CONSENTEMENT" s'il préfère.
- Le répondant tel que défini dans cette enquête est un adulte (ayant l'âge de la majorité spécifique au pays) membre du ménage et responsable des décisions journalières concernant la santé des membres du ménage; il ne doit pas par exemple être un voisin ou un visiteur.
- S'assurer que le répondant a pleinement compris l'objectif, les procédures, les risques et les bénéfices de cette étude avant d'obtenir son consentement à participer.
- Le répondant doit signer le "Formulaire 2 – FORMULAIRE DE CONSENTEMENT"; si le répondant est incapable de signer/analphabète, une empreinte digitale servira en lieu et place de la signature, et un adulte lettré indépendant de l'étude qui servira de témoin (un membre du même ménage ou un voisin par exemple) signera le "Formulaire 2 – FORMULAIRE DE CONSENTEMENT".
- Collecter les informations sur tous les membres du ménage indépendamment du fait qu'ils soient présents ou non au moment de l'entretien; Recueillir l'information sur seulement les individus présents au moment de l'entretien résultera en un biais dans les données.
- Remplir le "Formulaire 2 – FORMULAIRE DE CONSENTEMENT" pour chaque ménage consentant enrôlé dans l'étude.
- Expliquer au répondant qu'il est prévu de revisiter chaque ménage durant une période de 2 ans; a chaque visite, l'équipe de recherche obtiendra un nouveau consentement.
- «Site» et «sous-zone» doivent être un numéro à trois chiffres, «numéro d'habitation» un numéro à quatre chiffres, et «identifiant de l'enquêteur» un numéro à deux chiffres (utiliser les listes préparées individuellement pour chaque site pour obtenir les codes); saisir «date» au format JJ/MM/AAAA et «heure» au format HH:MM et entourer MATIN ou APRÈS-MIDI. «Étiquette de l'étude» doit être une combinaison d'un code «site» à trois chiffres, d'un code «sous-zone» à trois chiffres et d'un code «numéro d'habitation» à quatre chiffres; le «identifiant de l'enquêteur» doit être un numéro à trois chiffres.

Investigateur principal et co-investigateur de l'étude

[Ajouter les noms de l'investigateur principal et des co-investigateurs de l'étude ainsi que leurs affiliations respectives]

Introduction

L'Institut International des Vaccins (IVI) et [ajouter l'institution d'affiliation des investigateurs locaux] travaillent ensemble pour examiner les problèmes de santé et le comportement de santé dans votre communauté [ajouter le site SETA-pays], y compris le statu socio-économique, le comportement d'hygiène et d'assainissement et le statut vaccinal des enfants. Nous aimerions mieux comprendre où vous, votre famille et vos voisins cherchez des soins de santé en cas de maladies causant la fièvre/corps chaud et autres chante & symptômes. Cette information nous aidera à savoir à quelle fréquence vous souffrez de maladies causant de la fièvre et les autres liées à la fièvre, fournir une meilleure image des facteurs influençant la transmission de la maladie et l'infection, et à trouver des moyens pour les prévenir et traiter dans votre communauté. Vous, en tant que responsable principal pour le comportement de santé/l'utilisation des soins de santé quotidienne pour les membres de ce ménage, êtes invités à participer parce que vous vivez dans la localité où nous effectuons cette étude. Ce formulaire de consentement sera lu par vous ou pour vous et vous aurez le temps pour prendre votre décision d'accepter ou de refuser de participer à l'étude. S'il vous plaît, demandez aux membres de l'étude d'expliquer tout mot ou toute information que vous ne comprenez pas. On vous donnera en conséquence plus d'informations sur cette étude, y compris ses risques potentiels, les avantages et les inconvénients. Après vous avoir entièrement expliqué l'étude et si vous acceptez de participer, on vous demandera de signer ce formulaire de consentement au nom de tous les membres du ménage. Si vous ne pouvez pas signer le formulaire, nous vous demandons de fournir votre empreinte digitale, et une personne adulte indépendante de l'étude (membre de votre ménage/voisin) signera le formulaire en tant que témoin. Nous allons vous remettre une copie signée et datée du formulaire de consentement.

Objectif

L'objectif de cette étude est d'examiner les problèmes de santé et l'utilisation des soins de santé dans votre communauté [ajouter le site SETA-pays], y compris le statu socio-économique et le comportement d'hygiène et d'assainissement. Notre objectif est de mieux comprendre où vous, votre famille et vos voisins cherchez les soins de santé pour des maladies causant de la fièvre/corps chaud et autres chante & symptômes. Cette

For peer review only - http://bmjopen.bmj.com/site/about/guidelines.xhtml

information permettra de mieux estimer le fardeau des maladies communes et d'identifier les moyens pour la mise en place de mesures de prévention et de traitement pour les maladies.

Procédures

Nous visiterons environ [ajouter le nombre pour site d'étude et pays] ménages au total à [ajouter le site et pays] et poserons les mêmes questions dans chaque ménage. Votre ménage a été choisi pour cette étude simplement par hasard. Si vous acceptez de participer, nous vous poserons, au nom de tous les membres du ménage, quelques questions au sujet de votre ménage tels que les initiales, le sexe et l'âge de chaque membre. Nous aimerions savoir certaines informations relatives à l'éducation et l'occupation du chef de ménage, au revenu, à la possession de biens, au logement lui-même ainsi qu'à l'hygiène et à l'assainissement dans le contexte de l'hygiène personnelle, de la préparation et de la manipulation des aliments, des sources d'eau et de la manipulation, de l'élimination des déchets, de la manipulation des animaux de ferme et domestiques ainsi que de l'élevage de ce ménage. Nous allons également demander où les membres de votre ménage obtiennent les soins si quelqu'un a une maladie causant de la fièvre ou toute autre maladie et les facteurs qui peuvent influer sur l'utilisation des soins de santé des membres du ménage (c'est-à-dire les modalités de transport, la disponibilité d'assurance maladie, les facteurs culturels) en plus du statut vaccinal et de la perception de certaines maladies. Enfin, nous allons mesurer la position géographique de votre maison. Il n'y a pas de bonnes ou de mauvaises réponses. Vous êtes libre de choisir les questions à répondre. Somme toute, il devrait prendre environ 30-45 minutes pour répondre à toutes les questions. L'entretien peut prendre un peu plus de temps s'il y a beaucoup de membres dans ce ménage. En outre, nous visiterons votre ménage deux fois en différentes saisons (une fois pendant la saison sèche et une fois vers la fin de la saison des pluies) pendant la période totale de l'étude qui est de deux ans environ pour recueillir des données comme décrit dans ce paragraphe du formulaire de consentement.

Participation

Vous, le décideur principal pour le comportement de santé quotidienne et l'utilisation des soins de santé dans ce ménage, ainsi que les membres de votre ménage, êtes libres de choisir d'accepter ou de refuser de participer à l'étude. Il n'y a aucun problème si vous ne voulez pas participer. Il n'y a non plus aucun problème si vous voulez arrêter votre participation à tout moment, et cela ne causera aucun tord à vous ou aux membres de votre ménage. Donc, la participation à cette étude est volontaire, et vous êtes libre de refuser de participer à l'étude ou vous pouvez retirer votre consentement à tout moment sans donner de raisons et ceci n'entraînera aucune pénalité.

Risques et avantages

La participation à cette étude n'entraine pas de risque direct. Poser des questions au sujet de votre ménage peut mettre mal à l'aise vous ou les membres de votre ménage. Après avoir terminé les questions, l'équipe de recherche pourra fournir des informations sur les soins de santé communautaires pour les participants qui expriment de la détresse. Vous pouvez refuser de répondre à toute question à tout moment. Vous pouvez faire une pause ou arrêter de participer à cette étude à tout moment. Il n'y a pas d'avantages directs à être dans cette étude. Au cours des visites de votre ménage, notre équipe de recherche peut identifier les membres de votre ménage qui devraient chercher des soins de santé pour les maladies causant de la fièvre et autres. Par conséquent, elle peut aider à fournir de l'information sur l'offre de soins établie dans votre communauté. Les avantages indirects sont que toutes les informations collectées contribueront à fournir des données exactes sur les problèmes de santé, y compris le statu socio-économique, le comportement d'hygiène et d'assainissement et le statut vaccinal des enfants dans votre communauté. Cela aidera à donner une meilleure compréhension des facteurs influençant la transmission de la maladie et l'infection, et à estimer le fardeau des maladies courantes ainsi qu'à trouver des moyens de prévenir et de traiter les personnes dans votre communauté.

Coûts et Compensations

Il n'a pas de coûts pour les participants de cette étude. Ni vous ni aucune personne de votre ménage ne sera indemnisée d'avoir participé.

Confidentialité

Tous les fichiers de l'étude et de toutes les informations qui identifient vous et les membres de votre ménage seront gardées confidentielles. Tous les questionnaires sur papier seront gardés en toute sécurité et les fichiers informatisés seront protégés par un mot de passe; tous les fichiers de données (sur papier/informatisés) seront stockées pour un minimum de cinq ans; ces fichiers seront accessibles seulement à quelques personnes autorisées de l'étude. Ni votre nom ni aucun identifiant ne sera utilisé dans les publications ou les rapports de

For peer review only - http://bmjopen.bmj.com/site/about/guidelines.xhtml

cette étude. Les informations que nous collectons à votre sujet et au sujet des membres de votre ménage seront partagées uniquement avec le personnel de l'étude autorisé et des représentants du comité d'éthique.

Personnes de contact pour des questions
Si vous ou des membres de votre ménage avez des questions, s'il vous plaît, demandez maintenant à l'équipe de recherche ou à tout moment plus tard en contactant [ajouter le nom de l'investigateur principal et/ou du co-investigateur, leurs affiliations respectives et les numéros de téléphone]. Si vous avez des questions sur vos droits ou ceux des membres du ménage en tant que participant à l'étude, vous pouvez contacter [ajouter le nom et les coordonnées du comité d'éthique local].

Déclaration de consentement
Ce formulaire de consentement pour participer à l'évaluation des soins de santé et d'autres facteurs liés à la population à [ajouter le site et pays] a été bien lu par moi/pour moi. L'objectif, les procédures, les risques et les avantages ont m'ont été expliqués en détails. J'ai été autorisé à poser des questions à tout moment, et mes questions ont été répondues à ma satisfaction par l'équipe de recherche. On m'a dit la personne à contacter en cas de questions ou si je veux discuter de problèmes ou préoccupations. On m'a dit que je recevrai une copie signée et datée de ce formulaire de consentement. Je suis rassuré que toutes les informations obtenues par suite de cette étude seront gardées confidentielles et utilisées uniquement aux fins de cette étude, et seulement par les institutions participantes.

Moi, le répondant, consens, par la présente, volontairement à participer à cette étude. Je suivrai les instructions de l'équipe de recherche et donnerai mon entière collaboration. Je comprends que j'ai le droit de me retirer de l'étude à tout moment.

RÉPONDANT

Nom : ____
(prénom + deuxième prénom (si disponible) + nom de famille)

Signature : ____
(Représentation manuscrite du nom/ marque que la personne écrit sur un document comme une preuve d'identité)

Date ______/______/______________ (YY/MM/AAAA)

Empreinte du pouce

Si le répondant ne peut pas signer/est analphabète, mais accepte de participer, enregistre l'empreinte du pouce dans la boîte ci-dessus, et demande à un témoin adulte, alphabétisé et indépendant de l'étude (par exemple membre du même ménage ou un voisin), de signer ci-dessous :

TÉMOIN

Nom : ____
(prénom + deuxième prénom (si disponible) + nom de famille)

Signature : ____
(Représentation manuscrite du nom/ marque que la personne écrit sur un document comme une preuve d'identité)

Date ______/______/______________ (YY/MM/AAAA)

Moi, l'enquêteur, j'ai lu/expliqué l'étude au répondant nommé ci-dessus (témoin si le répondant est analphabète) dans une langue que le répondant comprend bien. Je suis certain que le répondant a compris l'information et accepte qu'on lui pose des questions.

INTERVIEWER

Nom : ____
(prénom + deuxième prénom (si disponible) + nom de famille)

Signature : ____
(Représentation manuscrite du nom/ marque que la personne écrit sur un document comme une preuve d'identité)

Date ______/______/______________ (YY/MM/AAAA)

For peer review only - http://bmjopen.bmj.com/site/about/guidelines.xhtml

Site ___ ___ ___ Sous-zone ___ ___ ___ Numéro d'habitation ___ ___ ___ ___ Identifiant de l'enquêteur ___ __

FORMULAIRE 3: INFORMATIONS GÉNÉRALES SUR LE MÉNAGE

Instructions pour l'enquêteur

- Compléter ce formulaire uniquement si la personne interrogée a consenti à participer (voir « Formulaire 2 — FORMULAIRE DE CONSENTEMENT ») ; la personne interrogée ainsi que définie pour cette enquête est un adulte (selon l'âge légal de majorité du pays), un membre du ménage et un décideur en ce qui concerne les soins de santé quotidiens et l'utilisation de soins de santé pour le ménage entier et ses membre ; il ne doit pas être, par exemple, un voisin ou un visiteur.
- Recueillir les données de tous les membres du ménage que tous les membres du ménage soient présents ou non au moment de l'entretien ; ne recueillir et n'inscrire les données que des personnes présentes au moment de l'entretien fausserait les données.
- Un ménage est défini/les membres d'un ménage sont définis comme une personne ou un groupe de personnes avec ou sans lien de parenté qui vivent ensemble dans la même unité d'habitation, qui reconnaissent un adulte de sexe masculin ou féminin comme chef de ménage, qui partagent les mêmes installations domestiques, qui sont considérés comme constituant une unité, et qui subviennent à leurs besoins en termes de nourriture et des autres éléments essentiels pour vivre.
- « Site » et « sous-zone » doivent être un numéro à trois chiffres, « numéro d'habitation » un numéro à quatre chiffres, et « identifiant de l'enquêteur » un numéro à deux chiffres (utiliser les listes préparées individuellement pour chaque site pour obtenir les codes) ; saisir « date » au format JJ/MM/AAAA et « heure » au format HH:MM et entourer MATIN ou APRÈS-MIDI. «Étiquette de l'étude» doit être une combinaison d'un code «site» à trois chiffres, d'un code «sous-zone» à trois chiffres et d'un code «numéro d'habitation» à quatre chiffres; le « identifiant de l'enquêteur » doit être un numéro à trois chiffres.

FORMULAIRE 3: INFORMATIONS GÉNÉRALES SUR LE MÉNAGE
PARTIE A – DONNÉES DÉMOGRAPHIQUES/ INFORMATIONS GÉNÉRALES SUR LE MÉNAGE

EMPLACEMENT DU DOMICILE

1 Numéros de site, de sous-zone et d'habitation

Instructions : Site (=site d'étude) et sous-zone (=plus petite unité du site d'étude) doivent être tous les deux des numéros à trois chiffres, et numéro d'habitation doit être un code à quatre chiffres ; utiliser les listes préparées individuellement pour obtenir les codes respectifs.

Site: ____ ____ ____ Sous-zone: ____ ____ ____ Numéro d'habitation: ____ ____ ____ ____

2 Coordonnées GPS

Instructions : Prendre les coordonnées GPS approximativement au centre de la maison. Inscrire l'altitude par un nombre à quatre chiffres (unité : mètre), et la latitude/longitude par un numéro à huit chiffres (unités : degrés, minutes décimales).

O 1=Ménage d'origine O 2=Ménage de remplacement (côte droit/gauche du ménage d'origine)

Altitude: ___ ___ ___ ___

Latitude: ___ ___°___ ___.___ ___ ___' Longitude: ___ ___°___ ___.___ ___ ___'

PERSONNE INTERROGÉE

3 Inscrire le nom complet de la personne interrogée.

Instructions : Inscrire le prénom, le deuxième nom (le cas échéant) et le nom de famille de la personne interrogée.

____

4 Inscrire l'âge de la personne interrogée.

Instruction : Utiliser un numéro à trois chiffres (unité: année). _____ _____ _____

5 Inscrire le sexe de la personne interrogée.

O 1=Masculin O 2=Féminin

6 Quel est le lien de la personne interrogée avec le ménage ?

O 1=Chef de ménage
O 2=Fils/fille du chef de ménage
O 7=Conjoint du chef de ménage
O 8=Conjoint du fils/fille du chef de ménage

For peer review only - http://bmjopen.bmj.com/site/about/guidelines.xhtml

○ 3=Frère/sœur du chef de ménage
○ 4=Mère/père du chef de ménage
○ 5=Petite-fille/petit-fils du chef de ménage
○ 6=Autre, préciser _________
○ 9=Conjoint du frère/sœur du chef de ménage
○ 10=Belle-mère/beau-père du chef de ménage
○ 11=Nièce/neveu du chef de ménage
○ 98=Pas de réponse

MEMBRES DU MÉNAGE

7 Combien de membres du ménage vivent dans ce domicile au total (au moment de la visite) **?**
Instruction : Utiliser un numéro à deux chiffres.
Nombre total de membres du ménage : ____ ____

8 Inscrire l'âge et le sexe de chaque membre du ménage.
Instructions : Inscrire les initiales, l'âge et le sexe de la personne interrogée en première position/ligne du tableau. Les initiales, l'âge et le sexe de tous les autres membres du ménage doivent être inscrits ensuite. Les initiales d'un membre du ménage consistent en les trois premières lettres du prénom et les trois première lettre du nom de famille (un éventuel deuxième nom ne fait pas partie des initiales). Inscrire l'âge de chaque membre du ménage avec un numéro à trois chiffres (unité : années) ; si l'âge d'un des membres du ménage ≤ 12 mois, inscrire 1 an comme âge. Merci de compléter un formulaire supplémentaire si le ménage a plus de vingt membres.

Membre du ménage Identifiant	Initiales [prénom/nom de famille]	Âge [années]	Sexe
1/Personne interrogée	____ ____ ____/____ ____ ____	____ ____ ____	○1=Masculin ○2=Féminin
2	____ ____ ____/____ ____ ____	____ ____ ____	○1=Masculin ○2=Féminin
3	____ ____ ____/____ ____ ____	____ ____ ____	○1=Masculin ○2=Féminin
4	____ ____ ____/____ ____ ____	____ ____ ____	○1=Masculin ○2=Féminin
5	____ ____ ____/____ ____ ____	____ ____ ____	○1=Masculin ○2=Féminin
6	____ ____ ____/____ ____ ____	____ ____ ____	○1=Masculin ○2=Féminin
7	____ ____ ____/____ ____ ____	____ ____ ____	○1=Masculin ○2=Féminin
8	____ ____ ____/____ ____ ____	____ ____ ____	○1=Masculin ○2=Féminin
9	____ ____ ____/____ ____ ____	____ ____ ____	○1=Masculin ○2=Féminin
10	____ ____ ____/____ ____ ____	____ ____ ____	○1=Masculin ○2=Féminin
11	____ ____ ____/____ ____ ____	____ ____ ____	○1=Masculin ○2=Féminin
12	____ ____ ____/____ ____ ____	____ ____ ____	○1=Masculin ○2=Féminin
13	____ ____ ____/____ ____ ____	____ ____ ____	○1=Masculin ○2=Féminin
14	____ ____ ____/____ ____ ____	____ ____ ____	○1=Masculin ○2=Féminin
15	____ ____ ____/____ ____ ____	____ ____ ____	○1=Masculin ○2=Féminin
16	____ ____ ____/____ ____ ____	____ ____ ____	○1=Masculin ○2=Féminin
17	____ ____ ____/____ ____ ____	____ ____ ____	○1=Masculin ○2=Féminin
18	____ ____ ____/____ ____ ____	____ ____ ____	○1=Masculin ○2=Féminin
19	____ ____ ____/____ ____ ____	____ ____ ____	○1=Masculin ○2=Féminin
20	____ ____ ____/____ ____ ____	____ ____ ____	○1=Masculin ○2=Féminin

For peer review only - http://bmjopen.bmj.com/site/about/guidelines.xhtml

FORMULAIRE 3: INFORMATIONS GÉNÉRALES SUR LE MÉNAGE
PARTIE B – DONNÉÉS SOCIO-ECONOMIQUES

ÉDUCATION

1 Le chef de ménage sait-il lire en [inscrire la langue officielle du site d'étude/pays] **?**
O 1=Oui O 2=Non O 99=Ne sait pas O 98=Pas de réponse

2 Le chef de ménage sait-il écrire en [inscrire la langue officielle du site d'étude/pays] **?**
O 1=Oui O 2=Non O 99=Ne sait pas O 98=Pas de réponse

3 Quel est le niveau d'éducation scolaire le plus élevé atteint par le chef de ménage ?
O 1=École primaire O 4=Plus élevé que le lycée O 99=Ne sait pas
O 2=Collège O 5= PAS D'ÉDUCATION O 98=Pas de réponse
O 3=Lycée O 6=Autre, préciser ___________

PROFESSION/RESSOURCES FINANCIÈRES

4 Quelle est la profession principale du chef de ménage ?
Instructions : Inscrire le statut du jour de visite uniquement.

O 1=Groupe 1 : Cadres
(directeurs généraux, hauts fonctionnaires, législateurs ; cadres administratifs et commerciaux ; responsables de production et de services spécialisés ; responsables dans l'hôtellerie, la vente et d'autres services)

O 2=Groupe 2 : Professionnels
(scientifiques ou ingénieurs ; professionnels de santé ; professionnels de l'enseignement ; professionnels de l'administration et des affaires ; professionnels de l'information et des technologies de communication ; professionnels du droit, du social ou de la culture)

O 3=Groupe 3 : Techniciens et professions intermédiaires
(Professions intermédiaires des sciences et techniques ; professions intermédiaires de la santé ; professions intermédiaires de l'administration et des affaires ; professions intermédiaires du droit, du social, de la culture et assimilés ; techniciens de l'information et des communications)

O 4=Groupe 4 : Employés de bureau
(secrétaires et dactylographes ; employé de service clientèle ; employé de conservation des données et de la documentation ; autres employés de bureau)

O 5=Groupe 5 : Travailleurs des services et de la vente
(travailleurs du service aux personnes ; travailleurs de la vente ; travailleurs des soins aux personnes ; travailleurs des services de protection)

O 6=Groupe 6 : Travailleurs qualifiés de l'agriculture, la sylviculture et la pêche
(travailleurs qualifiés de l'agriculture orientés marché ; éleveurs d'animaux; travailleurs qualifiés de la sylviculture, la pêche et la chasse orientés marché ; agriculteurs, pêcheurs, chasseurs et cueilleurs de subsistance)

O 7=Groupe 7 : Travailleurs manuels et assimilés
(travailleurs de la construction et assimilés (à l'exception des électriciens) ; travailleurs de la métallurgie, sur machines et assimilés ; artisans et travailleurs de l'imprimerie ; travailleurs des métiers de l'électrique et de l'électronique ; travailleurs des métiers de la transformation des aliments, du bois, des vêtements et assimilés)

O 8=Groupe 8 : Opérateurs de machines et d'installations, et monteurs
(Opérateurs de machines et d'installations fixes ; monteurs ; conducteurs opérateurs d'installations mobiles)

O 9=Groupe 9 : Emplois non qualifiés
(agents d'entretien et assistants ; ouvriers de l'agricultures, la sylviculture et la pêche ; travailleurs des mines, de la construction, de l'industrie et des transports ; assistants à la préparation des aliments ; vendeurs et services de rue et assimilés ; travailleurs de la collecte des ordures et autres travailleurs non qualifiés)

O 10=Groupe 0 : Professions des forces armées
(officiers des forces armées ; sous-officiers des forces armées ; professions des forces armées, autres grades)

O 11=Autre, préciser __________
O 12=PAS DE PROFESSION O 99=Ne sait pas O 98=Pas de réponse

For peer review only - http://bmjopen.bmj.com/site/about/guidelines.xhtml

5 Quel est le montant moyen des revenus mensuels du chef de ménage en lien avec à la profession indiquée en question 4 ainsi que celui des autres membre du ménage ?
Instruction: Tick one answer per household head/enter one code per household member if applicable/if income available.

Chef de ménage	Membre 1	Membre 2	Membre 3
Code ____________	Code ____________	Code ____________	Code ____________
[1] <100USD<br>[2] ≥100 à <150USD	[3] ≥150 à <200USD<br>[4] ≥200 à <250USD	[5] ≥250USD<br>[6] PAS DE REVENU (chef de ménage; voir Q4)	[99] Ne sait pas<br>[98] Pas de réponse

6 Quels sont les autres sources ou les sources supplémentaires de revenus mensuels du ménage et quel en est le montant ?
Instruction : Cocher plusieurs cases si nécessaire ; il peut y avoir plus d'une réponse.

Autres sources/Sources supplémentaires	Revenus financiers mensuels globaux
O 1=Aides sociales<br>O 2=Assurances (publiques ou privées)<br>O 3=Prêt (d'amis, de membres de la famille, d'une banque)<br>O 4=Vente (bétail, récoltes, meubles, autres biens)<br>O 5=Économies personnelles<br>O 6=Autre, préciser ________________	O 1=<100 USD<br>O 2=≥100 à <150 USD<br>O 3=≥150 à <200 USD<br>O 4=≥200 à <250 USD<br>O 5=≥250 USD
O 7=PAS D'AUTRES SOURCES/DE SOURCES SUPPLÉMENTAIRES<br>O 99=Ne sait pas<br>O 98=Pas de réponse	

LOGEMENT/HABITATION

7 Les membres du ménage sont-ils propriétaires de l'habitation ?
O 1=Oui O 3=Non, logement fourni (par le gouvernement, un employeur) O 99=Ne sait pas
O 2=Non, locataires O 4=Non, autres, préciser ________ O 98=Pas de réponse

8 Combien de pièces ou structures individuelles de l'habitation sont utilisées pour dormir, vivre, faire la cuisine ou se laver ?
Instruction : N'indiquer qu'un code par catégorie.

Chambre	Salon	Cuisine	Salle de bain
Code ____________	Code ____________	Code ____________	Code ____________
[1] 1-2<br>[2] 3-4	[2] >4<br>[2] Aucune	[99] Ne sait pas<br>[98] Pas de réponse	

9 En quel matériau sont construits le sol, les murs et le toit des pièces/des structures individuelles de l'habitation ?
Instruction : Indiquer tous les codes par catégorie ; plus d'une réponse par catégorie est possible.

Sol	Murs	Toit
Code ____________	Code ____________	Code ____________
[1] Brique [3] Tuiles<br>[2] Ciment/Béton [4] Terre	[5] Bois<br>[6] Paille/feuilles	[7] Métal/Étain [99] Ne sait pas<br>[8] Autre, préciser_________ [98] Pas de réponse

10 Les membres de ce ménage ont-ils accès à/utilisent-ils l'électricité dans cette habitation ?
O 1=Oui O 2=Non O 99=Ne sait pas O 98=Pas de réponse

11 À quelle source de lumière principale les membres de ce ménage ont-ils accès/Quelle source de lumière principale utilisent-ils ?
O 1=Lumière/ampoule(s) électrique(s) O 4=Lampe(s) à piles O 7= Autre, préciser_____
O 2=Lumière du soleil/Lampe(s) solaire(s) O 5=Bougie(s) O 99=Ne sait pas
O 3=Lampe(s) à pétrole/à huile O 6=PAS DE SOURCE DE LUMIÈRE O 98=Pas de réponse

For peer review only - http://bmjopen.bmj.com/site/about/guidelines.xhtml

BIENS DU MÉNAGE

12 Parmi les articles suivants, lesquels les membres du ménage possèdent-ils ?

Instructions : Cocher plusieurs cases si nécessaire ; il peut y avoir plus d'une réponse ; indiquer la quantité d'articles fonctionnels si nécessaire

O 1=Radio	Quantité ____	O Rien	O 11=Lecteur cassettes/CD	Quantité ____	O Rien
O 2=Télévision	Quantité ____	O Rien	O 12=Ordinateur de bureau/portable/tablette	Quantité ____	O Rien
O 3=Connexion internet	Quantité ____	O Rien	O 13=Téléphone portable	Quantité ____	O Rien
O 4=Voiture	Quantité ____	O Rien	O 14=Camion/bus	Quantité ____	O Rien
O 5=Moto	Quantité ____	O Rien	O 15=Vélo	Quantité ____	O Rien
O 6=Char à bœufs/ânes	Quantité ____	O Rien	O 16=Lit	Quantité ____	O Rien
O 7=Matelas	Quantité ____	O Rien	O 17=Moustiquaire	Quantité ____	O Rien
O 8=Table	Quantité ____	O Rien	O 18=Chaise	Quantité ____	O Rien
O 9=Réfrigérateur (4°C)	Quantité ____	O Rien	O 19=Congélateur (-20°C)	Quantité ____	O Rien
O 10=Ventilateur	Quantité ____	O Rien	O 20=Machine à coudre	Quantité ____	O Rien

For peer review only

For peer review only - http://bmjopen.bmj.com/site/about/guidelines.xhtml

FORMULAIRE 3: INFORMATIONS GÉNÉRALES SUR LE MÉNAGE
Partie C – Hygiène et installations sanitaires

TOILETTES

1 À quel type de toilettes les membres du ménage ont-ils habituellement accès ?

Instruction : À l'intérieur signifie à l'intérieur d'une pièce/d'une habitation individuelle/d'un bâtiment ; à l'extérieur signifie à l'extérieur d'une pièce/d'une habitation individuelle/d'un bâtiment.

- O 1=Toilettes avec chasse d'eau/Cabinets (à l'intérieur)
- O 2=Toilettes avec chasse d'eau/Cabinets (à l'extérieur)
- O 3=Latrines sans chasse/latrines à fosse (à l'intérieur)
- O 4=Latrines sans chasse/latrines à fosse (à l'extérieur)
- O 5=Latrines à fosse améliorées par une ventilation (KVIP, à l'intérieur)
- O 6=Latrines à fosse améliorées par une ventilation (KVIP, à l'extérieur)
- O 7=Toilettes à compostage (à l'intérieur)
- O 8=Toilettes à compostage (à l'extérieur)
- O 9=PAS de toilettes, utilisent un seau
- O 10=PAS de toilettes, creusent un trou
- O 11=PAS de toilettes, défécation libre/en plein air
- O 12=Autre, préciser __________
- O 99=Ne sait pas
- O 98=Pas de réponse

2 Les membres du ménage utilisent-ils habituellement les toilettes indiquées à la question 1 ?

- O 1=Non, les membres ne peuvent utiliser les toilettes de façon autonome (bébés, enfants en bas-âge)
- O 2=Non, les membres ne peuvent utiliser les toilettes de façon autonome (handicapés, malades chroniques/longue durée)
- O 3=Non, les membres ne peuvent utiliser les toilettes de façon autonome (personnes âgées)
- O 4=Non, accès limité (publiques, partagées avec d'autres ménages/communautés)
- O 5=Non, accès limité (publiques, partagées avec d'autres ménages/communautés)
- O 6= Non, mauvais état (cassé, pas propre)
- O 7=OUI
- O 8=Autre, préciser _________________
- O 99=Ne sait pas
- O 98=Pas de réponse

3 Où sont situées les toilettes indiquées à la question 1 ? Par combien de personnes sont-elles habituellement partagées ?

- O 1=Sur la propriété du ménage (privées, non partagées)
- O 2=Sur la propriété d'un ménage voisin (privées, partagées)
  - *Partagées par* O 1=<10 personnes O 2=11-24 personnes O 3=25-49 personnes O 4=>50 personnes O 99=Ne sait pas O 98=Pas de réponse
- O 3=Dans la communauté (publiques, partagées)
  - *Partagées par* O 1=<10 personnes O 2=11-24 personnes O 3=25-49 personnes O 4=>50 personnes O 99=Ne sait pas O 98=Pas de réponse
- O 4=Autre, préciser__________ O 99=Ne sait pas O 98=Pas de réponse

4 Comment les membres du ménage qui ont accès/n'ont pas accès aux toilettes se lavent-ils habituellement les mains tout de suite avant et après avoir uriné/déféqué ?

Instruction : Indiquer un code par catégorie uniquement.

Lavent les mains avant d'avoir uriné/déféqué	Lavent les mains après avoir uriné/déféqué
Code _____________	Code _____________
[1] Lavent les mains avec du savon et de l'eau<br>[2] Lavent les mains avec de l'eau uniquement<br>[3] Frottent les mains avec des feuilles/de la paille/de l'herbe<br>[4] Frottent les mains avec du sable<br>[5] Frottent les mains avec un chiffon/du tissu	[6] Ne lavent pas, pas besoin/pas sale<br>[7] Ne lavent pas, rien pour laver les mains<br>[8] Autre, préciser __________<br>[99] Ne sait pas<br>[98] Pas de réponse

CUISINE/MANIPULATION DES ALIMENTS

5 Les membres de ce ménage ont-ils une cuisine pour faire cuire/préparer la nourriture/les repas ? Où est-elle située ?

- O 1=Oui, cuisine intérieure/dans une pièce/structure individuelle d'habitation ; sur la propriété du ménage (privée, non partagée)
- O 2=Oui, cuisine extérieure/hors d'une pièce/structure individuelle d'habitation/ en plein air ; sur la propriété du ménage (privée, non partagée)

For peer review only - http://bmjopen.bmj.com/site/about/guidelines.xhtml

O 3=Oui, cuisine intérieure/dans une pièce/structure individuelle d'habitation ; sur la propriété d'un ménage voisin (privée, partagée)
O 4=Oui, cuisine extérieure/hors d'une pièce/structure individuelle d'habitation/ en plein air ; sur la propriété d'un ménage voisin (privée, partagée)
O 5=PAS DE CUISINE
O 6=Autre, préciser __________ O 99=Ne sait pas O 98=Pas de réponse

6 Quelle source d'énergie les membres de ce ménage utilisent-ils habituellement pour cuisiner/préparer la nourriture/les repas ?

O 1=Électricité O 3=Charbon de bois O 5=Excréments d'animaux O 99=Ne sait pas
O 2=Gaz/pétrole O 4=Bois/paille/feuilles O 6=Autre, préciser __________ O 98=Pas de réponse

7 Quelle moyen de cuisson les membres de ce ménage utilisent-ils habituellement pour cuisiner/préparer la nourriture/les repas ?

O 1=Foyer ouvert O 3=Fourneau intérieur avec cheminée O 99=Ne sait pas
O 2=Fourneau extérieur O 4=Autre, préciser __________ O 98=Pas de réponse

8 Où les membres de ce ménage conservent-ils habituellement les produits alimentaires frais/crus (comme les légumes, la salade, les fruits, les produits laitiers, les œufs, la viande et le poisson) et les restes/les aliments/repas préparés ?

Instruction : Indiquer un code par catégorie uniquement. À l'intérieur signifie à l'intérieur d'une pièce/d'une habitation individuelle/d'un bâtiment ; à l'extérieur signifie à l'extérieur d'une pièce/d'une habitation individuelle/d'un bâtiment.

Des produits alimentaires frais/crus (légumes, salade, fruits, produits laitiers, œufs, viande et poisson)	Des restes/aliments/repas préparés
Code _____________	Code _____________
[1] À température ambiante (à l'intérieur, sur la propriété du ménage, privé)<br>[2] À température ambiante (à l'extérieur, sur la propriété du ménage, privé)<br>[3] À température ambiante (à l'intérieur, sur la propriété d'un ménage voisin, privé, partagé)<br>[4] À température ambiante (à l'extérieur, sur la propriété d'un ménage voisin, privé, partagé)<br>[5] Réfrigérateur (sur la propriété du ménage, privé)<br>[6] Réfrigérateur (sur la propriété d'un ménage voisin, privé, partagé)<br>[7] Congélateur (sur la propriété du ménage, privé)<br>[8] Congélateur (sur la propriété d'un ménage voisin, privé, partagé)<br>[9] Autre, préciser ______________ [10] PAS DE CONSERVATION [99] Ne sait pas [98] Pas de réponse	

9 Si la réponse à la question 8 au sujet des produits alimentaires frais/crus et/ou des restes/aliments/repas préparés est «température ambiante», combien de temps les membres de ce ménage les conservent-ils habituellement?

Instruction: Indiquer un code par catégorie uniquement.

Des produits alimentaires frais/crus (légumes, salade, fruits, produits laitiers, œufs, viande et poisson)	Des restes/aliments/repas préparés
Code _____________	Code _____________
[1] <1 jour [3] 3-4 jours [5] Pas applicable [99] Ne sait pas<br>[2] 1-2 jours [4] >4 jours [98] Pas de réponse	

10 Les membres de ce ménage couvrent-ils habituellement les restes/aliments/repas préparés? Réchauffent-ils/recuisent-ils les restes/aliments/repas préparés avant de les consommer?

Instruction : Indiquer un code par catégorie uniquement.

Couvrent les restes/les aliments/repas préparés	Réchauffent/Recuisent les restes/les aliments/repas préparés
Code _____________	Code _____________
[1] Oui [2] Non [99] Ne sait pas [98] Pas de réponse	

For peer review only - http://bmjopen.bmj.com/site/about/guidelines.xhtml

11 Quel membre(s) de ce ménage cuisine(s)/prépare(s) la nourriture/les repas habituellement pour les autres membres de ce ménage ?

Instructions : Se référer à la question 9 du « Formulaire 3 Partie A – Données démographiques/Informations générales sur le ménage » et indiquer les initiales/ Identifiant (s) du membre.

O Membre du ménage, indiquer les initiales/identificateur(s) __________

O 99=Ne sait pas O 98=Pas de réponse

12 Comment le(s) membre(s) de la famille qui cuisine(nt)/prépare(nt) la nourriture/les repas habituellement pour les autres membres de ce ménage comme indiqué à la question 11 se prépare(nt)-t-il(s)/elle(s) habituellement avant et après la préparation des repas?

Instruction: Indiquer un code par catégorie uniquement.

Avant la préparation des repas	Après la préparation des repas
Code _____________	Code _____________
[1] Lavent les mains avec du savon et de l'eau<br>[2] Lavent les mains avec de l'eau uniquement<br>[3] Frottent les mains avec des feuilles/de la paille/de l'herbe<br>[4] Frottent les mains avec du sable<br>[5] Frottent les mains avec un chiffon/du tissu	[6] Ne lavent pas, pas besoin/pas sale<br>[7] Ne lavent pas, rien pour laver les mains<br>[8] Autre, préciser __________<br>[99] Ne sait pas<br>[98] Pas de réponse

13 Comment les membres de ce ménage se préparent-ils habituellement avant et après avoir mangé de la nourriture/des repas, y compris le membre du ménage qui cuisine/prépare la nourriture/les repas habituellement comme indiqué à la question 11?

Instruction : Indiquer un code par catégorie uniquement.

Avant la préparation des repas	Après la préparation des repas
Code _____________	Code _____________
[1] Lavent les mains avec du savon et de l'eau<br>[2] Lavent les mains avec de l'eau uniquement<br>[3] Frottent les mains avec des feuilles/de la paille/de l'herbe<br>[4] Frottent les mains avec du sable<br>[5] Frottent les mains avec un chiffon/du tissu	[6] Ne lavent pas, pas besoin/pas sale<br>[7] Ne lavent pas, rien pour laver les mains<br>[8] Autre, préciser __________<br>[99] Ne sait pas<br>[98] Pas de réponse

14 Comment les membres de ce ménage mangent-ils habituellement leur nourriture/leurs repas, y compris le membre du ménage qui cuisine/prépare la nourriture/les repas habituellement comme indiqué à la question 11?

O 1=Avec des couverts O 3=Autre, préciser __________ O 99=Ne sait pas

O 2=Avec les doints O 98=Pas de réponse

15 Les membres de ce ménage mangent-ils habituellement leur nourriture/leurs repas dans le même récipient, y compris le membre du ménage qui cuisine/prépare la nourriture/les repas habituellement comme indiqué à la question 11?

O 1=Oui O 2=Non O 99=Ne sait pas O 98=Pas de réponse

16 Où les membres de ce ménage obtiennent-ils habituellement leurs produits alimentaires frais/crus principaux comme les légumes, les fruits, les grains/céréales, les produits laitiers, les œufs, la viande et le poisson?

Instruction : Indiquer un code par catégorie uniquement.

Légumes, fruits, grains/céréales	Produits laitiers	Œufs, viande, poisson
Code _____________	Code _____________	Code _____________
[1] Agriculture vivrière (cour/arrière-cour)<br>[2] Supermarché/Épicerie<br>[3] Amis/famille<br>[4] Marché ouvert<br>[5] Vendeur de rue	[6] Agriculture vivrière (terre agricole)<br>[7] Boucher<br>[8] Autre, préciser ______<br>[99] Ne sait pas<br>[98] Pas de réponse	

For peer review only

17 Les membres de ce ménage consomment/mangent-ils des produits alimentaires frais/crus (comme les légumes, la salade, les fruits, les produits laitiers, la glace, les œufs, la viande et le poisson) ? Si oui, lesquels?

Instruction : Cocher plusieurs cases si nécessaire ; plus d'une réponse est possible.

O 1=Légumes/salade (cultivés sur arbuste)
O 2=Légumes/salade (récolte sauvage sur arbuste)
O 3=Fruits (cultivés sur arbuste/arbre)
O 4=Fruits (récolte sauvage sur arbuste/arbre)
O 5=Produits laitiers (lait, fromage, yaourt d'animaux domestiques produisant du lait), préciser ____________
O 6=Œufs (de volailles domestiques), préciser _________
O 7=Sang animal (d'animaux domestiques), domestiques), préciser _________
O 8=Bœuf/Zébu/Buffle (domestiqué), préciser _________
O 9=Porc (domestiqué)
O 10=Mouton (domestiqué)
O 11=Viande d'animaux non-domestiqués (antilope, sanglier, volaille, rongeurs, poisson), préciser ________
O 12=PAS DE CONSOMMATION de produits frais/crus

O 13=Légumes/salade (cultivés au sol)
O 14=Légumes/salade (récolte sauvage sur le sol)
O 15=Fruits (cultivés au sol)
O 16=Fruits (récolte sauvage sur le sol)
O 17=Produits laitiers (lait, fromage, yaourt d'animaux non-domestiques produisant du lait), préciser ____________
O 18= Œufs (de volailles non-domestiques), préciser _________
O 19= Sang animal (d'animaux non- préciser _________
O 20=Volaille (domestiquée; poulet, canard, pigeon, oie, dinde), préciser _________
O 21=Chèvre (domestiquée)
O 22= Rongeurs (domestiqués), préciser ______
O 23=Poisson/fruits de mer, préciser _____
O 24=Glace
O 25=Autre, préciser _________
O 99=Ne sait pas O 98=Pas de réponse

18 Les membres de ce ménage consomment-ils habituellement des repas/produits alimentaires/boissons également en dehors de l'habitation ? Si oui, quel(s) membre(s) de ce ménage et où ?

Instructions : Se référer à la question 9 du « Formulaire 3 Partie A – Données démographiques/Informations générales sur le ménage » et indiquer les initiales du/des membre(s). Indiquer tous les codes nécessaires pour les membres du ménage respectifs.

Membre 1<br>Initiales/Identifiant : ___	Membre 2<br>Initiales/Identifiant : ___	Membre 3<br>Initiales/Identifiant : ___	Membre 4<br>Initiales/ Identifiant : ___
Code ____________	Code ____________	Code ____________	Code ____________
[1] Restaurant<br>[2] Chaîne de restauration rapide	[3] Cafétéria<br>[4] Vendeur de rue	[5] Autre, préciser ____<br>[6] Pas applicable	[99] Ne sait pas<br>[98] Pas de réponse

19 Quels/Quelles repas/produits alimentaires/boissons le/les membre(s) de ce ménage listé à la question 18 consomment-ils habituellement en dehors de l'habitation ?

Instructions : Se référer à la question 9 du « Formulaire 3 Partie A – Données démographiques/Informations générales sur le ménage » et indiquer/identifiant les initiales du/des membre(s). Indiquer jusqu'à 5 repas/produits alimentaires/boissons par membre du ménage. Vous pouvez vous référer aux produits alimentaires listés à la question 17. Passer cette question si aucun membre du ménage ne consomme les repas/les produits alimentaires/les boissons en dehord de la maison comme indiqué dans Q18.

Membre 1<br>Initiales/Identifiant : ____	Membre 2<br>Initiales/Identifiant : ____	Membre 3<br>Initiales/Identifiant : ____	Membre 4<br>Initiales/ Identifiant : ___
1: ______________<br>2: ______________<br>3: ______________<br>4: ______________<br>5: ______________	1: ______________<br>2: ______________<br>3: ______________<br>4: ______________<br>5: ______________	1: ______________<br>2: ______________<br>3: ______________<br>4: ______________<br>5: ______________	1: ______________<br>2: ______________<br>3: ______________<br>4: ______________<br>5: ______________

EAU

20 Quelle est la source d'approvisionnement en eau principale des membres de ce ménage pour la boisson, la cuisine/la préparation des aliments/repas, le bain et le ménage (de

For peer review only - http://bmjopen.bmj.com/site/about/guidelines.xhtml

l'habitation/de la/les structure(s) individuelle(s) d'habitation(s), les vêtements/les outils de cuisine) ?

Instruction : Indiquer un code par catégorie uniquement.

Boisson	Cuisine	Bain	Nettoyage
Code _____________	Code _____________	Code _____________	Code _____________

[1] Eau du robinet (à l'intérieur de l'habitation ; privée)
[2] Eau du robinet (à l'extérieur de l'habitation : cour/arrière-cour ; privée)
[3] Eau du robinet (à l'extérieur de l'habitation ; publique/partagée)
[4] Borne-fontaine/eau courante (à l'intérieur de l'habitation ; privée)
[5] Borne-fontaine/eau courante (à l'extérieur de l'habitation : cour/arrière-cour ; privée)
[6] Borne-fontaine/eau courante (à l'extérieur de l'habitation ; publique/partagée)
[7] Camion-citerne (public ; partagé)
[8] Eaux de surface (canal, canal d'irrigation)
[9] Eaux de surface (lac, étang, rivière, ruisseau)
[10] Eau de source
[11] Eau de pluie
[12] Puits/Puits de forage (en plein air/non couvert/non protégé)
[13] Puits/Puits de forage (couvert/protégé)
[14] Eau en bouteille
[15] Autre, préciser ___________________ [99] Ne sait pas [98] Pas de réponse

21 Les membres de ce ménage traitent-ils habituellement l'eau pour la boisson, la cuisine/la préparation des aliments/des repas, le bain, et le nettoyage (de l'habitation/des/de la structure(s) individuelle(s) d'habitation(s), des vêtements/des outils de cuisine) avant la consommation ? Comment l'eau est-elle habituellement traitée ?

Instruction: Indiquer tous les codes nécessaires par catégorie ; plus d'une réponse par catégorie est possible.

Boisson	Cuisine	Bain	Nettoyage
Code _____________	Code _____________	Code _____________	Code _____________

[1] Non, pas du tout
[2] Non, seulement pour les malades du ménage
[3] Non, seulement selon la saison (saison sèche)
[4] Oui, par filtration (tissu, céramique, sable)
[5] Oui, par sédimentation
[6] Autre, préciser ________________
[7] Non, seulement pour les enfants du ménage
[8] Non, seulement selon la saison (saison humide)
[9] Oui, par ébullition
[10] Oui, par désinfection solaire
[11] Oui, par chloration
[99] Ne sait pas [98] Pas de réponse

22 Quel(s) membre(s) de ce ménage recueille(nt) habituellement l'eau potable comme indiqué à la question 20 pour les autres membres de ce ménage ?

Instructions : Se référer à la question 9 du « Formulaire 3 Partie A – Données démographiques/Informations générales sur le ménage » et indiquer/identifiant les initiales du membre.

○ Membre du ménage, indiquer les initiales/identifiant(s) _________
○ 99=Ne sait pas ○ 98=Pas de réponse

23 Comment les membres de ce ménage conservent-ils habituellement l'eau potable indiquée à la question 20?

○ 1=Réservoir d'eau (non couvert)
○ 2=Réservoir d'eau (couvert par chiffon/tissu, couvercle, feuilles)
○ 3=Récipient (non couvert)
○ 4=Récipient (couvert par chiffon/tissu, couvercle, feuilles)
○ 5=Pot en terre (non couvert)
○ 6=Pot en terre (couvert par chiffon/tissu, couvercle, feuilles)
○ 7=Seau (non couvert)
○ 10=Bol (couvert par chiffon/tissu, couvercle, feuilles)
○ 11=Pot en bois (non couvert)
○ 12= Pot en bois (couvert par chiffon/ tissu, couvercle, feuilles)
○ 13= Récipient en peau (chèvre, mouton, bovins, non couvert)
○ 14=Récipient en peau (chèvre, mouton, bovins ; couvert par un chiffon/du tissu, un couvercle, des feuilles
○ 15=NON CONSERVÉE
○ 16=Autre, préciser _________

For peer review only - http://bmjopen.bmj.com/site/about/guidelines.xhtml

○ 8=Seau (couvert par chiffon/tissu, couvercle, feuilles)
○ 9=Bol (non couvert)
○ 99=Ne sait pas
○ 98= Pas de réponse

24 Si les membres de ce ménage utilisent une source d'approvisionnement en eau potable partagée comme indiqué à la question 20, par combien de personnes cette eau potable est-elle partagée ?

○ 1=<10 personnes
○ 2=11-24 personnes
○ 3=25-49 personnes
○ 4=>50 personnes
○ 5=NON PARTAGÉE
○ 99=Ne sait pas
○ 98=Pas de réponse

TRAITEMENT DES DÉCHETS

25 Où les membres de ce ménage jettent-ils habituellement les eaux usées de, par exemple, la cuisine/la préparation des aliments/repas, le bain et/ou le ménage, et jettent-ils habituellement les déchets domestiques, les déchets humains/déjections ?

Instruction: Indiquer un code par catégorie uniquement.

Eaux usées de par exemple cuisine/préparation des aliments/ repas, bain et/ou ménage	Déchets domestiques	Déchets humains/déjections
Code ____________	Code ____________	Code ____________

[1] Sur le sol/surface libre proche de l'habitation (cour/arrière-cour, privé)
[2] Sur le sol/surface libre loin de l'habitation dans la communauté (partagé, public)
[3] Dans un trou proche de la maison (cour/arrière-cour, privé)
[4] Dans un trou loin de la maison dans la communauté (partagé, public)
[5] Versement dans des eaux de surface dans la communauté (lac, étang, rivière, ruisseau, public, partagé)
[6] Versement dans des eaux de surface dans la communauté (canal, canal d'irrigation, public, partagé)
[7] Versement dans de l'eau de source dans la communauté (public, partagé)
[8] Versement dans une fosse septique proche de l'habitation (cour/arrière-cour, proche de toilettes si disponibles, privé)
[9] Versement dans une fosse septique dans la communauté (partagé, public)
[10] Versement dans les égouts proches de la maison (cour/arrière-cour, privé)
[11] Versement dans les égouts dans la communauté (partagé, public)
[12] Autre, préciser ________________________ [99] Ne sait pas [98] Pas de réponse

26 Les membres de ce ménage traitent-ils habituellement les déchets domestiques de la maison au préalable avant de les jeter ?

○ 1=Oui, incinération des déchets
○ 2=Oui, séparation/tri des déchet dégradable et non dégradables
○ 3=NON
○ 4=Oui, Autre, préciser _________
○ 99=Ne sait pas
○ 98=Pas de réponse

27 À quelle distance de la source d'approvisionnement en eau de ce ménage comme indiqué à la question 20 se trouve l'emplacement de l'élimination des eaux usées, des déchets domestiques et humains/excrétions de ce ménage comme indiqué à la question 25 ?

Instructions : Indiquer un code par catégorie uniquement. Indiquer la distance en mètres [m].

Eaux usées de, par exemple, cuisine/préparation des aliments/ repas, bain et/ou ménage	Déchets domestiques	Déchets humains/déjections
Code ____________	Code ____________	Code ____________

[1] <5 m
[2] ≥5 à <25 m
[3] ≥25 à <50 m
[4] ≥50 à <100 m
[5] ≥100 à <500 m
[6] ≥500 à <1,000 m
[7] ≥1,000 m
[99] Ne sait pas [98] Pas de réponse

28 À quelle distance de la source d'approvisionnement en eau principale de ce ménage pour la cuisine/la préparation des aliments/des repas comme indiqué à la question 20 se trouve l'emplacement de l'élimination des eaux usées, des déchets domestiques et humains/excrétions de ce ménage comme indiqué à la question 25 ?

Instructions : Indiquer un code par catégorie uniquement. Indiquer la distance en mètres [m].

Eaux usées de par exemple cuisine/préparation des aliments/ repas, bain et/ou ménage	Déchets domestiques	Déchets humains/excréments

For peer review only - http://bmjopen.bmj.com/site/about/guidelines.xhtml

Code ____________	Code ____________	Code ____________
[1] <5 m [3] ≥25 à <50 m<br>[2] ≥5 à <25 m [4] ≥50 à <100 m	[5] ≥100 à <500 m<br>[6] ≥500 à <1,000 m	[7] ≥1,000 m<br>[99] Ne sait pas [98] Pas de réponse

ANIMAUX DE FERME/DOMESTIQUES

29 Quel genre d'animaux/de bétail et d'animaux domestiques/de compagnie les membres de ce ménage possèdent-ils ?

Instruction : Cocher plusieurs cases par catégorie si nécessaire ; il peut y avoir plus d'une réponse par catégorie.

Animaux de ferme/bétail		Animaux domestiques/de compagnie
O 1=Bovin	O9=Chameau	O 1=Chien
O 2=Mouton	O 10=Rongeurs (lapin), préciser ____________	O 2=Rongeurs (lapin), préciser ____________
O 3=Âne	O 11=Chèvre	O 3=Chat
O 4= Volaille (i.e. poulet, canard, pigeon, oie, dinde), préciser ____________	O 12=Cheval	O 4=PAS D'ANIMAUX DOMESTIQUES/DE COMPAGNIE
O 5=Zébu	O 13=PAS D'ANIMAUX DE FERME/DE BÉTAIL	O 5=Autre, préciser ____________
O 6=Cochon	O 14=Autre, préciser ____________	O 99=Ne sait pas
O 7=Poisson	O 99=Ne sait pas	O 98=Pas de réponse
O 8=Buffle	O 98=Pas de réponse	

30 Où les membres de ce ménage gardent-ils habituellement les animaux de ferme/le bétail et les animaux domestiques/de compagnie de ce ménage comme indiqué à la question 29 ?

Instruction : Indiquer un code par catégorie uniquement.

Animaux de ferme/bétail	Animaux domestiques/de compagnie
O 1=Étable/Cage/Bassin d'eau sur la propriété de l'habitation (arrière-cour/cour)<br>O 2=Étable/Cage/Bassin d'eau à l'extérieur de l'habitation dans la communauté<br>O 3=Étable/Cage/Bassin d'eau à l'extérieur de la communauté<br>O 4=Surface libre/Eaux de surface/Eau de source sur la propriété de l'habitation (habitation, arrière-cour/cour)<br>O 5=Surface libre/Eaux de surface/Eau de source à l'extérieur de l'habitation dans la communauté<br>O 6=Surface libre/Eaux de surface/Eau de source à l'extérieur de la communauté<br>O 7=Pas applicable (nulle part /pas d'animaux de ferme/bétail)<br>O 8=Autre, préciser __________<br>O 99=Ne sait pas<br>O 98=Pas de réponse	O 1=Cage/Chaîne proche de l'habitation (arrière-cour/cour)<br>O 2= Cage/Chaîne à l'extérieur de l'habitation dans la communauté<br>O 3= Cage/Chaîne à l'extérieur de la communauté (public)<br>O 4=Surface libre proche de l'habitation (dans la maison, arrière-cour/cour)<br>O 5=Surface libre à l'extérieur de l'habitation dans la communauté<br>O 6=Surface libre à l'extérieur de la communauté<br>O 7=Pas applicable (nulle part/pas d'animaux domestiques/de compagnie)<br>O 8=Autre, préciser __________<br>O 99=Ne sait pas<br>O 98=Pas de réponse

31 Quelle est l'importance de l'élevage que les membres de ce ménage pratiquent ?

O 1=Pour leurs propres besoins («élevage d'arrière-cour»)
O 2=Pas applicable (pas d'élevage)
O 3=3=Demande massive/industrielle («élevage intensif»)
O 4=Autre, préciser __________
O 99=Ne sait pas
O 98=Pas de réponse

For peer review only - http://bmjopen.bmj.com/site/about/guidelines.xhtml

32 Quel approvisionnement en eau les membres de ce ménage utilisent-ils pour fournir de l'eau aux animaux de ferme/au bétail et aux animaux domestiques/de compagnie de ce ménage comme indiqué à la question 29 ?
Instruction : Indiquer un code par catégorie uniquement.

Animaux de ferme/bétail	Animaux domestiques/de compagnie
Code ____________	Code ____________

[1] Eau du robinet (à l'intérieur de l'habitation ; privée)
[2] Eau du robinet (à l'extérieur de l'habitation : cour/arrière-cour ; privée)
[3] Eau du robinet (à l'extérieur de l'habitation ; publique/partagée)
[4] Borne-fontaine/eau courante (à l'intérieur de l'habitation ; privée)
[5] Borne-fontaine/eau courante (à l'extérieur de l'habitation : cour/arrière-cour ; privée)
[6] Borne-fontaine/eau courante (à l'extérieur de l'habitation ; publique/partagée)
[7] Camion-citerne (public ; partagé)
[8] Eaux de surface (canal, canal d'irrigation)
[9] Eaux de surface (lac, étang, rivière, ruisseau)
[10] Eau de source
[11] Eau de pluie
[12] Puits/Puits de forage (en plein air/non couvert/non protégé)
[13] Puits/Puits de forage (couvert/protégé)
[14] Pas applicable (pas d'animaux de ferme/bétail; pas d'animaux domestiques/de compagnie)
[15] Autre, préciser ____________________ [99] Ne sait pas [98] Pas de réponse

33 Les membres de ce ménage se lavent-ils habituellement les mains après avoir nourri, donné de l'eau et nettoyé l'étable/la cage/le bassin d'eau des animaux de ferme/du bétail de ce ménage comme indiqué à la question 29 ?
Instruction : Indiquer un code par catégorie uniquement.

Animaux de ferme/bétail	Animaux domestiques/de compagnie
Code ____________	Code ____________

[1] Lavent les mains avec du savon et de l'eau
[2] Lavent les mains avec de l'eau uniquement
[3] Frottent les mains avec des feuilles/de la paille/de l'herbe
[4] Frottent les mains avec du sable
[5] Frottent les mains avec un chiffon/du tissu
[6] Ne lavent pas, pas besoin/pas sale
[7] Ne lavent pas, rien pour laver les mains
[8] Autre, préciser __________
[9] Pas applicable (pas d'animaux de ferme/bétail ; pas d'animaux domestiques/de compagnie
[99] Ne sait pas [98] Pas de réponse

CULTURES AGRICOLES

34 Quel genre de cultures les membres de ce ménage font-ils pousser/cultivent-ils ?
Instruction : Cocher plusieurs cases si nécessaire ; il peut y avoir plus d'une réponse.

- O 1=Café
- O 2=Riz
- O 3=Fruits (cultivés sur arbustes/arbre)
- O 4=Fruits (cultivés au sol)
- O 5=Légumes/salade (cultivés sur arbustes/arbre)
- O 6=Légumes/salade (cultivés au sol)
- O 7=Céréales (blé, orge, avoine, maïs)
- O 8=Thé
- O 9=Noix de cajou
- O 10=Tabac
- O 11=Canne à Sucre
- O 12=Chad/Chat
- O 13=Bees/Honey
- O 14=Coton
- O 15=PAS DE CULTURES
- O 16=Autre, préciser __________
- O 99=Ne sait pas
- O 98=Pas de réponse

35 Où les champs/terres agricoles des membres de ce ménage se situent-ils/elles?

- O 1=On the property of the house (i.e. back-/courtyard)
- O 4=Outside the house in the community
- O 2=Outside the community
- O 5=Not applicable (no crop farming)
- O 3=Other, specify __________
- O 99=Don't know
- O 98=No response

For peer review only - http://bmjopen.bmj.com/site/about/guidelines.xhtml

36 Quelle est l'importance des cultures des membres de ce ménage?

- O 1=Leurs propres besoins (« agriculture d'arrière-cour »)
- O 2=2=Pas applicable (pas de cultures)
- O 3=Demande massive/industrielle (agriculture intensive)
- O 4=Autre, préciser __________
- O 99=Ne sait pas
- O 98= Pas de réponse

37 Les membres de ce ménage utilisent-ils habituellement des herbicides, pesticides et engrais pour les cultures indiquées à la question 34?

Instruction : Indiquer un code par catégorie uniquement.

Herbicides	Pesticides	Engrais
O 1=Oui, préciser l'herbicide ______________	O 1=Oui, préciser le pesticide ______________	O 1=Oui, préciser l'engrais ______________
O 2=Pas applicable (pas de cultures)	O 2=Pas applicable (pas de cultures)	O 2=Pas applicable (pas de cultures)
O 3=Pas d'utilisation des herbicides	O 3=Pas d'utilisation des pesticides	O 3=Pas d'utilisation des engrais
O 99=Ne sait pas	O 99=Ne sait pas	O 99=Ne sait pas
O 98=Pas de réponse	O 98=Pas de réponse	O 98=Pas de réponse

38 Les membres de ce ménage se lavent-ils les mains après avoir travaillé dans les terres agricoles pour les cultures indiquées à la question 34 ?

- O 1=Oui, se lavent les mains avec du savon et de l'eau
- O 2=Oui, se frottent les mains avec des feuilles/de la paille/de l'herbe
- O 3=Oui, se frottent les mains avec des chiffons/du tissu
- O 4=Pas applicable (pas de cultures)
- O 5=Oui, se lavent les mains avec de l'eau uniquement
- O 6=Oui, se frottent les mains avec du sable
- O 7=Non, ne se lavent pas les mains
- O 8=Autre, préciser __________
- O 99=Ne sait pas
- O 98= Pas de réponse

For peer review only

Site ___ ___ ___ Sous-zone ___ ___ ___ Numéro d'habitation ___ ___ ___ ___ Identifiant de l'enquêteur ___ __

FORMULAIRE 4: ÉVALUATION DE L'UTILISATION GÉNÉRIQUE DES SOINS DE SANTÉ GLOBAUX, STRATIFIÉE SELON L'ÂGE ET LE SEXE
Partie A –Évaluation de l'utilisation *générique* des soins de santé

Instructions pour l'enquêteur

- Compléter ce formulaire uniquement si la personne interrogée a consenti à participer (voir « Formulaire 2 — FORMULAIRE DE CONSENTEMENT ») ; la personne interrogée ainsi que définie pour cette enquête est un adulte (selon l'âge légal de majorité du pays), un membre du ménage et un décideur en ce qui concerne les soins de santé quotidiens et l'utilisation de soins de santé pour le ménage entier et ses membres ; il ne doit pas être, par exemple, un voisin ou un visiteur.
- Se référer à « Formulaire 3 — Partie A — Informations générales sur le ménage » comme référence pour obtenir le nombre total de membres du ménage ainsi que l'âge et le sexe de chaque membre du ménage.
- Recueillir les données de tous les membres du ménage que tous les membre du ménage soient présents ou non au moment de l'entretien ; ne recueillir et n'inscrire les données que des personnes présentes au moment de l'entretien fausserait les données.
- Un ménage est défini/les membres d'un ménage sont définis comme une personne ou un groupe de personnes avec ou sans lien de parenté qui vivent ensemble dans la même unité d'habitation, qui reconnaissent un adulte de sexe masculin ou féminin comme chef de ménage, qui partagent les mêmes installations domestiques, qui sont considérés comme constituant une unité, et qui subviennent à leurs besoins en termes de nourriture et des autres éléments essentiels pour vivre.
- « Site » et « sous-zone » doivent être un numéro à trois chiffres, « numéro d'habitation » un numéro à quatre chiffres, et « identifiant de l'enquêteur » un numéro à deux chiffres (utiliser les listes préparées individuellement pour chaque site pour obtenir les codes) ; saisir « date » au format JJ/MM/AAAA et « heure » au format HH:MM et entourer MATIN ou APRÈS-MIDI. «Étiquette de l'étude» doit être une combinaison d'un code «site» à trois chiffres, d'un code «sous-zone» à trois chiffres et d'un code «numéro d'habitation» à quatre chiffres; le « identifiant de l'enquêteur » doit être un numéro à trois chiffres.
- Abréviation : EDS = Établissement de santé public/privé primaire/secondaire/tertiaire.

MEMBRES DU MÉNAGE

1 Indiquer le nombre total de membres du ménage de cette habitation (au jour de la visite).
Instructions : Le nombre total de membres du ménage doit être indiqué à l'aide d'un numéro à 2 chiffres ; voir également « Formulaire 3 — Partie A — INFORMATIONS GÉNÉRALES SUR LE MÉNAGE ».

Nombre total de membres du ménage : ____ ____

2 Indiquer le nombre de membres FÉMININS du ménage pour chacun des groupes d'âge suivants :
Instructions : Le nombre total de membres féminins du ménage par groupe d'âge doit être indiqué à l'aide d'un numéro à 2 chiffres ; voir également « Formulaire 3 — Partie A — INFORMATIONS GÉNÉRALES SUR LE MÉNAGE ».

<2 ans : ____ ____ ≥2 à <5 ans : ____ ____ ≥5 à <15 ans : ____ ____ ≥15 ans : ____ ____

3 Indiquer le nombre de membres MASCULINS du ménage pour chacun des groupes d'âge suivants :
Instructions : Le nombre total de membres masculins du ménage par groupe d'âge doit être indiqué à l'aide d'un numéro à 2 chiffres ; voir également « Formulaire 3 — Partie A — INFORMATIONS GÉNÉRALES SUR LE MÉNAGE ».

<2 ans : ____ ____ ≥2 à <5 ans : ____ ____ ≥5 à <15 ans : ____ ____ ≥15 ans : ____ ____

COMPORTEMENT DE RECHERCHE DE SOINS DE SANTÉ

4 Indiquer où les membres féminins/masculins du foyer d'âges <2 ans/≥2 à <5 ans/≥5 à <15 ans/≥15 ans recherchent habituellement des soins de santé pour les signes/symptômes suivants. Merci d'attribuer une note entre 1 et 4.

Instructions :
- Compléter la question 4 séparément pour les membres du ménage féminins et masculins d'âge <2 ans, ≥2 à <5 ans, ≥5 à <15 ans et ≥15 ans et entoure le sexe et le groupe d'âge pour lequel Q4 a été completée; passer la question 4 s'il n'y a aucun membre féminin/masculin du ménage d'âge <2 ans/ ≥2 à <5 ans/ ≥5 à <15 ans/ ≥15 ans.
- Indiquer une réponse pour chaque choix de soins de santé pour chaque signe/symptôme; attribuer une note de 1 à 4 (1=1er choix; 2=2ème choix; 3=3ème choix; 4=non choisi); indiquer un code s'il y a lieu en utilisant les listes préparées (utiliser listes préparées individuellement pour chaque site pour obtenir les codes); cocher «Ne sait pas» s'il y a lieu.

For peer review only - http://bmjopen.bmj.com/site/about/guidelines.xhtml

Signe/Symptôme	Choix de soins de santé									
	EDS SETA		Autre EDS		Médecin	Pharmacie	Guérisseur traditionnel	Nulle part Auto-médication	Nulle part Rien	Ne sait pas
	Note	Code	Note	Code	Note	Note	Note	Note	Note	Cocher
**1** [**A**] Fièvre	○=1<br>○=2<br>○=3<br>○=4		○=1<br>○=2<br>○=3<br>○=4		○=1<br>○=2<br>○=3<br>○=4	○=1<br>○=2<br>○=3<br>○=4	○=1<br>○=2<br>○=3<br>○=4	○=1<br>○=2<br>○=3<br>○=4	○=1<br>○=2<br>○=3<br>○=4	○
**2** Fièvre <3 jours (continus)	○=1<br>○=2<br>○=3<br>○=4		○=1<br>○=2<br>○=3<br>○=4		○=1<br>○=2<br>○=3<br>○=4	○=1<br>○=2<br>○=3<br>○=4	○=1<br>○=2<br>○=3<br>○=4	○=1<br>○=2<br>○=3<br>○=4	○=1<br>○=2<br>○=3<br>○=4	○
**3** Fièvre ≥3 jours (continus)	○=1<br>○=2<br>○=3<br>○=4		○=1<br>○=2<br>○=3<br>○=4		○=1<br>○=2<br>○=3<br>○=4	○=1<br>○=2<br>○=3<br>○=4	○=1<br>○=2<br>○=3<br>○=4	○=1<br>○=2<br>○=3<br>○=4	○=1<br>○=2<br>○=3<br>○=4	○
**4** Frissons Tremblements	○=1<br>○=2<br>○=3<br>○=4		○=1<br>○=2<br>○=3<br>○=4		○=1<br>○=2<br>○=3<br>○=4	○=1<br>○=2<br>○=3<br>○=4	○=1<br>○=2<br>○=3<br>○=4	○=1<br>○=2<br>○=3<br>○=4	○=1<br>○=2<br>○=3<br>○=4	○
**5** Convulsion	○=1<br>○=2<br>○=3<br>○=4		○=1<br>○=2<br>○=3<br>○=4		○=1<br>○=2<br>○=3<br>○=4	○=1<br>○=2<br>○=3<br>○=4	○=1<br>○=2<br>○=3<br>○=4	○=1<br>○=2<br>○=3<br>○=4	○=1<br>○=2<br>○=3<br>○=4	○
**6** Perte de poids	○=1<br>○=2<br>○=3<br>○=4		○=1<br>○=2<br>○=3<br>○=4		○=1<br>○=2<br>○=3<br>○=4	○=1<br>○=2<br>○=3<br>○=4	○=1<br>○=2<br>○=3<br>○=4	○=1<br>○=2<br>○=3<br>○=4	○=1<br>○=2<br>○=3<br>○=4	○
**7** Déshydratation	○=1<br>○=2<br>○=3<br>○=4		○=1<br>○=2<br>○=3<br>○=4		○=1<br>○=2<br>○=3<br>○=4	○=1<br>○=2<br>○=3<br>○=4	○=1<br>○=2<br>○=3<br>○=4	○=1<br>○=2<br>○=3<br>○=4	○=1<br>○=2<br>○=3<br>○=4	○
**8** Malaise Faiblesse/ Fatigue	○=1<br>○=2<br>○=3<br>○=4		○=1<br>○=2<br>○=3<br>○=4		○=1<br>○=2<br>○=3<br>○=4	○=1<br>○=2<br>○=3<br>○=4	○=1<br>○=2<br>○=3<br>○=4	○=1<br>○=2<br>○=3<br>○=4	○=1<br>○=2<br>○=3<br>○=4	○
**9** Douleurs (muscles/articulations /os)	○=1<br>○=2<br>○=3<br>○=4		○=1<br>○=2<br>○=3<br>○=4		○=1<br>○=2<br>○=3<br>○=4	○=1<br>○=2<br>○=3<br>○=4	○=1<br>○=2<br>○=3<br>○=4	○=1<br>○=2<br>○=3<br>○=4	○=1<br>○=2<br>○=3<br>○=4	○
**10** Maux de tête	○=1<br>○=2<br>○=3<br>○=4		○=1<br>○=2<br>○=3<br>○=4		○=1<br>○=2<br>○=3<br>○=4	○=1<br>○=2<br>○=3<br>○=4	○=1<br>○=2<br>○=3<br>○=4	○=1<br>○=2<br>○=3<br>○=4	○=1<br>○=2<br>○=3<br>○=4	○
**11** Vertiges/ Confusion Perte de connaissance	○=1<br>○=2<br>○=3<br>○=4		○=1<br>○=2<br>○=3<br>○=4		○=1<br>○=2<br>○=3<br>○=4	○=1<br>○=2<br>○=3<br>○=4	○=1<br>○=2<br>○=3<br>○=4	○=1<br>○=2<br>○=3<br>○=4	○=1<br>○=2<br>○=3<br>○=4	○
**12** Troubles de la pression artérielle	○=1<br>○=2<br>○=3<br>○=4		○=1<br>○=2<br>○=3<br>○=4		○=1<br>○=2<br>○=3<br>○=4	○=1<br>○=2<br>○=3<br>○=4	○=1<br>○=2<br>○=3<br>○=4	○=1<br>○=2<br>○=3<br>○=4	○=1<br>○=2<br>○=3<br>○=4	○
**13** Troubles cardiaques	○=1<br>○=2<br>○=3<br>○=4		○=1<br>○=2<br>○=3<br>○=4		○=1<br>○=2<br>○=3<br>○=4	○=1<br>○=2<br>○=3<br>○=4	○=1<br>○=2<br>○=3<br>○=4	○=1<br>○=2<br>○=3<br>○=4	○=1<br>○=2<br>○=3<br>○=4	○

For peer review only

For peer review only - http://bmjopen.bmj.com/site/about/guidelines.xhtml

Signe/Symptôme	Choix de soins de santé									
	EDS SETA		Autre EDS		Médecin	Pharmacie	Guérisseur traditionnel	Nulle part Auto-médication	Nulle part Rien	Ne sait pas
	Note	Code	Note	Code	Note	Note	Note	Note	Note	Cocher
**14** Nausée Vomissements	O=1<br>O=2<br>O=3<br>O=4		O=1<br>O=2<br>O=3<br>O=4		O=1<br>O=2<br>O=3<br>O=4	O=1<br>O=2<br>O=3<br>O=4	O=1<br>O=2<br>O=3<br>O=4	O=1<br>O=2<br>O=3<br>O=4	O=1<br>O=2<br>O=3<br>O=4	O
**15** Diarrhée (liquide/sanglante/ mucopurulente)	O=1<br>O=2<br>O=3<br>O=4		O=1<br>O=2<br>O=3<br>O=4		O=1<br>O=2<br>O=3<br>O=4	O=1<br>O=2<br>O=3<br>O=4	O=1<br>O=2<br>O=3<br>O=4	O=1<br>O=2<br>O=3<br>O=4	O=1<br>O=2<br>O=3<br>O=4	O
**16** Douleur abdominale Douleur intestinale	O=1<br>O=2<br>O=3<br>O=4		O=1<br>O=2<br>O=3<br>O=4		O=1<br>O=2<br>O=3<br>O=4	O=1<br>O=2<br>O=3<br>O=4	O=1<br>O=2<br>O=3<br>O=4	O=1<br>O=2<br>O=3<br>O=4	O=1<br>O=2<br>O=3<br>O=4	O
**17** Perte de sang/ Hémorragie (interne)	O=1<br>O=2<br>O=3<br>O=4		O=1<br>O=2<br>O=3<br>O=4		O=1<br>O=2<br>O=3<br>O=4	O=1<br>O=2<br>O=3<br>O=4	O=1<br>O=2<br>O=3<br>O=4	O=1<br>O=2<br>O=3<br>O=4	O=1<br>O=2<br>O=3<br>O=4	O
**18** Perte de sang/ Hémorragie (externe)	O=1<br>O=2<br>O=3<br>O=4		O=1<br>O=2<br>O=3<br>O=4		O=1<br>O=2<br>O=3<br>O=4	O=1<br>O=2<br>O=3<br>O=4	O=1<br>O=2<br>O=3<br>O=4	O=1<br>O=2<br>O=3<br>O=4	O=1<br>O=2<br>O=3<br>O=4	O
**19** Éternuements Nez qui coule	O=1<br>O=2<br>O=3<br>O=4		O=1<br>O=2<br>O=3<br>O=4		O=1<br>O=2<br>O=3<br>O=4	O=1<br>O=2<br>O=3<br>O=4	O=1<br>O=2<br>O=3<br>O=4	O=1<br>O=2<br>O=3<br>O=4	O=1<br>O=2<br>O=3<br>O=4	O
**20** Toux	O=1<br>O=2<br>O=3<br>O=4		O=1<br>O=2<br>O=3<br>O=4		O=1<br>O=2<br>O=3<br>O=4	O=1<br>O=2<br>O=3<br>O=4	O=1<br>O=2<br>O=3<br>O=4	O=1<br>O=2<br>O=3<br>O=4	O=1<br>O=2<br>O=3<br>O=4	O
**21** Respiration rapide/ essoufflement	O=1<br>O=2<br>O=3<br>O=4		O=1<br>O=2<br>O=3<br>O=4		O=1<br>O=2<br>O=3<br>O=4	O=1<br>O=2<br>O=3<br>O=4	O=1<br>O=2<br>O=3<br>O=4	O=1<br>O=2<br>O=3<br>O=4	O=1<br>O=2<br>O=3<br>O=4	O
**22** Difficultés respiratoires graves	O=1<br>O=2<br>O=3<br>O=4		O=1<br>O=2<br>O=3<br>O=4		O=1<br>O=2<br>O=3<br>O=4	O=1<br>O=2<br>O=3<br>O=4	O=1<br>O=2<br>O=3<br>O=4	O=1<br>O=2<br>O=3<br>O=4	O=1<br>O=2<br>O=3<br>O=4	O
**23** Inflammation de la peau (éruptions cutanées/ rougeurs/pus/ démangeaisons)	O=1<br>O=2<br>O=3<br>O=4		O=1<br>O=2<br>O=3<br>O=4		O=1<br>O=2<br>O=3<br>O=4	O=1<br>O=2<br>O=3<br>O=4	O=1<br>O=2<br>O=3<br>O=4	O=1<br>O=2<br>O=3<br>O=4	O=1<br>O=2<br>O=3<br>O=4	O
**24** Gonflement/ Œdème	O=1<br>O=2<br>O=3<br>O=4		O=1<br>O=2<br>O=3<br>O=4		O=1<br>O=2<br>O=3<br>O=4	O=1<br>O=2<br>O=3<br>O=4	O=1<br>O=2<br>O=3<br>O=4	O=1<br>O=2<br>O=3<br>O=4	O=1<br>O=2<br>O=3<br>O=4	O
**25** Jaunisse	O=1<br>O=2<br>O=3<br>O=4		O=1<br>O=2<br>O=3<br>O=4		O=1<br>O=2<br>O=3<br>O=4	O=1<br>O=2<br>O=3<br>O=4	O=1<br>O=2<br>O=3<br>O=4	O=1<br>O=2<br>O=3<br>O=4	O=1<br>O=2<br>O=3<br>O=4	O

For peer review only

For peer review only - http://bmjopen.bmj.com/site/about/guidelines.xhtml

Site ___ ___ ___ Sous-zone ___ ___ ___ Numéro d'habitation ___ ___ ___ ___ Identifiant de l'enquêteur ___ __

FORMULAIRE 4: ÉVALUATION DE L'UTILISATION GÉNÉRIQUE DES SOINS DE SANTÉ GLOBAUX, STRATIFIÉE SELON L'ÂGE ET LE SEXE

Partie B – Facteurs associés aux soins de santé/Vaccination/Perception de la maladie

DÉPLACEMENT À L'ÉTABLISSEMENT DE SANTÉ

1 Au cas où un établissement de santé (EDS) a été noté comme 1er choix de soins à la question 4A (fièvre), quel est habituellement le moyen de transport principal, le temps de trajet (selon le moyen de transport), le coût du trajet (selon le moyen de transport) et la distance du trajet (selon le moyen de transport) pour s'y rendre ?

Instructions : Indiquer uniquement un code par catégorie, sexe ainsi que groupe d'âge. Passer cette question s'il n'y a pas de membre féminin/masculin d'âge <2 ans/≥2 à <5 ans/≥5 à <15 ans/≥15 ans dans ce ménage (voir également questions 2 et 3 de ce formulaire Partie A). Passer cette question si aucun EDS n'a été noté comme 1er choix de soins à la question 4A pour les membres masculins/féminins d'âge <2 ans/≥2 à <5 ans/≥5 à <15 ans/≥15 ans. Indiquer le temps de trajet en minute(s) [min]/ heure(s) [h], le coût total en USD, y compris les cadeaux éventuels, les aides financières et les dons, et la distance totale en mètre(s) [m]/kilomètre(s) [km].

Membre féminin				Membre masculin			
<2 ans	≥2 à <5 ans	≥5 à <15 ans	≥15 ans	<2 ans	≥2 à <5 ans	≥5 à <15 ans	≥15 ans
**1.1 Moyen de transport**							
Code ____	Code ____	Code ____	Code ____	Code ____	Code ____	Code ____	Code ____
[1] Private transportation (i.e. motorbike, car, bus)<br>[2] Public transportation (i.e. motorbike, car, bus)				[3] Walking<br>[4] Bicycle	[5] Other, specify ________<br>[99] Don't know		[98] No response
**1.2 Temps de trajet**							
Code ____	Code ____	Code ____	Code ____	Code ____	Code ____	Code ____	Code ____
[1] <30 min<br>[2] ≥30 à <60 min		[3] ≥1.0 à <2.0 h<br>[4] ≥2.0 à <3.0 h		[5] ≥3.0 à <4.0 h<br>[6] ≥4.0h		[99] Ne sait pas<br>[98] Pas de réponse	
**1.3 Coût du transport**							
Code ____	Code ____	Code ____	Code ____	Code ____	Code ____	Code ____	Code ____
[1] <1.0 USD<br>[2] ≥1.0 à <3.0 USD		[3] ≥3.0 à <5.0 USD<br>[4] ≥5.0 à <10.0 USD		[5] ≥10.0 USD		[99] Ne sait pas<br>[98] Pas de réponse	
**1.4 Distance du trajet**							
Code ____	Code ____	Code ____	Code ____	Code ____	Code ____	Code ____	Code ____
[1] <500 m<br>[2] ≥500 m à <1.0 km		[3] ≥1.0 à <2.0 km<br>[4] ≥2.0 à <3.0 km		[5] ≥3.0 à <4.0 km<br>[6] ≥4.0 km		[99] Ne sait pas<br>[98] Pas de réponse	

ASSURANCE

2 Pour quels membres de ce ménage l'assurance couvre-t-elle les dépenses de santé ?

Instructions : Indiquer un code par catégorie. Passer cette question s'il n'y a aucun membre d'âge <2 ans/≥2 à <5 ans/≥5 à <15 ans/≥15 ans dans ce ménage (voir questions 2 et 3 de ce formulaire partie A).

<2 ans	≥2 à <5 ans	≥5 à <15 ans	≥15 ans
Code ____________	Code ____________	Code ____________	Code ____________
[1] Oui	[2] Non<br>[99] Ne sait pas	[98] Pas de réponse	

3 Quels types de dépenses de santé sont habituellement couverts par l'assurance pour les membres de ce ménage ?

Instructions : Indiquer uniquement un code par catégorie et par groupe d'âge. Passer cette question s'il n'y a aucun membre d'âge <2 ans/≥2 à <5 ans/≥5 à <15 ans/≥15 ans dans ce ménage (voir également les questions 2 et 3 de ce formulaire partie

For peer review only - http://bmjopen.bmj.com/site/about/guidelines.xhtml

A). Passer cette question s'il n'y a aucune assurance qui couvre les dépenses de santé des membres d'âge <2 ans/≥2 à <5 ans/≥5 à <15 ans/≥15 ans.

	<2 ans	≥2 à <5 ans	≥5 à <15 ans	≥15 ans
	[1] Oui	[2] Non	[99] Ne sait pas	[98] Pas de réponse
**1** Inscription	Code _______	Code _______	Code _______	Code _______
**2** Consultation/Visite chez le médecin	Code _______	Code _______	Code _______	Code _______
**3** Examen physique - général	Code _______	Code _______	Code _______	Code _______
**4** Examen physique – spécifique, préciser_______	Code _______	Code _______	Code _______	Code _______
**5** Diagnostics - diagnostics généraux de laboratoire	Code _______	Code _______	Code _______	Code _______
**6** Diagnostics – diagnostics spécifiques de laboratoire, préciser__________	Code _______	Code _______	Code _______	Code _______
**7** Diagnostics Imagerie générale	Code _______	Code _______	Code _______	Code _______
**8** Diagnostics Imagerie spécifique, préciser__________	Code _______	Code _______	Code _______	Code _______
**9** Diagnostics Autre, préciser________	Code _______	Code _______	Code _______	Code _______
**10** Traitement Médication générale	Code _______	Code _______	Code _______	Code _______
**11** Traitement Médication spécifique, préciser________	Code _______	Code _______	Code _______	Code _______
**12** Traitement Autre médication, préciser__________	Code _______	Code _______	Code _______	Code _______
**13** Traitement Interventions (autres que chirurgie), préciser_______	Code _______	Code _______	Code _______	Code _______
**14** Traitement Chirurgie	Code _______	Code _______	Code _______	Code _______
**15** Hospitalisation <7 jours	Code _______	Code _______	Code _______	Code _______
**16** Hospitalisation ≥7 jours	Code _______	Code _______	Code _______	Code _______
**17** Services d'urgence	Code _______	Code _______	Code _______	Code _______
**18** Soins prénatals	Code _______	Code _______	Code _______	Code _______
**19** Soins postnatals	Code _______	Code _______	Code _______	Code _______
**20** Médecine préventive	Code _______	Code _______	Code _______	Code _______
**21** Vaccination	Code _______	Code _______	Code _______	Code _______
**22** Soins à domicile (personnes âgées, handicapés, malades chroniques/à long terme)	Code _______	Code _______	Code _______	Code _______
**23** Thérapie de soutien (physiothérapie, ergothérapie, réhabilitation)	Code _______	Code _______	Code _______	Code _______

For peer review only - http://bmjopen.bmj.com/site/about/guidelines.xhtml

ETHNOGRAPHIE

4 À quelle religion/groupe ethnique/tribu appartiennent les membres de ce ménage ?

Instructions : Donner une réponse par groupe d'âge uniquement. Passer cette question s'il n'y a aucun membre d'âge>2 ans/≥2 à <5 ans/≥5 à <15 ans/≥15 ans dans ce ménage (voir également questions 2 et 3 de ce formulaire Partie A).

	<2 ans	≥2 to <5 ans	≥5 to <15 ans	≥15 ans
Religion/Groupe ethnique/Tribu	___________	___________	___________	___________
	O 99=Ne sait pas<br>O 98=Pas de réponse	O 99=Ne sait pas<br>O 98=Pas de réponse	O 99=Ne sait pas<br>O 98=Pas de réponse	O 99=Ne sait pas<br>O 98=Pas de réponse

5 La religion/le groupe ethnique/la tribu comme indiqué à la question 4 influence-t-elle/il les différents aspects de l'utilisation des soins de santé pour les membres de ce ménage ?

Instructions : Indiquer un code par catégorie et groupe d'âge uniquement. Passer cette question s'il y a un membre d'âge >2 ans/≥2 à <5 ans/≥5 à <15 ans/≥15 ans dans ce ménage (voir également questions 2 t 3 de ce formulaire Partie A).

	<2 ans	≥2 à <5 ans	≥5 to <15 ans	≥15 ans
	[1] Oui	[2] Non	[99] Ne sait pas	[98] pas réponse
**1** Visite dans un établissement de santé (hôpital, centre de soins, poste de santé)	Code _____	Code _______	Code _______	Code _____
**2** Visite chez un médecin	Code _____	Code _______	Code _______	Code _____
**3** Visite dans une pharmacie	Code _____	Code _______	Code _______	Code _____
**4** Visite chez un guérisseur traditionnel	Code _____	Code _______	Code _______	Code _____
**5** Décision d'automédication	Code _____	Code _______	Code _______	Code _____
**6** Décision de non traitement	Code _____	Code _______	Code _______	Code _____
**7** Examen physique - général	Code _____	Code _______	Code _______	Code _____
**8** Examen physique – spécifique, préciser________	Code _____	Code _______	Code _______	Code _____
**9** Prélèvements d'échantillons (sang, urine, selles, frottis, écouvillons, liquide cérébro-spinal)	Code _____	Code _______	Code _______	Code _____
**10** Diagnostics - diagnostics généraux de laboratoire	Code _____	Code _______	Code _______	Code _____
**11** Diagnostics – diagnostics spécifiques de laboratoire, préciser___________	Code _____	Code _______	Code _______	Code _____
**12** Diagnostics Imagerie générale	Code _____	Code _______	Code _______	Code _____
**13** Diagnostics Imagerie spécifique, préciser___________	Code _____	Code _______	Code _______	Code _____
**14** Diagnostics Autre, préciser_________	Code _____	Code _______	Code _______	Code _____
**15** Traitement Médication générale	Code _____	Code _______	Code _______	Code _____
**16** Traitement Médication spécifique, préciser_________	Code _____	Code _______	Code _______	Code _____
**17** Traitement Autre médication, préciser___________	Code _____	Code _______	Code _______	Code _____

For peer review only - http://bmjopen.bmj.com/site/about/guidelines.xhtml

**18** Traitement<br>Interventions (autres que chirurgie), préciser________	Code _____	Code _______	Code _______	Code _____
**19** Traitement<br>Chirurgie	Code _____	Code _______	Code _______	Code _____
**20** Hospitalisation<br><7 jours	Code _____	Code _______	Code _______	Code _____
**21** Hospitalisation<br>≥7 jours	Code _____	Code _______	Code _______	Code _____
**22** Services d'urgence	Code _____	Code _______	Code _______	Code _____
**23** Soins prénatals	Code _____	Code _______	Code _______	Code _____
**24** Soins postnatals	Code _____	Code _______	Code _______	Code _____
**25** Médecine préventive	Code _____	Code _______	Code _______	Code _____
**26** Vaccination	Code _____	Code _______	Code _______	Code _____
**27** Soins à domicile (personnes âgées, handicapés, malades chroniques/à long terme)	Code _____	Code _______	Code _______	Code _____
**28** Thérapie de soutien (physiothérapie, ergothérapie, réhabilitation)	Code _____	Code _______	Code _______	Code _____

VACCINATION

6 Comment les enfants de ce ménage ont-ils été nourris pendant les 6 premiers mois de leur vie ?

Instructions : Passer cette question s'il n'y a pas d'enfants de sexe masculin ou féminin dans ce ménage (voir également questions 2 et 3 de ce formulaire partie A).

O 1=Lait maternisé exclusivement
O 2=Lait maternisé et autres aliments, préciser ______
O 3=Allaitement et autres aliments, préciser ________
O 4=Lait maternisé et allaitement
O 5=Allaitement exclusivement
O 6=Autre, préciser _____
O 99=Ne sait pas
O 98=Pas de réponse

7 Quelle source d'approvisionnement en eau était-elle habituellement utilisée pour préparer le lait maternisé ou le lait maternisé et d'autres aliments comme indiqué à la question 6 pour les enfants de ce ménage ?

Instructions : Passer cette question s'il n'y a pas d'enfants dans ce ménage (voir également les questions 2 et 3 de ce formulaire Partie A).

O 1=Eau du robinet (à l'intérieur de l'habitation, privé)
O 2=Eau du robinet (à l'extérieur de l'habitation, arrière- cour/cour, privé)
O 3=Eau du robinet (à l'extérieur de l'habitation, public, partagé)
O 4=Borne-fontaine/Eau courante (à l'intérieur de l'habitation, privé)
O 5=Borne-fontaine/Eau courante (à l'extérieur de l'habitation, arrière- cour/cour, privé)
O 6=Borne-fontaine/Eau courante (à l'extérieur de l'habitation, public, partagé)
O 7=Camion-citerne (public, partagé)
O 8=Eau en bouteille
O 9=Eaux de surface (lac, étang, rivière, ruisseau)
O 10=Eaux de surface (canal, canal d'irrigation)
O 11=Eau de source
O 12=Eau de pluie
O 13=Puits/Puits de forage (ouvert/non couvert/non protégé)
O 14=Puits/Puits de forage (couvert/protégé)
O 15=NO FORMULA/OTHER FEEDS PREPARED
O 16=Autre, préciser _____________ O 99=Ne sait pas O 98=Pas de réponse

8 Est-ce que les enfants de ce ménage ont été vaccinés depuis leur naissance, y compris les vaccins de naissance ?

For peer review only - http://bmjopen.bmj.com/site/about/guidelines.xhtml

Instructions : Indiquer un code par catégorie et par enfant uniquement. Passer cette question s'il n'y a aucun enfant dans ce ménage (voir également les questions 2 et 3 de ce formulaire PartieA). Se référer à la question 9 « Formulaire 3 Partie A – Données démographiques/Informations générales sur le ménage » et indiquer les initiales/identificateur du/des membre(s).

Enfant 1<br>Initiales/Identifiant : ____	Enfant 2<br>Initiales/Identifiant : ____	Enfant 3<br>Initiales/Identifiant : ____	Enfant 4<br>Initiales/Identifiant : ____
[1] OUI, vacciné [5] Non, manque de vaccins [9] Non, EDS débordé [2] Non, parent/tuteur débordé [6] Non, enfant trop jeune/vieux [10] Non, enfant malade [3] Non, parent/tuteur a décidé que pas nécessaire de vaccination [7] Non, parent/tuteur a refusé/trop inquiet [11] Non, pas de carte [4] Non, ne sait pas où aller [8] Non, Autre, préciser ____________ [99] Don't know [98] No response			
**8.1 Diphtérie et tétanos et coqueluche et *Haemophilus influenza* et vaccin contre l'hépatite B / DTwPHibHepB** (par exemple 6/8, 10/12, 14/16 semaines)			
Code __________	Code __________	Code __________	Code __________
**8.2 Anatoxine tétanique (TT)** (par exemple, 1er contact, grossesse, 1, 6, 12/24 mois)			
Code __________	Code __________	Code __________	Code __________
**8.3 Anatoxine tétanique et diphtérique (TD)** (par exemple, grossesse avec un premier contact, 1, 6, 12 mois)			
Code __________	Code __________	Code __________	Code __________
**8.4 Tuberculose / BCG** (à la naissance)			
Code __________	Code __________	Code __________	Code __________
**8.5 Rotavirus / RV** (par exemple 6/8, 10/12, 16 semaines)			
Code __________	Code __________	Code __________	Code __________
**8.6 Rougeole / rougeole et rubéole (MR)** (par exemple 9, 15/18 mois)			
Code __________	Code __________	Code __________	Code __________
**8.7 Polio / VPO** (par exemple naissance, 6/8, 10/12, 14/16 semaines)			
Code __________	Code __________	Code __________	Code __________
**8.8 Pneumocoque / PCV** (par exemple 6/8, 10/12, 14/16 semaines)			
Code __________	Code __________	Code __________	Code __________
**8.9 Fièvre jaune (YF)** (par exemple 9 mois)			
Code __________	Code __________	Code __________	Code __________
**8.10 Autres vaccins**			
Préciser, __________	Préciser, __________	Préciser, __________	Préciser, __________
Préciser, __________	Préciser, __________	Préciser, __________	Préciser, __________
Préciser, __________	Préciser, __________	Préciser, __________	Préciser, __________
Préciser, __________	Préciser, __________	Préciser, __________	Préciser, __________

For peer review only - http://bmjopen.bmj.com/site/about/guidelines.xhtml

PERCEPTION DE LA MALADIE

9 Connaissance, perception, causes et prévention des maladies infectieuses communes

Maladie	**9.1 Maladie connue des membres du ménage ?** [1] Oui [2] Non [99] Ne sait pas [98] Pas de réponse	**9.2 Perception de la maladie par les membres du ménage** *Instruction :* Décrire brièvement si elle est connue.	**9.3 Causes de la maladie connue des membres du ménage ?** *Instructions :* Indiquer tous les codes (liste A) qui s'appliquent; plus d'une réponse est possible	**9.4 Mesures pour prévenir la maladie connues des membres du ménage ?** *Instructions :* Indiquer tous les codes (liste B) qui s'appliquent ; plus d'une réponse est possible.	**9.5 Mesures pour prévenir la maladie utilisées par les membres du foyer ?** *Instructions :* Indiquer tous les codes (liste B) qui s'appliquent ; il peut y avoir plus d'une réponse.
**A.** Fièvre typhoïde	Code ___________		Code ___________	Code ___________	Code ___________
**B.** Choléra	Code ___________		Code ___________	Code ___________	Code ___________
**C.** Paludisme	Code ___________		Code ___________	Code ___________	Code ___________
**D.** Grippe	Code ___________		Code ___________	Code ___________	Code ___________
**E.** Méningite	Code ___________		Code ___________	Code ___________	Code ___________
**F.** Hépatite E	Code ___________		Code ___________	Code ___________	Code ___________
**G.** Tuberculose	Code ___________		Code ___________	Code ___________	Code ___________
**H.** VIH/SIDA	Code ___________		Code ___________	Code ___________	Code ___________

Liste A: Causes possibles	Liste B: Mesures préventives possibles
[1] Consommation d'alcool<br>[2] Temps/sorcellerie	[1] Pas de consommation d'alcool<br>[2] Dormir sous une moustiquaire

For peer review only - http://bmjopen.bmj.com/site/about/guidelines.xhtml

[3] Piqûre de moustique/d'insecte<br>[4] Morsure de chien<br>[5] Boire de l'eau non traitée de surface/de source (lac, étang, rivière, ruisseau, canal, canal d'irrigation)<br>[6] Contact physique étroit/baiser/embrassade<br>[7] Manger des produits alimentaires crus comme des légumes/de la salade, des fruits, des produits laitiers, des œufs, de la viande ou du poisson<br>[8] Ne pas se laver les mains correctement en préparant la nourriture/les repas<br>[9] Ne pas se laver les mains correctement avant/après être allé aux toilettes<br>[10] Contact avec du sang humain (injection, transfusion, chirurgie)<br>[11] Consommation de sang animal cru<br>[12] Autre, préciser ____________________<br>[99] Ne sait pas<br>[98] Pas de réponse	[3] Utiliser des sprays insecticides/anti-moustiques<br>[4] Maintenir la maison et ses environs immédiats propres<br>[5] Avoir une pratique correcte de l'élimination des déchets humains et domestiques<br>[6] Éviter le contact rapproché avec des chiens<br>[7] Traiter l'eau (faire bouillir, filtration, désinfection solaire, sédimentation, chloration)<br>[8] Ne pas cuisiner/préparer la nourriture/les repas avec de l'eau non traitée<br>[9] Ne pas laver les produits alimentaires à consommer crus avec de l'eau non traitée<br>[10] Faire bouillir/chauffer à nouveau les restes/les aliments/repas préparés avant de les consommer<br>[11] Ne pas manger des produits alimentaires crus comme légumes/salade, fruits, produits laitiers, œufs, viande ou poisson<br>[12] Couvrir les produits alimentaires/repas<br>[13] Protéger les produits alimentaires/repas contre les mouches<br>[14] Éviter la nourriture/les boissons des vendeurs de rue<br>[15] Éviter le contact rapproché avec les personnes malades<br>[16] Pas de contact physique étroit/baisers/embrassades<br>[17] Se couvrir le nez/la bouche en éternuant/toussant<br>[18] Pas de contact/consommation de sang humain/animal<br>[19] Avoir une bonne hygiène des mains en préparant la nourriture/les repas (se laver les mains avec du savon et de l'eau, se laver les mains par frottement)<br>[20] Avoir une bonne hygiène des mains avant/après être allé aux toilettes (se laver les mains avec du savon et de l'eau, se laver les mains par frottement)<br>[21] Autre, préciser ____________________<br>[99] Ne sait pas<br>[98] Pas de réponse

For peer review only

For peer review only - http://bmjopen.bmj.com/site/about/guidelines.xhtml

10 Pour quels membres de ce ménage les mesures préventives listées à la questions 9.5 contre la fièvre typhoïde sont-elles utilisées ?
Instructions : Cocher plusieurs cases si nécessaire ; il peut y avoir plus d'une réponse.
O 1=<2 ans O 3=≥5 à <15 ans O 5=PAS UTILISÉ O 99=Ne sait pas
O 2=≥2 à < 5 ans O 4=≥15 ans O 98=Pas de réponse

11 Nommer les membres du ménage pour lesquels une fièvre typhoïde a été diagnostiquée dans les 3 derniers mois. Quels éléments de diagnostic ont été utilisés pour confirmer la fièvre typhoïde?
Instructions : Indiquer plusieurs codes si nécessaire pour chaque membre du ménage ; il peut y avoir plus d'une réponse.

Membre 1 Initiales/Identifiant: __	Membre 2 Initiales/Identifiant: __	Membre 3 Initiales/Identifiant: __	Membre 4 Initiales/Identifiant: __
[1] Hémoculture [3] Test sanguin/de Widal [5] Pas applicable (pas diagnostiqué avec la fièvre typhoïde)<br>[2] Coproculture [4] Autre test, préciser ______________ [99] Ne sait pas [98] Pas de réponse			
Code ___________	Code ___________	Code ___________	Code ___________

12 (Où les membres de ce ménage indiqués à la question 11 ont-ils été diagnostiqués pour la fièvre typhoïde?) Où les membres de ce ménage ont-ils recherché des soins de santé?
Instructions : Indiquer un code par membre du ménage uniquement.

Membre 1 Initiales/Identifiant: __	Membre 2 Initiales/Identifiant: __	Membre 3 Initiales/Identifiant: __	Membre 4 Initiales/Identifiant: __
[1] Établissement de santé, préciser _____________<br>[2] Médecin<br>[3] Nulle part, automédication<br>[4] Guérisseur traditionnel<br>[5] Pharmacien		[6] Nulle part, pas de recherche de soins<br>[7] Autre, préciser _____________<br>[8] Pas applicable<br>[99] Ne sait pas<br>[98] Pas de réponse	
Code ___________	Code ___________	Code ___________	Code ___________

13 (Les membres de ce ménage pour lesquels une fièvre typhoïde a été diagnostiquée comme indiqué à la question 11 ont-ils reçu un traitement?) Quel traitement ont-ils reçu?
Instructions : Cocher une réponse par membre du ménage uniquement.

Membre 1 Initiales/Identifiant: __	Membre 2 Initiales/Identifiant: __	Membre 3 Initiales/Identifiant: __	Membre 4 Initiales/Identifiant: __
O1=Traitement, préciser ________________<br>O 2=Pas applicable<br>O 99=Ne sait pas<br>O 98=Pas de réponse	O1=Traitement, préciser ________________<br>O 2=Pas applicable<br>O 99=Ne sait pas<br>O 98=Pas de réponse	O1=Traitement, préciser ________________<br>O 2=Pas applicable<br>O 99=Ne sait pas<br>O 98=Pas de réponse	O1=Traitement, préciser ________________<br>O 2=Pas applicable<br>O 99=Ne sait pas<br>O 98=Pas de réponse

For peer review only - http://bmjopen.bmj.com/site/about/guidelines.xhtml

Site ___ ___ ___ Sous-zone ___ ___ ___ Numéro d'habitation ___ ___ ___ ___ Identifiant de l'enquêteur ___ __

FORMULAIRE 5: ÉVALUATION DE L'UTILISATION RÉELLE DES SOINS DE SANTÉ GLOBAUX, STRATIFIÉE SELON L'ÂGE ET LE SEXE

Partie A: Évaluation de l'utilisation *réelle* des soins de santé

COMPORTEMENT DE RECHERCHE DE SOINS

1 Consigner ci-dessous l'apparition de signe(s) ou symptôme(s) (voir liste 1) et préciser où (voir liste 2) et comment le(s) membre(s) a/ont recherché les soins de santé durant les 3 derniers mois ?

Instructions : voir « formulaire 3- partie A-INFORMATIONS GENERALES SUR LE MENAGE » pour obtenir les initiaux/identifiants de chacun des membres du ménage. Indiquer seulement un signe/symptome par ligne. Commencer avec une nouvelle ligne pour tous nouveaux soins de santé sélectionnés pour chaque signe/symptome recurrent. Indiquer le nombre des apparitions de chaque signe/symptômes durant les 3 derniers mois. Si une différente option de soins de santé a été sélectionnée pour un signe/symptôme recurrent, indiquer cela dans une nouvelle ligne. Completer s'il vous plaît, un formulaire supplémentaire s'il y a eu plus de 20 signes/symptômes apparus durant les 3 derniers mois.

**Liste 1: Signe/Symptôme**		**Liste 2: Option de soins de santé**
[A] Fièvre<br>[B] Fièvre <3 jours (continus)<br>[C] Fièvre ≥3 jours (continus)<br>[D] Frissons/Tremblements<br>[E] Convulsion<br>[F] Perte de poids<br>[G] Déshydratation<br>[H] Malaise/Faiblees/Fatigue<br>[I] Douleurs (muscles/articulations/os)<br>[J] Maux de tête<br>[K] Vertiges/Confusion/ Perte de connaissance<br>[L] Troubles de la pression sanguine<br>[M] Troubles cardiaques	[N] Nausée/Vomissements<br>[O] Diarrhée (liquide/sanglante/mucopurulente)<br>[P] Douleur abdominale/Douleur intestinale<br>[Q] Perte de sang/Hémorragie (interne)<br>[R] Perte de sang/Hémorragie (externe)<br>[S] Éternuements Nez qui coule<br>[T] Toux<br>[U] Respiration rapide/Essoufflement<br>[V] Difficultés respiratoires graves<br>[W] Inflammation de la peau (éruptions cutanées/ rougeurs/pus/démangeaisons)<br>[X] Gonflement/Œdème<br>[Y] Jaunisse	[1] EDS-SETA, Code _______________<br>[2] Autre EDS Code _______________<br>[3] Médecin<br>[4] Pharmacie<br>[5] Guérisseur traditionnel<br>[6] Nulle part Auto-médication<br>[7] Nulle part Rien

**No.**	**Membre du ménage** Enregistrer les initiaux/identifiants du membre du ménage ; voir formulaire 3 – partie A- INFORMATIONS GENERALES SUR LE MENAGE »	**Signes/ Symptômes** Utiliser la Liste 1 pour sélectionner le code	**Options de soins de santé**		**Fréquence** Indiquer le nombre d'incidences pour le signe/symptôme listé
			Utiliser la Liste 2 pour sélectionner le code	EDS Code	
1					
2					
3					
4					
5					

For peer review only - http://bmjopen.bmj.com/site/about/guidelines.xhtml

6				
7				
8				
9				
10				
11				
12				
13				
14				
15				
16				
17				
18				
19				
20				

For peer review only

For peer review only - http://bmjopen.bmj.com/site/about/guidelines.xhtml

Appendix 4/Supplementary file 4: Geospatial random selection with application of simple, weighted-stratified sampling (examples of Ghana and Madagascar)

Figure 1 Geographic random points generated by ArcGIS for Asante Akim North and Central, Ghana

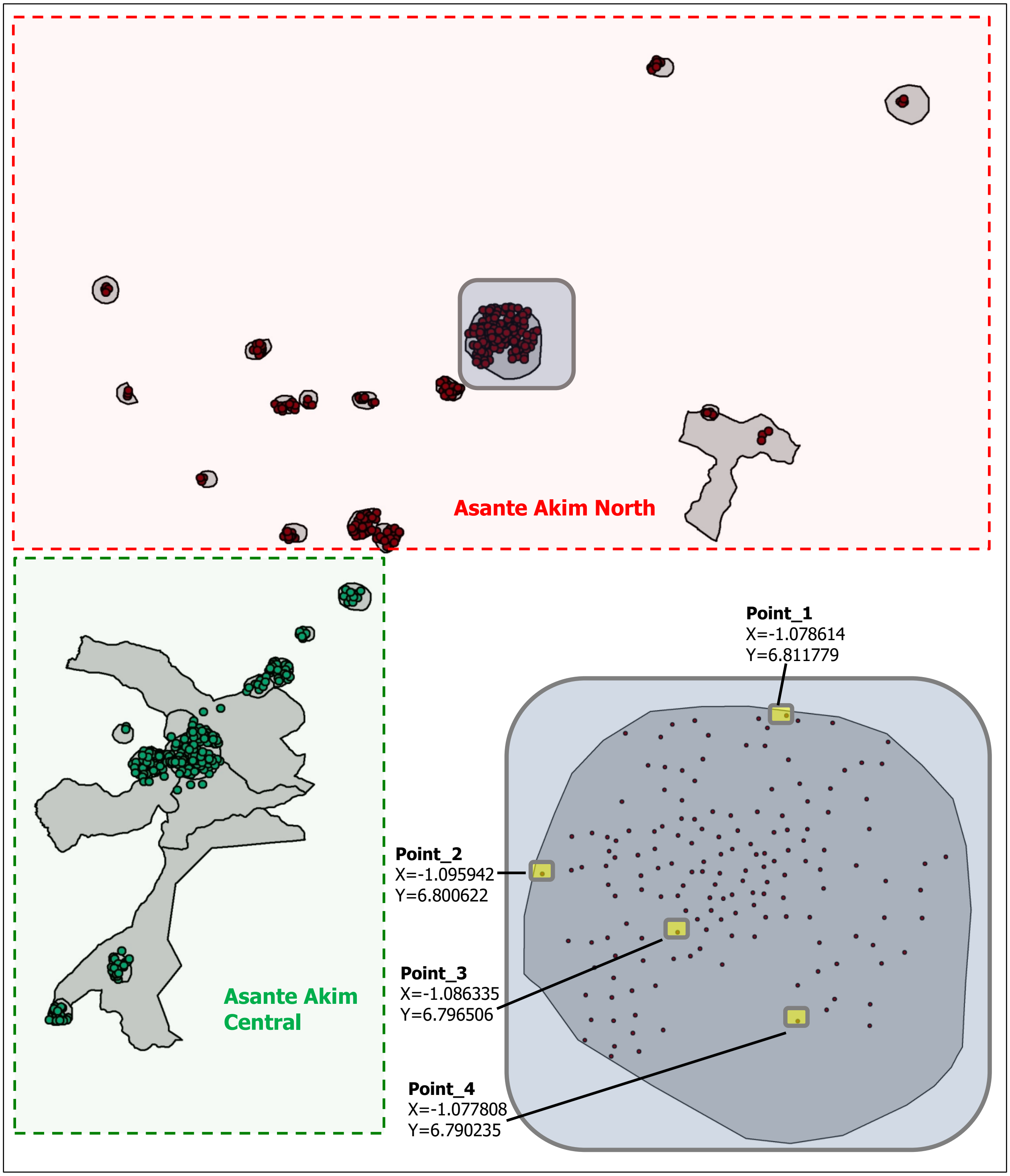

The study area of Asante Akim North (light red rectangle) and Asante Akim Central (light green rectangle) is highlighted in grey. The geographic random points are shown as red and green points for Asante Akim North and Asante Akim Central, respectively. The illustration at the bottom, right highlighted in light blue shows the enlarged area that is indicated as a small central light blue highlighted rectangle. The X- and Y-data of four geographic random points are given as examples; points are indicated as yellow highlighted rectangles. ArcGIS-generated geographic random points converted and visualized in Google Earth Pro® for Asante Akim North and Central, Ghana, can be shared on request.

Figure 2 Geographic random points generated by ArcGIS for Renivohitra/Antananarivo, Madagascar

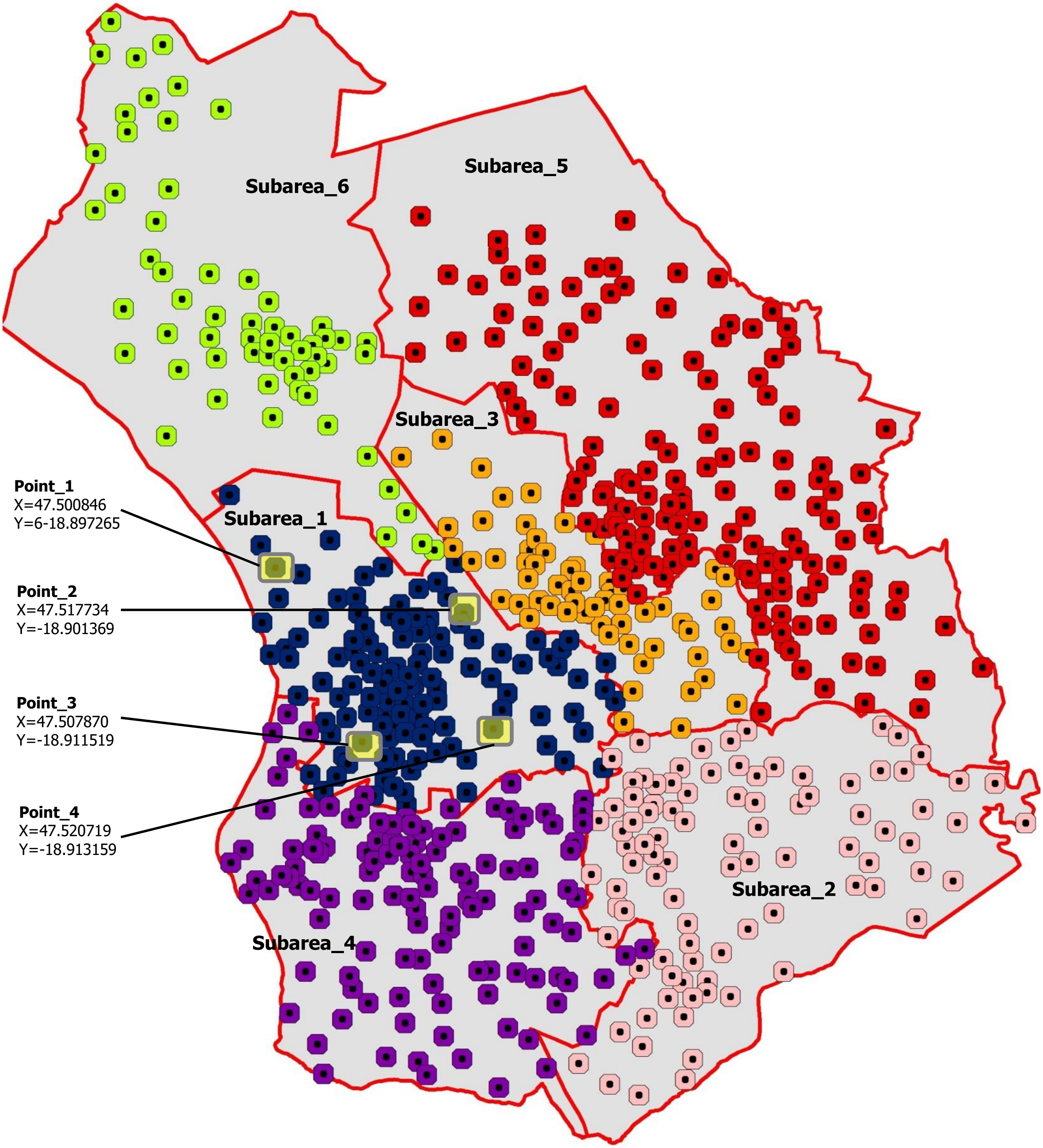

The study area of Renivohitra/Antananarivo, which is subdivided into six administrative subareas, is highlighted in grey. The geographic random points are shown as blue (subarea_1), pink (subarea_2), orange (subarea_3), purple (subarea_4), red (subarea_5) and green (subarea_6) points. The X- and Y-data of four geographic random points are given as examples; points are indicated as yellow highlighted rectangles. ArcGIS-generated geographic random points converted and visualized in Google Earth Pro® for Renivohitra/Antananarivo, Madagascar, can be shared on request.

Appendix 5/Supplementary file 5: System diagram of the data collection and data management platform: the Android application "HPA Collect" and the platform "HPA Web"

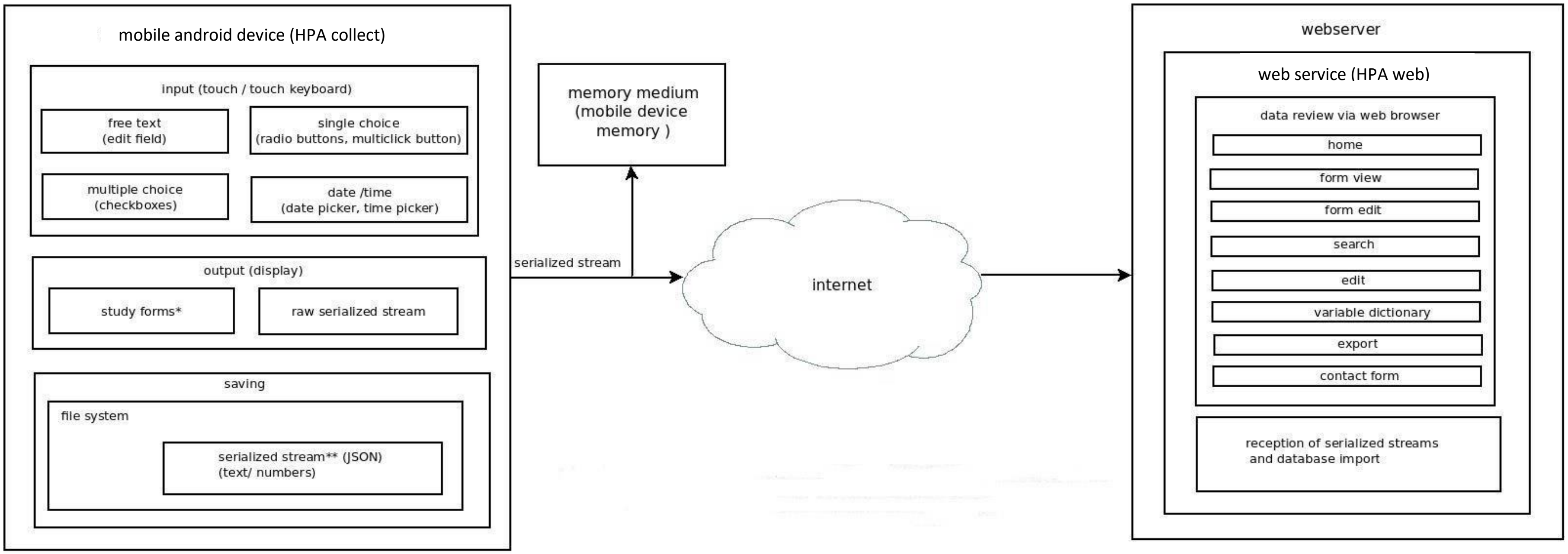

Note. *study forms: Form 1, Form 3 Part A to C, Form 4 Part A to B, Form 5; **serialized stream: two- or more-dimensional data (e.g. a table) is brought to a one-dimensional form; the application uses the JSON (Java Script Object Notation) format.

BMJ Open

The HPAfrica Study Protocol: Assessment of health behavior and population-based socio-economic, hygiene behavioral factors - a standardized repeated cross-sectional study in multiple cohorts in sub-Saharan Africa

Journal:	*BMJ Open*
Manuscript ID	bmjopen-2017-021438.R3
Article Type:	Protocol
Date Submitted by the Author:	31-Aug-2018

Complete List of Authors:	Pak, GiDeok; International Vaccine Institute, Biostatistics & Data Management<br>Haselbeck, Andrea; International Vaccine Institute, Epidemiology<br>Seo, Hyeong-Won; International Vaccine Institute, Biostatistics & Data Management<br>Osei, Isaac; Kumasi Centre for Collaborative Research in Tropical Medicine, Kwame Nkrumah University of Science and Technology (KNUST)<br>Amuasi, John; Kumasi Centre for Collaborative Research in Tropical Medicine, Kwame Nkrumah University of Science and Technology (KNUST)<br>Breiman, Robert; Emory University , Global Health Institute<br>Cruz Espinosa, Ligia Maria; International Vaccine Institute, Epidemiology<br>Holm, Marianne; International Vaccine Institute, Epidemiology<br>Im, Justin; International Vaccine Institute, Epidemiology<br>Jang, Geun-Hyeog; International Vaccine Institute, Biostatistics & Data Management<br>Jeon, Hyonjin; International Vaccine Institute, Epidemiology<br>Luby, Stephen; Stanford University Division of Infectious Diseases and Geographic Medicine<br>Lunguya-Metila, Octavie; Institut National de Recherche Biomedicales; Service de Microbiologie, Cliniques Universitaires de Kinshasa<br>MacWright, William; Global Health Institute, Emory University<br>Mogeni, Ondari; International Vaccine Institute, Epidemiology<br>Okeke, Iruka; University of Ibadan<br>Owusu-Dabo, Ellis; Kwame Nkrumah Univeristy of Science and Technology, School of Public Health<br>Park, Jin Kyung; International Vaccine Institute, Biostatistics & Data Management<br>Park, SeEun; International Vaccine Institute, Epidemiology<br>Popoola, Femi; University of Ibadan<br>Seo, Hye-jin; International Vaccine Institute, Epidemiology<br>Soura, Abdramane ; Institut Superieur des Sciences de la Population, University of Ouagadougou<br>Teferi, Mekonnen; Armauer Hansen Research Institute, ALERT Campus<br>Toy, Trevor; International Vaccine Institute, Epidemiology<br>Chon, Yun; International Vaccine Institute, Biostatistics & Data Management

	Rafindrakalia, Mathilde; Universite d'Antananarivo<br>Rakotozandrindrainy, Raphael; Universite d'Antananarivo<br>Meyer, Christian; Institute of Tropical Medicine, Eberhard Karls University; Faculty of Medicine, Duy Tan University<br>Marks, Florian ; International Vaccine Institute, Epidemiology; The Department of Medicine, The University of Cambridge<br>Panzner, Ursula; International Vaccine Institute, Epidemiology
**Primary Subject Heading**:	Epidemiology
Secondary Subject Heading:	Epidemiology, Global health, Health services research, Infectious diseases, Public health
Keywords:	health/hygiene behavior, sanitation, socio-economic, sub-Saharan Africa, HPAfrica study, population sampling frame/spatial sampling

SCHOLARONE™
Manuscripts

The HPAfrica Study Protocol: Assessment of health behavior and population-based socio-economic, hygiene behavioral factors - a standardized repeated cross-sectional study in multiple cohorts in sub-Saharan Africa

Short Title

The **H**ealth **P**opulation **Africa** (HPAfrica) study

Authors

Gi-Deok Pak[1#], Andrea Haselbeck[1#], Hyeong-Won Seo[1], Isaac Osei[2], John Amuasi[2], Robert F. Breiman[3], Ligia Maria Cruz Espinoza[1], Marianne Holm[1], Justin Im[1], Geun-Hyeog Jang[1], Hyon-Jin Jeon[1], Stephen Luby[4], Octavie Lunguya-Metila[5,6], William MacWright[3], Ondari D. Mogeni[1], Iruka N. Okeke[7], Ellis Owusu-Dabo[8], Jin-Kyung Park[1], Se Eun Park[1], Oluwafemi Popoola[7], Hye-Jin Seo[1], Abdramane Bassiahi Soura[9], Mekonnen Teferi[10], Trevor Toy[1], Yun Chon[1], Mathilde Rafindrakalia[11], Raphael Rakotozandrindrainy[11], Christian G. Meyer[12,13], Florian Marks[1,14*] and Ursula Panzner[1]

Affiliations

1. International Vaccine Institute, Seoul, Republic of Korea
2. Kumasi Centre for Collaborative Research in Tropical Medicine, Kwame Nkrumah University of Science and Technology (KNUST), Kumasi, Ghana
3. Global Health Institute, Emory University, Atlanta, United States
4. Infectious Diseases and Geographic Medicine, Stanford University, Stanford, United States
5. Institut National de Recherche Biom ́edicales, Kinshasa, Democratic Republic of the Congo
6. Service de Microbiologie, Cliniques Universitaires de Kinshasa, Kinshasa, Democratic Republic of the Congo
7. University of Ibadan, Ibadan, Nigeria
8. School of Public Health, Kwame Nkrumah Univeristy of Science and Technology, Kumasi, Ghana
9. Institut Supérieur des Sciences de la Population, University of Ouagadougou, Ouagadougou, Burkina Faso
10. Armauer Hansen Research Institute, Jimma Road, ALERT Campus, Addis Ababa, Ethiopia
11. University of Antananarivo, Antananarivo, Madagascar
12. Institute of Tropical Medicine, Eberhard Karls University, Tübingen, Germany
13. Faculty of Medicine, Duy Tan University, Da Nang, Vietnam
14. The Department of Medicine, The University of Cambridge, Cambridge, United Kingdom

# Contributed equally

*Corresponding Author:
Florian Marks, MPH, PhD, International Vaccine Institute, SNU Research Park, 1 Gwanak-ro, Gwanak-gu, Seoul, 08826, Republic of Korea, Phone: +82-2-881-1133, Fax: +82-2-881-1164, E-mail: fmarks@ivi.int

For peer review only - http://bmjopen.bmj.com/site/about/guidelines.xhtml

Abstract

Introduction

The objective of the **H**ealth **P**opulation **Africa** (HPAfrica) study is to determine health behavior and population-based factors, including socio-economic, ethnographic, hygiene and sanitation factors, at sites of the Severe Typhoid Fever in Africa (SETA) program. SETA aims to investigate healthcare facility-based fever surveillance in Burkina Faso, the Democratic Republic of the Congo, Ethiopia, Ghana, Madagascar, and Nigeria. Meaningful disease burden estimates require adjustment for health behavior patterns, which are assumed to vary among a study population.

Methods and Analysis

For the minimum sample size of household interviews required, the assumptions of an infinite population, a design effect, and age- and sex-stratification are considered. In the absence of a population sampling frame or household list, a spatial approach will be used to generate geographic random points with an Aeronautical Reconnaissance Coverage Geographic Information System (ArcGIS) tool. Printouts of Google Earth Pro® satellite imagery visualize these points. Data of interest will be assessed in different seasons by applying population-weighted stratified sampling. An Android-based application and a web service will be developed for electronic data capturing and synchronization with the database server in real time. Sampling weights will be computed to adjust for possible differences in selection probabilities. Descriptive data analyses will be performed in order to assess baseline information of each study population and age- and sex-stratified health behavior. This will allow adjusting disease burden estimates. In addition, multivariate analyses will be applied to look into associations between health behavior, population-based factors, and the disease burden as determined in the SETA study.

Ethics and dissemination

Ethic approvals for this protocol were obtained by the Institutional Review Board of the International Vaccine Institute (IVI, No. 2016-0003) and by all collaborating institutions of participating countries.

For peer review only - http://bmjopen.bmj.com/site/about/guidelines.xhtml

It is anticipated to disseminate findings from this study through publication on a peer-reviewed journal.

For peer review only

For peer review only - http://bmjopen.bmj.com/site/about/guidelines.xhtml

Strengths and limitations of this study

- Standardization in community-based multi-country/-site research requires cautiousness considering variations in setting, language and culture.
- Large-scale public health assessments will complement disease burden data by investigating sources of transmission and infection.
- Current population data in study sites and their administratively-defined subareas and population sampling frames are assumed to be limited.
- Electronic data collection may cause technical problems related to damage, functional failure, or loss of devices.
- Unstable wireless internet connection may limit data teams to update the Android-based application, communicate with study managers in real time, and synchronize collected data with the server.

Registration: Not required

Keywords: health/hygiene behavior, sanitation, socio-economic, population sampling frame/spatial sampling, sub-Saharan Africa, HPAfrica study

For peer review only - http://bmjopen.bmj.com/site/about/guidelines.xhtml

Introduction

Assessment of health behavior, including healthcare utilization, is an important determinant for the generation of accurate disease burden estimates among target populations. This is particularly critical for studies which utilize observational, healthcare facility-based surveillance designs as substantial variation in health behavior patterns strongly influences disease burden estimates.[1] The adjustment of disease burden calculations for variation in health behavior will increase the accuracy of measures of disease occurrence caused by bacterial, fungal, viral, and parasitic pathogens among populations under investigation. Furthermore, the influence of a large variety of population-based factors on both health behavior and disease burden has been recognized. This includes socioeconomic factors such as education, occupation, income, the availability of household assets,[2, 3] ethnical/religious and cultural factors,[4, 5] but also hygiene and sanitation facilities,[6-10] food handling,[11-14] and animal contacts.[9]

The **H**ealth **P**opulation **Africa** (HPAfrica) study will be conducted in Burkina Faso, the Democratic Republic of the Congo (DRC), Ethiopia, Ghana, Madagascar, and Nigeria. Experiences from the Typhoid Fever Surveillance in Africa Program (TSAP) will be transferred to the Severe Typhoid in Africa (SETA) program.[15, 16] In this program, standardized, healthcare facility-based fever surveillance is performed at selected study sites in participating countries over a two-year period.[17] SETA primarily aims to systematically collect information on the burden of severe *Salmonella* infections. Subjects living in defined catchment areas with either an acute fever episode, a fever history of ≥3 consecutive days, clinically-suspected typhoid fever or clinically-diagnosed gastrointestinal perforations due to typhoid fever will be eligible. In addition, the severity of *Salmonella* caused illnesses and the natural history of *Salmonella* infection will be recorded with further analyses on host immune responses and chronic carriage.

Objectives and Outcomes

The goal of the HPAfrica study is to assess the *generic* and *actual* age- and sex-stratified health

For peer review only - http://bmjopen.bmj.com/site/about/guidelines.xhtml

behavior. *Generic* health behavior pertains to behavior in conditions associated with fever and other signs and symptoms unrelated to onsets, whereas *actual* health behavior is related to onsets (Appendix 1/Supplementary file 1; Appendix 2/Supplementary file 2: Form 4 Part A, Form 5; Appendix 3/Supplementary file 3). These data will be used to adjust age- and sex-stratified measures of disease occurrence like incidences of infectious pathogens in the proportion of a study population not captured by the SETA surveillance program. Data documented for various signs and symptoms or a combination of these will be used for a gradual classification of healthcare utilization based on disease severity.

The HPAfrica study will, moreover, collect data on healthcare-associated factors such as travel modalities, possession of a health insurance, cultural/ethnographic factors, immunization status among children,[18, 19] and the frequency of occurrence and individual perception of selected diseases (Appendix 2/Supplementary file 2: Form 4 Part B; Appendix 3/Supplementary file 3:Form 4 Part B). Demographic data (Appendix 2/ Supplementary file 2: Form 3 Part A; Appendix 3/Supplementary file 3, Form 3 Part A) will be used for age- and sex-stratification of the population surveyed, which is required to compute adjustment factors for measures of disease occurrence. Socio-economic data (Appendix 2/ Supplementary file 2: Form 3 Part B; Appendix 3/ Supplementary file 3: Form 3 Part B) will be utilized to calculate wealth indices for descriptive and analytical approaches. Information on hygiene and sanitation facilities (Appendix 2/ Supplementary file 2: Form 3 Part C; Appendix 3/Supplementary file 3: Form 3 Part C) may provide a better understanding of the occurrence and frequency of pathogens identified among study populations.

Methods

Study sites

The boundaries of each site will be defined using pre-existing information set by statistical authorities or ministries of health (Table 1) coupled with open-source high-resolution geospatial data. A retrospective review of records of SETA recruitment healthcare facilities from the past two years will

For peer review only - http://bmjopen.bmj.com/site/about/guidelines.xhtml

support the re-defining of site limits. It is expected that this review will reveal the residences or at least the broader administrative residential areas such as communities, districts or villages of patients who sought healthcare for any reason. Maps and satellite imagery may be used to better visualize patients' residences and the overall boundaries, including the geographically- or administratively-defined subareas, census enumeration areas or strata, of each site. Where boundaries cannot be clearly defined, additional factors such as the distance to a recruitment healthcare facility may be considered.

For peer review only

For peer review only - http://bmjopen.bmj.com/site/about/guidelines.xhtml

Table 1: Background data of participating study sites of target countries

Country	Study site	Setting[*]	Approximate total site population (year)	Approximate age-stratified site population				Approximate sex-stratified site population	
				<2y	≥2y to <5y	≥5y to <15y	≥15y	Male	Female
Burkina Faso	Nioko II[1]	Urban	19,251 (2017)	2,394	1,977	4,657	10,223	9,321	9,930
	Polesgo[1]	Rural	7,897 (2017)	934	893	1,808	4,262	3,856	4,041
	Ouagadougou[1]	Urban	2,532,311 (2015)	421,429		693,723	1,417,159	1,271,302	1,261,009
DRC	Kisantu[2]	Urban	291,252 (2017)	48,043		243,209		91,598[#]	99,231[#]
Ethiopia	Wolayita Zone[3]	Urban/ Rural	1,968,735 (2017)	100,800	206,520	635,015	1,026300	964,577	1,004,058
	Wolayita Sodo[3]	Urban/ Rural	120,288 (2017)	6,161	12,617	38,807	62,703	59,898	60,390
	East Shewa & Arsi Zone[3]	Urban/ Rural	3,249,722 (2017)	104,408	422,939	1,016,917	1,705,458	1,671,699	1,578,023
	Adama Wenji[3]	Urban/ Rural	53,540 (2017)	3,330	7,204	17,028	25,978	27,199	26,341
Ghana	Asante Akim North & Central[4]	Urban/ Rural	140,694 (2010)	11,606	8,363	35,618	85,107	67,673	73,021
	Kumasi (Metropolis)[4,5]	Urban	1,730,249 (2010)	52,516	178,575	421,834	1,077,324	826,479	903,770
Madagascar	Antananarivo[6] (Renivohitra)	Urban	1,247,025 (2009)	N/A[$]	N/A[$]	N/A[$]	N/A[$]	N/A[$]	N/A[$]
	Imerintsiatosika[7]	Rural	44,669 (2016)	3,582	4,449	7,610	29,028	N/A[$]	N/A[$]
Nigeria	Ibadan[8]	Urban	1,343,147 (2006)	176,110		305,656	861,381	661,818	681,329

Note. Table 1 shows population data that were available at the time of the HPAfrica protocol writing; population data and boundaries of geographically and/or administratively defined study sites may be subject to changes during the course of the study. [*]The classification of sites by country is based on best local knowledge; [#]The sex-stratification is based on a total population of 190,829; [$]Population data not available at the time of protocol writing; y= years of age.

Source of population data: **Burkina Faso** ([1]http://www.insd.bf/n/; [2]http://www.indepth-network.org/member-centres/ouagadougou-hdss); **DRC** ([2]Kisantu Central Health Zone Office report, 2016); **Ethiopia** ([3]Health Management Information System of the Ethiopian Ministry of Health (zonal and district health offices); **Ghana** ([4]Ghana Statistical Service, 2010 Population & Housing Census, Asante Akim Central Municipality; [5]Ghana Statistical Service, 2010 Population & Housing Census, Summary report of final results); **Madagascar** ([6]Population par Fokotany selon la declaration des Chefs Fokotany: Source: Donnee de la cartographie censitaire mises-a-jour en juillet 2009 - INSTAT/DDSS; [7]University of Antananarivo); **Nigeria** ([8]Federal Republic of Nigeria 2006 Population and Housing Census (Table DS5), National Population Commission, Abuja, Nigeria).

For peer review only - http://bmjopen.bmj.com/site/about/guidelines.xhtml

Sample size

All available sources will be used to gather most up-to-date age- and sex-stratified population data by study site and its administratively- or geographically-defined subareas or strata. Strata will be defined as the smallest administrative unit as published by the census of a participating country. Households within strata are expected to be approximately homogeneously distributed. Population data sources for counts per stratum may include latest demographic information from census summary data or from a Demographic Surveillance System (DSS)/Health and Demographic Surveillance System (HDSS). Population summary figures and population growth factors, if available, may be an additional data source. For sites with outdated or unavailable population counts, open-access sampling tools coupled with population data sources like density-based gridded population data estimates from WorldPop may also be used.[20-25]

All strata per study site will be included. A household as defined for the HPAfrica study constitutes the primary sampling unit (PSU). The minimum number of randomly selected households to be interviewed will be calculated using the precision-based equation (Equation-I) assuming an infinite population and considering a design effect (DEFF),[26-28] including its accompanying assumptions:

Equation-I: Precision-based sample size calculation by study site for an infinite population [26-32]

$$n_0 = DEFF \cdot \left[{z^2}_{1-\frac{\alpha}{2}} \cdot \frac{4 \cdot p \cdot (1-p)}{d^2} \right]$$

Assumptions Equation-I

n_0	Minimum total number of households to be interviewed in a study area assuming an infinite population
$DEFF$	Design effect; set at 1.5<br>≤1.0=negative correlation of the outcome(s) of interest between household members; 1.0=no correlation of the outcome(s) of interest between household members; ≥1.0=positive correlation of the outcome(s) of interest between household members
$z_{1-\frac{\alpha}{2}}$	Normal deviation corresponding to a 95% confidence interval (1.96 for alpha of 0.05)
d	Precision (acceptable error); point estimation; set at 0.2
p	Proportion of the study population expected to visit a recruitment healthcare facility for conditions associated with fever and other signs and symptoms (proportion captured); set at 0.2
$1-p$	Proportion of the study population expected to not visit a recruitment healthcare facility for conditions associated with fever and other signs and symptoms (proportion not captured)

The design effect is defined as an adjustment factor for the natural clustering of health behavior as the

For peer review only

For peer review only - http://bmjopen.bmj.com/site/about/guidelines.xhtml

main outcome of interest among household members. It accounts for greater statistical variance and, therefore, lower precision compared to simple random sampling.[15, 30, 33, 34] For HPAfrica, the DEFF may be set conservatively at 1.5 based on an estimate of 1.42 resulting from an average household size of seven and an Intracluster Correlation Coefficient (ICC) of 0.07 from the TSAP study.[15, 31, 32, 35] A proportion p of 0.2 may be assumed if no other estimates are available, or p may be based on more precise information available by site or on experiences taken from the TSAP study (Table 2): Madagascar, Isotry: p =0.01, Burkina Faso, Polesgo: p =0.9.[36] Combining all assumptions into Equation-I will result in a total minimum number (n_0) of 92 household interviews per site.

In addition, n_0 will be accounted for the age- and sex-distribution of a study population reflected by q, the assumed minimum proportion of stratification per age group (<5 years, ≥5 to <15 years, ≥15 years). Factor q may be arbitrarily set at 0.2 or may be based on previous experiences: Ethiopia, Butajira: q=0.09, Burkina Faso, Polesgo: q=0.23.[15] Applying q to Equation-II for a binomial distribution, including its accompanying assumptions, will result in the age-stratified minimum number of household interviews ($\overline{n_0}$) of 461 (Equation-II):

For peer review only

Equation-II: Precision-based sample size calculation by study site for an infinite population considering age-stratification and population-weight by subarea or stratum:

$$\overline{n_0} = \left(DEFF \cdot \left[z^2{}_{1-\frac{\alpha}{2}} \cdot \frac{4 \cdot p \cdot (1-p)}{d^2} \right] \right) / q \qquad \overline{n_{0w}} = \overline{n_0} \cdot (n/N)$$

Assumptions Equation-II

$\overline{n_0}$	Minimum total number of households to be interviewed in the study area assuming an infinite population and age-stratification
$\overline{n_{0w}}$	Minimum number of households to be interviewed by subarea or stratum
n/N	Population size of subarea or stratum divided by the population size of the total study area
$DEFF$	Design effect; set at 1.5
$z_{1-\frac{\alpha}{2}}$	Normal deviation corresponding to a 95% confidence interval (1.96 for alpha of 0.05)
d	Precision (acceptable error); point estimation; set at 0.2
p	Proportion of the study population expected to visit a recruitment healthcare facility for conditions associated with fever and other signs and symptoms (proportion captured); set at 0.2
$1-p$	Proportion of the study population expected to not visit a recruitment healthcare facility for conditions associated with fever and other signs and symptoms (proportion not captured)
q	The minimum proportion of stratification per age group; set at 0.2

For peer review only - http://bmjopen.bmj.com/site/about/guidelines.xhtml

Health behavior will be assessed twice at the same households if possible or at alternative households in case the study population is instable due to migration and/or high birth/death rates. Additional households, assumed to be 20% on average, (Table 2) may be added to account for possible loss to follow-up between the two assessments. Eventually, population-weighted stratified sampling by the strata population proportion (n/N) to account for unequal selection probability due to heterogenous population distributions between strata (population-weight (n/N) by stratum) will be applied to $\overline{n_0}$ of 553.[37, 38]

Table 2: Sample sizes considering infinite population, differing estimates for p, age-stratification and loss to follow-up by study site applying Equation-II:

Proportion (p)	Total minimum number of households without DEFF	Total minimum number of households (n_0) with DEFF=1.5	Total minimum number of households ($\overline{n_0}$) with DEFF=1.5 and q=0.2	$\overline{n_0}$ adjusted for loss to follow-up
0.1	35	52	259	311
0.2	62	92	461	553
0.3	81	121	605	726
0.4	92	138	691	830
0.5	96	144	720	864
0.6	92	138	691	830
0.7	81	121	605	726
0.8	62	92	461	553
0.9	35	52	259	311

Abbreviations
p=Proportion of the study population expected to visit a recruitment healthcare facility for conditions associated with fever and other signs and symptoms (proportion captured); set at 0.2
DEFF=Design effect; set at 1.5
n_0=Total minimum number of households to be interviewed in a study area assuming an infinite population
$\overline{n_0}$=Total minimum number of households to be interviewed in the study area assuming an infinite population and age-stratification
q=Minimum proportion of stratification per age group; set at 0.2

Sampling with and without population sampling frame

If a comprehensive up-to-date population sampling frame or household list exists through DSS/HDSS or census [39, 40], computerized selection of households as PSUs will be performed using SAS (Statistical Analysis System, version 9.4, SAS Institute, Cary, NC) applying serial simple random sampling without replacement weighted by strata population proportion (n/N). Selected households labeled with identifiers are visualized using DSS/HDSS or census tools. In case of refusal, absence of

For peer review only - http://bmjopen.bmj.com/site/about/guidelines.xhtml

For peer review only

a respondent or locating of an abandoned household, interviewers are trained to visit an alternative household closest to the right or left side of an original household. This follows the principle of closest proximity as seen in previous research.[41-44] To limit, furthermore, chances of unequal selection probability, thus chances of selection bias by interviewers, we pre-choose an arbitrary number of five alternative households from the population sampling frame that require to be in closest proximity to the original household rather than applying for instance a lottery-based or clockwise spiral outward selection.[37, 41, 44] In addition, scoring from one (=closest) to five (=furthest) is applied to alternative households and interviewers are instructed to strictly follow the scoring sequence during the household selection procedure. Compliance of interviewers with the rules given for selecting original and alternative households will be verified on a daily basis. Deviations will be investigated and re-training provided to interviewers as required.

If no population sampling frame exists, we will apply a stratified spatial sampling technique weighted by the strata population proportion (n/N).[37] Guided by previous research conducted, random spatial points are generated using the ArcGIS (Aeronautical Reconnaissance Coverage Geographic Information System; Redlands, CA, USA, version 10.2) random point generation tool.[21, 23, 37, 40, 43, 45-51] The tool randomly places a number of points inside the feature of a polygon that corresponds to an administrative subarea or stratum. X- and Y-coordinates define one point until the calculated sample size by stratum is reached. ArcGIS-generated points are converted into an image file of the Keyhole Markup Language (KML) or Keyhole Markup language Zipped (KMZ) format and imported into Google Earth Pro®. A unique numerical identifier and geographic coordinates are assigned to each point. Appendix 4/Supplementary file 4 illustrates our approach utilizing examples from Ghana and Madagascar. Spatial points labeled with identifiers are pictured on poster-sized printouts of (60x60 to 60x90 cm) of Google Earth Pro® satellite imagery with high resolution or alternatively using an open-source application for offline/online automated navigation and mapping to locate points on the ground. Global Positioning System (GPS) receivers (Garmin-eTrex; Garmin Ltd., Lenexa, KS, USA) allow verifying the locations. The receivers will be positioned closely to a located

For peer review only - http://bmjopen.bmj.com/site/about/guidelines.xhtml

point, in a static position and an open area to assure barrier-free reading. Interviewers assess spatial points for the presence of a household as the PSUs.[24] In case of refusal, absence of a respondent or locating of a non-residential point, interviewers are trained to visit a pre-selected alternative spatial point closest to the right or left side of an original point and assess it for the presence of a household.[41-43, 52] To limit chances of unequal selection probability, we pre-select at least five alternative spatial points in closest proximity to the original point that represent a structure of appropriate size and rectangular or square shape from the Google Earth Pro® satellite imagery using the in-built distance measurement tool. Same as for sites with a population sampling frame, scoring from one to five is applied to alternative spatial points and interviewers are instructed to follow the scoring sequence. Compliance of interviewers with the rules given for selecting original and alternative households will be verified on a daily basis. Deviations will be investigated and re-training provided to interviewers as required. In the event that a spatial point is placed in equidistance to two (or more) structures, the interviewer skips the original spatial point and chooses a pre-selected alternative spatial point following the scoring sequence. In case two (or more) original spatial points are placed on the same structure, the interviewer assesses the structure for the presence of a household and chooses a second (or more) pre-selected alternative spatial point following the scoring sequence.

Irrespective of the presence or absence of a population sampling frame, a single-family, single-story household will be approached directly for study participation. However, one household only is enrolled at a multi-family, single-story structure based on following procedures: the interviewer enters the structure and assesses the total number of households; the first household on the right/left side (depending on the inner construction) of the structure is approached; if the first household does not participate, the second household on the right/left side of the same structure is approached. The interviewer continues until one household by structure is enrolled. Similarly, one household only is enrolled at a multi-family, multi-story structure. After assessing the total number of households present, the interviewer approaches the first household nearest to the entrance; if the first household does not participate, the second household closest to the entrance on the same/subsequent floor

For peer review only - http://bmjopen.bmj.com/site/about/guidelines.xhtml

(depending on the inner construction) is approached; the interviewer continues until one household by structure is enrolled. The interviewer chooses a pre-selected alternative spatial point following the scoring sequence in case no household can be enrolled.

Frequency of data collection

Residential points will be visited twice for household interviews during the SETA program to assess seasonal influences on health behavior.[53-57] Interviews will take place in differing seasons – one at the end of the dry season or at least one month after its beginning and one towards the end of the wet season or at least one month after its beginning (Table 3). Currently, the HPAfrica study is ongoing in Burkina Faso, Ghana and Madagascar and is anticipated to be continued in the remaining countries during the upcoming months.

Table 3: Seasonality in participating countries

Country	Period of Wet Season	Period of Dry Season
Burkina Faso	May/June - September/October	October/November - April/May
DRC	November – March	April - October
Ethiopia	June – August	October - May
Ghana	April – July, September – November	December - March
Madagascar	November - March/April	April/May - October
Nigeria	May – August	October - April

Note. The seasonality by country is based on best local knowledge.

Inclusion and exclusion criteria

Household members of all ages and both sexes living in a study area at the day of the interview will be eligible for inclusion. A household will be excluded if the designated respondent declines participation or is unavailable after three consecutive visits. All visitors and individuals with unknown residence or residence outside the study area will be excluded.

For the purpose of this investigation, a household is defined as a person or a group of related or unrelated persons living in the same dwelling unit, acknowledging one adult individual as the household head, sharing the same housekeeping arrangements, and independently procuring food and

For peer review only - http://bmjopen.bmj.com/site/about/guidelines.xhtml

other essential for living.[36]

The interview will exclusively be held with the respondent, who is an adult household member at the country-specific legal age of majority. This person may be identified as decision-maker by members of the same household and serves as a proxy for an entire household.[15] Further relevant definitions are explained in Appendix 1/Supplementary file 1 and Appendix 3/Supplementary file 3.

Data collection

On-site interviewers will be trained on locating the geographic points, identification of respondents, informed consent procedures with emphasis on voluntary participation, and the deployment of standardized, pre-tested study forms (Appendix 2/Supplementary file 2 and Appendix 3/Supplementary file 3) prior to the initiation of HPAfrica. [58] Informed consent and study forms will be forward-translated into the country-specific official language by two independent bilingual translators fluent in English and native speakers of the target language who are familiar with the concept and terminology of the forms. Both translations will be compared for discrepancies together with the translators and a coordinator, and consensus will be sought. The synthesized forward translation will be back-translated from the target language into English by two independent translators blinded to the original forms. Consensus on discrepancies of translations and the original forms will be sought together with the translators and a coordinator. Translated forms will be pilot-tested among a convenience sample of households of the target population prior to their finalization to assure cross-cultural comprehensibility and semantic, idiomatic, experiential and conceptual equivalence. A translation report will be prepared.[58-60]

Data management

Management of data will depend on the mode of collection, which will be primarily electronic rather than paper-based. Past experiences showed following disadvantages of paper-based data collection: a high probability of errors while filling study forms, and the need of subsequent data digitalization,

For peer review only - http://bmjopen.bmj.com/site/about/guidelines.xhtml

which is an extra, labor- and time-intensive and error-prone step. Electronic data capturing using an application for a mobile device like smartphone or tablet minimizes these limiting factors to the data quality.

There may be a pilot period during which both paper-based and paperless data will be collected prior to full implementation of electronic data collection. The latter utilizes the Android application "HPA Collect" (Google Android 5.0.1 9API 23; available at https://play.google.com/store/apps/details?id=anint.ivi.hpa) and the platform "HPA Web" (CSS, JavaScript and JSP; available at http://hpa.ivi.int/), which are accompanied by a variable dictionary containing codes, meaning and properties of variables in at least English and French. Both, "HPA Collect" and "HPA Web", will be designed specifically for the purpose of this study. IVI's expertise in computer engineering allows the fundamental construction, configuration and development of components required. Different from many open-source programs, a server installation feature enables the storage of all original data collected on the institutional server besides extra features for deep-error checking, logical relations between variables and forms, search functions, and analyzing and displaying of data collected. Stable wireless internet connectivity using default browsers is required to assure a contemporaneous, attributable, original and accurate synchronization of data between "HPA Collect" and "HPA Web". Access to both "HPA Collect" and "HPA Web" is password-protected. Appendix 5/Supplementary file 5 displays the system diagram, which has the following features:

Structure or features of "HPA Collect"

- **User management:** A simple user interface provides different privileges for users to enter, edit (except for study labels), and export or transfer data. It establishes an audit trail that records when users enter data into the server.
- **Data input:** Data are entered directly into the smartphone or tablet device. Entry fields limit possible answers to plausible ones only by offering different input methods:
  - Edit Text: free text

For peer review only - http://bmjopen.bmj.com/site/about/guidelines.xhtml

- Radio group/Multi click button: single choice
- Check box: multiple choice
- Date and time picker: entering date and time
- Spinner: drop-down button

Additional features allow the surveyor to save time by transferring data collected at an earlier stage to data fields required at a later stage of the interview by performing plausibility checks for diverse data input fields, and by easily recognizing skip patterns. Error checks, missing data notifications, code errors, and logical errors are also included.

- **Data view:** Raw, originally entered data can be seen in the same, human- and machine-readable format, namely the JSON format, as they will be transferred to a database server (Windows, MySQL). Additional functions like "clear" to clear all records, "reset" to return back to the main data view page, and "search" to search for a study label are available.
- **Export:** All entered data will be sent to the IVI server through wireless internet connection. Each form record having a unique study label of the household enrolled is transformed into a SQL insertion query and inserted onto the server only if there is no duplicated study label found. Insertion of data will be ignored in case of duplicated study labels.
- **Export to local:** All data entered will be backed-up threefold on a public storage folder of the mobile device as JSON and txt formats. Txt files contain data of each individual form. One version of JSON files contain data by each individual form and a second version of JSON files contain all data of a household enrolled. Data in the JSON format can be extracted by USB or wireless internet connection.
- **Report:** This feature allows a user to report any query to the leading "HPA Collect" administrator at IVI by e-mail or a third-party application.
- **Settings:** The ability to switch the system language between English and French is provided.

For peer review only - http://bmjopen.bmj.com/site/about/guidelines.xhtml

- **About:** The currently installed version of "HPA Collect" is displayed.

Structure or features of "HPA Web"

- **Home:** An introduction to the platform is given and a tutorial in French and English will be accessible.
- **Form View:** The study forms are displayed separately showing every question and the corresponding input data as defined variables.
- **Form Edit:** This feature allows the correction of the uploaded data. It is only accessible to data managers in each participating country and at IVI.
- **Search:** Specific study labels given to each study form can be filtered.
- **Variable dictionary:** The variable dictionary can be downloaded.
- **Export:** Data can be converted from the server into a transferrable excel file.
- **Contact Us:** Quick and easy correspondence between users and the data manager at IVI is given.

Data will be reviewed by key study personnel on a day-to-day basis and checked for consistency and accuracy prior to data analysis. The quality of data may be checked by selecting a subset of 5% for validation against the original, paper-based data if possible.[58] Names of household members will not be linked to study forms of HPAfrica and will not be recorded in the database in order to ensure confidentiality. Access to the database will be restricted to authorized study personnel only and data will be kept in a locked, protected location. Periodic interim backup files and at least three final backup files of the database stored in a secure, locked place will avoid loss of data and ensure data safety. All data will be stored for a minimum of five years.

Data analysis

Combined sampling weights will be computed to adjust for possible differences in selection probabilities and to increase the accuracy of study estimates.[22, 61] Weights are generated in a multi-

For peer review only - http://bmjopen.bmj.com/site/about/guidelines.xhtml

level approach to consider selection probabilities of e.g. households, inverse weights within households (i.e., multi-family single-story structure, multi-family multi-story structure) and inverse weights within household members participating in the HPAfrica study.[21, 43] Open-source tools, census data and HPAfrica-generated data such as the population size, the number of households or structures, the number of households within multi-family/multi-story structures and the household size by stratum will be used to compute sampling weights.[62, 63]

Descriptive analyses such as absolute and relative frequencies, medians or arithmetic means will be conducted to construct a contemporary baseline population description. Basic analyses will include the calculation of age- and sex-stratified proportions of *generic* and *actual* health behavior, including healthcare utilization, for various signs and symptoms or combinations of signs and symptoms. These analyses will allow for a gradual classification based on disease severity, including 95% confidence interval. Health behavior will be adjusted for actual clustering among household members. Most up-to-date stratified population data by site and demographic data derived from HPAfrica will be used to generate the proportions for the adjustment of incidence calculations.

Socio-economic information collected will be used to construct wealth indices of studied populations by applying factor analyses based on the principal component method. In addition, bi- and multivariate regression analyses will be performed to identify potential confounding factors or effect modification using factor scores.

Bi- and multivariate regression analyses will be carried out using factor scores for the assessment of the following variables:

- Socioeconomic data such as education, occupation,[64] health insurance, housing, and household assets
- Hygiene and sanitation such as toilet access, water sources, food handling, waste disposal, and animal contacts

For peer review only - http://bmjopen.bmj.com/site/about/guidelines.xhtml

- Health behavior: *actual* (age- and sex-stratified, severity, frequency), *generic* (age- and sex-stratified, severity, rating by choice)
- Travel modalities to health facilities, age-stratified
- Seasonality related to hygiene & sanitation, health behavior, travel modalities
- Cultural/religious/ethnographic factors, age-stratified
- Vaccination status among children [18, 19]
- Knowledge and perception of common diseases

Ethical considerations

All study participants are required to provide written informed consent. There are no direct benefits for enrolled households and their members. Indirect benefits for the general population at sites will be the updated information on the burden of communicable diseases, including factors influencing disease transmission and infection. The HPAfrica study generates evidence to support decision-makers on the introduction of appropriate measures for disease prevention and control.

This protocol was first approved by the Institutional Review Board of the International Vaccine Institute in October 2016 and undergoes annual review and renewal also by Ethics Committees of collaborating institutions (Ethics References are listed in Appendix1/Supplementary file1).

During and after the HPAfrica study, all data of enrolled subjects will be kept in strict confidence and will not be disclosed to a third party by any member of the research team. Password protection of devices and the database is used for strict confidence. All paper-based data (consent forms) will be kept in a secure, locked location. Confidential information stored on computers and paper-based data sources will only be made available to co-investigators and IVI staff directly involved in study activities of HPAfrica.

Patient and Public Involvement

For peer review only - http://bmjopen.bmj.com/site/about/guidelines.xhtml

No patients but household members in selected study sites will be involved. They did not participate in the development of the research question, outcome measures and the study design. However, we attempt to balance well between answering our study objectives and benefiting the general population at the sites by disseminating information on the disease burden and factors influencing disease transmission and infection derived from our study. Household members will contribute to the study conduct by direct participating in the gathering of data. They will not be involved in recruitment procedures. The households selected for participation will be identified purely by chance due to the random sampling applied. Study findings will be disseminated to study populations directly through collaborating institutions and indirectly through publications in peer-reviewed journals.

Dissemination

We plan to disseminate the study findings by reporting descriptive as well as analytic and stratified data through publications in peer-reviewed journals and collaborating institutions.

Authors' contributions

GDP, JKP and UP conceptualized the initial study concept of the HPAfrica study. HWS and GHJ developed the tools "HPA Collect" and "HPA Web" for electronic data collection with primary support of UP and AH. IO and JA supported the development and finalizing of the data collection tools "HPA Collect" and "HPA Web". MR and RR supported the development and finalization of the HPAfrica study forms. The appendices were conceptualized by GDP, JKP, YC, JI, TT, AH and UP. IO, JA, RB, LMCE, JI, HJJ, SL, OLM, WMW, ODM, INO, EOD, SP, OP, HJS, ABS, MT, TT, MR and RR supported the further development of the initial study protocol and appendices. FM, YC and CGM participated in the finalization of the study protocol, including appendices. AH wrote the first draft of the manuscript. GDP, HWS, IO, JA, RB, LMCE, MH, JI, GHJ, HJJ, SL, OLM, WMW, ODM, INO, EOD, JKP, SP, OP, HJS, ABS, MT, TT, YC, MR, RR, CGM and FM critically reviewed and revised the manuscript draft. GDP, AH, HWS, IO, JA, RB, LMCE, MH, JI, GHJ, HJJ, SL, OLM,

For peer review only - http://bmjopen.bmj.com/site/about/guidelines.xhtml

WMW, ODM, INO, EOD, JKP, SP, OP, HJS, ABS, MT, TT, YC, MR, RR, CGM, FM and UP agreed and approved the final manuscript as submitted.

Acknowledgement

We thank David Kauderer for the support and advice in computer engineering, Dr. Paul M. Tshiminyi for the support in proofreading French study materials, and Soo Young Kwon and Ji Hyun Han for administrative support. We are grateful to community leaders and site representatives for their assistance and facilitation in properly approaching household members for our study.

Funding statement

This study was supported by the Bill & Melinda Gates Foundation (OPP1127988). The funders had no role in study design, data collection, data analysis, data interpretation, or writing of the report; the conclusions and findings presented are those of the authors and do not necessarily reflect positions or policies of the Bill & Melinda Gates Foundation or the International Vaccine Institute (IVI). The IVI acknowledges its donors, including the Republic of Korea and the Swedish International Development Cooperation Agency (Sida).

Competing interests

The authors declare that they have no competing interests.

Abbreviations

JSON: JavaScript Objective Notation; CSS: Cascading Style Sheets; JSP: Java Server Page; SQL: Structured Query Language

Appendices

Appendix1 (Supplementary file 1)

Ethic References

For peer review only - http://bmjopen.bmj.com/site/about/guidelines.xhtml

Definitions/Terminology (English)

STROBE Statement—Checklist of items that should be included in reports of cross-sectional studies

Appendix 2 (Supplementary file 2)

Study Forms (English)

Form 1: Record of household visits

Form 2: Consent Form

Form 3: General household information

Part A: Demographic data/General household information

Part B: Socio-economic data

Part C: Hygiene and sanitation

Form 4: Assessment of age- and sex-stratified *generic* healthcare utilization

Part A: Assessment of *generic* healthcare utilization

Part B: Healthcare-associated factors/Vaccination/Disease perception

Form 5: Assessment of age- and sex-stratified *actual* healthcare utilization

Appendix 3 (Supplementary file 3)

Protocol, Definitions/Terminology and Study Forms (French)

Appendix 4 (Supplementary file 4)

Geospatial random selection (examples of Ghana and Madagascar)

Appendix 5 (Supplementary file 5)

System diagram of the data collection and data management: the Android application "HPA Collect" and the platform "HPA Web".

References

1. Bibigo, G., Audi, A., Aura, B., et al., Health-seeking patterns among participants of population-based morbidity surveillance in rural western Kenya: implications for calculating disease rates. *Int J Infect Dis*, 2010. 14(11): p. e967-73.

For peer review only - http://bmjopen.bmj.com/site/about/guidelines.xhtml

2. Jordan, H.T., Prapasiri, P., Areerat, P., et al., A comparison of population-based pneumonia surveillance and health-seeking behavior in two provinces in rural Thailand. *Int J Infect Dis*, 2009. 13(3): p. 355-61.

3. Saeed, B.I., Xicang, Z., Yawson, A.E., et al., Impact of socioeconomic status and medical conditions on health and healthcare utilization among aging Ghanaians. *BMC Public Health*, 2015. 15: p. 276.

4. Thoa, N.T., Thanh, N.X., Chuc, N.T., et al., The impact of economic growth on health care utilization: a longitudinal study in rural Vietnam. *Int J Equity Health*, 2013. 12: p. 19.

5. Okafor, I.P., Sekoni, A.O., Ezeiru, S.S., et al., Orthodox versus unorthodox care: A qualitative study on where rural women seek healthcare during pregnancy and childbirth in Southwest, Nigeria. *Malawi Med J*, 2014. 26(2): p. 45-9.

6. Farag, T.H., Kotloff, K.L., Levine, M.M., et al., Seeking care for pediatric diarrheal illness from traditional healers in Bamako, Mali. *Am J Trop Med Hyg*, 2013. 89(1 Suppl): p. 21-8.

7. Akullian, A., Ng'eno, E., Matheson, A.L., et al., Environmental Transmission of Typhoid Fever in an Urban Slum. *PLoS Negl Trop Dis*, 2015. 9(12): p. e0004212.

8. Steele, A.D., Hay Burgess, D.C., Diaz, Z., et al., Challenges and Opportunities for Typhoid Fever Control: A Call for Coordinated Action. *Clin Infect Dis*, 2016. 62 Suppl 1: p. S4-8.

9. Tran, H.H., Bjune, G., Nguyen, B.M., et al., Risk factors associated with typhoid fever in Son La province, northern Vietnam. *Trans R Soc Trop Med Hyg*, 2005. 99(11): p. 819-26.

10. Rweyemamu, M., Kambarage, D., Karimuribo, E., et al., Development of a One Health National Capacity in Africa : the Southern African Centre for Infectious Disease Surveillance (SACIDS) One Health Virtual Centre Model. *Curr Top Microbiol Immunol*, 2013. 366: p. 73-91.

11. Slayton, R.B., Date, K.A., Mintz, E.D., Vaccination for typhoid fever in sub-Saharan Africa. *Hum Vaccin Immunother*, 2013. 9(4): p. 903-6.

12. Sharma, P.K., Ramakrishnan, R., Hutin, Y., et al., Risk factors for typhoid in Darjeeling, West Bengal, India: evidence for practical action. *Trop Med Int Health*, 2009. 14(6): p. 696-702.

For peer review only - http://bmjopen.bmj.com/site/about/guidelines.xhtml

13. Eguale, T., Gebreyes, W.A., Asrat, D., et al., Non-typhoidal Salmonella serotypes, antimicrobial resistance and co-infection with parasites among patients with diarrhea and other gastrointestinal complaints in Addis Ababa, Ethiopia. *BMC Infect Dis*, 2015. 15: p. 497.

14. Paudyal, N., Anihouvi, V., Hounhouigan, J., et al., Prevalence of foodborne pathogens in food from selected African countries - A meta-analysis. *Int J Food Microbiol*, 2017. 249: p. 35-43.

15. Panzner, U., Pak, G.D., Aaby, P., et al., Utilization of Healthcare in the Typhoid Fever Surveillance in Africa Program. *Clin Infect Dis*, 2016. 62 Suppl 1: p. S56-68.

16. Von Kalckreuth, V., Konings, F., Aaby, P., et al., The Typhoid Fever Surveillance in Africa Program (TSAP): Clinical, Diagnostic, and Epidemiological Methodologies. *Clin Infect Dis*, 2016. 62 Suppl 1: p. S9-S16.

17. Park, S.E., Toy, T., Cruz Espinosa, L. M., et al., The Severe Typhoid in Africa Program: a multicentre population-based surveillance program with a nested case-control and cohort study (under submission).

18. WHO. WHO vaccine-preventable diseases: monitoring system. 2017 global summary 2017 [accessed 05 December 2017]; Available from: http://apps.who.int/immunization_monitoring/globalsummary/schedules/.

19. WHO, WHO recommendations for routine immunization - summary tables, 2017 [accessed 08 December 2017] ; Available from : http://www.who.int/immunization/policy/immunization_tables/en/.

20. GridSample. [accesed 05 June 2018]; Available from: www.gridsample.org.

21. Thomson, D.R., Hadley, M.B., Greenough, P.G., et al., Modelling strategic interventions in a population with a total fertility rate of 8.3: a cross-sectional study of Idjwi Island, DRC. *BMC Public Health*, 2012. 12: p. 959.

22. Thomson, D.R., Stevens, F.R., Ruktanonchai, N.W., et al., GridSample: an R package to generate household survey primary sampling units (PSUs) from gridded population data. *Int J Health Geogr*, 2017. 16(1): p. 25.

For peer review only - http://bmjopen.bmj.com/site/about/guidelines.xhtml

23. Galway, L.P., Bell, N., SAE, A.S., et al., A two-stage cluster sampling method using gridded population data, a GIS, and Google Earth(TM) imagery in a population-based mortality survey in Iraq. *Int J Health Geogr*, 2012. 11: p. 12.

24. Muñoz, J., Langeraar, W., A census-independent sampling strategy for a household survey in Myanmar. [04 June 2018]; Available from: http://winegis.com/images/census-independent-GIS-based-sampling-strategy-for-household-surveys-plan-of-action%20removed.pdf.

25. WorldPop. How would you like to access our data? [accessed 20 August 2018; Available from: www.worldpop.org.uk/data/get_data/.

26. Fleiss, J.L., Levin, B., Paik, M.C., Statistical Methods for Rates and Proportions. 3rd ed2003, New York: John. Wiley & Sons.

27. Henderson, R.H., Dacis, H., Eddins, D.L., et al., Assessment of vaccination coverage, vaccination scar rates, and smallpox scarring in five areas of West Africa. *Bull World Health Organ*, 1973. 48(2): p. 183-94.

28. Henderson, R.H., Sundaresan, T., Cluster sampling to assess immunization coverage: a review of experience with a simplified sampling method. *Bull World Health Organ*, 1982. 60(2): p. 253-60.

29. Chakraborty, H., et al., A simulation based technique to estimate intracluster correlation for a binary variable. *Contemp Clin Trials*, 2009. 30(1): p. 71-80.

30. Segura-Correa, J.C., Dominquez-Diaz, D., Avalos-Ramirez, R., et al., Intraherd correlation coefficients and design effects for bovine viral diarrhoea, infectious bovine rhinotracheitis, leptospirosis and neosporosis in cow-calf system herds in North-eastern Mexico. *Prev Vet Med*, 2010. 96(3-4): p. 272-5.

31. Katz, J. and S.L. Zeger, Estimation of design effects in cluster surveys. *Ann Epidemiol*, 1994. 4(4): p. 295-301.

32. van Breukelen, G.J., Candel, M.J., Calculating sample sizes for cluster randomized trials: we can keep it simple and efficient! *J Clin Epidemiol*, 2012. 65(11): p. 1212-8.

33. Kaiser, R., Woodruff, B.A., Bilukha, O., et al., Using design effects from previous cluster surveys to guide sample size calculation in emergency settings. *Disasters*, 2006. 30(2): p. 199-211.

For peer review only

For peer review only - http://bmjopen.bmj.com/site/about/guidelines.xhtml

34. Katz, J., Carey, V.J., Zeger, S.L., et al., Estimation of design effects and diarrhea clustering within households and villages. *Am J Epidemiol*, 1993. 138(11): p. 994-1006.

35. Newcombe, R.G., Two-sided confidence intervals for the single proportion: comparison of seven methods. *Stat Med*, 1998. 17(8): p. 857-72.

36. Panzner, U., Ali, M., Baker, S., et al., Geospatial sampling frames for household-based studies: Lessons learned from a multi-country surveillance network in Senegal, South Africa and Sudan. 2018 (under submission).

37. Kumar, N., Spatial Sampling Design for a Demographic and Health Survey. *Population Research and Policy Review*, 2007. 26(5/6): p. 581-599.

38. O'Muircheartaigh, C., Eckman, S., Smith, S., Statistical design and estimation for the national social life, health, and aging project. *J Gerontol B Psychol Sci Soc Sci*, 2009. 64(1): 30.

39. Eckman, S., English, N., Creating Housing Unit Frames from Address Databases: Geocoding Precision and Net Coverage Rates. *Field Methods*, 2012. 24(4), 399 - 408.

40. Himelein, K., Eckman, S., Murray, S., Sampling Nomads: A New Technique for Remote, Hard-to-Reach, and Mobile Populations. *JOS*, 2014. 30(2), 191 - 213.

41. Siri, J.G., Lindblade, K.A., Rosen, D.H., et al., A census-weighted, spatially-stratified household sampling strategy for urban malaria epidemiology. *Malar J*, 2008. 7: p. 39.

42. Sollom, R., Richards, A.K., Parmar, P., et al., Health and human rights in Chin State, Western Burma: a population-based assessment using multistaged household cluster sampling. *PLoS Med*, 2011. 8(2): p. e1001007.

43. Shannon, H.S., Hutson, R., Kolbe, A., et al., Choosing a survey sample when data on the population are limited: a method using Global Positioning Systems and aerial and satellite photographs. *Emerg Themes Epidemiol*, 2012. 9(1): p. 5.

44. Grais, R.F., Rose, A.M., Guthmann, J.P., Don't spin the pen: two alternative methods for second-stage sampling in urban cluster surveys. *Emerg Themes Epidemiol*, 2007. 4: p. 8.

45. Lohr, S.L., Sampling: Design and Analysis. 2nd edition ed2010, Pacific Grove, CA: Duxbury Press.

For peer review only

46. Lister, A.J., Scott, C.T., Use of space-filling curves to select sample locations in natural resource monitoring studies. *Environ Monit Assess*, 2009. 149(1-4): p. 71-80.

47. Keating, J., Macintyre, K., Mbogo, C., et al., A geographic sampling strategy for studying relationships between human activity and malaria vectors in urban Africa. *Am J Trop Med Hyg*, 2003. 68(3): p. 357-65.

48. Keating, J., Macintyre, K., Mbogo, C.M., et al., Characterization of potential larval habitats for Anopheles mosquitoes in relation to urban land-use in Malindi, Kenya. *Int J Health Geogr*, 2004. 3(1): p. 9.

49. ArcGIS. [accessed 10 October 2017]; Available from: http://pro.arcgis.com/en/pro-app/tool-reference/data-management/create-random-points.htm.

50. Wong, W.C., Zhao, Y., Wong, N.S., et al., Prevalence and risk factors of chlamydia infection in Hong Kong: A population-based geospatial household survey and testing. *PLoS One*, 2017. 12(2): p. e0172561.

51. Himelein, K., Eckman, S., Murray, S., et al., Alternatives to full listing for second stage sampling: Methods and implications. *Statistical Journal of the IAOS*, 2017. 33(3), 701-718.

52. Kondo, M.C., Bream, K.D., Barg, F.K., et al., A random spatial sampling method in a rural developing nation. *BMC public health*, 2014. 14: p. 338.

53. Kasper, M.R., Blair, P.J., Touch, S., et al., Infectious etiologies of acute febrile illness among patients seeking health care in south-central Cambodia. *Am J Trop Med Hyg*, 2012. 86(2): p. 246-53.

54. Dewan, A.M., Corner, R., Hashizume, M., et al., Typhoid Fever and its association with environmental factors in the Dhaka Metropolitan Area of Bangladesh: a spatial and time-series approach. *PLoS Negl Trop Dis*, 2013. 7(1): p. e1998.

55. Naeem Khan, M., Shafee, M., Hussain, K., et al., Typhoid fever in paediatric patients in Quetta, Balochistan, Pakistan. *Pak J Med Sci*, 2013. 29(4): p. 929-32.

56. Crump, J.A., Youssef, F.G., Luby, S.P., et al., Estimating the incidence of typhoid fever and other febrile illnesses in developing countries. *Emerg Infect Dis*, 2003. 9(5): p. 539-44.

For peer review only - http://bmjopen.bmj.com/site/about/guidelines.xhtml

57. Osei-Tutu, B., Anto, F., Trends of reported foodborne diseases at the Ridge Hospital, Accra, Ghana: a retrospective review of routine data from 2009-2013. *BMC Infect Dis*, 2016. 16: p. 139.

58. Wolf, C., Joye, D., Smith, T.W., et al., The SAGE Handbook of Survey Methodology, 2016. Los Angeles, CA. Sage Publications, Ltd.

59. Koller, M., Aaronson, N.K., Blazeby, J., et al., Translation procedures for standardised quality of life questionnaires: The European Organisation for Research and Treatment of Cancer (EORTC) approach. *Eur J Cancer*, 2007. 43(12): p. 1810-20.

60. Acquadro, C., Conway, K., Hareendran, A., et al., Literature review of methods to translate health-related quality of life questionnaires for use in multinational clinical trials. *Value Health*, 2008. 11(3): p. 509-21.

61. Milligan, P., Njie, A., Bennett,S., Comparison of two cluster sampling methods for health surveys in developing countries. *Int J Epidemiol*, 2004. 33(3): p. 469-76.

62. Kamanga, A., Renn, S., Pollard, D., et al., Open-source satellite enumeration to map households: planning and targeting indoor residual spraying for malaria. *Malar J*, 2015. 14: p. 345.

63. Elsey, H., Thomson, D.R., Lin, R.Y., et al., Addressing Inequities in Urban Health: Do Decision-Makers Have the Data They Need? Report from the Urban Health Data Special Session at International Conference on Urban Health Dhaka 2015. *J Urban Health*, 2016. 93(3): p. 526-37.

64. ILO), I.L.O. International Standard Classification of Occupations ISCO-08, 2012. [accessed 02 December 2017; Available from: http://www.ilo.org/wcmsp5/groups/public/---dgreports/---dcomm/---publ/documents/publication/wcms_172572.pdf.

For peer review only - http://bmjopen.bmj.com/site/about/guidelines.xhtml

Appendix 1/Supplementary file 1:
Ethic References
Definitions/Terminology (English)
STROBE Statement—Checklist of items that should be included in reports of *cross-sectional studies*

Ethics References

- IVI Institutional Review Board (IRB), No. 2016-003
- Institute of Tropical Medicine Antwerp Institutional Review Board, Belgium
- Universiteit Antwerpen, Comite voor medische Ethiek, Belgium
- Ministère de la Santé du Burkina Faso – Comité d'Ethique pour la Recherche en Santé, Burkina Faso
- Comité d'Ethique de l'Ecole de Santé Publique de l'Université de Kinshasa, Democratic Republic of Congo
- National Research Ethics Review committee (NRERC) Ministry of Science and Technology, Federal Democratic Republic of Ethiopia
- AHRI-ALERT Ethics Review Committee (AAERC), Ethiopia
- Kwame Nkrumah University of Science and Technology, School of Medical Sciences/Komfo Anokye Teaching Hospital, Committee on Human Research, Publication and Ethics, Ghana
- Ministère de la Santé du Repoblikan'l Madagaskar – Comité d'Ethique
- University of Ibadan/University College Hospital Ethics Committee, Ibadan, Nigeria

Definitions/Terminology (English)

**A**	
Abdominal pain/ Intestinal pain	Pain (acute/short-lived or chronic/long-lived) that occurs anywhere between the chest and the groin (often referred to as the stomach region/belly).
Aches/Pain	A condition of having continuous/prolonged dull pain in a part of the body; the physical feeling, suffering, or discomfort caused by an illness or injury.
**B**	
Bleeding (internal/external)/ Blood loss	The condition of losing blood from the vascular system, either internally into the body (internal bleeding) or externally through a natural orifice or break in the skin (external bleeding); blood escaping from an injured blood vessel.
Blood loss/ Bleeding	The condition of losing blood from the vascular system, either internally into the body (internal bleeding) or externally through a natural orifice or break in the skin (external bleeding); blood escaping from an injured blood vessel.
Blood pressure disorders	A high/low blood pressure or a blood pressure that shows excessive fluctuation.
**C**	
Chills/Shivering	The sensation of coldness that could be accompanied by shivering or shaking of the body and pallor of the skin.
Cholera	An infectious, sometimes fatal disease of the small intestine caused by the bacterium *Vibrio cholerae*; it is spread via the fecal-oral route from the ingestion of contaminated water and food and causes chills, shivering, severe watery diarrhea, vomiting, and dehydration.
Confusion/Dizziness/ Unconsciousness	The state of being bewildered or unclear in mind; an abnormal state of response to stimuli in the environment; faint, wooziness, weakness, unsteadiness or a tendency to fall; the interruption of the awareness of oneself and one's surroundings.
Convulsion	The sudden, violent, irregular movement of a limb/part of the body or of the entire body which is caused by involuntary contraction of muscles.
Cough	The condition of expelling air from the lungs suddenly/rapidly and with a harsh noise; often involuntarily in order to clear the lung airways of fluids/mucus/other material.
**D**	

For peer review only - http://bmjopen.bmj.com/site/about/guidelines.xhtml

Dehydration	A condition of excessive loss of water/moisture from the body.
Diarrhea	A condition of passing feces from the body very frequently and in a liquid rather than solid form.
Diphtheria	A highly contagious, sometimes fatal disease caused by the bacterium *Corynebacterium diphtheriae*. It is spread from person-to-person, through the air or contaminated objects and affects the upper respiratory tract. Common symptoms are sore throat, swollen glands/ lymph nodes, malaise, fever/chills, a membranous substance/thick gray material that forms in the throat/tonsils and makes it difficult to breathe/ rapid breathing ("barking cough") and swallow. A form of diphtheria that involves the skin, eyes, or genitals is also reported and is characterized by pain, redness, and swelling of the skin.
Dizziness/Confusion/ Unconsciousness	The state of being bewildered or unclear in mind; an abnormal state of response to stimuli in the environment; faint, wooziness, weakness, unsteadiness or a tendency to fall; the interruption of the awareness of oneself and one's surroundings.
**E**	
Edema/Swelling	The excess collection of a watery fluid/swelling in the cavities or tissues of the body; it's commonly noticed in the hands/arms/feet/legs; volume increase of tissue.
Education - formal	A systematic, organized education model that is structured and administered according to a given set of laws and norms; formal education institutions are administratively, physically and curricularly organized and require a minimum classroom attendance from students; it involves intermediate and final assessments in order to advance to the next learning stage; it confers degrees and diplomas pursuant to a quite strict set of regulations.
**F**	
Fatigue/Malaise/ Weakness	The state of feeling tired, exhausted, or weak; a general feeling of discomfort, uneasiness, unhealthiness, or unhappiness.
Fever, any	An abnormally high body temperature (usually above 38°C).
Fever <3 days	An abnormally high body temperature (usually above 38°C) that appears continuously for less than 3 days.
Fever >3 days	An abnormally high body temperature (usually above 38°C) that lasts longer than 3 days and appears continuously.
**G**	
**H**	
Headache	Any kind of pain in any region of the head.
Healthcare - actual	Healthcare that is related to specific sign/symptom-associated disease onsets or disease onset dates.
Healthcare - general	Healthcare that is unrelated to specific sign/symptom-associated disease onsets or disease onset dates.
Healthcare facility	Places or institutions where healthcare is provided; including hospitals, clinics, healthcare centers, health posts, and specialized care centers.
Haemophilus influenzae type B	A bacterium that causes a range of illnesses and is spread by droplets through coughs and sneezing, and occurs primarily among children <5years of age; common signs are fever, headache, and stiff neck up to sepsis. It can cause ear infections, cellulitis (soft tissue infection), arthritis, upper respiratory infections, pneumonia, meningitis (potential brain damage) and epiglottitis (with airway obstructions).
Heart disorders	Any disorder that affects or is caused by the heart; conditions that involve narrowed/blocked blood vessels that can lead to heart attack, chest pain, or stroke; condition that may affect one's heart muscle, valves, rhythm, or blood pressure.
Hepatitis B	An infectious disease caused by the hepatitis B virus (HBV) that affects the liver (acute/chronic liver infection; may result in liver failure, cancer, or cirrhosis). The virus is spread from person-to-person through blood, semen, or other bodily fluids. Common symptoms are fever, malaise/weakness/fatigue, loss of appetite, nausea/ vomiting, abdominal discomfort/pain, dark-colored urine, joint pain and jaundice.
Hepatitis E	An infectious disease caused by the hepatitis E virus (HEV) that affects the liver (only acute liver infection; may result in acute liver failure). The virus is spread via the fecal-oral route through the ingestion of contaminated food and water, infected

For peer review only - http://bmjopen.bmj.com/site/about/guidelines.xhtml

	animals (zoonotically) and blood. Common symptoms are fever, malaise, loss of appetite, diarrhea, nausea, abdominal discomfort, dark-colored urine, joint pain and jaundice.
HIV/AIDS	An infectious disease caused by the human immunodeficiency virus (HIV) that affects/damages the immune system and results in the acquired immunodeficiency syndrome (AIDS), a chronic, potentially life-threatening condition; it is transmitted through infectious body fluids such as blood, semen, and other bodily fluids. Common symptoms of an acute/early infection are fever, fatigue, headache, aches/joint pain, rash, sore throat, swollen glands/lymph nodes, diarrhea, weight loss, oral yeast infection and shingles. The untreated disease progresses to AIDS; symptoms caused by the severely damaged immune system are night sweats, recurring fever, chronic diarrhea, lesions on tongue and in the mouth, fatigue, weight loss, and skin rushes.
Household	A person or a group of related or unrelated persons that live together in the same dwelling unit, that acknowledge one male or female adult as the head of the household, that share the same housekeeping arrangements, that are considered to constitute one unit, and that provide themselves with food or other essentials for living. A household may be located in a single housing unit (single-story building) or in a set of collective living quarters (multi-story building).
**I**	
Income/Wage	Money or another form of payment that someone receives periodically in exchange for providing a good or service. It is usually received from a job/occupation.
Influenza	An infectious, highly contagious disease of the respiratory tract that is caused by flu viruses; it is spread from person-to-person via infected saliva droplets and contaminated objects. Common symptoms are fever, aches in muscles/arms/legs, chills/sweats, headache, fatigue/weakness, runny nose/nasal congestion, sneezing, sore throat and dry persistent cough.
Intestinal pain/ Abdominal pain	Pain (acute/short-lived or chronic/long-lived) that occurs anywhere between the chest and the groin (often referred to as the stomach region/belly).
**J**	
Jaundice	A condition of yellowing of the skin/whites of the eyes.
**K/L**	
**M**	
Malaise/Fatigue/ Weakness	The state of feeling tired, exhausted, or weak; a general feeling of discomfort, uneasiness, unhealthiness, or unhappiness.
Malaria	An infectious, sometimes fatal disease caused by a parasite (*Plasmodium* spp.) that is transmitted primarily by the bite of infected mosquitoes; infection may also spread from mother-to-child prenatally or during delivery, or through contact with contaminated blood. Common symptoms are recurrent attacks of chills, sweating, high fever, headache, vomiting and diarrhea.
Measles	An infectious, sometimes fatal disease of the respiratory tract (nose/throat) that is caused by a virus that affects mainly children <5years of age; it is spread from person-to-person via infected saliva droplets and contaminated objects. Common symptoms are fever, dry cough, runny nose, inflamed eyes, sore throat, large flat spots on the skin, and white spots in the mouth and on the cheek.
Meningitis	A viral/bacterial/fungal, sometimes life-threatening infectious inflammation of the membranes (meninges) surrounding the brain and spinal cord; it is spread from person-to-person via infected saliva droplets and contaminated objects. Common symptoms are high fever, severe headache, nausea/vomiting, confusion, seizures, loss of appetite and thirst, fatigues, sensitivity to light, skin rush and stiff neck.
Mumps	A viral infectious disease caused by the mumps virus that affects mainly the parotid glands that are situated below and in front of the ears; it is spread from person-to-person via infected saliva droplets and contaminated objects. Common symptoms are fever, swelling of one/both parotid glands, headache, muscle aches, weakness/fatigue, loss of appetite and pain while shewing/swallowing; a known complication may be hearing loss.

For peer review only - http://bmjopen.bmj.com/site/about/guidelines.xhtml

**N**	
Nausea/Vomiting	A feeling of sickness in the stomach with an inclination to vomit; a condition of ejecting part or all of the contents of the stomach, primarily through the mouth.
Neighbor	A person (related or unrelated) living next door to a household; a neighbor does not live together with members of a household (next door) in the same dwelling unit, does not share the same housekeeping arrangements, food, or other living essentials with members of a household (next door), and does not constitute one unit with members of a household (next door).
**O**	
Occupation	A job, or means of earning a wage/living; often requires the mastery of a complex set of knowledge, tasks, duties and skills acquired through formal education and/or practical experience.
**P**	
Pain/Aches	A condition of having continuous/prolonged dull pain in a part of the body; the physical feeling, suffering, or discomfort caused by an illness or injury.
Pertussis	Pertussis, also known as whooping cough, is a highly contagious bacterial disease. Symptoms similar to a common cold like runny nose, fever and cough are followed by weeks of sever coughing and a high-pitched whooping sound or gasping when a diseased person breathes in; severe coughing my even be followed by vomiting, breaking of ribs, or extreme fatigue. Infected very young and old people may have little or no cough, or they may have periods of interrupted breathing during which they do not breath are all. Pertussis is transmitted by the bacterium *Bordetella pertussis* through coughs and sneezes of an infected person.
Pharmacy	A place where medicines (prescription or nonprescription) are prepared, preserved, compounded and dispensed.
Physician	A person skilled/trained in healing; a person educated, clinically experienced, and licensed to practice medicine.
Pneumococcus	A bacterial infection caused by the bacterium *Streptococcus pneumoniae*; a sometimes fatal inflammation of the lungs that often affects immunocompromised people; it is spread from person-to-person via infected saliva droplets and contaminated objects. Common symptoms are fever, chills, cough (with phlegm/pus), chest pain, difficulty in breathing/shortness of breath, fatigue, nausea/vomiting and diarrhea; rare symptoms are sepsis, pleural effusion, and empyema.
Polio	A viral, highly contagious, sometimes fatal infectious disease caused by the poliovirus; in its most severe form causes paralysis and difficulties in breathing; it is transmitted via the fecal-oral route through ingestion of contaminated water and food, and from person-to-person contact. Common symptoms are fever, sore throat, headache, vomiting, fatigue, back pain/neck pain/stiffness, muscle weakness/aches, meningitis, loss of reflexes, and loose/floppy limbs.
**Q**	
**R**	
Rapid breathing/ Shortness of breath	The condition of abnormally fast breathing; a rate of >20 breaths per minute for adults, >30 breaths per minute for children and >45 breaths per minute for babies.
Respondent	An adult (at the country-specific legal age of majority) household member who is a decision-maker for the entire household within the study site; thus, this adult person serves as a proxy for all household members. This person may be identified by other members of the same household as the person who is primarily involved in the daily healthcare of household members.
Rotavirus	A viral infectious disease caused by rotavirus that results predominantly in diarrhea among children <5years of age; it spreads through the fecal-oral route from person-to-person contact and contaminated objects. Common symptoms are watery diarrhea, fever, vomiting, abdominal pain and dehydration.
Rubella	A contagious, viral infectious disease caused by the rubella virus; known by its distinctive red rash; it is spread from person-to-person via infected saliva droplets), from mother-to-child prenatally via blood, and contaminated objects. Common

For peer review only - http://bmjopen.bmj.com/site/about/guidelines.xhtml

	symptoms are mild fever, headache, stuffy/runny nose, inflamed red eyes, enlarged tender lymph nodes at the base of the skull/back of the neck/behind the ears, joint pain and a fine pink rash that moves from the face to the trunk, arms and legs.
Runny nose	The condition when the nose (nasal and adjacent tissues and blood vessels) produces extra mucus/excess drainage.
**S**	
Self-treatment	The medication of oneself or treatment of one's own disease or condition without medical supervision or prescription.
Severe breathing/ Difficulties in breathing	The condition of abnormal breathing that may be accompanied by wheezing, chest pain, chest in-drawing, stridor, swelling in the chest, or cough for instance.
Shivering/Chills	The sensation of coldness that could be accompanied by shivering or shaking of the body and pallor of the skin.
Shortness of breath/ Rapid breathing	The condition of abnormally fast and deep breathing; a rate of >20 breaths per minute for adults, >30 breaths per minute for children and >45 breaths per minute for babies.
Sneezing	The condition of suddenly forcing air out through nose and mouth with a usual loud noise.
Swelling/Edema	A condition of an excess collection of a watery fluid/swelling in the cavities or tissues of the body; it's commonly noticed in the hands/arms/feet/legs; volume increase of tissue.
**T**	
Tetanus	Tetanus is an infection characterized by muscle spasms which commonly begins in the jaw and progresses to the rest of the body. The spasms usually last for a few minutes only and occur frequently during 3-4 weeks of the illness but can be so severe that they may cause bone fractures or death when they affect the respiratory muscles. Tetanus may be characterized by further symptoms like fever, sweating, headache, difficulties in swallowing, high blood pressure, and an increased heart rate. The disease is caused by an infection with the bacterium *Clostridium tetani* that is found in soil, salvia, dust and manure. The bacterium enters through a skin injury (e.g. cut, puncture wound) by a contaminated object. It produces toxins that interfere with muscle contractions that result in the typical spasms.
Traditional healer	A person that aims to maintain health as well as to prevent, diagnose, and improve or treat illnesses by using a sum of knowledge, skills and practices based on theories, beliefs and experiences.
Tuberculosis	A contagious, potentially serious infectious disease caused by the bacteria *Mycobacterium tuberculosis*; primarily affects the lungs, but can also affect the kidneys and spine/brain; it is transmitted from person-to-person via infected saliva droplets. Common symptoms of active tuberculosis are cough (with or without blood), chest pain, weight loss, fatigue, fever, night sweats, chills and loss of appetite.
Typhoid fever	A bacterial, potentially life-threatening infectious disease caused by *Salmonella typhi*; it is transmitted via the fecal-oral route through contaminated food and water, or from person-to-person contact. Common symptoms are high fever, headache, abdominal pain, constipation/diarrhea, and rash. Serious complications (i.e. perforation of the gut) are reported.
**U**	
Unconsciousness/ Dizziness/Confusion	The state of being bewildered or unclear in mind; an abnormal state of response to stimuli in the environment; faint, wooziness, weakness, unsteadiness or a tendency to fall; the interruption of the awareness of oneself and one's surroundings.
**V**	
Varicella	A viral, highly contagious infectious disease, commonly known as chickenpox, that primarily affects children and is caused by the varicella-zoster virus; it is spread from person-to-person by contact with infected blisters or saliva droplets. Common symptoms are mild fever, headache, backache, loss of appetite, and vesicular skin eruptions/rash/red spots on the back and chest accompanied by severe itching.

For peer review only - http://bmjopen.bmj.com/site/about/guidelines.xhtml

Visitor	A person (related or unrelated) who comes to a household to spend some time with household members; a visitor does not live together with household members in the same dwelling unit, does not share the same housekeeping arrangements, food, or other living essentials with household members, and does not constitute one unit with household members.
Vomiting/Nausea	A feeling of sickness in the stomach with an inclination to vomit; a condition of ejecting part or all of the contents of the stomach, primarily through the mouth.
**W**	
Weakness/Malaise/ Fatigue	The state of feeling tired, exhausted, or weak; a general feeling of discomfort, uneasiness, unhealthiness, or unhappiness.
Weight loss	The (voluntary or involuntary) decrease in body weight.
Witness	A person who is an adult (at the country-specific legal age of majority), who is independent of the study, who cannot be unfairly influenced by study staff, who attends the informed consent process if the subject or subject's legally acceptable representative is illiterate, and who reads (literate) the informed consent form and any other written information supplied to the subject.
**X/Y/Z**	
Yellow fever	An acute infectious, human pathogenic, viral disease that is marked by the sudden onset of symptoms like fever, chills, loss of appetite, nausea, muscle pain, and headache, that typically resolve within a few days or are followed by more serious symptoms such as jaundice, high fever and hemorrhage. The disease is caused by a virus of the genus Flavivirus and is transmitted by the bite of an infected mosquito.

For peer review only

STROBE Statement—Checklist of items that should be included in reports of ***cross-sectional studies***

	Item No	Recommendation	
**Title and abstract**	1	(*a*) Indicate the study's design with a commonly used term in the title or the abstract	√
		(*b*) Provide in the abstract an informative and balanced summary of what was done and what was found	√
**Introduction**			
Background/rationale	2	Explain the scientific background and rationale for the investigation being reported	√
Objectives	3	State specific objectives, including any prespecified hypotheses	√
**Methods**			
Study design	4	Present key elements of study design early in the paper	√
Setting	5	Describe the setting, locations, and relevant dates, including periods of recruitment, exposure, follow-up, and data collection	√
Participants	6	(*a*) Give the eligibility criteria, and the sources and methods of selection of participants	√
Variables	7	Clearly define all outcomes, exposures, predictors, potential confounders, and effect modifiers. Give diagnostic criteria, if applicable	√
Data sources/ measurement	8*	For each variable of interest, give sources of data and details of methods of assessment (measurement). Describe comparability of assessment methods if there is more than one group	√
Bias	9	Describe any efforts to address potential sources of bias	√
Study size	10	Explain how the study size was arrived at	√
Quantitative variables	11	Explain how quantitative variables were handled in the analyses. If applicable, describe which groupings were chosen and why	√
Statistical methods	12	(*a*) Describe all statistical methods, including those used to control for confounding	√
		(*b*) Describe any methods used to examine subgroups and interactions	√
		(*c*) Explain how missing data were addressed	Not

For peer review only - http://bmjopen.bmj.com/site/about/guidelines.xhtml

			applicable
		(*d*) If applicable, describe analytical methods taking account of sampling strategy	Not applicable
		(*e*) Describe any sensitivity analyses	Not applicable
**Results**			
Participants	13*	(a) Report numbers of individuals at each stage of study—eg numbers potentially eligible, examined for eligibility, confirmed eligible, included in the study, completing follow-up, and analysed	Not applicable
		(b) Give reasons for non-participation at each stage	Not applicable
		(c) Consider use of a flow diagram	Not applicable
Descriptive data	14*	(a) Give characteristics of study participants (eg demographic, clinical, social) and information on exposures and potential confounders	Not applicable
		(b) Indicate number of participants with missing data for each variable of interest	Not applicable
Outcome data	15*	Report numbers of outcome events or summary measures	Not applicable
Main results	16	(*a*) Give unadjusted estimates and, if applicable, confounder-adjusted estimates and their precision (eg, 95% confidence interval). Make clear which confounders were adjusted for and why they were included	Not applicable
		(*b*) Report category boundaries when continuous variables were categorized	Not applicable
		(*c*) If relevant, consider translating estimates of relative risk into absolute risk for a meaningful time period	Not applicable
Other analyses	17	Report other analyses done—eg analyses of subgroups and interactions, and sensitivity analyses	Not applicable

For peer review only

**Discussion**			
Key results	18	Summarise key results with reference to study objectives	Not applicable
Limitations	19	Discuss limitations of the study, taking into account sources of potential bias or imprecision. Discuss both direction and magnitude of any potential bias	Not applicable
Interpretation	20	Give a cautious overall interpretation of results considering objectives, limitations, multiplicity of analyses, results from similar studies, and other relevant evidence	Not applicable
Generalisability	21	Discuss the generalisability (external validity) of the study results	Not applicable
**Other information**			
Funding	22	Give the source of funding and the role of the funders for the present study and, if applicable, for the original study on which the present article is based	√

For peer review only

For peer review only - http://bmjopen.bmj.com/site/about/guidelines.xhtml

Site ___ ___ ___ Subarea ___ ___ ___ House number ___ ___ ___ ___ Interviewer ID ___ ___

Appendix 2/Supplementary file 2: Study forms (English)

FORM 1 – RECORD OF HOUSEHOLD VISITS

Instructions to the interviewer

- Complete "Form 1 – RECORD OF HOUSEHOLD VISITS" for every household visited.
- Complete "Home/Respondent/Completed" once all survey parts have been completed; if not all data haven been collected at the initial and/or a consecutive visit (max. 3 visits), schedule a revisit.
- Attempt 1: complete the status of the household visit using given options; for "Not Home", "Home/Non-Respondent" and "Home/Respondent/Unavailable" schedule a revisit; for "Home/Respondent/Refusal" state the reason for refusal and visit a replacement household to the nearest on the right or left side of this household; for ~~and~~ "Non-existing/Non-residential" skip this household and visit a replacement household to the nearest on the right or left side of this household instead.
- Attempt 2: complete the status of the household visit using given options; for "Not Home", "Home/Non-Respondent" and "Home/Respondent/Unavailable" schedule a revisit.
- Attempt 3: complete the status of the household visit using given options; for "Not Home", "Home/Non-Respondent", "Home/Respondent/Unavailable" skip this household and visit a replacement household to the nearest on the right or left side of this household instead.
- "Site" and "Subarea" must be entered each as a 3-digit, "House number" as a 4-digit, and "Interviewer ID" as a 2-digit code (use lists individually prepared for each site to obtain codes); enter "Date" in the format DD/MM/YY and "Time" in the format HH:MM and circle AM or PM. "Study label" must be a combination of a 3-digit "Site" code, a 3-digit "Subarea" code and a 4-digit "House number" code; the "Interviewer ID" must be a entered as a 3-digit code.

**Attempt 1**	Date ____/____/____ (DD/MM/YY)	Time _____/_____ AM/PM	Interviewer ID ___ ___
STATUS		Tick one	
1=Not home		O	➡ Schedule revisit (Date/Time)
2=Home/Non-Respondent		O	➡ Schedule revisit (Date/Time)
3=Home/Respondent/Unavailable		O	➡ Schedule revisit (Date/Time)
4=Home/Respondent/Completed		O	
5=Home/Respondent/Refusal		O	Record the reason(s) for refusal; tick all that applies<br>O 1=No time to answer questions<br>O 2=Don't want to participate/answer questions<br>O 3=Other ______________________________<br>O 98=No response O 99=Don't know<br>➡ Visit a replacement household to the nearest right or left side of this household
6=Non-existing/Non-residential		O	➡ Visit a replacement household to the nearest right or left side of this household
**Attempt 2**	Date ____/____/____ (DD/MM/YY)	Time _____/_____ AM/PM	Interviewer ID ___ ___
STATUS		Tick one	
1=Not home		O	➡ Schedule revisit (Date/Time)
2=Home/Non-Respondent		O	➡ Schedule revisit (Date/Time)
3=Home/Respondent/Unavailable		O	➡ Schedule revisit (Date/Time)

For peer review only - http://bmjopen.bmj.com/site/about/guidelines.xhtml

Site ___ ___ ___ Subarea ___ ___ ___ House number ___ ___ ___ ___ Interviewer ID ___ ___

4=Home/Respondent/Completed	O	
5=Home/Respondent/Refusal	O	Record the reason(s) for refusal; tick all that applies<br>O 1=No time to answer questions<br>O 2=Don't want to participate/answer questions<br><br>O 3=Other ________________<br>O 98=No response O 99=Don't know<br><br>➡ Visit a replacement household to the nearest right or left side of this household
6=Non-existing/Non-residential	O	➡ Visit a replacement household to the nearest right or left side of this household
**Attempt 3** Date ____/____/____ (DD/MM/YY)	Time _____/_____ AM/PM	Interviewer ID ___ ___
STATUS	Tick one	
1=Not home	O	➡ Visit a replacement household to the nearest right or left side of this household
2=Home/Non-Respondent	O	➡ Visit a replacement household to the nearest right or left side of this household
3=Home/Respondent/Unavailable	O	➡ Visit a replacement household to the nearest right or left side of this household
4=Home/Respondent/Completed	O	
5=Home/Respondent/Refusal	O	Record the reason(s) for refusal; tick all that applies<br>O 1=No time to answer questions<br>O 2=Don't want to participate/answer questions<br><br>O 3=Other ________________<br>O 98=No response O 99=Don't know<br><br>➡ Visit a replacement household to the nearest right or left side of this household
6=Non-existing/Non-residential	O	➡ Visit a replacement household to the nearest right or left side of this household

For peer review only

Site ___ ___ ___ Subarea ___ ___ ___ House number ___ ___ ___ ___ Interviewer ID ___ ___

FORM 2 – CONSENT FORM

Healthcare assessment in [add study site and -country]

Instructions to the interviewer

- Read "Form 2 – CONSENT FORM" to the respondent/ask the respondent to carefully read through "Form 2 – CONSENT FORM" if preferred.
- The respondent as defined for this investigation is an adult (at the country-specific legal age of majority) household member and decision-maker with respect to daily healthcare/healthcare utilization for the entire household and its members; it must not be e.g. a neighbour or a visitor.
- Assure that the respondent has fully understood the purpose, procedures, risks and benefits of this study prior to obtaining the respondent's consent to participate.
- The respondent must sign "Form 2 – CONSENT FORM"; if the respondent is unable to sign/illiterate, a thumbprint will serve in place of a signature and a study-independent literate adult witness (e.g. member of same household/neighbour) will sign "Form 2 – CONSENT FORM".
- Collect data about all household members regardless of whether all household members are present at the time of the interview; collecting and recording data only about individuals present at the time of the interview will result in biased data.
- Complete "Form 2 – CONSENT FORM" once for every household consented/enrolled in the study.
- Explain to the respondent that it is anticipated to revisit every household during a 2-year period; the study team will obtain a new consent for participation during each visit.
- "Site" and "Subarea" must be entered each as a 3-digit, "House number" as a 4-digit, "Interviewer ID" as a 2-digit code (use lists individually prepared for each site to obtain codes), and "Date" in the format DD/MM/YYYY. "Study label" must be a combination of a 3-digit "Site" code, a 3-digit "Subarea" code and a 4-digit "House number" code; the "Interviewer ID" must be entered as a 3-digit code.

Study principal- and co-investigators

[add names of study principal- and co-investigators and respective affiliations]

Introduction

The International Vaccine Institute (IVI) and the [add affiliation(s) of local investigators] are working together to look into health problems and healthcare seeking behaviour in your community [add study site and -country], including the socio-economic status, hygiene and sanitation behaviour, and the vaccination status among children. We would like to better understand where you, your family and your neighbours seek healthcare in case of medical conditions associated with fever/hot body and other signs & symptoms. This information will help us to find out how common fever-related and other conditions are, provide a better picture of influencing factors involved in disease transmission and infection, and to find ways to prevent and treat illnesses in your community. You, the primary decision-maker for daily healthcare/healthcare utilization for this household and its members, are invited to participate because you live in the area where we are conducting this study. This consent form will be read by you/to you and you should take your time to make your decision to accept or decline the participation in the study. Please ask members of the study team to explain any word(s) or information that you do not understand. Further information about this study, including its potential risks, benefits and inconveniences will be explained subsequently. After the study has been fully explained to you and if you agree to participate, you will be asked to sign this consent form on behalf of all household members. If you are unable to sign the form, we will ask you to provide your thumbprint, and a study-independent adult person (member of your household/neighbour) will sign the form as a witness. We will give you a signed and dated copy of the consent form.

Purpose

The purpose of this study is to look into health problems and utilization of healthcare in your community [add study site and -country], including the socio-economic status and hygiene and sanitation behaviour. We aim to better understand where you, your family and your neighbours seek healthcare for conditions associated with fever/hot body or other sings & symptoms. This information will help better estimate the burden of illnesses common in your community, and to identify ways to introduce measures to prevent and treat them.

For peer review only - http://bmjopen.bmj.com/site/about/guidelines.xhtml

Site ___ ___ ___ Subarea ___ ___ ___ House number ___ ___ ___ ___ Interviewer ID ___ ___

Procedures

We will visit about [add number for study site and -country] households in total in [add study site and -country] and will ask the same questions to each household. Your household was chosen for this study simply by chance. If you agree to participate, we will ask you on behalf of all household members a few questions about your household such as the initials, sex and age of each member. We would like to know some information related to the education and occupation of the household head, and income, possession of household items, the housing itself as well as hygiene and sanitation in the context of personal hygiene, food preparation and handling, water sources and handling, waste disposal, handling of farm and domestic animals as well as farming of this household. We will also ask where members of your household get help if anyone is sick due to fever-related and other conditions and about factors that may influence healthcare seeking behaviour of household members (i.e. travel modalities, availability of health insurance, cultural/ethnographic factors) besides the immunization status of children of this house and the perception of some diseases. Finally, we will measure the geographic location of your house. There are no right or wrong answers. You are free to choose which questions to answer. All in all, it should take about 30-45 minutes to answer all questions. The interview may take a bit longer if there are many members in this household. Also, we will visit your household two times in different seasons (once during the dry season and once towards the end of the rainy season) during the overall study period of about two years to collect data as described in this paragraph of the consent form.

Participation

You, the primary decision-maker for daily healthcare/healthcare utilization for this household and its members, and members of your household are free to choose to accept or decline to be part of the study. If you do not want to participate, that is ok. If you want to stop your participation at any time, that is ok, and there will be no harm to you or members of your household. So, participation in this study is voluntary, and you are free to refuse to participate in the study or you can withdraw your consent at any time without giving reasons and this will not involve any penalty.

Risks & Benefits

There are no direct risks for participating in this study. Asking questions about your household may make you or household members feel uncomfortable. Study teams may help providing information on established community healthcare resources for participants that express distress after completing the questions. You can refuse to answer any question at any time. You may take a break or stop participating in this study at any time. There are no direct benefits for being in this study. During visits of your household, our study teams may identify members of your household that should seek healthcare for fever-associated conditions and others. Hence, they may help providing information on established healthcare resources in your community. Indirect benefits are that all information collected will provide exact information on health problems and healthcare utilization for various conditions, including vaccination for young children, the socio-economic status as well as hygiene and sanitation behaviour in your community. This will help to provide a better picture of influencing factors involved in disease transmission and infection, and to estimate the burden of common diseases as well as to find ways to prevent and treat those in your community.

Costs & Compensation

There are no costs for participants of this study. You and anyone from your household will not be compensated for your participation.

Confidentiality

All study records and all collected information that identify you and members of your household will be kept confidential. All paper-based files will be locked safely and computerized files will be password-protected; all data files (paper-based/computerized) will be stored for a minimum of five years; these files will be accessible to authorized few study staff only. Your name or any identifier will not be used in any publication or reports from this study. Information that we collect about you and members of your household will only be shared with authorized study members such as authorized study staff and officials from ethics committees.

For peer review only - http://bmjopen.bmj.com/site/about/guidelines.xhtml

Site ___ ___ ___ Subarea ___ ___ ___ House number ___ ___ ___ ___ Interviewer ID ___ ___

Contact point for questions

If you or members of your household have any questions, please ask them now to the study team or at any time later by contacting [add name of local principal- and/or co-investigator, respective affiliations, and phone numbers]. If you have questions as a study participant about your/your household members' rights, you can contact the [add name and contact details of the local ethics committee].

Statement of consent

This consent form for participating in the assessment of health behaviour and other population-based factors in [add study site and -country] has been read by me/to me carefully. The purpose, procedures, risks & benefits have been explained to me in detail. I have been allowed to ask questions at any time, and my questions have been answered to my satisfaction by the study team. I have been told whom to contact if I have questions, want to discuss problems or concerns. I have been told that I will be given a signed and dated copy of this consent form. I have been reassured that all information obtained as result of this study will be kept confidential and used only for the purpose of this study and only by participating institutions.

I, the respondent, consent hereby voluntarily to participate in this study. I will follow the directions of the study team and give them my full cooperation. I understand that I have the right to withdraw from the study at any time.

RESPONDENT

Name: ____
(given name + middle name (if available) + family name)

Signature: ____
(handwritten depiction of person's name/mark that the person writes on a document as a proof of identity)

Date ______/______/_______________ (DD/MM/YYYY)

Thumbprint

If the respondent is unable to sign/illiterate but agrees to participate, record the respondent's thumb print in the above box, and have an adult study-independent literate witness (e.g. member of the same household or a neighbor) sign below:

WITNESS

Name: ____
(given name + middle name (if available) + family name)

Signature: ____
(handwritten depiction of person's name/mark that the person writes on a document as a proof of identity)

Date ______/______/_______________ (DD/MM/YYYY)

I, the interviewer, have read/explained the study to the above named respondent (witness if respondent is illiterate) in a language that the respondent understands well. I am certain that the respondent has understood the information and he/she agrees to be asked some questions.

INTERVIEWER

Name: ____
(given name + middle name (if available) + family name)

Signature: ____
(handwritten depiction of person's name/mark that the person writes on a document as a proof of identity)

Date ______/______/_______________ (DD/MM/YYYY)

For peer review only

For peer review only - http://bmjopen.bmj.com/site/about/guidelines.xhtml

Site ___ ___ ___ Subarea ___ ___ ___ House number ___ ___ ___ ___ Interviewer ID ___ ___

FORM 3: GENERAL HOUSEHOLD INFORMATION

Instructions to the interviewer

- Complete this form only if the respondent consented to participate (see "Form 2 – CONSENT FORM"); the respondent as defined for this investigation is an adult (at the country-specific legal age of majority) household member and decision-maker with respect to daily healthcare/healthcare utilization for the entire household and its members; it must not be e.g. a neighbour or a visitor.
- Collect data about all household members regardless of whether all household members are present at the time of the interview; collecting and recording data only about individuals present at the time of the interview will result in biased data.
- A household is/household members are defined as a person or a group of related or unrelated persons that live together in the same dwelling unit, that acknowledge one male or female adult as the head of the household, that share the same housekeeping arrangements, that are considered to constitute one unit, and that provide themselves with food or other essentials for living.
- "Site" and "Subarea" must be entered each as a 3-digit, "House number" as a 4-digit, and "Interviewer ID" as a 2-digit code (use lists individually prepared for each site to obtain codes). "Study label" must be a combination of a 3-digit "Site" code, a 3-digit "Subarea" code and a 4-digit "House number" code; the "Interviewer ID" must be entered as a 3-digit code.

FORM 3: GENERAL HOUSEHOLD INFORMATION
PART A – DEMOGRAPHIC DATA/GENERAL HOUSEHOLD INFORMATION

HOUSE LOCATION

1 Site, subarea and house number

Instructions: Site (=study site) and subarea (=smallest unit of the study site) must be recorded each as a 3-digit, and house number as a 4-digit code; use individually prepared lists to obtain the respective codes.

Site: ____ ____ ____ Subarea: ____ ____ ____ House number: ____ ____ ____ ____

2 GPS coordinates

Instructions: Take GPS coordinates at the approx. center of the house. Record altitude as a 4-digit number (unit: meter), and latitude/longitude as a 7-digit number (unit: degrees, decimal minutes).

O 1=Original household O 2=Replacement household (right/left side of original household)

Altitude: ___ ___ ___ ___

Latitude: ___ ___°___ ___.___ ___ ___' Longitude: ___ ___°___ ___.___ ___ ___'

RESPONDENT

3 Provide the full name of the respondent.

Instructions: Record given name, middle name (if available) and family name of the respondent.

____

4 Provide the age of the respondent.

Instruction: Recorded as a 3-digit number (unit: year). ____ ____ ____

5 Provide the sex of the respondent.

O 1=Male O 2=Female

6 What is the respondent's relationship to this household?

O 1=Household head
O 2=Son/daughter of household head
O 3=Brother/sister of household head
O 4=Mother/father of household head
O 5=Granddaughter/grandson of household head
O 6=Other, specify __________
O 7=Spouse of household head
O 8=Spouse of son/daughter of household head
O 9=Spouse of brother/sister of household head
O 10=Mother/father in law of household head
O 11=Niece/nephew of household head
O 98=No response

For peer review only - http://bmjopen.bmj.com/site/about/guidelines.xhtml

Site ___ ___ ___ Subarea ___ ___ ___ House number ___ ___ ___ ___ Interviewer ID ___ ___

HOUSEHOLD MEMBERS

7 How many household members are living in this house in total (at the day of visit)**?**

Instruction: Recorded as a 2-digit number.

Total number of household members: ____ ____

8 Record age and sex of each household member.

Instructions: Record the respondent's initials, age and sex at the first position/row of the table. Initials, age and sex of all other household members must be recorded subsequently. The initials of a household member consist of the first three letters of the given name and the first three letters of the family name (a potential middle name is not part of the initials). Record the age of each household member as a 3-digit number (unit: year); if a household member's age is ≤12 months, record the age as 1 year. Please complete an additional form if a household has >20 members.

**Household member identifier**	**Initials** [given name/family name]	**Age** [year]	**Sex**
1/Respondent	____ ____ ____/____ ____ ____	____ ____ ____	O1=Male O2=Female
2	____ ____ ____/____ ____ ____	____ ____ ____	O1=Male O2=Female
3	____ ____ ____/____ ____ ____	____ ____ ____	O1=Male O2=Female
4	____ ____ ____/____ ____ ____	____ ____ ____	O1=Male O2=Female
5	____ ____ ____/____ ____ ____	____ ____ ____	O1=Male O2=Female
6	____ ____ ____/____ ____ ____	____ ____ ____	O1=Male O2=Female
7	____ ____ ____/____ ____ ____	____ ____ ____	O1=Male O2=Female
8	____ ____ ____/____ ____ ____	____ ____ ____	O1=Male O2=Female
9	____ ____ ____/____ ____ ____	____ ____ ____	O1=Male O2=Female
10	____ ____ ____/____ ____ ____	____ ____ ____	O1=Male O2=Female
11	____ ____ ____/____ ____ ____	____ ____ ____	O1=Male O2=Female
12	____ ____ ____/____ ____ ____	____ ____ ____	O1=Male O2=Female
13	____ ____ ____/____ ____ ____	____ ____ ____	O1=Male O2=Female
14	____ ____ ____/____ ____ ____	____ ____ ____	O1=Male O2=Female
15	____ ____ ____/____ ____ ____	____ ____ ____	O1=Male O2=Female
16	____ ____ ____/____ ____ ____	____ ____ ____	O1=Male O2=Female
17	____ ____ ____/____ ____ ____	____ ____ ____	O1=Male O2=Female
18	____ ____ ____/____ ____ ____	____ ____ ____	O1=Male O2=Female
19	____ ____ ____/____ ____ ____	____ ____ ____	O1=Male O2=Female
20	____ ____ ____/____ ____ ____	____ ____ ____	O1=Male O2=Female

For peer review only

For peer review only - http://bmjopen.bmj.com/site/about/guidelines.xhtml

Site ___ ___ ___ Subarea ___ ___ ___ House number ___ ___ ___ ___ Interviewer ID ___ ___

FORM 3: GENERAL HOUSEHOLD INFORMATION
PART B – SOCIO-ECONOMIC DATA

EDUCATION

1 Can the head of household read in [add the official language of the study site/country]**?**
O 1=Yes O 2=No O 99=Don't know O 98=No response

2 Can the head of household write in [add the official language of the study site/country]**?**
O 1=Yes O 2=No O 99=Don't know O 98=No response

3 What is the highest completed level of formal school education of the head of household?
O 1=Primary school O 4=Higher than secondary school O 99=Don't know
O 2=Middle school O 5=NO EDUCATION O 98=No response
O 3=Secondary school O 6=Other, specify ___________

OCCUPATION/INCOME

4 What is the main occupation of the household head?
Instructions: Record the status of the day of visit only.

O 1=Group 1: Managers
(chief executives, senior officials, legislators; administrative and commercial managers; production and specialized services managers; hospitality, retail and other services managers)

O 2=Group 2: Professional
(science and engineering professionals; health professionals; teaching professionals; business and administration professionals; information and communications technology professionals; legal, social and cultural professionals)

O 3=Group 3: Technicians and Associate Professionals
(science and engineering associate professionals; health associate professionals; business and administration associate professionals; legal, social, cultural and related associate professionals; information and communications technicians)

O 4=Group 4: Clerical Support Workers
(general and keyboard clerks; customer services clerks; numerical and material recording clerks; other clerical support workers)

O 5=Group 5: Services and Sales Workers
(personal services workers; sales workers; personal care workers; protective services workers)

O 6=Group 6: Skilled Agricultural, Forestry, and Fishery Workers
(market-oriented skilled agricultural workers; animal producers; marked-oriented skilled forestry, fishery and hunting workers; subsistence farmers, fishers, hunters and gatherers)

O 7=Group 7: Craft and Related Trades Workers
(building and related trades workers (excluding electricians); metal, machinery and related trades workers; handicraft and printing workers; electrical and electronics trades workers; food processing, woodworking, garment and other craft and related trades workers)

O 8=Group 8: Plant and Machine Operators, and Assemblers
(stationary plant and machine operators; assemblers; drivers and mobile plant operators)

O 9=Group 9: Elementary Occupations
(cleaners and helpers; agricultural, forestry and fishery labourers; labourers in mining, construction, manufacturing and transport; food preparation assistants; street and related sales and services workers; refuse workers and other elementary workers)

O 10=Group 0: Armed Forces Occupations
(commissioned armed forces officers; non-commissioned armed forces officers; armed forces occupations, other ranks)

O 11=Other, specify __________

O 12=NO OCCUPATION O 99=Don't' know O 98=No response

For peer review only

For peer review only - http://bmjopen.bmj.com/site/about/guidelines.xhtml

Site ___ ___ ___ Subarea ___ ___ ___ House number ___ ___ ___ ___ Interviewer ID ___ ___

5 What is the average monthly income of the household head based on the occupation stated in question 4 and of other household members?
Instruction: Tick one answer per household head/enter one code per household member if applicable/if income available.

Head of household	Member 1	Member 2	Member 3
Code ____________	Code ____________	Code ____________	Code ____________

[1] <100USD [3] ≥150 to <200USD [5] ≥250USD [99] Don't know
[2] ≥100 to <150USD [4] ≥200 to <250USD [6] Head of household NO INCOME (see Q4) [98] No response

6 Which and how much other/additional source(s) of monthly monetary earnings has this household?
Instruction: Tick all that applies; more than one answer is possible.

Other/additional sources	Overall monthly monetary earnings
O 1=Social grants<br>O 2=Insurance (i.e. private, public)<br>O 3=Loan (i.e. from friends, family, bank)<br>O 4=Selling (i.e. livestock, crops, furniture, other goods)<br>O 5=Personal savings<br>O 6=Other, specify ________________	O 1=<100 USD<br>O 2=≥100 to <150 USD<br>O 3=≥150 to <200 USD<br>O 4=≥200 to <250 USD<br>O 5=≥250 USD
O 7=NO OTHER/ADDITIONAL SOURCES<br>O 99=Don't know<br>O 98=No response	

For peer review only

HOUSING/DWELLING

7 Is the house owned by the members of this household?
O 1=Yes O 3=No, supplied (i.e. government, employer) O 99=Don't know
O 2=No, rented O 4=No, other, specify ________ O 98=No response

8 How many room(s)/individual house structure(s) of this household are used for sleeping, living, cooking and bathing?
Instruction: Enter one code per category only.

Bedroom	Living room	Kitchen	Bathroom
Code ____________	Code ____________	Code ____________	Code ____________

[1] 1-2 [3] >4 [99] Don't know
[2] 3-4 [4] None [98] No response

9 What is the construction material of the floor, the walls and the roof of room(s)/individual house structure(s) of this house?
Instruction: Enter all codes that applies per category; more than one answer per category is possible.

Floor	Walls	Roof
Code ____________	Code ____________	Code ____________

[1] Brick [3] Tiles [5] Wood [7] Metal/Tin [99] Don't know
[2] Cement/Concrete [4] Mud [6] Straw/Leaves [8] Other, specify ________ [98] No response

10 Do members of this household have access to/utilize electricity in this household?
O 1=Yes O 2=No O 99=Don't know O 98=No response

11 Which main source of light have members of this household access to/are utilizing?
O 1=Electric light/Light bulb(s) O 4=Battery-powered lamp(s) O 7=Other, specify ________
O 2=Solar light/Solar lamp(s) O 5=Candle(s) O 99=Don't know
O 3=Paraffin lamp(s)/Oil lamp(s) O 6=NO SOURCE OF LIGHT O 98=No response

Site ___ ___ ___ Subarea ___ ___ ___ House number ___ ___ ___ ___ Interviewer ID ___ ___

HOUSEHOLD ASSETS

12 Which of the following items do members of this household own?

Instructions: Tick all that applies; more than one answer is possible; record the quantity of functional items where applicable.

O 1=Radio	Quantity ____	O None	O 11=Tape/CD player	Quantity ____	O None
O 2=Television	Quantity ____	O None	O 12=Computer/Laptop	Quantity ____	O None
O 3=Internet connection	Quantity ____	O None	O 13=Mobile/Cell phone	Quantity ____	O None
O 4=Car	Quantity ____	O None	O 14=Truck/Bus	Quantity ____	O None
O 5=Motorbike	Quantity ____	O None	O 15=Bicycle	Quantity ____	O None
O 6=Oxcart/Donkey	Quantity ____	O None	O 16=Bed	Quantity ____	O None
O 7=Mattress	Quantity ____	O None	O 17=Bed net	Quantity ____	O None
O 8=Table	Quantity ____	O None	O 18=Chair	Quantity ____	O None
O 9=Refrigerator (4°C)	Quantity ____	O None	O 19=Freezer (-20°C)	Quantity ____	O None
O 10=Fan	Quantity ____	O None	O 20=Sewing machine	Quantity ____	O None

For peer review only

For peer review only - http://bmjopen.bmj.com/site/about/guidelines.xhtml

Site ___ ___ ___ Subarea ___ ___ ___ House number ___ ___ ___ ___ Interviewer ID ___ ___

FORM 3: GENERAL HOUSEHOLD INFORMATION
Part C – Hygiene and sanitation

TOILET FACILITY

1 To what type of toilet facility have members of this household usually access?
Instruction: Inside means inside a room/individual house structure/building; outside means outside a room/individual house structure/building.

- O 1=Flush toilet/Water closet (inside)
- O 2=Flush toilet/Water closet (outside)
- O 3=Non-flush latrine/pit latrine (inside)
- O 4=Non-flush latrine/pit latrine (outside)
- O 5=Ventilated Improved Pit Latrine (KVIP, inside)
- O 6=Ventilated Improved Pit Latrine (KVIP, outside)
- O 7=Composting toilet (inside)
- O 8=Composting toilet (outside)
- O 9=NO toilet facility, use a bucket
- O 10=NO toilet facility, dig a hole
- O 11=NO toilet facility, free range/open defecation
- O 12=Other, specify __________
- O 99=Don't know
- O 98=No response

2 Do members of this household usually utilize the toilet facility stated in question 1?

- O 1=No, members cannot utilize the facility independently (i.e. infants, toddlers)
- O 2=No, members cannot utilize the facility independently (i.e. disabled, long-term/chronically diseased)
- O 3=No, members cannot utilize the facility independently (i.e. seniors)
- O 4=No, limited access (i.e. public, shared with other households/community)
- O 5=No, too far away (i.e. public, shared with other households/community)
- O 6=No, bad condition (i.e. broken, not clean)
- O 7=YES
- O 8=Other, specify _________________
- O 99=Don't know
- O 98=No response

3 Where is the toilet facility stated in question 1 located? With how many other people is it usually shared?

- O 1=On the property of this household (private, not shared)
- O 2=On the property of a neighbouring household (private, shared)
  - *Shared* O 1=<10 people O 2=11-24 people O 3=25-49 people O 4=>50 people O 99=Don't know O 98=No response
- O 3=In the community (public, shared)
  - *Shared* O 1=<10 people O 2=11-24 people O 3=25-49 people O 4=>50 people O 99=Don't know O 98=No response
- O 4=Other, specify __________ O 99=Don't know O 98=No response

4 How do members of this household that have/don't have access to a toilet facility usually clean their hands directly before and after urination/defection?
Instruction: Enter one code per category only.

Clean hands before urination/defection	Clean hands after urination/defection
Code _____________	Code _____________
[1] Wash hands with soap and water<br>[2] Wash hands with water only<br>[3] Rub hands clean with some leaves/straw/ grass<br>[4] Rub hands clean with some sand<br>[5] Rub hands clean with some cloth/fabric	[6] Don't clean, not needed/not dirty<br>[7] Don't clean, nothing to clean hands nearby/on hands<br>[8] Other, specify __________<br>[99] Don't know<br>[98] No response

KITCHEN/FOOD HANDLING

5 Do members of this household have a kitchen for cooking/preparing food/meals? Where is it located?

- O 1=Yes, inside kitchen/in a room/individual house structure; on the property of this household (private, not shared)
- O 2=Yes, outside kitchen/outside a room/individual house structure/ open air; on the property of this

For peer review only - http://bmjopen.bmj.com/site/about/guidelines.xhtml

Site ___ ___ ___ Subarea ___ ___ ___ House number ___ ___ ___ ___ Interviewer ID ___ ___

household (private, not shared)
O 3=Yes, inside kitchen/in a room/individual house structure; on the property of a neighbouring household (private, shared)
O 4=Yes, outside kitchen/outside a room/individual house structure/ open air; on the property of a neighbouring household (private, shared)
O 5=NO KITCHEN
O 6=Other, specify __________ O 99=Don't know O98=No response

6 What energy source do members of this household usually use for cooking/preparing food/meals?

O 1=Electricity O 3=Charcoal O 5=Animal dung O 99=Don't know
O 2=Gas/Kerosene O 4=Wood/straw/leaves O 6=Other, specify _____ O 98=No response

7 What cooking spot do members of this household usually use for cooking/preparing food/meals?

O 1=Open fire O 3=Closed stove with chimney O 99=Don't know
O 2=Open stove O 4=Other, specify __________ O 98=No response

8 Where do members of this household usually store fresh/raw food items (e.g. vegetables, lettuce, fruits, dairy products, eggs, meat and fish) and left-over/prepared food/meals?

Instruction: Enter one code per category only. Inside means inside a room/individual house structure/building; outside means outside a room/individual house structure/building.

Fresh/raw food items (i.e. vegetables, lettuce, fruits, dairy products, eggs, meat and fish)	Left-over/prepared food/meals
Code _____________	Code _____________

[1] At room temperature (inside, on the property of this household, private)
[2] At room temperature (outside, on the property of this household, private)
[3] At room temperature (inside, on the property of a neighboring household, private, shared)
[4] At room temperature (outside, on the property of a neighboring household, private, shared)
[5] Refrigerator (on the property of this household, private)
[6] Refrigerator (on the property of a neighboring household, private, shared)
[7] Freezer (on the property of this household, private)
[8] Freezer (on the property of a neighboring household, private, shared)
[9] Other, specify _______________
[10] NO STORAGE [99] Don't know [98] No response

9 If the answer is "Room temperature" for fresh/raw food items and/or left-over/ prepared food/meals in question 8, how long do members of this household usually store them?

Instruction: Enter one code per category only.

Fresh/raw food items (i.e. vegetables, lettuce, fruits, dairy products, eggs, meat and fish)	Left-over/prepared food/meals
Code _____________	Code _____________

[1] <1 day [3] 3-4 days [5] Not applicable [99] Don't know
[2] 1-2 days [4] >4 days [98] No response

10 Do members of this household usually cover left-over/prepared food/meals? Do they re-heat/re-cook left-over/prepared food/meals prior to consumption?

Instruction: Enter one code per category only.

Cover left-over/prepared food/meals	Re-heat/Re-cook left-over/prepared food/meals
Code _____________	Code _____________

[1] Yes [2] No [99] Don't know [98] No response

For peer review only - http://bmjopen.bmj.com/site/about/guidelines.xhtml

Site ___ ___ ___ Subarea ___ ___ ___ House number ___ ___ ___ ___ Interviewer ID ___ ___

11 Which member(s) of this household usually cook(s)/prepare(s) food/meals for other members of this household?

Instructions: Refer to question 9 of "Form 3 Part A – Demographic data/General household information" and record the initials/identifier(s) of the member.

O Household member, record initials/identifier(s) __________ O 99=Don't know O 98=No response

12 How does/do the household member(s) that usually cook(s)/prepare(s) food/meals for other members of this household as stated in question 11 usually prepare(s) herself/himself/themselves before and after the preparation of meals?

Instruction: Enter one code per category only.

Before the preparation of meals	After the preparation of meals
Code _____________	Code _____________
[1] Wash hands with soap and water<br>[2] Wash hands with water only<br>[3] Rub hands clean with some leaves/straw/ grass<br>[4] Rub hands clean with some sand<br>[5] Rub hands clean with some cloth/fabric	[6] Don't clean, not needed/not dirty<br>[7] Don't clean, nothing to clean hands nearby/on hands<br>[8] Other, specify __________<br>[99] Don't know<br>[98] No response

13 How do members of this household usually prepare themselves before and after eating food/meals, including the household member that usually cooks/prepares food/meals as stated in question 11?

Instruction: Enter one code per category only.

Before the eating of food/meals	After the eating of food/meals
Code _____________	Code _____________
[1] Wash hands with soap and water<br>[2] Wash hands with water only<br>[3] Rub hands clean with some leaves/straw/ grass<br>[4] Rub hands clean with some sand<br>[5] Rub hands clean with some cloth/fabric	[6] Don't clean, not needed/not dirty<br>[7] Don't clean, nothing to clean hands nearby/on hands<br>[8] Other, specify __________<br>[99] Don't know<br>[98] No response

14 How do members of this household usually eat their food/meals, including the household member that usually cooks/prepares food/meals as stated in question 11?

O 1=With cutlery O 3=Other, specify _____________ O 99=Don't know
O 2=With fingers O 98=No response

15 Do members of this household usually eat their food/meals from the same pot, including the household member that usually cooks/prepares food/meals as stated in question 11?

O 1=Yes O 2=No O 99=Don't know O 98=No response

16 Where do members of this household usually get their main fresh/raw food items such as vegetables, fruits and grains/cereals, dairy products, eggs, meat and fish?

Instruction: Enter one code per category only.

Vegetables, fruits, grains/ cereals	Dairy products	Eggs, meat, fish
Code _____________	Code _____________	Code _____________

[1] Self-farming (i.e. back-/courtyard)
[2] Supermarket/Grocery store
[3] Friend/Family
[4] Open market
[5] Street vendor
[6] Self-farming (i.e. agricultural land)
[7] Butcher
[8] Other, specify ______________
[99] Don't know
[98] No response

17 Do members of this household usually consume/eat fresh/raw food items (e.g. vegetables, lettuce, fruits, dairy products, ice cream, eggs, meat and fish)? If so, which ones?

For peer review only - http://bmjopen.bmj.com/site/about/guidelines.xhtml

Site ___ ___ ___ Subarea ___ ___ ___ House number ___ ___ ___ ___ Interviewer ID ___ ___

Instruction: Tick all that applies; more than one answer is possible.

- O 1=Vegetables/Lettuce (cultivated on bush)
- O 2=Vegetables/Lettuce (wild harvesting from bush)
- O 3=Fruits (cultivated on bush/tree)
- O 4=Fruits (wild harvesting from bush/tree)
- O 5=Dairy products (i.e. milk, cheese, yoghurt from domesticated milk producing animals), specify _____________
- O 6=Eggs (from domesticated poultry), specify __________
- O 7=Animal blood (from domesticated animals), specify __________
- O 8=Beef/Zebu/Buffalo (domesticated), specify __________
- O 9=Pork (domesticated)
- O 10=Sheep (domesticated)
- O 11=Meat from non-domesticated animals (i.e. antelope, boar, poultry, rodents, fish), specify _________
- O 12=NO CONSUMPTION of fresh/raw food items
- O 13=Vegetables/Lettuce (cultivated on ground)
- O 14=Vegetables/Lettuce (wild harvesting from ground)
- O 15=Fruits (cultivated on ground)
- O 16=Fruits (wild harvesting from ground)
- O 17=Dairy products (i.e. milk, cheese, yoghurt from non-domesticated milk producing animals), specify _____________
- O 18=Eggs (from non-domesticated poultry), specify __________
- O 19=Animal blood (from non-domesticated animals), specify __________
- O 20=Poultry (domesticated; i.e. chicken, duck, pigeon, goose, turkey), specify __________
- O 21=Goat (domesticated)
- O 22=Rodents (domesticated), specify __________
- O 23=Fish/Seafood, specify __________
- O 24=Ice cream
- O 25=Other, specify __________
- O 99=Don't know O 98=No response

18 Do members of this household consume meals/food items/beverages usually also outside the house? If so, which member(s) of this household and where?

Instructions: Refer to question 9 of "Form 3 Part A – Demographic data/General household information" and record the initials/identifier of the member(s). Enter all codes that apply for the respective household member.

Member 1<br>Initials/Identifier: _____	Member 2<br>Initials/Identifier: _____	Member 3<br>Initials/Identifier: _____	Member 4<br>Initials/Identifier: _____
Code _____________	Code _____________	Code _____________	Code _____________
[1] Restaurant<br>[2] Fast food chain	[3] Cafeteria<br>[4] Street vendor	[5] Other, specify ____<br>[6] Not applicable	[99] Don't know<br>[98] No response

19 Which meals/food items/beverages do the member(s) of this household listed in question 18 usually consume outside the house?

Instructions: Refer to question 9 of "Form 3 Part A – Demographic data/General household information" and record the initials/identifier of the member(s). List up to 5 meals/food items/beverages per household member. You can refer to food items listed in question 17. Skip this question of no household member consumes meals/food items/beverages outside the house as stated in Q18.

Member 1<br>Initials/Identifier: _____	Member 2<br>Initials/Identifier: _____	Member 3<br>Initials/Identifier: _____	Member 4<br>Initials/Identifier: _____
1: _______________<br>2: _______________<br>3: _______________<br>4: _______________<br>5: _______________	1: _______________<br>2: _______________<br>3: _______________<br>4: _______________<br>5: _______________	1: _______________<br>2: _______________<br>3: _______________<br>4: _______________<br>5: _______________	1: _______________<br>2: _______________<br>3: _______________<br>4: _______________<br>5: _______________

WATER

20 What is the main water supply for members of this household for drinking, cooking/ preparing food/meals, bathing, and cleaning (of house/individual house structure(s), clothing/kitchen tools)?

Instruction: Enter one code per category only.

Drinking	Cooking	Bathing	Cleaning
Code _____________	Code _____________	Code _____________	Code _____________

[1] Tap water (inside the house; private)
[2] Tap water (outside the house i.e. back-/courtyard; private)

For peer review only - http://bmjopen.bmj.com/site/about/guidelines.xhtml

Site ___ ___ ___ Subarea ___ ___ ___ House number ___ ___ ___ ___ Interviewer ID ___ ___

[3] Tap water (outside the house; public/shared)
[4] Standpipe/Piped water (inside the house; private)
[5] Standpipe/Piped water (outside the house i.e. back-/ courtyard; private)
[6] Standpipe/Piped water (outside the house; public/shared)
[7] Water truck (public; shared)
[8] Surface water (i.e. canal, irrigation canal)
[9] Surface water (i.e. lake, pond, river, stream)
[10] Spring water
[11] Rain water
[12] Well/Borehole (open/uncovered/unprotected)
[13] Well/Borehole (covered/protected)
[14] Bottled water
[15] Other, specify ________________ [99] Don't know [98] No response

21 Do members of this household usually treat water for drinking, cooking/ preparing food/meals, bathing, and cleaning (of house/individual house structure(s), clothing/ kitchen tools) prior to consumption? How is water usually treated?

Instruction: Enter all codes that applies per category; more than one answer per category is possible.

Drinking	Cooking	Bathing	Cleaning
Code ____________	Code ____________	Code ____________	Code ____________

[1] No, not at all
[2] No, only for sick members of this household
[3] No, only depending on the season (dry season)
[4] Yes, filtration (i.e. cloth, ceramic, sand)
[5] Yes, sedimentation
[6] Other, specify ________________
[7] No, only for children of this household
[8] No, only depending on the season (rainy season)
[9] Yes, boiling
[10] Yes, solar disinfection
[11] Yes, chlorination
[99] Don't know [98] No response

22 Which member(s) of this household usually collect(s) the drinking water as stated in question 20 for other members of this household?

Instructions: Refer to question 9 of "Form 3 Part A – Demographic data/General household information" and record the initials/identifier of the member.

O Household member, record initials/identifier(s) _________ O 99=Don't know O 98=No response

23 How do members of this household usually store the drinking water stated in question 20?

O 1=Water tank (uncovered)
O 2=Water tank (covered with i.e. cloth/fabric, lid, leaves)
O 3=Container (uncovered)
O 4=Container (covered with i.e. cloth/fabric, lid, leaves)
O 5=Clay pot (uncovered)
O 6=Clay pot (covered with i.e. cloth/fabric, lid, leaves)
O 7=Bucket (uncovered)
O 8=Bucket (covered with i.e. cloth/fabric, lid, leaves)
O 9=Bowl (uncovered)
O 10=Bowl (covered with i.e. cloth/fabric, lid, leaves)
O 11=Wooden pot (uncovered)
O 12=Wooden pot (covered with i.e. cloth/fabric, lid, leaves)
O 13=Skin vessel (i.e. goat, sheep, cattle; uncovered)
O 14=Skin vessel (i.e. goat, sheep, cattle; covered with i.e. cloth/fabric, lid, leaves)
O 15=NOT STORED
O 16=Other, specify _________
O 99=Don't know
O 98=No response

24 If members of this household use a shared drinking water supply as stated in question 20, with how many other people is this drinking water supply usually shared?

O 1=<10 people
O 2=11-24 people
O 3=25-49 people
O 4=>50 people
O 5=NOT SHARED
O 99=Don't know
O 98=No response

For peer review only - http://bmjopen.bmj.com/site/about/guidelines.xhtml

Site ___ ___ ___ Subarea ___ ___ ___ House number ___ ___ ___ ___ Interviewer ID ___ ___

WASTE DISPOSAL

25 Where do members of this household usually dispose waste water from e.g. cooking/ preparation of food/meals, bathing and/or cleaning, dispose domestic waste, human waste/excreta?

Instruction: Enter one code per category only.

Waste water from (e.g. cooking/ preparation of food/ meals, bathing and/or cleaning)	Domestic waste	Human waste/excreta
Code ____________	Code ____________	Code ____________

[1] On the ground/free range near the house (i.e. back-/courtyard, private)
[2] On the ground/free range away from the house in the community (shared, public)
[3] In a hole near the house (i.e. back-/courtyard, private)
[4] In a hole away from the house in the community (shared, public)
[5] Pour in surface water in the community (i.e. lake, pond, river, stream, public, shared)
[6] Pour in surface water in the community (i.e. canal, irrigation canal, public, shared)
[7] Pour in spring water in the community (public, shared)
[8] Pour in a septic tank near the house (i.e. back-/courtyard, near toilet facility if available, private)
[9] Pour in a septic tank in the community (shared, public)
[10] Pour in the sewer near the house (i.e. back-/courtyard, private)
[11] Pour in the sewer in the community (shared, public)
[12] Other, specify ______________________ [99] Don't know [98] No response

26 Do members of this household usually pre-process domestic waste from this house prior to disposing it?

O 1=Yes, burning garbage
O 2=Yes, separate/sort out degradable from non-degradable garbage
O 3=NO
O 4=Yes, other, specify _________
O 99=Don't know O 98=No response

27 How far from the main drinking water supply of this household as stated in question 20 is the location for disposal of waste water, domestic waste and human waste/excreta of this household as stated in question 25?

Instructions: Enter one code per category only. Record the distance as meter [m].

Waste water from (e.g. cooking/ preparation of food/ meals, bathing and/or cleaning)	Domestic waste	Human waste/excreta
Code ____________	Code ____________	Code ____________

[1] <5 m [3] ≥25 to <50 m [5] ≥100 to <500 m [7] ≥1,000 m
[2] ≥5 to <25 m [4] ≥50 to <100 m [6] ≥500 to <1,000 m [99] Don't know [98] No response

28 How far from the main water supply for cooking/preparing food/meals of this household as stated in question 20 is the location for disposal of waste water, domestic waste and human waste/excreta of this household as stated in question 25?

Instructions: Enter one code per category only. Record the distance as meter [m].

Waste water from (e.g. cooking/preparation of food/ meals, bathing and/or cleaning)	Domestic waste	Human waste/excreta
Code ____________	Code ____________	Code ____________

[1] <5 m [3] ≥25 to <50 m [5] ≥100 to <500 m [7] ≥1,000 m
[2] ≥5 to <25 m [4] ≥50 to <100 m [6] ≥500 to <1,000 m [99] Don't know [98] No response

FARM/DOMESTIC ANIMALS

29 What kind of farm animals/livestock and domestic animals/pets do members of this household have?

Instruction: Tick all that applies per category; more than one answer per category is possible.

For peer review only - http://bmjopen.bmj.com/site/about/guidelines.xhtml

Site ___ ___ ___ Subarea ___ ___ ___ House number ___ ___ ___ ___ Interviewer ID ___ ___

Farm animals/livestock		Domestic animals/pets
O 1=Cattle	O 9=Camel	O 1=Dog
O 2=Sheep	O 10= Rodents (i.e. rabbit), specify ______________________	O 2=Rodents (i.e. rabbit), specify ______________________
O 3=Donkey	O 11=Goat	O 3=Cat
O 4=Poultry (i.e. chicken, duck, pigeon, goose, turkey), specify ______________	O 12=Horse	O 4=NO DOMESTIC ANIMALS/ PETS
O 5=Zebu	O 13=NO FARM ANIMALS/ LIVESTOCK	O 5=Other, specify ______________________
O 6=Pig	O 14=Other, specify ______________	O 99=Don't know
O 7=Fish	O 99=Don't know	O 98=No response
O 8=Buffalo	O 98=No response	

30 Where do members of this household usually keep farm animals/livestock and domestic animals/pets of this household as stated in question 29?
Instruction: Tick one answer per category only.

Farm animals/livestock	Domestic animals/pets
O 1=Stable/Cage/Water basin on the property of the house (i.e. back-/courtyard)	O 1=Cage/Chain near the house (i.e. back-/courtyard)
O 2=Stable/Cage/Water basin outside the house in the community	O 2=Cage/Chain outside the house in the community
O 3=Stable/Cage/Water basin outside the community	O 3=Cage/Chain outside the community (public)
O 4=Free range/Surface water/Spring water on the property of the house (i.e. house, back-/courtyard)	O 4=Free range near the house (i.e. in the house, back-/courtyard)
O 5=Free range/Surface water/Spring water outside the house in the community	O 5=Free range outside the house in the community
O 6=Free range/Surface water/Spring water outside the community	O 6=Free range outside the community
O 7=Not applicable (nowhere/no farm animals/livestock)	O 7=Not applicable (nowhere/no domestic animals/pets)
O 8=Other, specify __________	O 8=Other, specify __________
O 99=Don't know	O 99=Don't know
O 98=No response	O 98=No response

31 What is the extent of the livestock farming that members of this household practice?

O 1=Own demand ("backyard farming")
O 2=Not applicable (no livestock farming)
O 3=Mass/Industrial demand ("mass animal farming")
O 4=Other, specify __________
O 99=Don't know
O 98=No response

32 Which water supply do members of this household usually use to water farm animals/livestock and domestic animals/pets of this household as stated in question 29?
Instruction: Enter one code per category only.

Farm animals/livestock	Domestic animals/pets
Code _____________	Code _____________

[1] Tap water (inside the house; private)
[2] Tap water (outside the house i.e. back-/courtyard; private)
[3] Tap water (outside the house; public/shared)
[4] Standpipe/Piped water (inside the house; private)
[5] Standpipe/Piped water (outside the house i.e. back-/ courtyard; private)
[6] Standpipe/Piped water (outside the house; public/shared)

For peer review only - http://bmjopen.bmj.com/site/about/guidelines.xhtml

Site ___ ___ ___ Subarea ___ ___ ___ House number ___ ___ ___ ___ Interviewer ID ___ ___

[7] Water truck (public; shared)
[8] Surface water (i.e. canal, irrigation canal)
[9] Surface water (i.e. lake, pond, river, stream)
[10] Spring water
[11] Rain water
[12] Well/Borehole (open/uncovered/unprotected)
[13] Well/Borehole (covered/protected)
[14] Not applicable (no farm animals/livestock; no domestic animals/pets)
[15] Other, specify ___________________ [99] Don't know [98] No response

33 Do members of this household usually wash their hands after they fed & watered and cleaned the stable/cage/water basin of the farm animals/livestock and domestic animals/pets of this household as stated in question 29?
Instruction: Enter one code per category only.

Farm animals/livestock	Domestic animals/pets
Code _____________	Code _____________
[1] Wash hands with soap and water<br>[2] Wash hands with water only<br>[3] Rub hands clean with some leaves/straw/ grass<br>[4] Rub hands clean with some sand<br>[5] Rub hands clean with some cloth/fabric<br>[6] Don't clean, not needed/not dirty	[7] Don't clean, nothing to clean hands nearby/on hands<br>[8] Other, specify _________<br>[9] Not applicable (no farm animals/livestock; no domestic animals/pets)<br>[99] Don't know<br>[98] No response

CROP FARMING

34 What kind of crops do members of this household grow/cultivate?
Instruction: Tick all that applies; more than one answer is possible.

O 1=Coffee
O 2=Rice
O 3=Fruits (cultivated on bush/tree)
O 4=Fruits (cultivated on the ground)
O 5=Vegetables/Lettuce (cultivated on bush/tree)
O 6=Vegetables/Lettuce (cultivated on the ground)
O 7=Grains (i.e. wheat, barley, oat, corn)
O 8=Tea
O 9=Cashew
O 10=Tobacco
O 11=Sugar cane
O 12=Chad/Chat
O 13=Bees/Honey
O 14=Cotton
O 15=NO CROPS
O 16=Other, specify __________
O 99=Don't know
O 98=No response

35 Where do members of this household have the farmland/agricultural land for crop farming?

O 1=On the property of the house (i.e. back-/courtyard)
O 4=Outside the house in the community
O 2=Outside the community
O 5=Not applicable (no crop farming)
O 3=Other, specify __________ O 99=Don't know O 98=No response

36 What is the extent of the crop farming that members of this household practice?

O 1=Own demand ("backyard farming")
O 2=Not applicable (no crop farming)
O 3=Mass/Industrial demand ("mass crop farming")
O 4=Other, specify __________
O 99=Don't know
O 98=No response

37 Do members of this household usually use herbicides, pesticides and fertilizers for the crop farming as stated in question 34?
Instruction: Tick one answer per category only.

Herbicides	Pesticides	Fertilizers
O 1=Yes, specify herbicide<br>_______________________	O 1=Yes, specify pesticide<br>_______________________	O 1=Yes, specify fertilizer<br>_______________________

For peer review only - http://bmjopen.bmj.com/site/about/guidelines.xhtml

Site ___ ___ ___ Subarea ___ ___ ___ House number ___ ___ ___ ___ Interviewer ID ___ ___

O 2=Not applicable (no crop farming)<br>O 3=No use of herbicides<br>O 99=Don't know<br>O 98=No response	O 2=Not applicable (no crop farming)<br>O 3=No use of pesticides<br>O 99=Don't know<br>O 98=No response	O 2=Not applicable (no crop farming)<br>O 3=No use of fertilizers<br>O 99=Don't know<br>O 98=No response

38 Do members of this household usually wash their hands after the fieldwork for the crop farming as stated in question 34?

O 1=Yes, wash hands with soap and water
O 2=Yes, rub hands clean with some leaves/straw/grass
O 3=Yes, rub hands clean with some cloth/fabric
O 4=Not applicable (no crop farming)
O 5=Yes, wash hands with water only
O 6=Yes, rub hands clean with some sand
O 7=No, don't clean my hands
O 8=Other, specify __________
O 99=Don't know
O 98=No response

For peer review only

For peer review only - http://bmjopen.bmj.com/site/about/guidelines.xhtml

Site ___ ___ ___ Subarea ___ ___ ___ House number ___ ___ ___ ___ Interviewer ID ___ ___

FORM 4: ASSESSMENT OF OVERALL, AGE- AND SEX-STRATIFIED GENERIC HEALTHCARE UTILIZATION

Part A – Assessment of *generic* healthcare utilization

Instructions to the interviewer

- Complete this form only if the respondent consented to participate (see "Form 2 - CONSENT FORM"); the respondent as defined for this investigation is an adult (at the country-specific legal age of majority) household member and decision-maker with respect to daily healthcare/healthcare utilization for the entire household and its members; it must not be e.g. a neighbour or a visitor.
- Refer to "Form 3 – Part A - GENERAL HOUSEHOLD INFORMATION" as a reference to obtain the total number of household members as well as age and sex of each household member.
- Collect data about all household members regardless of whether all household members are present at the time of the interview; collecting and recording data only about individuals present at the time of the interview will result in biased data.
- A household is/household members are defined as a person or a group of related or unrelated persons that live together in the same dwelling unit, that acknowledge one male or female adult as the head of the household, that share the same housekeeping arrangements, that are considered to constitute one unit, and that provide themselves with food or other essentials for living.
- "Site" and "Subarea" must be entered each as a 3-digit, "House number" as a 4-digit, and "Interviewer ID" as a 2-digit code (use lists individually prepared for each site to obtain codes). "Study label" must be a combination of a 3-digit "Site" code, a 3-digit "Subarea" code and a 4-digit "House number" code; the "Interviewer ID" must be entered as a 3-digit code.
- Abbreviation: HCF=private/public primary/secondary/tertiary healthcare facility.

HOUSEHOLD MEMBERS

1 Record the total number of household members of this house (at the day of visit)**.**

Instructions: The total number of household members must be recorded as a 2-digit number; see also "Form 3 – Part A - GENERAL HOUSEHOLD INFORMATION".

Total number of household members: ____ ____

2 Record the number of FEMALE household members by each of the following age groups:

Instructions: The total number of female household members by age group must be recorded as a 2-digit number; see also "Form 3 – Part A - GENERAL HOUSEHOLD INFORMATION".

<2years: ____ ____ ≥2 to <5years: ____ ____ ≥5 to <15years: ____ ____ ≥15years: ____ ____

3 Record the number of MALE household members by each of the following age groups:

Instructions: The total number of male household members by age group must be recorded as a 2-digit number; see also "Form 3 – Part A - GENERAL HOUSEHOLD INFORMATION".

<2years: ____ ____ ≥2 to <5years: ____ ____ ≥5 to <15years: ____ ____ ≥15years: ____ ____

HEALTHCARE SEEKING BEHAVIOR

4 Record where female/male household members <2years/≥2 to <5years/≥5 to <15years/≥15years of age usually seek healthcare for the following signs/symptoms. Please rate using a range from 1-4.

Instructions:

- Complete question 4 separately for female and male household members <2years, ≥2 to <5years, ≥5 to <15years and ≥15years of age and circle the sex and age group Q4 was completed for; skip question 4 if there is no female/male household member <2years/ ≥2 to <5years/ ≥5 to <15years/ ≥15years of age.
- Provide an answer to each healthcare option for each sign/symptom; use range 1-4 for rating (1=1st choice; 2=2nd choice; 3=3rd choice; 4=not chosen); enter a code where applicable by using prepared lists (use lists individually prepared for each site to obtain codes); tick "Don't know" if applicable.

For peer review only - http://bmjopen.bmj.com/site/about/guidelines.xhtml

Site ___ ___ ___ Subarea ___ ___ ___ House number ___ ___ ___ ___ Interviewer ID ___ ___

Sign/Symptom	Healthcare options									
	Study-HCF		Other HCF		Physician	Pharmacy	Traditional healer	Nowhere Self-treatment	Nowhere Nothing	Don't know
	Rate	Code	Rate	Code	Rate	Rate	Rate	Rate	Rate	Tick
**1** [**A**] Fever, any	○=1<br>○=2<br>○=3<br>○=4		○=1<br>○=2<br>○=3<br>○=4		○=1<br>○=2<br>○=3<br>○=4	○=1<br>○=2<br>○=3<br>○=4	○=1<br>○=2<br>○=3<br>○=4	○=1<br>○=2<br>○=3<br>○=4	○=1<br>○=2<br>○=3<br>○=4	○
**2** Fever <3days (continuous)	○=1<br>○=2<br>○=3<br>○=4		○=1<br>○=2<br>○=3<br>○=4		○=1<br>○=2<br>○=3<br>○=4	○=1<br>○=2<br>○=3<br>○=4	○=1<br>○=2<br>○=3<br>○=4	○=1<br>○=2<br>○=3<br>○=4	○=1<br>○=2<br>○=3<br>○=4	○
**3** Fever ≥3days (continuous)	○=1<br>○=2<br>○=3<br>○=4		○=1<br>○=2<br>○=3<br>○=4		○=1<br>○=2<br>○=3<br>○=4	○=1<br>○=2<br>○=3<br>○=4	○=1<br>○=2<br>○=3<br>○=4	○=1<br>○=2<br>○=3<br>○=4	○=1<br>○=2<br>○=3<br>○=4	○
**4** Chills Shivering	○=1<br>○=2<br>○=3<br>○=4		○=1<br>○=2<br>○=3<br>○=4		○=1<br>○=2<br>○=3<br>○=4	○=1<br>○=2<br>○=3<br>○=4	○=1<br>○=2<br>○=3<br>○=4	○=1<br>○=2<br>○=3<br>○=4	○=1<br>○=2<br>○=3<br>○=4	○
**5** Convulsion	○=1<br>○=2<br>○=3<br>○=4		○=1<br>○=2<br>○=3<br>○=4		○=1<br>○=2<br>○=3<br>○=4	○=1<br>○=2<br>○=3<br>○=4	○=1<br>○=2<br>○=3<br>○=4	○=1<br>○=2<br>○=3<br>○=4	○=1<br>○=2<br>○=3<br>○=4	○
**6** Weight loss	○=1<br>○=2<br>○=3<br>○=4		○=1<br>○=2<br>○=3<br>○=4		○=1<br>○=2<br>○=3<br>○=4	○=1<br>○=2<br>○=3<br>○=4	○=1<br>○=2<br>○=3<br>○=4	○=1<br>○=2<br>○=3<br>○=4	○=1<br>○=2<br>○=3<br>○=4	○
**7** Dehydration	○=1<br>○=2<br>○=3<br>○=4		○=1<br>○=2<br>○=3<br>○=4		○=1<br>○=2<br>○=3<br>○=4	○=1<br>○=2<br>○=3<br>○=4	○=1<br>○=2<br>○=3<br>○=4	○=1<br>○=2<br>○=3<br>○=4	○=1<br>○=2<br>○=3<br>○=4	○
**8** Malaise Weakness/ Fatigue	○=1<br>○=2<br>○=3<br>○=4		○=1<br>○=2<br>○=3<br>○=4		○=1<br>○=2<br>○=3<br>○=4	○=1<br>○=2<br>○=3<br>○=4	○=1<br>○=2<br>○=3<br>○=4	○=1<br>○=2<br>○=3<br>○=4	○=1<br>○=2<br>○=3<br>○=4	○
**9** Aches/ Pain (muscle/joint/bone)	○=1<br>○=2<br>○=3<br>○=4		○=1<br>○=2<br>○=3<br>○=4		○=1<br>○=2<br>○=3<br>○=4	○=1<br>○=2<br>○=3<br>○=4	○=1<br>○=2<br>○=3<br>○=4	○=1<br>○=2<br>○=3<br>○=4	○=1<br>○=2<br>○=3<br>○=4	○
**10** Headache	○=1<br>○=2<br>○=3<br>○=4		○=1<br>○=2<br>○=3<br>○=4		○=1<br>○=2<br>○=3<br>○=4	○=1<br>○=2<br>○=3<br>○=4	○=1<br>○=2<br>○=3<br>○=4	○=1<br>○=2<br>○=3<br>○=4	○=1<br>○=2<br>○=3<br>○=4	○
**11** Dizziness/ Confusion Unconsciousness	○=1<br>○=2<br>○=3<br>○=4		○=1<br>○=2<br>○=3<br>○=4		○=1<br>○=2<br>○=3<br>○=4	○=1<br>○=2<br>○=3<br>○=4	○=1<br>○=2<br>○=3<br>○=4	○=1<br>○=2<br>○=3<br>○=4	○=1<br>○=2<br>○=3<br>○=4	○
**12** Blood pressure disorders	○=1<br>○=2<br>○=3<br>○=4		○=1<br>○=2<br>○=3<br>○=4		○=1<br>○=2<br>○=3<br>○=4	○=1<br>○=2<br>○=3<br>○=4	○=1<br>○=2<br>○=3<br>○=4	○=1<br>○=2<br>○=3<br>○=4	○=1<br>○=2<br>○=3<br>○=4	○

For peer review only

Site ___ ___ ___ Subarea ___ ___ ___ House number ___ ___ ___ ___ Interviewer ID ___ ___

Sign/Symptom	Healthcare options									
	Study-HCF		Other HCF		Physician	Pharmacy	Traditional healer	Nowhere Self-treatment	Nowhere Nothing	Don't know
	Rate	Code	Rate	Code	Rate	Rate	Rate	Rate	Rate	Code
**13** Heart disorders	O=1<br>O=2<br>O=3<br>O=4		O=1<br>O=2<br>O=3<br>O=4		O=1<br>O=2<br>O=3<br>O=4	O=1<br>O=2<br>O=3<br>O=4	O=1<br>O=2<br>O=3<br>O=4	O=1<br>O=2<br>O=3<br>O=4	O=1<br>O=2<br>O=3<br>O=4	O
**14** Nausea Vomitting	O=1<br>O=2<br>O=3<br>O=4		O=1<br>O=2<br>O=3<br>O=4		O=1<br>O=2<br>O=3<br>O=4	O=1<br>O=2<br>O=3<br>O=4	O=1<br>O=2<br>O=3<br>O=4	O=1<br>O=2<br>O=3<br>O=4	O=1<br>O=2<br>O=3<br>O=4	O
**15** Diarrhea (watery/ bloody/ mucopurulent)	O=1<br>O=2<br>O=3<br>O=4		O=1<br>O=2<br>O=3<br>O=4		O=1<br>O=2<br>O=3<br>O=4	O=1<br>O=2<br>O=3<br>O=4	O=1<br>O=2<br>O=3<br>O=4	O=1<br>O=2<br>O=3<br>O=4	O=1<br>O=2<br>O=3<br>O=4	O
**16** Abdominal pain Intestinal pain	O=1<br>O=2<br>O=3<br>O=4		O=1<br>O=2<br>O=3<br>O=4		O=1<br>O=2<br>O=3<br>O=4	O=1<br>O=2<br>O=3<br>O=4	O=1<br>O=2<br>O=3<br>O=4	O=1<br>O=2<br>O=3<br>O=4	O=1<br>O=2<br>O=3<br>O=4	O
**17** Blood loss/ Bleeding (internal)	O=1<br>O=2<br>O=3<br>O=4		O=1<br>O=2<br>O=3<br>O=4		O=1<br>O=2<br>O=3<br>O=4	O=1<br>O=2<br>O=3<br>O=4	O=1<br>O=2<br>O=3<br>O=4	O=1<br>O=2<br>O=3<br>O=4	O=1<br>O=2<br>O=3<br>O=4	O
**18** Blood loss/ Bleeding (external)	O=1<br>O=2<br>O=3<br>O=4		O=1<br>O=2<br>O=3<br>O=4		O=1<br>O=2<br>O=3<br>O=4	O=1<br>O=2<br>O=3<br>O=4	O=1<br>O=2<br>O=3<br>O=4	O=1<br>O=2<br>O=3<br>O=4	O=1<br>O=2<br>O=3<br>O=4	O
**19** Sneezing Runny nose	O=1<br>O=2<br>O=3<br>O=4		O=1<br>O=2<br>O=3<br>O=4		O=1<br>O=2<br>O=3<br>O=4	O=1<br>O=2<br>O=3<br>O=4	O=1<br>O=2<br>O=3<br>O=4	O=1<br>O=2<br>O=3<br>O=4	O=1<br>O=2<br>O=3<br>O=4	O
**20** Cough	O=1<br>O=2<br>O=3<br>O=4		O=1<br>O=2<br>O=3<br>O=4		O=1<br>O=2<br>O=3<br>O=4	O=1<br>O=2<br>O=3<br>O=4	O=1<br>O=2<br>O=3<br>O=4	O=1<br>O=2<br>O=3<br>O=4	O=1<br>O=2<br>O=3<br>O=4	O
**21** Rapid breathing/ shortness of breath	O=1<br>O=2<br>O=3<br>O=4		O=1<br>O=2<br>O=3<br>O=4		O=1<br>O=2<br>O=3<br>O=4	O=1<br>O=2<br>O=3<br>O=4	O=1<br>O=2<br>O=3<br>O=4	O=1<br>O=2<br>O=3<br>O=4	O=1<br>O=2<br>O=3<br>O=4	O
**22** Severe breathing/ Difficulties in breathing	O=1<br>O=2<br>O=3<br>O=4		O=1<br>O=2<br>O=3<br>O=4		O=1<br>O=2<br>O=3<br>O=4	O=1<br>O=2<br>O=3<br>O=4	O=1<br>O=2<br>O=3<br>O=4	O=1<br>O=2<br>O=3<br>O=4	O=1<br>O=2<br>O=3<br>O=4	O
**23** Skin inflammation (rashes/ redness/ pus/ itching)	O=1<br>O=2<br>O=3<br>O=4		O=1<br>O=2<br>O=3<br>O=4		O=1<br>O=2<br>O=3<br>O=4	O=1<br>O=2<br>O=3<br>O=4	O=1<br>O=2<br>O=3<br>O=4	O=1<br>O=2<br>O=3<br>O=4	O=1<br>O=2<br>O=3<br>O=4	O
**24** Swelling/ Edema	O=1<br>O=2<br>O=3<br>O=4		O=1<br>O=2<br>O=3<br>O=4		O=1<br>O=2<br>O=3<br>O=4	O=1<br>O=2<br>O=3<br>O=4	O=1<br>O=2<br>O=3<br>O=4	O=1<br>O=2<br>O=3<br>O=4	O=1<br>O=2<br>O=3<br>O=4	O
**25** Jaundice	O=1<br>O=2<br>O=3<br>O=4		O=1<br>O=2<br>O=3<br>O=4		O=1<br>O=2<br>O=3<br>O=4	O=1<br>O=2<br>O=3<br>O=4	O=1<br>O=2<br>O=3<br>O=4	O=1<br>O=2<br>O=3<br>O=4	O=1<br>O=2<br>O=3<br>O=4	O

For peer review only

For peer review only - http://bmjopen.bmj.com/site/about/guidelines.xhtml

Site ___ ___ ___ Subarea ___ ___ ___ House number ___ ___ ___ ___ Interviewer ID ___ ___

FORM 4: ASSESSMENT OF OVERALL, AGE- AND SEX-STRATIFED GENERIC HEALTHCARE UTILIZATION

Part B – Healthcare-associated factors/Vaccination/Disease perception

TRAVEL HEALTHCARE FACILITY

1 In case a healthcare facility (HCF) was rated the 1st choice of care in question 4A (fever), what is the main mode of transportation, travel time (depending on mode of transportation), travel cost (depending on mode of transportation) and travel distance (depending on mode of transportation) that is usually taken to get there?

Instructions: Enter one code per category and sex as well as age group only. Skip this question if there is no female/male member <2years/≥2 to <5years/≥5 to <15years/≥15years of age in this household (see also question 2 and 3 of this form PartA). Skip this question if no HCF was rated the 1st choice of care in question 4A for female/male member <2years/≥2 to <5years/≥5 to <15years/≥15years of age. Record the travel time as minute(s) [min]/ hour(s) [hr], the total costs in USD, including potential gifts, favours and tips, and the total distance as meter(s) [m]/kilometre(s) [km].

Female				Male			
<2 years	≥2 to <5 years	≥5 to <15 years	≥15 years	<2 years	≥2 to <5 years	≥5 to <15 years	≥15 years
**1.1 Transportation mode**							
Code ___	Code ____	Code ____	Code ___	Code ___	Code ____	Code ____	Code ___
[1] Private transportation (i.e. motorbike, car, bus)<br>[2] Public transportation (i.e. motorbike, car, bus)				[3] Walking<br>[4] Bicycle	[5] Other, specify _________<br>[99] Don't know		[98] No response
**1.2 Travel time**							
Code ___	Code ____	Code ____	Code ___	Code ___	Code ____	Code ____	Code ___
[1] <30 mins<br>[2] ≥30 to <60 mins		[3] ≥1.0 to <2.0 hrs<br>[4] ≥2.0 to <3.0 hrs		[5] ≥3.0 to <4.0 hrs<br>[6] ≥4.0 hrs		[99] Don't know<br>[98] No response	
**1.3 Travel cost**							
Code ___	Code ____	Code ____	Code ___	Code ___	Code ____	Code ____	Code ___
[1] <1.0 USD<br>[2] ≥1.0 to <3.0 USD		[3] ≥3.0 to <5.0 USD<br>[4] ≥5.0 to <10.0 USD		[5] ≥10.0 USD		[99] Don't know<br>[98] No response	
**1.4 Travel distance**							
Code ___	Code ____	Code ____	Code ___	Code ___	Code ____	Code ____	Code ___
[1] <500 m<br>[2] ≥500 m to <1.0 km		[3] ≥1.0 to <2.0 km<br>[4] ≥2.0 to <3.0 km		[5] ≥3.0 to <4.0 km<br>[6] ≥4.0 km		[99] Don't know<br>[98] No response	

INSURANCE

2 For which members of this household does the insurance cover health expenses?

Instructions: Enter one code per category. Skip this question if there is no member <2years/≥2 to <5years/≥5 to <15years/≥15years of age in this household (see question 2 and 3 of this form PartA).

<2 years	≥2 to <5 years	≥5 to <15 years	≥15 years
Code _____________	Code _____________	Code _____________	Code _____________
[1] Yes	[2] No<br>[99] Don't know		[98] No response

For peer review only - http://bmjopen.bmj.com/site/about/guidelines.xhtml

Site ___ ___ ___ Subarea ___ ___ ___ House number ___ ___ ___ ___ Interviewer ID ___ ___

3 What kind of health expenses are usually covered by the insurance for members of this household?

Instructions: Enter one code by category and age group only. Skip this question if there is no member <2years/≥2 to <5years/≥5 to <15years/≥15years of age in this household (see also question 2 and 3 of this form PartA). Skip this question if there is no insurance that covers health expenses for <2years/≥2 to <5years/≥5 to <15years/≥15years of age.

	<2 years	≥2 to <5years	≥5 to <15years	≥15years
	[1] Yes	[2] No	[99] Don't know	[98] No response
**1** Registration	Code ______	Code _______	Code _______	Code _____
**2** Consultation/ Doctor's visit	Code ______	Code _______	Code _______	Code _____
**3** Physical examination - general	Code ______	Code _______	Code _______	Code _____
**4** Physical examination – specific, specify_______	Code ______	Code _______	Code _______	Code _____
**5** Diagnostics - general laboratory diagnostics	Code ______	Code _______	Code _______	Code _____
**6** Diagnostics - specific laboratory diagnostics, specify__________	Code ______	Code _______	Code _______	Code _____
**7** Diagnostics - general imaging	Code ______	Code _______	Code _______	Code _____
**8** Diagnostics - specific imaging, specify__________	Code ______	Code _______	Code _______	Code _____
**9** Diagnostics - other, specify________	Code ______	Code _______	Code _______	Code _____
**10** Treatment - general medication	Code ______	Code _______	Code _______	Code _____
**11** Treatment - specific medication, specify________	Code ______	Code _______	Code _______	Code _____
**12** Treatment - other medication, specify__________	Code ______	Code _______	Code _______	Code _____
**13** Treatment - interventions (other than surgery), specify_______	Code ______	Code _______	Code _______	Code _____
**14** Treatment Surgery	Code ______	Code _______	Code _______	Code _____
**15** Hospitalization <7days	Code ______	Code _______	Code _______	Code _____
**16** Hospitalization ≥7days	Code ______	Code _______	Code _______	Code _____
**17** Emergency services	Code ______	Code _______	Code _______	Code _____
**18** Antenatal care	Code ______	Code _______	Code _______	Code _____
**19** Postnatal care	Code ______	Code _______	Code _______	Code _____
**20** Preventive medicine	Code ______	Code _______	Code _______	Code _____
**21** Vaccination	Code ______	Code _______	Code _______	Code _____
**22** Home healthcare (i.e. elderly, disabled, chronic/long-term diseased)	Code ______	Code _______	Code _______	Code _____
**23** Supportive therapy (i.e. physiotherapy, ergotherapy, rehabilitation)	Code ______	Code _______	Code _______	Code _____

For peer review only

For peer review only - http://bmjopen.bmj.com/site/about/guidelines.xhtml

Site ___ ___ ___ Subarea ___ ___ ___ House number ___ ___ ___ ___ Interviewer ID ___ ___

ETHNOGRAPHY

4 To which religion/ethnic group/tribe belong members of this household?

Instructions: Give one answer by age group only. Skip this question if there is no member >2years/≥2 to <5years/≥5 to <15years/≥15years of age in this household (see also question 2 and 3 of this form PartA).

	<2 years	≥2 to <5years	≥5 to <15years	≥15years
Religion/Ethnic group/Tribe	___________	___________	___________	___________
	O 99=Don't know<br>O 98=No response	O 99=Don't know<br>O 98=No response	O 99=Don't know<br>O 98=No response	O 99=Don't know<br>O 98=No response

5 Does the religion/ethnic group/tribe as stated in question 4 influence the various aspects of utilization of healthcare for members of this household?

Instructions: Enter one code by category and age group only. Skip this question if there is member >2years/≥2 to <5years/≥5 to <15years/≥15years of age in this household (see also question 2 and 3 of this form PartA).

	<2 years	≥2 to <5years	≥5 to <15years	≥15years
	[1] Yes	[2] No	[99] Don't know	[98] No response
**1** Visiting a healthcare facility (i.e. hospital, health care center, health post)	Code _____	Code _______	Code _______	Code _____
**2** Visiting a physician	Code _____	Code _______	Code _______	Code _____
**3** Visiting a pharmacy	Code _____	Code _______	Code _______	Code _____
**4** Visiting a traditional healer	Code _____	Code _______	Code _______	Code _____
**5** Deciding for self-treatment	Code _____	Code _______	Code _______	Code _____
**6** Deciding for non-treatment	Code _____	Code _______	Code _______	Code _____
**7** Physical examination – general	Code _____	Code _______	Code _______	Code _____
**8** Physical examination – specific, specify________	Code _____	Code _______	Code _______	Code _____
**9** Collection of specimen (i.e. blood, urine, stool, smear, swab, cerebrospinal fluid)	Code _____	Code _______	Code _______	Code _____
**10** Diagnostics – general laboratory diagnostics	Code _____	Code _______	Code _______	Code _____
**11** Diagnostics – specific laboratory diagnostics, specify___________	Code _____	Code _______	Code _______	Code _____
**12** Diagnostics - general imaging	Code _____	Code _______	Code _______	Code _____
**13** Diagnostics - specific imaging, specify_________	Code _____	Code _______	Code _______	Code _____
**14** Diagnostics - other, specify_________	Code _____	Code _______	Code _______	Code _____
**15** Treatment - general medication	Code _____	Code _______	Code _______	Code _____
**16** Treatment - specific medication, specify________	Code _____	Code _______	Code _______	Code _____
**17** Treatment - other medication, specify___________	Code _____	Code _______	Code _______	Code _____

For peer review only - http://bmjopen.bmj.com/site/about/guidelines.xhtml

Site ___ ___ ___ Subarea ___ ___ ___ House number ___ ___ ___ ___ Interviewer ID ___ ___

**18** Treatment - interventions (other than surgery), specify________________	Code _____	Code _______	Code _______	Code _____
**19** Treatment - Surgery	Code _____	Code _______	Code _______	Code _____
**20** Hospitalization <7days	Code _____	Code _______	Code _______	Code _____
**21** Hospitalization ≥7days	Code _____	Code _______	Code _______	Code _____
**22** Emergency services	Code _____	Code _______	Code _______	Code _____
**23** Antenatal care	Code _____	Code _______	Code _______	Code _____
**24** Postnatal care	Code _____	Code _______	Code _______	Code _____
**25** Preventive medicine	Code _____	Code _______	Code _______	Code _____
**26** Vaccination	Code _____	Code _______	Code _______	Code _____
**27** Home healthcare (i.e. elderly, disabled, chronic/long-term diseased)	Code _____	Code _______	Code _______	Code _____
**28** Supportive therapy (i.e. physiotherapy, ergotherapy, rehabilitation)	Code _____	Code _______	Code _______	Code _____

VACCINATION

6 How were children of this household fed during the first six months of life?

Instructions: Skip this question if there are no male/female children in this household (see also question 2 and 3 of this form PartA).

- O 1=Breastfed exclusively
- O 2=Breastfed and other feeds, specify ______
- O 3=Formula and other feeds, specify ________
- O 4=Breastfed and formula
- O 5=Formula exclusively
- O 6=Other, specify ________________
- O 99=Don't know
- O 98=No response

7 Which water source was usually used to prepare formula or formula and other feeds as stated in question 6 for children of this household?

Instructions: Skip this question if there are no children in this household (see also question 2 and 3 of this form PartA).

- O 1=Tap water (inside the house; private)
- O 2=Tap water (outside the house i.e. back-/courtyard; private)
- O 3=Tap water (outside the house; public, shared)
- O 4=Standpipe/Piped water (inside the house; private)
- O 5=Standpipe/Piped water (outside the house i.e. back-/courtyard; private)
- O 6=Standpipe/Piped water (outside the house; public, shared)
- O 7=Water truck (public; shared)
- O 8=Bottled water
- O 9=Surface water (i.e. lake, pond, river, stream)
- O 10=Surface water (i.e. canal, irrigation canal)
- O 11=Spring water
- O 12=Rain water
- O 13=Well/Borehole (open/uncovered/unprotected)
- O 14=Well/Borehole (covered/protected)
- O 15=NO FORMULA/OTHER FEEDS PREPARED
- O 16=Other, specify _____________
- O 99=Don't know
- O 98=No response

8 Did children of this household receive any vaccines since birth, including the birth vaccines?

Instructions: Enter one code by category and child only. Skip this question if there are no children in this household (see also question 2 and 3 of this form PartA). Refer to question 9 of "Form 3 Part A – Demographic data/General household information" and record the initials/identifier of the member(s).

Child 1	Child 2	Child 3	Child 4
Initials/Identifier: _____	Initials/Identifier: _____	Initials/Identifier: _____	Initials/Identifier: _____

For peer review only - http://bmjopen.bmj.com/site/about/guidelines.xhtml

Site ___ ___ ___ Subarea ___ ___ ___ House number ___ ___ ___ ___ Interviewer ID ___ ___

[1] YES, vaccinated	[5] No, lack of vaccine	[9] No, HCF busy
[2] No, caretaker busy	[6] No, child too old/young	[10] No, child sick
[3] No, caretaker decided no need	[7] No, caretaker refused/too worried	[11] No, no vaccination card
[4] No, unknown where to go	[8] No, other, specify ___________	[99] Don't know [98] No response

**8.1 Diphtheria and Tetanus and Pertussis and *Haemophilus influenza* and Hepatitis B vaccine/DTwPHibHepB** (e.g. 6/8, 10/12, 14/16 weeks)			
Code __________	Code __________	Code __________	Code __________
**8.2 Tetanus toxoid (TT)** (e.g. 1st contact pregnancy, 1, 6, 12/24 months)			
Code __________	Code __________	Code __________	Code __________
**8.3 Tetanus and diphtheria toxoid (TD)** (e.g. 1st contact pregnancy, 1, 6, 12 months)			
Code __________	Code __________	Code __________	Code __________
**8.4 Tuberculosis/BCG** (at birth)			
Code __________	Code __________	Code __________	Code __________
**8.5 Rotavirus/RV** (e.g. 6/8, 10/12, 16 weeks)			
Code __________	Code __________	Code __________	Code __________
**8.6 Measles/Measles and Rubella (MR)** (e.g. 9, 15/18months)			
Code __________	Code __________	Code __________	Code __________
**8.7 Polio/OPV** (e.g. birth, 6/8, 10/12, 14/16 weeks)			
Code __________	Code __________	Code __________	Code __________
**8.8 Pneumococcus/PCV** (e.g. 6/8, 10/12, 14/16 weeks)			
Code __________	Code __________	Code __________	Code __________
**8.9 Yellow fever (YF)** (e.g. 9 months)			
Code __________	Code __________	Code __________	Code __________
**8.10 Other Vaccines**			
Specify, __________	Specify, __________	Specify, __________	Specify, __________
Specify, __________	Specify, __________	Specify, __________	Specify, __________
Specify, __________	Specify, __________	Specify, __________	Specify, __________
Specify, __________	Specify, __________	Specify, __________	Specify, __________

For peer review only - http://bmjopen.bmj.com/site/about/guidelines.xhtml

Site ___ ___ ___ Subarea ___ ___ ___ House number ___ ___ ___ ___ Interviewer ID ___ ___

DISEASE PERCEPTION

9 Knowledge, perception, causes and prevention of common infectious diseases

**Disease**	**9.1 Disease known by household members?**<br>[1] Yes<br>[2] No<br>[99] Don't know<br>[98] No response	**9.2 Perception of disease by household members?**<br>*Instruction:* Describe briefly if known.	**9.3 Causes for disease known by household members?**<br>*Instructions:* Enter all codes (list A) that apply; more than one answer is possible.	**9.4 Measures to prevent disease known by household members?**<br>*Instructions:* Enter all codes (list B) that apply; more than one answer is possible.	**9.5 Measures to prevent disease utilized by household members?**<br>*Instructions:* Enter all codes (list B) that apply; more than one answer is possible.
**A.** Typhoid fever	Code ___________		Code ___________	Code ___________	Code ___________
**B.** Cholera	Code ___________		Code ___________	Code ___________	Code ___________
**C.** Malaria	Code ___________		Code ___________	Code ___________	Code ___________
**D.** Influenza	Code ___________		Code ___________	Code ___________	Code ___________
**E.** Meningitis	Code ___________		Code ___________	Code ___________	Code ___________
**F.** Hepatitis E	Code ___________		Code ___________	Code ___________	Code ___________
**G.** Tuberculosis	Code ___________		Code ___________	Code ___________	Code ___________
**H.** HIV/AIDS	Code ___________		Code ___________	Code ___________	Code ___________

List A: Possible causes	List B: Possible preventive measures
[1] Alcohol consumption<br>[2] Weather/witchcraft<br>[3] Mosquito/insect bite<br>[4] Dog bite	[1] No alcohol consumption<br>[2] Sleeping under bed net<br>[3] Using mosquito/insect spray<br>[4] Maintain a clean house and immediate surroundings of the house

For peer review only

Site ___ ___ ___ Subarea ___ ___ ___ House number ___ ___ ___ ___ Interviewer ID ___ ___

[5] Drinking untreated surface/spring water (i.e. lake, pond, river, stream, canal/irrigation canal)<br>[6] Close body contact/kissing/hugging<br>[7] Eating raw food items such as vegetables/lettuce, fruits, dairy products, eggs, meat or fish<br>[8] Not cleaning hands properly when preparing food/meals<br>[9] Not cleaning hands properly before/after visiting a toilet<br>[10] Contact to human blood (i.e. injection, transfusion, surgery)<br>[11] Consuming raw animal blood<br>[12] Other, specify _______________<br>[99] Don't know<br>[98] No response	[5] Practice proper disposal of human and domestic waste<br>[6] Avoiding close contact to dogs<br>[7] Treating water (i.e. boiling, filtration, solar disinfection, sedimentation, chlorination)<br>[8] Not cooking/preparing food/meals with untreated water<br>[9] Not washing food items to be consumed raw with untreated water<br>[10] Re-boiling/re-heating left-over/prepared food/meals prior to consumption<br>[11] Not eating raw food items such as vegetables/lettuce, fruits, dairy products, eggs, meat or fish<br>[12] Cover food items/meals<br>[13] Protect food items/meals from flies<br>[14] Avoid food/beverage from street vendors<br>[15] Avoid close contact to sick people<br>[16] No close body contact/kissing/hugging<br>[17] Covering nose/mouth while sneezing/coughing<br>[18] No contact/consumption of human/animal blood<br>[19] Practicing proper hand hygiene when preparing food/meals (i.e. washing hands with soap and water, rub hands clean)<br>[20] Practicing proper hand hygiene before/after visiting a toilet (i.e. washing hands with soap and water, rub hands clean)<br>[21] Other, specify ____________________<br>[99] Don't know<br>[98] No response

For peer review only - http://bmjopen.bmj.com/site/about/guidelines.xhtml

Site ___ ___ ___ Subarea ___ ___ ___ House number ___ ___ ___ ___ Interviewer ID ___ ___

10 For which members of this household are the preventive measures as listed in question 9.5 against typhoid fever used?

Instructions: Tick all that applies; more than one answer is possible.

O 1=<2years O 3=≥5 to <15years O 5=NOT USED O 99=Don't know
O 2=≥2 to < 5years O 4=≥15years O 98=No response

11 Name the household members that were diagnosed with typhoid fever during the past 3 months. Which diagnostics were used to confirm typhoid fever?

Instructions: Enter all codes that apply for each household member; more than one answer is possible.

Member 1 Initials/Identifier: ______	Member 2 Initials/Identifier: ______	Member 3 Initials/Identifier: ______	Member 4 Initials/Identifier: ______
[1] Blood culture<br>[2] Stool culture<br>[3] Blood/Widal test<br>[4] Other test, specify______________<br>[5] Not applicable (not diagnosed with typhoid fever)<br>[99] Don't know<br>[98] No response			
Code ___________	Code ___________	Code ___________	Code ___________

12 (Where were members of this household recorded in question 11 diagnosed for typhoid fever?) Where did members of this household seek care?

Instructions: Enter one code by household member only.

Member 1 Initials/Identifier: ______	Member 2 Initials/Identifier: ______	Member 3 Initials/Identifier: ______	Member 4 Initials/Identifier: ______
[1] Healthcare facility, specify ____________<br>[2] Physician<br>[3] Nowhere, self-treatment<br>[4] Traditional healer<br>[5] Pharmacy<br>[6] Nowhere, did not seek care<br>[7] Other, specify ____________<br>[8] Not applicable<br>[99] Don't know<br>[98] No response			
Code ___________	Code ___________	Code ___________	Code ___________

13 (Did members of this household that were diagnosed with typhoid fever as recorded in question 11 get any treatment?) Which treatment did they get?

Instructions: Tick one answer by household member only.

Member 1 Initials/Identifier: ______	Member 2 Initials/Identifier: ______	Member 3 Initials/Identifier: ______	Member 4 Initials/Identifier: ______
O 1=Treatment, specify<br>_______________<br>O 2=Not applicable<br>O 99=Don't know<br>O 98=No response	O 1=Treatment, specify<br>_______________<br>O 2=Not applicable<br>O 99=Don't know<br>O 98=No response	O 1=Treatment, specify<br>_______________<br>O 2=Not applicable<br>O 99=Don't know<br>O 98=No response	O 1=Treatment, specify<br>_______________<br>O 2=Not applicable<br>O 99=Don't know<br>O 98=No response

For peer review only

Site ___ ___ ___ Subarea ___ ___ ___ House number ___ ___ ___ ___ Interviewer ID ___ ___

FORM 5: ASSESSMENT OF OVERALL, AGE-AND SEX-STRATIFIED ACTUAL HEALTHCARE UTILIZATION

HEALTHCARE SEEKING BEHAVIOR

1 Record below any occurrence of a sign(s)/ a symptom(s) (see List 1) and specify where (see List 2) and how often a member/members sought healthcare during the past 3 months?

Instructions: See "Form 3 - Part A - GENERAL HOUSEHOLD INFORMATION" to obtain the initials/identifier of each household member. Indicate one sign/symptom per line only. Start a new line if different healthcare options were selected for recurring signs/symptoms. Indicate the number of occurrences for each sign/symptom during the past 3 months. If a different healthcare option was taken for a recurring sign/symptom, indicate this on a new line. Please complete an additional form if >20 occurrences took place during the past 3 months.

**List 1: Sign/Symptom**		**List 2: Healthcare option**
[A] Fever, any<br>[B] Fever <3days (continuous)<br>[C] Fever ≥3days (continuous)<br>[D] Chills/ Shivering<br>[E] Convulsion<br>[F] Weight loss<br>[G] Dehydration<br>[H] Malaise/ Weakness/ Fatigue<br>[I] Aches/Pain (muscle/joint/bone)<br>[J] Headache<br>[K] Dizziness/Confusion/ Unconsciousness<br>[L] Blood pressure disorders<br>[M] Heart disorders	[N] Nausea/ Vomiting<br>[O] Diarrhea (watery/bloody/mucopurulent)<br>[P] Abdominal pain/ Intestinal pain<br>[Q] Blood loss/ Bleeding (internal)<br>[R] Blood loss/ Bleeding (external)<br>[S] Sneezing/ Runny nose<br>[T] Cough<br>[U] Rapid breathing/ Shortness of breath<br>[V] Severe breathing/ Difficulties in breathing<br>[W] Skin inflammation (rashes/redness/ pus/itching)<br>[X] Swelling/ Edema<br>[Y] Jaundice	[1] Study-HCF, Code __________<br>[2] Other HCF, Code __________<br>[3] Physician<br>[4] Pharmacy<br>[5] Traditional healer<br>[6] Nowhere/Self-treatment<br>[7] Nowhere/Nothing

**No.**	**Household member** Record the initials/identifier of a household member; see "Form 3 - Part A - GENERAL HOUSEHOLD INFORMATION"	**Sign/ Symptom** Use List 1 to select a code	**Healthcare option**		**Frequency** Indicate the number of incidences for the sign/symptom listed
			Use List 2 to select a code	HCF code	
1					
2					
3					
4					
5					
6					
7					
8					
9					

For peer review only - http://bmjopen.bmj.com/site/about/guidelines.xhtml

Site ___ ___ ___ Subarea ___ ___ ___ House number ___ ___ ___ ___ Interviewer ID ___ ___

10					
11					
12					
13					
14					
15					
16					
17					
18					
19					
20					

For peer review only

For peer review only - http://bmjopen.bmj.com/site/about/guidelines.xhtml

Appendix 3/Supplementary file 3: Protocol, Definitions/Terminology and Study Forms (French)

Protocol

Le protocole d'étude HPAfrica: Évaluation du comportement de santé et des facteurs démographiques socio-économiques, comportement d'hygiène - une étude standardisée répétée transversale dans plusieurs cohortes en Afrique subsaharienne

Abstrait

Introduction L'objectif de l'étude HPAfrica est de déterminer les comportements sanitaires et facteurs démographiques, y compris les facteurs socio-économiques, ethnographiques aussi bien que d'hygiène et d'assainissement, sur les sites d'étude du programme Fièvre Typhoïde Sévère en Afrique (SETA). L'objectif du programme SETA est d'enquêter sur la surveillance de la fièvre au niveau des établissements de soins de santé au Burkina Faso, en République Démocratique du Congo, en Éthiopie, au Ghana, à Madagascar et au Nigéria. Les estimations précises de la charge de morbidité nécessitent un ajustement des styles de comportements de santé, qui sont supposés varier au sein d'une population d'étude.

Methodes et Analyses Pour la taille d'échantillon minimale d'interviews de ménages requise, les hypothèses d'une population infinie, d'un effet de design et d'une stratification par âge et par sexe sont considérées. En l'absence d'un cadre d'échantillonnage de la population ou d'une liste de ménages, une approche spatiale sera utilisée pour générer des points aléatoires géographiques avec un outil ArcGIS (Aeronautical Reconnaissance Coverage Geographic Information System). Les impressions de l'imagerie satellite de Google Earth Pro® visualisent ces points. Les données d'intérêt seront évaluées au niveau des ménages en différentes saisons en appliquant un échantillonnage stratifié en fonction de la population. Une application basée sur Android et un service Web sera développé pour la capture de données électroniques et la synchronisation avec le serveur de base de données sur Internet en temps réel. Les poids d'échantillonnage seront calculés pour tenir compte des differences possibles dans les probabilitiés de selection. Des analyses de données descriptives seront effectuées afin d'évaluer l'informations de base de chaque population étudée et le comportement santé stratifié par âge et par sexe. Ceci permettra d'ajuster les estimations de la charge de morbidité. En outre, des analyses multivariées seront appliquées pour examiner les associations entre le comportement santé, les facteurs demographiques et le fardeau de la maladie trouvée dans l'étude SETA.

Ethique et diffusion

Les approbations éthique pour ce protocole ont été reçues par l'International Vaccine Institute (IVI, n ° 2016-0003) et par toutes les institutions collaboratrices des pays participants. Il est prévu de diffuser les résultats de cette étude par publication dans un journal révise par des pairs.

Les forces et les limites de cette étude

- La normalisation dans la recherche multi-pays /-site à base communautaire nécessite de la prudence compte tenu des variations de cadre, de langue et de culture.
- Les évaluations de santé publique à grande échelle complèteront les données sur la charge de morbidité en étudiant les sources de transmission et d'infection.
- Les données de population actuelles dans les sites d'étude et leurs sous-zones définies administrativement et les bases de sondage de population sont supposées être limitées.
- La collecte de données électroniques peut entraîner problèmes techniques liés aux dommages, aux pannes fonctionnelles ou à la perte de dispositifs.
- Une connexion Internet sans fil instable peut limiter les équipes de données à mettre à jour l'application basée sur Android, communiquer en temps réel avec les responsables de l'étude et à synchroniser les données collectées avec le serveur.

Inscription: non requise

For peer review only - http://bmjopen.bmj.com/site/about/guidelines.xhtml

Mots clés: comportement sanité/hygiénique, assainissement, socio-économique, cadre d'échantillonnage de la population/l'échantillonnage spatiale, Afrique subsaharienne, HPAfrica étude

Contexte

L'évaluation du comportement de santé, y compris l'utilisation des soins de santé, est un déterminant important pour la production d'estimations précises du fardeau de la maladie parmi les populations cibles. Ceci est particulièrement important pour les études qui utilisent un modèle de surveillance observationnelle basé sur les établissements de santé, car une variation substantielle des comportements de santé influence fortement les estimations de la maladie (1). L'ajustement des calculs de la maladie pour des variations du comportement de santé augmentera l'exactitude de l'évaluation des mesures de la fréquence des maladies causées par des pathogènes bactériens, fongiques, viraux ou parasitaires parmi les populations étudiées. De plus, l'influence d'une grande variété de facteurs basés sur la population sur le comportement de santé et le fardeau de la maladie a été reconnue. Cela inclut des facteurs socioéconomiques tels que l'éducation, la profession, le revenu, la disponibilité des biens des ménages (2, 3), les facteurs ethniques / religieux et culturels (4, 5), mais aussi l'hygiène et l'assainissement (6-10), la manipulation des aliments (11-14) et les contacts avec des animaux (9).

L'étude **H**ealth **P**opulation **Africa** (HPAfrica) sera menée au Burkina Faso, en République démocratique du Congo (RDC), en Éthopie, au Ghana, à Madagascar et au Nigéria. Les expériences du programme Typhoid Fever Surveillance in Africa Program (TSAP) seront reportées sur le programme Severe Typhoid in Africa (SETA) (15, 16). Dans ce programme, une surveillance standardisée de la fièvre, basée sur les établissements de santé, est effectuée sur des sites d'étude sélectionnés dans les pays participants sur une période de deux ans (17). SETA vise principalement à collecter systématiquement des informations sur le fardeau des infections graves à *Salmonella*. Les sujets vivant dans la zone the chalandise définie, avec un épisode de fièvre aiguë, des antécédents de fièvre ≥ 3 jours consécutifs, une fièvre typhoïde cliniquement soupçonnée ou des perforations gastro-intestinales cliniquement diagnostiquées dues à la fièvre typhoïde seront éligibles. En outre, la sévérité des maladies provoquées par *Salmonella* et l'histoire naturelle de l'infection à *Salmonella* seront enregistrées avec des analyses supplémentaires sur les réponses immunitaires de l'hôte et le portage chronique.

Objectifs et Résultats

L'objectif de l'étude HPAfrica est d'évaluer le comportement de santé générique et réel stratifié par âge et par sexe. Le comportement générique se rapporte au comportement associé à la fièvre et à d'autres signes et symptômes sans l'apparition, alors que le comportement réel est lié à l'apparition (Formulaire 4 Partie A, Formulaire 5). Les données sont utilisées pour ajuster les mesures de l'occurrence de la maladie comme l'incidence d'agents pathogènes infectieux stratifiés selon l'âge et le sexe pour la proportion d'une population étudiée non capturée dans le programme de surveillance SETA. Les données documentaires pour divers signes et symptômes ou une combinaison de ceux-ci sont utilisés pour une classification progressive de l'utilisation des soins de santé en fonction de la gravité de la maladie.

L'étude HPAfrica recueillera des données sur les facteurs associés aux soins de santé, tels que les modalités de voyage, la possession d'une assurance maladie, les facteurs culturels/ethnographiques, l'état vaccinal des enfants (18, 19) et la fréquence et la perception individuelle des maladies (Formulaire 4 Partie B). Les données démographiques (Formulaire 3 Partie A) seront utilisées pour la stratification par âge et par sexe de la population étudiée qui est nécessaire pour calculer les facteurs d'ajustement pour les mesures de l'occurrence de la maladie. Les données socio-économiques (Formulaire 3 Partie B) seront utilisées pour calculer les indices de richesse pour les approches descriptives et analytiques. Les informations sur l'hygiène et l'assainissement (Formulaire 3 Partie C) peuvent permettre une meilleure compréhension de l'occurrence et de la fréquence des agents pathogènes identifiés parmi les populations étudiées.

Méthodes

Sites d'étude

For peer review only - http://bmjopen.bmj.com/site/about/guidelines.xhtml

Les limites de chaque site seront définies en utilisant des indications préexistantes qui avaient été déterminées par les autorités statistiques ou ministères de la santé (Tableau 1), couplées à des données géospatiales à haute résolution de source ouverte. L'examen rétrospectif des registres de des deux dernières années aux établissements de soins de santé de recrutement SETA permettra de redéfinir les limites du site. On s'attend à ce que cette revue révèle les résidences ou au moins les zones résidentielles administratives plus larges telles que des communautés, des districts ou des villages de patients qui ont cherché des soins de santé pour une raison quelconque. Des cartes et des images satellites peuvent être utilisées pour mieux visualiser les résidences des patients et les limites générales, y compris les sous-zones définies géographiquement ou administrativement, les secteurs de recensement ou les strates de chaque site. Là où les limites ne peuvent être clairement définies, des facteurs supplémentaires, tels que la distance à un établissement de soins de santé de recrutement, peuvent être pris en compte.

Taille d'échantillons

Toutes les sources disponibles seront utilisées pour recueillir des données sur la population le plus à jour stratifiées selon l'âge et le sexe par site d'étude et par les sous-zones définies administrativement ou géographiquement ou les strates. Les strates seront définies comme la plus petite unité administrative publiée par le recensement d'un pays participant. Les ménages dans les strates devraient être répartis de manière homogène. Les sources de données sur la population pour les dénombrements par strate c~~C~~ela peut inclure les dernières informations démographiques issues d'un recensement d'un total de la population ou d'un DSS (Demographic Surveillance System) / HDSS (Health and Demographic Surveillance System). Les chiffres sommaires sur la population et les facteurs de croissance de la population, s'ils sont disponibles, peuvent constituer une source de données supplémentaire. Pour les sites avec des chiffres de population obsolètes ou indisponibles, des outils d'échantillonnage en accès libre couplés à des sources de données sur la population, comme les données sur la population quadrillée basées sur la densité de WorldPop peuvent également être utilisés (20-25).

Toutes les strates par site d'étude seront incluses. Un ménage tel que défini pour l'étude HPAfrica constitue l'unité d'échantillonnage primaire (PSU). Le nombre minimum de ménages sélectionnés au hasard sera calculé en utilisant l'équation basée de précision (équation-I) en supposant une population infinie et en considérant un effet de design (DEFF) (26-28), y compris les hypothèses qui l'accompagnent:

Equation-I: Calcul de la taille d'échantillon basé sur la précision par site d'étude pour une population infinie (26-32)

$$n_0 = DEFF \cdot \left[{z^2}_{1-\alpha/2} \cdot \frac{4 \cdot p \cdot (1-p)}{d^2} \right]$$

<u>Hypothèses d'Equation-I</u>

n_0	Nombre minimum total de ménages à interroger dans une zone d'étude en supposant une population infinie
$DEFF$	Effect de design; fixé à 1.5<br>≤1.0=negative correlation of the outcome(s) of interest between household members; 1.0=no correlation of the outcome(s) of interest between household members; ≥1.0=positive correlation of the outcome(s) of interest between household members
$z_{1-\frac{\alpha}{2}}$	Déviation Standard correspondant à un intervalle de confiance de 95% (1.96 pour alpha de 0.05)
d	Précision (erreur acceptable); estimation de point; fixé à 0.2
p	Proportion de la population de l'étude devant se rendre dans un établissement de soins de santé de recrutement pour des conditions associées à de la fièvre et d'autres signes et symptômes (proportion capturée); fixé à 0.2
$1-p$	Proportion de la population de l'étude ne devant se rendre dans un établissement de soins de santé de recrutement pour des conditions associées à de la fièvre et d'autres signes et symptômes (proportion non capturée)

For peer review only

For peer review only - http://bmjopen.bmj.com/site/about/guidelines.xhtml

L'effet de design est défini comme un facteur d'ajustement pour le regroupement naturel des comportements de santé comme principal résultat d'intérêt parmi les membres du ménage. Il rend compte d'une plus grande variance statistique et, par conséquent, d'une plus faible précision par rapport à l'échantillonnage aléatoire simple (15, 30, 33, 34). Pour HPAfrica, le DEFF peut être fixé de manière conservatrice à 1,5 sur la base d'une estimation de 1,42 résultant d'une taille moyenne de ménage de sept et d'un c'efficient de corrélation intracluster (ICC) de 0,07 provenant de l'étude TSAP (15, 31, 32, 35). Une proportion p de 0,2 pourra être considérée si aucune autre estimation n'est disponible ou p peut être basé sur des informations plus précises disponibles par site ou sur les expériences au cours de l'étude TSAP (Tableau 2): Madagascar, Isotry : p=0,01, Burkina Faso, Polesgo: p=0,9 (36). Combiner tous les hypothèses dans l'équation-I va avoir pour résultat un nombre global minimum (n_0) de 92 ménages à interroger par site.

En outre, n_0 pourra être pris en compte pour la répartition par âge et par sexe de chaque population de l'étude représentée par q, la proportion minimum supposée de stratification par groupe d'âge (<5 ans, ≥5 à <15 ans, ≥15 ans). Le facteur q pourra être défini de façon arbitraire à 0,2 ou être basé sur les expériences précédentes: Éthopie, Butajira : q =0,09, Burkina Faso, Polesgo : q =0,23 (15). Appliquer $\widehat{q}$ à l'Equation-I pour une distribution binomiale, y compris ses postulats connexes, aura pour résultat le nombre minimum de ménages à interroger ($\overline{n_0}$) de 461 stratifié selon l'âge (Equation-II):

Equation-II: Calcul de la taille d'échantillon basé sur la précision par site d'étude pour une population infinie avec stratification selon l'âge et population-poids par sous-zone ou strate:

$$\overline{n_0} = \left(DEFF \cdot \left[z^2{}_{1-\frac{\alpha}{2}} \cdot \frac{4 \cdot p \cdot (1-p)}{d^2}\right]\right)/q \qquad \overline{n_{0w}} = \overline{n_0} \cdot (n/N)$$

Hypothèses d'Equation-II

$\overline{n_0}$	Nombre total minimum de ménages à interroger dans une zone d'étude en supposant une population infinie et une stratification selon l'âge
$\overline{n_{0w}}$	Minimum number of households to be interviewed by subarea or stratum
n/N	Population size of subarea or stratum divided by the population size of the total study area
$DEFF$	Effect de design; fixé à 1.5
$z_{1-\frac{\alpha}{2}}$	Déviation Standard correspondant à un intervalle de confiance de 95% (1.96 pour alpha de 0.05)
d	Précision (erreur acceptable); estimation de point; fixé à 0.2
p	Proportion de la population de l'étude devant se rendre dans un établissement de soins de santé de recrutement pour des conditions associées à de la fièvre et d'autres signes et symptômes (proportion capturée); fixé à 0.2
$1-p$	Proportion de la population de l'étude ne devant se rendre dans un établissement de soins de santé de recrutement pour des conditions associées à de la fièvre et d'autres signes et symptômes (proportion non capturée)
q	Proportion minimum de stratification par groupe d'âge, définie à 0,2

Les comportements de santé seront évalués deux fois dans les mêmes ménages si possible ou dans des ménages alternatifs, au cas où la population de l'étude serait instable en raison de la migration et / ou de taux de natalité / mortalité élevés. Des ménages supplémentaires, supposés être de 20% en moyenne (Tableau 2), peuvent être ajoutés pour tenir compte de la possible perte de suivi entre les deux évaluations. Eventuellement, l'échantillonnage stratifié pondéré en fonction de la population selon la proportion de la population des strates (n/N) pour tenir compte de la probabilité inégale de sélection due à la répartition hétérogène des populations entre les strates (poids de la population (n/N) par strat) sera appliqué à $\overline{n_0}$de 553 (37, 38).

For peer review only

Echantillonnage avec et sans cadre d'échantillonnage de population

Si une base d'échantillonnage de la population ou une liste de ménages compréhensif et à jour existe par DSS / HDSS ou recensement (39, 40), une sélection aléatoire informatisée des ménages en tant que PSU sera effectuée en utilisant SAS (Système d'analyse statistique, version 9.4, SAS Institute, Cary NC) en appliquant une sélection aléatoire simple en série sans remplacement pondéré selon la proportion de la population des strates (n/N). Les ménages sélectionnés avec des identifiants sont visualisés en utilisant des DDS / HDSS ou des outils de recensement. En cas de refus, d'absence d'un répondant ou de localisation d'un ménage abandonné, les enquêteurs sont formés pour visiter un ménage alternatif le plus proche du la côte droit ou gauche d'un ménage d'origine. Cela suit le principe de la proximité la plus proche comme vu dans la recherche précédente (41-44). Pour limiter, en outre, les probabilités de sélection inégale, donc les probabilités de sélection par les enquêteurs, nous présélectionnons un nombre arbitraire de cinq ménages alternatifs d'échantillonnage de population qui doivent être plus proches du ménage d'original plutôt que de demander par exemple une sélection en base de loterie ou une sélection dans le sens des aiguilles d'une montre (37, 41, 44). En outre, la notation de l'un (=le plus proche) à cinq (=le plus éloigné) est appliquée aux ménages alternatifs et les enquêteurs sont invités à suivre strictement la séquence de notation au cours de la procédure de sélection du ménage. La conformité des enquêteurs avec les règles de sélection des ménages originaux et alternatifs sera vérifiée quotidiennement. Les écarts seront étudiés et une formation complémentaire sera fournie aux enquêteurs au besoin.

S'il n'existe pas de cadre d'échantillonnage de la population, nous appliquerons une technique d'échantillonnage spatial stratifié pondérée par la population de proportion (n/N) (37). Guidés par des recherches antérieures, des points spatiaux aléatoires sont générés à l'aide de l'outil de génération de points aléatoires ArcGIS (Aeronautical Reconnaissance Coverage Geographic Information System, Redlands, CA, USA, version 10.2) (21, 23, 37, 40, 43, 45-51). L'outil place de la manière aléatoire des nombres spécifiés de points dans les entités d'un polygone correspondant à une sous-zone administrative ou à une strate. Les coordonnées X et Y définissent un point jusqu'à ce que la taille d'échantillon calculée par strate soit atteinte. Les points générés par ArcGIS sont convertis en un fichier image au format KML (Keyhole Markup Language) ou KMZ (Keyhole Markup landguage Zipped) et importés dans Google Earth Pro®. Un identifiant numérique unique et des coordonnées géographiques sont affectés à chaque point. L'annexe 4 illustre notre approche en utilisant des exemples du Ghana et de Madagascar. Les points spatiaux étiquetés avec des identifiants sont représentés sur des impressions de la taille d'une affiche de (60 x 60 à 60 x 90 cm) d'images satellite Google Earth Pro avec une haute résolution ou alternativement en utilisant une application de source ouverte pour la navigation et la cartographie automatiques hors ligne / en ligne pour localiser les points sélectionnés sur le terrain. Des récepteurs GPS (Global Positioning System) portables (Garmin-eTrex, Garmin Ltd., Lenexa, KS, USA) aident à vérifier les emplacements. Les récepteurs seront positionnés près d'un point localisé, dans une position statique et en plein air pour assurer une lecture sans obstacle. Les enquêteurs évaluent les points spatiaux de la présence d'un ménage en tant que PSU (24). En cas de refus, d'absence du répondant ou de localisation d'un point non résidentiel, les enquêteurs sont formés pour visiter un point spatial alternatif présélectionné le plus proche de la droite ou de la gauche d'un point original et l'évaluer pour la présence d'un ménage (41-43, 52). Pour limiter les chances de probabilité de sélection inégale, nous présélectionnons au moins cinq points spatiaux alternatifs à proximité immédiate du point d'origine qui représentent une structure de taille appropriée et de forme rectangulaire ou carrée à partir de l'imagerie satellite Google Earth Pro outil de mesure de distance. Commue pour les sites ayant un cadre d'échantillonnage de la population, la notation d'un à cinq est appliquée aux points spatiaux alternatifs et les enquêteurs doivent suivre la séquence de notation. La conformité des enquêteurs avec les règles de sélection des ménages originaux et alternatifs sera vérifiée quotidiennement. Les écarts seront étudiés et une formation complémentaire sera fournie aux enquêteurs au besoin. Dans le cas où un point spatial est placé dans l'équidistance de deux structures (ou plus), l'intervieweur saute le point spatial original et choisit un point spatial alternatif présélectionné suivant la séquence de notation. Dans le cas où deux points spatiaux originaux (ou plus) sont placés sur la même structure, l'intervieweur évalue la structure de la présence d'un ménage et choisit un second point spatial alternatif (ou plus) présélectionné après la séquence de notation.

For peer review only - http://bmjopen.bmj.com/site/about/guidelines.xhtml

Indépendamment de la présence ou de l'absence d'un cadre d'échantillonnage de la population, un ménage unifamilial à un étage sera directement contacté pour participer à l'étude. Cependant, un ménage seulement est inscrit dans une structure multifamiliale à un seul étage selon les procédures suivants : l'enquêteur entre dans la structure et évalue le nombre total de ménage : le premier ménage du côté droit / gauche (selon la construction intérieure) de la structure est approché ; si le premier ménage ne participe pas, le second ménage du côté droit / gauche de la même structure est approché. L'enquêteur continue jusqu'à ce qu'un ménage par structure soit inscrit. De même, un ménage seulement est inscrit dans une structure multifamiliale à plusieurs étages. Après avoir évalué le nombre total de ménage présents, l'enquêteur se rapproche du premier ménage ne participe pas, le deuxième ménage le plus proche de l'entrée sur le même étage / suivant (en fonction de la construction intérieure) est approché ; l'enquêteur continue jusqu'à ce qu'un ménage par structure soit inscrit. L'enquêteur choisit un point spatial alternatif présélectionné après la séquence de notation au cas où aucun ménage ne peut être inscrit.

Fréquence de la collecte de données

Les points résidentiels seront visités deux fois pour des entrevues au cours du programme SETA pour prendre en compte un possible impact lié à la saison, sur le comportement sanitaire (53-57). Les enquêtes auront lieu à différentes saisons - une à la fin de la saison sèche ou au moins un mois après son début et une vers la fin de la saison des pluies ou au moins un mois après son début (Tableau 3). Actuellement, l'étude HPAfrica est en cours au Burkina Faso, au Ghana et à Madagascar et devrait se poursuivre dans les autres pays au cours des prochains mois.

Critères d'inclusion et d'exclusion

Les membres du ménage de tout âge et des deux sexes qui vivent dans la zone d'étude au jour de l'interview seront éligibles pour l'inclusion. Un ménage sera exclu si le répondant désigne refuse de participer ou n'est pas disponible après trois visites consécutives. Tous les visiteurs et individus dont le lieu de résidence n'est pas connu ou est en-dehors de la zone d'étude seront exclus.

Pour raison de cette enquête, un ménage est défini comme une personne ou un groupe de personnes apparentées ou non apparentées vivant dans la même unité d'habitation, reconnaissant un individu adulte comme chef du ménage, partageant les mêmes installations domestiques, et subvenant indépendamment à leurs besoins en termes de nourriture et des autres éléments essentiels pour vivre (36).

L'interview aura lieu exclusivement avec le répondant, qui est un membre adulte du ménage à l'âge de majorité légale spécifique au pays. Cette personne peut être identifiée comme décideur par les membres du même ménage et sert de substitut pour un ménage entier (15). D'autres définitions pertinentes sont expliquées subséquemment.

Collecte de données

Les intervieweurs de site recevront une formation sur la localisation des points géographiques, l'identification des répondants, les procédures de consentement éclairé en mettant l'accent sur la participation volontaire, et sur le déploiement de formulaires d'étude standardisés et pré-testés avant le lancement de HPAfrica (58). Les formulaires de consentement éclairé et d'étude seront traduits dans la langue officielle du pays par deux traducteurs bilingue indépendants parlant couramment l'anglais et les locuteurs natifs de la langue cible qui connaissent le concept et la terminologie des formulaires. Les deux traductions seront comparées pour les divergences avec les traducteurs et un coordinateur, et un consensus sera recherché. La traduction avancée synthétisée sera traduite et traduite de la langue cible vers l'anglais par deux traducteurs indépendants, aveugles aux formes originales. Un consensus sur les écarts de traduction et les formulaires originaux seront recherchés avec les traducteurs et un coordinateur. Les formes traduites feront l'objet d'un test pilote parmi un échantillon de commodité des ménages de la population cible avant leur finalisation pour assurer la compréhensibilité interculturelle et l'équivalence sémantique, idiomatique, expérientielle et conceptuelle. Un rapport de traduction sera préparé (58-60).

Gestion de données

For peer review only - http://bmjopen.bmj.com/site/about/guidelines.xhtml

La gestion des données dépendra du mode de collecte, qui sera principalement électronique plutôt que sur support papier. Les expériences passées ont montré les inconvénients suivants de la collecte de données sur papier: une forte probabilité d'erreurs lors du remplissage des formulaires d'étude, et le besoin de numérisation des données, qui est une étape supplémentaire, laborieuse, qui consomme du temps et sujette aux erreurs. La capture des données électroniques à l'aide d'une application pour un appareil mobile, comme un smartphone ou une tablette, réduit ces facteurs limitants à la qualité des données.

Il peut y avoir une période pilote au cours de laquelle des données sur support papier et sans papier seront collectées avant la mise en œuvre complète de la collecte de données électroniques. Ce dernier utilise l'application Android « HPA Collect » (Google Android 5.0.1 9API 23, disponible à l'adresse https://play.google.com/store/apps/details?id=anint.ivi.hpa) et la plate-forme « HPA Web » (CSS, JavaScript et JSP, disponible sur http://hpa.ivi.int/), qui sont accompagnés d'un dictionnaire de variables contenant les codes, la signification et les propriétés des variables au moins en anglais et en français. Les deux, « HPA Collect » et « HPA Web », seront conçus spécifiquement pour les besoins de cette étude. L'expertise d'IVI en ingénierie informatique permet la construction fondamentale, la configuration et le développement des composants nécessaires. Différent de nombreux programmes de source ouverte, une fonction d'installation serveur permet le stockage de toutes les données originales collectées sur le serveur institutionnel en plus des fonctionnalités supplémentaires pour la vérification des erreurs profondes, les relations logiques entre variables et formulaires, les fonctions de recherche et l'analyse des données collectées. Une connectivité Internet sans fil stable utilisant des navigateurs par défaut est nécessaire pour assurer une synchronisation simultanée, attribuable, originale et précise des données entre « HPA Collect » et « HPA Web ». L'accès à « HPA Collect » et « HPA Web » est protégé par un mot de passe. L'annexe 5 affiche le diagramme du système, qui a les caractéristiques suivantes:

Structure ou fonctionnalités de « HPA Collect »

- **Gestion des utilisateurs:** Une interface utilisateur simple fournit différents privilèges aux utilisateurs pour entrer, modifier (sauf pour les étiquettes d'étude) et exporter ou transférer des données. Il établit une piste d'audit qui enregistre lorsque les utilisateurs entrent des données dans le serveur.
- **Saisie de données:** Les données sont entrées directement dans le smartphone ou la tablette. Les champs de saisie limitent les réponses possibles à des réponses plausibles uniquement en offrant différentes méthodes de saisie:
    - Modifier le texte: texte libre
    - Groupe radio / bouton Multi-clic: choix unique (radio group/Multi click button)
    - Case à cocher: choix multiple
    - Sélecteur de date et d'heure: date et heure d'entrée
    - Spinner: bouton déroulant

  Des fonctionnalités supplémentaires permettent à l'enquêteur de gagner du temps en transférant les données collectées plus tôt aux champs de données requis à un stade ultérieur de l'interview en effectuant des vérifications de vraisemblance pour divers champs de saisie de données et en reconnaissant facilement les modèles de saut. Les vérifications d'erreurs, les notifications de données manquantes, les erreurs de code et les erreurs logiques sont également incluses.
- **Vue des données:** Les données brutes entrées à l'origine peuvent être vues dans le même format lisible par l'homme et la machine, à savoir le format JSON, étant donné qu'elles seront transférées vers un serveur de base de données (Windows, MySQL). Des fonctions supplémentaires telles que «effacer» pour effacer tous les enregistrements, «réinitialiser» pour revenir à la page de vue de données principale, et «rechercher» pour rechercher une étiquette d'étude sont disponibles.
- **Exporter :** Toutes les données entrées seront envoyées au serveur IVI via une connexion Internet sans fil. Chaque enregistrement de formulaire ayant une étiquette d'étude unique du ménage inscrit est transformé en une requête d'insertion SQL et inséré sur le serveur

For peer review only - http://bmjopen.bmj.com/site/about/guidelines.xhtml

uniquement s'il n'y a pas d'étiquette d'étude en double trouvée. L'insertion de données sera ignorée dans le cas d'étiquettes d'étude dupliquées.

- **Exporter ers local :** Tous les données entrées seront sauvegardées trois fois dans un dossier de stockage public de l'appareil mobile au format JSON et txt. Les fichiers txt contiennent des données de chaque formulaire individuel. Une version de fichiers JSON contient des données par chaque formulaire individuel et une deuxième version de fichiers JSON contient toutes les données d'un ménage inscrit. Les données au format JSON peuvent être extraites par USB ou connexion Internet sans fil.
- **Rapport :** Cette fonctionnalité permet à un utilisateur de signaler toute requête à l'administrateur principal de « HPA Collect » à IVI par e-mail our par une application tierce.
- **Paramètres:** la possibilité de changer la langue du système entre l'anglais et le français est fournie.
- **À propos de:** La version actuellement installée de «HPA Collect» est affichée.

Structure ou caractéristiques de « HPA Web »

- **Accueil:** une introduction à la plateforme est donnée et un tutoriel en français et en anglais sera accessible.
- **Aperçu du Formulaire:** Les formulaires d'étude sont affichés séparément montrant chaque question et les données d'entrée correspondantes en tant que variables définies.
- **Modifier le formulaire:** cette fonction permet la correction des données téléchargées. Il est uniquement accessible aux gestionnaires de données de chaque pays participant et à IVI.
- **Cherche:** Les étiquettes d'étude spécifiques données à chaque formulaire d'étude peuvent être filtrées.
- **Dictionnaire des variables:** Le dictionnaire variable peut être téléchargé.
- **Exporter:** Les données peuvent être converties à partir du serveur dans un fichier Excel transférable.
- **Contactez-nous:** Une correspondance rapide et facile entre les utilisateurs et le gestionnaire de données à IVI est donnée.

Les données seront examinées quotidiennement par le personnel clé de l'étude et vérifiées en termes de consistance et d'exactitude avant l'analyse des données. La qualité des données peut être vérifiée en sélectionnant un sous-ensemble de 5% pour la validation par rapport aux données originales sur support papier, si possible (58). Les noms des membres du ménage ne seront pas liés aux formulaires d'étude de HPAfrica et ne seront pas enregistrés dans la base de données afin d'assurer la confidentialité. L'accès à la base de données sera limité au personnel d'étude autorisé uniquement et les données seront conservées dans un endroit sécurisé et protégé. Des fichiers de sauvegarde provisoires périodiques et au moins trois fichiers de sauvegarde définitifs de la base de données stockés dans un endroit sécurisé et verrouillé éviteront la perte de données et garantiront la sécurité des données. Toutes les données seront conservées pendant au moins cinq ans.

L'analyse des données

Les poids d'échantillonnage combinés seront calculés pour tenir compte des différences possibles dans les probabilités de sélection et pour accroître la précision des estimations de l'étude (22, 61). Les poids sont générés dans une approche à plusieurs niveaux pour prendre en compte des probabilités de sélection de par exemple les ménages, les poids inverses au sein des ménages (c'est-à-dire la structure à plusieurs étages à plusieurs familles, la structure à plusieurs étages multifamiliale) et les poids inverses parmi les membres du ménage participant à l'étude HPAfrica (21, 43). Les outils de source ouverte, les données de recensement et les données générées par HPAfrica telles que la taille de la population, le nombre de ménages ou de structures, le nombre de ménages dans les structures multifamiliales / multi-étages et la taille des ménages par strate seront utilisés pour calculer poids (62, 63).

Des analyses descriptives telles que les fréquences absolues et relatives, les médianes ou les moyennes arithmétiques seront effectuées pour construire une description contemporaine de la population de base.

For peer review only - http://bmjopen.bmj.com/site/about/guidelines.xhtml

Les analyses de base comprendront le calcul des proportions stratifiées selon l'âge et le sexe, des comportements sanitaires *génériques* et *réels*, y compris l'utilisation des soins de santé, pour divers signes et symptômes ou combinaisons de signes et de symptômes. Ces analyses permettront une classification graduelle basée sur la sévérité de la maladie, incluant un intervalle de confiance de 95%. Le comportement de santé sera ajusté pour un effet de regroupement naturel présumé parmi les membres du ménage. Des données stratifiées les plus à jour sur la population par site et les données démographiques dérivées de HPAfrica seront utilisées pour générer les proportions pour l'ajustement des calculs d'incidence.

Les informations socio-économiques collectées seront utilisées pour construire des indices de richesse des populations étudiées en appliquant des analyses factorielles basées sur la méthode des composantes principales (38). De plus, des analyses de régression bi et multivariée seront effectuées pour identifier les facteurs de confusion potentiels ou la modification des effets en utilisant les scores factoriels.

Les analyses de régression bi et multivariée seront effectuées en utilisant les scores factoriels pour l'évaluation des variables suivantes:

- Données socioéconomiques telles que l'éducation, le revenu (64), l'assurance maladie, le logement et les biens des ménages
- Hygiène et assainissement tels que l'accès aux toilettes, les sources d'eau, la manipulation des aliments, l'élimination des déchets et les contacts avec les animaux
- Comportement sanitaire: *réel* (stratifié selon l'âge et le sexe, gravité, fréquence), *générique* (stratifié selon l'âge et le sexe, gravité, classement par choix)
- Modalités de déplacement vers les établissements de santé, stratifiées selon l'âge
- Saisonnalité liée à l'hygiène et à l'assainissement, comportement sanitaire, modalités de déplacement
- Facteurs culturels / religieux / ethnographiques, stratifiés selon l'âge
- Statut de vaccination parmi les enfants ≤ 6 ans (18, 19)
- Connaissance et perception des maladies courantes

Considérations éthiques

Il n'y a pas d'avantages directs pour les ménages inscrits et leurs membres. Les avantages indirects pour la population générale sur les sites seront les informations mises à jour sur le fardeau des maladies transmissibles, y compris les facteurs influençant la transmission de la maladie et l'infection. L'étude HPAfrica génère des évidences pour aider les décideurs à introduire des mesures appropriées pour la prévention et le contrôle des maladies.

Ce protocole a fait l'objet d'examens par le Comité de Révision Institutionnelle d'IVI et des comités locaux spécifiques aux sites, comme indiqué dans la section «Références éthiques». Pendant et après l'étude HPAfrica, toutes les données des sujets inscrits seront gardés avec une stricte confidentialité et ne seront pas divulguées à une tierce partie par aucun membre de l'équipe de recherche. La protection par mot de passe des appareils et de la base de données est utilisée pour une stricte confidentialité. Toutes les données sur support papier (formulaires de consentement) seront conservées dans un endroit sécurisé et verrouillé. Les informations confidentielles stockées sur des ordinateurs et des sources de données sur support papier seront seulement mises à la disposition des co-chercheurs et du personnel d'IVI directement impliqués dans les activités d'étude de HPAfrica.

Abbréviations

JSON: JavaScript Objective Notation: CSS: Cascading Style Sheets: JSP: Java Server Page; SQL: Structured Query Language

For peer review only - http://bmjopen.bmj.com/site/about/guidelines.xhtml

Définitions/Terminologie

**A**	
Automédication	Fait de se médicamenter soi-même ou de traiter sa propre maladie ou son état sans supervision médicale ou ordonnance.
**B**	
**C**	
Choléra	Maladie infectieuse de l'intestin grêle qui peut être mortelle causée par la bactérie *Vibrio cholerae* ; elle se propage par voie fécale-orale par l'ingestion d'eau et d'aliments contaminés et provoque fièvre, diarrhée aqueuse sévère, vomissements et déshydratation.
Confusion/Vertiges/Perte de connaissance	L'état de désorientation ou d'absence de clarté d'esprit ; l'état anormal de réponse aux stimuli dans l'environnement; faible, wooziness, faiblesse, instabilité ou tendance à tomber; l'interruption de la conscience de soi et de son environnement.
Convulsion	Mouvement soudain, violent et irrégulier d'un membre/d'une partie du corps ou de tout le corps causé par une contraction involontaire des muscles.
Coqueluche (Pertussis)	La coqueluche est une maladie bactérienne très contagieuse. Les symptômes d'un froid commun comme nez qui coule, fièvre et toux sont suivis par des semaines de toux sévère et un son aigu de la coqueluche ou haletant quand une personne malade inspire; toux sévère peut être suivie de vomissements, rupture des côtes ou fatigue extrême. Les très jeunes et les personnes âgées infectées peuvent avoir peu ou pas de toux, ou peuvent avoir des périodes de respiration interrompue pendant lesquelles elles ne respirent pas. La coqueluche est transmise par la bactérie *Bordetella pertussis* par toux et éternuements d'une personne infectée.
**D**	
Déshydration	Une condition de perte excessive d'eau/d'hydratation par le corps.
Diarrhée	Une condition d'évacuer les matières fécales du corps très fréquemment et sous forme plutôt liquide que solide.
Difficultés respiratoires/ Respiration sévère	Une condition de la respiration anormale accompagnée d'une respiration sifflante, d'une douleur thoracique, d'un tirage thoracique, d'un stridor, d'un gonflement de la poitrine ou d'une toux par exemple.
Diphthérie	Maladie hautement contagieuse, parfois mortelle, causée par la bactérie *Corynebacterium diphtheriae*. Elle se transmet de personne à personne, par l'air ou les objets contaminés et affecte les voies respiratoires supérieures. Les symptômes courants sont maux de gorge, gonflement des glandes/ ganglions lymphatiques, malaise, fièvre/frissons, substance membraneuse/matière grise et épaisse qui se forme dans la gorge/sur les amygdales et gêne/accélère la respiration (toux rauque) et gêne pour avaler. Une forme de diphtérie qui implique la peau, les yeux et les organes génitaux est également signalée et se caractérise par une peau douloureuse, rouge et enflée.
Douleur abdominale/ Douleur intestinale	Douleur (aiguë/courte durée ou chronique/longue durée) qui survient entre la poitrine et l'aine (souvent désignée comme la région de l'estomac/du ventre)
Douleur intestinale/Douleur abdominale	Douleur (aiguë/courte durée ou chronique/longue durée) qui survient entre la poitrine et l'aine (souvent désignée comme la région de l'estomac/du ventre)

For peer review only - http://bmjopen.bmj.com/site/about/guidelines.xhtml

Douleur/Maux	État caractérisé par une douleur sourde continue/prolongée dans une partie du corps ; la sensation/souffrance/inconfort physique causée par une maladie/une blessure/quelque chose qui fait mal au corps.
**E**	
Éducation formelle	Modèle d'éducation systématique et organisée structuré et administré selon un ensemble de lois et de normes ; les institutions d'éducation formelle sont organisées de façon administrative, physique et pédagogique et exigent des étudiants une assiduité minimum ; il implique des évaluations intermédiaires et finales afin d'accéder au niveau d'apprentissage suivant ; il délivre des certificats et des diplômes conformément à un ensemble de règles strictes.
Essoufflement/ Respiration rapide	Fait de respirer anormalement rapidement et profondément ; à une fréquence de >20 respirations par minute pour les adultes, >30 respirations par minute pour les enfants et >45 respirations par minute par les bébés.
Établissement de santé	Lieux ou institutions où des soins de santé sont prodigués ; dont les hôpitaux, les cliniques, les centres de santé, les postes de santé et les centres de soins spécialisé.
Éternuement	Fait d'expulser soudainement l'air par le nez et la bouche avec un bruit important caractéristique.
**F**	
Faiblesse/Malaise/Fatigue	État de se sentir fatigué/épuisé/à bout de forces ; sentiment général d'inconfort/malaise/de ne pas être en bonne santé ou heureux.
Fatigue/Malaise/ Faiblesse	État de se sentir fatigué/épuisé/à bout de forces ; sentiment général d'inconfort/malaise/de ne pas être en bonne santé ou heureux.
Fièvre	Température corporelle anormalement élevée (habituellement au-dessus de 38°C).
Fièvre <3 jours	Température corporelle anormalement élevée (habituellement au-dessus de 38°C) qui dure moins de 3 jours et apparaît en continu.
Fièvre >3 jours	Température corporelle anormalement élevée (habituellement au-dessus de 38°C) qui dure plus de 3 jours et apparaît en continu.
Fièvre jaune	Maladie infectieuse aiguë, pathogène pour l'homme et virale caractérisée par l'apparition soudaine de symptômes comme fièvre, frissons, perte d'appétit, nausée, douleurs musculaires et maux de tête qui disparaissent généralement en quelques jours ou sont suivis de symptômes plus graves comme jaunisse, fièvre forte et hémorragie. La maladie est causée par un virus du genre Flavivirus et est transmise par la piqûre d'un moustique infecté.
Fièvre typhoïde	Maladie infectieuse bactérienne potentiellement mortelle causée par *Salmonella typhi* ; elle se transmet par la voie fécale-orale par les aliments et l'eau contaminés ou de personne à personne. Les symptômes courants sont forte fièvre, maux de tête, douleur abdominale, constipation/diarrhée et éruption cutanée. Des complications graves (perforation de l'intestin) sont rapportées.
Frissons	Sensation de froid qui peut être accompagnée de frissons/tremblements du corps et de pâleur de la peau
**G**	
Gonflement/Œdème	État d'accumulation excessive de liquide aqueux/gonflement des cavités ou des tissus du corps ; couramment constaté dans les mains/bras/pieds/jambes ; augmentation du volume de tissu.
Grippe	Maladie infectieuse hautement contagieuse des voies respiratoires causée par le virus de la grippe ; elle se propage de personne à personne

For peer review only - http://bmjopen.bmj.com/site/about/guidelines.xhtml

	par les particules expulsées par la toux/les éternuements/la parole et les objets contaminés. Les symptômes courants sont fièvre, douleurs dans les muscles/bras/jambes, frissons/sueur, maux de tête, fatigue/faiblesse, nez qui coule/congestion nasale, éternuements, maux de gorge et toux sèche et persistante.
Guérisseur traditionnel	Personne qui cherche à maintenir la santé aussi bien qu'à prévenir, diagnostiquer et améliorer ou traiter les maladies en utilisant un ensemble de connaissances, compétences et pratiques basées sur des théories, des croyances et des expériences.
**H**	
Haemophilus influenzae de type B	Bactérie qui provoque une série de maladies et se propage par les particules expulsées par la toux ou les éternuements, et surviennent surtout chez l'enfant de moins de 5 ans ; les symptômes courants sont fièvre, maux de tête et raideur dans le cou jusqu'à l'état septique. Elle peut causer otites, cellulites (infection des tissus mous), arthrite, infections des voies respiratoires supérieures, pneumonies, méningites (avec dommages potentiels au cerveau) et épiglottites (avec obstruction des voies aériennes).
Hémorragie (interne/externe)/ Perte de sang	État caractérisé par une perte de sang du système vasculaire, soit de façon interne à l'intérieur du corps (hémorragie interne) soit de façon externe par un orifice naturel ou une lésion de la peau (hémorragie externe) ; sang qui s'échappe d'un vaisseau sanguin endommagé.
Hépatite B	Maladie infectieuse causée par le virus de l'hépatite B (VHB) qui affecte le foie (infection hépatique aiguë/chronique ; peut entraîner une insuffisance hépatique/un cancer/une cirrhose). Le virus se transmet de personne à personne par le sang/le sperme/les autres fluides corporels. Les symptômes courants sont fièvre, malaise/faiblesse/fatigue, perte d'appétit, nausée/vomissements, gêne/douleur abdominale, urines foncées, douleurs articulaires et jaunisse.
Hépatite E	Maladie infectieuse causée par le virus de l'hépatite E (VHE) qui affecte le foie (infection hépatique aiguë uniquement ; peut entraîner une insuffisance hépatique aiguë). Le virus se propage par les voies fécales-orales par l'ingestion d'eau ou d'aliments contaminés, les animaux contaminés (de façon zoonotique) et par le sang. Les symptômes courants sont fièvre, malaise, perte d'appétit, diarrhée, nausée, gêne abdominale, urines foncées, douleurs articulaires et jaunisse.
**I**	
**J**	
Jaunisse	Maladie qui fait jaunir la peau/le blanc des yeux.
**K/L**	
**M**	
Malaise/Fatigue/ Faiblesse	État de se sentir fatigué/épuisé/à bout de forces ; sentiment général d'inconfort/malaise/de ne pas être en bonne santé ou heureux.
Maux de tête	Douleur continue dans une région de la tête.
Maux/Douleur	État caractérisé par une douleur sourde continue/prolongée dans une partie du corps ; la sensation/souffrance/inconfort physique causée par une maladie/une blessure/quelque chose qui fait mal au corps.
Médecin	Une personne compétente/formée aux soins ; une personne éduquée, cliniquement expérimentée et autorisée à pratiquer la médecine.
Ménage	Personne ou groupe de personnes apparentées ou non qui vivent ensemble dans la même unité d'habitation, qui reconnaissent un adulte de sexe masculin ou féminin comme le chef du ménage, qui partagent

For peer review only - http://bmjopen.bmj.com/site/about/guidelines.xhtml

	les mêmes installations domestiques, qu'on considère comme formant une unité, et qui subviennent à leurs besoins en termes de nourriture et des autres éléments essentiels pour vivre. Un ménage peut être situé dans une unité d'habitation unique (bâtiment à un seul étage) ou dans un ensemble de logements collectifs (bâtiment à plusieurs étages).
Méningite	Inflammation virale/bactérienne/fongique infectieuse parfois mortelle des membranes (méninges) qui entourent le cerveau et la moelle épinière ; elle se propage de personne à personne par les particules expulsées par la toux/les éternuements/la parole et les objets contaminés. Les symptômes courants sont forte fièvre, maux de tête sévères, nausée/vomissements, confusion, convulsions ; perte d'appétit et de la soif, fatigue, sensibilité à la lumière, éruption cutanée et raideur au cou.
**N**	
Nausée/Vomissements	Sensation de malaise dans l'estomac avec tendance à vomir ; fait d'éjecter une partie/tout le contenu de l'estomac principalement par la bouche.
Nez qui coule	État résultant de la production par le nez (tissus nasaux et adjacents et vaisseaux sanguins) de mucus/drainage excessif, qui coule du nez pour retirer quelque chose.
**O**	
Œdème/Gonflement	État d'accumulation excessive de liquide aqueux/gonflement des cavités ou des tissus du corps ; couramment constaté dans les mains/bras/pieds/jambes ; augmentation du volume de tissu.
Oreillons	Maladie infectieuse virale causée par le virus des oreillons qui affecte principalement les glandes parotides qui sont situées sous et devant les oreilles ; elle se propage de personne à personne par la salive infectée/les particules de salive infectées expulsées par la toux/les éternuements/la parole et les objets contaminés. Les symptômes courants sont fièvre, gonflement d'une/des deux glandes parotides, maux de tête ; douleurs musculaires, faiblesse/fatigue, perte d'appétit et douleurs en mâchant/avalant ; une complication connue est une possible perte d'audition.
**P**	
Paludisme	Maladie infectieuse parfois mortelle causée par un parasite (*Plasmodium* spp.) transmis principalement par la piqûre de moustiques infectés, en dehors de la transmission mère-enfant et par le sang. Les symptômes courants sont attaques récurrentes de frissons, sueurs, forte fièvre, maux de tête, vomissements et diarrhée.
Personne interrogée	Membre adulte (à l'âge légal de majorité du pays concerné) du ménage qui est un décideur en ce qui concerne les soins de santé/l'utilisation des soins de santé pour le ménage entier à l'intérieur du site d'étude ; cette personne adulte parle ainsi au nom de tous les membres du ménage. Cette personne peut être identifiée par les autres membres du même ménage comme la personne qui est principalement impliquée dans les soins quotidiens des membres du ménage.
Perte de connaissance/ Vertiges/Confusion	L'état de désorientation ou d'absence de clarté d'esprit ; l'état anormal de réponse aux stimuli dans l'environnement; faible, wooziness, faiblesse, instabilité ou tendance à tomber; l'interruption de la conscience de soi et de son environnement.
Perte de poids	Baisse (volontaire/involontaire) du poids du corps.
Perte de sang/ Hémorragie	État caractérisé par une perte de sang du système vasculaire, soit de façon interne à l'intérieur du corps (hémorrage interne) soit de façon

For peer review only - http://bmjopen.bmj.com/site/about/guidelines.xhtml

	externe par un orifice naturel ou une lésion de la peau (hémorragie externe) ; sang qui s'échappe d'un vaisseau sanguin endommagé.
Pharmacie	Endroit où seuls des médicaments (ordonnance requise/non requise) sont préparés, conservés, composés et dispensés.
Pneumocoque	Une inflammation infectieuse bactérienne des poumons parfois mortelle qui affecte souvent les personnes immunodéprimées et est causée par la bactérie *Streptococcus pneumoniae* ; elle se propage de personne à personne par les particules expulsées par la toux/les éternuements/la parole et les objets contaminés. Les symptômes courants sont fièvre, frissons, toux (avec mucosités/pus), douleurs thoraciques, difficulté à respirer/essoufflement, fatigue, nausée/vomissements et diarrhée ; des symptômes rares sont septicémie, épanchement pleural et empyème.
Polio	Maladie virale infectieuse, très contagieuse, parfois mortelle, causée par le poliovirus qui cause dans sa forme la plus sévère paralysie et difficultés à respirer ; il se transmet par la voie fécale-orale par ingestion d'eau et d'aliments contaminés et de personne à personne. Les symptômes courants sont fièvre, maux de gorge, maux de tête, vomissements, fatigue, maux de dos, de cou/raideur au cou, faiblesse des muscles/muscles douloureux, méningite, perte de réflexes, membres lourds/pendants.
Occupation	A job, or means of earning a wage/living; often requires the mastery of a complex set of knowledge, tasks, duties and skills acquired through formal education and/or practical experience.
**Q**	
**R**	
Respiration rapide/ Essoufflement	Fait de respirer anormalement rapidement et profondément ; à une fréquence de >20 respirations par minute pour les adultes, >30 respirations par minute pour les enfants et >45 respirations par minute par les bébés.
Respiration sévère/ Difficultés respiratoires	Une condition de la respiration anormale accompagnée d'une respiration sifflante, d'une douleur thoracique, d'un tirage thoracique, d'un stridor, d'un gonflement de la poitrine ou d'une toux par exemple.
Revenus/Salaire	Argent ou autre forme de paiement que quelqu'un reçoit périodiquement/régulièrement en échange de biens ou de services. Provient généralement d'un emploi/d'une profession.
Rotavirus	Infection virale causée par le rotavirus qui cause principalement des diarrhées pour les enfants de moins de 5 ans ; elle se propage par la voie fécale-orale, de personne à personne et par les objets contaminés. Les symptômes courants sont diarrhée aqueuse, fièvre, vomissements, douleurs abdominales et déshydratation.
Rougeole	Maladie infectieuse parfois mortelle des voies respiratoire (nez/gorge) causée par un virus qui affecte principalement les enfants de moins de 5 ans ; elle se propage de personne à personne par les particules expulsées par la toux/les éternuements/la parole et les objets contaminés. Les symptômes courants sont fièvre, toux sèche, nez qui coule, inflammation des yeux, maux de gorge, larges plaques sur la peau et points blancs dans la bouche et sur les joues.
Rubéole	Maladie infectieuse virale contagieuse connue pour son éruption cutanée rouge distinctive qui est causée par le virus de la rubéole ; elle se transmet de personne à personne (toux/éternuements/parole), de la mère à l'enfant par le sang et les objets contaminés. Les symptômes courants sont fièvre modérée, maux de tête, nez bouché/qui coule, yeux rouges/inflammation des yeux, ganglions lymphatiques gonflés et

For peer review only - http://bmjopen.bmj.com/site/about/guidelines.xhtml

	sensibles à la base du crâne/à l'arrière du cou/derrière les oreilles, douleurs articulaires et éruption cutanée rose qui se déplace du visage au torse, aux bras et aux jambes.
**S**	
Soins de santé - généraux	Soins de santé pour les maladies qui n'ont aucun lien avec l'apparition de maladies associées aux signes et symptômes ou les dates de début de maladies.
Soins de santé - réels	Soins de santé liés l'apparition de maladies associées aux signes et symptômes ou les dates de début de maladies.
**T**	
Témoin	Personne adulte (à l'âge légal de majorité du pays concerné), qui est indépendante de l'étude, qui ne peut pas être indûment influencée par le personnel de l'étude, qui participe au processus de consentement éclairé si le sujet/le représentant légal du sujet est analphabète, et qui lit (lettré) le formulaire de consentement éclairé et toute autre information écrite présentée au sujet.
Tétanos	Une maladie infectieuse caractérisée par des spasmes musculaires qui commencent généralement dans la mâchoire et progresse vers le reste du corps. Les spasmes durent généralement quelques minutes et se produisent fréquemment pendant les 3-4 semaines au début de la maladie, ils peuvent être si graves qu'ils peuvent causer des fractures osseuses ou la mort lorsqu'ils affectent les muscles respiratoires. Elle peut être caractérisée par d'autres symptômes comme fièvre, sueurs, maux de tête, difficultés à avaler, pression artérielle élevée et augmentation du rythme cardiaque. La maladie est causée par une infection par la bactérie *Clostridium tetani* qui se trouve dans sol, saliva, poussière et fumier. La bactérie entre par une lésion cutanée (par exemple, coupure, blessure par ponction) par un objet contaminé. Il produit des toxines qui interfèrent avec les contractions musculaires qui résultent des spasmes typiques.
Toux	Fait d'expulser de façon soudaine/rapide et bruyamment l'air des poumons ; souvent de façon involontaire afin de dégager les voies respiratoires des poumons de fluides/mucus/autres matières
Troubles cardiaques	Tout trouble que affecte le cœur ; maladies qui impliquent des vaisseaux sanguins rétrécis/bloqués qui peuvent entraîner une attaque cardiaque/douleur thoracique/accident vasculaire cérébral ; maladie qui affecte le muscle/les valves/le rythme du cœur.
Troubles de la pression artérielle	Pression artérielle élevée/faible ou pression artérielle qui présente des fluctuations excessives.
Tuberculose	Maladie infectieuse bactérienne (*Mycobacterium tuberculosum*), contagieuse, potentiellement grave qui affecte principalement les poumons en plus des reins et de la colonne vertébrale/du cerveau ; elle se transmet de personne à personne par les particules expulsées par la toux/les éternuements/la parole. Les symptômes courants d'une tuberculose active sont toux (avec/sans sang), douleur thoracique, perte de poids, fatigue, fièvre, sueurs nocturnes, frissons et perte d'appétit.
**U**	
**V**	
Vertiges/Confusion/Perte de connaissance	L'état de désorientation ou d'absence de clarté d'esprit ; l'état anormal de réponse aux stimuli dans l'environnement; faible, wooziness, faiblesse, instabilité ou tendance à tomber; l'interruption de la conscience de soi et de son environnement.

For peer review only - http://bmjopen.bmj.com/site/about/guidelines.xhtml

VIH/SIDA	Maladie infectieuse causée par le virus de l'immunodéficience humaine (VIH) qui affecte/endommage le système immunitaire et entraîne le syndrome immunodéficitaire acquis (SIDA), une maladie chronique et potentiellement mortelle ; il se transmet par les liquides corporels infectieux comme le sang/le sperme/les autres fluides corporels. Les symptômes courants d'une infection aiguë/précoce sont fièvre, fatigue, maux de tête, douleurs articulaires, éruption cutanée, maux de gorge, ganglions lymphatiques enflés, diarrhée, perte de poids, candidose orale et zona. La maladie non traitée évolue en SIDA ; les symptômes causés par un système immunitaire gravement endommagé sont sueurs, fièvre récurrente, diarrhée chronique, lésions sur la langue et dans la bouche, fatigue, perte de poids et éruptions cutanées.
Visiteur	Personne (apparentée ou non) qui rend visite à un ménage pour passer du temps avec les membres du ménage ; un visiteur ne vit pas avec les membres du ménage dans la même unité d'habitation, ne partage pas les mêmes installations domestiques, la même nourriture et autres éléments essentiels à la vie que les membres du ménage, et ne constitue pas une unité avec les membres du ménage.
Voisin	Personne (apparentée ou non) vivant dans l'habitation à côté de celle d'un ménage cible ; un voisin ne vit pas avec les membres d'un ménage (à côté) dans la même unité d'habitation, ne partage pas les mêmes installations domestiques, aliments ou autres éléments essentiels à la vie que les membres du ménage (à côté), et ne constitue pas une unité avec les membres du ménage (à côté).
Vomissements/Nausée	Sensation de malaise dans l'estomac avec tendance à vomir ; fait d'éjecter une partie/tout le contenu de l'estomac principalement par la bouche.
**W/X/Y/Z**	

For peer review only

Formulaires de l'étude

Site ___ ___ ___ Sous-zone ___ ___ ___ Numéro d'habitation ___ ___ ___ ___ Identifiant de l'enquêteur ___ __

FORMULAIRE 1 – COMPTE RENDU DES VISITES AUX MÉNAGES

Instructions pour l'enquêteur

- Compléter « Formulaire 1 – COMPTE RENDU DES VISITES AUX MÉNAGES » pour chaque ménage visité.
- Compléter « Foyer/Personne interrogée/Complété » une fois que toutes les parties de l'enquête ont été complétées ; si toutes les données n'ont pas été recueillies lors de la visite initiale et/ou d'une visite ultérieure (max. 3 visites), programmer une nouvelle visite.
- Tentative 1: Compléter le statut de la visite au ménage et utilisant les choix proposés ; pour « pas à la maison », « à la maison/ne répond pas » et « à la maison/répond/indisponible », programmer une nouvelle visite ; pour « à la maison/répond/refus » indiquez la raison de refus et visitez un ménage de remplacement au plus proche à droite ou à gauche de ce ménage; pour « inexistant/pas d' habitation », passer ce ménage et visiter un ménage de remplacement au plus proche à droite ou à gauche à la place de ce ménage.
- Tentative 2: Compléter le statut de la visite du ménage et utilisant les choix proposés ; pour « pas à la maison », « à la maison/ne répond pas » et « à la maison/répond/indisponible », programmer une nouvelle visite.
- Tentative 3: Compléter le statut de la visite au ménage et utilisant les choix proposés ; pour « pas à la maison », « à la maison/ne répond pas » et « à la maison/répond/indisponible », passer ce ménage et visiter un ménage de remplacement au plus proche à droite ou à gauche à la place de ce ménage.
- « Site » et « sous-zone » doivent être un numéro à trois chiffres, « numéro d'habitation » un numéro à quatre chiffres, et « identifiant de l'enquêteur » un numéro à deux chiffres (utiliser les listes préparées individuellement pour chaque site pour obtenir les codes) ; saisir « date » au format JJ/MM/AAAA et « heure » au format HH:MM et entourer MATIN ou APRÈS-MIDI. «Étiquette de l'étude» doit être une combinaison d'un code «site» à trois chiffres, d'un code «sous-zone» à trois chiffres et d'un code «numéro d'habitation» à quatre chiffres; le « identifiant de l'enquêteur » doit être un numéro à trois chiffres.

**Tentative 1**	Date ____/____/____ (JJ/MM/AA)	Heure _____/_____ MATIN/APRÈS-MIDI	Identifiant de l'enquêteur ___ ___
STATUT		Cocher un	
1=Pas à la maison		O	➡ Planifier nouvelle visite (date/heure)
2=À la maison/ Non-répondant		O	➡ Planifier nouvelle visite (date/heure)
3=À la maison/ répondant /Indisponible		O	➡ Planifier nouvelle visite (date/heure)
4= À la maison/ Répondant /Complété		O	
5= À la maison/ répondant /Refus		O	Notez la/les raison(s) du refus; cochez tout ce que s'applique:<br>O 1=Pas de temps pour répondre aux questions<br>O 2=Ne veulent pas participer/répondre aux questions<br>O 3=Autre ______________<br>O 99=Pas de réponse O 98=Ne sait pas<br>➡ Visitez un ménage de remplacement le plus proche de ce ménage du côté droit ou gauche

For peer review only - http://bmjopen.bmj.com/site/about/guidelines.xhtml

6=Inexistant/Pas une habitation		O	➡ Visitez un ménage de remplacement le plus proche de ce ménage du côté droit ou gauche
**Tentative 2**	Date ____/____/____ (JJ/MM/AA)	Heure _____/_____ MATIN/APRÈS-MIDI	Identifiant de l'enquêteur ___ ___
STATUT		Cocher un	
1=Pas à la maison		O	➡ Planifier nouvelle visite (date/heure)
2=À la maison/Ne répond pas		O	➡ Planifier nouvelle visite (date/heure)
3=À la maison/Répond/Indisponible		O	➡ Planifier nouvelle visite (date/heure)
4= À la maison/Répond/Complété		O	
5= À la maison/ répondant /Refus		O	Notez la/les raison(s) du refus; cochez tout ce que s'applique:<br>O 1=Pas de temps pour répondre aux questions<br>O 2=Ne veulent pas participer/répondre aux questions<br><br>O 3=Autre ______________________<br>O 99=Pas de réponse O 98=Ne sait pas<br><br>➡ Visitez un ménage de remplacement le plus proche de ce ménage du côté droit ou gauche
6=Inexistant/Pas une habitation		O	➡ Visitez un ménage de remplacement le plus proche de ce ménage du côté droit ou gauche
**Tentative 3**	Date ____/____/____ (JJ/MM/AA)	Heure _____/_____ MATIN/APRÈS-MIDI	Identifiant de l'enquêteur ___ ___
STATUT		Cocher un	
1=Pas à la maison		O	➡ Visitez un ménage de remplacement le plus proche de ce ménage du côté droit ou gauche
2=À la maison/Ne répond pas		O	➡ Visitez un ménage de remplacement le plus proche de ce ménage du côté droit ou gauche
3=À la maison/Répond/Indisponible		O	➡ Visitez un ménage de remplacement le plus proche de ce ménage du côté droit ou gauche
4= À la maison/Répond/Complété		O	
5= À la maison/ répondant /Refus		O	Notez la/les raison(s) du refus; cochez tout ce que s'applique:<br>O 1=Pas de temps pour répondre aux questions<br>O 2=Ne veulent pas participer/répondre aux questions<br><br>O 3=Autre ______________________<br>O 99=Pas de réponse O 98=Ne sait pas

For peer review only

		➡ Visitez un ménage de remplacement le plus proche de ce ménage du côté droit ou gauche
6=Inexistant/Pas une habitation	○	➡ Visitez un ménage de remplacement le plus proche de ce ménage du côté droit ou gauche

For peer review only

For peer review only - http://bmjopen.bmj.com/site/about/guidelines.xhtml

Site ___ ___ ___ Sous-zone ___ ___ ___ Numéro d'habitation ___ ___ ___ ___ Identifiant de l'enquêteur ___ __

FORMULAIRE 2 – FORMULAIRE DE CONSENTEMENT

Evaluation des soins de santé à [site SETA and –pays]

Instructions à l'enquêteur

- Lire le "Formulaire 2 – FORMULAIRE DE CONSENTEMENT" pour le répondant/demander au répondant de lire attentivement le "Formulaire 2 – FORMULAIRE DE CONSENTEMENT" s'il préfère.
- Le répondant tel que défini dans cette enquête est un adulte (ayant l'âge de la majorité spécifique au pays) membre du ménage et responsable des décisions journalières concernant la santé des membres du ménage; il ne doit pas par exemple être un voisin ou un visiteur.
- S'assurer que le répondant a pleinement compris l'objectif, les procédures, les risques et les bénéfices de cette étude avant d'obtenir son consentement à participer.
- Le répondant doit signer le "Formulaire 2 – FORMULAIRE DE CONSENTEMENT"; si le répondant est incapable de signer/analphabète, une empreinte digitale servira en lieu et place de la signature, et un adulte lettré indépendant de l'étude qui servira de témoin (un membre du même ménage ou un voisin par exemple) signera le "Formulaire 2 – FORMULAIRE DE CONSENTEMENT".
- Collecter les informations sur tous les membres du ménage indépendamment du fait qu'ils soient présents ou non au moment de l'entretien; Recueillir l'information sur seulement les individus présents au moment de l'entretien résultera en un biais dans les données.
- Remplir le "Formulaire 2 – FORMULAIRE DE CONSENTEMENT" pour chaque ménage consentant enrôlé dans l'étude.
- Expliquer au répondant qu'il est prévu de revisiter chaque ménage durant une période de 2 ans; a chaque visite, l'équipe de recherche obtiendra un nouveau consentement.
- «Site» et «sous-zone» doivent être un numéro à trois chiffres, «numéro d'habitation» un numéro à quatre chiffres, et «identifiant de l'enquêteur» un numéro à deux chiffres (utiliser les listes préparées individuellement pour chaque site pour obtenir les codes); saisir «date» au format JJ/MM/AAAA et «heure» au format HH:MM et entourer MATIN ou APRÈS-MIDI. «Étiquette de l'étude» doit être une combinaison d'un code «site» à trois chiffres, d'un code «sous-zone» à trois chiffres et d'un code «numéro d'habitation» à quatre chiffres; le «identifiant de l'enquêteur» doit être un numéro à trois chiffres.

Investigateur principal et co-investigateur de l'étude

[Ajouter les noms de l'investigateur principal et des co-investigateurs de l'étude ainsi que leurs affiliations respectives]

Introduction

L'Institut International des Vaccins (IVI) et [ajouter l'institution d'affiliation des investigateurs locaux] travaillent ensemble pour examiner les problèmes de santé et le comportement de santé dans votre communauté [ajouter le site SETA-pays], y compris le statu socio-économique, le comportement d'hygiène et d'assainissement et le statut vaccinal des enfants. Nous aimerions mieux comprendre où vous, votre famille et vos voisins cherchez des soins de santé en cas de maladies causant la fièvre/corps chaud et autres chante & symptômes. Cette information nous aidera à savoir à quelle fréquence vous souffrez de maladies causant de la fièvre et les autres liées à la fièvre, fournir une meilleure image des facteurs influençant la transmission de la maladie et l'infection, et à trouver des moyens pour les prévenir et traiter dans votre communauté. Vous, en tant que responsable principal pour le comportement de santé/l'utilisation des soins de santé quotidienne pour les membres de ce ménage, êtes invités à participer parce que vous vivez dans la localité où nous effectuons cette étude. Ce formulaire de consentement sera lu par vous ou pour vous et vous aurez le temps pour prendre votre décision d'accepter ou de refuser de participer à l'étude. S'il vous plaît, demandez aux membres de l'étude d'expliquer tout mot ou toute information que vous ne comprenez pas. On vous donnera en conséquence plus d'informations sur cette étude, y compris ses risques potentiels, les avantages et les inconvénients. Après vous avoir entièrement expliqué l'étude et si vous acceptez de participer, on vous demandera de signer ce formulaire de consentement au nom de tous les membres du ménage. Si vous ne pouvez pas signer le formulaire, nous vous demandons de fournir votre empreinte digitale, et une personne adulte indépendante de l'étude (membre de votre ménage/voisin) signera le formulaire en tant que témoin. Nous allons vous remettre une copie signée et datée du formulaire de consentement.

Objectif

L'objectif de cette étude est d'examiner les problèmes de santé et l'utilisation des soins de santé dans votre communauté [ajouter le site SETA-pays], y compris le statu socio-économique et le comportement d'hygiène et d'assainissement. Notre objectif est de mieux comprendre où vous, votre famille et vos voisins cherchez les soins de santé pour des maladies causant de la fièvre/corps chaud et autres chante & symptômes. Cette information

For peer review only - http://bmjopen.bmj.com/site/about/guidelines.xhtml

permettra de mieux estimer le fardeau des maladies communes et d'identifier les moyens pour la mise en place de mesures de prévention et de traitement pour les maladies.

Procédures
Nous visiterons environ [ajouter le nombre pour site d'étude et pays] ménages au total à [ajouter le site et pays] et poserons les mêmes questions dans chaque ménage. Votre ménage a été choisi pour cette étude simplement par hasard. Si vous acceptez de participer, nous vous poserons, au nom de tous les membres du ménage, quelques questions au sujet de votre ménage tel que les initiales, le sexe et l'âge de chaque membre. Nous aimerions savoir certaines informations relatives à l'éducation et l'occupation du chef de ménage, au revenu, à la possession de biens, au logement lui-même ainsi qu'à l'hygiène et à l'assainissement dans le contexte de l'hygiène personnelle, de la préparation et de la manipulation des aliments, des sources d'eau et de la manipulation, de l'élimination des déchets, de la manipulation des animaux de ferme et domestiques ainsi que de l'élevage de ce ménage. Nous allons également demander où les membres de votre ménage obtiennent les soins si quelqu'un a une maladie causant de la fièvre ou toute autre maladie et les facteurs qui peuvent influer sur l'utilisation des soins de santé des membres du ménage (c'est-à-dire les modalités de transport, la disponibilité d'assurance maladie, les facteurs culturels) en plus du statut vaccinal et de la perception de certaines maladies. Enfin, nous allons mesurer la position géographique de votre maison. Il n'y a pas de bonnes ou de mauvaises réponses. Vous êtes libre de choisir les questions à répondre. Somme toute, il devrait prendre environ 30-45 minutes pour répondre à toutes les questions. L'entretien peut prendre un peu plus de temps s'il y a beaucoup de membres dans ce ménage. En outre, nous visiterons votre ménage deux fois en différentes saisons (une fois pendant la saison sèche et une fois vers la fin de la saison des pluies) pendant la période totale de l'étude qui est de deux ans environ pour recueillir des données comme décrit dans ce paragraphe du formulaire de consentement.

Participation
Vous, le décideur principal pour le comportement de santé quotidienne et l'utilisation des soins de santé dans ce ménage, ainsi que les membres de votre ménage, êtes libres de choisir d'accepter ou de refuser de participer à l'étude. Il n'y a aucun problème si vous ne voulez pas participer. Il n'y a non plus aucun problème si vous voulez arrêter votre participation à tout moment, et cela ne causera aucun tort à vous ou aux membres de votre ménage. Donc, la participation à cette étude est volontaire, et vous êtes libre de refuser de participer à l'étude ou vous pouvez retirer votre consentement à tout moment sans donner de raisons et ceci n'entraînera aucune pénalité.

Risques et avantages
La participation à cette étude n'entraine pas de risque direct. Poser des questions au sujet de votre ménage peut mettre mal à l'aise vous ou les membres de votre ménage. Après avoir terminé les questions, l'équipe de recherche pourra fournir des informations sur les soins de santé communautaires pour les participants qui expriment de la détresse. Vous pouvez refuser de répondre à toute question à tout moment. Vous pouvez faire une pause ou arrêter de participer à cette étude à tout moment. Il n'y a pas d'avantages directs à être dans cette étude. Au cours des visites de votre ménage, notre équipe de recherche peut identifier les membres de votre ménage qui devraient chercher des soins de santé pour les maladies causant de la fièvre et autres. Par conséquent, elle peut aider à fournir de l'information sur l'offre de soins établie dans votre communauté. Les avantages indirects sont que toutes les informations collectées contribueront à fournir des données exactes sur les problèmes de santé, y compris le statu socio-économique, le comportement d'hygiène et d'assainissement et le statut vaccinal des enfants dans votre communauté. Cela aidera à donner une meilleure compréhension des facteurs influençant la transmission de la maladie et l'infection, et à estimer le fardeau des maladies courantes ainsi qu'à trouver des moyens de prévenir et de traiter les personnes dans votre communauté.

Coûts et Compensations
Il n'a pas de coûts pour les participants de cette étude. Ni vous ni aucune personne de votre ménage ne sera indemnisée d'avoir participé.

Confidentialité
Tous les fichiers de l'étude et de toutes les informations qui identifient vous et les membres de votre ménage seront gardées confidentielles. Tous les questionnaires sur papier seront gardés en toute sécurité et les fichiers informatisés seront protégés par un mot de passe; tous les fichiers de données (sur papier/informatisés) seront stockées pour un minimum de cinq ans; ces fichiers seront accessibles seulement à quelques personnes autorisées de l'étude. Ni votre nom ni aucun identifiant ne sera utilisé dans les publications ou les rapports de cette étude. Les informations que nous collectons à votre sujet et au sujet des membres de votre ménage seront

For peer review only - http://bmjopen.bmj.com/site/about/guidelines.xhtml

partagées uniquement avec le personnel de l'étude autorisé et des représentants du comité d'éthique.

Personnes de contact pour des questions
Si vous ou des membres de votre ménage avez des questions, s'il vous plaît, demandez maintenant à l'équipe de recherche ou à tout moment plus tard en contactant [ajouter le nom de l'investigateur principal et/ou du co-investigateur, leurs affiliations respectives et les numéros de téléphone]. Si vous avez des questions sur vos droits ou ceux des membres du ménage en tant que participant à l'étude, vous pouvez contacter [ajouter le nom et les coordonnées du comité d'éthique local].

Déclaration de consentement
Ce formulaire de consentement pour participer à l'évaluation des soins de santé et d'autres facteurs liés à la population à [ajouter le site et pays] a été bien lu par moi/pour moi. L'objectif, les procédures, les risques et les avantages ont m'ont été expliqués en détails. J'ai été autorisé à poser des questions à tout moment, et mes questions ont été répondues à ma satisfaction par l'équipe de recherche. On m'a dit la personne à contacter en cas de questions ou si je veux discuter de problèmes ou préoccupations. On m'a dit que je recevrai une copie signée et datée de ce formulaire de consentement. Je suis rassuré que toutes les informations obtenues par suite de cette étude seront gardées confidentielles et utilisées uniquement aux fins de cette étude, et seulement par les institutions participantes.

Moi, le répondant, consens, par la présente, volontairement à participer à cette étude. Je suivrai les instructions de l'équipe de recherche et donnerai mon entière collaboration. Je comprends que j'ai le droit de me retirer de l'étude à tout moment.

RÉPONDANT

Nom : ________________________________
(prénom + deuxième prénom (si disponible) + nom de famille)

Signature : ________________________________
(Représentation manuscrite du nom/ marque que la personne écrit sur un document comme une preuve d'identité)

Date ______/______/______________ (YY/MM/AAAA)

Empreinte du pouce

Si le répondant ne peut pas signer/est analphabète, mais accepte de participer, enregistre l'empreinte du pouce dans la boîte ci-dessus, et demande à un témoin adulte, alphabétisé et indépendant de l'étude (par exemple membre du même ménage ou un voisin), de signer ci-dessous :

TÉMOIN

Nom : ________________________________
(prénom + deuxième prénom (si disponible) + nom de famille)

Signature : ________________________________
(Représentation manuscrite du nom/ marque que la personne écrit sur un document comme une preuve d'identité)

Date ______/______/______________ (YY/MM/AAAA)

Moi, l'enquêteur, j'ai lu/expliqué l'étude au répondant nommé ci-dessus (témoin si le répondant est analphabète) dans une langue que le répondant comprend bien. Je suis certain que le répondant a compris l'information et accepte qu'on lui pose des questions.

INTERVIEWER

Nom : ________________________________
(prénom + deuxième prénom (si disponible) + nom de famille)

Signature : ________________________________
(Représentation manuscrite du nom/ marque que la personne écrit sur un document comme une preuve d'identité)

Date ______/______/______________ (YY/MM/AAAA)

For peer review only

Site ___ ___ ___ Sous-zone ___ ___ ___ Numéro d'habitation ___ ___ ___ ___ Identifiant de l'enquêteur ___ __

FORMULAIRE 3: INFORMATIONS GÉNÉRALES SUR LE MÉNAGE

Instructions pour l'enquêteur

- Compléter ce formulaire uniquement si la personne interrogée a consenti à participer (voir « Formulaire 2 — FORMULAIRE DE CONSENTEMENT ») ; la personne interrogée ainsi que définie pour cette enquête est un adulte (selon l'âge légal de majorité du pays), un membre du ménage et un décideur en ce qui concerne les soins de santé quotidiens et l'utilisation de soins de santé pour le ménage entier et ses membre ; il ne doit pas être, par exemple, un voisin ou un visiteur.
- Recueillir les données de tous les membres du ménage que tous les membres du ménage soient présents ou non au moment de l'entretien ; ne recueillir et n'inscrire les données que des personnes présentes au moment de l'entretien fausserait les données.
- Un ménage est défini/les membres d'un ménage sont définis comme une personne ou un groupe de personnes avec ou sans lien de parenté qui vivent ensemble dans la même unité d'habitation, qui reconnaissent un adulte de sexe masculin ou féminin comme chef de ménage, qui partagent les mêmes installations domestiques, qui sont considérés comme constituant une unité, et qui subviennent à leurs besoins en termes de nourriture et des autres éléments essentiels pour vivre.
- « Site » et « sous-zone » doivent être un numéro à trois chiffres, « numéro d'habitation » un numéro à quatre chiffres, et « identifiant de l'enquêteur » un numéro à deux chiffres (utiliser les listes préparées individuellement pour chaque site pour obtenir les codes) ; saisir « date » au format JJ/MM/AAAA et « heure » au format HH:MM et entourer MATIN ou APRÈS-MIDI. «Étiquette de l'étude» doit être une combinaison d'un code «site» à trois chiffres, d'un code «sous-zone» à trois chiffres et d'un code «numéro d'habitation» à quatre chiffres; le « identifiant de l'enquêteur » doit être un numéro à trois chiffres.

FORMULAIRE 3: INFORMATIONS GÉNÉRALES SUR LE MÉNAGE
PARTIE A – DONNÉES DÉMOGRAPHIQUES/ INFORMATIONS GÉNÉRALES SUR LE MÉNAGE

EMPLACEMENT DU DOMICILE

1 Numéros de site, de sous-zone et d'habitation

Instructions : Site (=site d'étude) et sous-zone (=plus petite unité du site d'étude) doivent être tous les deux des numéros à trois chiffres, et numéro d'habitation doit être un code à quatre chiffres ; utiliser les listes préparées individuellement pour obtenir les codes respectifs.

Site: ____ ____ ____ Sous-zone: ____ ____ ____ Numéro d'habitation: ____ ____ ____ ____

2 Coordonnées GPS

Instructions : Prendre les coordonnées GPS approximativement au centre de la maison. Inscrire l'altitude par un nombre à quatre chiffres (unité : mètre), et la latitude/longitude par un numéro à huit chiffres (unités : degrés, minutes décimales).

○ 1=Ménage d'origine ○ 2=Ménage de remplacement (côte droit/gauche du ménage d'origine)

Altitude: ___ ___ ___ ___

Latitude: ___ ___°___ ___.___ ___ ___' Longitude: ___ ___°___ ___.___ ___ ___'

PERSONNE INTERROGÉE

3 Inscrire le nom complet de la personne interrogée.

Instructions : Inscrire le prénom, le deuxième nom (le cas échéant) et le nom de famille de la personne interrogée.

____

4 Inscrire l'âge de la personne interrogée.

Instruction : Utiliser un numéro à trois chiffres (unité: année). _____ _____ _____

5 Inscrire le sexe de la personne interrogée.

○ 1=Masculin ○ 2=Féminin

6 Quel est le lien de la personne interrogée avec le ménage ?

○ 1=Chef de ménage ○ 7=Conjoint du chef de ménage
○ 2=Fils/fille du chef de ménage ○ 8=Conjoint du fils/fille du chef de ménage

For peer review only

O 3=Frère/sœur du chef de ménage
O 4=Mère/père du chef de ménage
O 5=Petite-fille/petit-fils du chef de ménage
O 6=Autre, préciser __________
O 9=Conjoint du frère/sœur du chef de ménage
O 10=Belle-mère/beau-père du chef de ménage
O 11=Nièce/neveu du chef de ménage
O 98=Pas de réponse

MEMBRES DU MÉNAGE

7 Combien de membres du ménage vivent dans ce domicile au total (au moment de la visite) **?**
Instruction : Utiliser un numéro à deux chiffres.
Nombre total de membres du ménage : ____ ____

8 Inscrire l'âge et le sexe de chaque membre du ménage.
Instructions : Inscrire les initiales, l'âge et le sexe de la personne interrogée en première position/ligne du tableau. Les initiales, l'âge et le sexe de tous les autres membres du ménage doivent être inscrits ensuite. Les initiales d'un membre du ménage consistent en les trois premières lettres du prénom et les trois premières lettres du nom de famille (un éventuel deuxième nom ne fait pas partie des initiales). Inscrire l'âge de chaque membre du ménage avec un numéro à trois chiffres (unité : années) ; si l'âge d'un des membres du ménage ≤ 12 mois, inscrire 1 an comme âge. Merci de compléter un formulaire supplémentaire si le ménage a plus de vingt membres.

Membre du ménage Identifiant	Initiales [prénom/nom de famille]	Âge [années]	Sexe
1/Personne interrogée	____ ____ ____/____ ____ ____	____ ____ ____	O1=Masculin O2=Féminin
2	____ ____ ____/____ ____ ____	____ ____ ____	O1=Masculin O2=Féminin
3	____ ____ ____/____ ____ ____	____ ____ ____	O1=Masculin O2=Féminin
4	____ ____ ____/____ ____ ____	____ ____ ____	O1=Masculin O2=Féminin
5	____ ____ ____/____ ____ ____	____ ____ ____	O1=Masculin O2=Féminin
6	____ ____ ____/____ ____ ____	____ ____ ____	O1=Masculin O2=Féminin
7	____ ____ ____/____ ____ ____	____ ____ ____	O1=Masculin O2=Féminin
8	____ ____ ____/____ ____ ____	____ ____ ____	O1=Masculin O2=Féminin
9	____ ____ ____/____ ____ ____	____ ____ ____	O1=Masculin O2=Féminin
10	____ ____ ____/____ ____ ____	____ ____ ____	O1=Masculin O2=Féminin
11	____ ____ ____/____ ____ ____	____ ____ ____	O1=Masculin O2=Féminin
12	____ ____ ____/____ ____ ____	____ ____ ____	O1=Masculin O2=Féminin
13	____ ____ ____/____ ____ ____	____ ____ ____	O1=Masculin O2=Féminin
14	____ ____ ____/____ ____ ____	____ ____ ____	O1=Masculin O2=Féminin
15	____ ____ ____/____ ____ ____	____ ____ ____	O1=Masculin O2=Féminin
16	____ ____ ____/____ ____ ____	____ ____ ____	O1=Masculin O2=Féminin
17	____ ____ ____/____ ____ ____	____ ____ ____	O1=Masculin O2=Féminin
18	____ ____ ____/____ ____ ____	____ ____ ____	O1=Masculin O2=Féminin
19	____ ____ ____/____ ____ ____	____ ____ ____	O1=Masculin O2=Féminin
20	____ ____ ____/____ ____ ____	____ ____ ____	O1=Masculin O2=Féminin

For peer review only - http://bmjopen.bmj.com/site/about/guidelines.xhtml

FORMULAIRE 3: INFORMATIONS GÉNÉRALES SUR LE MÉNAGE
PARTIE B – DONNÉÉS SOCIO-ECONOMIQUES

ÉDUCATION

1 Le chef de ménage sait-il lire en [inscrire la langue officielle du site d'étude/pays] **?**
O 1=Oui O 2=Non O 99=Ne sait pas O 98=Pas de réponse

2 Le chef de ménage sait-il écrire en [inscrire la langue officielle du site d'étude/pays] **?**
O 1=Oui O 2=Non O 99=Ne sait pas O 98=Pas de réponse

3 Quel est le niveau d'éducation scolaire le plus élevé atteint par le chef de ménage ?
O 1=École primaire O 4=Plus élevé que le lycée O 99=Ne sait pas
O 2=Collège O 5= PAS D'ÉDUCATION O 98=Pas de réponse
O 3=Lycée O 6=Autre, préciser ___________

PROFESSION/RESSOURCES FINANCIÈRES

4 Quelle est la profession principale du chef de ménage ?
Instructions : Inscrire le statut du jour de visite uniquement.

O 1=Groupe 1 : Cadres
(directeurs généraux, hauts fonctionnaires, législateurs ; cadres administratifs et commerciaux ; responsables de production et de services spécialisés ; responsables dans l'hôtellerie, la vente et d'autres services)

O 2=Groupe 2 : Professionnels
(scientifiques ou ingénieurs ; professionnels de santé ; professionnels de l'enseignement ; professionnels de l'administration et des affaires ; professionnels de l'information et des technologies de communication ; professionnels du droit, du social ou de la culture)

O 3=Groupe 3 : Techniciens et professions intermédiaires
(Professions intermédiaires des sciences et techniques ; professions intermédiaires de la santé ; professions intermédiaires de l'administration et des affaires ; professions intermédiaires du droit, du social, de la culture et assimilés ; techniciens de l'information et des communications)

O 4=Groupe 4 : Employés de bureau
(secrétaires et dactylographes ; employé de service clientèle ; employé de conservation des données et de la documentation ; autres employés de bureau)

O 5=Groupe 5 : Travailleurs des services et de la vente
(travailleurs du service aux personnes ; travailleurs de la vente ; travailleurs des soins aux personnes ; travailleurs des services de protection)

O 6=Groupe 6 : Travailleurs qualifiés de l'agriculture, la sylviculture et la pêche
(travailleurs qualifiés de l'agriculture orientés marché ; éleveurs d'animaux; travailleurs qualifiés de la sylviculture, la pêche et la chasse orientés marché ; agriculteurs, pêcheurs, chasseurs et cueilleurs de subsistance)

O 7=Groupe 7 : Travailleurs manuels et assimilés
(travailleurs de la construction et assimilés (à l'exception des électriciens) ; travailleurs de la métallurgie, sur machines et assimilés ; artisans et travailleurs de l'imprimerie ; travailleurs des métiers de l'électrique et de l'électronique ; travailleurs des métiers de la transformation des aliments, du bois, des vêtements et assimilés)

O 8=Groupe 8 : Opérateurs de machines et d'installations, et monteurs
(Opérateurs de machines et d'installations fixes ; monteurs ; conducteurs opérateurs d'installations mobiles)

O 9=Groupe 9 : Emplois non qualifiés
(agents d'entretien et assistants ; ouvriers de l'agricultures, la sylviculture et la pêche ; travailleurs des mines, de la construction, de l'industrie et des transports ; assistants à la préparation des aliments ; vendeurs et services de rue et assimilés ; travailleurs de la collecte des ordures et autres travailleurs non qualifiés)

O 10=Groupe 0 : Professions des forces armées
(officiers des forces armées ; sous-officiers des forces armées ; professions des forces armées, autres grades)

O 11=Autre, préciser __________
O 12=PAS DE PROFESSION O 99=Ne sait pas O 98=Pas de réponse

For peer review only - http://bmjopen.bmj.com/site/about/guidelines.xhtml

5 Quel est le montant moyen des revenus mensuels du chef de ménage en lien avec à la profession indiquée en question 4 ainsi que celui des autres membres du ménage ?

Instruction: Tick one answer per household head/enter one code per household member if applicable/if income available.

Chef de ménage	Membre 1	Membre 2	Membre 3
Code ____________	Code ____________	Code ____________	Code ____________
[1] <100USD<br>[2] ≥100 à <150USD	[3] ≥150 à <200USD<br>[4] ≥200 à <250USD	[5] ≥250USD<br>[6] PAS DE REVENU (chef de ménage; voir Q4)	[99] Ne sait pas<br>[98] Pas de réponse

6 Quels sont les autres sources ou les sources supplémentaires de revenus mensuels du ménage et quel en est le montant ?

Instruction : Cocher plusieurs cases si nécessaire ; il peut y avoir plus d'une réponse.

Autres sources/Sources supplémentaires	Revenus financiers mensuels globaux
O 1=Aides sociales<br>O 2=Assurances (publiques ou privées)<br>O 3=Prêt (d'amis, de membres de la famille, d'une banque)<br>O 4=Vente (bétail, récoltes, meubles, autres biens)<br>O 5=Économies personnelles<br>O 6=Autre, préciser ________________	O 1=<100 USD<br>O 2=≥100 à <150 USD<br>O 3=≥150 à <200 USD<br>O 4=≥200 à <250 USD<br>O 5=≥250 USD
O 7=PAS D'AUTRES SOURCES/DE SOURCES SUPPLÉMENTAIRES<br>O 99=Ne sait pas<br>O 98=Pas de réponse	

LOGEMENT/HABITATION

7 Les membres du ménage sont-ils propriétaires de l'habitation ?

O 1=Oui
O 2=Non, locataires
O 3=Non, logement fourni (par le gouvernement, un employeur)
O 4=Non, autres, préciser _________
O 99=Ne sait pas
O 98=Pas de réponse

8 Combien de pièces ou structures individuelles de l'habitation sont utilisées pour dormir, vivre, faire la cuisine ou se laver ?

Instruction : N'indiquer qu'un code par catégorie.

Chambre	Salon	Cuisine	Salle de bain
Code ____________	Code ____________	Code ____________	Code ____________
[1] 1-2<br>[2] 3-4	[2] >4<br>[2] Aucune	[99] Ne sait pas<br>[98] Pas de réponse	

9 En quel matériau sont construits le sol, les murs et le toit des pièces/des structures individuelles de l'habitation ?

Instruction : Indiquer tous les codes par catégorie ; plus d'une réponse par catégorie est possible.

Sol	Murs	Toit
Code ____________	Code ____________	Code ____________

[1] Brique [3] Tuiles [5] Bois [7] Métal/Étain [99] Ne sait pas
[2] Ciment/Béton [4] Terre [6] Paille/feuilles [8] Autre, préciser_________ [98] Pas de réponse

10 Les membres de ce ménage ont-ils accès à/utilisent-ils l'électricité dans cette habitation ?

O 1=Oui O 2=Non O 99=Ne sait pas O 98=Pas de réponse

11 À quelle source de lumière principale les membres de ce ménage ont-ils accès/Quelle source de lumière principale utilisent-ils ?

O 1=Lumière/ampoule(s) électrique(s)
O 2=Lumière du soleil/Lampe(s) solaire(s)
O 3=Lampe(s) à pétrole/à huile
O 4=Lampe(s) à piles
O 5=Bougie(s)
O 6=PAS DE SOURCE DE LUMIÈRE
O 7= Autre, préciser_____
O 99=Ne sait pas
O 98=Pas de réponse

For peer review only - http://bmjopen.bmj.com/site/about/guidelines.xhtml

BIENS DU MÉNAGE

12 Parmi les articles suivants, lesquels les membres du ménage possèdent-ils ?

Instructions : Cocher plusieurs cases si nécessaire ; il peut y avoir plus d'une réponse ; indiquer la quantité d'articles fonctionnels si nécessaire

O 1=Radio	Quantité ____	O Rien	O 11=Lecteur cassettes/CD	Quantité ____	O Rien
O 2=Télévision	Quantité ____	O Rien	O 12=Ordinateur de bureau/portable/tablette	Quantité ____	O Rien
O 3=Connexion internet	Quantité ____	O Rien	O 13=Téléphone portable	Quantité ____	O Rien
O 4=Voiture	Quantité ____	O Rien	O 14=Camion/bus	Quantité ____	O Rien
O 5=Moto	Quantité ____	O Rien	O 15=Vélo	Quantité ____	O Rien
O 6=Char à bœufs/ânes	Quantité ____	O Rien	O 16=Lit	Quantité ____	O Rien
O 7=Matelas	Quantité ____	O Rien	O 17=Moustiquaire	Quantité ____	O Rien
O 8=Table	Quantité ____	O Rien	O 18=Chaise	Quantité ____	O Rien
O 9=Réfrigérateur (4°C)	Quantité ____	O Rien	O 19=Congélateur (-20°C)	Quantité ____	O Rien
O 10=Ventilateur	Quantité ____	O Rien	O 20=Machine à coudre	Quantité ____	O Rien

For peer review only

FORMULAIRE 3: INFORMATIONS GÉNÉRALES SUR LE MÉNAGE
Partie C – Hygiène et installations sanitaires

TOILETTES

1 À quel type de toilettes les membres du ménage ont-ils habituellement accès ?

Instruction : À l'intérieur signifie à l'intérieur d'une pièce/d'une habitation individuelle/d'un bâtiment ; à l'extérieur signifie à l'extérieur d'une pièce/d'une habitation individuelle/d'un bâtiment.

- O 1=Toilettes avec chasse d'eau/Cabinets (à l'intérieur)
- O 2=Toilettes avec chasse d'eau/Cabinets (à l'extérieur)
- O 3=Latrines sans chasse/latrines à fosse (à l'intérieur)
- O 4=Latrines sans chasse/latrines à fosse (à l'extérieur)
- O 5=Latrines à fosse améliorées par une ventilation (KVIP, à l'intérieur)
- O 6=Latrines à fosse améliorées par une ventilation (KVIP, à l'extérieur)
- O 7=Toilettes à compostage (à l'intérieur)
- O 8=Toilettes à compostage (à l'extérieur)
- O 9=PAS de toilettes, utilisent un seau
- O 10=PAS de toilettes, creusent un trou
- O 11=PAS de toilettes, défécation libre/en plein air
- O 12=Autre, préciser __________
- O 99=Ne sait pas
- O 98=Pas de réponse

2 Les membres du ménage utilisent-ils habituellement les toilettes indiquées à la question 1 ?

- O 1=Non, les membres ne peuvent utiliser les toilettes de façon autonome (bébés, enfants en bas-âge)
- O 2=Non, les membres ne peuvent utiliser les toilettes de façon autonome (handicapés, malades chroniques/longue durée)
- O 3=Non, les membres ne peuvent utiliser les toilettes de façon autonome (personnes âgées)
- O 4=Non, accès limité (publiques, partagées avec d'autres ménages/communautés)
- O 5=Non, accès limité (publiques, partagées avec d'autres ménages/communautés)
- O 6= Non, mauvais état (cassé, pas propre)
- O 7=OUI
- O 8=Autre, préciser _________________
- O 99=Ne sait pas
- O 98=Pas de réponse

3 Où sont situées les toilettes indiquées à la question 1 ? Par combien de personnes sont-elles habituellement partagées ?

- O 1=Sur la propriété du ménage (privées, non partagées)
- O 2=Sur la propriété d'un ménage voisin (privées, partagées)
  - *Partagées par* O 1=<10 personnes O 2=11-24 personnes O 3=25-49 personnes O 4=>50 personnes O 99=Ne sait pas O 98=Pas de réponse
- O 3=Dans la communauté (publiques, partagées)
  - *Partagées par* O 1=<10 personnes O 2=11-24 personnes O 3=25-49 personnes O 4=>50 personnes O 99=Ne sait pas O 98=Pas de réponse
- O 4=Autre, préciser__________ O 99=Ne sait pas O 98=Pas de réponse

4 Comment les membres du ménage qui ont accès/n'ont pas accès aux toilettes se lavent-ils habituellement les mains tout de suite avant et après avoir uriné/déféqué ?

Instruction : Indiquer un code par catégorie uniquement.

Lavent les mains avant d'avoir uriné/déféqué	Lavent les mains après avoir uriné/déféqué
Code _____________	Code _____________
[1] Lavent les mains avec du savon et de l'eau<br>[2] Lavent les mains avec de l'eau uniquement<br>[3] Frottent les mains avec des feuilles/de la paille/de l'herbe<br>[4] Frottent les mains avec du sable<br>[5] Frottent les mains avec un chiffon/du tissu	[6] Ne lavent pas, pas besoin/pas sale<br>[7] Ne lavent pas, rien pour laver les mains<br>[8] Autre, préciser __________<br>[99] Ne sait pas<br>[98] Pas de réponse

CUISINE/MANIPULATION DES ALIMENTS

5 Les membres de ce ménage ont-ils une cuisine pour faire cuire/préparer la nourriture/les repas ? Où est-elle située ?

- O 1=Oui, cuisine intérieure/dans une pièce/structure individuelle d'habitation ; sur la propriété du ménage (privée, non partagée)
- O 2=Oui, cuisine extérieure/hors d'une pièce/structure individuelle d'habitation/ en plein air ; sur la propriété du ménage (privée, non partagée)

For peer review only - http://bmjopen.bmj.com/site/about/guidelines.xhtml

- O 3=Oui, cuisine intérieure/dans une pièce/structure individuelle d'habitation ; sur la propriété d'un ménage voisin (privée, partagée)
- O 4=Oui, cuisine extérieure/hors d'une pièce/structure individuelle d'habitation/ en plein air ; sur la propriété d'un ménage voisin (privée, partagée)
- O 5=PAS DE CUISINE
- O 6=Autre, préciser __________ O 99=Ne sait pas O 98=Pas de réponse

6 Quelle source d'énergie les membres de ce ménage utilisent-ils habituellement pour cuisiner/préparer la nourriture/les repas ?

O 1=Électricité O 3=Charbon de bois O 5=Excréments d'animaux O 99=Ne sait pas
O 2=Gaz/pétrole O 4=Bois/paille/feuilles O 6=Autre, préciser __________ O 98=Pas de réponse

7 Quelle moyen de cuisson les membres de ce ménage utilisent-ils habituellement pour cuisiner/préparer la nourriture/les repas ?

O 1=Foyer ouvert O 3=Fourneau intérieur avec cheminée O 99=Ne sait pas
O 2=Fourneau extérieur O 4=Autre, préciser __________ O 98=Pas de réponse

8 Où les membres de ménage conservent-ils habituellement les produits alimentaires frais/crus (comme les légumes, la salade, les fruits, les produits laitiers, les œufs, la viande et le poisson) et les restes/les aliments/repas préparés ?

Instruction : Indiquer un code par catégorie uniquement. À l'intérieur signifie à l'intérieur d'une pièce/d'une habitation individuelle/d'un bâtiment ; à l'extérieur signifie à l'extérieur d'une pièce/d'une habitation individuelle/d'un bâtiment.

Des produits alimentaires frais/crus (légumes, salade, fruits, produits laitiers, œufs, viande et poisson)	Des restes/aliments/repas préparés
Code _____________	Code _____________

[1] À température ambiante (à l'intérieur, sur la propriété du ménage, privé)
[2] À température ambiante (à l'extérieur, sur la propriété du ménage, privé)
[3] À température ambiante (à l'intérieur, sur la propriété d'un ménage voisin, privé, partagé)
[4] À température ambiante (à l'extérieur, sur la propriété d'un ménage voisin, privé, partagé)
[5] Réfrigérateur (sur la propriété du ménage, privé)
[6] Réfrigérateur (sur la propriété d'un ménage voisin, privé, partagé)
[7] Congélateur (sur la propriété du ménage, privé)
[8] Congélateur (sur la propriété d'un ménage voisin, privé, partagé)
[9] Autre, préciser ______________ [10] PAS DE CONSERVATION [99] Ne sait pas [98] Pas de réponse

9 Si la réponse à la question 8 au sujet des produits alimentaires frais/crus et/ou des restes/aliments/repas préparés est «température ambiante», combien de temps les membres de ce ménage les conservent-ils habituellement?

Instruction: Indiquer un code par catégorie uniquement.

Des produits alimentaires frais/crus (légumes, salade, fruits, produits laitiers, œufs, viande et poisson)	Des restes/aliments/repas préparés
Code _____________	Code _____________

[1] <1 jour [3] 3-4 jours [5] Pas applicable [99] Ne sait pas
[2] 1-2 jours [4] >4 jours [98] Pas de réponse

10 Les membres de ce ménage couvrent-ils habituellement les restes/aliments/repas préparés? Réchauffent-ils/recuisent-ils les restes/aliments/repas préparés avant de les consommer?

Instruction : Indiquer un code par catégorie uniquement.

Couvrent les restes/les aliments/repas préparés	Réchauffent/Recuisent les restes/les aliments/repas préparés
Code _____________	Code _____________

[1] Oui [2] Non [99] Ne sait pas [98] Pas de réponse

For peer review only - http://bmjopen.bmj.com/site/about/guidelines.xhtml

11 Quel membre(s) de ce ménage cuisine(s)/prépare(s) la nourriture/les repas habituellement pour les autres membres de ce ménage ?

Instructions : Se référer à la question 9 du « Formulaire 3 Partie A – Données démographiques/Informations générales sur le ménage » et indiquer les initiales/ Identifiant (s) du membre.

O Membre du ménage, indiquer les initiales/identificateur(s) __________

O 99=Ne sait pas O 98=Pas de réponse

12 Comment le(s) membre(s) de la famille qui cuisine(nt)/prépare(nt) la nourriture/les repas habituellement pour les autres membres de ce ménage comme indiqué à la question 11 se prépare(nt)-t-il(s)/elle(s) habituellement avant et après la préparation des repas?

Instruction: Indiquer un code par catégorie uniquement.

Avant la préparation des repas	Après la préparation des repas
Code _____________	Code _____________
[1] Lavent les mains avec du savon et de l'eau<br>[2] Lavent les mains avec de l'eau uniquement<br>[3] Frottent les mains avec des feuilles/de la paille/de l'herbe<br>[4] Frottent les mains avec du sable<br>[5] Frottent les mains avec un chiffon/du tissu	[6] Ne lavent pas, pas besoin/pas sale<br>[7] Ne lavent pas, rien pour laver les mains<br>[8] Autre, préciser __________<br>[99] Ne sait pas<br>[98] Pas de réponse

13 Comment les membres de ce ménage se préparent-ils habituellement avant et après avoir mangé de la nourriture/des repas, y compris le membre du ménage qui cuisine/prépare la nourriture/les repas habituellement comme indiqué à la question 11?

Instruction : Indiquer un code par catégorie uniquement.

Avant la préparation des repas	Après la préparation des repas
Code _____________	Code _____________
[1] Lavent les mains avec du savon et de l'eau<br>[2] Lavent les mains avec de l'eau uniquement<br>[3] Frottent les mains avec des feuilles/de la paille/de l'herbe<br>[4] Frottent les mains avec du sable<br>[5] Frottent les mains avec un chiffon/du tissu	[6] Ne lavent pas, pas besoin/pas sale<br>[7] Ne lavent pas, rien pour laver les mains<br>[8] Autre, préciser __________<br>[99] Ne sait pas<br>[98] Pas de réponse

14 Comment les membres de ce ménage mangent-ils habituellement leur nourriture/leurs repas, y compris le membre du ménage qui cuisine/prépare la nourriture/les repas habituellement comme indiqué à la question 11?

O 1=Avec des couverts O 3=Autre, préciser __________ O 99=Ne sait pas

O 2=Avec les doints O 98=Pas de réponse

15 Les membres de ce ménage mangent-ils habituellement leur nourriture/leurs repas dans le même récipient, y compris le membre du ménage qui cuisine/prépare la nourriture/les repas habituellement comme indiqué à la question 11?

O 1=Oui O 2=Non O 99=Ne sait pas O 98=Pas de réponse

16 Où les membres de ce ménage obtiennent-ils habituellement leurs produits alimentaires frais/crus principaux comme les légumes, les fruits, les grains/céréales, les produits laitiers, les œufs, la viande et le poisson?

Instruction : Indiquer un code par catégorie uniquement.

Légumes, fruits, grains/céréales	Produits laitiers	Œufs, viande, poisson
Code _____________	Code _____________	Code _____________

[1] Agriculture vivrière (cour/arrière-cour)<br>[2] Supermarché/Épicerie<br>[3] Amis/famille<br>[4] Marché ouvert<br>[5] Vendeur de rue	[6] Agriculture vivrière (terre agricole)<br>[7] Boucher<br>[8] Autre, préciser _______<br>[99] Ne sait pas<br>[98] Pas de réponse

For peer review only - http://bmjopen.bmj.com/site/about/guidelines.xhtml

17 Les membres de ce ménage consomment/mangent-ils des produits alimentaires frais/crus (comme les légumes, la salade, les fruits, les produits laitiers, la glace, les œufs, la viande et le poisson) ? Si oui, lesquels?

Instruction : Cocher plusieurs cases si nécessaire ; plus d'une réponse est possible.

O 1=Légumes/salade (cultivés sur arbuste)
O 2=Légumes/salade (récolte sauvage sur arbuste)
O 3=Fruits (cultivés sur arbuste/arbre)
O 4=Fruits (récolte sauvage sur arbuste/arbre)
O 5=Produits laitiers (lait, fromage, yaourt d'animaux domestiques produisant du lait), préciser ____________
O 6=Œufs (de volailles domestiques), préciser _________
O 7=Sang animal (d'animaux domestiques), domestiques), préciser _________
O 8=Bœuf/Zébu/Buffle (domestiqué), préciser _________
O 9=Porc (domestiqué)
O 10=Mouton (domestiqué)
O 11=Viande d'animaux non-domestiqués (antilope, sanglier, volaille, rongeurs, poisson), préciser ________
O 12=PAS DE CONSOMMATION de produits frais/crus
O 13=Légumes/salade (cultivés au sol)
O 14=Légumes/salade (récolte sauvage sur le sol)
O 15=Fruits (cultivés au sol)
O 16=Fruits (récolte sauvage sur le sol)
O 17=Produits laitiers (lait, fromage, yaourt d'animaux non-domestiques produisant du lait), préciser ____________
O 18= Œufs (de volailles non-domestiques), préciser _________
O 19= Sang animal (d'animaux non-préciser _________
O 20=Volaille (domestiquée; poulet, canard, pigeon, oie, dinde), préciser _________
O 21=Chèvre (domestiquée)
O 22= Rongeurs (domestiqués), préciser ______
O 23=Poisson/fruits de mer, préciser _____
O 24=Glace
O 25=Autre, préciser _________
O 99=Ne sait pas O 98=Pas de réponse

18 Les membres de ce ménage consomment-ils habituellement des repas/produits alimentaires/boissons également en dehors de l'habitation ? Si oui, quel(s) membre(s) de ce ménage et où ?

Instructions : Se référer à la question 9 du « Formulaire 3 Partie A – Données démographiques/Informations générales sur le ménage » et indiquer les initiales du/des membre(s). Indiquer tous les codes nécessaires pour les membres du ménage respectifs.

Membre 1<br>Initiales/Identifiant : ___	Membre 2<br>Initiales/Identifiant : ___	Membre 3<br>Initiales/Identifiant : ___	Membre 4<br>Initiales/ Identifiant : ___
Code ___________	Code ___________	Code ___________	Code ___________
[1] Restaurant<br>[2] Chaîne de restauration rapide	[3] Cafétéria<br>[4] Vendeur de rue	[5] Autre, préciser ____<br>[6] Pas applicable	[99] Ne sait pas<br>[98] Pas de réponse

19 Quels/Quelles repas/produits alimentaires/boissons le/les membre(s) de ce ménage listé à la question 18 consomment-ils habituellement en dehors de l'habitation ?

Instructions : Se référer à la question 9 du « Formulaire 3 Partie A – Données démographiques/Informations générales sur le ménage » et indiquer/identifiant les initiales du/des membre(s). Indiquer jusqu'à 5 repas/produits alimentaires/boissons par membre du ménage. Vous pouvez vous référer aux produits alimentaires listés à la question 17. Passer cette question si aucun membre du ménage ne consomme les repas/les produits alimentaires/les boissons en dehord de la maison comme indiqué dans Q18.

Membre 1<br>Initiales/Identifiant : ____	Membre 2<br>Initiales/Identifiant : ____	Membre 3<br>Initiales/Identifiant : ____	Membre 4<br>Initiales/ Identifiant : ___
1: ____________<br>2: ____________<br>3: ____________<br>4: ____________<br>5: ____________	1: ____________<br>2: ____________<br>3: ____________<br>4: ____________<br>5: ____________	1: ____________<br>2: ____________<br>3: ____________<br>4: ____________<br>5: ____________	1: ____________<br>2: ____________<br>3: ____________<br>4: ____________<br>5: ____________

EAU

20 Quelle est la source d'approvisionnement en eau principale des membres de ce ménage pour la boisson, la cuisine/la préparation des aliments/repas, le bain et le ménage (de l'habitation/de la/les structure(s) individuelle(s) d'habitation(s), les vêtements/les outils de cuisine) ?

For peer review only - http://bmjopen.bmj.com/site/about/guidelines.xhtml

Instruction : Indiquer un code par catégorie uniquement.

Boisson	Cuisine	Bain	Nettoyage
Code _____________	Code _____________	Code _____________	Code _____________

[1] Eau du robinet (à l'intérieur de l'habitation ; privée)
[2] Eau du robinet (à l'extérieur de l'habitation : cour/arrière-cour ; privée)
[3] Eau du robinet (à l'extérieur de l'habitation ; publique/partagée)
[4] Borne-fontaine/eau courante (à l'intérieur de l'habitation ; privée)
[5] Borne-fontaine/eau courante (à l'extérieur de l'habitation : cour/arrière-cour ; privée)
[6] Borne-fontaine/eau courante (à l'extérieur de l'habitation ; publique/partagée)
[7] Camion-citerne (public ; partagé)
[8] Eaux de surface (canal, canal d'irrigation)
[9] Eaux de surface (lac, étang, rivière, ruisseau)
[10] Eau de source
[11] Eau de pluie
[12] Puits/Puits de forage (en plein air/non couvert/non protégé)
[13] Puits/Puits de forage (couvert/protégé)
[14] Eau en bouteille
[15] Autre, préciser ____________________ [99] Ne sait pas [98] Pas de réponse

21 Les membres de ce ménage traitent-ils habituellement l'eau pour la boisson, la cuisine/la préparation des aliments/des repas, le bain, et le nettoyage (de l'habitation/des/de la structure(s) individuelle(s) d'habitation(s), des vêtements/des outils de cuisine) avant la consommation ? Comment l'eau est-elle habituellement traitée ?

Instruction: Indiquer tous les codes nécessaires par catégorie ; plus d'une réponse par catégorie est possible.

Boisson	Cuisine	Bain	Nettoyage
Code _____________	Code _____________	Code _____________	Code _____________

[1] Non, pas du tout
[2] Non, seulement pour les malades du ménage
[3] Non, seulement selon la saison (saison sèche)
[4] Oui, par filtration (tissu, céramique, sable)
[5] Oui, par sédimentation
[6] Autre, préciser ________________
[7] Non, seulement pour les enfants du ménage
[8] Non, seulement selon la saison (saison humide)
[9] Oui, par ébullition
[10] Oui, par désinfection solaire
[11] Oui, par chloration
[99] Ne sait pas [98] Pas de réponse

22 Quel(s) membre(s) de ce ménage recueille(nt) habituellement l'eau potable comme indiqué à la question 20 pour les autres membres de ce ménage ?

Instructions : Se référer à la question 9 du « Formulaire 3 Partie A – Données démographiques/Informations générales sur le ménage » et indiquer/identifiant les initiales du membre.

O Membre du ménage, indiquer les initiales/identifiant(s) _________
O 99=Ne sait pas O 98=Pas de réponse

23 Comment les membres de ce ménage conservent-ils habituellement l'eau potable indiquée à la question 20?

O 1=Réservoir d'eau (non couvert)
O 2=Réservoir d'eau (couvert par chiffon/tissu, couvercle, feuilles)
O 3=Récipient (non couvert)
O 4=Récipient (couvert par chiffon/tissu, couvercle, feuilles)
O 5=Pot en terre (non couvert)
O 6=Pot en terre (couvert par chiffon/tissu, couvercle, feuilles)
O 7=Seau (non couvert)
O 8=Seau (couvert par chiffon/tissu, couvercle, feuilles)
O 9=Bol (non couvert)
O 10=Bol (couvert par chiffon/tissu, couvercle, feuilles)
O 11=Pot en bois (non couvert)
O 12= Pot en bois (couvert par chiffon/ tissu, couvercle, feuilles)
O 13= Récipient en peau (chèvre, mouton, bovins, non couvert)
O 14=Récipient en peau (chèvre, mouton, bovins ; couvert par un chiffon/du tissu, un couvercle, des feuilles
O 15=NON CONSERVÉE
O 16=Autre, préciser _________
O 99=Ne sait pas
O 98= Pas de réponse

For peer review only - http://bmjopen.bmj.com/site/about/guidelines.xhtml

24 Si les membres de ce ménage utilisent une source d'approvisionnement en eau potable partagée comme indiqué à la question 20, par combien de personnes cette eau potable est-elle partagée ?

O 1=<10 personnes   O 3=25-49 personnes   O 5=NON PARTAGÉE   O 99=Ne sait pas
O 2=11-24 personnes   O 4=>50 personnes   O 98=Pas de réponse

TRAITEMENT DES DÉCHETS

25 Où les membres de ce ménage jettent-ils habituellement les eaux usées de, par exemple, la cuisine/la préparation des aliments/repas, le bain et/ou le ménage, et jettent-ils habituellement les déchets domestiques, les déchets humains/déjections ?

Instruction: Indiquer un code par catégorie uniquement.

Eaux usées de par exemple cuisine/préparation des aliments/ repas, bain et/ou ménage	Déchets domestiques	Déchets humains/déjections
Code ____________	Code ____________	Code ____________

[1] Sur le sol/surface libre proche de l'habitation (cour/arrière-cour, privé)
[2] Sur le sol/surface libre loin de l'habitation dans la communauté (partagé, public)
[3] Dans un trou proche de la maison (cour/arrière-cour, privé)
[4] Dans un trou loin de la maison dans la communauté (partagé, public)
[5] Versement dans des eaux de surface dans la communauté (lac, étang, rivière, ruisseau, public, partagé)
[6] Versement dans des eaux de surface dans la communauté (canal, canal d'irrigation, public, partagé)
[7] Versement dans de l'eau de source dans la communauté (public, partagé)
[8] Versement dans une fosse septique proche de l'habitation (cour/arrière-cour, proche de toilettes si disponibles, privé)
[9] Versement dans une fosse septique dans la communauté (partagé, public)
[10] Versement dans les égouts proches de la maison (cour/arrière-cour, privé)
[11] Versement dans les égouts dans la communauté (partagé, public)
[12] Autre, préciser __________________________ [99] Ne sait pas [98] Pas de réponse

26 Les membres de ce ménage traitent-ils habituellement les déchets domestiques de la maison au préalable avant de les jeter ?

O 1=Oui, incinération des déchets
O 2=Oui, séparation/tri des déchet dégradable et non dégradables
O 3=NON
O 4=Oui, Autre, préciser _________
O 99=Ne sait pas   O 98=Pas de réponse

27 À quelle distance de la source d'approvisionnement en eau de ce ménage comme indiqué à la question 20 se trouve l'emplacement de l'élimination des eaux usées, des déchets domestiques et humains/excrétions de ce ménage comme indiqué à la question 25 ?

Instructions : Indiquer un code par catégorie uniquement. Indiquer la distance en mètres [m].

Eaux usées de, par exemple, cuisine/préparation des aliments/ repas, bain et/ou ménage	Déchets domestiques	Déchets humains/déjections
Code ____________	Code ____________	Code ____________

[1] <5 m   [3] ≥25 à <50 m   [5] ≥100 à <500 m   [7] ≥1,000 m
[2] ≥5 à <25 m   [4] ≥50 à <100 m   [6] ≥500 à <1,000 m   [99] Ne sait pas [98] Pas de réponse

28 À quelle distance de la source d'approvisionnement en eau principale de ce ménage pour la cuisine/la préparation des aliments/des repas comme indiqué à la question 20 se trouve l'emplacement de l'élimination des eaux usées, des déchets domestiques et humains/excrétions de ce ménage comme indiqué à la question 25 ?

Instructions : Indiquer un code par catégorie uniquement. Indiquer la distance en mètres [m].

Eaux usées de par exemple cuisine/préparation des aliments/ repas, bain et/ou ménage	Déchets domestiques	Déchets humains/excréments
Code ____________	Code ____________	Code ____________

For peer review only - http://bmjopen.bmj.com/site/about/guidelines.xhtml

[1] <5 m	[3] ≥25 à <50 m	[5] ≥100 à <500 m	[7] ≥1,000 m
[2] ≥5 à <25 m	[4] ≥50 à <100 m	[6] ≥500 à <1,000 m	[99] Ne sait pas [98] Pas de réponse

ANIMAUX DE FERME/DOMESTIQUES

29 Quel genre d'animaux/de bétail et d'animaux domestiques/de compagnie les membres de ce ménage possèdent-ils ?

Instruction : Cocher plusieurs cases par catégorie si nécessaire ; il peut y avoir plus d'une réponse par catégorie.

Animaux de ferme/bétail		Animaux domestiques/de compagnie
O 1=Bovin	O9=Chameau	O 1=Chien
O 2=Mouton	O 10=Rongeurs (lapin), préciser ____________	O 2=Rongeurs (lapin), préciser ____________
O 3=Âne	O 11=Chèvre	O 3=Chat
O 4= Volaille (i.e. poulet, canard, pigeon, oie, dinde), préciser ____________	O 12=Cheval	O 4=PAS D'ANIMAUX DOMESTIQUES/DE COMPAGNIE
O 5=Zébu	O 13=PAS D'ANIMAUX DE FERME/DE BÉTAIL	O 5=Autre, préciser ____________
O 6=Cochon	O 14=Autre, préciser ____________	O 99=Ne sait pas
O 7=Poisson	O 99=Ne sait pas	O 98=Pas de réponse
O 8=Buffle	O 98=Pas de réponse	

30 Où les membres de ce ménage gardent-ils habituellement les animaux de ferme/le bétail et les animaux domestiques/de compagnie de ce ménage comme indiqué à la question 29 ?

Instruction : Indiquer un code par catégorie uniquement.

Animaux de ferme/bétail	Animaux domestiques/de compagnie
O 1=Étable/Cage/Bassin d'eau sur la propriété de l'habitation (arrière-cour/cour)<br>O 2=Étable/Cage/Bassin d'eau à l'extérieur de l'habitation dans la communauté<br>O 3=Étable/Cage/Bassin d'eau à l'extérieur de la communauté<br>O 4=Surface libre/Eaux de surface/Eau de source sur la propriété de l'habitation (habitation, arrière-cour/cour)<br>O 5=Surface libre/Eaux de surface/Eau de source à l'extérieur de l'habitation dans la communauté<br>O 6=Surface libre/Eaux de surface/Eau de source à l'extérieur de la communauté<br>O 7=Pas applicable (nulle part /pas d'animaux de ferme/bétail)<br>O 8=Autre, préciser ____________<br>O 99=Ne sait pas<br>O 98=Pas de réponse	O 1=Cage/Chaîne proche de l'habitation (arrière-cour/cour)<br>O 2= Cage/Chaîne à l'extérieur de l'habitation dans la communauté<br>O 3= Cage/Chaîne à l'extérieur de la communauté (public)<br>O 4=Surface libre proche de l'habitation (dans la maison, arrière-cour/cour)<br>O 5=Surface libre à l'extérieur de l'habitation dans la communauté<br>O 6=Surface libre à l'extérieur de la communauté<br>O 7=Pas applicable (nulle part/pas d'animaux domestiques/de compagnie)<br>O 8=Autre, préciser ____________<br>O 99=Ne sait pas<br>O 98=Pas de réponse

31 Quelle est l'importance de l'élevage que les membres de ce ménage pratiquent ?

O 1=Pour leurs propres besoins («élevage d'arrière-cour»)
O 2=Pas applicable (pas d'élevage)
O 3=3=Demande massive/industrielle («élevage intensif»)
O 4=Autre, préciser ____________
O 99=Ne sait pas
O 98=Pas de réponse

For peer review only - http://bmjopen.bmj.com/site/about/guidelines.xhtml

32 Quel approvisionnement en eau les membres de ce ménage utilisent-ils pour fournir de l'eau aux animaux de ferme/au bétail et aux animaux domestiques/de compagnie de ce ménage comme indiqué à la question 29 ?

Instruction : Indiquer un code par catégorie uniquement.

Animaux de ferme/bétail	Animaux domestiques/de compagnie
Code ____________	Code ____________

[1] Eau du robinet (à l'intérieur de l'habitation ; privée)
[2] Eau du robinet (à l'extérieur de l'habitation : cour/arrière-cour ; privée)
[3] Eau du robinet (à l'extérieur de l'habitation ; publique/partagée)
[4] Borne-fontaine/eau courante (à l'intérieur de l'habitation ; privée)
[5] Borne-fontaine/eau courante (à l'extérieur de l'habitation : cour/arrière-cour ; privée)
[6] Borne-fontaine/eau courante (à l'extérieur de l'habitation ; publique/partagée)
[7] Camion-citerne (public ; partagé)
[8] Eaux de surface (canal, canal d'irrigation)
[9] Eaux de surface (lac, étang, rivière, ruisseau)
[10] Eau de source
[11] Eau de pluie
[12] Puits/Puits de forage (en plein air/non couvert/non protégé)
[13] Puits/Puits de forage (couvert/protégé)
[14] Pas applicable (pas d'animaux de ferme/bétail; pas d'animaux domestiques/de compagnie)
[15] Autre, préciser ____________________ [99] Ne sait pas [98] Pas de réponse

33 Les membres de ce ménage se lavent-ils habituellement les mains après avoir nourri, donné de l'eau et nettoyé l'étable/la cage/le bassin d'eau des animaux de ferme/du bétail de ce ménage comme indiqué à la question 29 ?

Instruction : Indiquer un code par catégorie uniquement.

Animaux de ferme/bétail	Animaux domestiques/de compagnie
Code ____________	Code ____________

[1] Lavent les mains avec du savon et de l'eau
[2] Lavent les mains avec de l'eau uniquement
[3] Frottent les mains avec des feuilles/de la paille/de l'herbe
[4] Frottent les mains avec du sable
[5] Frottent les mains avec un chiffon/du tissu
[6] Ne lavent pas, pas besoin/pas sale
[7] Ne lavent pas, rien pour laver les mains
[8] Autre, préciser _________
[9] Pas applicable (pas d'animaux de ferme/bétail ; pas d'animaux domestiques/de compagnie
[99] Ne sait pas [98] Pas de réponse

CULTURES AGRICOLES

34 Quel genre de cultures les membres de ce ménage font-ils pousser/cultivent-ils ?

Instruction : Cocher plusieurs cases si nécessaire ; il peut y avoir plus d'une réponse.

O 1=Café
O 2=Riz
O 3=Fruits (cultivés sur arbustes/arbre)
O 4=Fruits (cultivés au sol)
O 5=Légumes/salade (cultivés sur arbustes/arbre)
O 6=Légumes/salade (cultivés au sol)
O 7=Céréales (blé, orge, avoine, maïs)
O 8=Thé
O 9=Noix de cajou
O 10=Tabac
O 11=Canne à Sucre
O 12=Chad/Chat
O 13=Bees/Honey
O 14=Coton
O 15=PAS DE CULTURES
O 16=Autre, préciser _________
O 99=Ne sait pas
O 98=Pas de réponse

35 Où les champs/terres agricoles des membres de ce ménage se situent-ils/elles?

O 1=On the property of the house (i.e. back-/courtyard)
O 2=Outside the community
O 3=Other, specify _________
O 4=Outside the house in the community
O 5=Not applicable (no crop farming)
O 99=Don't know
O 98=No response

For peer review only - http://bmjopen.bmj.com/site/about/guidelines.xhtml

36 Quelle est l'importance des cultures des membres de ce ménage?

- O 1=Leurs propres besoins (« agriculture d'arrière-cour »)
- O 2=2=Pas applicable (pas de cultures)
- O 3=Demande massive/industrielle (agriculture intensive)
- O 4=Autre, préciser __________
- O 99=Ne sait pas
- O 98= Pas de réponse

37 Les membres de ce ménage utilisent-ils habituellement des herbicides, pesticides et engrais pour les cultures indiquées à la question 34?

Instruction : Indiquer un code par catégorie uniquement.

Herbicides	Pesticides	Engrais
O 1=Oui, préciser l'herbicide ______________	O 1=Oui, préciser le pesticide ______________	O 1=Oui, préciser l'engrais ______________
O 2=Pas applicable (pas de cultures)	O 2=Pas applicable (pas de cultures)	O 2=Pas applicable (pas de cultures)
O 3=Pas d'utilisation des herbicides	O 3=Pas d'utilisation des pesticides	O 3=Pas d'utilisation des engrais
O 99=Ne sait pas	O 99=Ne sait pas	O 99=Ne sait pas
O 98=Pas de réponse	O 98=Pas de réponse	O 98=Pas de réponse

38 Les membres de ce ménage se lavent-ils les mains après avoir travaillé dans les terres agricoles pour les cultures indiquées à la question 34 ?

- O 1=Oui, se lavent les mains avec du savon et de l'eau
- O 2=Oui, se frottent les mains avec des feuilles/de la paille/de l'herbe
- O 3=Oui, se frottent les mains avec des chiffons/du tissu
- O 4=Pas applicable (pas de cultures)
- O 5=Oui, se lavent les mains avec de l'eau uniquement
- O 6=Oui, se frottent les mains avec du sable
- O 7=Non, ne se lavent pas les mains
- O 8=Autre, préciser __________
- O 99=Ne sait pas
- O 98= Pas de réponse

For peer review only

For peer review only - http://bmjopen.bmj.com/site/about/guidelines.xhtml

Site ___ ___ ___ Sous-zone ___ ___ ___ Numéro d'habitation ___ ___ ___ ___ Identifiant de l'enquêteur ___ __

FORMULAIRE 4: ÉVALUATION DE L'UTILISATION GÉNÉRIQUE DES SOINS DE SANTÉ GLOBAUX, STRATIFIÉE SELON L'ÂGE ET LE SEXE

Partie A –Évaluation de l'utilisation *générique* des soins de santé

Instructions pour l'enquêteur

- Compléter ce formulaire uniquement si la personne interrogée a consenti à participer (voir « Formulaire 2 — FORMULAIRE DE CONSENTEMENT ») ; la personne interrogée ainsi que définie pour cette enquête est un adulte (selon l'âge légal de majorité du pays), un membre du ménage et un décideur en ce qui concerne les soins de santé quotidiens et l'utilisation de soins de santé pour le ménage entier et ses membres ; il ne doit pas être, par exemple, un voisin ou un visiteur.
- Se référer à « Formulaire 3 — Partie A — Informations générales sur le ménage » comme référence pour obtenir le nombre total de membres du ménage ainsi que l'âge et le sexe de chaque membre du ménage.
- Recueillir les données de tous les membres du ménage que tous les membres du ménage soient présents ou non au moment de l'entretien ; ne recueillir et n'inscrire les données que des personnes présentes au moment de l'entretien fausserait les données.
- Un ménage est défini/les membres d'un ménage sont définis comme une personne ou un groupe de personnes avec ou sans lien de parenté qui vivent ensemble dans la même unité d'habitation, qui reconnaissent un adulte de sexe masculin ou féminin comme chef de ménage, qui partagent les mêmes installations domestiques, qui sont considérés comme constituant une unité, et qui subviennent à leurs besoins en termes de nourriture et des autres éléments essentiels pour vivre.
- « Site » et « sous-zone » doivent être un numéro à trois chiffres, « numéro d'habitation » un numéro à quatre chiffres, et « identifiant de l'enquêteur » un numéro à deux chiffres (utiliser les listes préparées individuellement pour chaque site pour obtenir les codes) ; saisir « date » au format JJ/MM/AAAA et « heure » au format HH:MM et entourer MATIN ou APRÈS-MIDI. «Étiquette de l'étude» doit être une combinaison d'un code «site» à trois chiffres, d'un code «sous-zone» à trois chiffres et d'un code «numéro d'habitation» à quatre chiffres; le « identifiant de l'enquêteur » doit être un numéro à trois chiffres.
- Abréviation : EDS = Établissement de santé public/privé primaire/secondaire/tertiaire.

MEMBRES DU MÉNAGE

1 Indiquer le nombre total de membres du ménage de cette habitation (au jour de la visite).
Instructions : Le nombre total de membres du ménage doit être indiqué à l'aide d'un numéro à 2 chiffres ; voir également « Formulaire 3 — Partie A — INFORMATIONS GÉNÉRALES SUR LE MÉNAGE ».

Nombre total de membres du ménage : ____ ____

2 Indiquer le nombre de membres FÉMININS du ménage pour chacun des groupes d'âge suivants :
Instructions : Le nombre total de membres féminins du ménage par groupe d'âge doit être indiqué à l'aide d'un numéro à 2 chiffres ; voir également « Formulaire 3 — Partie A — INFORMATIONS GÉNÉRALES SUR LE MÉNAGE ».

<2 ans : ____ ____ ≥2 à <5 ans : ____ ____ ≥5 à <15 ans : ____ ____ ≥15 ans : ____ ____

3 Indiquer le nombre de membres MASCULINS du ménage pour chacun des groupes d'âge suivants :
Instructions : Le nombre total de membres masculins du ménage par groupe d'âge doit être indiqué à l'aide d'un numéro à 2 chiffres ; voir également « Formulaire 3 — Partie A — INFORMATIONS GÉNÉRALES SUR LE MÉNAGE ».

<2 ans : ____ ____ ≥2 à <5 ans : ____ ____ ≥5 à <15 ans : ____ ____ ≥15 ans : ____ ____

COMPORTEMENT DE RECHERCHE DE SOINS DE SANTÉ

4 Indiquer où les membres féminins/masculins du foyer d'âges <2 ans/≥2 à <5 ans/≥5 à <15 ans/≥15 ans recherchent habituellement des soins de santé pour les signes/symptômes suivants. Merci d'attribuer une note entre 1 et 4.

Instructions :
- Compléter la question 4 séparément pour les membres du ménage féminins et masculins d'âge <2 ans, ≥2 à <5 ans, ≥5 à <15 ans et ≥15 ans et entoure le sexe et le groupe d'âge pour lequel Q4 a été completée; passer la question 4 s'il n'y a aucun membre féminin/masculin du ménage d'âge <2 ans/ ≥2 à <5 ans/ ≥5 à <15 ans/ ≥15 ans.
- Indiquer une réponse pour chaque choix de soins de santé pour chaque signe/symptôme; attribuer une note de 1 à 4 (1=1er choix; 2=2ème choix; 3=3ème choix; 4=non choisi); indiquer un code s'il y a lieu en utilisant les listes préparées (utiliser listes préparées individuellement pour chaque site pour obtenir les codes); cocher «Ne sait pas» s'il y a lieu.

For peer review only - http://bmjopen.bmj.com/site/about/guidelines.xhtml

Signe/Symptôme	Choix de soins de santé									
	EDS SETA		Autre EDS		Médecin	Pharmacie	Guérisseur traditionnel	Nulle part Auto-médication	Nulle part Rien	Ne sait pas
	Note	Code	Note	Code	Note	Note	Note	Note	Note	Cocher
**1** [**A**] Fièvre	O=1<br>O=2<br>O=3<br>O=4		O=1<br>O=2<br>O=3<br>O=4		O=1<br>O=2<br>O=3<br>O=4	O=1<br>O=2<br>O=3<br>O=4	O=1<br>O=2<br>O=3<br>O=4	O=1<br>O=2<br>O=3<br>O=4	O=1<br>O=2<br>O=3<br>O=4	O
**2** Fièvre <3 jours (continus)	O=1<br>O=2<br>O=3<br>O=4		O=1<br>O=2<br>O=3<br>O=4		O=1<br>O=2<br>O=3<br>O=4	O=1<br>O=2<br>O=3<br>O=4	O=1<br>O=2<br>O=3<br>O=4	O=1<br>O=2<br>O=3<br>O=4	O=1<br>O=2<br>O=3<br>O=4	O
**3** Fièvre ≥3 jours (continus)	O=1<br>O=2<br>O=3<br>O=4		O=1<br>O=2<br>O=3<br>O=4		O=1<br>O=2<br>O=3<br>O=4	O=1<br>O=2<br>O=3<br>O=4	O=1<br>O=2<br>O=3<br>O=4	O=1<br>O=2<br>O=3<br>O=4	O=1<br>O=2<br>O=3<br>O=4	O
**4** Frissons Tremblements	O=1<br>O=2<br>O=3<br>O=4		O=1<br>O=2<br>O=3<br>O=4		O=1<br>O=2<br>O=3<br>O=4	O=1<br>O=2<br>O=3<br>O=4	O=1<br>O=2<br>O=3<br>O=4	O=1<br>O=2<br>O=3<br>O=4	O=1<br>O=2<br>O=3<br>O=4	O
**5** Convulsion	O=1<br>O=2<br>O=3<br>O=4		O=1<br>O=2<br>O=3<br>O=4		O=1<br>O=2<br>O=3<br>O=4	O=1<br>O=2<br>O=3<br>O=4	O=1<br>O=2<br>O=3<br>O=4	O=1<br>O=2<br>O=3<br>O=4	O=1<br>O=2<br>O=3<br>O=4	O
**6** Perte de poids	O=1<br>O=2<br>O=3<br>O=4		O=1<br>O=2<br>O=3<br>O=4		O=1<br>O=2<br>O=3<br>O=4	O=1<br>O=2<br>O=3<br>O=4	O=1<br>O=2<br>O=3<br>O=4	O=1<br>O=2<br>O=3<br>O=4	O=1<br>O=2<br>O=3<br>O=4	O
**7** Déshydratation	O=1<br>O=2<br>O=3<br>O=4		O=1<br>O=2<br>O=3<br>O=4		O=1<br>O=2<br>O=3<br>O=4	O=1<br>O=2<br>O=3<br>O=4	O=1<br>O=2<br>O=3<br>O=4	O=1<br>O=2<br>O=3<br>O=4	O=1<br>O=2<br>O=3<br>O=4	O
**8** Malaise Faiblesse/ Fatigue	O=1<br>O=2<br>O=3<br>O=4		O=1<br>O=2<br>O=3<br>O=4		O=1<br>O=2<br>O=3<br>O=4	O=1<br>O=2<br>O=3<br>O=4	O=1<br>O=2<br>O=3<br>O=4	O=1<br>O=2<br>O=3<br>O=4	O=1<br>O=2<br>O=3<br>O=4	O
**9** Douleurs (muscles/articulations /os)	O=1<br>O=2<br>O=3<br>O=4		O=1<br>O=2<br>O=3<br>O=4		O=1<br>O=2<br>O=3<br>O=4	O=1<br>O=2<br>O=3<br>O=4	O=1<br>O=2<br>O=3<br>O=4	O=1<br>O=2<br>O=3<br>O=4	O=1<br>O=2<br>O=3<br>O=4	O
**10** Maux de tête	O=1<br>O=2<br>O=3<br>O=4		O=1<br>O=2<br>O=3<br>O=4		O=1<br>O=2<br>O=3<br>O=4	O=1<br>O=2<br>O=3<br>O=4	O=1<br>O=2<br>O=3<br>O=4	O=1<br>O=2<br>O=3<br>O=4	O=1<br>O=2<br>O=3<br>O=4	O
**11** Vertiges/ Confusion Perte de connaissance	O=1<br>O=2<br>O=3<br>O=4		O=1<br>O=2<br>O=3<br>O=4		O=1<br>O=2<br>O=3<br>O=4	O=1<br>O=2<br>O=3<br>O=4	O=1<br>O=2<br>O=3<br>O=4	O=1<br>O=2<br>O=3<br>O=4	O=1<br>O=2<br>O=3<br>O=4	O
**12** Troubles de la pression artérielle	O=1<br>O=2<br>O=3<br>O=4		O=1<br>O=2<br>O=3<br>O=4		O=1<br>O=2<br>O=3<br>O=4	O=1<br>O=2<br>O=3<br>O=4	O=1<br>O=2<br>O=3<br>O=4	O=1<br>O=2<br>O=3<br>O=4	O=1<br>O=2<br>O=3<br>O=4	O
**13** Troubles cardiaques	O=1<br>O=2<br>O=3<br>O=4		O=1<br>O=2<br>O=3<br>O=4		O=1<br>O=2<br>O=3<br>O=4	O=1<br>O=2<br>O=3<br>O=4	O=1<br>O=2<br>O=3<br>O=4	O=1<br>O=2<br>O=3<br>O=4	O=1<br>O=2<br>O=3<br>O=4	O

For peer review only - http://bmjopen.bmj.com/site/about/guidelines.xhtml

Signe/Symptôme	Choix de soins de santé									
	EDS SETA		Autre EDS		Médecin	Pharmacie	Guérisseur traditionnel	Nulle part Auto-médication	Nulle part Rien	Ne sait pas
	Note	Code	Note	Code	Note	Note	Note	Note	Note	Cocher
**14** Nausée Vomissements	○=1<br>○=2<br>○=3<br>○=4		○=1<br>○=2<br>○=3<br>○=4		○=1<br>○=2<br>○=3<br>○=4	○=1<br>○=2<br>○=3<br>○=4	○=1<br>○=2<br>○=3<br>○=4	○=1<br>○=2<br>○=3<br>○=4	○=1<br>○=2<br>○=3<br>○=4	○
**15** Diarrhée (liquide/sanglante/ mucopurulente)	○=1<br>○=2<br>○=3<br>○=4		○=1<br>○=2<br>○=3<br>○=4		○=1<br>○=2<br>○=3<br>○=4	○=1<br>○=2<br>○=3<br>○=4	○=1<br>○=2<br>○=3<br>○=4	○=1<br>○=2<br>○=3<br>○=4	○=1<br>○=2<br>○=3<br>○=4	○
**16** Douleur abdominale Douleur intestinale	○=1<br>○=2<br>○=3<br>○=4		○=1<br>○=2<br>○=3<br>○=4		○=1<br>○=2<br>○=3<br>○=4	○=1<br>○=2<br>○=3<br>○=4	○=1<br>○=2<br>○=3<br>○=4	○=1<br>○=2<br>○=3<br>○=4	○=1<br>○=2<br>○=3<br>○=4	○
**17** Perte de sang/ Hémorragie (interne)	○=1<br>○=2<br>○=3<br>○=4		○=1<br>○=2<br>○=3<br>○=4		○=1<br>○=2<br>○=3<br>○=4	○=1<br>○=2<br>○=3<br>○=4	○=1<br>○=2<br>○=3<br>○=4	○=1<br>○=2<br>○=3<br>○=4	○=1<br>○=2<br>○=3<br>○=4	○
**18** Perte de sang/ Hémorragie (externe)	○=1<br>○=2<br>○=3<br>○=4		○=1<br>○=2<br>○=3<br>○=4		○=1<br>○=2<br>○=3<br>○=4	○=1<br>○=2<br>○=3<br>○=4	○=1<br>○=2<br>○=3<br>○=4	○=1<br>○=2<br>○=3<br>○=4	○=1<br>○=2<br>○=3<br>○=4	○
**19** Éternuements Nez qui coule	○=1<br>○=2<br>○=3<br>○=4		○=1<br>○=2<br>○=3<br>○=4		○=1<br>○=2<br>○=3<br>○=4	○=1<br>○=2<br>○=3<br>○=4	○=1<br>○=2<br>○=3<br>○=4	○=1<br>○=2<br>○=3<br>○=4	○=1<br>○=2<br>○=3<br>○=4	○
**20** Toux	○=1<br>○=2<br>○=3<br>○=4		○=1<br>○=2<br>○=3<br>○=4		○=1<br>○=2<br>○=3<br>○=4	○=1<br>○=2<br>○=3<br>○=4	○=1<br>○=2<br>○=3<br>○=4	○=1<br>○=2<br>○=3<br>○=4	○=1<br>○=2<br>○=3<br>○=4	○
**21** Respiration rapide/ essoufflement	○=1<br>○=2<br>○=3<br>○=4		○=1<br>○=2<br>○=3<br>○=4		○=1<br>○=2<br>○=3<br>○=4	○=1<br>○=2<br>○=3<br>○=4	○=1<br>○=2<br>○=3<br>○=4	○=1<br>○=2<br>○=3<br>○=4	○=1<br>○=2<br>○=3<br>○=4	○
**22** Difficultés respiratoires graves	○=1<br>○=2<br>○=3<br>○=4		○=1<br>○=2<br>○=3<br>○=4		○=1<br>○=2<br>○=3<br>○=4	○=1<br>○=2<br>○=3<br>○=4	○=1<br>○=2<br>○=3<br>○=4	○=1<br>○=2<br>○=3<br>○=4	○=1<br>○=2<br>○=3<br>○=4	○
**23** Inflammation de la peau (éruptions cutanées/ rougeurs/pus/ démangeaisons)	○=1<br>○=2<br>○=3<br>○=4		○=1<br>○=2<br>○=3<br>○=4		○=1<br>○=2<br>○=3<br>○=4	○=1<br>○=2<br>○=3<br>○=4	○=1<br>○=2<br>○=3<br>○=4	○=1<br>○=2<br>○=3<br>○=4	○=1<br>○=2<br>○=3<br>○=4	○
**24** Gonflement/ Œdème	○=1<br>○=2<br>○=3<br>○=4		○=1<br>○=2<br>○=3<br>○=4		○=1<br>○=2<br>○=3<br>○=4	○=1<br>○=2<br>○=3<br>○=4	○=1<br>○=2<br>○=3<br>○=4	○=1<br>○=2<br>○=3<br>○=4	○=1<br>○=2<br>○=3<br>○=4	○
**25** Jaunisse	○=1<br>○=2<br>○=3<br>○=4		○=1<br>○=2<br>○=3<br>○=4		○=1<br>○=2<br>○=3<br>○=4	○=1<br>○=2<br>○=3<br>○=4	○=1<br>○=2<br>○=3<br>○=4	○=1<br>○=2<br>○=3<br>○=4	○=1<br>○=2<br>○=3<br>○=4	○

For peer review only

Site ___ ___ ___ Sous-zone ___ ___ ___ Numéro d'habitation ___ ___ ___ ___ Identifiant de l'enquêteur ___ __

FORMULAIRE 4: ÉVALUATION DE L'UTILISATION GÉNÉRIQUE DES SOINS DE SANTÉ GLOBAUX, STRATIFIÉE SELON L'ÂGE ET LE SEXE
Partie B – Facteurs associés aux soins de santé/Vaccination/Perçéption de la maladie

DÉPLACEMENT À L'ÉTABLISSEMENT DE SANTÉ

1 Au cas où un établissement de santé (EDS) a été noté comme 1er choix de soins à la question 4A (fièvre), quel est habituellement le moyen de transport principal, le temps de trajet (selon le moyen de transport), le coût du trajet (selon le moyen de transport) et la distance du trajet (selon le moyen de transport) pour s'y rendre ?

Instructions : Indiquer uniquement un code par catégorie, sexe ainsi que groupe d'âge. Passer cette question s'il n'y a pas de membre féminin/masculin d'âge <2 ans/≥2 à <5 ans/≥5 à <15 ans/≥15 ans dans ce ménage (voir également questions 2 et 3 de ce formulaire Partie A). Passer cette question si aucun EDS n'a été noté comme 1er choix de soins à la question 4A pour les membres masculins/féminins d'âge <2 ans/≥2 à <5 ans/≥5 à <15 ans/≥15 ans. Indiquer le temps de trajet en minute(s) [min]/ heure(s) [h], le coût total en USD, y compris les cadeaux éventuels, les aides financières et les dons, et la distance totale en mètre(s) [m]/kilomètre(s) [km].

Membre féminin				Membre masculin			
<2 ans	≥2 à <5 ans	≥5 à <15 ans	≥15 ans	<2 ans	≥2 à <5 ans	≥5 à <15 ans	≥15 ans
**1.1 Moyen de transport**							
Code ____	Code ____	Code ____	Code ____	Code ____	Code ____	Code ____	Code ____
[1] Private transportation (i.e. motorbike, car, bus)<br>[2] Public transportation (i.e. motorbike, car, bus)				[3] Walking<br>[4] Bicycle	[5] Other, specify ________<br>[99] Don't know		[98] No response
**1.2 Temps de trajet**							
Code ____	Code ____	Code ____	Code ____	Code ____	Code ____	Code ____	Code ____
[1] <30 min<br>[2] ≥30 à <60 min		[3] ≥1.0 à <2.0 h<br>[4] ≥2.0 à <3.0 h		[5] ≥3.0 à <4.0 h<br>[6] ≥4.0h		[99] Ne sait pas<br>[98] Pas de réponse	
**1.3 Coût du transport**							
Code ____	Code ____	Code ____	Code ____	Code ____	Code ____	Code ____	Code ____
[1] <1.0 USD<br>[2] ≥1.0 à <3.0 USD		[3] ≥3.0 à <5.0 USD<br>[4] ≥5.0 à <10.0 USD		[5] ≥10.0 USD		[99] Ne sait pas<br>[98] Pas de réponse	
**1.4 Distance du trajet**							
Code ____	Code ____	Code ____	Code ____	Code ____	Code ____	Code ____	Code ____
[1] <500 m<br>[2] ≥500 m à <1.0 km		[3] ≥1.0 à <2.0 km<br>[4] ≥2.0 à <3.0 km		[5] ≥3.0 à <4.0 km<br>[6] ≥4.0 km		[99] Ne sait pas<br>[98] Pas de réponse	

ASSURANCE

2 Pour quels membres de ce ménage l'assurance couvre-t-elle les dépenses de santé ?

Instructions : Indiquer un code par catégorie. Passer cette question s'il n'y a aucun membre d'âge <2 ans/≥2 à <5 ans/≥5 à <15 ans/≥15 ans dans ce ménage (voir questions 2 et 3 de ce formulaire partie A).

<2 ans	≥2 à <5 ans	≥5 à <15 ans	≥15 ans
Code ____________	Code ____________	Code ____________	Code ____________
[1] Oui	[2] Non	[99] Ne sait pas	[98] Pas de réponse

3 Quels types de dépenses de santé sont habituellement couverts par l'assurance pour les membres de ce ménage ?

Instructions : Indiquer uniquement un code par catégorie et par groupe d'âge. Passer cette question s'il n'y a aucun membre d'âge <2 ans/≥2 à <5 ans/≥5 à <15 ans/≥15 ans dans ce ménage (voir également les questions 2 et 3 de ce formulaire partie

For peer review only - http://bmjopen.bmj.com/site/about/guidelines.xhtml

A). Passer cette question s'il n'y a aucune assurance qui couvre les dépenses de santé des membres d'âge <2 ans/≥2 à <5 ans/≥5 à <15 ans/≥15 ans.

	<2 ans	≥2 à <5 ans	≥5 à <15 ans	≥15 ans
	[1] Oui réponse	[2] Non	[99] Ne sait pas	[98] Pas de
**1** Inscription	Code _______	Code _______	Code _______	Code _______
**2** Consultation/Visite chez le médecin	Code _______	Code _______	Code _______	Code _______
**3** Examen physique - général	Code _______	Code _______	Code _______	Code _______
**4** Examen physique – spécifique, préciser________	Code _______	Code _______	Code _______	Code _______
**5** Diagnostics - diagnostics généraux de laboratoire	Code _______	Code _______	Code _______	Code _______
**6** Diagnostics – diagnostics spécifiques de laboratoire, préciser___________	Code _______	Code _______	Code _______	Code _______
**7** Diagnostics Imagerie générale	Code _______	Code _______	Code _______	Code _______
**8** Diagnostics Imagerie spécifique, préciser___________	Code _______	Code _______	Code _______	Code _______
**9** Diagnostics Autre, préciser_________	Code _______	Code _______	Code _______	Code _______
**10** Traitement Médication générale	Code _______	Code _______	Code _______	Code _______
**11** Traitement Médication spécifique, préciser_________	Code _______	Code _______	Code _______	Code _______
**12** Traitement Autre médication, préciser___________	Code _______	Code _______	Code _______	Code _______
**13** Traitement Interventions (autres que chirurgie), préciser________	Code _______	Code _______	Code _______	Code _______
**14** Traitement Chirurgie	Code _______	Code _______	Code _______	Code _______
**15** Hospitalisation <7 jours	Code _______	Code _______	Code _______	Code _______
**16** Hospitalisation ≥7 jours	Code _______	Code _______	Code _______	Code _______
**17** Services d'urgence	Code _______	Code _______	Code _______	Code _______
**18** Soins prénatals	Code _______	Code _______	Code _______	Code _______
**19** Soins postnatals	Code _______	Code _______	Code _______	Code _______
**20** Médecine préventive	Code _______	Code _______	Code _______	Code _______
**21** Vaccination	Code _______	Code _______	Code _______	Code _______
**22** Soins à domicile (personnes âgées, handicapés, malades chroniques/à long terme)	Code _______	Code _______	Code _______	Code _______

For peer review only - http://bmjopen.bmj.com/site/about/guidelines.xhtml

**23** Thérapie de soutien (physiothérapie, ergothérapie, réhabilitation)	Code _______	Code _______	Code _______	Code _______

ETHNOGRAPHIE

4 À quelle religion/groupe ethnique/tribu appartiennent les membres de ce ménage ?

Instructions : Donner une réponse par groupe d'âge uniquement. Passer cette question s'il n'y a aucun membre d'âge>2 ans/≥2 à <5 ans/≥5 à <15 ans/≥15 ans dans ce ménage (voir également questions 2 et 3 de ce formulaire Partie A).

	<2 ans	≥2 to <5 ans	≥5 to <15 ans	≥15 ans
Religion/Groupe ethnique/Tribu	_____________	_____________	_____________	_____________
	O 99=Ne sait pas<br>O 98=Pas de réponse	O 99=Ne sait pas<br>O 98=Pas de réponse	O 99=Ne sait pas<br>O 98=Pas de réponse	O 99=Ne sait pas<br>O 98=Pas de réponse

5 La religion/le groupe ethnique/la tribu comme indiqué à la question 4 influence-t-elle/il les différents aspects de l'utilisation des soins de santé pour les membres de ce ménage ?

Instructions : Indiquer un code par catégorie et groupe d'âge uniquement. Passer cette question s'il y a un membre d'âge >2 ans/≥2 à <5 ans/≥5 à <15 ans/≥15 ans dans ce ménage (voir également questions 2 t 3 de ce formulaire Partie A).

	<2 ans	≥2 à <5 ans	≥5 to <15 ans	≥15 ans
	[1] Oui	[2] Non	[99] Ne sait pas	[98] pas réponse
**1** Visite dans un établissement de santé (hôpital, centre de soins, poste de santé)	Code _____	Code _______	Code _______	Code _____
**2** Visite chez un médecin	Code _____	Code _______	Code _______	Code _____
**3** Visite dans une pharmacie	Code _____	Code _______	Code _______	Code _____
**4** Visite chez un guérisseur traditionnel	Code _____	Code _______	Code _______	Code _____
**5** Décision d'automédication	Code _____	Code _______	Code _______	Code _____
**6** Décision de non traitement	Code _____	Code _______	Code _______	Code _____
**7** Examen physique - général	Code _____	Code _______	Code _______	Code _____
**8** Examen physique – spécifique, préciser_______	Code _____	Code _______	Code _______	Code _____
**9** Prélèvements d'échantillons (sang, urine, selles, frottis, écouvillons, liquide cérébro-spinal)	Code _____	Code _______	Code _______	Code _____
**10** Diagnostics - diagnostics généraux de laboratoire	Code _____	Code _______	Code _______	Code _____
**11** Diagnostics – diagnostics spécifiques de laboratoire, préciser__________	Code _____	Code _______	Code _______	Code _____
**12** Diagnostics Imagerie générale	Code _____	Code _______	Code _______	Code _____
**13** Diagnostics Imagerie spécifique, préciser__________	Code _____	Code _______	Code _______	Code _____
**14** Diagnostics Autre, préciser_________	Code _____	Code _______	Code _______	Code _____
**15** Traitement Médication générale	Code _____	Code _______	Code _______	Code _____
**16** Traitement Médication spécifique, préciser_________	Code _____	Code _______	Code _______	Code _____

For peer review only

For peer review only - http://bmjopen.bmj.com/site/about/guidelines.xhtml

**17** Traitement<br>Autre médication, préciser___________	Code _____	Code _______	Code _______	Code _____
**18** Traitement<br>Interventions (autres que chirurgie), préciser________	Code _____	Code _______	Code _______	Code _____
**19** Traitement<br>Chirurgie	Code _____	Code _______	Code _______	Code _____
**20** Hospitalisation<br><7 jours	Code _____	Code _______	Code _______	Code _____
**21** Hospitalisation<br>≥7 jours	Code _____	Code _______	Code _______	Code _____
**22** Services d'urgence	Code _____	Code _______	Code _______	Code _____
**23** Soins prénatals	Code _____	Code _______	Code _______	Code _____
**24** Soins postnatals	Code _____	Code _______	Code _______	Code _____
**25** Médecine préventive	Code _____	Code _______	Code _______	Code _____
**26** Vaccination	Code _____	Code _______	Code _______	Code _____
**27** Soins à domicile (personnes âgées, handicapés, malades chroniques/à long terme)	Code _____	Code _______	Code _______	Code _____
**28** Thérapie de soutien (physiothérapie, ergothérapie, réhabilitation)	Code _____	Code _______	Code _______	Code _____

VACCINATION

6 Comment les enfants de ce ménage ont-ils été nourris pendant les 6 premiers mois de leur vie ?

Instructions : Passer cette question s'il n'y a pas d'enfants de sexe masculin ou féminin dans ce ménage (voir également questions 2 et 3 de ce formulaire partie A).

O 1=Lait maternisé exclusivement
O 2=Lait maternisé et autres aliments, préciser ______
O 3=Allaitement et autres aliments, préciser ________
O 4=Lait maternisé et allaitement
O 5=Allaitement exclusivement
O 6=Autre, préciser _____
O 99=Ne sait pas
O 98=Pas de réponse

7 Quelle source d'approvisionnement en eau était-elle habituellement utilisée pour préparer le lait maternisé ou le lait maternisé et d'autres aliments comme indiqué à la question 6 pour les enfants de ce ménage ?

Instructions : Passer cette question s'il n'y a pas d'enfants dans ce ménage (voir également les questions 2 et 3 de ce formulaire Partie A).

O 1=Eau du robinet (à l'intérieur de l'habitation, privé)
O 2=Eau du robinet (à l'extérieur de l'habitation, arrière- cour/cour, privé)
O 3=Eau du robinet (à l'extérieur de l'habitation, public, partagé)
O 4=Borne-fontaine/Eau courante (à l'intérieur de l'habitation, privé)
O 5=Borne-fontaine/Eau courante (à l'extérieur de l'habitation, arrière- cour/cour, privé)
O 6=Borne-fontaine/Eau courante (à l'extérieur de l'habitation, public, partagé)
O 7=Camion-citerne (public, partagé)
O 8=Eau en bouteille
O 9=Eaux de surface (lac, étang, rivière, ruisseau)
O 10=Eaux de surface (canal, canal d'irrigation)
O 11=Eau de source
O 12=Eau de pluie
O 13=Puits/Puits de forage (ouvert/non couvert/non protégé)
O 14=Puits/Puits de forage (couvert/protégé)
O 15=NO FORMULA/OTHER FEEDS PREPARED
O 16=Autre, préciser _____________ O 99=Ne sait pas O 98=Pas de réponse

For peer review only - http://bmjopen.bmj.com/site/about/guidelines.xhtml

8 Est-ce que les enfants de ce ménage ont été vaccinés depuis leur naissance, y compris les vaccins de naissance ?

Instructions : Indiquer un code par catégorie et par enfant uniquement. Passer cette question s'il n'y a aucun enfant dans ce ménage (voir également les questions 2 et 3 de ce formulaire PartieA). Se référer à la question 9 « Formulaire 3 Partie A – Données démographiques/Informations générales sur le ménage » et indiquer les initiales/identificateur du/des membre(s).

Enfant 1<br>Initiales/Identifiant :<br>____	Enfant 2<br>Initiales/Identifiant :<br>____	Enfant 3<br>Initiales/Identifiant :<br>____	Enfant 4<br>Initiales/Identifiant :<br>____
[1] OUI, vacciné [5] Non, manque de vaccins [9] Non, EDS débordé<br>[2] Non, parent/tuteur débordé [6] Non, enfant trop jeune/vieux [10] Non, enfant malade<br>[3] Non, parent/tuteur a décidé que pas nécessaire [7] Non, parent/tuteur a refusé/trop inquiet [11] Non, pas de carte de vaccination<br>[4] Non, ne sait pas où aller [8] Non, Autre, préciser ____________ [99] Don't know [98] No response			
**8.1 Diphtérie et tétanos et coqueluche et *Haemophilus influenza* et vaccin contre l'hépatite B / DTwPHibHepB** (par exemple 6/8, 10/12, 14/16 semaines)			
Code __________	Code __________	Code __________	Code __________
**8.2 Anatoxine tétanique (TT)** (par exemple, 1er contact, grossesse, 1, 6, 12/24 mois)			
Code __________	Code __________	Code __________	Code __________
**8.3 Anatoxine tétanique et diphtérique (TD)** (par exemple, grossesse avec un premier contact, 1, 6, 12 mois)			
Code __________	Code __________	Code __________	Code __________
**8.4 Tuberculose / BCG** (à la naissance)			
Code __________	Code __________	Code __________	Code __________
**8.5 Rotavirus / RV** (par exemple 6/8, 10/12, 16 semaines)			
Code __________	Code __________	Code __________	Code __________
**8.6 Rougeole / rougeole et rubéole (MR)** (par exemple 9, 15/18 mois)			
Code __________	Code __________	Code __________	Code __________
**8.7 Polio / VPO** (par exemple naissance, 6/8, 10/12, 14/16 semaines)			
Code __________	Code __________	Code __________	Code __________
**8.8 Pneumocoque / PCV** (par exemple 6/8, 10/12, 14/16 semaines)			
Code __________	Code __________	Code __________	Code __________
**8.9 Fièvre jaune (YF)** (par exemple 9 mois)			
Code __________	Code __________	Code __________	Code __________
**8.10 Autres vaccins**			
Préciser, __________	Préciser, __________	Préciser, __________	Préciser, __________
Préciser, __________	Préciser, __________	Préciser, __________	Préciser, __________

For peer review only - http://bmjopen.bmj.com/site/about/guidelines.xhtml

Préciser, ___________	Préciser, ___________	Préciser, ___________	Préciser, ___________
Préciser, ___________	Préciser, ___________	Préciser, ___________	Préciser, ___________

For peer review only

PERCEPTION DE LA MALADIE

9 Connaissance, perception, causes et prévention des maladies infectieuses communes

Maladie	**9.1 Maladie connue des membres du ménage ?**<br>[1] Oui<br>[2] Non<br>[99] Ne sait pas<br>[98] Pas de réponse	**9.2 Perception de la maladie par les membres du ménage**<br>*Instruction :* Décrire brièvement si elle est connue.	**9.3 Causes de la maladie connue des membres du ménage ?**<br>*Instructions :* Indiquer tous les codes (liste A) qui s'appliquent; plus d'une réponse est possible	**9.4 Mesures pour prévenir la maladie connues des membres du ménage ?**<br>*Instructions :* Indiquer tous les codes (liste B) qui s'appliquent ; plus d'une réponse est possible.	**9.5 Mesures pour prévenir la maladie utilisées par les membres du foyer ?**<br>*Instructions :* Indiquer tous les codes (liste B) qui s'appliquent ; il peut y avoir plus d'une réponse.
**A.** Fièvre typhoïde	Code __________		Code __________	Code __________	Code __________
**B.** Choléra	Code __________		Code __________	Code __________	Code __________
**C.** Paludisme	Code __________		Code __________	Code __________	Code __________
**D.** Grippe	Code __________		Code __________	Code __________	Code __________
**E.** Méningite	Code __________		Code __________	Code __________	Code __________
**F.** Hépatite E	Code __________		Code __________	Code __________	Code __________
**G.** Tuberculose	Code __________		Code __________	Code __________	Code __________
**H.** VIH/SIDA	Code __________		Code __________	Code __________	Code __________

Liste A: Causes possibles	Liste B: Mesures préventives possibles
[1] Consommation d'alcool	[1] Pas de consommation d'alcool

For peer review only - http://bmjopen.bmj.com/site/about/guidelines.xhtml

[2] Temps/sorcellerie<br>[3] Piqûre de moustique/d'insecte<br>[4] Morsure de chien<br>[5] Boire de l'eau non traitée de surface/de source (lac, étang, rivière, ruisseau, canal, canal d'irrigation)<br>[6] Contact physique étroit/baiser/embrassade<br>[7] Manger des produits alimentaires crus comme des légumes/de la salade, des fruits, des produits laitiers, des œufs, de la viande ou du poisson<br>[8] Ne pas se laver les mains correctement en préparant la nourriture/les repas<br>[9] Ne pas se laver les mains correctement avant/après être allé aux toilettes<br>[10] Contact avec du sang humain (injection, transfusion, chirurgie)<br>[11] Consommation de sang animal cru<br>[12] Autre, préciser ____________________<br>[99] Ne sait pas<br>[98] Pas de réponse	[2] Dormir sous une moustiquaire<br>[3] Utiliser des sprays insecticides/anti-moustiques<br>[4] Maintenir la maison et ses environs immédiats propres<br>[5] Avoir une pratique correcte de l'élimination des déchets humains et domestiques<br>[6] Éviter le contact rapproché avec des chiens<br>[7] Traiter l'eau (faire bouillir, filtration, désinfection solaire, sédimentation, chloration)<br>[8] Ne pas cuisiner/préparer la nourriture/les repas avec de l'eau non traitée<br>[9] Ne pas laver les produits alimentaires à consommer crus avec de l'eau non traitée<br>[10] Faire bouillir/chauffer à nouveau les restes/les aliments/repas préparés avant de les consommer<br>[11] Ne pas manger des produits alimentaires crus comme légumes/salade, fruits, produits laitiers, œufs, viande ou poisson<br>[12] Couvrir les produits alimentaires/repas<br>[13] Protéger les produits alimentaires/repas contre les mouches<br>[14] Éviter la nourriture/les boissons des vendeurs de rue<br>[15] Éviter le contact rapproché avec les personnes malades<br>[16] Pas de contact physique étroit/baisers/embrassades<br>[17] Se couvrir le nez/la bouche en éternuant/toussant<br>[18] Pas de contact/consommation de sang humain/animal<br>[19] Avoir une bonne hygiène des mains en préparant la nourriture/les repas (se laver les mains avec du savon et de l'eau, se laver les mains par frottement)<br>[20] Avoir une bonne hygiène des mains avant/après être allé aux toilettes (se laver les mains avec du savon et de l'eau, se laver les mains par frottement)<br>[21] Autre, préciser ____________________<br>[99] Ne sait pas<br>[98] Pas de réponse

For peer review only - http://bmjopen.bmj.com/site/about/guidelines.xhtml

10 Pour quels membres de ce ménage les mesures préventives listées à la question 9.5 contre la fièvre typhoïde sont-elles utilisées ?

Instructions : Cocher plusieurs cases si nécessaire ; il peut y avoir plus d'une réponse.

O 1=<2 ans
O 2=≥2 à < 5 ans
O 3=≥5 à <15 ans
O 4=≥15 ans
O 5=PAS UTILISÉ
O 99=Ne sait pas
O 98=Pas de réponse

11 Nommer les membres du ménage pour lesquels une fièvre typhoïde a été diagnostiquée dans les 3 derniers mois. Quels éléments de diagnostic ont été utilisés pour confirmer la fièvre typhoïde?

Instructions : Indiquer plusieurs codes si nécessaire pour chaque membre du ménage ; il peut y avoir plus d'une réponse.

Membre 1<br>Initiales/Identifiant: __	Membre 2<br>Initiales/Identifiant: __	Membre 3<br>Initiales/Identifiant: __	Membre 4<br>Initiales/Identifiant: __
[1] Hémoculture [2] Coproculture [3] Test sanguin/de Widal [4] Autre test, préciser _____________ [5] Pas applicable (pas diagnostiqué avec la fièvre typhoïde) [99] Ne sait pas [98] Pas de réponse			
Code __________	Code __________	Code __________	Code __________

12 (Où les membres de ce ménage indiqués à la question 11 ont-ils été diagnostiqués pour la fièvre typhoïde?) Où les membres de ce ménage ont-ils recherché des soins de santé?

Instructions : Indiquer un code par membre du ménage uniquement.

Membre 1<br>Initiales/Identifiant: __	Membre 2<br>Initiales/Identifiant: __	Membre 3<br>Initiales/Identifiant: __	Membre 4<br>Initiales/Identifiant: __
[1] Établissement de santé, préciser ____________<br>[2] Médecin<br>[3] Nulle part, automédication<br>[4] Guérisseur traditionnel<br>[5] Pharmacien<br>[6] Nulle part, pas de recherche de soins<br>[7] Autre, préciser ____________<br>[8] Pas applicable<br>[99] Ne sait pas<br>[98] Pas de réponse			
Code __________	Code __________	Code __________	Code __________

13 (Les membres de ce ménage pour lesquels une fièvre typhoïde a été diagnostiquée comme indiqué à la question 11 ont-ils reçu un traitement?) Quel traitement ont-ils reçu?

Instructions : Cocher une réponse par membre du ménage uniquement.

Membre 1<br>Initiales/Identifiant: __	Membre 2<br>Initiales/Identifiant: __	Membre 3<br>Initiales/Identifiant: __	Membre 4<br>Initiales/Identifiant: __
O1=Traitement, préciser ______________<br>O 2=Pas applicable<br>O 99=Ne sait pas<br>O 98=Pas de réponse	O1=Traitement, préciser ______________<br>O 2=Pas applicable<br>O 99=Ne sait pas<br>O 98=Pas de réponse	O1=Traitement, préciser ______________<br>O 2=Pas applicable<br>O 99=Ne sait pas<br>O 98=Pas de réponse	O1=Traitement, préciser ______________<br>O 2=Pas applicable<br>O 99=Ne sait pas<br>O 98=Pas de réponse

For peer review only

For peer review only - http://bmjopen.bmj.com/site/about/guidelines.xhtml

Site ___ ___ ___ Sous-zone ___ ___ ___ Numéro d'habitation ___ ___ ___ ___ Identifiant de l'enquêteur ___ __

FORMULAIRE 5: ÉVALUATION DE L'UTILISATION RÉELLE DES SOINS DE SANTÉ GLOBAUX, STRATIFIÉE SELON L'ÂGE ET LE SEXE

Partie A: Évaluation de l'utilisation *réelle* des soins de santé

COMPORTEMENT DE RECHERCHE DE SOINS

1 Consigner ci-dessous l'apparition de signe(s) ou symptôme(s) (voir liste 1) et préciser où (voir liste 2) et comment le(s) membre(s) a/ont recherché les soins de santé durant les 3 derniers mois ?

Instructions : voir « formulaire 3- partie A-INFORMATIONS GENERALES SUR LE MENAGE » pour obtenir les initiaux/identifiants de chacun des membres du ménage. Indiquer seulement un signe/symptome par ligne. Commencer avec une nouvelle ligne pour tous nouveaux soins de santé sélectionnée pour chaque signe/symptome recurrent. Indiquer le nombre des apparitions de chaque signe/symptômes durant les 3 derniers mois. Si une différente option de soins de santé a été sélectionnée pour un signe/symptôme recurrent, indiquer cela dans une nouvelle ligne. Completer s'il vous plaît, un formulaire supplémentaire s'il y a eu plus de 20 signes/symptômes apparus durant les 3 derniers mois.

**Liste 1: Signe/Symptôme**		**Liste 2: Option de soins de santé**
[A] Fièvre<br>[B] Fièvre <3 jours (continus)<br>[C] Fièvre ≥3 jours (continus)<br>[D] Frissons/Tremblements<br>[E] Convulsion<br>[F] Perte de poids<br>[G] Déshydratation<br>[H] Malaise/Faiblees/Fatigue<br>[I] Douleurs (muscles/articulations/os)<br>[J] Maux de tête<br>[K] Vertiges/Confusion/ Perte de connaissance<br>[L] Troubles de la pression sanguine<br>[M] Troubles cardiaques	[N] Nausée/Vomissements<br>[O] Diarrhée (liquide/sanglante/mucopurulente)<br>[P] Douleur abdominale/Douleur intestinale<br>[Q] Perte de sang/Hémorragie (interne)<br>[R] Perte de sang/Hémorragie (externe)<br>[S] Éternuements Nez qui coule<br>[T] Toux<br>[U] Respiration rapide/Essoufflement<br>[V] Difficultés respiratoires graves<br>[W] Inflammation de la peau (éruptions cutanées/ rougeurs/pus/démangeaisons)<br>[X] Gonflement/Œdème<br>[Y] Jaunisse	[1] EDS-SETA, Code ______________<br>[2] Autre EDS Code ______________<br>[3] Médecin<br>[4] Pharmacie<br>[5] Guérisseur traditionnel<br>[6] Nulle part Auto-médication<br>[7] Nulle part Rien

**No.**	**Membre du ménage** Enregistrer les initiaux/identifiants du membre du ménage ; voir formulaire 3 –partie A- INFORMATIONS GENERALES SUR LE MENAGE »	**Signes/ Symptômes** Utiliser la Liste 1 pour sélectionner le code	**Options de soins de santé** Utiliser la Liste 2 pour sélectionner le code	EDS Code	**Fréquence** Indiquer le nombre d'incidences pour le signe/symptôme listé
1					
2					
3					
4					
5					

For peer review only - http://bmjopen.bmj.com/site/about/guidelines.xhtml

6				
7				
8				
9				
10				
11				
12				
13				
14				
15				
16				
17				
18				
19				
20				

For peer review only

For peer review only - http://bmjopen.bmj.com/site/about/guidelines.xhtml

Appendix 4/Supplementary file 4: Geospatial random selection with application of simple, weighted-stratified sampling (examples of Ghana and Madagascar)

Figure 1 Geographic random points generated by ArcGIS for Asante Akim North and Central, Ghana

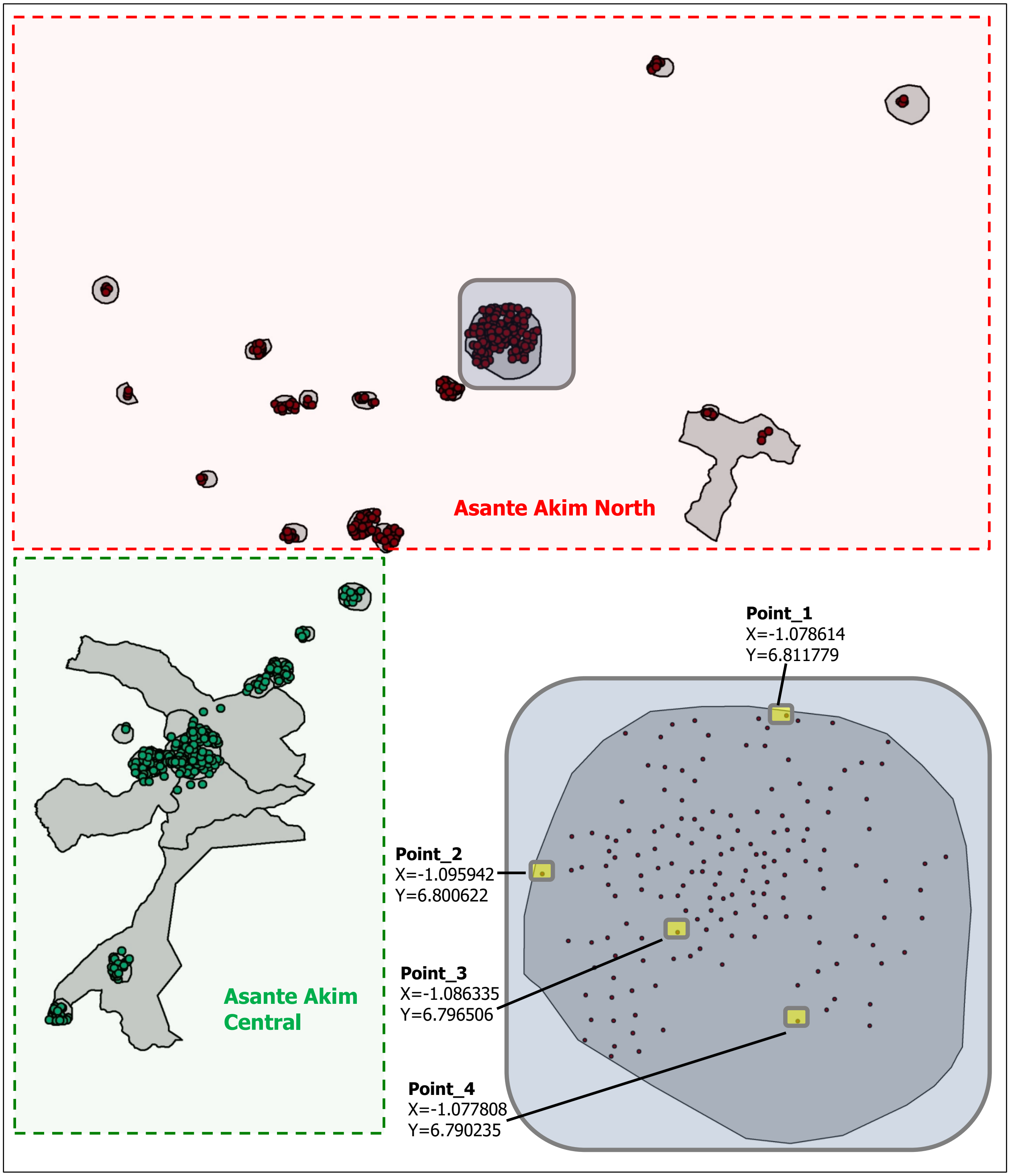

The study area of Asante Akim North (light red rectangle) and Asante Akim Central (light green rectangle) is highlighted in grey. The geographic random points are shown as red and green points for Asante Akim North and Asante Akim Central, respectively. The illustration at the bottom, right highlighted in light blue shows the enlarged area that is indicated as a small central light blue highlighted rectangle. The X- and Y-data of four geographic random points are given as examples; points are indicated as yellow highlighted rectangles. ArcGIS-generated geographic random points converted and visualized in Google Earth Pro® for Asante Akim North and Central, Ghana, can be shared on request.

Figure 2 Geographic random points generated by ArcGIS for Renivohitra/Antananarivo, Madagascar

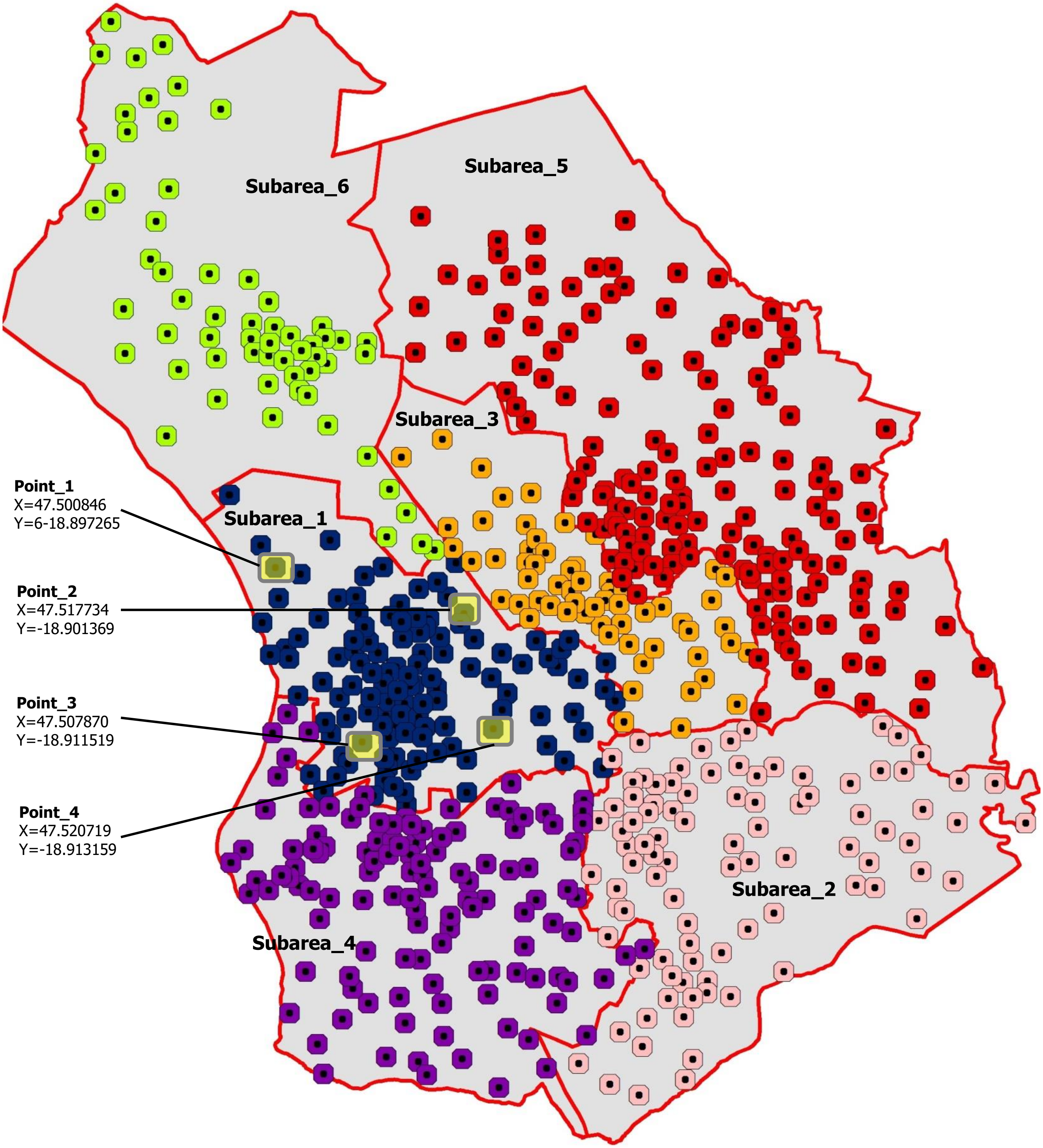

The study area of Renivohitra/Antananarivo, which is subdivided into six administrative subareas, is highlighted in grey. The geographic random points are shown as blue (subarea_1), pink (subarea_2), orange (subarea_3), purple (subarea_4), red (subarea_5) and green (subarea_6) points. The X- and Y-data of four geographic random points are given as examples; points are indicated as yellow highlighted rectangles. ArcGIS-generated geographic random points converted and visualized in Google Earth Pro® for Renivohitra/Antananarivo, Madagascar, can be shared on request.

Appendix 5/Supplementary file 5: System diagram of the data collection and data management platform: the Android application "HPA Collect" and the platform "HPA Web"

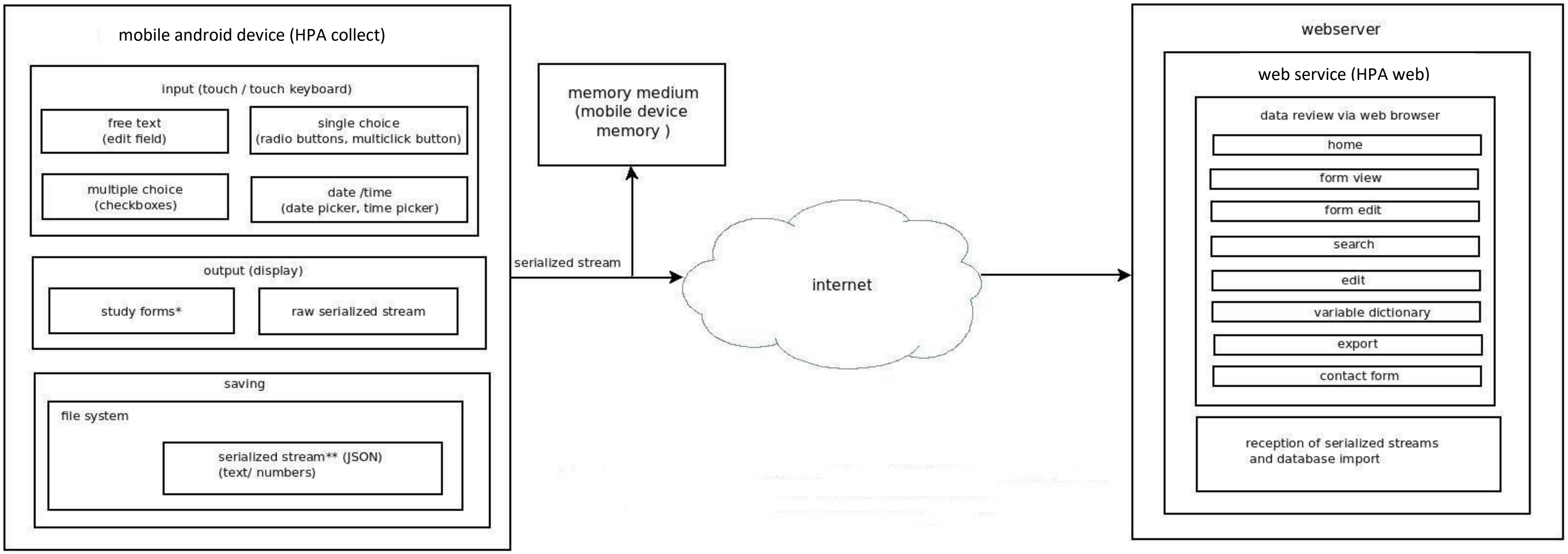

Note. *study forms: Form 1, Form 3 Part A to C, Form 4 Part A to B, Form 5; **serialized stream: two- or more-dimensional data (e.g. a table) is brought to a one-dimensional form; the application uses the JSON (Java Script Object Notation) format.
